# Role of physical performance measures for identifying functional disability among Chinese older adults: Data from the China Health and Retirement Longitudinal Study

**DOI:** 10.1371/journal.pone.0215693

**Published:** 2019-04-18

**Authors:** Li Zhang, Linwen Guo, Huitao Wu, Xiaowen Gong, Junqi Lv, Yanfang Yang

**Affiliations:** 1 West China School of Public Health and West China Fourth Hospital, Sichuan University, Chengdu, Sichuan, China; 2 Medical Big Data Center, People's Liberation Army General Hospital, Beijing, China; 3 Epidemiology and Biostatistics Institute, Tianjin Medical University, Tianjin, China; Universidade Federal de Pelotas, BRAZIL

## Abstract

**Background:**

Functional disability is a common health burden in older adults and follows a hierarchical pattern. Physical performance measures are useful for the objective estimation of functional disability. This study primarily aimed to compare the validity of handgrip strength and gait speed, alone and in combination, for recognizing the functional disability among Chinese older adults. This study also aimed to stratify the functional disability according to the criterion-referenced values of handgrip strength and gait speed.

**Methods:**

We selected 6127 respondents from the 2011 wave of the China Health and Retirement Longitudinal Study. Here, we defined functional disability as needing any help in any items of activities of daily living (ADL) and instrumental activities of daily living (IADL). To assess the validity of physical performance measures alone and in combination for the recognition of functional disability, we conducted the receiver operating characteristic analysis.

**Results:**

Compared with handgrip strength, the gait speed could better discriminate ADL disability and showed a satisfactory discriminant validity (area under the curve ≥ 0.7) in men. However, this finding was not found in the recognition of IADL disability. When combining these two measures, the parallel test showed a high sensitivity with a poor specificity, whereas the serial test showed a perfect specificity with a poor sensitivity.

**Conclusion:**

We developed the hierarchical cut-off values of handgrip strength and gait speed for identifying and stratifying the functional disability among Chinese adults over 60 years old. The speed test was superior to handgrip strength in identifying ADL disability. The parallel tests of those with high sensitivity perhaps could help identify the functional disability. Further work on cost-utility analysis is warranted.

## Introduction

Functional disability, defined as a dependency in activities of daily living (ADL) and/or instrumental activities of daily living (IADL), is a significant and profound health outcome for older adults [1–4]. Functional disability is associated with future falls, cognitive decline, hospitalization, and mortality [5]. IADL are related to functioning independently in a given environment, whereas ADL are essential for self-care in routine activities [4–6]. In most older adults, functional disability presents a hierarchical pattern. They first encounter difficulty in performing IADL, followed by ADL [7–8]. ADL disability represents a relative severity stage in the disablement process. Functional disability can indicate the intrinsic capacity of older adults [9]. Many individuals experience periods of high and stable capacity, declining capacity (they may encounter IADL disability), and a significant loss of capacity (they may experience ADL disability). Interventions may have to be tailored depending on the presence and stage of intrinsic capacity. Therefore, stratifying older people according to the stage of intrinsic capacity trajectory (or functional disability) would likely facilitate the intervention programs to optimize the intrinsic capacity trajectory and achieve the goal of healthy aging. Thus, a valid, simple, and reliable tool for identifying high-risk disability population is critical, particularly in a health care program.

The most widely used measuring instruments for functional disability are ADL and IADL, which are self-reported measuring tools without biometric measures. Furthermore, the physical performance measures are key factors to obtain the objective estimates of the older adults’ functional disability [3]. This importance is mainly due to the large evidence that physical performance measures are more rapid, portable, and less influenced by cultural and educational backgrounds, compared with self-report measures [10, 11, 12]. According to substantial evidence, handgrip strength and gait speed are the “vital signs” of functional disability in the general older population [13–16]. However, their abilities to identify functional disability are seldom examined and compared; these abilities may be highly beneficial to interventions. Meng-Chih Lee et al. reported the cut-off values of handgrip strength and gait speed to best discriminate the IADL disability of 2420 community-dwelling older adults in Taiwan [7]. Wen-Ni Wennie Huang et al. suggested that gait speed and handgrip strength could identify the onset of basic ADL disability over an 18-month period in older adults [17]. AlytOppewal et al. successively discovered that physical fitness, including balance, comfortable and fast gait speed, and muscular endurance, was helpful to early recognize the decline of ADL [18] and IADL [8] in 601 older patients with intellectual disabilities. However, most of the existing studies focus on the discernment role of every single physical performance measure in older adults with IADL or ADL disability, whereas the combined recognition function of these indicators is relatively rarely explored. Additionally, the hierarchical models of physical performance measures for IADL and ADL disabilities are seldom compared.

Thus, this study aimed to evaluate the discrimination power of gait speed and handgrip strength alone or in combination for recognizing the functional disability of a large Chinese older population-based sample. This study also intended to determine the criterion-referenced values of physical performance measures for stratifying the functional disability of older adults, guiding the intervention for older people with functional disability at varying risks.

## Methods

### Setting and participants

We used the collected data from China Health and Retirement Longitudinal Study (CHARLS) [19], a nationally representative longitudinal survey among Chinese community-dwelling residents aged 45 and older and their spouses, offering a wide range of information on socioeconomic status and health circumstances. The CHARLS baseline dataset was performed from 2011 to 2012, involving 150 counties and 450 villages/resident committees in 28 provinces. We selected 17,707 respondents by using a four-staged stratified cluster-sampling method. CHARLS respondents were followed biennially by using a face-to-face computer-assisted personal interview. Physical attributes were measured every 2-year follow-up [20].

**Fig 1:**
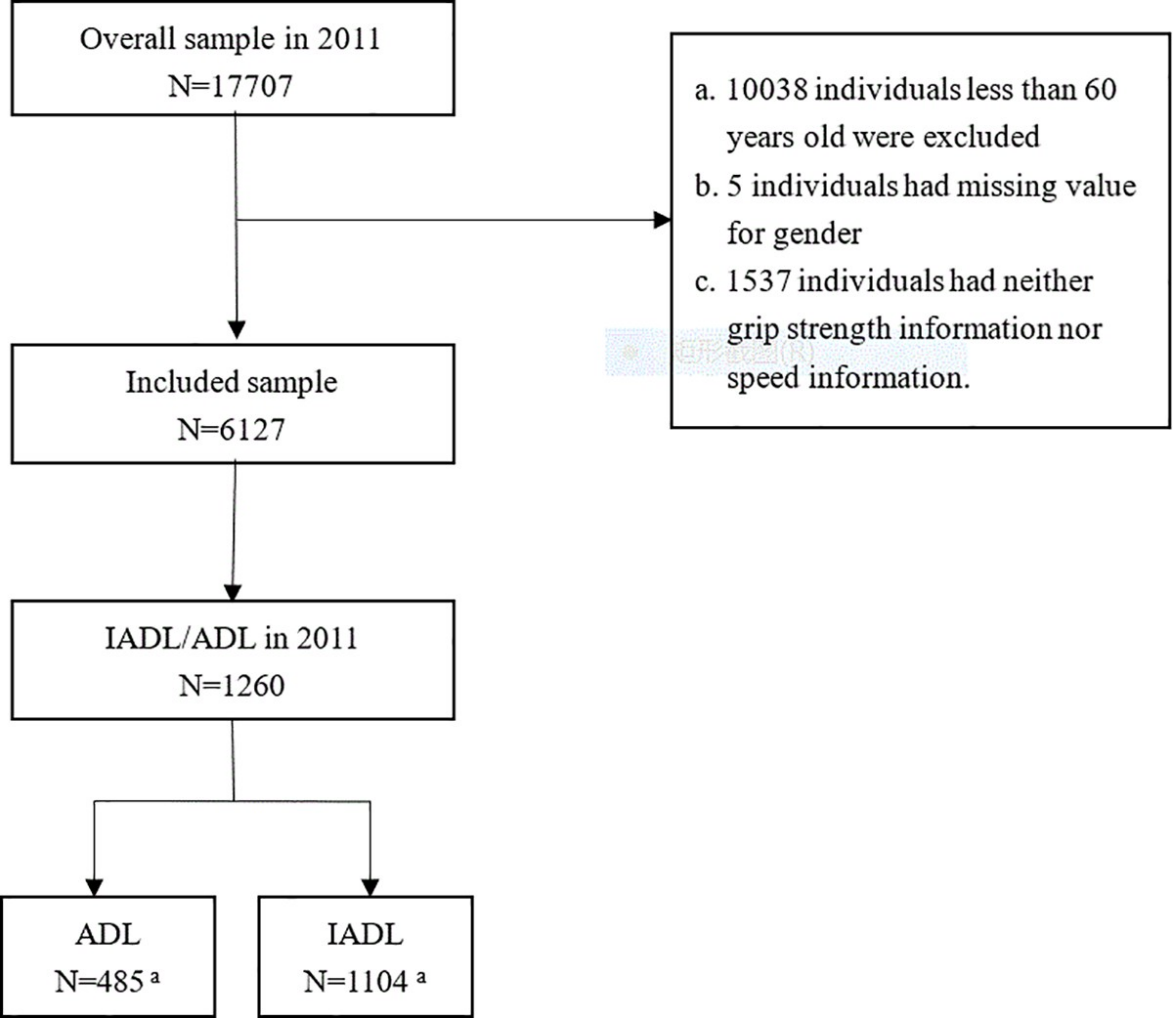
Participants' flow in the study. IADL, instrumental activities of daily living; ADL, activities of daily living. a. 1104 IADL and 485 ADL disabled persons both include 329 persons with IADL and ADL disabilities simultaneously.

For our analysis, we used the 2011 wave of CHARLS data, which included a total of 17,707 subjects. Among them, respondents who are younger than 60 years old, or did not provide consent or had incomplete gender information, or no physical performance measure information at all were excluded from subsequent analyses. Finally, we included 6127 respondents in the final study, comprising 1104 (18.0%) and 485 (7.9%) persons with IADL and ADL disabilities, respectively. The detailed exclusion process is shown in Fig 1.

### Measures

#### Physical performance measures on handgrip strength

Handgrip strength was assessed by a trained examiner using a YuejianTM WL-1000 dynamometer (Nantong Yuejian Physical Measurement Instrument Co., Ltd., Nantong, China) in kilograms [20]. Beginning the test with dominant or nondominant hand, respondents were in standing position, holding the dynamometer at a right angle (90°) for a couple of seconds and then releasing it. Participants were asked to provide maximum effort for the measures. Measurements were demonstrated alternately on each hand twice. Considering that prior or present conditions may affect a person’s grip strength, participants who had surgery on either hand or wrist within the past 6 months or had severe hand pain were excluded from the handgrip strength measurement [21]. The maximum handgrip strength (kg) from all four attempts was used in the analysis [22].

#### Physical performance measures on gait speed

In the test of gait speed, all participants aged 60 years or older without health conditions that may restrict walking (e.g., injury) were eligible for the test. Participants were asked to walk along a straight 2.5-meter flat course twice (there and back) at their usual speed. A stopwatch was used to time how fast the participant could walk [21]. The average speed of two trials was used as a measure of gait speed.

#### Functional disability

The Katz ADL [23] and the Lawton IADL [24] were used to evaluate self-reported functional disability. ADL refer to daily self-care tasks, including taking a bath, eating, getting in and out of bed, dressing, using the toilet, and maintaining continence of urine and feces. Meanwhile, the abilities such as doing housework, cooking, taking medicine, shopping, and taking care of finances, which are required for living independently in the community, were used to assess the IADL. Each answer in CHARLS was divided into four responses as follows: (1) No, I do not have any difficulty, (2) I have difficulty but still can do it, (3) Yes, I have difficulty and need help, and (4) I cannot do it. The respondents who completed all items without any help were classified as ADL- or IADL-independent, whereas these participants who reported needing any help in any items were classified as having ADL or IADL disability [1–3].

#### Demographic characteristics and their covariates

The demographic characteristics included age, gender, household registration system (Hukou), marital status, and education level. Age was divided into four groups at every 5 years: 60 − 64, 65 −69, 70 −74, and older than 75. Gender included male and female. Furthermore, Hukou was categorized into agricultural and nonagricultural. Marital status was divided into two categories, namely, married or cohabiting and another marital status. Finally, education level was allocated into the following five categories: illiterate, primary school and below, middle school, high/vocational school, and college and above.

#### Health behavior status and their covariates

Health behavior status includes body mass index (BMI), social activity frequency, physical activity frequency, and comorbidity condition. BMI is defined as weight divided by height squared and was used with the following WHO cut-off points for Chinese: underweight (BMI < 18.5 kg/m^2^), normal weight (BMI = 18.5 kg/m^2^ to 24 kg/m^2^), and overweight or obese (BMI ≥ 24 kg/m^2^) [25–26]. Social activity frequency was classified into the following three categories: never, not regularly (less than every week), and regularly (more than every week). Moreover, physical activity frequency was classified into three categories according to whether participants met the basic WHO guideline for physical activity in older age (Guaranteeing 150 minutes per week of physical activity at moderate intensity) [9]. Such categories were as follows: never, inadequate, adequate. The participants were asked “Have you been diagnosed with the following conditions by a doctor?” The conditions were as follows: hypertension, dyslipidemia, diabetes or high blood sugar, cancer or malignant tumor, chronic lung diseases, liver disease, heart problems, stroke, kidney disease, digestive diseases, emotional, nervous, or psychiatric problems, memory-related disease, arthritis or rheumatism, and asthma. The number of chronic diseases ≥ 2 was defined as comorbidity condition.

### Statistical analysis

In the descriptive analysis, means and 95% confidence intervals (CI) (mean percent and 95% CI of the percentage for categorical variables) were used to describe the demographics, health behavior status, and physical performance measures of the study sample. The gender-specific receiver operating characteristic (ROC) curves and the area under the curve (AUC) were conducted to assess the discriminating ability of physical performance measures. We listed 50% as the limit of sensitivity and specificity. The optimal cut-off values were obtained from the maximal Youden’s index, calculated as (sensitivity + specificity − 1) [7, 11, 27]. In addition, the validity of combined physical performance measures was evaluated by sensitivity and specificity. Adjusted binary logistic regression model was used for estimating the odds ratio (OR) and 95% CI of physical performance measures on functional disability (dependent variable). We adjusted the demographic characteristics and health behavior status in the binary logistic regression model. The two-sided with p-values ≤0.05 was considered statistically significant. All statistical analyses were performed using the SPSS Statistics version 22 (IBM Corp., Armonk, NY).

## Results

### Demographic characteristics and health-related information of the sample

Of the 6127 respondents, 3063 were females (50%), and 3064 were males (50%). Approximately 7.9% reported that they had ADL disability, and nearly one-fifth (18.0%) indicated that they had IADL disability. The statistical description of the demographic characteristics, health-related variables, and physical performances of our participants is reported in Table 1. The mean handgrip strength was 29.14 (28.90 − 29.38), 24.80 (23.83 − 25.78), and 25.27 (24.69 − 25.85) kg, and the mean gait speed was 0.62 (0.62 − 0.63), 0.49 (0.47 − 0.51), and 0.52 (0.51 − 0.53) m/s in all cohorts, the ADL disability group, and the IADL disability group, respectively.

**Table 1:** Demographic characteristics and health-related information of the sample.

		Overall (n = 6127)		ADL (n = 485)		IADL (n = 1104)	
Variables	Categories	n	% (95%CI)	n	% (95%CI)	n	% (95%CI)
**Age**	60–64 years	2339	38.2(37.0–39.4)	112	23.1(19.4–27.1)	286	25.9(23.3–28.6)
	65–69 years	1546	25.2(24.1–26.3)	111	22.9(19.2–26.9)	267	24.2(21.7–26.8)
	70–74 years	1096	17.9(17.0–18.9)	97	20.0(16.5–23.8)	204	18.5(16.3–20.9)
	75 and above	1146	18.7(17.7–19.7)	165	34.0(29.8–38.4)	347	31.4(28.7–34.2)
**Gender**	Male	3064	50.0(48.7–51.3)	219	45.2(40.7–49.8)	455	41.2(38.3–44.2)
	Female	3063	50.0(48.7–51.3)	266	54.8(50.3–59.3)	649	58.8(55.8–61.7)
**Hukou**	Non-agricultural	4839	79.0(78.0–80.0)	399	82.4(78.7–85.7)	947	85.8(83.6–87.8)
	Agricultural	1284	21.0(20.0–22.0)	85	17.6(14.3–23.3)	157	14.2(12.2–16.4)
**Education**	Illiterate	2288	37.3(36.1–38.5)	249	51.3(46.8–55.8)	616	55.8(52.8–58.8)
	Primary school	2769	45.2(44.0–46.5)	178	36.7(32.4–41.2)	385	34.9(32.1–37.8)
	Middle school	709	11.6(10.8–12.4)	40	8.2(5.9–11.0)	78	7.1(5.7–8.8)
	High school	248	4.0(3.5–4.5)	14	2.9(1.6–4.8)	18	1.6(1.0–2.5)
	College and above	108	1.8(1.5–2.2)	4	0.8(0.2–2.1)	7	0.6(0.2–1.3)
**Marital status**	Married/ Cohabitated	4773	77.9(76.8–78.9)	352	72.6(68.4–76.5)	792	71.7(68.9–74.3)
	Other	1354	22.1(21.1–23.2)	133	27.4(23.5–31.6)	312	28.3(25.7–31.1)
**Physical activity**	1 Never	329	5.4(4.9–6.0)	63	30.7(26.6–35.0)	115	26.7(24.1–29.4)
	2 Inadequate	61	1.0(0.8–1.3)	7	3.4(2.0–5.4)	18	4.2(3.1–5.6)
	3 Adequate	2158	35.2(34.0–36.4)	135	65.9(61.5–70.1)	297	69.1(66.3–71.8)
**Social activity**	Never	3139	51.2(49.9–52.5)	296	64.2(59.8–68.7)	656	63.1(60.2–66.0)
	Not regularly	685	11.2(10.4–12.0)	30	6.5(4.5–9.1)	113	10.9(9.1–12.9)
	Almost weekly	604	9.9(9.2–10.7)	37	8.0(5.8–10.8)	76	7.3(5.8–9.0)
	Almost daily	1524	24.9(23.8–26.0)	98	21.3(17.7–25.2)	194	18.7(16.4–21.1)
**Number of diseases**	0	1526	24.9(23.8–26.0)	41	8.5(6.2–11.4)	180	16.3(14.2–18.6)
	1	1803	29.4(28.3–30.6)	112	23.1(19.4–27.1)	263	23.8(21.3–26.4)
	≥2	2797	45.7(44.5–47.0)	332	68.5(64.2–72.6)	661	59.9(56.9–62.8)
**BMI**	Underweight	626	10.2(9.5–11.0)	46	10.7(8.1–13.8)	139	13.4(11.4–15.6)
	Normal	3277	53.5(52.2–54.8)	212	49.4(44.9–53.9)	556	53.5(50.5–56.5)
	Obese	2066	33.7(32.5–34.9)	171	39.9(35.6–44.4)	345	33.2(30.4–36.1)
**Handgrip strength** **(kg)**	Mean (95%CI)	29.14(28.90–29.38)		24.80(23.83–25.78)		25.27(24.69–25.85)	
**Speed (m/s)**	Mean (95%CI)	0.62(0.62–0.63)		0.49(0.47–0.51)		0.52(0.51–0.53)	

BMI, body mass index; IADL, instrumental activities of daily living; ADL, activities of daily living.

### Cut-off values of handgrip strength and gait speed according to the ROC curve

For recognizing ADL disability, the cut-off values of handgrip strength were 30.15 and 20.05 kg, and those for the gait speed were 0.49 and 0.47 m/s for men and women, respectively. For discriminating IADL disability, the cut-off values of handgrip strength were 31.65 and 23.55 kg, and those for the gait speed were 0.59 and 0.54 m/s for men and women, respectively (Table 2). The cut-off values of handgrip strength and gait speed for IADL disability were generally higher than those for ADL disability both in men and women.

**Table 2:** Validity of handgrip strength and gait speed for ADL and IADL disabilities according to the ROC analysis for men and women separately.

	Physical Performance Test	Gender	Cut-off	n (%) ^a^	AUC (95%CI)	Se (%)	Sp (%)
**ADL**	Handgrip strength, kg	Male	30.15	882(28.8)	0.67 (0.63–0.72)	57.8	72.8
		Female	20.05	952(31.1)	0.64 (0.59–0.68)	54.0	70.1
	Speed, m/s	Male	0.49	634(20.7)	0.70 (0.66–0.74)	53.5	79.2
		Female	0.47	825(26.9)	0.68 (0.64–0.72)	50.0	74.1
**IADL**	Handgrip strength, kg	Male	31.65	1041(34.0)	0.66 (0.63–0.69)	55.5	68.8
		Female	23.55	1455(47.5)	0.63 (0.60–0.66)	65.5	55.6
	Speed, m/s	Male	0.59	1163(38.0)	0.66 (0.63–0.69)	60.8	62.8
		Female	0.54	1168(38.1)	0.66 (0.63–0.68)	59.4	63.4

AUC, area under the curve; CI, confidence interval; Se, sensitivity; Sp, specificity; IADL, instrumental activities of daily living; ADL, activities of daily living.

^a.^ The number and proportion of males and females that are below the cut-off values are listed in Table 2.

**Fig 2:**
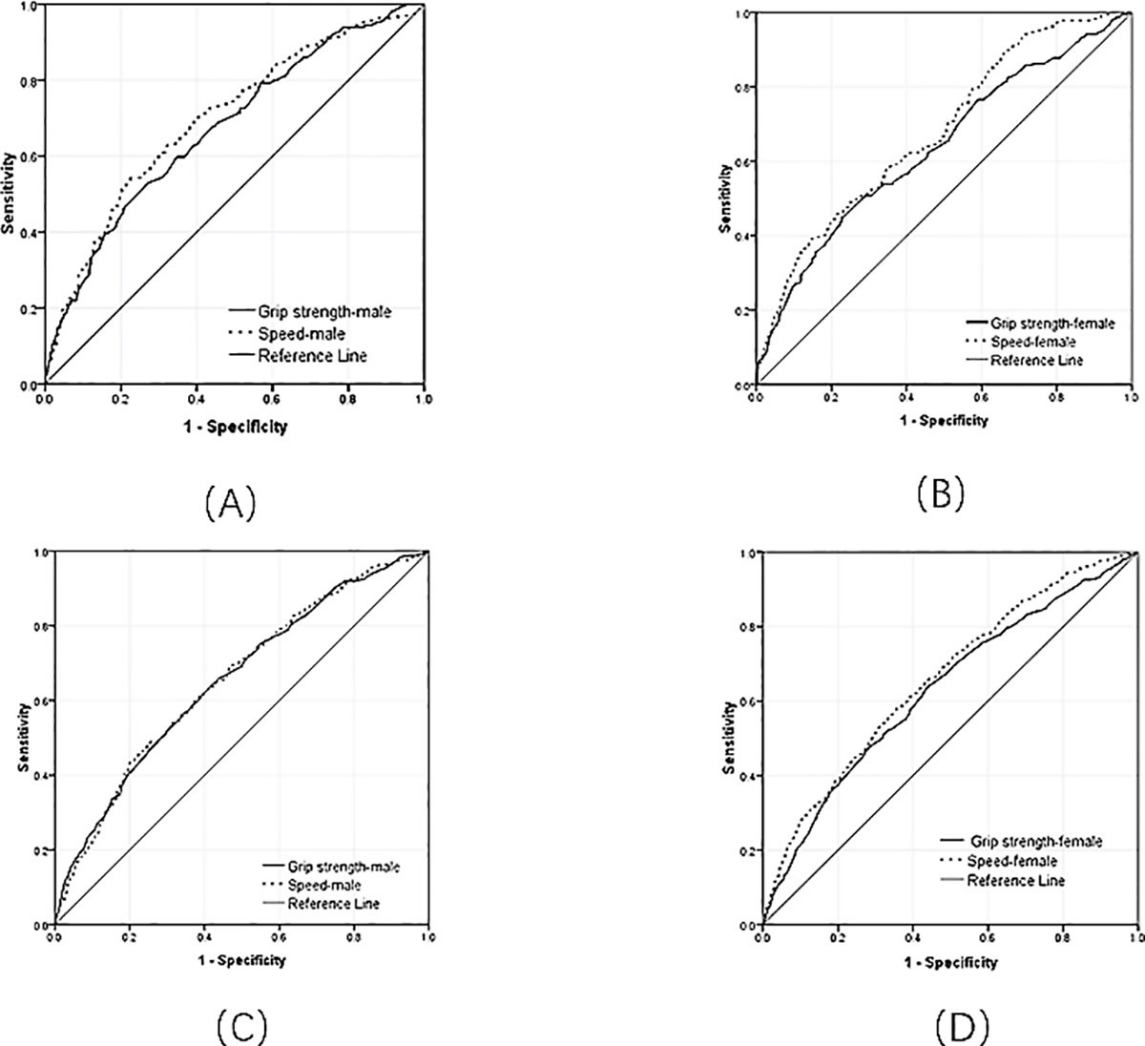
ROC curves for each variable for ADL and IADL disabilities. (A) ROC curves for each variable for ADL disability in males. (B) ROC curves for each variable for ADL disability in females. (C) ROC curves for each variable for IADL disability in males. (D) ROC curves for each variable for IADL disability in females.

The ROC curve shown in Fig 2 represents the discernment value of handgrip strength and gait speed for men and women separately. For recognizing ADL disability, the speed test could better distinguish than handgrip strength and showed a satisfactory discriminant validity (AUC ≥ 0.7) in men. For discriminating IADL disability, the AUC of handgrip strength and gait speed had roughly no difference both in men and women.

### Logistic regression analysis of the associations between physical performances with ADL and IADL disabilities

Logistic regression analysis of the relationships between physical performances with ADL and IADL disabilities is presented in Table 3. After adjusted by the covariates, handgrip strength and gait speed were significantly associated with the ADL disability (OR = 2.26, 95% CI = 1.53 − 3.36 for handgrip strength; OR = 1.91, 95% CI = 1.30 − 2.81 for gait speed) and IADL disability (OR = 1.66, 95% CI = 1.27 − 2.15 for handgrip strength; OR = 1.70, 95% CI = 1.32 − 2.19 for gait speed).

**Table 3:** Associations of physical performances with ADL and IADL disabilities in Chinese older adults (N = 6127).

variables		OR (95%CI) ^a^
**ADL**	Grip strength ^b^	2.26(1.53–3.36) ***
	Speed ^b^	1.91(1.30–2.81) ***
**IADL**	Grip strength ^b^	1.66(1.27–2.15) ***
	Speed ^b^	1.70(1.32–2.19) ***

CI, confidence interval; OR, odds ratio; IADL, instrumental activities of daily living; ADL, activities of daily living.

^a.^ Hukou, age, gender, marital status, education level, BMI, social and physical activity frequency, comorbidity condition, handgrip strength and gait speed were controlled in in Model.

^b.^ We defined handgrip strength and gait speed as binary variables based on the cut-off values we calculated in Table 2.

All analyses were conducted at a 5% significance level. Statistical thresholds:

*** p-value < 0.001.

### Validity of physical performance measures alone and in combination

The validity of physical performance measures alone and in combination is summarized in Table 4. For recognizing ADL disability, the sensitivity and specificity of handgrip strength and gait speed alone were 55.7% and 71.4%, and 50.9% and 76.1%, respectively, whereas those for identifying IADL disability were 61.4% and 62.5%, 60.0% and 62.8%, respectively. When combining the handgrip strength and gait speed together, the parallel test showed a good sensitivity with a poor specificity whereas the serial test showed a high specificity with a poor sensitivity.

**Table 4:** Validity of physical performance measures alone and in combination for recognizing functional disability.

index	ADL		IADL	
	Sensitivity (%)	Specificity (%)	Sensitivity (%)	Specificity (%)
Grip strength	55.7	71.4	61.4	62.5
Speed	50.9	76.1	60.0	62.8
Parallel test ^a^	76.0	56.5	81.0	41.4
Serial test ^b^	28.5	90.6	38.2	83.9

IADL, instrumental activities of daily living; ADL, activities of daily living.

^a.^ parallel test: a test that the result was positive if any of the screening tests were positive.

^b.^ serial test: a test in which the result was positive only if all screening tests were positive.

## Discussion

In this study, we developed the criterion-referenced values of handgrip strength and gait speed for functional disability. Additionally, we validated the significantly positive association between poor physical performances with ADL and IADL disabilities by logistic regression model. On these bases, we compared the validity of the combined tests of handgrip strength and gait speed. The parallel test showed a high sensitivity with a poor specificity, whereas the serial tests showed a perfect specificity with a poor sensitivity.

Similar investigations according to handgrip strength have also been found in other countries. Janne Sallinen et al. determined optimal handgrip strength cut-off points for increased likelihood for mobility limitation and studied whether these cut-points differ according to BMI in the Finnish population-based Health 2000 Survey [28]. McGrath RP et al. identified sex- and ethnic-specific handgrip strength weakness thresholds associated with functional limitations and determined the odds of functional limitations for each ethnicity by sex among older adults in Spain [29]. Our study, to some extent, parallels the above investigations, creating cut-off points of handgrip strength from the ROC curves and using them for a logistic model. The difference is that our research focuses not only on the discernment role of single physical performance indicator but also on the combined recognition function of these indicators. We collected the data from the general Chinese population, where the demographic and socioeconomic characteristics are different from these above countries.

We calculated the cut-off values of handgrip strength and gait speed for the discrimination of ADL and IADL disabilities in older adults according to the ROC curve. The cut-off values for IADL disability were generally higher than those for ADL disability. Our findings, to some extent, parallel the results that the limitations in performing IADL often preceded the start of ADL limitations, according to the hierarchical disablement model [7–8]. Functional disability could measure intrinsic capacity [9]. Therefore, interventions that are tailored for the specific stage of internal capability trajectory according to the cut-off values at different risks of functional disabilities, could be implemented. For older people with high and stable levels of capacity, the goal is to continue to maintain these levels for as long as possible. For older people with declining capacity, the emphasis of interventions will generally shift from prevention or cure to delay or even partially reverse the process of becoming care-dependent. For older people with a significant loss of capacity, a long-term care system is vital to enable older persons to have basic rights and human dignity [9].

Decreased handgrip strength and gait speed are associated with increased odds for ADL and IADL disabilities [13, 30, 31]. Similarly, our study also validated a positive association between poor physical performances and functional disability. Based on these, older people should try to stay as active as possible to maintain favorable strength and speed [32]. All domains of fitness, namely, aerobic exercise and progressive resistance training exercises, were important, and resistance training was particularly essential if capacity was declining. Progressive resistance training not only benefited on muscular strength and physical capacity [33] but also helped improve the daily functioning [8]. Even if the function decline in older age could limit mobility, providing assistive technologies to aid mobility was perhaps most easily illustrated. For older people with a significant loss of capacity, Gill et al. found that a home-based intervention program consisting of balance and muscle strengthening and conditioning exercises, with the proper use of assistive devices, was effective in improving the ability to perform functional limitations [34]. Thus, multicomponent physical activity interventions, including aerobic exercise and progressive resistance training exercises (with or without an assistive device), should run through the whole stages of intrinsic capacity.

The AUC ≥0.70 is the criterion of good discrimination [7]. In our study, only the AUC of the gait speed test for discriminating ADL disability in men was ≥ 0.7. Compared with handgrip strength, the gait speed test could better discriminate ADL disability both in men and women. Thus, the gait speed is more preferable than handgrip strength for identifying ADL disability. To the best of our knowledge, no studies have compared physical performance measures among general elderly population to identify the best discriminating measures for ADL disability. However, one study had compared the ability of three performance-based measures, namely, short physical performance battery, gait speed, and handgrip strength, to identify the functional disability among older women with breast cancer; as a result, the short physical performance battery and gait speed were the most robust identification indicators for functional disability [35]. Our results are fairly consistent with these findings in which the gait speed was the satisfactory measure for ADL disability. Meanwhile, for the IADL disability, the AUCs of handgrip strength and gait speed were all ≥ 0.6 and ≤ 0.7, respectively, indicating a relatively poor recognition ability for functional disability. This finding is consistent with the results from the study of Meng-Chih Lee [7], which observed that the AUCs of the handgrip strength in women and gait speed both in men and women for discriminating IADL disability were all ≤ 0.7.

In our study, the parallel test showed a high sensitivity with a poor specificity, whereas the serial test showed a good specificity with a poor sensitivity. Given that one of the main goals was to identify as many high-risk groups with disabilities as possible and then interrupt the disability process, the performance of the parallel test with high sensitivity was more preferable than that of the serial test. Nevertheless, compared with the sensitivity (50%−60%) of handgrip strength and gait speed alone to identify functional disabilities, the sensitivity in the parallel test (70% − 80%) did not clearly increase. In addition, the specificity of some data in the parallel test was unsatisfactory (specificity ≤ 50%). To some extent, the combined test increased time, labor, and money cost. Therefore, future studies about cost-utility analysis on the parallel tests are necessary.

To the best of our knowledge, this study is the first large-scale older population-based study to compare the discriminant ability of physical performance indices for functional disability in China. Furthermore, we provided the cut-off values of physical performance measures stratified according to the degree of disability. However, several limitations need to be mentioned in this study. First, our results were based on cross-sectional data, and fully valuing the utilities of physical performance measures might be difficult. Second, we used self-reported functional disability questionnaires with a broader domain to test the validity of the physical performance measures. Thus, direct comparisons between the physical performance indices and self-reported questionnaires might be challenging. However, both modes of measurement are considered measures of the functional disability domain, and both are advocated as outcomes of this domain [11].

Nevertheless, we do not suggest replacing the self-reported measure of functional disability in older adults with physical performance measures. Many other factors, including cognitive status, depression, as well as other personal and environmental factors, are associated with functional disability [36–37]. However, knowing the physical performance indices of older adults on these tests could help clinicians to determine the disability risk levels to perform appropriate interventions.

## Conclusion

We developed the cut-off points of handgrip strength and gait speed for the stratification of functional disability in older adults. Furthermore, after comparing the validity of their cut-off points, we found that the gait speed was superior to handgrip strength for identifying ADL disability. Based on the goals of interventions for high-risk group, the performance of parallel tests with high sensitivity was more preferable than serial tests. Nonetheless, further cost-utility analysis is necessary. Overall, the physical performance measures could provide additional information about the physical function of older adults to help clinicians identify the high-risk groups with functional disability and guide the targeted interventions.

## Supporting information

S1 File Relevant dataset underlying the findings described in manuscript. (RAR)

S2 File The codebook of the dataset. (PDF)
