# Supplementary material for: Role of physical performance measures for identifying functional disability among Chinese older adults: Data from the China Health and Retirement Longitudinal Study
Source: PLoS One. 2019 Apr 18;14(4):e0215693. doi: 10.1371/journal.pone.0215693 (PMC6472820; doi:10.1371/journal.pone.0215693)
Supplement: S2 File — (PDF) [file pone.0215693.s002.pdf]

China Health and Retirement Longitudinal Study

National Baseline

CodeBook

Mar 2013

China Center for Ecomomic Research
Peking University

Contents

1 Household Roster 1

2 Demographic Background 133

3 Family Information 173

4 Family Transfer 451

5 Health Status and Functioning 603

6 Health Care and Insurance 730

7 Work, Retirement and Pension 814

8 Household Income 950

9 Individual Income 1109

10 Housing Characteristics 1135

11 Interviewer Observation 1143

12 Biomarkers 1146

13 Community 1191

14 Weight 1332

15 PSU 1341

1 Household Roster

- **householdID : Household ID**

A String Variable	
OBS:	10,029

- **ID : Individual ID**

A String Variable	
OBS:	10,029

- **communityID : Community ID**

A String Variable	
OBS:	10,029

- **a001 : Area Type Taken Down by IWER**

	No	%
1 Rural Village	7,718	75.47
2 Urban Community	2,509	24.53
Total	10,227	100.00

- **a002_1_ : Gender of This Household Member**

	No	%
1 Male	577	72.95
2 Female	214	27.05
Total	791	100.00

- **a002_2_ : Gender of This Household Member**

	No	%
1 Male	1,014	55.29
2 Female	820	44.71
Total	1,834	100.00

- **a002_3_ : Gender of This Household Member**

	No	%

1 Male	3,593	60.75
2 Female	2,321	39.25
Total	5,914	100.00

- **a002_4_ : Gender of This Household Member**

	No	%
1 Male	1,772	41.41
2 Female	2,507	58.59
Total	4,279	100.00

- **a002_5_ : Gender of This Household Member**

	No	%
1 Male	1,380	49.89
2 Female	1,386	50.11
Total	2,766	100.00

- **a002_6_ : Gender of This Household Member**

	No	%
1 Male	687	50.78
2 Female	666	49.22
Total	1,353	100.00

- **a002_7_ : Gender of This Household Member**

	No	%
1 Male	263	50.29
2 Female	260	49.71
Total	523	100.00

- **a002_8_ : Gender of This Household Member**

	No	%
1 Male	126	51.64
2 Female	118	48.36
Total	244	100.00

- **a002_9_ : Gender of This Household Member**

	No	%
1 Male	63	45.99
2 Female	74	54.01
Total	137	100.00

- **a002_10_ : Gender of This Household Member**

	No	%
1 Male	37	55.22
2 Female	30	44.78
Total	67	100.00

- **a002_11_ : Gender of This Household Member**

	No	%
1 Male	19	55.88
2 Female	15	44.12
Total	34	100.00

- **a002_12_ : Gender of This Household Member**

	No	%
1 Male	6	31.58
2 Female	13	68.42
Total	19	100.00

- **a002_13_ : Gender of This Household Member**

	No	%
1 Male	9	90.00
2 Female	1	10.00
Total	10	100.00

- **a002_14_ : Gender of This Household Member**

	No	%
1 Male	5	83.33
2 Female	1	16.67
Total	6	100.00

- **a002_15_ : Gender of This Household Member**

	No	%
2 Female	2	100.00
Total	2	100.00

- **a002_16_ : Gender of This Household Member**

	No	%
2 Female	1	100.00
Total	1	100.00

- **a003_1_1_ : Year**

Mean	Min	Max	OBS
1,962.6	1,900.0	2,010.0	775

- **a003_1_2_ : Year**

Mean	Min	Max	OBS
1,976.8	1,900.0	2,011.0	1,790

- **a003_1_3_ : Year**

Mean	Min	Max	OBS
1,984.8	1,900.0	2,011.0	5,826

- **a003_1_4_ : Year**

Mean	Min	Max	OBS
1,988.4	1,910.0	2,011.0	4,184

- **a003_1_5_ : Year**

Mean	Min	Max	OBS
1,994.9	1,902.0	2,011.0	2,725

- **a003_1_6_ : Year**

Mean	Min	Max	OBS
1,994.8	1,903.0	2,011.0	1,321

- **a003_1_7_ : Year**

Mean	Min	Max	OBS
1,993.3	1,919.0	2,011.0	508

- **a003_1_8_ : Year**

Mean	Min	Max	OBS
1,994.8	1,921.0	2,011.0	241

- **a003_1_9_ : Year**

Mean	Min	Max	OBS
1,994.1	1,938.0	2,011.0	133

- **a003_1_10_ : Year**

Mean	Min	Max	OBS
1,997.1	1,967.0	2,011.0	66

- **a003_1_11_ : Year**

Mean	Min	Max	OBS
1,998.7	1,950.0	2,011.0	31

- **a003_1_12_ : Year**

Mean	Min	Max	OBS
1,997.3	1,974.0	2,011.0	18

- **a003_1_13_ : Year**

Mean	Min	Max	OBS
1,999.0	1,972.0	2,010.0	9

- **a003_1_14_ : Year**

Mean	Min	Max	OBS
1,996.0	1,977.0	2,008.0	5

- **a003_1_15_ : Year**

Mean	Min	Max	OBS
1,970.0	1,952.0	1,988.0	2

- **a003_1_16_ : Year**

Mean	Min	Max	OBS
1,980.0	1,980.0	1,980.0	1

- **a003_2_1_ : Month**

	No	%
0	46	5.94
1	64	8.27
2	59	7.62
3	62	8.01
4	52	6.72
5	48	6.20
6	56	7.24
7	54	6.98
8	73	9.43
9	60	7.75
10	73	9.43
11	53	6.85
12	74	9.56
Total	774	100.00

- **a003_2_2_ : Month**

	No	%
0	143	8.00
1	163	9.12
2	136	7.61
3	145	8.11
4	125	6.99
5	144	8.05
6	131	7.33
7	122	6.82

8	146	8.17
9	133	7.44
10	153	8.56
11	115	6.43
12	132	7.38
Total	1,788	100.00

- **a003_2_3_ : Month**

	No	%
0	332	5.70
1	434	7.45
2	489	8.39
3	421	7.23
4	400	6.87
5	416	7.14
6	431	7.40
7	441	7.57
8	487	8.36
9	515	8.84
10	584	10.02
11	436	7.48
12	440	7.55
Total	5,826	100.00

- **a003_2_4_ : Month**

	No	%
0	365	8.74
1	311	7.45
2	322	7.71
3	277	6.63
4	264	6.32
5	278	6.66
6	323	7.73
7	341	8.16
8	343	8.21
9	333	7.97
10	404	9.67
11	310	7.42
12	306	7.33
Total	4,177	100.00

- **a003_2_5_ : Month**

	No	%
0	196	7.20

1	213	7.83
2	193	7.09
3	200	7.35
4	198	7.28
5	192	7.06
6	195	7.17
7	187	6.87
8	212	7.79
9	261	9.59
10	246	9.04
11	208	7.64
12	220	8.09
Total	2,721	100.00

- **a003_2_6_ : Month**

	No	%
0	124	9.41
1	120	9.10
2	93	7.06
3	99	7.51
4	87	6.60
5	82	6.22
6	104	7.89
7	106	8.04
8	117	8.88
9	101	7.66
10	97	7.36
11	85	6.45
12	103	7.81
Total	1,318	100.00

- **a003_2_7_ : Month**

	No	%
0	44	8.68
1	28	5.52
2	48	9.47
3	40	7.89
4	32	6.31
5	37	7.30
6	42	8.28
7	39	7.69
8	53	10.45
9	35	6.90
10	44	8.68
11	33	6.51
12	32	6.31
Total	507	100.00

- **a003_2_8_ : Month**

	No	%
0	17	7.05
1	15	6.22
2	27	11.20
3	16	6.64
4	13	5.39
5	24	9.96
6	19	7.88
7	18	7.47
8	20	8.30
9	18	7.47
10	20	8.30
11	18	7.47
12	16	6.64
Total	241	100.00

- **a003_2_9_ : Month**

	No	%
0	18	13.64
1	10	7.58
2	7	5.30
3	6	4.55
4	11	8.33
5	8	6.06
6	9	6.82
7	14	10.61
8	9	6.82
9	11	8.33
10	10	7.58
11	9	6.82
12	10	7.58
Total	132	100.00

- **a003_2_10_ : Month**

	No	%
0	9	13.64
1	6	9.09
2	3	4.55
3	5	7.58
4	4	6.06
5	4	6.06
6	4	6.06

7	9	13.64
8	4	6.06
9	6	9.09
10	5	7.58
11	3	4.55
12	4	6.06
Total	66	100.00

- **a003_2_11_ : Month**

	No	%
0	1	3.23
1	2	6.45
2	4	12.90
5	4	12.90
6	4	12.90
7	3	9.68
8	2	6.45
9	5	16.13
10	2	6.45
11	2	6.45
12	2	6.45
Total	31	100.00

- **a003_2_12_ : Month**

	No	%
1	1	5.56
2	1	5.56
4	3	16.67
6	2	11.11
8	4	22.22
9	1	5.56
10	2	11.11
11	2	11.11
12	2	11.11
Total	18	100.00

- **a003_2_13_ : Month**

	No	%
1	2	22.22
3	1	11.11
4	2	22.22
6	2	22.22
10	2	22.22
Total	9	100.00

- **a003_2_14_ : Month**

	No	%
0	1	20.00
3	1	20.00
5	1	20.00
7	1	20.00
9	1	20.00
Total	5	100.00

- **a003_2_15_ : Month**

	No	%
3	1	50.00
9	1	50.00
Total	2	100.00

- **a003_2_16_ : Month**

	No	%
0	1	100.00
Total	1	100.00

- **a004_1_ : Marital Status of This Household Member**

	No	%
1 Married with spouse present	488	63.05
2 Married but not living with spouse temporarily for reasons such as work	53	6.85
3 Separated	4	0.52
4 Divorced	15	1.94
5 Widowed	151	19.51
6 Never married	63	8.14
Total	774	100.00

- **a004_2_ : Marital Status of This Household Member**

	No	%
1 Married with spouse present	962	60.69
2 Married but not living with spouse temporarily for reasons such as work	125	7.89
3 Separated	9	0.57
4 Divorced	31	1.96
5 Widowed	102	6.44

6 Never married	356	22.46
Total	1,585	100.00

- **a004_3_ : Marital Status of This Household Member**

	No	%
1 Married with spouse present	2,190	46.21
2 Married but not living with spouse temporarily for reasons such as work	244	5.15
3 Separated	25	0.53
4 Divorced	91	1.92
5 Widowed	166	3.50
6 Never married	2,023	42.69
Total	4,739	100.00

- **a004_4_ : Marital Status of This Household Member**

	No	%
1 Married with spouse present	1,720	55.97
2 Married but not living with spouse temporarily for reasons such as work	150	4.88
3 Separated	7	0.23
4 Divorced	18	0.59
5 Widowed	58	1.89
6 Never married	1,120	36.45
Total	3,073	100.00

- **a004_5_ : Marital Status of This Household Member**

	No	%
1 Married with spouse present	527	40.95
2 Married but not living with spouse temporarily for reasons such as work	114	8.86
3 Separated	4	0.31
4 Divorced	12	0.93
5 Widowed	35	2.72
6 Never married	595	46.23
Total	1,287	100.00

- **a004_6_ : Marital Status of This Household Member**

	No	%
1 Married with spouse present	278	44.27
2 Married but not living with spouse temporarily for reasons such as work	64	10.19
3 Separated	3	0.48
4 Divorced	10	1.59
5 Widowed	18	2.87
6 Never married	255	40.61

Total	628	100.00

- **a004_7_ : Marital Status of This Household Member**

	No	%
1 Married with spouse present	130	48.87
2 Married but not living with spouse temporarily for reasons such as work	19	7.14
3 Separated	1	0.38
4 Divorced	4	1.50
5 Widowed	9	3.38
6 Never married	103	38.72
Total	266	100.00

- **a004_8_ : Marital Status of This Household Member**

	No	%
1 Married with spouse present	72	60.50
2 Married but not living with spouse temporarily for reasons such as work	11	9.24
4 Divorced	1	0.84
5 Widowed	3	2.52
6 Never married	32	26.89
Total	119	100.00

- **a004_9_ : Marital Status of This Household Member**

	No	%
1 Married with spouse present	40	55.56
2 Married but not living with spouse temporarily for reasons such as work	7	9.72
6 Never married	25	34.72
Total	72	100.00

- **a004_10_ : Marital Status of This Household Member**

	No	%
1 Married with spouse present	13	46.43
2 Married but not living with spouse temporarily for reasons such as work	4	14.29
6 Never married	11	39.29
Total	28	100.00

- **a004_11_ : Marital Status of This Household Member**

	No	%
1 Married with spouse present	6	42.86

2 Married but not living with spouse temporarily for reasons such as work	1	7.14
5 Widowed	1	7.14
6 Never married	6	42.86
Total	14	100.00

- **a004_12_ : Marital Status of This Household Member**

	No	%
1 Married with spouse present	1	11.11
2 Married but not living with spouse temporarily for reasons such as work	2	22.22
6 Never married	6	66.67
Total	9	100.00

- **a004_13_ : Marital Status of This Household Member**

	No	%
1 Married with spouse present	1	33.33
2 Married but not living with spouse temporarily for reasons such as work	2	66.67
Total	3	100.00

- **a004_14_ : Marital Status of This Household Member**

	No	%
2 Married but not living with spouse temporarily for reasons such as work	2	66.67
6 Never married	1	33.33
Total	3	100.00

- **a004_15_ : Marital Status of This Household Member**

	No	%
5 Widowed	1	50.00
6 Never married	1	50.00
Total	2	100.00

- **a004_16_ : Marital Status of This Household Member**

	No	%
1 Married with spouse present	1	100.00
Total	1	100.00

- **a005_1_ : Whether This Household Member Unmarried but Living with**

a Partner

	No	%
1 Yes	6	3.35
2 No	173	96.65
Total	179	100.00

- **a005_2_ : Whether This Household Member Unmarried but Living with a Partner**

	No	%
1 Yes	5	3.60
2 No	134	96.40
Total	139	100.00

- **a005_3_ : Whether This Household Member Unmarried but Living with a Partner**

	No	%
1 Yes	4	1.79
2 No	219	98.21
Total	223	100.00

- **a005_4_ : Whether This Household Member Unmarried but Living with a Partner**

	No	%
1 Yes	4	4.94
2 No	77	95.06
Total	81	100.00

- **a005_5_ : Whether This Household Member Unmarried but Living with a Partner**

	No	%
1 Yes	1	1.85
2 No	53	98.15
Total	54	100.00

- **a005_6_ : Whether This Household Member Unmarried but Living with a Partner**

	No	%
2 No	42	100.00
Total	42	100.00

- **a005_7_ : Whether This Household Member Unmarried but Living with a Partner**

	No	%
2 No	19	100.00
Total	19	100.00

- **a005_8_ : Whether This Household Member Unmarried but Living with a Partner**

	No	%
2 No	4	100.00
Total	4	100.00

- **a005_9_ : Whether This Household Member Unmarried but Living with a Partner**

	No	%
2 No	4	100.00
Total	4	100.00

- **a005_10_ : Whether This Household Member Unmarried but Living with a Partner**

	No	%
2 No	2	100.00
Total	2	100.00

- **a005_11_ : Whether This Household Member Unmarried but Living with a Partner**

	No	%
2 No	2	100.00
Total	2	100.00

- **a005_12_ : Whether This Household Member Unmarried but Living with a Partner**

	No	%
2 No	1	100.00
Total	1	100.00

- **a005_15_ : Whether This Household Member Unmarried but Living with a Partner**

	No	%
2 No	1	100.00
Total	1	100.00

- **a006_1_ : Relationship to FamilyR**

	No	%
1 Mother	81	10.24
2 Father	35	4.42
3 Mother-in-law	28	3.54
4 Father-in-law	29	3.67
5 Sibling	25	3.16
6 Brother-in-law, sister-in-law	1	0.13
7 Child	484	61.19
8 Spouse of child	70	8.85
9 Grandchild	30	3.79
10 Other relative specify	8	1.01
Total	791	100.00

- **a006_2_ : Relationship to FamilyR**

	No	%
1 Mother	56	3.06
2 Father	16	0.87
3 Mother-in-law	27	1.47
4 Father-in-law	8	0.44
5 Sibling	15	0.82
6 Brother-in-law, sister-in-law	6	0.33
7 Child	976	53.28
8 Spouse of child	436	23.80
9 Grandchild	264	14.41
10 Other relative specify	28	1.53
Total	1,832	100.00

- **a006_3_ : Relationship to FamilyR**

	No	%
1 Mother	86	1.45
2 Father	39	0.66
3 Mother-in-law	49	0.83
4 Father-in-law	20	0.34
5 Sibling	18	0.30
6 Brother-in-law, sister-in-law	6	0.10
7 Child	3,802	64.29
8 Spouse of child	675	11.41
9 Grandchild	1,127	19.06
10 Other relative specify	92	1.56
Total	5,914	100.00

- **a006_4_ : Relationship to FamilyR**

	No	%
1 Mother	40	0.93
2 Father	11	0.26
3 Mother-in-law	26	0.61
4 Father-in-law	4	0.09
5 Sibling	12	0.28
6 Brother-in-law, sister-in-law	4	0.09
7 Child	1,497	34.98
8 Spouse of child	1,334	31.18
9 Grandchild	1,260	29.45
10 Other relative specify	91	2.13
Total	4,279	100.00

- **a006_5_ : Relationship to FamilyR**

	No	%
1 Mother	23	0.83
2 Father	4	0.14
3 Mother-in-law	8	0.29
4 Father-in-law	2	0.07
5 Sibling	4	0.14
6 Brother-in-law, sister-in-law	4	0.14
7 Child	683	24.73
8 Spouse of child	316	11.44
9 Grandchild	1,643	59.49
10 Other relative specify	75	2.72
Total	2,762	100.00

- **a006_6_ : Relationship to FamilyR**

	No	%
1 Mother	9	0.67
2 Father	5	0.37
3 Mother-in-law	5	0.37
4 Father-in-law	3	0.22
5 Sibling	8	0.59
6 Brother-in-law, sister-in-law	1	0.07
7 Child	300	22.21
8 Spouse of child	187	13.84
9 Grandchild	769	56.92
10 Other relative specify	64	4.74
Total	1,351	100.00

- **a006_7_ : Relationship to FamilyR**

	No	%
1 Mother	7	1.34
2 Father	4	0.77
3 Mother-in-law	1	0.19
5 Sibling	3	0.57
7 Child	126	24.14
8 Spouse of child	79	15.13
9 Grandchild	265	50.77
10 Other relative specify	37	7.09
Total	522	100.00

- **a006_8_ : Relationship to FamilyR**

	No	%
3 Mother-in-law	1	0.41
4 Father-in-law	1	0.41
5 Sibling	3	1.23
6 Brother-in-law, sister-in-law	1	0.41
7 Child	44	18.03
8 Spouse of child	42	17.21
9 Grandchild	134	54.92
10 Other relative specify	18	7.38
Total	244	100.00

- **a006_9_ : Relationship to FamilyR**

	No	%
2 Father	1	0.74
5 Sibling	4	2.94
7 Child	25	18.38

8 Spouse of child	28	20.59
9 Grandchild	69	50.74
10 Other relative specify	9	6.62
Total	136	100.00

- **a006_10_ : Relationship to FamilyR**

	No	%
5 Sibling	2	2.94
7 Child	8	11.76
8 Spouse of child	12	17.65
9 Grandchild	42	61.76
10 Other relative specify	4	5.88
Total	68	100.00

- **a006_11_ : Relationship to FamilyR**

	No	%
3 Mother-in-law	1	2.94
7 Child	6	17.65
8 Spouse of child	2	5.88
9 Grandchild	23	67.65
10 Other relative specify	2	5.88
Total	34	100.00

- **a006_12_ : Relationship to FamilyR**

	No	%
7 Child	7	36.84
9 Grandchild	12	63.16
Total	19	100.00

- **a006_13_ : Relationship to FamilyR**

	No	%
7 Child	1	11.11
9 Grandchild	8	88.89
Total	9	100.00

- **a006_14_ : Relationship to FamilyR**

	No	%
7 Child	2	40.00

9 Grandchild	2	40.00
10 Other relative specify	1	20.00
Total	5	100.00

- **a006_15_ : Relationship to FamilyR**

	No	%
1 Mother	1	50.00
7 Child	1	50.00
Total	2	100.00

- **a006_16_ : Relationship to FamilyR**

	No	%
8 Spouse of child	1	100.00
Total	1	100.00

- **a007_1_ : Relationship to FamilyR**

	No	%
1 The biological child of you and your current spouse	172	96.09
2 The biological child of you, but not of your current spouse	3	1.68
3 The biological child of your current spouse, but not of you	2	1.12
4 The adopted or foster child of you or your spouse	2	1.12
Total	179	100.00

- **a007_2_ : Relationship to FamilyR**

	No	%
1 The biological child of you and your current spouse	246	96.09
2 The biological child of you, but not of your current spouse	9	3.52
4 The adopted or foster child of you or your spouse	1	0.39
Total	256	100.00

- **a007_3_ : Relationship to FamilyR**

	No	%
1 The biological child of you and your current spouse	3,519	97.29
2 The biological child of you, but not of your current spouse	33	0.91
3 The biological child of your current spouse, but not of you	22	0.61
4 The adopted or foster child of you or your spouse	43	1.19
Total	3,617	100.00

- **a007_4_ : Relationship to FamilyR**

	No	%
1 The biological child of you and your current spouse	1,367	97.16
2 The biological child of you, but not of your current spouse	16	1.14
3 The biological child of your current spouse, but not of you	13	0.92
4 The adopted or foster child of you or your spouse	11	0.78
Total	1,407	100.00

- **a007_5_ : Relationship to FamilyR**

	No	%
1 The biological child of you and your current spouse	614	97.15
2 The biological child of you, but not of your current spouse	7	1.11
3 The biological child of your current spouse, but not of you	7	1.11
4 The adopted or foster child of you or your spouse	4	0.63
Total	632	100.00

- **a007_6_ : Relationship to FamilyR**

	No	%
1 The biological child of you and your current spouse	273	98.20
2 The biological child of you, but not of your current spouse	3	1.08
3 The biological child of your current spouse, but not of you	1	0.36
4 The adopted or foster child of you or your spouse	1	0.36
Total	278	100.00

- **a007_7_ : Relationship to FamilyR**

	No	%
1 The biological child of you and your current spouse	113	97.41
4 The adopted or foster child of you or your spouse	3	2.59
Total	116	100.00

- **a007_8_ : Relationship to FamilyR**

	No	%
1 The biological child of you and your current spouse	40	100.00
Total	40	100.00

- **a007_9_ : Relationship to FamilyR**

	No	%

1 The biological child of you and your current spouse	21	100.00
Total	21	100.00

- **a007_10_ : Relationship to FamilyR**

	No	%
1 The biological child of you and your current spouse	8	100.00
Total	8	100.00

- **a007_11_ : Relationship to FamilyR**

	No	%
1 The biological child of you and your current spouse	3	75.00
4 The adopted or foster child of you or your spouse	1	25.00
Total	4	100.00

- **a007_12_ : Relationship to FamilyR**

	No	%
1 The biological child of you and your current spouse	5	83.33
4 The adopted or foster child of you or your spouse	1	16.67
Total	6	100.00

- **a007_13_ : Relationship to FamilyR**

	No	%
1 The biological child of you and your current spouse	1	100.00
Total	1	100.00

- **a007_14_ : Relationship to FamilyR**

	No	%
1 The biological child of you and your current spouse	2	100.00
Total	2	100.00

- **a007_15_ : Relationship to FamilyR**

	No	%
1 The biological child of you and your current spouse	1	100.00
Total	1	100.00

- **a008_1_ : Relationship to FamilyR**

	No	%
1 Your biological child.	308	99.35
2 Your adopted or foster child or step child.	2	0.65
Total	310	100.00

- **a008_2_ : Relationship to FamilyR**

	No	%
1 Your biological child.	712	98.48
2 Your adopted or foster child or step child.	11	1.52
Total	723	100.00

- **a008_3_ : Relationship to FamilyR**

	No	%
1 Your biological child.	200	98.52
2 Your adopted or foster child or step child.	3	1.48
Total	203	100.00

- **a008_4_ : Relationship to FamilyR**

	No	%
1 Your biological child.	93	95.88
2 Your adopted or foster child or step child.	4	4.12
Total	97	100.00

- **a008_5_ : Relationship to FamilyR**

	No	%
1 Your biological child.	54	98.18
2 Your adopted or foster child or step child.	1	1.82
Total	55	100.00

- **a008_6_ : Relationship to FamilyR**

	No	%
1 Your biological child.	25	100.00
Total	25	100.00

- **a008_7_ : Relationship to FamilyR**

	No	%
1 Your biological child.	10	100.00
Total	10	100.00

- **a008_8_ : Relationship to FamilyR**

	No	%
1 Your biological child.	5	100.00
Total	5	100.00

- **a008_9_ : Relationship to FamilyR**

	No	%
1 Your biological child.	5	100.00
Total	5	100.00

- **a008_11_ : Relationship to FamilyR**

	No	%
1 Your biological child.	1	50.00
2 Your adopted or foster child or step child.	1	50.00
Total	2	100.00

- **a008_12_ : Relationship to FamilyR**

	No	%
2 Your adopted or foster child or step child.	1	100.00
Total	1	100.00

- **a009_1_ : Current Hukou Status of This Household Member**

	No	%
1 Agriculture Hukou	638	80.76
2 Non-Agriculture Hukou	148	18.73
3 Unified Residency Hukou	2	0.25
4 Do not have Hukou	2	0.25
Total	790	100.00

- **a009_2_ : Current Hukou Status of This Household Member**

	No	%
1 Agriculture Hukou	1,441	78.70
2 Non-Agriculture Hukou	371	20.26
3 Unified Residency Hukou	7	0.38
4 Do not have Hukou	12	0.66
Total	1,831	100.00

- **a009_3_ : Current Hukou Status of This Household Member**

	No	%
1 Agriculture Hukou	4,477	75.86
2 Non-Agriculture Hukou	1,352	22.91
3 Unified Residency Hukou	38	0.64
4 Do not have Hukou	35	0.59
Total	5,902	100.00

- **a009_4_ : Current Hukou Status of This Household Member**

	No	%
1 Agriculture Hukou	3,451	80.84
2 Non-Agriculture Hukou	767	17.97
3 Unified Residency Hukou	16	0.37
4 Do not have Hukou	35	0.82
Total	4,269	100.00

- **a009_5_ : Current Hukou Status of This Household Member**

	No	%
1 Agriculture Hukou	2,289	82.90
2 Non-Agriculture Hukou	425	15.39
3 Unified Residency Hukou	10	0.36
4 Do not have Hukou	37	1.34
Total	2,761	100.00

- **a009_6_ : Current Hukou Status of This Household Member**

	No	%
1 Agriculture Hukou	1,168	86.58
2 Non-Agriculture Hukou	150	11.12
3 Unified Residency Hukou	7	0.52
4 Do not have Hukou	24	1.78
Total	1,349	100.00

- **a009_7_ : Current Hukou Status of This Household Member**

	No	%
1 Agriculture Hukou	454	87.14
2 Non-Agriculture Hukou	47	9.02
3 Unified Residency Hukou	4	0.77
4 Do not have Hukou	16	3.07
Total	521	100.00

- **a009_8_ : Current Hukou Status of This Household Member**

	No	%
1 Agriculture Hukou	214	88.07
2 Non-Agriculture Hukou	22	9.05
3 Unified Residency Hukou	2	0.82
4 Do not have Hukou	5	2.06
Total	243	100.00

- **a009_9_ : Current Hukou Status of This Household Member**

	No	%
1 Agriculture Hukou	117	86.03
2 Non-Agriculture Hukou	14	10.29
3 Unified Residency Hukou	1	0.74
4 Do not have Hukou	4	2.94
Total	136	100.00

- **a009_10_ : Current Hukou Status of This Household Member**

	No	%
1 Agriculture Hukou	60	89.55
2 Non-Agriculture Hukou	6	8.96
4 Do not have Hukou	1	1.49
Total	67	100.00

- **a009_11_ : Current Hukou Status of This Household Member**

	No	%
1 Agriculture Hukou	29	87.88
2 Non-Agriculture Hukou	1	3.03
4 Do not have Hukou	3	9.09
Total	33	100.00

- **a009_12_ : Current Hukou Status of This Household Member**

	No	%
1 Agriculture Hukou	18	94.74
2 Non-Agriculture Hukou	1	5.26
Total	19	100.00

- **a009_13_ : Current Hukou Status of This Household Member**

	No	%
1 Agriculture Hukou	8	88.89
2 Non-Agriculture Hukou	1	11.11
Total	9	100.00

- **a009_14_ : Current Hukou Status of This Household Member**

	No	%
1 Agriculture Hukou	5	100.00
Total	5	100.00

- **a009_15_ : Current Hukou Status of This Household Member**

	No	%
1 Agriculture Hukou	2	100.00
Total	2	100.00

- **a009_16_ : Current Hukou Status of This Household Member**

	No	%
1 Agriculture Hukou	1	100.00
Total	1	100.00

- **a010_1_ : Hukou Status before Unified**

	No	%
1 Agriculture Hukou	1	25.00
2 Non-Agriculture Hukou	2	50.00
3 Do not have Hukou	1	25.00
Total	4	100.00

- **a010_2_ : Hukou Status before Unified**

	No	%
1 Agriculture Hukou	5	55.56
2 Non-Agriculture Hukou	3	33.33
3 Do not have Hukou	1	11.11
Total	9	100.00

- **a010_3_ : Hukou Status before Unified**

	No	%
1 Agriculture Hukou	25	54.35
2 Non-Agriculture Hukou	17	36.96
3 Do not have Hukou	4	8.70
Total	46	100.00

- **a010_4_ : Hukou Status before Unified**

	No	%
1 Agriculture Hukou	8	47.06
2 Non-Agriculture Hukou	6	35.29
3 Do not have Hukou	3	17.65
Total	17	100.00

- **a010_5_ : Hukou Status before Unified**

	No	%
1 Agriculture Hukou	1	10.00
2 Non-Agriculture Hukou	6	60.00
3 Do not have Hukou	3	30.00
Total	10	100.00

- **a010_6_ : Hukou Status before Unified**

	No	%
1 Agriculture Hukou	1	12.50
2 Non-Agriculture Hukou	3	37.50
3 Do not have Hukou	4	50.00
Total	8	100.00

- **a010_7_ : Hukou Status before Unified**

	No	%

	No	%
1 Agriculture Hukou	3	75.00
2 Non-Agriculture Hukou	1	25.00
Total	4	100.00

- **a010_8_ : Hukou Status before Unified**

	No	%
2 Non-Agriculture Hukou	1	50.00
3 Do not have Hukou	1	50.00
Total	2	100.00

- **a010_9_ : Hukou Status before Unified**

	No	%
2 Non-Agriculture Hukou	1	100.00
Total	1	100.00

- **a011_1_ : Year**

No Observations

- **a011_2_ : Year**

Mean	Min	Max	OBS
2,005.7	2,000.0	2,010.0	3

- **a011_3_ : Year**

Mean	Min	Max	OBS
2,005.7	2,000.0	2,011.0	28

- **a011_4_ : Year**

Mean	Min	Max	OBS
2,004.0	2,000.0	2,010.0	12

- **a011_5_ : Year**

Mean	Min	Max	OBS
2,004.4	2,000.0	2,010.0	7

- **a011_6_ : Year**

Mean	Min	Max	OBS
2,004.4	2,000.0	2,009.0	5

- **a011_7_ : Year**

Mean	Min	Max	OBS
2,002.7	2,000.0	2,008.0	3

- **a011_8_ : Year**

Mean	Min	Max	OBS
2,007.0	2,007.0	2,007.0	1

- **a011_9_ : Year**

No Observations

- **a012_1_ : Location of Current Hukou**

	No	%
1 This Household	595	75.60
2 This Village/Neighborhood	137	17.41
3 Another Village/Neighborhood in this County/City	42	5.34
4 Another County/City in this Province	6	0.76
5 Another Province	7	0.89
Total	787	100.00

- **a012_1_1_ : City**

	No	%
04	1	16.67
24	1	16.67
27	1	16.67
50	1	16.67

77	1	16.67
82	1	16.67
Total	6	100.00

- **a012_1_2_ : City**

	No	%
04	2	6.25
11	3	9.38
27	2	6.25
40	13	40.63
46	2	6.25
53	3	9.38
74	1	3.13
77	2	6.25
82	2	6.25
86	1	3.13
96	1	3.13
Total	32	100.00

- **a012_1_3_ : City**

	No	%
01	5	3.40
04	11	7.48
07	1	0.68
11	8	5.44
16	2	1.36
17	2	1.36
24	8	5.44
27	3	2.04
40	50	34.01
42	2	1.36
46	13	8.84
49	4	2.72
50	1	0.68
52	1	0.68
53	7	4.76
55	6	4.08
60	1	0.68
63	4	2.72
74	3	2.04
82	7	4.76
84	1	0.68
86	5	3.40
96	2	1.36
Total	147	100.00

- **a012_1_4_ : City**

	No	%
01	6	5.36
02	2	1.79
04	5	4.46
07	1	0.89
11	7	6.25
16	1	0.89
17	1	0.89
24	7	6.25
27	5	4.46
35	2	1.79
40	37	33.04
42	4	3.57
46	8	7.14
53	3	2.68
55	3	2.68
60	6	5.36
63	2	1.79
74	2	1.79
76	1	0.89
77	1	0.89
82	6	5.36
84	1	0.89
96	1	0.89
Total	112	100.00

- **a012_1_5_ : City**

	No	%
01	5	8.77
04	3	5.26
11	2	3.51
16	3	5.26
24	1	1.75
25	1	1.75
27	2	3.51
35	1	1.75
40	12	21.05
42	1	1.75
46	7	12.28
51	1	1.75
53	1	1.75
60	2	3.51
63	3	5.26
67	1	1.75
74	2	3.51
76	1	1.75
82	5	8.77

84	1	1.75
86	1	1.75
96	1	1.75
Total	57	100.00

- **a012_1_6_ : City**

	No	%
01	1	3.70
11	1	3.70
16	1	3.70
24	2	7.41
35	1	3.70
40	9	33.33
42	1	3.70
46	1	3.70
49	2	7.41
53	3	11.11
55	1	3.70
60	1	3.70
63	1	3.70
74	1	3.70
82	1	3.70
Total	27	100.00

- **a012_1_7_ : City**

	No	%
40	3	33.33
46	1	11.11
49	2	22.22
63	2	22.22
82	1	11.11
Total	9	100.00

- **a012_1_8_ : City**

	No	%
25	1	100.00
Total	1	100.00

- **a012_1_9_ : City**

	No	%
40	1	50.00

99	1	50.00
Total	2	100.00

- **a012_1_10_ : City**

	No	%
27	1	100.00
Total	1	100.00

- **a012_2_ : Location of Current Hukou**

	No	%
1 This Household	1,394	76.72
2 This Village/Neighborhood	234	12.88
3 Another Village/Neighborhood in this County/City	124	6.82
4 Another County/City in this Province	33	1.82
5 Another Province	32	1.76
Total	1,817	100.00

- **a012_2_1_ : County**

	No	%
02	1	16.67
06	1	16.67
59	1	16.67
63	3	50.00
Total	6	100.00

- **a012_2_2_ : County**

	No	%
02	3	10.34
03	2	6.90
04	1	3.45
08	2	6.90
16	1	3.45
28	2	6.90
31	2	6.90
38	1	3.45
50	1	3.45
54	1	3.45
57	1	3.45
59	2	6.90
63	8	27.59
81	1	3.45

92	1	3.45
Total	29	100.00

- **a012_2_3_ : County**

	No	%
02	5	3.88
03	1	0.78
04	12	9.30
06	4	3.10
08	1	0.78
13	2	1.55
16	6	4.65
28	4	3.10
31	7	5.43
37	1	0.78
39	6	4.65
43	1	0.78
46	3	2.33
51	1	0.78
54	4	3.10
57	2	1.55
58	1	0.78
59	2	1.55
63	47	36.43
73	1	0.78
75	1	0.78
76	6	4.65
78	1	0.78
81	3	2.33
90	2	1.55
91	1	0.78
92	3	2.33
98	1	0.78
Total	129	100.00

- **a012_2_4_ : County**

	No	%
02	6	6.00
03	1	1.00
04	7	7.00
06	4	4.00
13	1	1.00
16	7	7.00
23	1	1.00
28	3	3.00
31	6	6.00
39	3	3.00

43	4	4.00
44	1	1.00
46	1	1.00
49	1	1.00
51	1	1.00
54	8	8.00
57	3	3.00
59	4	4.00
63	27	27.00
71	1	1.00
75	1	1.00
76	2	2.00
78	1	1.00
81	2	2.00
90	1	1.00
92	1	1.00
97	1	1.00
98	1	1.00
Total	100	100.00

- **a012_2_5_ : County**

	No	%
04	3	5.66
06	2	3.77
16	1	1.89
28	5	9.43
31	3	5.66
37	3	5.66
43	3	5.66
44	1	1.89
46	1	1.89
54	2	3.77
56	1	1.89
59	7	13.21
63	14	26.42
76	3	5.66
78	1	1.89
81	1	1.89
92	1	1.89
98	1	1.89
Total	53	100.00

- **a012_2_6_ : County**

	No	%
02	1	4.76
04	2	9.52
28	1	4.76

37	1	4.76
46	1	4.76
51	1	4.76
54	1	4.76
59	1	4.76
63	7	33.33
78	1	4.76
81	2	9.52
92	1	4.76
98	1	4.76
Total	21	100.00

- **a012_2_7_ : County**

	No	%
04	3	37.50
39	1	12.50
44	1	12.50
46	1	12.50
63	2	25.00
Total	8	100.00

- **a012_2_8_ : County**

	No	%
28	1	100.00
Total	1	100.00

- **a012_2_9_ : County**

	No	%
63	1	100.00
Total	1	100.00

- **a012_2_10_ : County**

	No	%
76	1	100.00
Total	1	100.00

- **a012_3_ : Location of Current Hukou**

	No	%

1 This Household	4,514	77.07
2 This Village/Neighborhood	646	11.03
3 Another Village/Neighborhood in this County/City	426	7.27
4 Another County/City in this Province	146	2.49
5 Another Province	122	2.08
6 Abroad	3	0.05
Total	5,857	100.00

- **a012_3_1_ : Province**

	No	%
03	1	14.29
05	1	14.29
07	2	28.57
12	1	14.29
26	1	14.29
27	1	14.29
Total	7	100.00

- **a012_3_2_ : Province**

	No	%
01	3	9.38
03	1	3.13
07	3	9.38
09	1	3.13
11	1	3.13
12	1	3.13
15	1	3.13
16	3	9.38
18	1	3.13
21	2	6.25
24	1	3.13
26	2	6.25
27	1	3.13
28	3	9.38
29	2	6.25
32	3	9.38
33	2	6.25
34	1	3.13
Total	32	100.00

- **a012_3_3_ : Province**

	No	%
01	5	4.10
02	1	0.82

03	3	2.46
05	9	7.38
06	5	4.10
07	2	1.64
08	2	1.64
09	10	8.20
10	4	3.28
11	6	4.92
12	5	4.10
13	1	0.82
14	3	2.46
15	10	8.20
16	2	1.64
17	6	4.92
18	8	6.56
19	3	2.46
20	5	4.10
21	3	2.46
23	1	0.82
24	7	5.74
25	1	0.82
26	4	3.28
27	5	4.10
28	1	0.82
29	2	1.64
32	3	2.46
33	3	2.46
34	2	1.64
Total	122	100.00

- **a012_3_4_ : Province**

	No	%
01	2	2.25
02	1	1.12
03	2	2.25
05	12	13.48
06	4	4.49
07	3	3.37
08	3	3.37
09	2	2.25
10	3	3.37
11	4	4.49
12	4	4.49
13	2	2.25
14	4	4.49
16	4	4.49
17	1	1.12
18	2	2.25
20	3	3.37
24	3	3.37

25	2	2.25
26	5	5.62
27	3	3.37
28	2	2.25
29	4	4.49
32	5	5.62
33	5	5.62
34	4	4.49
Total	89	100.00

- **a012_3_5_ : Province**

	No	%
01	1	3.57
05	4	14.29
08	1	3.57
10	2	7.14
11	3	10.71
16	2	7.14
17	1	3.57
18	1	3.57
19	1	3.57
21	3	10.71
27	3	10.71
29	2	7.14
33	4	14.29
Total	28	100.00

- **a012_3_6_ : Province**

	No	%
01	1	4.55
05	1	4.55
06	2	9.09
07	1	4.55
08	1	4.55
10	2	9.09
11	4	18.18
12	1	4.55
15	3	13.64
20	1	4.55
26	2	9.09
27	1	4.55
33	2	9.09
Total	22	100.00

- **a012_3_7_ : Province**

	No	%
11	1	12.50
12	1	12.50
13	1	12.50
15	2	25.00
27	1	12.50
32	1	12.50
33	1	12.50
Total	8	100.00

- **a012_3_8_ : Province**

	No	%
01	2	22.22
15	3	33.33
17	1	11.11
20	1	11.11
24	1	11.11
26	1	11.11
Total	9	100.00

- **a012_3_9_ : Province**

	No	%
15	1	50.00
24	1	50.00
Total	2	100.00

- **a012_3_10_ : Province**

	No	%
15	1	100.00
Total	1	100.00

- **a012_4_ : Location of Current Hukou**

	No	%
1 This Household	3,181	75.29
2 This Village/Neighborhood	423	10.01
3 Another Village/Neighborhood in this County/City	418	9.89
4 Another County/City in this Province	112	2.65
5 Another Province	89	2.11
6 Abroad	2	0.05

Total	4,225	100.00

- **a012_4_1_ : City**

	No	%
11	1	14.29
24	1	14.29
46	1	14.29
49	1	14.29
63	1	14.29
74	1	14.29
99	1	14.29
Total	7	100.00

- **a012_4_2_ : City**

	No	%
04	2	6.67
05	1	3.33
24	1	3.33
27	1	3.33
40	11	36.67
46	1	3.33
49	2	6.67
53	3	10.00
55	1	3.33
60	1	3.33
63	1	3.33
74	2	6.67
77	1	3.33
99	2	6.67
Total	30	100.00

- **a012_4_3_ : City**

	No	%
01	4	3.57
04	4	3.57
11	4	3.57
16	2	1.79
17	1	0.89
24	7	6.25
35	1	0.89
40	51	45.54
46	5	4.46
49	4	3.57
53	4	3.57

55	6	5.36
56	2	1.79
60	2	1.79
63	2	1.79
66	1	0.89
67	1	0.89
74	4	3.57
82	3	2.68
86	1	0.89
96	1	0.89
99	2	1.79
Total	112	100.00

- **a012_4_4_ : City**

	No	%
01	3	3.95
02	1	1.32
04	1	1.32
05	2	2.63
11	3	3.95
24	11	14.47
27	1	1.32
40	24	31.58
42	2	2.63
46	5	6.58
49	6	7.89
51	1	1.32
53	5	6.58
55	4	5.26
60	1	1.32
66	1	1.32
82	1	1.32
86	1	1.32
96	1	1.32
99	2	2.63
Total	76	100.00

- **a012_4_5_ : City**

	No	%
01	4	16.00
11	1	4.00
24	1	4.00
27	2	8.00
40	10	40.00
46	1	4.00
53	3	12.00
74	1	4.00

82	2	8.00
Total	25	100.00

- **a012_4_6_ : City**

	No	%
01	2	10.00
04	1	5.00
11	3	15.00
18	1	5.00
24	3	15.00
27	1	5.00
40	5	25.00
49	1	5.00
60	1	5.00
82	1	5.00
96	1	5.00
Total	20	100.00

- **a012_4_7_ : City**

	No	%
04	1	12.50
11	1	12.50
40	4	50.00
53	1	12.50
60	1	12.50
Total	8	100.00

- **a012_4_8_ : City**

	No	%
02	1	12.50
04	1	12.50
11	1	12.50
40	2	25.00
66	1	12.50
77	1	12.50
82	1	12.50
Total	8	100.00

- **a012_4_9_ : City**

	No	%
11	1	50.00

82	1	50.00
Total	2	100.00

- **a012_4_10_ : City**

	No	%
82	1	100.00
Total	1	100.00

- **a012_5_ : Location of Current Hukou**

	No	%
1 This Household	2,172	79.76
2 This Village/Neighborhood	235	8.63
3 Another Village/Neighborhood in this County/City	215	7.90
4 Another County/City in this Province	57	2.09
5 Another Province	44	1.62
Total	2,723	100.00

- **a012_5_1_ : County**

	No	%
04	1	14.29
06	1	14.29
49	1	14.29
63	2	28.57
76	2	28.57
Total	7	100.00

- **a012_5_2_ : County**

	No	%
02	1	4.17
04	1	4.17
06	1	4.17
08	1	4.17
16	1	4.17
23	1	4.17
31	3	12.50
33	1	4.17
43	2	8.33
59	1	4.17
63	9	37.50
76	1	4.17
81	1	4.17

Total	24	100.00

- **a012_5_3_ : County**

	No	%
02	2	2.47
04	3	3.70
06	1	1.23
08	4	4.94
23	2	2.47
28	1	1.23
31	5	6.17
33	1	1.23
43	2	2.47
49	1	1.23
51	1	1.23
54	6	7.41
56	1	1.23
57	2	2.47
59	7	8.64
63	27	33.33
75	1	1.23
76	8	9.88
81	2	2.47
91	1	1.23
92	1	1.23
99	2	2.47
Total	81	100.00

- **a012_5_4_ : County**

	No	%
02	3	4.76
03	1	1.59
06	2	3.17
16	2	3.17
28	5	7.94
31	4	6.35
39	1	1.59
43	3	4.76
44	1	1.59
46	3	4.76
49	1	1.59
51	1	1.59
54	1	1.59
59	6	9.52
63	22	34.92
76	2	3.17
78	1	1.59

81	2	3.17
92	1	1.59
97	1	1.59
Total	63	100.00

- **a012_5_5_ : County**

	No	%
02	2	11.11
06	1	5.56
16	1	5.56
28	1	5.56
31	2	11.11
46	1	5.56
56	1	5.56
59	1	5.56
63	8	44.44
Total	18	100.00

- **a012_5_6_ : County**

	No	%
06	3	15.79
39	1	5.26
43	1	5.26
44	1	5.26
46	1	5.26
54	2	10.53
63	10	52.63
Total	19	100.00

- **a012_5_7_ : County**

	No	%
04	1	14.29
31	1	14.29
59	1	14.29
63	4	57.14
Total	7	100.00

- **a012_5_8_ : County**

	No	%
46	1	33.33
54	1	33.33

63	1	33.33
Total	3	100.00

- **a012_5_9_ : County**

No Observations

- **a012_5_10_ : County**

No Observations

- **a012_6_ : Location of Current Hukou**

	No	%
1 This Household	1,076	81.09
2 This Village/Neighborhood	118	8.89
3 Another Village/Neighborhood in this County/City	82	6.18
4 Another County/City in this Province	27	2.03
5 Another Province	23	1.73
6 Abroad	1	0.08
Total	1,327	100.00

- **a012_7_ : Location of Current Hukou**

	No	%
1 This Household	407	80.43
2 This Village/Neighborhood	39	7.71
3 Another Village/Neighborhood in this County/City	42	8.30
4 Another County/City in this Province	9	1.78
5 Another Province	9	1.78
Total	506	100.00

- **a012_8_ : Location of Current Hukou**

	No	%
1 This Household	191	80.25
2 This Village/Neighborhood	21	8.82
3 Another Village/Neighborhood in this County/City	16	6.72
4 Another County/City in this Province	1	0.42
5 Another Province	9	3.78
Total	238	100.00

- **a012_9_ : Location of Current Hukou**

	No	%
1 This Household	111	82.84
2 This Village/Neighborhood	6	4.48
3 Another Village/Neighborhood in this County/City	12	8.96
4 Another County/City in this Province	2	1.49
5 Another Province	2	1.49
6 Abroad	1	0.75
Total	134	100.00

- **a012_10_ : Location of Current Hukou**

	No	%
1 This Household	60	90.91
2 This Village/Neighborhood	2	3.03
3 Another Village/Neighborhood in this County/City	2	3.03
4 Another County/City in this Province	1	1.52
5 Another Province	1	1.52
Total	66	100.00

- **a012_11_ : Location of Current Hukou**

	No	%
1 This Household	26	86.67
2 This Village/Neighborhood	2	6.67
3 Another Village/Neighborhood in this County/City	2	6.67
Total	30	100.00

- **a012_12_ : Location of Current Hukou**

	No	%
1 This Household	15	78.95
2 This Village/Neighborhood	3	15.79
3 Another Village/Neighborhood in this County/City	1	5.26
Total	19	100.00

- **a012_13_ : Location of Current Hukou**

	No	%
1 This Household	6	66.67
2 This Village/Neighborhood	2	22.22
3 Another Village/Neighborhood in this County/City	1	11.11
Total	9	100.00

- **a012_14_ : Location of Current Hukou**

	No	%
1 This Household	4	80.00
2 This Village/Neighborhood	1	20.00
Total	5	100.00

- **a012_15_ : Location of Current Hukou**

	No	%
1 This Household	1	50.00
2 This Village/Neighborhood	1	50.00
Total	2	100.00

- **a012_16_ : Location of Current Hukou**

	No	%
2 This Village/Neighborhood	1	100.00
Total	1	100.00

- **a013_1_ : Is He/She Still in School Now**

	No	%
1 Yes	18	2.30
2 No	765	97.70
Total	783	100.00

- **a013_2_ : Is He/She Still in School Now**

	No	%
1 Yes	258	14.62
2 No	1,507	85.38
Total	1,765	100.00

- **a013_3_ : Is He/She Still in School Now**

	No	%
1 Yes	1,244	22.52
2 No	4,279	77.48
Total	5,523	100.00

- **a013_4_ : Is He/She Still in School Now**

	No	%
1 Yes	1,065	27.80
2 No	2,766	72.20
Total	3,831	100.00

- **a013_5_ : Is He/She Still in School Now**

	No	%
1 Yes	881	43.53
2 No	1,143	56.47
Total	2,024	100.00

- **a013_6_ : Is He/She Still in School Now**

	No	%
1 Yes	345	37.34
2 No	579	62.66
Total	924	100.00

- **a013_7_ : Is He/She Still in School Now**

	No	%
1 Yes	119	31.32
2 No	261	68.68
Total	380	100.00

- **a013_8_ : Is He/She Still in School Now**

	No	%
1 Yes	44	26.51
2 No	122	73.49
Total	166	100.00

- **a013_9_ : Is He/She Still in School Now**

	No	%
1 Yes	19	20.88
2 No	72	79.12
Total	91	100.00

- **a013_10_ : Is He/She Still in School Now**

	No	%
1 Yes	12	30.77
2 No	27	69.23
Total	39	100.00

- **a013_11_ : Is He/She Still in School Now**

	No	%
1 Yes	6	33.33
2 No	12	66.67
Total	18	100.00

- **a013_12_ : Is He/She Still in School Now**

	No	%
1 Yes	4	33.33
2 No	8	66.67
Total	12	100.00

- **a013_13_ : Is He/She Still in School Now**

	No	%
1 Yes	3	60.00
2 No	2	40.00
Total	5	100.00

- **a013_14_ : Is He/She Still in School Now**

	No	%
2 No	2	100.00
Total	2	100.00

- **a013_15_ : Is He/She Still in School Now**

	No	%
2 No	2	100.00
Total	2	100.00

- **a013_16_ : Is He/She Still in School Now**

	No	%
2 No	1	100.00
Total	1	100.00

- **a014_1_ : Level of Schooling**

	No	%
1 Primary school grade 1	1	5.56
2 Primary school grade 2	1	5.56
5 Primary school grade 5	3	16.67
6 Primary school grade 6	1	5.56
7 Middle school grade 1	2	11.11
8 Middle school grade 2	2	11.11
9 Middle school grade 3	2	11.11
11 High school, grade 1	2	11.11
12 High school, grade 2	1	5.56
13 High school, grade 3	1	5.56
14 Vocational/technical high school year 1	1	5.56
19 College year 3	1	5.56
Total	18	100.00

- **a014_2_ : Level of Schooling**

	No	%
1 Primary school grade 1	33	12.74
2 Primary school grade 2	9	3.47
3 Primary school grade 3	22	8.49
4 Primary school grade 4	16	6.18
5 Primary school grade 5	22	8.49
6 Primary school grade 6	13	5.02
7 Middle school grade 1	24	9.27
8 Middle school grade 2	24	9.27
9 Middle school grade 3	17	6.56
10 Middle school grade 4	1	0.39
11 High school, grade 1	10	3.86
12 High school, grade 2	13	5.02
13 High school, grade 3	19	7.34
14 Vocational/technical high school year 1	2	0.77
15 Vocational/technical high school year 2	1	0.39
16 Vocational/technical high school year 3	1	0.39
17 College year 1	13	5.02
18 College year 2	8	3.09
19 College year 3	8	3.09
20 College year 4	1	0.39
23 Masters degree	2	0.77
Total	259	100.00

- **a014_3_ : Level of Schooling**

	No	%
1 Primary school grade 1	143	11.45
2 Primary school grade 2	71	5.68
3 Primary school grade 3	70	5.60
4 Primary school grade 4	82	6.57
5 Primary school grade 5	68	5.44
6 Primary school grade 6	65	5.20
7 Middle school grade 1	88	7.05
8 Middle school grade 2	90	7.21
9 Middle school grade 3	78	6.24
10 Middle school grade 4	5	0.40
11 High school, grade 1	68	5.44
12 High school, grade 2	52	4.16
13 High school, grade 3	89	7.13
14 Vocational/technical high school year 1	12	0.96
15 Vocational/technical high school year 2	11	0.88
16 Vocational/technical high school year 3	11	0.88
17 College year 1	46	3.68
18 College year 2	65	5.20
19 College year 3	70	5.60
20 College year 4	34	2.72
21 College year 5	1	0.08
23 Masters degree	26	2.08
24 Doctoral degree/Ph.D. degree	4	0.32
Total	1,249	100.00

- **a014_4_ : Level of Schooling**

	No	%
1 Primary school grade 1	132	12.42
2 Primary school grade 2	86	8.09
3 Primary school grade 3	68	6.40
4 Primary school grade 4	84	7.90
5 Primary school grade 5	78	7.34
6 Primary school grade 6	74	6.96
7 Middle school grade 1	73	6.87
8 Middle school grade 2	78	7.34
9 Middle school grade 3	67	6.30
10 Middle school grade 4	3	0.28
11 High school, grade 1	48	4.52
12 High school, grade 2	29	2.73
13 High school, grade 3	48	4.52
14 Vocational/technical high school year 1	8	0.75
15 Vocational/technical high school year 2	8	0.75
16 Vocational/technical high school year 3	9	0.85
17 College year 1	38	3.57
18 College year 2	53	4.99
19 College year 3	44	4.14

20 College year 4	19	1.79
23 Masters degree	15	1.41
24 Doctoral degree/Ph.D. degree	1	0.09
Total	1,063	100.00

- **a014_5_ : Level of Schooling**

	No	%
1 Primary school grade 1	173	19.70
2 Primary school grade 2	93	10.59
3 Primary school grade 3	76	8.66
4 Primary school grade 4	71	8.09
5 Primary school grade 5	63	7.18
6 Primary school grade 6	49	5.58
7 Middle school grade 1	61	6.95
8 Middle school grade 2	62	7.06
9 Middle school grade 3	50	5.69
11 High school, grade 1	28	3.19
12 High school, grade 2	28	3.19
13 High school, grade 3	31	3.53
14 Vocational/technical high school year 1	5	0.57
15 Vocational/technical high school year 2	7	0.80
16 Vocational/technical high school year 3	6	0.68
17 College year 1	24	2.73
18 College year 2	23	2.62
19 College year 3	22	2.51
20 College year 4	1	0.11
21 College year 5	2	0.23
23 Masters degree	2	0.23
24 Doctoral degree/Ph.D. degree	1	0.11
Total	878	100.00

- **a014_6_ : Level of Schooling**

	No	%
1 Primary school grade 1	82	23.84
2 Primary school grade 2	31	9.01
3 Primary school grade 3	35	10.17
4 Primary school grade 4	22	6.40
5 Primary school grade 5	22	6.40
6 Primary school grade 6	23	6.69
7 Middle school grade 1	26	7.56
8 Middle school grade 2	27	7.85
9 Middle school grade 3	13	3.78
11 High school, grade 1	15	4.36
12 High school, grade 2	8	2.33
13 High school, grade 3	9	2.62
16 Vocational/technical high school year 3	3	0.87
17 College year 1	4	1.16

18 College year 2	7	2.03
19 College year 3	11	3.20
20 College year 4	2	0.58
23 Masters degree	3	0.87
24 Doctoral degree/Ph.D. degree	1	0.29
Total	344	100.00

- **a014_7_ : Level of Schooling**

	No	%
1 Primary school grade 1	24	20.34
2 Primary school grade 2	12	10.17
3 Primary school grade 3	18	15.25
4 Primary school grade 4	13	11.02
5 Primary school grade 5	9	7.63
6 Primary school grade 6	8	6.78
7 Middle school grade 1	6	5.08
8 Middle school grade 2	7	5.93
9 Middle school grade 3	5	4.24
12 High school, grade 2	1	0.85
13 High school, grade 3	1	0.85
15 Vocational/technical high school year 2	1	0.85
17 College year 1	4	3.39
18 College year 2	4	3.39
19 College year 3	5	4.24
Total	118	100.00

- **a014_8_ : Level of Schooling**

	No	%
1 Primary school grade 1	15	33.33
2 Primary school grade 2	3	6.67
3 Primary school grade 3	5	11.11
4 Primary school grade 4	3	6.67
5 Primary school grade 5	4	8.89
6 Primary school grade 6	3	6.67
7 Middle school grade 1	4	8.89
8 Middle school grade 2	1	2.22
9 Middle school grade 3	2	4.44
11 High school, grade 1	1	2.22
14 Vocational/technical high school year 1	1	2.22
15 Vocational/technical high school year 2	1	2.22
19 College year 3	2	4.44
Total	45	100.00

- **a014_9_ : Level of Schooling**

	No	%
1 Primary school grade 1	7	36.84
2 Primary school grade 2	1	5.26
3 Primary school grade 3	3	15.79
6 Primary school grade 6	1	5.26
7 Middle school grade 1	3	15.79
8 Middle school grade 2	2	10.53
19 College year 3	2	10.53
Total	19	100.00

- **a014_10_ : Level of Schooling**

	No	%
2 Primary school grade 2	1	8.33
3 Primary school grade 3	3	25.00
5 Primary school grade 5	2	16.67
6 Primary school grade 6	1	8.33
7 Middle school grade 1	1	8.33
8 Middle school grade 2	1	8.33
11 High school, grade 1	1	8.33
17 College year 1	1	8.33
18 College year 2	1	8.33
Total	12	100.00

- **a014_11_ : Level of Schooling**

	No	%
1 Primary school grade 1	1	16.67
2 Primary school grade 2	1	16.67
4 Primary school grade 4	2	33.33
7 Middle school grade 1	1	16.67
17 College year 1	1	16.67
Total	6	100.00

- **a014_12_ : Level of Schooling**

	No	%
4 Primary school grade 4	2	50.00
7 Middle school grade 1	1	25.00
19 College year 3	1	25.00
Total	4	100.00

- **a014_13_ : Level of Schooling**

	No	%

2 Primary school grade 2	1	33.33
3 Primary school grade 3	1	33.33
5 Primary school grade 5	1	33.33
Total	3	100.00

- **a015_1_ : Education Completed**

	No	%
1 No formal education illiterate	149	19.61
2 Did not finish primary school but capable of reading or writing	95	12.50
3 Sishu/home school	4	0.53
4 Graduate from elementary school	177	23.29
5 Graduate from middle school	213	28.03
6 Graduate from high school	48	6.32
7 Graduate from vocational school	36	4.74
8 Graduate from Two/Three Year College / Associate degree	18	2.37
9 Graduate from Four Year College / Bachelors degree	17	2.24
10 Graduate from Post-graduate, Masters degree	2	0.26
11 Graduate from Post-graduate, Doctoral degree/Ph.D.	1	0.13
Total	760	100.00

- **a015_2_ : Education Completed**

	No	%
1 No formal education illiterate	196	13.25
2 Did not finish primary school but capable of reading or writing	137	9.26
3 Sishu/home school	5	0.34
4 Graduate from elementary school	326	22.04
5 Graduate from middle school	494	33.40
6 Graduate from high school	117	7.91
7 Graduate from vocational school	82	5.54
8 Graduate from Two/Three Year College / Associate degree	67	4.53
9 Graduate from Four Year College / Bachelors degree	51	3.45
10 Graduate from Post-graduate, Masters degree	3	0.20
11 Graduate from Post-graduate, Doctoral degree/Ph.D.	1	0.07
Total	1,479	100.00

- **a015_3_ : Education Completed**

	No	%
1 No formal education illiterate	286	6.78
2 Did not finish primary school but capable of reading or writing	247	5.86
3 Sishu/home school	6	0.14
4 Graduate from elementary school	761	18.05
5 Graduate from middle school	1,548	36.71
6 Graduate from high school	385	9.13

7 Graduate from vocational school	348	8.25
8 Graduate from Two/Three Year College / Associate degree	343	8.13
9 Graduate from Four Year College / Bachelors degree	271	6.43
10 Graduate from Post-graduate, Masters degree	21	0.50
11 Graduate from Post-graduate, Doctoral degree/Ph.D.	1	0.02
Total	4,217	100.00

- **a015_4_ : Education Completed**

	No	%
1 No formal education illiterate	180	6.72
2 Did not finish primary school but capable of reading or writing	119	4.44
3 Sishu/home school	3	0.11
4 Graduate from elementary school	492	18.37
5 Graduate from middle school	1,162	43.39
6 Graduate from high school	223	8.33
7 Graduate from vocational school	186	6.95
8 Graduate from Two/Three Year College / Associate degree	176	6.57
9 Graduate from Four Year College / Bachelors degree	129	4.82
10 Graduate from Post-graduate, Masters degree	8	0.30
Total	2,678	100.00

- **a015_5_ : Education Completed**

	No	%
1 No formal education illiterate	80	7.45
2 Did not finish primary school but capable of reading or writing	64	5.96
3 Sishu/home school	2	0.19
4 Graduate from elementary school	218	20.30
5 Graduate from middle school	446	41.53
6 Graduate from high school	82	7.64
7 Graduate from vocational school	75	6.98
8 Graduate from Two/Three Year College / Associate degree	58	5.40
9 Graduate from Four Year College / Bachelors degree	45	4.19
10 Graduate from Post-graduate, Masters degree	4	0.37
Total	1,074	100.00

- **a015_6_ : Education Completed**

	No	%
1 No formal education illiterate	44	8.19
2 Did not finish primary school but capable of reading or writing	23	4.28
3 Sishu/home school	2	0.37
4 Graduate from elementary school	129	24.02
5 Graduate from middle school	215	40.04
6 Graduate from high school	39	7.26
7 Graduate from vocational school	38	7.08

8 Graduate from Two/Three Year College / Associate degree	22	4.10
9 Graduate from Four Year College / Bachelors degree	25	4.66
Total	537	100.00

- **a015_7_ : Education Completed**

	No	%
1 No formal education illiterate	32	12.90
2 Did not finish primary school but capable of reading or writing	11	4.44
4 Graduate from elementary school	48	19.35
5 Graduate from middle school	102	41.13
6 Graduate from high school	23	9.27
7 Graduate from vocational school	15	6.05
8 Graduate from Two/Three Year College / Associate degree	11	4.44
9 Graduate from Four Year College / Bachelors degree	5	2.02
10 Graduate from Post-graduate, Masters degree	1	0.40
Total	248	100.00

- **a015_8_ : Education Completed**

	No	%
1 No formal education illiterate	9	8.04
2 Did not finish primary school but capable of reading or writing	4	3.57
4 Graduate from elementary school	18	16.07
5 Graduate from middle school	65	58.04
6 Graduate from high school	5	4.46
7 Graduate from vocational school	5	4.46
8 Graduate from Two/Three Year College / Associate degree	4	3.57
9 Graduate from Four Year College / Bachelors degree	2	1.79
Total	112	100.00

- **a015_9_ : Education Completed**

	No	%
1 No formal education illiterate	7	10.61
2 Did not finish primary school but capable of reading or writing	5	7.58
4 Graduate from elementary school	12	18.18
5 Graduate from middle school	32	48.48
6 Graduate from high school	6	9.09
7 Graduate from vocational school	1	1.52
8 Graduate from Two/Three Year College / Associate degree	1	1.52
9 Graduate from Four Year College / Bachelors degree	2	3.03
Total	66	100.00

- **a015_10_ : Education Completed**

	No	%
1 No formal education illiterate	6	23.08
4 Graduate from elementary school	4	15.38
5 Graduate from middle school	14	53.85
7 Graduate from vocational school	1	3.85
9 Graduate from Four Year College / Bachelors degree	1	3.85
Total	26	100.00

- **a015_11_ : Education Completed**

	No	%
1 No formal education illiterate	4	33.33
2 Did not finish primary school but capable of reading or writing	1	8.33
4 Graduate from elementary school	2	16.67
5 Graduate from middle school	3	25.00
6 Graduate from high school	1	8.33
8 Graduate from Two/Three Year College / Associate degree	1	8.33
Total	12	100.00

- **a015_12_ : Education Completed**

	No	%
1 No formal education illiterate	1	12.50
2 Did not finish primary school but capable of reading or writing	1	12.50
5 Graduate from middle school	5	62.50
7 Graduate from vocational school	1	12.50
Total	8	100.00

- **a015_13_ : Education Completed**

	No	%
5 Graduate from middle school	2	100.00
Total	2	100.00

- **a015_14_ : Education Completed**

	No	%
5 Graduate from middle school	2	100.00
Total	2	100.00

- **a015_15_ : Education Completed**

	No	%
1 No formal education illiterate	1	50.00
5 Graduate from middle school	1	50.00
Total	2	100.00

- **a015_16_ : Education Completed**

	No	%
2 Did not finish primary school but capable of reading or writing	1	100.00
Total	1	100.00

- **a016_1_ : Whether One or More Months Away**

	No	%
1 Yes	89	11.51
2 No	684	88.49
Total	773	100.00

- **a016_2_ : Whether One or More Months Away**

	No	%
1 Yes	357	22.59
2 No	1,223	77.41
Total	1,580	100.00

- **a016_3_ : Whether One or More Months Away**

	No	%
1 Yes	1,800	38.06
2 No	2,929	61.94
Total	4,729	100.00

- **a016_4_ : Whether One or More Months Away**

	No	%
1 Yes	1,257	40.96
2 No	1,812	59.04
Total	3,069	100.00

- **a016_5_ : Whether One or More Months Away**

	No	%
1 Yes	709	55.26
2 No	574	44.74
Total	1,283	100.00

- **a016_6_ : Whether One or More Months Away**

	No	%
1 Yes	379	60.64
2 No	246	39.36
Total	625	100.00

- **a016_7_ : Whether One or More Months Away**

	No	%
1 Yes	170	64.15
2 No	95	35.85
Total	265	100.00

- **a016_8_ : Whether One or More Months Away**

	No	%
1 Yes	75	63.56
2 No	43	36.44
Total	118	100.00

- **a016_9_ : Whether One or More Months Away**

	No	%
1 Yes	47	66.20
2 No	24	33.80
Total	71	100.00

- **a016_10_ : Whether One or More Months Away**

	No	%
1 Yes	20	74.07
2 No	7	25.93
Total	27	100.00

- **a016_11_ : Whether One or More Months Away**

	No	%
1 Yes	9	69.23
2 No	4	30.77
Total	13	100.00

- **a016_12_ : Whether One or More Months Away**

	No	%
1 Yes	4	44.44
2 No	5	55.56
Total	9	100.00

- **a016_13_ : Whether One or More Months Away**

	No	%
1 Yes	2	100.00
Total	2	100.00

- **a016_14_ : Whether One or More Months Away**

	No	%
1 Yes	2	100.00
Total	2	100.00

- **a016_15_ : Whether One or More Months Away**

	No	%
1 Yes	1	50.00
2 No	1	50.00
Total	2	100.00

- **a016_16_ : Whether One or More Months Away**

	No	%
2 No	1	100.00
Total	1	100.00

- **a017_1_ : How Many Months**

	No	%

0	1	1.12
1	8	8.99
2	19	21.35
3	9	10.11
4	4	4.49
5	8	8.99
6	8	8.99
7	4	4.49
8	7	7.87
9	1	1.12
10	7	7.87
11	6	6.74
12	7	7.87
Total	89	100.00

- **a017_2_ : How Many Months**

	No	%
0	4	1.11
1	15	4.16
2	20	5.54
3	33	9.14
4	17	4.71
5	17	4.71
6	32	8.86
7	12	3.32
8	16	4.43
9	23	6.37
10	48	13.30
11	59	16.34
12	65	18.01
Total	361	100.00

- **a017_3_ : How Many Months**

	No	%
0	9	0.50
1	64	3.54
2	84	4.65
3	80	4.42
4	78	4.31
5	78	4.31
6	178	9.85
7	30	1.66
8	103	5.70
9	127	7.02
10	263	14.55
11	311	17.20

12	401	22.18
14	1	0.06
16	1	0.06
Total	1,808	100.00

- **a017_4_ : How Many Months**

	No	%
0	2	0.16
1	25	1.99
2	47	3.73
3	52	4.13
4	35	2.78
5	44	3.49
6	98	7.78
7	25	1.99
8	86	6.83
9	101	8.02
10	201	15.97
11	265	21.05
12	276	21.92
16	1	0.08
20	1	0.08
Total	1,259	100.00

- **a017_5_ : How Many Months**

	No	%
0	1	0.14
1	10	1.40
2	19	2.66
3	29	4.06
4	18	2.52
5	20	2.80
6	44	6.16
7	17	2.38
8	45	6.30
9	49	6.86
10	126	17.65
11	173	24.23
12	162	22.69
18	1	0.14
Total	714	100.00

- **a017_6_ : How Many Months**

	No	%

0	1	0.26
1	4	1.06
2	10	2.64
3	9	2.37
4	9	2.37
5	8	2.11
6	23	6.07
7	14	3.69
8	22	5.80
9	16	4.22
10	67	17.68
11	102	26.91
12	94	24.80
Total	379	100.00

- **a017_7_ : How Many Months**

	No	%
2	3	1.76
3	5	2.94
4	5	2.94
5	7	4.12
6	6	3.53
7	2	1.18
8	11	6.47
9	6	3.53
10	31	18.24
11	49	28.82
12	45	26.47
Total	170	100.00

- **a017_8_ : How Many Months**

	No	%
2	1	1.33
3	2	2.67
4	2	2.67
6	2	2.67
7	3	4.00
8	3	4.00
10	14	18.67
11	23	30.67
12	25	33.33
Total	75	100.00

- **a017_9_ : How Many Months**

	No	%
1	1	2.13
3	2	4.26
6	2	4.26
7	2	4.26
8	2	4.26
10	8	17.02
11	14	29.79
12	16	34.04
Total	47	100.00

- **a017_10_ : How Many Months**

	No	%
3	1	5.00
6	1	5.00
8	1	5.00
9	1	5.00
10	6	30.00
11	3	15.00
12	7	35.00
Total	20	100.00

- **a017_11_ : How Many Months**

	No	%
2	1	11.11
6	1	11.11
8	1	11.11
10	1	11.11
11	2	22.22
12	3	33.33
Total	9	100.00

- **a017_12_ : How Many Months**

	No	%
6	1	25.00
10	1	25.00
11	1	25.00
12	1	25.00
Total	4	100.00

- **a017_13_ : How Many Months**

	No	%
10	1	50.00
12	1	50.00
Total	2	100.00

- **a017_14_ : How Many Months**

	No	%
10	1	50.00
12	1	50.00
Total	2	100.00

- **a017_15_ : How Many Months**

	No	%
11	1	100.00
Total	1	100.00

- **a018_1_ : Main Place**

	No	%
1 This County/City	28	31.82
2 Another County/City in This Province	25	28.41
3 Another province	34	38.64
4 Abroad	1	1.14
Total	88	100.00

- **a018_1_1_ : City**

	No	%
01	1	3.85
04	1	3.85
17	1	3.85
24	3	11.54
40	7	26.92
42	4	15.38
46	1	3.85
53	1	3.85
60	1	3.85
82	3	11.54
84	2	7.69
96	1	3.85
Total	26	100.00

- **a018_1_2_ : City**

	No	%
01	5	4.46
04	6	5.36
11	6	5.36
24	10	8.93
27	1	0.89
28	1	0.89
40	48	42.86
42	2	1.79
46	3	2.68
49	1	0.89
51	1	0.89
52	1	0.89
53	5	4.46
55	1	0.89
63	2	1.79
67	1	0.89
74	7	6.25
76	1	0.89
82	7	6.25
84	2	1.79
96	1	0.89
Total	112	100.00

- **a018_1_3_ : City**

	No	%
01	25	4.37
02	6	1.05
04	27	4.72
11	30	5.24
16	3	0.52
17	5	0.87
18	6	1.05
24	44	7.69
25	1	0.17
27	2	0.35
35	5	0.87
40	263	45.98
42	5	0.87
46	23	4.02
49	7	1.22
51	1	0.17
52	1	0.17
53	26	4.55
55	15	2.62
60	7	1.22
63	4	0.70

66	4	0.70
74	18	3.15
76	1	0.17
77	1	0.17
82	24	4.20
84	6	1.05
86	6	1.05
96	4	0.70
99	2	0.35
Total	572	100.00

- **a018_1_4_ : City**

	No	%
01	17	4.11
02	3	0.72
04	22	5.31
05	1	0.24
11	31	7.49
16	2	0.48
17	4	0.97
18	5	1.21
24	28	6.76
27	4	0.97
35	2	0.48
40	193	46.62
42	2	0.48
46	16	3.86
49	3	0.72
52	1	0.24
53	14	3.38
55	8	1.93
60	8	1.93
63	4	0.97
66	3	0.72
74	15	3.62
76	2	0.48
82	19	4.59
84	3	0.72
86	2	0.48
96	1	0.24
99	1	0.24
Total	414	100.00

- **a018_1_5_ : City**

	No	%
01	13	6.16
02	1	0.47

04	17	8.06
11	9	4.27
16	1	0.47
17	2	0.95
18	1	0.47
24	7	3.32
25	1	0.47
27	3	1.42
35	1	0.47
40	114	54.03
46	7	3.32
50	1	0.47
51	1	0.47
53	1	0.47
55	2	0.95
60	1	0.47
63	1	0.47
74	8	3.79
76	3	1.42
82	12	5.69
84	1	0.47
86	1	0.47
96	2	0.95
Total	211	100.00

- **a018_1_6_ : City**

	No	%
01	3	2.63
04	12	10.53
11	4	3.51
16	1	0.88
17	1	0.88
24	7	6.14
27	1	0.88
35	1	0.88
40	56	49.12
42	1	0.88
46	2	1.75
49	1	0.88
51	1	0.88
53	2	1.75
60	1	0.88
63	3	2.63
66	1	0.88
74	4	3.51
76	2	1.75
82	4	3.51
84	6	5.26
Total	114	100.00

- **a018_1_7_ : City**

	No	%
01	1	2.17
04	3	6.52
11	1	2.17
17	1	2.17
18	1	2.17
24	2	4.35
40	27	58.70
42	1	2.17
46	1	2.17
49	1	2.17
53	1	2.17
63	1	2.17
74	4	8.70
84	1	2.17
Total	46	100.00

- **a018_1_8_ : City**

	No	%
01	1	5.26
04	5	26.32
11	1	5.26
40	6	31.58
42	1	5.26
46	4	21.05
74	1	5.26
Total	19	100.00

- **a018_1_9_ : City**

	No	%
25	1	11.11
40	4	44.44
46	2	22.22
53	1	11.11
84	1	11.11
Total	9	100.00

- **a018_1_10_ : City**

	No	%
40	2	40.00
46	1	20.00

55	1	20.00
84	1	20.00
Total	5	100.00

- **a018_1_11_ : City**

	No	%
40	1	100.00
Total	1	100.00

- **a018_1_12_ : City**

	No	%
04	1	50.00
40	1	50.00
Total	2	100.00

- **a018_2_ : Main Place**

	No	%
1 This County/City	91	26.38
2 Another County/City in This Province	112	32.46
3 Another province	141	40.87
4 Abroad	1	0.29
Total	345	100.00

- **a018_2_1_ : County**

	No	%
04	2	9.52
06	3	14.29
31	2	9.52
43	1	4.76
46	1	4.76
57	1	4.76
59	1	4.76
63	7	33.33
76	1	4.76
78	1	4.76
81	1	4.76
Total	21	100.00

- **a018_2_2_ : County**

	No	%
02	2	2.30
04	3	3.45
06	3	3.45
08	2	2.30
16	2	2.30
28	2	2.30
31	3	3.45
37	1	1.15
39	1	1.15
43	2	2.30
44	3	3.45
46	1	1.15
51	1	1.15
54	2	2.30
56	2	2.30
57	4	4.60
58	2	2.30
59	4	4.60
63	36	41.38
67	1	1.15
76	2	2.30
78	4	4.60
92	1	1.15
99	3	3.45
Total	87	100.00

- **a018_2_3_ : County**

	No	%
02	9	1.87
03	1	0.21
04	38	7.88
06	13	2.70
08	1	0.21
13	3	0.62
16	14	2.90
28	17	3.53
31	19	3.94
37	5	1.04
38	3	0.62
39	13	2.70
43	3	0.62
44	3	0.62
46	8	1.66
50	1	0.21
51	3	0.62
54	9	1.87
56	5	1.04
57	9	1.87

58	7	1.45
59	11	2.28
63	228	47.30
73	3	0.62
75	1	0.21
76	10	2.07
78	23	4.77
81	2	0.41
90	1	0.21
91	2	0.41
92	11	2.28
97	1	0.21
99	5	1.04
Total	482	100.00

- **a018_2_4_ : County**

	No	%
02	7	2.01
03	1	0.29
04	25	7.16
06	12	3.44
13	2	0.57
16	15	4.30
28	11	3.15
31	11	3.15
37	7	2.01
38	1	0.29
39	7	2.01
43	4	1.15
44	3	0.86
46	8	2.29
49	1	0.29
54	5	1.43
56	3	0.86
57	8	2.29
58	2	0.57
59	14	4.01
63	166	47.56
75	1	0.29
76	3	0.86
78	18	5.16
81	1	0.29
92	11	3.15
97	1	0.29
99	1	0.29
Total	349	100.00

- **a018_2_5_ : County**

	No	%
02	1	0.55
04	14	7.73
06	5	2.76
16	8	4.42
28	10	5.52
31	5	2.76
37	3	1.66
38	1	0.55
39	3	1.66
43	2	1.10
45	2	1.10
46	2	1.10
54	3	1.66
56	4	2.21
57	3	1.66
58	2	1.10
59	7	3.87
63	79	43.65
73	1	0.55
75	1	0.55
76	2	1.10
78	9	4.97
81	3	1.66
91	1	0.55
92	7	3.87
99	3	1.66
Total	181	100.00

- **a018_2_6_ : County**

	No	%
03	1	1.15
04	7	8.05
06	3	3.45
16	6	6.90
28	4	4.60
31	4	4.60
37	2	2.30
39	1	1.15
43	3	3.45
44	2	2.30
56	3	3.45
57	2	2.30
59	2	2.30
63	38	43.68
76	1	1.15
78	3	3.45
81	1	1.15
92	3	3.45

99	1	1.15
Total	87	100.00

- **a018_2_7_ : County**

	No	%
04	3	7.50
06	1	2.50
28	1	2.50
31	2	5.00
37	1	2.50
39	1	2.50
46	1	2.50
54	1	2.50
56	2	5.00
57	1	2.50
59	1	2.50
63	19	47.50
75	1	2.50
76	1	2.50
78	1	2.50
99	3	7.50
Total	40	100.00

- **a018_2_8_ : County**

	No	%
04	2	16.67
16	1	8.33
31	1	8.33
56	1	8.33
59	1	8.33
63	5	41.67
78	1	8.33
Total	12	100.00

- **a018_2_9_ : County**

	No	%
06	1	20.00
28	1	20.00
63	3	60.00
Total	5	100.00

- **a018_2_10_ : County**

	No	%
63	3	100.00
Total	3	100.00

- **a018_2_11_ : County**

	No	%
63	1	100.00
Total	1	100.00

- **a018_2_12_ : County**

	No	%
63	1	100.00
Total	1	100.00

- **a018_3_ : Main Place**

	No	%
1 This County/City	438	24.93
2 Another County/City in This Province	569	32.38
3 Another province	740	42.12
4 Abroad	10	0.57
Total	1,757	100.00

- **a018_3_1_ : Province**

	No	%
01	1	2.94
03	2	5.88
05	1	2.94
06	1	2.94
08	3	8.82
09	4	11.76
11	5	14.71
12	1	2.94
13	1	2.94
15	5	14.71
17	2	5.88
18	4	11.76
19	1	2.94
20	1	2.94
23	2	5.88
Total	34	100.00

- **a018_3_2_ : Province**

	No	%
01	2	1.39
02	1	0.69
03	3	2.08
04	2	1.39
05	4	2.78
06	4	2.78
07	1	0.69
08	4	2.78
09	14	9.72
10	2	1.39
11	12	8.33
14	1	0.69
15	28	19.44
16	6	4.17
17	5	3.47
18	9	6.25
19	5	3.47
20	19	13.19
21	1	0.69
23	2	1.39
24	3	2.08
25	1	0.69
26	1	0.69
27	2	1.39
28	1	0.69
29	8	5.56
32	1	0.69
33	1	0.69
34	1	0.69
Total	144	100.00

- **a018_3_3_ : Province**

	No	%
01	11	1.47
02	5	0.67
03	21	2.82
04	2	0.27
05	12	1.61
06	18	2.41
07	9	1.21
08	15	2.01
09	66	8.85
10	18	2.41
11	64	8.58

12	7	0.94
13	5	0.67
14	9	1.21
15	179	23.99
16	17	2.28
17	7	0.94
18	50	6.70
19	30	4.02
20	86	11.53
21	6	0.80
23	2	0.27
24	7	0.94
25	10	1.34
26	14	1.88
27	12	1.61
28	11	1.47
29	28	3.75
32	10	1.34
33	7	0.94
34	8	1.07
Total	746	100.00

- **a018_3_4_ : Province**

	No	%
01	3	0.55
02	3	0.55
03	22	4.01
04	1	0.18
05	13	2.37
06	5	0.91
07	5	0.91
08	23	4.20
09	36	6.57
10	7	1.28
11	57	10.40
12	7	1.28
13	3	0.55
14	4	0.73
15	132	24.09
16	13	2.37
17	6	1.09
18	29	5.29
19	22	4.01
20	72	13.14
21	3	0.55
23	3	0.55
24	3	0.55
25	8	1.46
26	10	1.82
27	15	2.74

28	8	1.46
29	16	2.92
32	7	1.28
33	1	0.18
34	11	2.01
Total	548	100.00

- **a018_3_5_ : Province**

	No	%
01	5	1.49
02	3	0.90
03	11	3.28
05	4	1.19
06	3	0.90
07	3	0.90
08	5	1.49
09	22	6.57
10	2	0.60
11	37	11.04
12	4	1.19
14	3	0.90
15	104	31.04
16	5	1.49
17	1	0.30
18	19	5.67
19	9	2.69
20	54	16.12
21	3	0.90
23	3	0.90
24	2	0.60
25	6	1.79
26	4	1.19
27	2	0.60
28	2	0.60
29	8	2.39
32	3	0.90
33	7	2.09
34	1	0.30
Total	335	100.00

- **a018_3_6_ : Province**

	No	%
01	4	2.12
03	5	2.65
05	3	1.59
06	1	0.53
08	5	2.65

09	5	2.65
11	20	10.58
12	2	1.06
13	1	0.53
14	1	0.53
15	60	31.75
17	1	0.53
18	15	7.94
19	3	1.59
20	32	16.93
23	2	1.06
24	2	1.06
25	9	4.76
26	3	1.59
27	2	1.06
28	3	1.59
29	1	0.53
32	3	1.59
33	5	2.65
34	1	0.53
Total	189	100.00

- **a018_3_7_ : Province**

	No	%
03	3	3.23
05	1	1.08
08	3	3.23
09	5	5.38
11	9	9.68
12	1	1.08
15	32	34.41
17	2	2.15
18	4	4.30
19	1	1.08
20	17	18.28
24	1	1.08
25	6	6.45
27	3	3.23
28	1	1.08
32	1	1.08
33	3	3.23
Total	93	100.00

- **a018_3_8_ : Province**

	No	%
03	1	2.17
06	2	4.35

08	2	4.35
11	6	13.04
15	13	28.26
16	1	2.17
17	1	2.17
18	1	2.17
19	1	2.17
20	14	30.43
25	1	2.17
26	1	2.17
27	1	2.17
28	1	2.17
Total	46	100.00

- **a018_3_9_ : Province**

	No	%
03	3	10.34
09	1	3.45
11	4	13.79
15	7	24.14
18	1	3.45
19	2	6.90
20	8	27.59
24	1	3.45
25	1	3.45
28	1	3.45
Total	29	100.00

- **a018_3_10_ : Province**

	No	%
09	1	10.00
11	1	10.00
15	6	60.00
20	1	10.00
34	1	10.00
Total	10	100.00

- **a018_3_11_ : Province**

	No	%
06	1	16.67
07	1	16.67
11	1	16.67
15	2	33.33
20	1	16.67

Total	6	100.00

- **a018_3_12_ : Province**

	No	%
11	1	50.00
15	1	50.00
Total	2	100.00

- **a018_3_13_ : Province**

	No	%
15	1	50.00
20	1	50.00
Total	2	100.00

- **a018_3_14_ : Province**

	No	%
15	2	100.00
Total	2	100.00

- **a018_3_15_ : Province**

	No	%
15	1	100.00
Total	1	100.00

- **a018_4_ : Main Place**

	No	%
1 This County/City	263	21.33
2 Another County/City in This Province	415	33.66
3 Another province	547	44.36
4 Abroad	8	0.65
Total	1,233	100.00

- **a018_4_1_ : City**

	No	%
01	1	3.03

11	2	6.06
18	1	3.03
24	7	21.21
27	1	3.03
35	1	3.03
38	1	3.03
40	14	42.42
66	1	3.03
67	1	3.03
74	1	3.03
82	1	3.03
96	1	3.03
Total	33	100.00

- **a018_4_2_ : City**

	No	%
01	7	5.98
04	4	3.42
05	1	0.85
11	10	8.55
16	2	1.71
17	1	0.85
24	12	10.26
25	1	0.85
27	2	1.71
28	2	1.71
40	54	46.15
46	1	0.85
51	1	0.85
52	1	0.85
53	1	0.85
55	2	1.71
56	1	0.85
60	1	0.85
66	1	0.85
74	3	2.56
82	2	1.71
84	4	3.42
96	1	0.85
99	2	1.71
Total	117	100.00

- **a018_4_3_ : City**

	No	%
01	43	6.47
02	1	0.15
04	23	3.46

05	2	0.30
11	74	11.13
16	3	0.45
17	1	0.15
18	1	0.15
24	62	9.32
27	4	0.60
35	1	0.15
40	313	47.07
46	8	1.20
49	4	0.60
51	1	0.15
53	25	3.76
55	8	1.20
56	5	0.75
60	9	1.35
63	1	0.15
66	6	0.90
67	1	0.15
74	15	2.26
77	1	0.15
82	16	2.41
83	1	0.15
84	30	4.51
86	3	0.45
96	2	0.30
99	1	0.15
Total	665	100.00

- **a018_4_4_ : City**

	No	%
01	44	9.26
02	1	0.21
04	9	1.89
05	3	0.63
11	54	11.37
24	38	8.00
25	2	0.42
27	5	1.05
28	2	0.42
35	3	0.63
38	1	0.21
40	222	46.74
46	1	0.21
49	2	0.42
51	1	0.21
52	1	0.21
53	15	3.16
55	2	0.42
56	2	0.42

60	9	1.89
63	1	0.21
66	3	0.63
74	11	2.32
82	17	3.58
83	2	0.42
84	19	4.00
86	2	0.42
96	1	0.21
99	2	0.42
Total	475	100.00

- **a018_4_5_ : City**

	No	%
01	31	10.88
04	7	2.46
05	1	0.35
11	38	13.33
24	24	8.42
25	1	0.35
27	2	0.70
28	2	0.70
38	1	0.35
40	123	43.16
46	3	1.05
53	6	2.11
55	3	1.05
56	2	0.70
60	9	3.16
63	1	0.35
66	7	2.46
74	4	1.40
82	7	2.46
83	1	0.35
84	10	3.51
86	2	0.70
Total	285	100.00

- **a018_4_6_ : City**

	No	%
01	12	7.36
02	1	0.61
04	1	0.61
05	1	0.61
11	30	18.40
16	1	0.61
24	9	5.52

25	1	0.61
27	1	0.61
38	1	0.61
40	71	43.56
49	3	1.84
51	1	0.61
53	4	2.45
60	7	4.29
63	1	0.61
66	3	1.84
74	1	0.61
76	1	0.61
82	3	1.84
84	9	5.52
99	1	0.61
Total	163	100.00

- **a018_4_7_ : City**

	No	%
01	4	5.13
02	1	1.28
04	1	1.28
05	1	1.28
11	12	15.38
24	5	6.41
38	1	1.28
40	34	43.59
46	1	1.28
49	2	2.56
53	1	1.28
55	1	1.28
60	2	2.56
66	2	2.56
82	2	2.56
84	7	8.97
96	1	1.28
Total	78	100.00

- **a018_4_8_ : City**

	No	%
01	2	4.65
02	1	2.33
04	3	6.98
11	6	13.95
24	5	11.63
27	2	4.65
40	18	41.86

46	1	2.33
51	1	2.33
66	1	2.33
74	1	2.33
82	1	2.33
84	1	2.33
Total	43	100.00

- **a018_4_9_ : City**

	No	%
01	1	3.85
04	3	11.54
11	7	26.92
24	3	11.54
40	7	26.92
46	1	3.85
53	1	3.85
82	1	3.85
84	2	7.69
Total	26	100.00

- **a018_4_10_ : City**

	No	%
04	1	10.00
11	3	30.00
24	1	10.00
40	2	20.00
53	1	10.00
66	1	10.00
84	1	10.00
Total	10	100.00

- **a018_4_11_ : City**

	No	%
01	1	16.67
04	2	33.33
11	1	16.67
24	1	16.67
66	1	16.67
Total	6	100.00

- **a018_4_12_ : City**

	No	%
04	1	50.00
11	1	50.00
Total	2	100.00

- **a018_4_13_ : City**

	No	%
11	1	50.00
24	1	50.00
Total	2	100.00

- **a018_4_14_ : City**

	No	%
11	1	50.00
40	1	50.00
Total	2	100.00

- **a018_4_15_ : City**

	No	%
11	1	100.00
Total	1	100.00

- **a018_5_ : Main Place**

	No	%
1 This County/City	145	20.89
2 Another County/City in This Province	213	30.69
3 Another province	333	47.98
4 Abroad	3	0.43
Total	694	100.00

- **a018_5_1_ : County**

	No	%
06	1	3.70
08	1	3.70
31	1	3.70
38	1	3.70

45	1	3.70
49	1	3.70
59	3	11.11
63	13	48.15
75	1	3.70
78	1	3.70
81	2	7.41
99	1	3.70
Total	27	100.00

- **a018_5_2_ : County**

	No	%
02	1	1.16
06	2	2.33
08	1	1.16
10	1	1.16
13	2	2.33
16	1	1.16
28	2	2.33
31	6	6.98
37	2	2.33
43	1	1.16
44	1	1.16
45	1	1.16
46	1	1.16
54	3	3.49
56	3	3.49
58	2	2.33
59	1	1.16
63	46	53.49
76	2	2.33
78	5	5.81
99	2	2.33
Total	86	100.00

- **a018_5_3_ : County**

	No	%
02	4	0.83
04	10	2.07
06	8	1.66
08	13	2.69
10	1	0.21
13	2	0.41
16	7	1.45
23	3	0.62
28	4	0.83
31	15	3.11

37	6	1.24
39	15	3.11
43	3	0.62
44	6	1.24
45	2	0.41
46	2	0.41
51	1	0.21
54	23	4.76
56	16	3.31
57	5	1.04
58	4	0.83
59	8	1.66
63	274	56.73
73	5	1.04
75	4	0.83
76	6	1.24
78	14	2.90
81	1	0.21
90	1	0.21
92	10	2.07
97	3	0.62
99	7	1.45
Total	483	100.00

- **a018_5_4_ : County**

	No	%
02	5	1.47
03	2	0.59
04	4	1.18
06	5	1.47
08	7	2.06
16	3	0.88
28	7	2.06
31	15	4.42
37	3	0.88
38	1	0.29
39	10	2.95
43	2	0.59
44	6	1.77
46	1	0.29
51	1	0.29
54	9	2.65
56	9	2.65
57	8	2.36
58	2	0.59
59	5	1.47
63	199	58.70
73	4	1.18
75	1	0.29
76	7	2.06

78	7	2.06
83	1	0.29
92	8	2.36
97	1	0.29
99	6	1.77
Total	339	100.00

- **a018_5_5_ : County**

	No	%
02	3	1.44
04	3	1.44
06	6	2.88
08	3	1.44
16	2	0.96
28	1	0.48
31	1	0.48
38	1	0.48
39	5	2.40
43	1	0.48
44	3	1.44
46	1	0.48
54	11	5.29
56	4	1.92
57	4	1.92
58	1	0.48
59	2	0.96
63	134	64.42
73	1	0.48
75	3	1.44
76	2	0.96
78	5	2.40
92	8	3.85
99	3	1.44
Total	208	100.00

- **a018_5_6_ : County**

	No	%
02	1	0.88
04	1	0.88
06	6	5.31
08	1	0.88
16	2	1.77
23	1	0.88
28	3	2.65
31	1	0.88
38	1	0.88
54	6	5.31

56	2	1.77
58	1	0.88
59	2	1.77
63	75	66.37
73	2	1.77
75	2	1.77
76	1	0.88
78	2	1.77
92	3	2.65
Total	113	100.00

- **a018_5_7_ : County**

	No	%
02	1	1.79
16	1	1.79
23	1	1.79
31	2	3.57
38	2	3.57
39	1	1.79
46	1	1.79
54	5	8.93
56	2	3.57
57	1	1.79
58	3	5.36
63	31	55.36
76	1	1.79
92	3	5.36
99	1	1.79
Total	56	100.00

- **a018_5_8_ : County**

	No	%
02	1	2.94
16	1	2.94
37	1	2.94
38	3	8.82
44	1	2.94
54	3	8.82
58	2	5.88
63	19	55.88
73	1	2.94
78	1	2.94
92	1	2.94
Total	34	100.00

- **a018_5_9_ : County**

	No	%
02	1	5.88
06	1	5.88
28	1	5.88
38	2	11.76
54	3	17.65
63	7	41.18
76	1	5.88
78	1	5.88
Total	17	100.00

- **a018_5_10_ : County**

	No	%
54	1	16.67
56	1	16.67
63	4	66.67
Total	6	100.00

- **a018_5_11_ : County**

	No	%
54	1	33.33
63	2	66.67
Total	3	100.00

- **a018_5_12_ : County**

	No	%
63	1	100.00
Total	1	100.00

- **a018_5_13_ : County**

	No	%
54	1	50.00
63	1	50.00
Total	2	100.00

- **a018_5_14_ : County**

	No	%

63	1	50.00
99	1	50.00
Total	2	100.00

- **a018_5_15_ : County**

	No	%
63	1	100.00
Total	1	100.00

- **a018_6_ : Main Place**

	No	%
1 This County/City	68	18.53
2 Another County/City in This Province	113	30.79
3 Another province	186	50.68
Total	367	100.00

- **a018_7_ : Main Place**

	No	%
1 This County/City	28	16.77
2 Another County/City in This Province	46	27.54
3 Another province	93	55.69
Total	167	100.00

- **a018_8_ : Main Place**

	No	%
1 This County/City	9	12.16
2 Another County/City in This Province	19	25.68
3 Another province	46	62.16
Total	74	100.00

- **a018_9_ : Main Place**

	No	%
1 This County/City	6	13.04
2 Another County/City in This Province	9	19.57
3 Another province	29	63.04
4 Abroad	2	4.35
Total	46	100.00

- **a018_10_ : Main Place**

	No	%
1 This County/City	5	25.00
2 Another County/City in This Province	5	25.00
3 Another province	10	50.00
Total	20	100.00

- **a018_11_ : Main Place**

	No	%
1 This County/City	2	22.22
2 Another County/City in This Province	1	11.11
3 Another province	6	66.67
Total	9	100.00

- **a018_12_ : Main Place**

	No	%
2 Another County/City in This Province	2	50.00
3 Another province	2	50.00
Total	4	100.00

- **a018_13_ : Main Place**

	No	%
3 Another province	2	100.00
Total	2	100.00

- **a018_14_ : Main Place**

	No	%
3 Another province	2	100.00
Total	2	100.00

- **a018_15_ : Main Place**

	No	%
3 Another province	1	100.00
Total	1	100.00

- **a019_1_ : Type of Location**

	No	%
1 City	39	44.32
2 County	25	28.41
3 Town	11	12.50
4 Village	13	14.77
Total	88	100.00

- **a019_2_ : Type of Location**

	No	%
1 City	196	57.14
2 County	73	21.28
3 Town	43	12.54
4 Village	31	9.04
Total	343	100.00

- **a019_3_ : Type of Location**

	No	%
1 City	1,178	66.67
2 County	324	18.34
3 Town	160	9.05
4 Village	105	5.94
Total	1,767	100.00

- **a019_4_ : Type of Location**

	No	%
1 City	832	67.81
2 County	226	18.42
3 Town	94	7.66
4 Village	75	6.11
Total	1,227	100.00

- **a019_5_ : Type of Location**

	No	%
1 City	465	67.78
2 County	117	17.06
3 Town	72	10.50
4 Village	32	4.66
Total	686	100.00

- **a019_6_ : Type of Location**

	No	%
1 City	240	65.40
2 County	60	16.35
3 Town	45	12.26
4 Village	22	5.99
Total	367	100.00

- **a019_7_ : Type of Location**

	No	%
1 City	112	68.71
2 County	25	15.34
3 Town	17	10.43
4 Village	9	5.52
Total	163	100.00

- **a019_8_ : Type of Location**

	No	%
1 City	49	64.47
2 County	13	17.11
3 Town	12	15.79
4 Village	2	2.63
Total	76	100.00

- **a019_9_ : Type of Location**

	No	%
1 City	28	60.87
2 County	10	21.74
3 Town	6	13.04
4 Village	2	4.35
Total	46	100.00

- **a019_10_ : Type of Location**

	No	%
1 City	14	70.00
2 County	2	10.00
3 Town	3	15.00
4 Village	1	5.00
Total	20	100.00

- **a019_11_ : Type of Location**

	No	%
1 City	5	55.56
3 Town	3	33.33
4 Village	1	11.11
Total	9	100.00

- **a019_12_ : Type of Location**

	No	%
1 City	4	100.00
Total	4	100.00

- **a019_13_ : Type of Location**

	No	%
1 City	1	50.00
3 Town	1	50.00
Total	2	100.00

- **a019_14_ : Type of Location**

	No	%
1 City	2	100.00
Total	2	100.00

- **a019_15_ : Type of Location**

	No	%
1 City	1	100.00
Total	1	100.00

- **a021_1_ : Birth Place**

	No	%
1 This Village	392	80.82
2 Another Village in This County/City	65	13.40
3 Another County/City in This Province	15	3.09
4 Another Province	13	2.68
Total	485	100.00

- **a021_1_1_ : City**

	No	%
11	1	6.67
27	2	13.33
40	5	33.33
46	2	13.33
50	1	6.67
53	1	6.67
60	1	6.67
77	1	6.67
82	1	6.67
Total	15	100.00

- **a021_1_2_ : City**

	No	%
04	1	4.35
05	1	4.35
11	1	4.35
24	2	8.70
27	3	13.04
40	4	17.39
46	1	4.35
51	1	4.35
53	2	8.70
55	1	4.35
63	1	4.35
74	1	4.35
82	3	13.04
99	1	4.35
Total	23	100.00

- **a021_1_3_ : City**

	No	%
01	2	3.45
04	3	5.17
05	1	1.72
07	1	1.72
11	3	5.17
16	2	3.45
17	1	1.72
24	1	1.72
27	1	1.72
35	1	1.72
40	14	24.14
46	7	12.07

49	1	1.72
53	3	5.17
55	1	1.72
60	2	3.45
63	2	3.45
74	2	3.45
82	5	8.62
86	3	5.17
96	1	1.72
99	1	1.72
Total	58	100.00

- **a021_1_4_ : City**

	No	%
01	2	13.33
05	2	13.33
24	1	6.67
40	3	20.00
46	1	6.67
53	2	13.33
55	1	6.67
74	1	6.67
82	1	6.67
86	1	6.67
Total	15	100.00

- **a021_1_5_ : City**

	No	%
01	3	27.27
05	1	9.09
11	1	9.09
24	3	27.27
27	2	18.18
46	1	9.09
Total	11	100.00

- **a021_1_6_ : City**

	No	%
11	1	33.33
16	1	33.33
82	1	33.33
Total	3	100.00

- **a021_1_7_ : City**

	No	%
86	1	100.00
Total	1	100.00

- **a021_1_9_ : City**

	No	%
46	1	100.00
Total	1	100.00

- **a021_2_ : Birth Place**

	No	%
1 This Village	811	83.09
2 Another Village in This County/City	122	12.50
3 Another County/City in This Province	23	2.36
4 Another Province	20	2.05
Total	976	100.00

- **a021_2_1_ : County**

	No	%
02	1	7.14
04	2	14.29
06	1	7.14
16	1	7.14
31	1	7.14
54	1	7.14
59	3	21.43
63	4	28.57
Total	14	100.00

- **a021_2_2_ : County**

	No	%
02	2	9.52
03	1	4.76
04	1	4.76
06	1	4.76
08	1	4.76
31	4	19.05
39	2	9.52

45	1	4.76
50	1	4.76
63	5	23.81
76	2	9.52
Total	21	100.00

- **a021_2_3_ : County**

	No	%
02	6	10.71
03	1	1.79
04	3	5.36
16	3	5.36
19	1	1.79
28	2	3.57
31	5	8.93
39	2	3.57
43	5	8.93
45	1	1.79
46	5	8.93
54	2	3.57
57	1	1.79
59	2	3.57
63	6	10.71
76	7	12.50
81	3	5.36
99	1	1.79
Total	56	100.00

- **a021_2_4_ : County**

	No	%
02	2	13.33
16	1	6.67
31	1	6.67
43	1	6.67
46	1	6.67
54	1	6.67
63	6	40.00
76	1	6.67
91	1	6.67
Total	15	100.00

- **a021_2_5_ : County**

	No	%
31	2	22.22

43	1	11.11
54	1	11.11
59	1	11.11
63	4	44.44
Total	9	100.00

- **a021_2_6_ : County**

	No	%
43	1	33.33
76	2	66.67
Total	3	100.00

- **a021_2_7_ : County**

	No	%
46	1	100.00
Total	1	100.00

- **a021_2_9_ : County**

	No	%
06	1	100.00
Total	1	100.00

- **a021_3_ : Birth Place**

	No	%
1 This Village	3,274	86.29
2 Another Village in This County/City	412	10.86
3 Another County/City in This Province	57	1.50
4 Another Province	51	1.34
Total	3,794	100.00

- **a021_3_1_ : Province**

	No	%
05	2	15.38
06	1	7.69
07	1	7.69
11	1	7.69
12	2	15.38
16	1	7.69

26	1	7.69
27	2	15.38
32	1	7.69
34	1	7.69
Total	13	100.00

- **a021_3_2_ : Province**

	No	%
03	1	5.00
05	1	5.00
07	1	5.00
08	1	5.00
13	1	5.00
14	3	15.00
16	2	10.00
19	1	5.00
21	2	10.00
24	1	5.00
25	1	5.00
27	1	5.00
28	1	5.00
29	1	5.00
32	1	5.00
33	1	5.00
Total	20	100.00

- **a021_3_3_ : Province**

	No	%
05	4	7.69
06	1	1.92
07	2	3.85
08	2	3.85
09	1	1.92
10	4	7.69
12	1	1.92
13	1	1.92
14	7	13.46
15	4	7.69
17	3	5.77
20	1	1.92
21	4	7.69
24	1	1.92
26	3	5.77
27	1	1.92
29	3	5.77
32	3	5.77
33	5	9.62

34	1	1.92
Total	52	100.00

- **a021_3_4_ : Province**

	No	%
05	1	5.00
07	1	5.00
08	1	5.00
10	1	5.00
11	1	5.00
12	2	10.00
13	1	5.00
15	2	10.00
17	2	10.00
24	1	5.00
26	2	10.00
27	2	10.00
32	1	5.00
33	1	5.00
34	1	5.00
Total	20	100.00

- **a021_3_5_ : Province**

	No	%
05	2	25.00
06	1	12.50
10	1	12.50
15	1	12.50
21	1	12.50
27	1	12.50
34	1	12.50
Total	8	100.00

- **a021_3_6_ : Province**

	No	%
05	1	33.33
10	1	33.33
15	1	33.33
Total	3	100.00

- **a021_3_7_ : Province**

	No	%
27	1	100.00
Total	1	100.00

- **a021_4_ : Birth Place**

	No	%
1 This Village	1,345	89.85
2 Another Village in This County/City	118	7.88
3 Another County/City in This Province	15	1.00
4 Another Province	19	1.27
Total	1,497	100.00

- **a021_4_1_ : City**

	No	%
04	1	7.69
24	2	15.38
40	1	7.69
46	1	7.69
49	1	7.69
55	1	7.69
63	1	7.69
74	1	7.69
77	1	7.69
82	1	7.69
83	1	7.69
99	1	7.69
Total	13	100.00

- **a021_4_2_ : City**

	No	%
01	3	15.79
04	1	5.26
05	1	5.26
24	2	10.53
25	1	5.26
27	1	5.26
40	1	5.26
53	1	5.26
55	1	5.26
60	1	5.26
63	1	5.26
74	2	10.53
82	3	15.79

Total	19	100.00

- **a021_4_3_ : City**

	No	%
01	1	2.13
04	2	4.26
11	2	4.26
18	1	2.13
24	4	8.51
27	2	4.26
28	1	2.13
38	1	2.13
40	8	17.02
49	5	10.64
52	1	2.13
53	3	6.38
55	3	6.38
56	1	2.13
60	2	4.26
63	1	2.13
74	3	6.38
82	3	6.38
86	1	2.13
99	2	4.26
Total	47	100.00

- **a021_4_4_ : City**

	No	%
01	2	11.11
02	1	5.56
05	1	5.56
11	1	5.56
24	2	11.11
46	1	5.56
49	1	5.56
52	1	5.56
53	2	11.11
55	2	11.11
60	1	5.56
74	1	5.56
82	2	11.11
Total	18	100.00

- **a021_4_5_ : City**

	No	%
01	1	12.50
05	1	12.50
11	1	12.50
16	1	12.50
40	1	12.50
46	1	12.50
52	1	12.50
82	1	12.50
Total	8	100.00

- **a021_4_6_ : City**

	No	%
11	1	33.33
40	1	33.33
82	1	33.33
Total	3	100.00

- **a021_4_7_ : City**

	No	%
82	1	100.00
Total	1	100.00

- **a021_5_ : Birth Place**

	No	%
1 This Village	615	90.18
2 Another Village in This County/City	48	7.04
3 Another County/City in This Province	11	1.61
4 Another Province	8	1.17
Total	682	100.00

- **a021_5_1_ : County**

	No	%
02	1	7.69
31	2	15.38
33	1	7.69
49	1	7.69
63	6	46.15
76	2	15.38
Total	13	100.00

- **a021_5_2_ : County**

	No	%
02	1	5.26
03	1	5.26
06	2	10.53
28	3	15.79
31	4	21.05
39	1	5.26
46	1	5.26
59	3	15.79
63	1	5.26
76	1	5.26
81	1	5.26
Total	19	100.00

- **a021_5_3_ : County**

	No	%
02	4	10.00
04	1	2.50
06	2	5.00
16	2	5.00
28	2	5.00
31	5	12.50
33	1	2.50
39	1	2.50
43	1	2.50
51	1	2.50
54	1	2.50
59	5	12.50
63	7	17.50
76	2	5.00
78	1	2.50
81	2	5.00
92	1	2.50
99	1	2.50
Total	40	100.00

- **a021_5_4_ : County**

	No	%
02	1	5.88
04	1	5.88
06	2	11.76
16	2	11.76
31	2	11.76
33	1	5.88

49	1	5.88
59	3	17.65
63	3	17.65
76	1	5.88
Total	17	100.00

- **a021_5_5_ : County**

	No	%
16	1	14.29
39	1	14.29
54	1	14.29
59	2	28.57
63	1	14.29
76	1	14.29
Total	7	100.00

- **a021_5_6_ : County**

	No	%
39	1	50.00
59	1	50.00
Total	2	100.00

- **a021_5_7_ : County**

	No	%
16	1	100.00
Total	1	100.00

- **a021_6_ : Birth Place**

	No	%
1 This Village	276	92.00
2 Another Village in This County/City	18	6.00
3 Another County/City in This Province	3	1.00
4 Another Province	3	1.00
Total	300	100.00

- **a021_7_ : Birth Place**

	No	%
1 This Village	117	92.86

2 Another Village in This County/City	7	5.56
3 Another County/City in This Province	1	0.79
4 Another Province	1	0.79
Total	126	100.00

- **a021_8_ : Birth Place**

	No	%
1 This Village	44	97.78
2 Another Village in This County/City	1	2.22
Total	45	100.00

- **a021_9_ : Birth Place**

	No	%
1 This Village	23	92.00
2 Another Village in This County/City	1	4.00
3 Another County/City in This Province	1	4.00
Total	25	100.00

- **a021_10_ : Birth Place**

	No	%
1 This Village	8	100.00
Total	8	100.00

- **a021_11_ : Birth Place**

	No	%
1 This Village	4	80.00
2 Another Village in This County/City	1	20.00
Total	5	100.00

- **a021_12_ : Birth Place**

	No	%
1 This Village	6	100.00
Total	6	100.00

- **a021_13_ : Birth Place**

	No	%
1 This Village	1	100.00
Total	1	100.00

- **a021_14_ : Birth Place**

	No	%
1 This Village	2	100.00
Total	2	100.00

- **a021_15_ : Birth Place**

	No	%
1 This Village	1	100.00
Total	1	100.00

- **a022_1_ : Whether Hukou Location Same as Birth Location**

	No	%
1 Yes	422	86.83
2 No	64	13.17
Total	486	100.00

- **a022_2_ : Whether Hukou Location Same as Birth Location**

	No	%
1 Yes	836	85.74
2 No	139	14.26
Total	975	100.00

- **a022_3_ : Whether Hukou Location Same as Birth Location**

	No	%
1 Yes	3,289	86.51
2 No	513	13.49
Total	3,802	100.00

- **a022_4_ : Whether Hukou Location Same as Birth Location**

	No	%

1 Yes	1,330	88.79
2 No	168	11.21
Total	1,498	100.00

- **a022_5_ : Whether Hukou Location Same as Birth Location**

	No	%
1 Yes	615	90.31
2 No	66	9.69
Total	681	100.00

- **a022_6_ : Whether Hukou Location Same as Birth Location**

	No	%
1 Yes	266	88.67
2 No	34	11.33
Total	300	100.00

- **a022_7_ : Whether Hukou Location Same as Birth Location**

	No	%
1 Yes	114	90.48
2 No	12	9.52
Total	126	100.00

- **a022_8_ : Whether Hukou Location Same as Birth Location**

	No	%
1 Yes	40	88.89
2 No	5	11.11
Total	45	100.00

- **a022_9_ : Whether Hukou Location Same as Birth Location**

	No	%
1 Yes	23	92.00
2 No	2	8.00
Total	25	100.00

- **a022_10_ : Whether Hukou Location Same as Birth Location**

	No	%
1 Yes	8	100.00
Total	8	100.00

- **a022_11_ : Whether Hukou Location Same as Birth Location**

	No	%
1 Yes	5	100.00
Total	5	100.00

- **a022_12_ : Whether Hukou Location Same as Birth Location**

	No	%
1 Yes	6	85.71
2 No	1	14.29
Total	7	100.00

- **a022_13_ : Whether Hukou Location Same as Birth Location**

	No	%
1 Yes	1	100.00
Total	1	100.00

- **a022_14_ : Whether Hukou Location Same as Birth Location**

	No	%
1 Yes	2	100.00
Total	2	100.00

- **a022_15_ : Whether Hukou Location Same as Birth Location**

	No	%
1 Yes	1	100.00
Total	1	100.00

- **a023_1_ : The Change of Hukou Status and Location**

	No	%
1 Both Hukou status and location have changed	34	47.89
2 Only Hukou status has changed	8	11.27

3 Only Hukou location has changed	29	40.85
Total	71	100.00

- **a023_2_ : The Change of Hukou Status and Location**

	No	%
1 Both Hukou status and location have changed	55	36.67
2 Only Hukou status has changed	23	15.33
3 Only Hukou location has changed	72	48.00
Total	150	100.00

- **a023_3_ : The Change of Hukou Status and Location**

	No	%
1 Both Hukou status and location have changed	254	46.27
2 Only Hukou status has changed	68	12.39
3 Only Hukou location has changed	227	41.35
Total	549	100.00

- **a023_4_ : The Change of Hukou Status and Location**

	No	%
1 Both Hukou status and location have changed	91	52.00
2 Only Hukou status has changed	20	11.43
3 Only Hukou location has changed	64	36.57
Total	175	100.00

- **a023_5_ : The Change of Hukou Status and Location**

	No	%
1 Both Hukou status and location have changed	38	54.29
2 Only Hukou status has changed	5	7.14
3 Only Hukou location has changed	27	38.57
Total	70	100.00

- **a023_6_ : The Change of Hukou Status and Location**

	No	%
1 Both Hukou status and location have changed	18	48.65
2 Only Hukou status has changed	5	13.51
3 Only Hukou location has changed	14	37.84
Total	37	100.00

- **a023_7_ : The Change of Hukou Status and Location**

	No	%
1 Both Hukou status and location have changed	8	66.67
3 Only Hukou location has changed	4	33.33
Total	12	100.00

- **a023_8_ : The Change of Hukou Status and Location**

	No	%
1 Both Hukou status and location have changed	4	66.67
2 Only Hukou status has changed	2	33.33
Total	6	100.00

- **a023_9_ : The Change of Hukou Status and Location**

	No	%
1 Both Hukou status and location have changed	1	33.33
2 Only Hukou status has changed	1	33.33
3 Only Hukou location has changed	1	33.33
Total	3	100.00

- **a023_12_ : The Change of Hukou Status and Location**

	No	%
3 Only Hukou location has changed	1	100.00
Total	1	100.00

- **a024_1_ : First Hukou Status**

	No	%
1 Agricultural Hukou	34	91.89
2 Non-agricultual Hukou	3	8.11
Total	37	100.00

- **a024_2_ : First Hukou Status**

	No	%
1 Agricultural Hukou	71	100.00
Total	71	100.00

- **a024_3_ : First Hukou Status**

	No	%
1 Agricultural Hukou	290	96.35
2 Non-agricultual Hukou	11	3.65
Total	301	100.00

- **a024_4_ : First Hukou Status**

	No	%
1 Agricultural Hukou	102	97.14
2 Non-agricultual Hukou	3	2.86
Total	105	100.00

- **a024_5_ : First Hukou Status**

	No	%
1 Agricultural Hukou	40	95.24
2 Non-agricultual Hukou	2	4.76
Total	42	100.00

- **a024_6_ : First Hukou Status**

	No	%
1 Agricultural Hukou	19	95.00
2 Non-agricultual Hukou	1	5.00
Total	20	100.00

- **a024_7_ : First Hukou Status**

	No	%
1 Agricultural Hukou	8	100.00
Total	8	100.00

- **a024_8_ : First Hukou Status**

	No	%
1 Agricultural Hukou	5	100.00
Total	5	100.00

- **a024_9_ : First Hukou Status**

	No	%
1 Agricultural Hukou	1	100.00
Total	1	100.00

- **a025_1_ : First Hukou Location**

	No	%
1 This Village	10	16.39
2 Another Village in This County/City	33	54.10
3 Another County/City in This Province	11	18.03
4 Another Province	7	11.48
Total	61	100.00

- **a025_1_1_ : City**

	No	%
11	1	9.09
27	1	9.09
40	5	45.45
46	1	9.09
60	1	9.09
82	1	9.09
86	1	9.09
Total	11	100.00

- **a025_1_2_ : City**

	No	%
05	1	6.67
24	1	6.67
27	2	13.33
40	3	20.00
46	1	6.67
53	1	6.67
63	1	6.67
74	1	6.67
82	3	20.00
99	1	6.67
Total	15	100.00

- **a025_1_3_ : City**

	No	%

01	2	5.71
04	1	2.86
05	1	2.86
11	2	5.71
16	2	5.71
28	1	2.86
35	1	2.86
40	9	25.71
46	4	11.43
53	1	2.86
60	2	5.71
63	1	2.86
74	1	2.86
82	3	8.57
86	3	8.57
96	1	2.86
Total	35	100.00

- **a025_1_4_ : City**

	No	%
05	1	11.11
24	1	11.11
40	3	33.33
46	1	11.11
74	1	11.11
82	1	11.11
86	1	11.11
Total	9	100.00

- **a025_1_5_ : City**

	No	%
01	2	33.33
05	1	16.67
24	1	16.67
27	2	33.33
Total	6	100.00

- **a025_1_6_ : City**

	No	%
16	1	50.00
82	1	50.00
Total	2	100.00

- **a025_1_7_ : City**

	No	%
86	1	100.00
Total	1	100.00

- **a025_2_ : First Hukou Location**

	No	%
1 This Village	38	31.15
2 Another Village in This County/City	61	50.00
3 Another County/City in This Province	14	11.48
4 Another Province	9	7.38
Total	122	100.00

- **a025_2_1_ : County**

	No	%
04	1	10.00
16	1	10.00
31	1	10.00
54	1	10.00
59	2	20.00
63	4	40.00
Total	10	100.00

- **a025_2_2_ : County**

	No	%
03	1	7.14
04	1	7.14
06	1	7.14
08	1	7.14
31	2	14.29
39	2	14.29
45	1	7.14
63	3	21.43
76	2	14.29
Total	14	100.00

- **a025_2_3_ : County**

	No	%
02	5	14.71

03	1	2.94
16	1	2.94
19	1	2.94
28	1	2.94
31	4	11.76
43	3	8.82
44	1	2.94
46	4	11.76
59	1	2.94
63	4	11.76
76	4	11.76
81	3	8.82
99	1	2.94
Total	34	100.00

- **a025_2_4_ : County**

	No	%
02	2	22.22
46	1	11.11
59	1	11.11
63	3	33.33
75	1	11.11
76	1	11.11
Total	9	100.00

- **a025_2_5_ : County**

	No	%
31	1	20.00
59	2	40.00
63	2	40.00
Total	5	100.00

- **a025_2_6_ : County**

	No	%
63	1	50.00
76	1	50.00
Total	2	100.00

- **a025_2_7_ : County**

	No	%
46	1	100.00

Total	1	100.00

- **a025_3_ : First Hukou Location**

	No	%
1 This Village	238	51.07
2 Another Village in This County/City	169	36.27
3 Another County/City in This Province	36	7.73
4 Another Province	23	4.94
Total	466	100.00

- **a025_3_1_ : Province**

	No	%
05	1	14.29
06	1	14.29
11	1	14.29
12	1	14.29
16	1	14.29
27	1	14.29
32	1	14.29
Total	7	100.00

- **a025_3_2_ : Province**

	No	%
07	1	11.11
08	1	11.11
14	1	11.11
16	1	11.11
21	2	22.22
25	1	11.11
27	1	11.11
29	1	11.11
Total	9	100.00

- **a025_3_3_ : Province**

	No	%
05	3	12.50
06	1	4.17
08	2	8.33
10	1	4.17
12	1	4.17
13	1	4.17

14	2	8.33
15	1	4.17
17	2	8.33
20	1	4.17
21	3	12.50
26	1	4.17
27	1	4.17
29	2	8.33
33	2	8.33
Total	24	100.00

- **a025_3_4_ : Province**

	No	%
10	1	16.67
11	1	16.67
13	1	16.67
17	1	16.67
26	1	16.67
27	1	16.67
Total	6	100.00

- **a025_3_5_ : Province**

	No	%
05	2	50.00
06	1	25.00
27	1	25.00
Total	4	100.00

- **a025_3_6_ : Province**

	No	%
05	1	25.00
06	1	25.00
10	1	25.00
11	1	25.00
Total	4	100.00

- **a025_3_7_ : Province**

	No	%
27	1	100.00
Total	1	100.00

- **a025_4_ : First Hukou Location**

	No	%
1 This Village	89	58.55
2 Another Village in This County/City	49	32.24
3 Another County/City in This Province	8	5.26
4 Another Province	6	3.95
Total	152	100.00

- **a025_4_1_ : City**

	No	%
04	1	14.29
24	1	14.29
40	1	14.29
55	1	14.29
74	1	14.29
82	1	14.29
83	1	14.29
Total	7	100.00

- **a025_4_2_ : City**

	No	%
01	1	11.11
05	1	11.11
24	1	11.11
25	1	11.11
55	1	11.11
74	2	22.22
82	2	22.22
Total	9	100.00

- **a025_4_3_ : City**

	No	%
04	2	9.52
18	1	4.76
24	3	14.29
28	1	4.76
40	5	23.81
49	1	4.76
53	1	4.76
56	1	4.76
74	3	14.29
82	1	4.76

86	1	4.76
99	1	4.76
Total	21	100.00

- **a025_4_4_ : City**

	No	%
11	1	16.67
53	1	16.67
55	1	16.67
74	1	16.67
82	2	33.33
Total	6	100.00

- **a025_4_5_ : City**

	No	%
05	1	25.00
11	1	25.00
16	1	25.00
24	1	25.00
Total	4	100.00

- **a025_4_6_ : City**

	No	%
11	1	25.00
40	1	25.00
46	1	25.00
82	1	25.00
Total	4	100.00

- **a025_4_7_ : City**

	No	%
82	1	100.00
Total	1	100.00

- **a025_5_ : First Hukou Location**

	No	%
1 This Village	37	60.66
2 Another Village in This County/City	14	22.95

3 Another County/City in This Province	6	9.84
4 Another Province	4	6.56
Total	61	100.00

- **a025_5_1_ : County**

	No	%
02	1	14.29
31	2	28.57
63	4	57.14
Total	7	100.00

- **a025_5_2_ : County**

	No	%
06	1	11.11
28	3	33.33
39	1	11.11
59	1	11.11
63	1	11.11
76	1	11.11
81	1	11.11
Total	9	100.00

- **a025_5_3_ : County**

	No	%
02	4	23.53
04	1	5.88
16	2	11.76
31	1	5.88
33	1	5.88
39	1	5.88
54	1	5.88
59	1	5.88
63	1	5.88
76	1	5.88
81	2	11.76
92	1	5.88
Total	17	100.00

- **a025_5_4_ : County**

	No	%
16	1	16.67

33	1	16.67
44	1	16.67
59	2	33.33
63	1	16.67
Total	6	100.00

- **a025_5_5_ : County**

	No	%
03	1	33.33
16	1	33.33
59	1	33.33
Total	3	100.00

- **a025_5_6_ : County**

	No	%
54	1	33.33
59	1	33.33
63	1	33.33
Total	3	100.00

- **a025_5_7_ : County**

	No	%
16	1	100.00
Total	1	100.00

- **a025_6_ : First Hukou Location**

	No	%
1 This Village	22	68.75
2 Another Village in This County/City	5	15.63
3 Another County/City in This Province	2	6.25
4 Another Province	3	9.38
Total	32	100.00

- **a025_7_ : First Hukou Location**

	No	%
1 This Village	9	75.00
2 Another Village in This County/City	1	8.33
3 Another County/City in This Province	1	8.33

4 Another Province	1	8.33
Total	12	100.00

- **a025_8_ : First Hukou Location**

	No	%
1 This Village	4	100.00
Total	4	100.00

- **a025_9_ : First Hukou Location**

	No	%
1 This Village	1	50.00
2 Another Village in This County/City	1	50.00
Total	2	100.00

- **a025_12_ : First Hukou Location**

	No	%
1 This Village	1	100.00
Total	1	100.00

- **proxy : Interview Done by Proxy**

	No	%
0 No	9,970	97.50
1 Yes	256	2.50
Total	10,226	100.00

2 Demographic Background

- **ID : Individual ID**

A String Variable	
OBS:	17,705

- **householdID : Household ID**

A String Variable	
OBS:	17,705

- **communityID : Community ID**

A String Variable	
OBS:	17,705

- **ba001 : Chinese Zodiac**

	No	%
1 Rat	1,270	7.20
2 Ox	1,341	7.60
3 Tiger	1,691	9.59
4 Rabbit	1,841	10.44
5 Dragon	1,757	9.96
6 Snake	1,843	10.45
7 Horse	1,590	9.01
8 Goat	1,310	7.43
9 Monkey	1,254	7.11
10 Rooster	1,312	7.44
11 Dog	1,205	6.83
12 Pig	1,225	6.94
Total	17,639	100.00

- **ba002_1 : Birth Year**

Mean	Min	Max	OBS
1,951.9	1,910.0	1,989.0	17,651

- **ba002_2 : Birth Month**

	No	%

0	481	2.73
1	1,394	7.90
2	1,500	8.50
3	1,404	7.95
4	1,236	7.00
5	1,316	7.46
6	1,299	7.36
7	1,423	8.06
8	1,476	8.36
9	1,449	8.21
10	1,635	9.26
11	1,443	8.18
12	1,595	9.04
Total	17,651	100.00

- **ba002_3 : Birth Day**

	No	%
0	928	5.26
1	722	4.09
2	623	3.53
3	498	2.82
4	520	2.95
5	572	3.24
6	639	3.62
7	479	2.71
8	559	3.17
9	546	3.09
10	743	4.21
11	487	2.76
12	643	3.64
13	540	3.06
14	559	3.17
15	778	4.41
16	622	3.52
17	561	3.18
18	586	3.32
19	532	3.01
20	687	3.89
21	454	2.57
22	472	2.67
23	494	2.80
24	496	2.81
25	551	3.12
26	484	2.74
27	506	2.87
28	538	3.05
29	508	2.88
30	293	1.66
31	31	0.18
Total	17,651	100.00

- **ba003 : Calender Type**

	No	%
1 Solar calendar	3,921	22.28
2 Lunar calendar	13,681	77.72
Total	17,602	100.00

- **ba004 : Age**

	No	%
19	1	2.94
24	1	2.94
42	1	2.94
45	2	5.88
46	1	2.94
48	2	5.88
50	1	2.94
55	2	5.88
56	3	8.82
60	4	11.76
62	2	5.88
63	2	5.88
64	3	8.82
65	1	2.94
67	1	2.94
69	2	5.88
70	1	2.94
72	1	2.94
78	1	2.94
81	1	2.94
82	1	2.94
Total	34	100.00

- **ba005 : Return Home Every Week**

	No	%
1 Yes	1,823	10.31
2 No	15,860	89.69
Total	17,683	100.00

- **bb001 : Birth Place**

	No	%
1 This Village/Neighborhood	8,553	48.44

2 Another Village/Neighborhood in this County/City	6,843	38.75
3 Another County/City in this Province	1,241	7.03
4 Another Province	1,017	5.76
5 Abroad	4	0.02
Total	17,658	100.00

- **bb001_1 : City**

	No	%
01	75	6.03
02	12	0.96
04	62	4.98
05	17	1.37
07	3	0.24
11	77	6.19
16	32	2.57
17	8	0.64
18	5	0.40
24	55	4.42
27	56	4.50
28	3	0.24
35	10	0.80
40	180	14.47
42	7	0.56
46	112	9.00
49	25	2.01
50	2	0.16
51	9	0.72
52	5	0.40
53	55	4.42
55	61	4.90
56	10	0.80
60	63	5.06
63	49	3.94
66	5	0.40
74	63	5.06
76	1	0.08
77	8	0.64
82	113	9.08
83	2	0.16
84	1	0.08
86	26	2.09
96	21	1.69
99	11	0.88
Total	1,244	100.00

- **bb001_2 : County**

	No	%

02	83	7.67
03	21	1.94
04	45	4.16
06	101	9.33
07	3	0.28
08	1	0.09
13	3	0.28
16	21	1.94
19	5	0.46
28	35	3.23
31	104	9.61
33	9	0.83
37	7	0.65
38	4	0.37
39	13	1.20
43	62	5.73
44	14	1.29
45	5	0.46
46	73	6.75
49	2	0.18
50	2	0.18
51	33	3.05
54	46	4.25
56	2	0.18
57	7	0.65
58	4	0.37
59	128	11.83
63	53	4.90
73	1	0.09
75	2	0.18
76	108	9.98
78	3	0.28
81	48	4.44
90	10	0.92
91	11	1.02
92	8	0.74
98	1	0.09
99	4	0.37
Total	1,082	100.00

- **bb001_3 : Province**

	No	%
01	16	1.55
03	6	0.58
04	1	0.10
05	69	6.69
06	94	9.12
07	16	1.55
08	4	0.39
09	8	0.78

10	21	2.04
11	56	5.43
12	47	4.56
13	12	1.16
14	74	7.18
15	8	0.78
16	122	11.83
17	21	2.04
18	15	1.45
19	18	1.75
20	19	1.84
21	72	6.98
23	2	0.19
24	29	2.81
25	3	0.29
26	28	2.72
27	20	1.94
28	18	1.75
29	122	11.83
32	58	5.63
33	37	3.59
34	15	1.45
Total	1,031	100.00

- **bb001_4 : City**

	No	%
01	35	3.53
02	1	0.10
04	72	7.26
05	4	0.40
11	76	7.66
16	19	1.92
18	3	0.30
24	144	14.52
27	44	4.44
28	10	1.01
40	153	15.42
42	1	0.10
46	56	5.65
49	35	3.53
51	3	0.30
52	3	0.30
53	32	3.23
55	53	5.34
56	2	0.20
60	42	4.23
63	25	2.52
66	1	0.10
67	1	0.10
74	32	3.23

76	1	0.10
82	77	7.76
86	23	2.32
88	3	0.30
96	4	0.40
99	37	3.73
Total	992	100.00

- **bb001_5 : County**

	No	%
02	48	6.04
03	14	1.76
04	21	2.64
06	95	11.95
07	5	0.63
08	2	0.25
13	3	0.38
16	1	0.13
19	3	0.38
23	7	0.88
28	13	1.64
31	87	10.94
33	11	1.38
37	6	0.75
38	5	0.63
39	61	7.67
43	55	6.92
44	12	1.51
45	4	0.50
46	44	5.53
49	5	0.63
51	14	1.76
54	41	5.16
56	3	0.38
57	9	1.13
58	2	0.25
59	75	9.43
63	20	2.52
73	1	0.13
75	11	1.38
76	52	6.54
78	10	1.26
81	32	4.03
83	3	0.38
90	1	0.13
91	9	1.13
92	7	0.88
99	3	0.38
Total	795	100.00

- **bb002 : Type of Birth Place**

	No	%
1 Rural Village	1,842	81.07
2 Urban Community	430	18.93
Total	2,272	100.00

- **bb003 : Year**

Mean	Min	Max	OBS
1,976.4	1,930.0	2,011.0	2,221

- **bb004 : Whether Live in the Same County/City Where You First Move To**

	No	%
1 Yes	1,589	70.15
2 No	676	29.85
Total	2,265	100.00

- **bb005 : Year**

Mean	Min	Max	OBS
1,980.9	1,900.0	2,011.0	7,341

- **bb006 : Where did You Mainly Live before 16**

	No	%
1 City / Town	15,759	89.18
2 Village	1,912	10.82
Total	17,671	100.00

- **bb007 : Did You Ever Live Outside Your Present County/City for More Than 6 Months**

	No	%
1 Yes	267	11.82
2 No	1,992	88.18
Total	2,259	100.00

- **bb008_1 : Years**

Mean	Min	Max	OBS
77.6	0.0	2,011.0	264

- **bb008_2 : Months**

	No	%
0	202	80.80
1	4	1.60
2	5	2.00
3	1	0.40
4	1	0.40
6	15	6.00
7	4	1.60
8	6	2.40
9	4	1.60
10	3	1.20
11	5	2.00
Total	250	100.00

- **bb009 : Did You Ever Lived Outside this County/City for More than 6 Months**

	No	%
1 Yes	2,115	13.72
2 No	13,305	86.28
Total	15,420	100.00

- **bb010_1 : Years**

Mean	Min	Max	OBS
31.5	0.0	2,010.0	2,165

- **bb010_2 : Months**

Mean	Min	Max	OBS
2.8	0.0	1,987.0	2,006

- **bb011 : Most Recent Year Lived in This County/City**

Mean	Min	Max	OBS

1,989.8	1,920.0	2,011.0	2,355

- **bb012 : Where Did You Live for at Least Six Months before Moving to the Current County/C**

	No	%
1 Birth Place	227	9.49
2 Another County/City in this Province	963	40.26
3 Another Province	1,183	49.46
4 Abroad	19	0.79
Total	2,392	100.00

- **bb012_1 : City**

	No	%
01	54	5.83
02	16	1.73
04	52	5.62
05	2	0.22
07	1	0.11
11	56	6.05
16	7	0.76
17	3	0.32
18	6	0.65
24	96	10.37
27	11	1.19
35	2	0.22
40	244	26.35
42	8	0.86
46	49	5.29
49	27	2.92
51	3	0.32
52	1	0.11
53	50	5.40
55	33	3.56
56	4	0.43
60	25	2.70
63	18	1.94
66	3	0.32
74	42	4.54
76	5	0.54
77	4	0.43
82	67	7.24
83	6	0.65
84	9	0.97
86	17	1.84
89	1	0.11
96	1	0.11
99	3	0.32

Total	926	100.00

- **bb012_2 : County**

	No	%
02	22	2.53
03	5	0.58
04	53	6.11
06	39	4.49
07	1	0.12
08	3	0.35
13	1	0.12
16	24	2.76
19	1	0.12
28	21	2.42
31	84	9.68
33	14	1.61
37	10	1.15
38	1	0.12
39	20	2.30
43	27	3.11
44	6	0.69
45	2	0.23
46	17	1.96
49	2	0.23
50	4	0.46
51	6	0.69
54	40	4.61
56	6	0.69
57	16	1.84
58	6	0.69
59	44	5.07
63	309	35.60
73	1	0.12
75	2	0.23
76	24	2.76
78	13	1.50
81	6	0.69
89	1	0.12
91	2	0.23
92	27	3.11
98	1	0.12
99	7	0.81
Total	868	100.00

- **bb012_3 : Province**

	No	%
01	40	3.39

02	13	1.10
03	42	3.56
04	12	1.02
05	24	2.03
06	43	3.64
07	21	1.78
08	45	3.81
09	95	8.05
10	30	2.54
11	74	6.27
12	18	1.53
13	13	1.10
14	38	3.22
15	188	15.93
16	45	3.81
17	37	3.14
18	61	5.17
19	12	1.02
20	82	6.95
21	22	1.86
23	16	1.36
24	26	2.20
25	18	1.53
26	21	1.78
27	28	2.37
28	35	2.97
29	17	1.44
32	36	3.05
33	14	1.19
34	14	1.19
Total	1,180	100.00

- **bb012_4 : City**

	No	%
01	68	6.19
02	7	0.64
04	40	3.64
05	9	0.82
07	2	0.18
11	99	9.01
16	2	0.18
17	4	0.36
18	5	0.45
24	103	9.37
25	1	0.09
27	15	1.36
28	5	0.45
35	3	0.27
38	5	0.45
40	438	39.85

42	6	0.55
46	17	1.55
49	15	1.36
51	6	0.55
52	2	0.18
53	54	4.91
55	34	3.09
56	2	0.18
60	17	1.55
63	13	1.18
66	4	0.36
67	1	0.09
69	2	0.18
74	36	3.28
76	1	0.09
77	3	0.27
82	33	3.00
83	1	0.09
84	17	1.55
86	12	1.09
88	1	0.09
89	2	0.18
96	11	1.00
99	3	0.27
Total	1,099	100.00

- **bb012_5 : County**

	No	%
02	17	2.50
03	7	1.03
04	21	3.08
06	22	3.23
07	2	0.29
08	21	3.08
10	4	0.59
13	6	0.88
16	14	2.06
23	1	0.15
28	16	2.35
31	55	8.08
33	4	0.59
37	6	0.88
38	7	1.03
39	11	1.62
43	10	1.47
44	4	0.59
45	4	0.59
46	15	2.20
49	1	0.15
50	1	0.15

51	8	1.17
54	33	4.85
56	14	2.06
57	13	1.91
58	6	0.88
59	36	5.29
63	211	30.98
73	4	0.59
75	11	1.62
76	26	3.82
78	21	3.08
81	10	1.47
90	2	0.29
91	3	0.44
92	33	4.85
99	1	0.15
Total	681	100.00

- **bc001 : What is Your Current Hukou Status**

	No	%
1 Agricultual Hukou	13,694	77.49
2 Non-Agricultural Hukou	3,862	21.85
3 Unified Residence Hukou	107	0.61
4 Do not have Hukou	10	0.06
Total	17,673	100.00

- **bc002 : What is Your Hukou Status before You Have the Unified Residence Hukou**

	No	%
1 Agricultual Hukou	92	74.80
2 Non-agricultural Hukou	28	22.76
3 Do not have Hukou	3	2.44
Total	123	100.00

- **bc003_1 : Year**

Mean	Min	Max	OBS
2,005.6	2,000.0	2,011.0	63

- **bc003_2 : Month**

	No	%
0	7	10.00

1	7	10.00
3	1	1.43
5	2	2.86
6	2	2.86
7	5	7.14
8	27	38.57
9	5	7.14
10	6	8.57
11	4	5.71
12	4	5.71
Total	70	100.00

- **bc004_1 : Year**

Mean	Min	Max	OBS
1,966.5	1,910.0	2,011.0	17,543

- **bc004_2 : Month**

	No	%
0	4,402	25.09
1	1,356	7.73
2	1,103	6.29
3	919	5.24
4	848	4.83
5	931	5.31
6	799	4.55
7	953	5.43
8	1,046	5.96
9	1,020	5.81
10	1,467	8.36
11	1,066	6.08
12	1,633	9.31
Total	17,543	100.00

- **bc005 : Where is Your Current Hukou**

	No	%
1 Same as Birthplace	5,417	30.67
2 This Village/Neighborhood	10,892	61.68
3 Another Village/Neighborhood in this County/City	1,136	6.43
4 Another County/City in this Province	150	0.85
5 Another Province	65	0.37
Total	17,660	100.00

- **bc005_1 : City**

	No	%
01	5	3.33
04	12	8.00
07	1	0.67
11	12	8.00
16	4	2.67
17	2	1.33
24	11	7.33
27	6	4.00
40	33	22.00
42	1	0.67
46	15	10.00
49	2	1.33
51	4	2.67
53	10	6.67
55	8	5.33
60	4	2.67
63	4	2.67
74	4	2.67
77	1	0.67
82	7	4.67
86	3	2.00
96	1	0.67
Total	150	100.00

- **bc005_2 : County**

	No	%
02	5	3.52
04	13	9.15
06	5	3.52
08	1	0.70
13	1	0.70
16	7	4.93
28	9	6.34
31	15	10.56
39	4	2.82
43	5	3.52
44	1	0.70
46	6	4.23
50	1	0.70
51	1	0.70
54	4	2.82
56	2	1.41
57	2	1.41
58	2	1.41
59	10	7.04
63	30	21.13

76	4	2.82
78	5	3.52
81	3	2.11
91	1	0.70
92	3	2.11
99	2	1.41
Total	142	100.00

- **bc005_3 : Province**

	No	%
02	1	1.49
03	1	1.49
04	1	1.49
05	3	4.48
06	4	5.97
07	4	5.97
09	1	1.49
10	3	4.48
11	2	2.99
12	4	5.97
13	2	2.99
14	7	10.45
15	1	1.49
16	2	2.99
17	2	2.99
18	2	2.99
19	1	1.49
20	2	2.99
21	6	8.96
26	2	2.99
27	2	2.99
29	4	5.97
32	3	4.48
33	4	5.97
34	3	4.48
Total	67	100.00

- **bc005_4 : City**

	No	%
01	1	1.54
04	5	7.69
11	3	4.62
16	1	1.54
24	6	9.23
27	3	4.62
28	2	3.08
40	16	24.62

46	3	4.62
49	3	4.62
53	8	12.31
55	4	6.15
60	1	1.54
63	1	1.54
82	5	7.69
86	1	1.54
99	2	3.08
Total	65	100.00

- **bc005_5 : County**

	No	%
02	3	5.45
06	5	9.09
08	2	3.64
28	1	1.82
31	9	16.36
38	1	1.82
39	1	1.82
43	2	3.64
44	2	3.64
45	1	1.82
49	1	1.82
51	2	3.64
54	3	5.45
56	1	1.82
59	8	14.55
63	6	10.91
76	3	5.45
81	3	5.45
92	1	1.82
Total	55	100.00

- **bc006_1 : Years**

	No	%
0	38	9.84
1	28	7.25
2	19	4.92
3	21	5.44
4	22	5.70
5	16	4.15
6	17	4.40
7	10	2.59
8	12	3.11
9	12	3.11
10	19	4.92

11	7	1.81
12	10	2.59
13	10	2.59
14	15	3.89
15	9	2.33
16	7	1.81
17	7	1.81
18	8	2.07
19	6	1.55
20	20	5.18
21	3	0.78
22	11	2.85
23	5	1.30
24	6	1.55
25	4	1.04
26	7	1.81
27	5	1.30
28	3	0.78
29	1	0.26
30	1	0.26
31	1	0.26
34	1	0.26
35	1	0.26
36	1	0.26
37	1	0.26
39	1	0.26
40	5	1.30
41	3	0.78
42	1	0.26
44	2	0.52
46	1	0.26
47	1	0.26
49	1	0.26
50	2	0.52
52	1	0.26
56	1	0.26
60	1	0.26
63	1	0.26
80	1	0.26
Total	386	100.00

- **bc006_2 : Months**

	No	%
0	268	69.43
1	14	3.63
2	12	3.11
3	18	4.66
4	15	3.89
5	10	2.59
6	15	3.89

7	9	2.33
8	11	2.85
9	5	1.30
10	6	1.55
11	3	0.78
Total	386	100.00

- **bc007 : Is Your First Hukou Same as Your Current Hukou**

	No	%
1 Yes	9,524	53.97
2 No	8,123	46.03
Total	17,647	100.00

- **bc008 : Any other Hukou between Your First and Current Hukou**

	No	%
1 Yes.	1,256	15.06
2 No.	7,083	84.94
Total	8,339	100.00

- **bc009 : Has Your Hukou Type or Place Ever Changed since Your First Hukou**

	No	%
1 Both Hukou type and place have changed	1,634	19.92
2 Only Hukou type has changed	316	3.85
3 Only Hukou place has changed	6,254	76.23
Total	8,204	100.00

- **bc010 : What Was Your First Hukou Status**

	No	%
1 Agricultural Hukou	1,822	93.77
2 Non-agricultual Hukou	121	6.23
Total	1,943	100.00

- **bc011 : Where was Your Last Hukou**

	No	%
1 Same as Birthplace	584	48.55
2 Another Village/Neighborhood in this County/City	360	29.93
3 Another County/City in this Province	128	10.64

4 Another Province	131	10.89
Total	1,203	100.00

- **bc011_1 : City**

	No	%
01	3	2.40
02	3	2.40
04	6	4.80
11	8	6.40
16	6	4.80
24	10	8.00
27	7	5.60
35	1	0.80
40	25	20.00
46	9	7.20
49	1	0.80
51	1	0.80
53	8	6.40
55	6	4.80
60	10	8.00
63	1	0.80
74	6	4.80
76	1	0.80
82	10	8.00
86	1	0.80
99	2	1.60
Total	125	100.00

- **bc011_2 : County**

	No	%
02	9	7.56
03	1	0.84
04	10	8.40
06	5	4.20
28	7	5.88
31	8	6.72
33	4	3.36
37	1	0.84
39	1	0.84
44	2	1.68
45	1	0.84
46	4	3.36
49	1	0.84
50	1	0.84
54	3	2.52
56	2	1.68
57	1	0.84

58	1	0.84
59	12	10.08
63	33	27.73
75	1	0.84
76	3	2.52
78	1	0.84
81	3	2.52
90	1	0.84
91	2	1.68
92	1	0.84
Total	119	100.00

- **bc011_3 : Province**

	No	%
01	5	3.94
02	3	2.36
03	2	1.57
05	7	5.51
06	14	11.02
07	3	2.36
08	4	3.15
09	6	4.72
10	2	1.57
11	4	3.15
12	3	2.36
13	3	2.36
14	6	4.72
15	5	3.94
16	12	9.45
17	7	5.51
18	1	0.79
19	1	0.79
20	6	4.72
21	6	4.72
24	3	2.36
25	2	1.57
26	3	2.36
27	4	3.15
28	3	2.36
29	3	2.36
32	2	1.57
33	3	2.36
34	4	3.15
Total	127	100.00

- **bc011_4 : City**

	No	%

01	3	2.38
02	2	1.59
04	9	7.14
05	1	0.79
11	4	3.17
17	1	0.79
18	1	0.79
24	14	11.11
27	4	3.17
28	1	0.79
40	34	26.98
42	1	0.79
46	6	4.76
49	3	2.38
51	1	0.79
53	7	5.56
55	7	5.56
56	1	0.79
60	5	3.97
63	2	1.59
74	5	3.97
77	1	0.79
82	7	5.56
86	4	3.17
96	2	1.59
Total	126	100.00

- **bc011_5 : County**

	No	%
02	4	3.64
03	4	3.64
04	1	0.91
06	6	5.45
08	1	0.91
23	1	0.91
28	1	0.91
31	8	7.27
33	4	3.64
39	2	1.82
43	2	1.82
44	1	0.91
45	3	2.73
46	2	1.82
51	2	1.82
54	3	2.73
57	1	0.91
58	2	1.82
59	3	2.73
63	47	42.73
76	4	3.64

78	4	3.64
81	2	1.82
91	1	0.91
92	1	0.91
Total	110	100.00

- **bc012 : Why the Location of Last Hukou is Different from Your Birth Place**

	No	%
1 marriage	195	31.40
2 go to school	25	4.03
3 employment	175	28.18
4 retirement/ revolutionary retirement	5	0.81
5 sent down to the countryside to do manual labor	15	2.42
6 migration	34	5.48
7 migration of the whole village	11	1.77
8 others, pls specify:	161	25.93
Total	621	100.00

- **bc013 : What was the Location of Your First Hukou**

	No	%
1 Same as Birth Place	6,613	84.25
2 Same as Current Hukou	64	0.82
3 This Village/Neighborhood	141	1.80
4 Another Village/Neighborthood in this Country/City	644	8.20
5 Another County/City in this Province	224	2.85
6 Another Province	163	2.08
Total	7,849	100.00

- **bc013_1 : City**

	No	%
01	11	4.91
02	1	0.45
04	11	4.91
05	2	0.89
11	17	7.59
16	1	0.45
24	10	4.46
27	7	3.13
28	1	0.45
35	4	1.79
40	29	12.95
42	1	0.45
46	33	14.73

49	5	2.23
51	1	0.45
52	1	0.45
53	5	2.23
55	11	4.91
56	5	2.23
60	17	7.59
63	6	2.68
74	9	4.02
76	1	0.45
82	20	8.93
86	6	2.68
96	8	3.57
99	1	0.45
Total	224	100.00

- **bc013_2 : County**

	No	%
02	25	11.68
03	3	1.40
04	4	1.87
06	9	4.21
08	1	0.47
16	6	2.80
28	17	7.94
31	24	11.21
33	1	0.47
37	2	0.93
43	2	0.93
44	2	0.93
45	1	0.47
46	10	4.67
51	1	0.47
54	5	2.34
59	35	16.36
63	30	14.02
75	1	0.47
76	21	9.81
78	3	1.40
81	8	3.74
91	3	1.40
Total	214	100.00

- **bc013_3 : Province**

	No	%
01	5	3.07
03	1	0.61

05	18	11.04
06	14	8.59
08	1	0.61
09	3	1.84
10	6	3.68
11	5	3.07
12	6	3.68
13	3	1.84
14	15	9.20
15	1	0.61
16	15	9.20
17	4	2.45
18	2	1.23
19	2	1.23
20	5	3.07
21	3	1.84
23	1	0.61
24	4	2.45
26	5	3.07
27	1	0.61
28	5	3.07
29	17	10.43
32	10	6.13
33	6	3.68
34	5	3.07
Total	163	100.00

- **bc013_4 : City**

	No	%
01	9	5.59
02	1	0.62
04	10	6.21
11	16	9.94
16	3	1.86
24	14	8.70
27	6	3.73
28	5	3.11
40	25	15.53
46	9	5.59
49	6	3.73
51	1	0.62
52	1	0.62
53	2	1.24
55	13	8.07
60	8	4.97
63	5	3.11
74	6	3.73
76	1	0.62
82	8	4.97
86	5	3.11

88	1	0.62
96	2	1.24
99	4	2.48
Total	161	100.00

- **bc013_5 : County**

	No	%
02	13	9.15
03	4	2.82
04	2	1.41
06	12	8.45
07	2	1.41
16	4	2.82
19	1	0.70
23	3	2.11
28	5	3.52
31	18	12.68
33	3	2.11
39	3	2.11
43	11	7.75
46	4	2.82
51	2	1.41
54	8	5.63
56	1	0.70
59	12	8.45
63	12	8.45
75	1	0.70
76	5	3.52
78	2	1.41
81	9	6.34
83	1	0.70
91	1	0.70
92	2	1.41
99	1	0.70
Total	142	100.00

- **bc014 : Why the Location of Current Hukou is Different from Your First Hukou**

	No	%
1 marriage	4,991	81.05
2 go to school	26	0.42
3 employment	232	3.77
4 retirement/ revolutionary retirement	16	0.26
5 sent down to the countryside to do manual labor	24	0.39
6 migration	595	9.66
7 migration of the whole village	36	0.58
8 escape of famine	93	1.51

9 others, pls specify:	145	2.35
Total	6,158	100.00

- **bc015 : The Reason of Non-Agricultural Hukou**

	No	%
1 sent down during 1967-1977 and did not come back to city	13	10.57
2 was a student	2	1.63
3 marriage	18	14.63
4 employment	22	17.89
6 escape of famine	5	4.07
7 others, pls specify:	63	51.22
Total	123	100.00

- **bc016_1 : Year**

Mean	Min	Max	OBS
1,958.6	1,927.0	1,997.0	101

- **bc016_2 : Year**

Mean	Min	Max	OBS
1,973.1	1,938.0	2,010.0	99

- **bc017 : The Reason of Agricultural Hukou**

	No	%
1 sent down during 1967-1977 and then come back to city	54	3.08
2 live in rural areas before go to college student	1,266	72.18
3 marriage	72	4.10
4 employment	140	7.98
5 Land is acquired by the government	49	2.79
6 migration of the whole village	5	0.29
7 escape of famine	2	0.11
8 others,pls specify:	166	9.46
Total	1,754	100.00

- **bc018_1 : Year**

Mean	Min	Max	OBS
1,950.7	1,910.0	1,987.0	1,717

- **bc018_2 : Year**

Mean	Min	Max	OBS
1,979.9	1,935.0	2,010.0	1,716

- **bd001 : Highest Level of Education Attained**

	No	%
1 No formal education illiterate	4,803	27.21
2 Did not finish primary school but capable of reading or writing	3,056	17.31
3 Sishu	84	0.48
4 Elementary school	3,812	21.59
5 Middle school	3,652	20.69
6 High school	1,388	7.86
7 Vocational school	429	2.43
8 Two/Three Year College / Associate degree	280	1.59
9 Four Year College / Bachelors degree	143	0.81
10 Post-graduate, Masters degree	6	0.03
Total	17,653	100.00

- **bd002 : Highest Grade Finished in Primary School**

	No	%
1	363	11.86
2	704	23.00
3	888	29.01
4	677	22.12
5	375	12.25
6	54	1.76
Total	3,061	100.00

- **bd003 : Additional Years of Schooling after bd001**

	No	%
0	8,797	89.83
1	485	4.95
2	376	3.84
3	80	0.82
4	15	0.15
5	8	0.08
6	3	0.03
7	8	0.08
8	13	0.13
9	5	0.05
10	1	0.01
11	2	0.02

Total	9,793	100.00

- **bd004 : when did You Go to College**

Mean	Min	Max	OBS
1,981.7	1,932.0	2,009.0	410

- **bd005 : Age that Began Formal Schooling**

Mean	Min	Max	OBS
8.1	0.0	58.0	12,324

- **bd006 : Age that Finished Schooling**

	No	%
0	8	0.06
1	1	0.01
2	1	0.01
3	2	0.02
4	1	0.01
5	3	0.02
6	4	0.03
7	32	0.26
8	120	0.97
9	273	2.21
10	509	4.12
11	615	4.98
12	975	7.89
13	1,294	10.47
14	1,249	10.11
15	1,401	11.34
16	1,719	13.91
17	1,342	10.86
18	1,091	8.83
19	609	4.93
20	419	3.39
21	179	1.45
22	118	0.96
23	83	0.67
24	60	0.49
25	50	0.40
26	28	0.23
27	14	0.11
28	17	0.14
29	8	0.06
30	12	0.10
31	13	0.11

32	10	0.08
33	8	0.06
34	9	0.07
35	6	0.05
36	8	0.06
37	8	0.06
38	6	0.05
39	1	0.01
40	11	0.09
41	4	0.03
42	4	0.03
43	4	0.03
44	3	0.02
45	3	0.02
46	1	0.01
47	3	0.02
48	5	0.04
49	1	0.01
50	2	0.02
51	1	0.01
52	2	0.02
55	1	0.01
57	2	0.02
78	1	0.01
79	1	0.01
90	1	0.01
Total	12,356	100.00

- **bd007s1 : Adult School**

	No	%
1 None	16,650	100.00
Total	16,650	100.00

- **bd007s2 : Adult School**

	No	%
2 TV University	59	100.00
Total	59	100.00

- **bd007s3 : Adult School**

	No	%
3 Evening Schoo	288	100.00
Total	288	100.00

- **bd007s4 : Adult School**

	No	%
4 Zikao	92	100.00
Total	92	100.00

- **bd007s5 : Adult School**

	No	%
5 Hanshou	242	100.00
Total	242	100.00

- **bd007s6 : Adult School**

	No	%
6 Saomang Class	201	100.00
Total	201	100.00

- **bd007s7 : Adult School**

	No	%
7 Sucheng Class	36	100.00
Total	36	100.00

- **bd007s8 : Adult School**

	No	%
8 Other explain	118	100.00
Total	118	100.00

- **bd008 : Years Spend in Adult School**

Mean	Min	Max	OBS
2.0	0.0	14.0	985

- **bd009 : Did You Get Diploma or Degree from Adult Education Program**

	No	%
1 Yes	408	40.56
2 No	598	59.44

Total	1,006	100.00

- **bd010 : When did You Receive the Diploma**

Mean	Min	Max	OBS
1,990.5	1,948.0	2,010.0	370

- **bd011 : Highest level of Schooling Obtained from the Adult Education Program**

	No	%
1 Vocational school	117	28.82
2 Two/Three Year College / Associate degree	194	47.78
3 Four Year College / Bachelors degree	45	11.08
4 Others	50	12.32
Total	406	100.00

- **be001 : Marital Status**

	No	%
1 Married with spouse present	14,170	80.17
2 Married but not living with spouse temporarily for reasons such as work	1,247	7.06
3 Separated	83	0.47
4 Divorced	147	0.83
5 Widowed	1,869	10.57
6 Never married	159	0.90
Total	17,675	100.00

- **be002 : Are You Unmarried but Living with a Partner**

	No	%
1 Yes	34	1.51
2 No	2,223	98.49
Total	2,257	100.00

- **be003 : How Many Times have You Been Married**

	No	%
0	17	0.10
1	16,735	95.57
2	718	4.10
3	36	0.21
4	2	0.01

5	1	0.01
6	1	0.01
Total	17,510	100.00

- **be004_1 : Year**

Mean	Min	Max	OBS
1,974.8	1,900.0	2,010.0	16,490

- **be004_2 : Month**

	No	%
0	2,983	18.09
1	1,826	11.08
2	1,203	7.30
3	704	4.27
4	580	3.52
5	619	3.75
6	478	2.90
7	484	2.94
8	818	4.96
9	772	4.68
10	1,795	10.89
11	1,398	8.48
12	2,827	17.15
Total	16,487	100.00

- **be005 : Value of Cash and Goods Received from Parents When Married**

Mean	Min	Max	OBS
281.5	-2,000.0	50,000.0	16,399

- **be006_1 : Year**

Mean	Min	Max	OBS
1,971.0	1,937.0	2,009.0	701

- **be006_2 : Month**

	No	%
0	256	36.62
1	59	8.44
2	40	5.72

3	23	3.29
4	19	2.72
5	32	4.58
6	18	2.58
7	20	2.86
8	28	4.01
9	35	5.01
10	60	8.58
11	35	5.01
12	74	10.59
Total	699	100.00

- **be007 : Year Your First Marriage End**

Mean	Min	Max	OBS
1,983.6	1,900.0	2,009.0	754

- **be008 : Why End Your First Marriage**

	No	%
1 Death of spouse	409	54.10
2 Divorce	347	45.90
Total	756	100.00

- **be009_1 : Year**

Mean	Min	Max	OBS
1,988.1	1,944.0	2,010.0	763

- **be009_2 : Month**

	No	%
0	212	27.79
1	64	8.39
2	46	6.03
3	34	4.46
4	37	4.85
5	37	4.85
6	40	5.24
7	46	6.03
8	52	6.82
9	32	4.19
10	70	9.17
11	39	5.11
12	54	7.08

Total	763	100.00

- **bf001 : Spouse/Partner's Chinese Zodiac**

	No	%
1 Rat	141	7.30
2 Ox	149	7.71
3 Tiger	162	8.39
4 Rabbit	161	8.33
5 Dragon	169	8.75
6 Snake	170	8.80
7 Horse	171	8.85
8 Goat	138	7.14
9 Monkey	170	8.80
10 Rooster	185	9.58
11 Dog	149	7.71
12 Pig	167	8.64
Total	1,932	100.00

- **bf002_1 : Spouse/Partner's Born Year**

Mean	Min	Max	OBS
1,941.0	1,900.0	1,985.0	1,889

- **bf002_2 : Spouse/Partner's Born Month**

	No	%
0	588	31.13
1	123	6.51
2	111	5.88
3	95	5.03
4	90	4.76
5	122	6.46
6	108	5.72
7	115	6.09
8	101	5.35
9	114	6.03
10	112	5.93
11	92	4.87
12	117	6.19
20	1	0.05
Total	1,889	100.00

- **bf003 : Solar or Lunar Calendar**

	No	%
1 Solar calendar	359	18.27
2 Lunar calendar	1,606	81.73
Total	1,965	100.00

- **bf004 : Highest Level of Education of Spouse/Partner**

	No	%
1 No formal education illiterate	896	44.27
2 Did not finish primary school but capable of reading or writing	260	12.85
3 Sishu	56	2.77
4 Elementary school	387	19.12
5 Middle school	263	12.99
6 High school	95	4.69
7 Vocational school	33	1.63
8 Two/Three Year College / Associate degree	17	0.84
9 Four Year College / Bachelors degree	17	0.84
Total	2,024	100.00

- **bf005_1 : Year Separated**

Mean	Min	Max	OBS
1,999.7	1,977.0	2,011.0	74

- **bf005_2 : Month Separated**

	No	%
0	22	29.73
1	12	16.22
2	4	5.41
3	3	4.05
4	7	9.46
5	3	4.05
6	6	8.11
7	2	2.70
8	5	6.76
9	3	4.05
10	3	4.05
11	2	2.70
12	2	2.70
Total	74	100.00

- **bf006_1 : Year Divorced**

Mean	Min	Max	OBS

1,994.7	1,954.0	2,011.0	143

- **bf006_2 : Month Divorced**

	No	%
0	57	39.86
1	12	8.39
2	4	2.80
3	11	7.69
4	5	3.50
5	11	7.69
6	7	4.90
7	6	4.20
8	7	4.90
9	7	4.90
10	7	4.90
11	5	3.50
12	4	2.80
Total	143	100.00

- **bf007_1 : Year Spouse Passed Away**

Mean	Min	Max	OBS
1,998.6	1,941.0	2,012.0	1,848

- **bf007_2 : Month Spouse Passed Away**

	No	%
0	386	20.89
1	148	8.01
2	150	8.12
3	122	6.60
4	110	5.95
5	134	7.25
6	112	6.06
7	119	6.44
8	103	5.57
9	102	5.52
10	126	6.82
11	112	6.06
12	124	6.71
Total	1,848	100.00

- **bf008 : How Often did the R Receive Assistance**

	No	%
1 Never	14,549	82.36
2 A few times	2,489	14.09
3 Most or all the time	314	1.78
4 The section was done by a proxy reporter (the respondent is absent)	313	1.77
Total	17,665	100.00

- **bf009 : Relationship to R**

	No	%
1 Spouse	223	68.83
2 Mother	3	0.93
3 Father	2	0.62
4 Mother-in-law	3	0.93
6 Sibling	4	1.23
8 Child	58	17.90
9 Spouse of child	19	5.86
10 Grandchildren	8	2.47
11 Other relative	3	0.93
12 Helper or other non-relative	1	0.31
Total	324	100.00

- **bf010 : Reason for Proxy**

	No	%
1 The respondent has serious physical handicaps	46	14.51
2 The respondent has serious mental handicaps	12	3.79
3 The respondent has rejected this interview	28	8.83
4 Other:	231	72.87
Total	317	100.00

- **proxy : Interview Down By Proxy**

	No	%
0 No	16,455	92.99
1 Yes	1,240	7.01
Total	17,695	100.00

- **rgender : Gender**

	No	%
	1	0.01
1 Male	8,471	47.88

2 Female	9,221	52.12
Total	17,693	100.00

3 Family Information

- **householdID : Household ID**

A String Variable	
OBS:	10,199

- **ID : Individual ID**

A String Variable	
OBS:	10,199

- **communityID : Community ID**

A String Variable	
OBS:	10,199

- **ca001_1_ : Whether Parent Still Living**

	No	%
1 Yes	1,349	13.48
2 No	8,655	86.52
Total	10,004	100.00

- **ca001_2_ : Whether Parent Still Living**

	No	%
1 Yes	2,335	23.73
2 No	7,503	76.27
Total	9,838	100.00

- **ca001_3_ : Whether Parent Still Living**

	No	%
1 Yes	1,325	16.62
2 No	6,649	83.38
Total	7,974	100.00

- **ca001_4_ : Whether Parent Still Living**

	No	%

1 Yes	2,303	29.18
2 No	5,589	70.82
Total	7,892	100.00

- **ca003_1_ : The Type of Your Parent**

	No	%
1 Biological	9,955	98.37
2 Adoptive	105	1.04
3 Step	50	0.49
4 A different biological relative who raised you	5	0.05
5 Another individual who raised you	5	0.05
Total	10,120	100.00

- **ca003_2_ : The Type of Your Parent**

	No	%
1 Biological	9,955	98.46
2 Adoptive	85	0.84
3 Step	58	0.57
4 A different biological relative who raised you	8	0.08
5 Another individual who raised you	5	0.05
Total	10,111	100.00

- **ca003_3_ : The Type of Your Parent**

	No	%
1 Biological	7,895	98.37
2 Adoptive	74	0.92
3 Step	53	0.66
4 A different biological relative who raised you	3	0.04
5 Another individual who raised you	1	0.01
Total	8,026	100.00

- **ca003_4_ : The Type of Your Parent**

	No	%
1 Biological	7,905	98.57
2 Adoptive	71	0.89
3 Step	39	0.49
4 A different biological relative who raised you	5	0.06
Total	8,020	100.00

- **ca004_1_ : Where was Your Parent Born**

	No	%
1 This Village/Neighborhood	588	40.08
2 Another Village/Neighborhood in this County/city	629	42.88
3 Another County/City in this Province	127	8.66
4 Another Province	123	8.38
Total	1,467	100.00

- **ca004_1_1_ : City**

	No	%
01	7	5.47
02	1	0.78
04	10	7.81
05	2	1.56
11	7	5.47
16	5	3.91
24	5	3.91
27	8	6.25
40	25	19.53
42	1	0.78
46	12	9.38
49	2	1.56
51	1	0.78
53	6	4.69
55	5	3.91
56	1	0.78
60	2	1.56
63	3	2.34
74	5	3.91
82	14	10.94
86	1	0.78
96	3	2.34
99	2	1.56
Total	128	100.00

- **ca004_1_2_ : City**

	No	%
01	14	4.39
02	3	0.94
04	26	8.15
05	5	1.57
11	21	6.58
16	13	4.08
17	2	0.63
18	1	0.31

24	14	4.39
27	9	2.82
35	1	0.31
40	47	14.73
42	2	0.63
46	33	10.34
49	6	1.88
51	1	0.31
53	15	4.70
55	12	3.76
56	3	0.94
60	20	6.27
63	12	3.76
66	1	0.31
74	17	5.33
77	2	0.63
82	23	7.21
86	9	2.82
96	5	1.57
99	2	0.63
Total	319	100.00

- **ca004_1_3_ : City**

	No	%
01	5	4.67
02	1	0.93
04	9	8.41
11	10	9.35
16	1	0.93
24	7	6.54
27	1	0.93
40	17	15.89
46	11	10.28
49	3	2.80
51	1	0.93
52	1	0.93
53	5	4.67
55	8	7.48
60	6	5.61
63	2	1.87
74	3	2.80
82	12	11.21
86	3	2.80
96	1	0.93
Total	107	100.00

- **ca004_1_4_ : City**

	No	%
01	13	5.78
02	1	0.44
04	20	8.89
05	4	1.78
11	15	6.67
16	5	2.22
24	9	4.00
27	6	2.67
35	1	0.44
40	22	9.78
42	2	0.89
46	27	12.00
49	5	2.22
50	1	0.44
51	1	0.44
53	10	4.44
55	16	7.11
60	16	7.11
63	10	4.44
74	7	3.11
82	19	8.44
86	10	4.44
96	1	0.44
99	4	1.78
Total	225	100.00

- **ca004_2_ : Where was Your Parent Born**

	No	%
1 This Village/Neighborhood	436	16.83
2 Another Village/Neighborhood in this County/city	1,615	62.33
3 Another County/City in this Province	320	12.35
4 Another Province	220	8.49
Total	2,591	100.00

- **ca004_2_1_ : County**

	No	%
02	10	8.06
03	1	0.81
04	4	3.23
06	8	6.45
08	1	0.81
16	1	0.81
19	1	0.81
28	1	0.81
31	6	4.84

33	1	0.81
38	1	0.81
39	2	1.61
43	8	6.45
44	3	2.42
46	10	8.06
51	3	2.42
54	7	5.65
57	1	0.81
58	1	0.81
59	13	10.48
63	17	13.71
76	12	9.68
78	1	0.81
81	7	5.65
90	1	0.81
91	1	0.81
92	2	1.61
Total	124	100.00

- **ca004_2_2_ : County**

	No	%
02	27	8.88
03	1	0.33
04	4	1.32
06	26	8.55
08	1	0.33
10	1	0.33
13	1	0.33
16	3	0.99
28	10	3.29
31	23	7.57
33	2	0.66
37	6	1.97
38	4	1.32
39	6	1.97
43	17	5.59
44	4	1.32
46	11	3.62
50	1	0.33
51	14	4.61
54	20	6.58
56	2	0.66
57	1	0.33
59	36	11.84
63	43	14.14
75	1	0.33
76	20	6.58
78	1	0.33
81	12	3.95

90	1	0.33
91	3	0.99
92	1	0.33
99	1	0.33
Total	304	100.00

- **ca004_2_3_ : County**

	No	%
02	9	9.00
03	1	1.00
04	4	4.00
06	6	6.00
08	1	1.00
16	1	1.00
28	3	3.00
31	9	9.00
33	1	1.00
37	1	1.00
39	1	1.00
43	6	6.00
44	2	2.00
45	1	1.00
46	8	8.00
51	3	3.00
54	3	3.00
57	4	4.00
59	7	7.00
63	10	10.00
76	11	11.00
81	5	5.00
90	1	1.00
91	1	1.00
99	1	1.00
Total	100	100.00

- **ca004_2_4_ : County**

	No	%
02	16	7.44
04	7	3.26
06	24	11.16
19	1	0.47
28	3	1.40
31	22	10.23
33	3	1.40
37	2	0.93
39	5	2.33
43	15	6.98

45	3	1.40
46	13	6.05
51	6	2.79
54	13	6.05
57	4	1.86
59	23	10.70
63	26	12.09
75	1	0.47
76	14	6.51
81	8	3.72
90	2	0.93
91	2	0.93
98	1	0.47
99	1	0.47
Total	215	100.00

- **ca004_3_ : Where was Your Parent Born**

	No	%
1 This Village/Neighborhood	605	43.37
2 Another Village/Neighborhood in this County/city	588	42.15
3 Another County/City in this Province	106	7.60
4 Another Province	95	6.81
5 Abroad	1	0.07
Total	1,395	100.00

- **ca004_3_1_ : Province**

	No	%
01	5	4.00
03	1	0.80
05	8	6.40
06	7	5.60
07	3	2.40
09	2	1.60
10	1	0.80
11	3	2.40
12	3	2.40
13	3	2.40
14	5	4.00
16	14	11.20
17	4	3.20
20	3	2.40
21	10	8.00
23	1	0.80
24	4	3.20
26	6	4.80
27	1	0.80
29	21	16.80

32	11	8.80
33	8	6.40
34	1	0.80
Total	125	100.00

- **ca004_3_2_ : Province**

	No	%
01	7	3.17
03	2	0.90
05	12	5.43
06	25	11.31
07	4	1.81
09	2	0.90
10	4	1.81
11	15	6.79
12	9	4.07
13	4	1.81
14	7	3.17
15	3	1.36
16	29	13.12
17	2	0.90
18	2	0.90
19	2	0.90
20	5	2.26
21	7	3.17
23	1	0.45
24	10	4.52
25	1	0.45
26	4	1.81
27	4	1.81
28	4	1.81
29	32	14.48
32	16	7.24
33	6	2.71
34	2	0.90
Total	221	100.00

- **ca004_3_3_ : Province**

	No	%
01	3	3.13
03	3	3.13
05	8	8.33
06	9	9.38
07	2	2.08
09	1	1.04
10	4	4.17
11	5	5.21

12	5	5.21
13	1	1.04
14	2	2.08
16	10	10.42
17	1	1.04
18	1	1.04
20	2	2.08
21	5	5.21
24	4	4.17
26	3	3.13
27	1	1.04
28	2	2.08
29	9	9.38
32	8	8.33
33	5	5.21
34	2	2.08
Total	96	100.00

- **ca004_3_4_ : Province**

	No	%
01	3	1.66
03	1	0.55
05	13	7.18
06	21	11.60
07	2	1.10
09	3	1.66
10	3	1.66
11	11	6.08
12	3	1.66
13	4	2.21
14	8	4.42
15	2	1.10
16	15	8.29
17	7	3.87
19	2	1.10
20	5	2.76
21	9	4.97
23	2	1.10
24	8	4.42
25	1	0.55
26	6	3.31
27	2	1.10
28	3	1.66
29	25	13.81
32	17	9.39
33	4	2.21
34	1	0.55
Total	181	100.00

- **ca004_4_ : Where was Your Parent Born**

	No	%
1 This Village/Neighborhood	475	19.86
2 Another Village/Neighborhood in this County/city	1,509	63.09
3 Another County/City in this Province	225	9.41
4 Another Province	180	7.53
5 Abroad	3	0.13
Total	2,392	100.00

- **ca004_4_1_ : City**

	No	%
01	2	1.77
04	12	10.62
11	11	9.73
16	2	1.77
24	17	15.04
27	6	5.31
40	10	8.85
46	4	3.54
49	1	0.88
53	6	5.31
55	9	7.96
56	1	0.88
60	2	1.77
63	6	5.31
74	7	6.19
82	10	8.85
86	4	3.54
96	1	0.88
99	2	1.77
Total	113	100.00

- **ca004_4_2_ : City**

	No	%
01	8	3.86
04	11	5.31
07	1	0.48
11	19	9.18
16	5	2.42
18	1	0.48
24	30	14.49
27	7	3.38
28	1	0.48
38	1	0.48
40	29	14.01

46	13	6.28
49	5	2.42
51	1	0.48
53	12	5.80
55	10	4.83
60	8	3.86
63	5	2.42
74	10	4.83
77	1	0.48
82	12	5.80
84	1	0.48
86	7	3.38
96	1	0.48
99	8	3.86
Total	207	100.00

- **ca004_4_3_ : City**

	No	%
01	2	2.35
04	2	2.35
11	7	8.24
16	1	1.18
24	10	11.76
27	4	4.71
40	7	8.24
46	8	9.41
49	7	8.24
51	1	1.18
52	1	1.18
53	3	3.53
55	8	9.41
60	5	5.88
63	5	5.88
74	1	1.18
76	1	1.18
82	10	11.76
86	2	2.35
Total	85	100.00

- **ca004_4_4_ : City**

	No	%
01	6	3.61
04	11	6.63
05	1	0.60
11	4	2.41
16	5	3.01
24	23	13.86

27	4	2.41
28	1	0.60
40	25	15.06
46	14	8.43
49	9	5.42
50	1	0.60
53	9	5.42
55	9	5.42
60	9	5.42
63	9	5.42
74	6	3.61
76	1	0.60
82	10	6.02
86	6	3.61
99	3	1.81
Total	166	100.00

- **ca004_5_1_ : County**

	No	%
02	6	5.61
03	3	2.80
04	2	1.87
06	10	9.35
16	1	0.93
28	2	1.87
31	9	8.41
33	2	1.87
37	1	0.93
38	2	1.87
39	9	8.41
43	7	6.54
44	3	2.80
46	2	1.87
49	1	0.93
51	3	2.80
54	9	8.41
57	1	0.93
59	6	5.61
63	19	17.76
76	4	3.74
78	1	0.93
81	2	1.87
83	1	0.93
92	1	0.93
Total	107	100.00

- **ca004_5_2_ : County**

	No	%
02	6	3.09
03	4	2.06
04	4	2.06
06	15	7.73
13	1	0.52
23	3	1.55
28	2	1.03
31	25	12.89
33	1	0.52
37	2	1.03
38	2	1.03
39	10	5.15
43	17	8.76
44	3	1.55
46	6	3.09
49	2	1.03
50	1	0.52
51	4	2.06
54	9	4.64
57	1	0.52
59	14	7.22
63	36	18.56
76	11	5.67
78	2	1.03
81	5	2.58
83	1	0.52
91	2	1.03
92	3	1.55
99	2	1.03
Total	194	100.00

- **ca004_5_3_ : County**

	No	%
02	7	9.09
03	2	2.60
04	3	3.90
06	7	9.09
13	1	1.30
31	8	10.39
39	4	5.19
43	3	3.90
44	3	3.90
46	1	1.30
49	1	1.30
50	1	1.30
51	2	2.60
54	5	6.49
57	1	1.30

59	7	9.09
63	12	15.58
76	6	7.79
78	1	1.30
81	1	1.30
90	1	1.30
Total	77	100.00

- **ca004_5_4_ : County**

	No	%
02	7	4.67
03	5	3.33
04	2	1.33
06	12	8.00
13	2	1.33
23	1	0.67
28	1	0.67
31	14	9.33
33	2	1.33
39	14	9.33
43	2	1.33
44	3	2.00
46	4	2.67
49	1	0.67
50	1	0.67
51	3	2.00
54	10	6.67
57	2	1.33
58	1	0.67
59	10	6.67
63	35	23.33
76	9	6.00
78	1	0.67
81	6	4.00
91	1	0.67
92	1	0.67
Total	150	100.00

- **ca005_1_ : Area Parent Growed Up**

	No	%
1 City	114	7.69
2 Village	1,369	92.31
Total	1,483	100.00

- **ca005_2_ : Area Parent Growed Up**

	No	%
1 City	207	7.88
2 Village	2,419	92.12
Total	2,626	100.00

- **ca005_3_ : Area Parent Growed Up**

	No	%
1 City	112	7.95
2 Village	1,296	92.05
Total	1,408	100.00

- **ca005_4_ : Area Parent Growed Up**

	No	%
1 City	182	7.48
2 Village	2,250	92.52
Total	2,432	100.00

- **ca006_1_ : CHINESE ZODIAC**

	No	%
1 Rat	101	8.09
2 Ox	110	8.81
3 Tiger	101	8.09
4 Rabbit	108	8.65
5 Dragon	101	8.09
6 Snake	101	8.09
7 Horse	104	8.33
8 Goat	105	8.41
9 Monkey	96	7.69
10 Rooster	130	10.42
11 Dog	88	7.05
12 Pig	103	8.25
Total	1,248	100.00

- **ca006_2_ : CHINESE ZODIAC**

	No	%
1 Rat	164	7.74
2 Ox	172	8.11
3 Tiger	157	7.41
4 Rabbit	175	8.25
5 Dragon	179	8.44

6 Snake	180	8.49
7 Horse	166	7.83
8 Goat	187	8.82
9 Monkey	152	7.17
10 Rooster	201	9.48
11 Dog	190	8.96
12 Pig	197	9.29
Total	2,120	100.00

- **ca006_3_ : CHINESE ZODIAC**

	No	%
1 Rat	95	8.57
2 Ox	88	7.94
3 Tiger	94	8.48
4 Rabbit	87	7.84
5 Dragon	110	9.92
6 Snake	69	6.22
7 Horse	95	8.57
8 Goat	102	9.20
9 Monkey	83	7.48
10 Rooster	101	9.11
11 Dog	75	6.76
12 Pig	110	9.92
Total	1,109	100.00

- **ca006_4_ : CHINESE ZODIAC**

	No	%
1 Rat	135	6.97
2 Ox	173	8.93
3 Tiger	169	8.72
4 Rabbit	169	8.72
5 Dragon	148	7.64
6 Snake	159	8.20
7 Horse	166	8.57
8 Goat	167	8.62
9 Monkey	168	8.67
10 Rooster	166	8.57
11 Dog	153	7.89
12 Pig	165	8.51
Total	1,938	100.00

- **ca007_1_ : Birth Year of Parent**

Mean	Min	Max	OBS
1,081.4	-9,999.0	9,999.0	10,044

- **ca007_2_ : Birth Year of Parent**

Mean	Min	Max	OBS
1,174.8	-9,999.0	9,999.0	10,026

- **ca007_3_ : Birth Year of Parent**

Mean	Min	Max	OBS
998.0	-9,999.0	9,999.0	7,935

- **ca007_4_ : Birth Year of Parent**

Mean	Min	Max	OBS
1,128.7	-9,999.0	9,999.0	7,937

- **ca008_1_1_ : Year Parent Passed Away**

Mean	Min	Max	OBS
1,942.9	-9,999.0	9,999.0	3,074

- **ca008_1_2_ : Year Parent Passed Away**

Mean	Min	Max	OBS
1,940.7	-9,999.0	9,999.0	2,836

- **ca008_1_3_ : Year Parent Passed Away**

Mean	Min	Max	OBS
1,970.3	-9,999.0	9,999.0	2,350

- **ca008_1_4_ : Year Parent Passed Away**

Mean	Min	Max	OBS
1,971.7	-9,999.0	9,999.0	1,972

- **ca008_2_1_ : Age Parent Passed Away**

Mean	Min	Max	OBS
68.6	3.0	108.0	4,833

- **ca008_2_2_ : Age Parent Passed Away**

Mean	Min	Max	OBS
69.9	1.0	103.0	4,023

- **ca008_2_3_ : Age Parent Passed Away**

	No	%
1	1	0.03
7	1	0.03
10	1	0.03
21	1	0.03
22	2	0.06
23	1	0.03
24	1	0.03
25	2	0.06
26	1	0.03
27	4	0.12
28	3	0.09
29	6	0.18
30	25	0.75
31	2	0.06
32	5	0.15
33	2	0.06
34	1	0.03
35	10	0.30
36	17	0.51
37	7	0.21
38	9	0.27
39	3	0.09
40	47	1.42
41	10	0.30
42	15	0.45
43	11	0.33
44	12	0.36
45	25	0.75
46	6	0.18
47	13	0.39
48	26	0.78
49	15	0.45
50	73	2.20
51	18	0.54
52	20	0.60
53	26	0.78

54	21	0.63
55	40	1.21
56	37	1.12
57	30	0.90
58	38	1.15
59	53	1.60
60	177	5.34
61	38	1.15
62	63	1.90
63	74	2.23
64	42	1.27
65	87	2.62
66	65	1.96
67	49	1.48
68	68	2.05
69	85	2.56
70	214	6.45
71	83	2.50
72	101	3.05
73	160	4.83
74	95	2.86
75	107	3.23
76	93	2.80
77	71	2.14
78	96	2.90
79	81	2.44
80	175	5.28
81	76	2.29
82	82	2.47
83	85	2.56
84	93	2.80
85	67	2.02
86	43	1.30
87	31	0.93
88	27	0.81
89	26	0.78
90	30	0.90
91	14	0.42
92	20	0.60
93	19	0.57
94	6	0.18
95	5	0.15
96	12	0.36
97	4	0.12
98	4	0.12
99	2	0.06
100	5	0.15
Total	3,316	100.00

- **ca008_2_4_ : Age Parent Passed Away**

Mean	Min	Max	OBS
70.0	1.0	106.0	2,815

- **ca009_1_ : Parent' Highest Level of Education**

	No	%
1 No formal education illiterate	5,872	62.20
2 Did not finish primary school but capable of reading or writing	1,093	11.58
3 Sishu	897	9.50
4 Elementary school	948	10.04
5 Middle school	327	3.46
6 High school	134	1.42
7 Vocational school	92	0.97
8 Two/Three Year College / Associate degree	25	0.26
9 Four Year College / Bachelors degree	49	0.52
10 Post-graduate, Masters degree	3	0.03
Total	9,440	100.00

- **ca009_2_ : Parent' Highest Level of Education**

	No	%
1 No formal education illiterate	8,458	88.70
2 Did not finish primary school but capable of reading or writing	400	4.19
3 Sishu	124	1.30
4 Elementary school	379	3.97
5 Middle school	99	1.04
6 High school	42	0.44
7 Vocational school	21	0.22
8 Two/Three Year College / Associate degree	4	0.04
9 Four Year College / Bachelors degree	9	0.09
Total	9,536	100.00

- **ca009_3_ : Parent' Highest Level of Education**

	No	%
1 No formal education illiterate	4,836	65.62
2 Did not finish primary school but capable of reading or writing	768	10.42
3 Sishu	531	7.20
4 Elementary school	720	9.77
5 Middle school	273	3.70
6 High school	107	1.45
7 Vocational school	76	1.03
8 Two/Three Year College / Associate degree	24	0.33
9 Four Year College / Bachelors degree	35	0.47
Total	7,370	100.00

- **ca009_4_ : Parent' Highest Level of Education**

	No	%
1 No formal education illiterate	6,771	89.58
2 Did not finish primary school but capable of reading or writing	263	3.48
3 Sishu	84	1.11
4 Elementary school	299	3.96
5 Middle school	79	1.05
6 High school	30	0.40
7 Vocational school	26	0.34
8 Two/Three Year College / Associate degree	3	0.04
9 Four Year College / Bachelors degree	4	0.05
Total	7,559	100.00

- **ca010_1_ : Parent' Marital Status**

	No	%
1 Married, with my mother	879	64.73
2 Married, but not with my mother	31	2.28
3 Separated	4	0.29
4 Divorced	7	0.52
5 Widowed	436	32.11
6 Never Married	1	0.07
Total	1,358	100.00

- **ca010_2_ : Parent' Marital Status**

	No	%
1 Married, with my mother	917	39.24
2 Married, but not with my mother	27	1.16
3 Separated	4	0.17
4 Divorced	6	0.26
5 Widowed	1,383	59.18
Total	2,337	100.00

- **ca010_3_ : Parent' Marital Status**

	No	%
1 Married, with my mother	903	67.49
2 Married, but not with my mother	16	1.20
3 Separated	3	0.22
4 Divorced	4	0.30
5 Widowed	410	30.64
6 Never Married	2	0.15
Total	1,338	100.00

- **ca010_4_ : Parent' Marital Status**

	No	%
1 Married, with my mother	946	41.08
2 Married, but not with my mother	21	0.91
3 Separated	3	0.13
4 Divorced	6	0.26
5 Widowed	1,326	57.58
6 Never Married	1	0.04
Total	2,303	100.00

- **ca011_1_ : Whether Living with a Partner**

	No	%
1 Yes	18	4.06
2 No	425	95.94
Total	443	100.00

- **ca011_2_ : Whether Living with a Partner**

	No	%
1 Yes	30	2.18
2 No	1,344	97.82
Total	1,374	100.00

- **ca011_3_ : Whether Living with a Partner**

	No	%
1 Yes	15	3.63
2 No	398	96.37
Total	413	100.00

- **ca011_4_ : Whether Living with a Partner**

	No	%
1 Yes	37	2.83
2 No	1,270	97.17
Total	1,307	100.00

- **ca012_1_ : Does Your Parent Work Currently**

	No	%

	No	%
1 Yes	228	15.61
2 No	1,233	84.39
Total	1,461	100.00

- **ca012_2_ : Does Your Parent Work Currently**

	No	%
1 Yes	229	8.71
2 No	2,399	91.29
Total	2,628	100.00

- **ca012_3_ : Does Your Parent Work Currently**

	No	%
1 Yes	192	13.83
2 No	1,196	86.17
Total	1,388	100.00

- **ca012_4_ : Does Your Parent Work Currently**

	No	%
1 Yes	189	7.76
2 No	2,245	92.24
Total	2,434	100.00

- **ca013_1_ : How is Your Parent' Health**

	No	%
1 Very good	101	6.98
2 Good	257	17.75
3 Fair	622	42.96
4 Poor	383	26.45
5 Very poor	85	5.87
Total	1,448	100.00

- **ca013_2_ : How is Your Parent' Health**

	No	%
1 Very good	115	4.39
2 Good	422	16.12
3 Fair	1,098	41.94
4 Poor	792	30.25
5 Very poor	191	7.30

Total	2,618	100.00

- **ca013_3_ : How is Your Parent' Health**

	No	%
1 Very good	82	5.94
2 Good	282	20.42
3 Fair	583	42.22
4 Poor	330	23.90
5 Very poor	104	7.53
Total	1,381	100.00

- **ca013_4_ : How is Your Parent' Health**

	No	%
1 Very good	101	4.15
2 Good	395	16.25
3 Fair	1,015	41.75
4 Poor	748	30.77
5 Very poor	172	7.08
Total	2,431	100.00

- **ca014_1_ : Highest Occupation of Your Parent**

	No	%
1 Managers	216	14.93
2 Professionals and technicians	60	4.15
3 Clerks	68	4.70
4 Commercial and service workers	36	2.49
5 Agricultural, forestry, husbandry and fishery producers	906	62.61
6 Production and transportation workers	107	7.39
	54	3.73
Total	1,447	100.00

- **ca014_2_ : Highest Occupation of Your Parent**

	No	%
1 Managers	73	2.79
2 Professionals and technicians	34	1.30
3 Clerks	32	1.22
4 Commercial and service workers	53	2.02
5 Agricultural, forestry, husbandry and fishery producers	2,149	82.02
6 Production and transportation workers	98	3.74
	181	6.91
Total	2,620	100.00

- **ca014_3_ : Highest Occupation of Your Parent**

	No	%
1 Managers	172	12.50
2 Professionals and technicians	49	3.56
3 Clerks	53	3.85
4 Commercial and service workers	29	2.11
5 Agricultural, forestry, husbandry and fishery producers	953	69.26
6 Production and transportation workers	75	5.45
	45	3.27
Total	1,376	100.00

- **ca014_4_ : Highest Occupation of Your Parent**

	No	%
1 Managers	54	2.22
2 Professionals and technicians	28	1.15
3 Clerks	28	1.15
4 Commercial and service workers	40	1.65
5 Agricultural, forestry, husbandry and fishery producers	2,003	82.46
6 Production and transportation workers	94	3.87
	182	7.49
Total	2,429	100.00

- **ca015_1_1_ : Income of your Parent, (Yuan/Year)**

Mean	Min	Max	OBS
2,875.1	0.0	20,000.0	144

- **ca015_1_2_ : Income of your Parent, (Yuan/Year)**

Mean	Min	Max	OBS
1,636.4	0.0	20,000.0	152

- **ca015_1_3_ : Income of your Parent, (Yuan/Year)**

Mean	Min	Max	OBS
3,904.1	0.0	40,000.0	121

- **ca015_1_4_ : Income of your Parent, (Yuan/Year)**

Mean	Min	Max	OBS
2,392.7	0.0	30,000.0	130

- **ca015_2_1_ : Income of your Parent, (Yuan/Month)**

Mean	Min	Max	OBS
382.9	0.0	1,800.0	59

- **ca015_2_2_ : Income of your Parent, (Yuan/Month)**

Mean	Min	Max	OBS
524.3	0.0	16,000.0	55

- **ca015_2_3_ : Income of your Parent, (Yuan/Month)**

Mean	Min	Max	OBS
455.1	0.0	3,000.0	39

- **ca015_2_4_ : Income of your Parent, (Yuan/Month)**

Mean	Min	Max	OBS
100.9	0.0	1,000.0	33

- **ca016_1_ : Where does Your Parent Normally Live**

	No	%
1 The same or an adjacent dwelling/courtyard with me	90	6.71
2 Another household in this Village/Neighborhood	474	35.35
3 Another Village in this County/City	629	46.91
4 Another County/City in this Province	87	6.49
5 Another Province	61	4.55
Total	1,341	100.00

- **ca016_1_1_ : Distance in the Same City**

Mean	Min	Max	OBS
16.0	-999.0	2,000.0	630

- **ca016_1_2_ : Distance in the Same City**

Mean	Min	Max	OBS
12.2	-999.0	250.0	1,018

- **ca016_1_3_ : Distance in the Same City**

Mean	Min	Max	OBS
12.7	0.1	200.0	562

- **ca016_1_4_ : Distance in the Same City**

Mean	Min	Max	OBS
13.7	0.0	300.0	1,033

- **ca016_2_ : Where does Your Parent Normally Live**

	No	%
1 The same or an adjacent dwelling/courtyard with me	176	7.56
2 Another household in this Village/Neighborhood	869	37.34
3 Another Village in this County/City	1,022	43.92
4 Another County/City in this Province	160	6.88
5 Another Province	100	4.30
Total	2,327	100.00

- **ca016_2_1_ : City**

	No	%
01	5	5.62
02	2	2.25
04	6	6.74
05	2	2.25
11	5	5.62
16	4	4.49
18	1	1.12
24	4	4.49
27	5	5.62
40	19	21.35
42	1	1.12
46	7	7.87
49	3	3.37
51	1	1.12
53	2	2.25
55	2	2.25

60	1	1.12
63	4	4.49
74	2	2.25
82	6	6.74
86	3	3.37
96	3	3.37
99	1	1.12
Total	89	100.00

- **ca016_2_2_ : City**

	No	%
01	4	2.52
04	10	6.29
05	2	1.26
07	1	0.63
11	11	6.92
16	8	5.03
17	3	1.89
18	1	0.63
24	9	5.66
27	7	4.40
40	24	15.09
42	2	1.26
46	13	8.18
49	5	3.14
51	1	0.63
53	7	4.40
55	4	2.52
56	2	1.26
60	8	5.03
63	5	3.14
66	1	0.63
74	8	5.03
77	1	0.63
82	11	6.92
86	5	3.14
96	5	3.14
99	1	0.63
Total	159	100.00

- **ca016_2_3_ : City**

	No	%
01	5	7.94
02	1	1.59
04	4	6.35
11	6	9.52
16	1	1.59

24	3	4.76
27	1	1.59
35	1	1.59
40	6	9.52
46	6	9.52
49	3	4.76
53	5	7.94
55	6	9.52
60	4	6.35
63	1	1.59
74	2	3.17
82	4	6.35
86	2	3.17
96	1	1.59
99	1	1.59
Total	63	100.00

- **ca016_2_4_ : City**

	No	%
01	7	6.42
02	1	0.92
04	9	8.26
05	1	0.92
11	5	4.59
16	3	2.75
24	6	5.50
27	2	1.83
35	3	2.75
40	9	8.26
42	2	1.83
46	13	11.93
49	2	1.83
50	1	0.92
53	4	3.67
55	5	4.59
60	7	6.42
63	5	4.59
74	3	2.75
82	13	11.93
86	5	4.59
96	1	0.92
99	2	1.83
Total	109	100.00

- **ca016_3_ : Where does Your Parent Normally Live**

	No	%
1 The same or an adjacent dwelling/courtyard with me	84	6.37

2 Another household in this Village/Neighborhood	541	41.05
3 Another Village in this County/City	565	42.87
4 Another County/City in this Province	63	4.78
5 Another Province	64	4.86
6 Abroad	1	0.08
Total	1,318	100.00

- **ca016_3_1_ : County**

	No	%
02	8	9.20
03	1	1.15
04	2	2.30
06	7	8.05
08	1	1.15
16	1	1.15
19	1	1.15
28	4	4.60
31	2	2.30
33	1	1.15
38	1	1.15
43	6	6.90
46	8	9.20
51	4	4.60
54	3	3.45
58	1	1.15
59	6	6.90
63	18	20.69
76	6	6.90
81	3	3.45
91	1	1.15
92	1	1.15
99	1	1.15
Total	87	100.00

- **ca016_3_2_ : County**

	No	%
02	12	8.00
03	2	1.33
04	8	5.33
06	8	5.33
08	1	0.67
16	2	1.33
19	1	0.67
28	5	3.33
31	8	5.33
33	2	1.33
38	1	0.67

43	7	4.67
44	1	0.67
46	10	6.67
49	1	0.67
51	7	4.67
54	8	5.33
58	1	0.67
59	17	11.33
63	25	16.67
75	1	0.67
76	11	7.33
81	8	5.33
90	1	0.67
92	1	0.67
99	1	0.67
Total	150	100.00

- **ca016_3_3_ : County**

	No	%
02	5	8.20
04	4	6.56
06	5	8.20
16	1	1.64
28	2	3.28
31	6	9.84
33	1	1.64
37	1	1.64
39	1	1.64
43	2	3.28
46	7	11.48
51	2	3.28
54	1	1.64
57	2	3.28
59	1	1.64
63	11	18.03
76	5	8.20
81	3	4.92
91	1	1.64
Total	61	100.00

- **ca016_3_4_ : County**

	No	%
02	9	8.41
03	1	0.93
04	6	5.61
06	10	9.35
19	1	0.93

28	2	1.87
31	12	11.21
33	1	0.93
37	1	0.93
39	1	0.93
43	5	4.67
44	2	1.87
45	1	0.93
46	12	11.21
51	1	0.93
54	2	1.87
57	3	2.80
59	7	6.54
63	15	14.02
76	7	6.54
78	1	0.93
81	2	1.87
89	1	0.93
90	2	1.87
98	1	0.93
99	1	0.93
Total	107	100.00

- **ca016_4_ : Where does Your Parent Normally Live**

	No	%
1 The same or an adjacent dwelling/courtyard with me	129	5.63
2 Another household in this Village/Neighborhood	916	39.97
3 Another Village in this County/City	1,036	45.20
4 Another County/City in this Province	109	4.76
5 Another Province	100	4.36
6 Abroad	2	0.09
Total	2,292	100.00

- **ca016_4_1_ : Distance of Another County in this Province**

Mean	Min	Max	OBS
294.8	0.5	10,000.0	86

- **ca016_4_2_ : Distance of Another County in this Province**

Mean	Min	Max	OBS
180.6	0.0	3,000.0	158

- **ca016_4_3_ : Distance of Another County in this Province**

Mean	Min	Max	OBS
4.0	-9,999.0	1,500.0	61

- **ca016_4_4_ : Distance of Another County in this Province**

Mean	Min	Max	OBS
137.5	-9,999.0	5,000.0	107

- **ca016_5_1_ : Province**

	No	%
01	4	6.35
03	1	1.59
05	6	9.52
06	3	4.76
07	3	4.76
10	1	1.59
11	1	1.59
12	2	3.17
13	2	3.17
14	3	4.76
16	4	6.35
17	2	3.17
18	2	3.17
20	1	1.59
21	4	6.35
24	4	6.35
25	1	1.59
26	5	7.94
27	2	3.17
29	6	9.52
32	3	4.76
33	3	4.76
Total	63	100.00

- **ca016_5_2_ : Province**

	No	%
01	5	4.76
03	2	1.90
05	10	9.52
06	9	8.57
07	2	1.90
09	2	1.90
10	3	2.86
11	5	4.76

12	7	6.67
13	1	0.95
14	6	5.71
15	1	0.95
16	6	5.71
17	1	0.95
18	1	0.95
20	2	1.90
21	4	3.81
24	9	8.57
25	1	0.95
26	4	3.81
27	3	2.86
28	1	0.95
29	11	10.48
32	5	4.76
33	3	2.86
34	1	0.95
Total	105	100.00

- **ca016_5_3_ : Province**

	No	%
01	3	4.62
03	2	3.08
05	6	9.23
06	3	4.62
07	2	3.08
08	1	1.54
09	1	1.54
10	4	6.15
11	3	4.62
12	3	4.62
13	1	1.54
14	4	6.15
15	1	1.54
17	1	1.54
18	1	1.54
21	2	3.08
24	5	7.69
25	1	1.54
26	2	3.08
27	2	3.08
28	3	4.62
29	2	3.08
32	5	7.69
33	4	6.15
34	3	4.62
Total	65	100.00

- **ca016_5_4_ : Province**

	No	%
01	2	2.00
03	1	1.00
05	10	10.00
06	9	9.00
07	2	2.00
08	2	2.00
09	4	4.00
10	2	2.00
11	5	5.00
12	2	2.00
13	3	3.00
14	9	9.00
15	3	3.00
16	1	1.00
17	3	3.00
18	1	1.00
19	1	1.00
20	3	3.00
21	3	3.00
23	1	1.00
24	5	5.00
25	1	1.00
26	3	3.00
27	1	1.00
28	5	5.00
29	6	6.00
32	7	7.00
33	3	3.00
34	2	2.00
Total	100	100.00

- **ca016_6_1_ : City**

	No	%
01	1	1.69
04	1	1.69
11	4	6.78
16	2	3.39
24	6	10.17
27	4	6.78
40	8	13.56
46	2	3.39
49	1	1.69
53	3	5.08
55	8	13.56
63	3	5.08
74	4	6.78

82	6	10.17
86	4	6.78
96	1	1.69
99	1	1.69
Total	59	100.00

- **ca016_6_2_ : City**

	No	%
01	4	4.04
04	4	4.04
11	11	11.11
16	2	2.02
18	1	1.01
24	14	14.14
27	2	2.02
38	1	1.01
40	11	11.11
46	8	8.08
49	2	2.02
53	7	7.07
55	5	5.05
60	3	3.03
63	3	3.03
74	5	5.05
82	6	6.06
86	4	4.04
96	1	1.01
99	5	5.05
Total	99	100.00

- **ca016_6_3_ : City**

	No	%
01	2	3.17
04	2	3.17
05	1	1.59
11	4	6.35
24	4	6.35
40	9	14.29
46	5	7.94
49	5	7.94
51	1	1.59
52	1	1.59
53	6	9.52
55	7	11.11
60	2	3.17
63	2	3.17
74	2	3.17

76	1	1.59
77	1	1.59
82	5	7.94
86	3	4.76
Total	63	100.00

- **ca016_6_4_ : City**

	No	%
01	4	4.12
04	2	2.06
05	2	2.06
11	6	6.19
16	3	3.09
24	7	7.22
27	1	1.03
35	1	1.03
40	22	22.68
46	6	6.19
49	6	6.19
53	5	5.15
55	8	8.25
60	4	4.12
63	5	5.15
74	3	3.09
82	7	7.22
86	2	2.06
99	3	3.09
Total	97	100.00

- **ca016_7_1_ : County**

	No	%
02	4	7.55
04	1	1.89
06	7	13.21
31	3	5.66
33	1	1.89
38	2	3.77
39	2	3.77
43	3	5.66
46	2	3.77
49	1	1.89
51	1	1.89
54	4	7.55
59	2	3.77
63	13	24.53
76	4	7.55
81	2	3.77

	No	%
92	1	1.89
Total	53	100.00

- **ca016_7_2_ : County**

	No	%
02	6	7.14
03	3	3.57
04	2	2.38
06	7	8.33
08	1	1.19
28	1	1.19
31	5	5.95
33	1	1.19
37	1	1.19
38	2	2.38
39	5	5.95
43	7	8.33
46	2	2.38
49	2	2.38
50	1	1.19
54	3	3.57
57	1	1.19
59	7	8.33
63	18	21.43
76	7	8.33
81	2	2.38
Total	84	100.00

- **ca016_7_3_ : County**

	No	%
02	5	8.77
03	1	1.75
06	6	10.53
13	1	1.75
16	1	1.75
28	3	5.26
31	2	3.51
33	1	1.75
43	3	5.26
46	2	3.51
50	1	1.75
51	2	3.51
54	4	7.02
57	1	1.75
59	6	10.53
63	12	21.05
76	5	8.77

78	1	1.75
Total	57	100.00

- **ca016_7_4_ : County**

	No	%
02	5	5.81
03	2	2.33
04	1	1.16
06	4	4.65
08	1	1.16
13	3	3.49
23	1	1.16
28	1	1.16
31	4	4.65
33	1	1.16
39	1	1.16
43	1	1.16
46	3	3.49
49	1	1.16
50	1	1.16
51	3	3.49
54	7	8.14
56	1	1.16
57	1	1.16
59	13	15.12
63	20	23.26
76	7	8.14
81	3	3.49
92	1	1.16
Total	86	100.00

- **ca016_8_1_ : Distance of Other Province**

Mean	Min	Max	OBS
578,051.0	-9,999.0	30,000,000.0	52

- **ca016_8_2_ : Distance of Other Province**

Mean	Min	Max	OBS
3,339,074.9	-9,999.0	300,000,000.0	90

- **ca016_8_3_ : Distance of Other Province**

Mean	Min	Max	OBS

5,715,593.1	-9,999.0	300,000,000.0	56

- **ca016_8_4_ : Distance of Other Province**

Mean	Min	Max	OBS
9,252.5	-9,999.0	500,000.0	86

- **ca017_1_ : What Kind of Location does Your Parent Live in**

	No	%
1 City	119	15.34
2 County	69	8.89
3 Town	42	5.41
4 Village	546	70.36
Total	776	100.00

- **ca017_2_ : What Kind of Location does Your Parent Live in**

	No	%
1 City	218	16.99
2 County	96	7.48
3 Town	61	4.75
4 Village	908	70.77
Total	1,283	100.00

- **ca017_3_ : What Kind of Location does Your Parent Live in**

	No	%
1 City	103	14.91
2 County	50	7.24
3 Town	43	6.22
4 Village	495	71.64
Total	691	100.00

- **ca017_4_ : What Kind of Location does Your Parent Live in**

	No	%
1 City	183	14.70
2 County	107	8.59
3 Town	57	4.58
4 Village	898	72.13
Total	1,245	100.00

- **ca018_1_ : Is His Hukou in the Same Place as His Current Residence**

	No	%
1 Yes	1,247	93.06
2 No	91	6.79
3 Do not have Hukou	2	0.15
Total	1,340	100.00

- **ca018_2_ : Is His Hukou in the Same Place as His Current Residence**

	No	%
1 Yes	2,143	92.01
2 No	181	7.77
3 Do not have Hukou	5	0.21
Total	2,329	100.00

- **ca018_3_ : Is His Hukou in the Same Place as His Current Residence**

	No	%
1 Yes	1,225	93.01
2 No	90	6.83
3 Do not have Hukou	2	0.15
Total	1,317	100.00

- **ca018_4_ : Is His Hukou in the Same Place as His Current Residence**

	No	%
1 Yes	2,113	92.19
2 No	174	7.59
3 Do not have Hukou	5	0.22
Total	2,292	100.00

- **ca019_1_ : Current Hukou Location of Your Parent**

	No	%
1 This Village/Neighborhood	17	18.89
2 Another Village/Neighborhood in this County/City	58	64.44
3 Another County/City in this Province	9	10.00
4 Another province	6	6.67
Total	90	100.00

- **ca019_1_1_ : City**

	No	%
04	1	11.11
11	1	11.11
40	1	11.11
46	3	33.33
55	1	11.11
74	1	11.11
82	1	11.11
Total	9	100.00

- **ca019_1_2_ : City**

	No	%
01	1	5.26
04	1	5.26
11	2	10.53
24	2	10.53
40	3	15.79
46	2	10.53
49	1	5.26
51	1	5.26
53	1	5.26
55	1	5.26
56	1	5.26
60	2	10.53
82	1	5.26
Total	19	100.00

- **ca019_1_3_ : City**

	No	%
04	1	9.09
11	2	18.18
24	2	18.18
40	2	18.18
46	2	18.18
63	1	9.09
82	1	9.09
Total	11	100.00

- **ca019_1_4_ : City**

	No	%
11	1	7.14
24	1	7.14

40	3	21.43
46	3	21.43
49	1	7.14
53	1	7.14
63	2	14.29
82	1	7.14
86	1	7.14
Total	14	100.00

- **ca019_2_ : Current Hukou Location of Your Parent**

	No	%
1 This Village/Neighborhood	66	35.29
2 Another Village/Neighborhood in this County/City	94	50.27
3 Another County/City in this Province	18	9.63
4 Another province	8	4.28
5 Abroad	1	0.53
Total	187	100.00

- **ca019_2_1_ : County**

	No	%
04	2	25.00
31	2	25.00
63	2	25.00
76	2	25.00
Total	8	100.00

- **ca019_2_2_ : County**

	No	%
02	1	6.25
03	1	6.25
04	1	6.25
06	1	6.25
08	1	6.25
28	1	6.25
31	2	12.50
43	1	6.25
44	1	6.25
54	1	6.25
59	1	6.25
63	2	12.50
76	2	12.50
Total	16	100.00

- **ca019_2_3_ : County**

	No	%
31	1	9.09
46	2	18.18
56	1	9.09
59	3	27.27
63	3	27.27
76	1	9.09
Total	11	100.00

- **ca019_2_4_ : County**

	No	%
06	1	7.14
08	1	7.14
28	1	7.14
31	1	7.14
43	2	14.29
46	1	7.14
51	1	7.14
59	2	14.29
63	2	14.29
78	1	7.14
81	1	7.14
Total	14	100.00

- **ca019_3_ : Current Hukou Location of Your Parent**

	No	%
1 This Village/Neighborhood	20	21.74
2 Another Village/Neighborhood in this County/City	55	59.78
3 Another County/City in this Province	11	11.96
4 Another province	6	6.52
Total	92	100.00

- **ca019_3_1_ : Province**

	No	%
05	1	16.67
06	1	16.67
10	2	33.33
19	1	16.67
21	1	16.67
Total	6	100.00

- **ca019_3_2_ : Province**

	No	%
05	2	25.00
06	1	12.50
14	1	12.50
19	1	12.50
21	1	12.50
24	1	12.50
27	1	12.50
Total	8	100.00

- **ca019_3_3_ : Province**

	No	%
11	1	16.67
12	1	16.67
16	1	16.67
18	1	16.67
26	1	16.67
33	1	16.67
Total	6	100.00

- **ca019_3_4_ : Province**

	No	%
06	1	14.29
08	1	14.29
11	1	14.29
16	1	14.29
26	1	14.29
32	1	14.29
34	1	14.29
Total	7	100.00

- **ca019_4_ : Current Hukou Location of Your Parent**

	No	%
1 This Village/Neighborhood	62	35.43
2 Another Village/Neighborhood in this County/City	91	52.00
3 Another County/City in this Province	15	8.57
4 Another province	7	4.00
Total	175	100.00

- **ca019_4_1_ : City**

	No	%
24	1	16.67
40	4	66.67
82	1	16.67
Total	6	100.00

- **ca019_4_2_ : City**

	No	%
04	2	25.00
40	4	50.00
46	1	12.50
53	1	12.50
Total	8	100.00

- **ca019_4_3_ : City**

	No	%
24	1	20.00
49	1	20.00
53	1	20.00
55	1	20.00
60	1	20.00
Total	5	100.00

- **ca019_4_4_ : City**

	No	%
05	1	14.29
11	1	14.29
51	1	14.29
53	3	42.86
74	1	14.29
Total	7	100.00

- **ca019_5_1_ : County**

	No	%
16	2	50.00
45	1	25.00
63	1	25.00
Total	4	100.00

- **ca019_5_2_ : County**

	No	%
16	1	16.67
38	1	16.67
46	1	16.67
58	1	16.67
63	1	16.67
76	1	16.67
Total	6	100.00

- **ca019_5_3_ : County**

	No	%
06	1	20.00
31	1	20.00
49	1	20.00
63	2	40.00
Total	5	100.00

- **ca019_5_4_ : County**

	No	%
43	1	16.67
63	5	83.33
Total	6	100.00

- **ca020_1_ : Current Hukou Status of Your Parent**

	No	%
1 Agriculture Hukou	1,035	77.53
2 Non-Agriculture Hukou	294	22.02
3 Unified Residency Hukou	4	0.30
4 Do not have Hukou	2	0.15
Total	1,335	100.00

- **ca020_2_ : Current Hukou Status of Your Parent**

	No	%
1 Agriculture Hukou	1,922	82.74
2 Non-Agriculture Hukou	388	16.70
3 Unified Residency Hukou	11	0.47
4 Do not have Hukou	2	0.09
Total	2,323	100.00

- **ca020_3_ : Current Hukou Status of Your Parent**

	No	%
1 Agriculture Hukou	1,041	79.53
2 Non-Agriculture Hukou	263	20.09
3 Unified Residency Hukou	4	0.31
4 Do not have Hukou	1	0.08
Total	1,309	100.00

- **ca020_4_ : Current Hukou Status of Your Parent**

	No	%
1 Agriculture Hukou	1,905	83.37
2 Non-Agriculture Hukou	371	16.24
3 Unified Residency Hukou	9	0.39
Total	2,285	100.00

- **ca021_1_ : Does Your Parent Own House**

	No	%
1 Yes	570	42.73
2 No	764	57.27
Total	1,334	100.00

- **ca021_2_ : Does Your Parent Own House**

	No	%
1 Yes	582	25.06
2 No	1,740	74.94
Total	2,322	100.00

- **ca021_3_ : Does Your Parent Own House**

	No	%
1 Yes	574	43.82
2 No	736	56.18
Total	1,310	100.00

- **ca021_4_ : Does Your Parent Own House**

	No	%
1 Yes	554	24.29

2 No	1,727	75.71
Total	2,281	100.00

- **ca022_1_ : The Present Value of Parent' House**

Mean	Min	Max	OBS
699.8	0.0	50,000.0	486

- **ca022_2_ : The Present Value of Parent' House**

Mean	Min	Max	OBS
717.9	0.0	150,000.0	483

- **ca022_3_ : The Present Value of Parent' House**

Mean	Min	Max	OBS
12,924.1	0.0	3,600,000.0	487

- **ca022_4_ : The Present Value of Parent' House**

Mean	Min	Max	OBS
6,958.3	0.0	2,000,000.0	464

- **ca022_a_1_ : Does Your Parent Share House with Others**

	No	%
1 Yes	201	45.68
2 No	239	54.32
Total	440	100.00

- **ca022_a_2_ : Does Your Parent Share House with Others**

	No	%
1 Yes	235	51.65
2 No	220	48.35
Total	455	100.00

- **ca022_a_3_ : Does Your Parent Share House with Others**

	No	%
1 Yes	224	50.00
2 No	224	50.00
Total	448	100.00

- **ca022_a_4_ : Does Your Parent Share House with Others**

	No	%
1 Yes	225	52.20
2 No	206	47.80
Total	431	100.00

- **ca023 : Who Answer Questions about the Spouse's Parents**

	No	%
1 The family respondent	5,297	67.17
2 The spouse	2,589	32.83
Total	7,886	100.00

- **ca024 : Do You Keep in Contact with Your Spouse's Parents**

	No	%
1 Yes	148	7.93
2 No	1,719	92.07
Total	1,867	100.00

- **cb001 : How Many Biological Children**

	No	%
0	502	22.33
1	317	14.10
2	396	17.62
3	373	16.59
4	307	13.66
5	200	8.90
6	93	4.14
7	46	2.05
8	10	0.44
9	4	0.18
Total	2,248	100.00

- **cb003 : Num. of Dead Children**

	No	%
0	1,822	81.38
1	301	13.44
2	72	3.22
3	21	0.94
4	17	0.76
5	4	0.18
6	2	0.09
Total	2,239	100.00

- **cb004_1_1_ : Year this Child Born**

Mean	Min	Max	OBS
1,963.4	1,900.0	2,010.0	307

- **cb004_1_2_ : Year this Child Born**

Mean	Min	Max	OBS
1,962.8	1,943.0	1,990.0	59

- **cb004_1_3_ : Year this Child Born**

Mean	Min	Max	OBS
1,966.6	1,952.0	1,987.0	17

- **cb004_1_4_ : Year this Child Born**

Mean	Min	Max	OBS
1,963.4	1,953.0	1,978.0	8

- **cb004_1_5_ : Year this Child Born**

Mean	Min	Max	OBS
1,966.0	1,965.0	1,967.0	2

- **cb004_1_6_ : Year this Child Born**

No Observations

- **cb004_2_1_ : Month this Child Born**

	No	%
0	87	28.43
1	19	6.21
2	20	6.54
3	18	5.88
4	18	5.88
5	19	6.21
6	15	4.90
7	16	5.23
8	18	5.88
9	18	5.88
10	12	3.92
11	20	6.54
12	26	8.50
Total	306	100.00

- **cb004_2_2_ : Month this Child Born**

	No	%
0	20	33.90
1	2	3.39
2	3	5.08
3	6	10.17
4	3	5.08
5	5	8.47
6	2	3.39
7	4	6.78
8	4	6.78
9	1	1.69
10	2	3.39
11	3	5.08
12	4	6.78
Total	59	100.00

- **cb004_2_3_ : Month this Child Born**

	No	%
0	4	23.53
1	2	11.76
3	1	5.88
4	1	5.88
5	2	11.76
6	1	5.88
7	1	5.88
8	1	5.88
9	1	5.88

11	2	11.76
12	1	5.88
Total	17	100.00

- **cb004_2_4_ : Month this Child Born**

	No	%
0	2	25.00
2	1	12.50
5	1	12.50
6	1	12.50
8	1	12.50
10	1	12.50
12	1	12.50
Total	8	100.00

- **cb004_2_5_ : Month this Child Born**

	No	%
0	1	50.00
6	1	50.00
Total	2	100.00

- **cb004_2_6_ : Month this Child Born**

No Observations

- **cb005_1_ : Solar or Lunar Calendar**

	No	%
1 Solar calendar	49	16.12
2 Lunar calendar	255	83.88
Total	304	100.00

- **cb005_2_ : Solar or Lunar Calendar**

	No	%
1 Solar calendar	11	18.97
2 Lunar calendar	47	81.03
Total	58	100.00

- **cb005_3_ : Solar or Lunar Calendar**

	No	%
1 Solar calendar	4	25.00
2 Lunar calendar	12	75.00
Total	16	100.00

- **cb005_4_ : Solar or Lunar Calendar**

	No	%
1 Solar calendar	2	25.00
2 Lunar calendar	6	75.00
Total	8	100.00

- **cb005_5_ : Solar or Lunar Calendar**

	No	%
1 Solar calendar	1	50.00
2 Lunar calendar	1	50.00
Total	2	100.00

- **cb006_1_ : Gender of this Child**

	No	%
1 Male	258	63.55
2 Female	148	36.45
Total	406	100.00

- **cb006_2_ : Gender of this Child**

	No	%
1 Male	56	50.00
2 Female	56	50.00
Total	112	100.00

- **cb006_3_ : Gender of this Child**

	No	%
1 Male	26	63.41
2 Female	15	36.59
Total	41	100.00

- **cb006_4_ : Gender of this Child**

	No	%
1 Male	12	52.17
2 Female	11	47.83
Total	23	100.00

- **cb006_5_ : Gender of this Child**

	No	%
1 Male	4	66.67
2 Female	2	33.33
Total	6	100.00

- **cb006_6_ : Gender of this Child**

	No	%
1 Male	1	50.00
2 Female	1	50.00
Total	2	100.00

- **cb007_1_1_ : Year this Child Passed Away**

Mean	Min	Max	OBS
1,987.1	1,900.0	2,019.0	315

- **cb007_1_2_ : Year this Child Passed Away**

Mean	Min	Max	OBS
1,981.8	1,950.0	2,010.0	62

- **cb007_1_3_ : Year this Child Passed Away**

Mean	Min	Max	OBS
1,979.3	1,957.0	2,011.0	18

- **cb007_1_4_ : Year this Child Passed Away**

Mean	Min	Max	OBS
1,972.6	1,960.0	1,996.0	9

- **cb007_1_5_ : Year this Child Passed Away**

Mean	Min	Max	OBS
1,976.3	1,965.0	1,995.0	3

- **cb007_1_6_ : Year this Child Passed Away**

No Observations

- **cb007_2_1_ : Month this Child Passed Away**

	No	%
0	100	31.75
1	14	4.44
2	13	4.13
3	19	6.03
4	16	5.08
5	35	11.11
6	18	5.71
7	17	5.40
8	20	6.35
9	21	6.67
10	15	4.76
11	9	2.86
12	18	5.71
Total	315	100.00

- **cb007_2_2_ : Month this Child Passed Away**

	No	%
0	24	38.71
1	5	8.06
2	3	4.84
3	4	6.45
4	1	1.61
5	4	6.45
6	3	4.84
7	6	9.68
8	6	9.68
9	1	1.61
10	3	4.84
11	2	3.23
Total	62	100.00

- **cb007_2_3_ : Month this Child Passed Away**

	No	%
0	6	33.33
2	1	5.56
3	3	16.67
4	1	5.56
5	1	5.56
7	3	16.67
11	1	5.56
12	2	11.11
Total	18	100.00

- **cb007_2_4_ : Month this Child Passed Away**

	No	%
0	2	22.22
2	2	22.22
6	1	11.11
8	1	11.11
9	1	11.11
10	2	22.22
Total	9	100.00

- **cb007_2_5_ : Month this Child Passed Away**

	No	%
0	1	33.33
1	1	33.33
2	1	33.33
Total	3	100.00

- **cb007_2_6_ : Month this Child Passed Away**

No Observations

- **cb008_1_ : Solar or Lunar Calendar**

	No	%
1 Solar calendar	76	24.36
2 Lunar calendar	236	75.64
Total	312	100.00

- **cb008_2_ : Solar or Lunar Calendar**

	No	%
1 Solar calendar	12	19.67
2 Lunar calendar	49	80.33
Total	61	100.00

- **cb008_3_ : Solar or Lunar Calendar**

	No	%
1 Solar calendar	4	23.53
2 Lunar calendar	13	76.47
Total	17	100.00

- **cb008_4_ : Solar or Lunar Calendar**

	No	%
1 Solar calendar	2	22.22
2 Lunar calendar	7	77.78
Total	9	100.00

- **cb008_5_ : Solar or Lunar Calendar**

	No	%
1 Solar calendar	1	33.33
2 Lunar calendar	2	66.67
Total	3	100.00

- **cb009 : Num. of Adopted or Fostered Children Currently Living but Not Living with You**

	No	%
0	2,191	97.81
1	42	1.88
2	5	0.22
3	2	0.09
Total	2,240	100.00

- **cb011 : Num. of Passed Away Adopted or Step Children**

	No	%
0	2,236	99.73

1	5	0.22
2	1	0.04
Total	2,242	100.00

- **cb012_1_1_ : Year this Child Was Born**

Mean	Min	Max	OBS
1,955.0	1,945.0	1,962.0	4

- **cb012_1_2_ : Year this Child Was Born**

Mean	Min	Max	OBS
1,941.0	1,941.0	1,941.0	1

- **cb012_2_1_ : Month this Child Was Born**

	No	%
1	2	66.67
7	1	33.33
Total	3	100.00

- **cb012_2_2_ : Month this Child Was Born**

	No	%
1	1	100.00
Total	1	100.00

- **cb013_1_ : Solar or Lunar Calendar**

	No	%
2 Lunar calendar	3	100.00
Total	3	100.00

- **cb013_2_ : Solar or Lunar Calendar**

	No	%
2 Lunar calendar	1	100.00
Total	1	100.00

- **cb014_1_ : Gender of this Child**

	No	%
1 Male	3	60.00
2 Female	2	40.00
Total	5	100.00

- **cb014_2_ : Gender of this Child**

	No	%
2 Female	1	100.00
Total	1	100.00

- **cb015_1_1_ : Year this Child Passed Away**

Mean	Min	Max	OBS
2,005.0	1,998.0	2,011.0	3

- **cb015_1_2_ : Year this Child Passed Away**

Mean	Min	Max	OBS
1,999.0	1,999.0	1,999.0	1

- **cb015_2_1_ : Month this Child Passed Away**

	No	%
2	1	33.33
9	2	66.67
Total	3	100.00

- **cb015_2_2_ : Month this Child Passed Away**

	No	%
1	1	100.00
Total	1	100.00

- **cb016_1_ : Solar or Lunar Calendar**

	No	%
2 Lunar calendar	3	100.00
Total	3	100.00

- **cb016_2_ : Solar or Lunar Calendar**

	No	%
2 Lunar calendar	1	100.00
Total	1	100.00

- **cb017 : Num. of Biological Children with Your Current Spouse Who are Currently Living bu**

	No	%
0	2,322	29.31
1	1,837	23.19
2	1,695	21.40
3	1,064	13.43
4	549	6.93
5	299	3.77
6	104	1.31
7	39	0.49
8	11	0.14
9	1	0.01
Total	7,921	100.00

- **cb019 : Num. of Dead Biological Children**

	No	%
0	7,370	93.07
1	454	5.73
2	71	0.90
3	15	0.19
4	6	0.08
5	3	0.04
Total	7,919	100.00

- **cb020_1_1_ : Year this Child Was Born**

Mean	Min	Max	OBS
1,972.5	1,900.0	2,004.0	457

- **cb020_1_2_ : Year this Child Was Born**

Mean	Min	Max	OBS
1,971.3	1,949.0	1,992.0	61

- **cb020_1_3_ : Year this Child Was Born**

Mean	Min	Max	OBS
1,974.3	1,956.0	1,992.0	15

- **cb020_1_4_ : Year this Child Was Born**

Mean	Min	Max	OBS
1,969.8	1,956.0	1,984.0	6

- **cb020_1_5_ : Year this Child Was Born**

Mean	Min	Max	OBS
1,968.0	1,957.0	1,985.0	3

- **cb020_2_1_ : Month this Child was Born**

	No	%
0	89	19.43
1	38	8.30
2	29	6.33
3	19	4.15
4	22	4.80
5	20	4.37
6	30	6.55
7	38	8.30
8	36	7.86
9	28	6.11
10	37	8.08
11	33	7.21
12	39	8.52
Total	458	100.00

- **cb020_2_2_ : Month this Child was Born**

	No	%
0	16	26.23
1	6	9.84
2	5	8.20
3	4	6.56
4	2	3.28
5	2	3.28
6	1	1.64
7	5	8.20

	No	%
8	7	11.48
9	2	3.28
10	5	8.20
11	2	3.28
12	4	6.56
Total	61	100.00

- **cb020_2_3_ : Month this Child was Born**

	No	%
0	7	46.67
1	1	6.67
2	1	6.67
4	1	6.67
5	1	6.67
7	1	6.67
8	1	6.67
9	1	6.67
11	1	6.67
Total	15	100.00

- **cb020_2_4_ : Month this Child was Born**

	No	%
0	2	33.33
6	1	16.67
8	1	16.67
9	1	16.67
12	1	16.67
Total	6	100.00

- **cb020_2_5_ : Month this Child was Born**

	No	%
0	2	66.67
9	1	33.33
Total	3	100.00

- **cb021_1_ : Solar or Lunar Calendar**

	No	%
1 Solar calenda	58	12.80
2 Lunar calendar	395	87.20
Total	453	100.00

- **cb021_2_ : Solar or Lunar Calendar**

	No	%
1 Solar calenda	8	13.11
2 Lunar calendar	53	86.89
Total	61	100.00

- **cb021_3_ : Solar or Lunar Calendar**

	No	%
1 Solar calenda	2	13.33
2 Lunar calendar	13	86.67
Total	15	100.00

- **cb021_4_ : Solar or Lunar Calendar**

	No	%
1 Solar calenda	1	16.67
2 Lunar calendar	5	83.33
Total	6	100.00

- **cb021_5_ : Solar or Lunar Calendar**

	No	%
1 Solar calenda	1	33.33
2 Lunar calendar	2	66.67
Total	3	100.00

- **cb022_1_ : Gender of this Child**

	No	%
1 Male	341	61.66
2 Female	212	38.34
Total	553	100.00

- **cb022_2_ : Gender of this Child**

	No	%
1 Male	48	51.61
2 Female	45	48.39
Total	93	100.00

- **cb022_3_ : Gender of this Child**

	No	%
1 Male	13	56.52
2 Female	10	43.48
Total	23	100.00

- **cb022_4_ : Gender of this Child**

	No	%
1 Male	5	55.56
2 Female	4	44.44
Total	9	100.00

- **cb022_5_ : Gender of this Child**

	No	%
1 Male	1	33.33
2 Female	2	66.67
Total	3	100.00

- **cb023_1_1_ : Year this Child Passed Away**

Mean	Min	Max	OBS
1,987.5	1,900.0	2,011.0	460

- **cb023_1_2_ : Year this Child Passed Away**

Mean	Min	Max	OBS
1,981.3	1,954.0	2,009.0	61

- **cb023_1_3_ : Year this Child Passed Away**

Mean	Min	Max	OBS
1,982.0	1,956.0	2,009.0	15

- **cb023_1_4_ : Year this Child Passed Away**

Mean	Min	Max	OBS
1,977.4	1,957.0	2,003.0	7

- **cb023_1_5_ : Year this Child Passed Away**

Mean	Min	Max	OBS
1,971.3	1,962.0	1,985.0	3

- **cb023_2_1_ : Month this Child Passed Away**

	No	%
0	98	21.30
1	32	6.96
2	25	5.43
3	32	6.96
4	33	7.17
5	35	7.61
6	40	8.70
7	30	6.52
8	37	8.04
9	25	5.43
10	28	6.09
11	20	4.35
12	25	5.43
Total	460	100.00

- **cb023_2_2_ : Month this Child Passed Away**

	No	%
0	20	32.79
1	6	9.84
2	4	6.56
3	2	3.28
4	5	8.20
5	3	4.92
7	4	6.56
8	5	8.20
9	4	6.56
10	3	4.92
11	4	6.56
12	1	1.64
Total	61	100.00

- **cb023_2_3_ : Month this Child Passed Away**

	No	%
0	6	40.00
4	1	6.67
5	2	13.33

6	2	13.33
7	1	6.67
8	1	6.67
9	1	6.67
12	1	6.67
Total	15	100.00

- **cb023_2_4_ : Month this Child Passed Away**

	No	%
0	2	28.57
6	2	28.57
8	1	14.29
9	1	14.29
12	1	14.29
Total	7	100.00

- **cb023_2_5_ : Month this Child Passed Away**

	No	%
0	3	100.00
Total	3	100.00

- **cb024_1_ : Solar or Lunar Calendar**

	No	%
1 Solar calendar	73	15.94
2 Lunar calendar	385	84.06
Total	458	100.00

- **cb024_2_ : Solar or Lunar Calendar**

	No	%
1 Solar calendar	7	11.48
2 Lunar calendar	54	88.52
Total	61	100.00

- **cb024_3_ : Solar or Lunar Calendar**

	No	%
1 Solar calendar	2	13.33
2 Lunar calendar	13	86.67
Total	15	100.00

- **cb024_4_ : Solar or Lunar Calendar**

	No	%
1 Solar calendar	1	14.29
2 Lunar calendar	6	85.71
Total	7	100.00

- **cb024_5_ : Solar or Lunar Calendar**

	No	%
1 Solar calendar	1	33.33
2 Lunar calendar	2	66.67
Total	3	100.00

- **cb025 : Num. of Additional Biological Children Currently Living but Not Living with You**

	No	%
0	451	74.67
1	67	11.09
2	38	6.29
3	31	5.13
4	11	1.82
5	4	0.66
6	2	0.33
Total	604	100.00

- **cb027 : Num. of Additional Biological Dead Children**

	No	%
0	578	95.70
1	22	3.64
2	3	0.50
3	1	0.17
Total	604	100.00

- **cb028_1_1_ : Year this Child Was Born**

Mean	Min	Max	OBS
1,968.5	1,946.0	1,988.0	20

- **cb028_1_2_ : Year this Child Was Born**

Mean	Min	Max	OBS
1,977.5	1,976.0	1,979.0	2

- **cb028_1_3_ : Year this Child Was Born**

No Observations

- **cb028_2_1_ : Month this Child Was Born**

	No	%
1	4	20.00
2	1	5.00
3	1	5.00
4	1	5.00
6	2	10.00
7	3	15.00
8	2	10.00
9	3	15.00
10	1	5.00
11	1	5.00
12	1	5.00
Total	20	100.00

- **cb028_2_2_ : Month this Child Was Born**

	No	%
9	1	50.00
12	1	50.00
Total	2	100.00

- **cb028_2_3_ : Month this Child Was Born**

No Observations

- **cb029_1_ : Solar or Lunar Calendar**

	No	%
1 Solar calendar	1	5.00
2 Lunar calendar	19	95.00

Total	20	100.00

- **cb029_2_ : Solar or Lunar Calendar**

	No	%
2 Lunar calendar	3	100.00
Total	3	100.00

- **cb030_1_ : Gender of this Child**

	No	%
1 Male	12	48.00
2 Female	13	52.00
Total	25	100.00

- **cb030_2_ : Gender of this Child**

	No	%
1 Male	2	50.00
2 Female	2	50.00
Total	4	100.00

- **cb030_3_ : Gender of this Child**

	No	%
2 Female	1	100.00
Total	1	100.00

- **cb031_1_1_ : Year this Child Passed Away**

Mean	Min	Max	OBS
1,986.7	1,959.0	2,011.0	18

- **cb031_1_2_ : Year this Child Passed Away**

Mean	Min	Max	OBS
1,983.5	1,983.0	1,984.0	2

- **cb031_1_3_ : Year this Child Passed Away**

No Observations

- **cb031_2_1_ : Month this Child Passed Away**

	No	%
1	1	5.56
2	4	22.22
5	3	16.67
6	1	5.56
7	3	16.67
8	1	5.56
9	1	5.56
10	2	11.11
12	2	11.11
Total	18	100.00

- **cb031_2_2_ : Month this Child Passed Away**

	No	%
7	1	50.00
11	1	50.00
Total	2	100.00

- **cb031_2_3_ : Month this Child Passed Away**

No Observations

- **cb032_1_ : Solar or Lunar Calendar**

	No	%
1 Solar calendar	2	10.53
2 Lunar calendar	17	89.47
Total	19	100.00

- **cb032_2_ : Solar or Lunar Calendar**

	No	%
2 Lunar calendar	2	100.00
Total	2	100.00

- **cb033 : Num. of Your Spouse's Additional Biological Children Currently Living but not Li**

	No	%
0	7,735	97.69
1	74	0.93
2	56	0.71
3	27	0.34
4	16	0.20
5	3	0.04
6	6	0.08
7	1	0.01
Total	7,918	100.00

- **cb035 : Num. of Spouse's Additional Biological Dead Children**

	No	%
0	7,896	99.73
1	17	0.21
2	2	0.03
4	1	0.01
5	1	0.01
Total	7,917	100.00

- **cb036_1_1_ : Year this Child Born**

Mean	Min	Max	OBS
1,981.0	1,964.0	1,990.0	12

- **cb036_1_2_ : Year this Child Born**

Mean	Min	Max	OBS
1,976.5	1,965.0	1,988.0	2

- **cb036_1_3_ : Year this Child Born**

Mean	Min	Max	OBS
1,981.0	1,973.0	1,989.0	2

- **cb036_1_4_ : Year this Child Born**

Mean	Min	Max	OBS

1,982.0	1,974.0	1,990.0	2

- **cb036_1_5_ : Year this Child Born**

Mean	Min	Max	OBS
1,977.0	1,977.0	1,977.0	1

- **cb036_2_1_ : Month this Child Born**

	No	%
3	1	9.09
4	1	9.09
7	1	9.09
8	2	18.18
9	2	18.18
10	2	18.18
12	2	18.18
Total	11	100.00

- **cb036_2_2_ : Month this Child Born**

	No	%
4	1	50.00
11	1	50.00
Total	2	100.00

- **cb036_2_3_ : Month this Child Born**

	No	%
1	1	50.00
4	1	50.00
Total	2	100.00

- **cb036_2_4_ : Month this Child Born**

	No	%
4	1	50.00
12	1	50.00
Total	2	100.00

- **cb036_2_5_ : Month this Child Born**

	No	%
10	1	100.00
Total	1	100.00

- **cb037_1_ : Solar or Lunar Calendar**

	No	%
1 Solar calendar	4	36.36
2 Lunar calendar	7	63.64
Total	11	100.00

- **cb037_2_ : Solar or Lunar Calendar**

	No	%
2 Lunar calendar	2	100.00
Total	2	100.00

- **cb037_3_ : Solar or Lunar Calendar**

	No	%
2 Lunar calendar	2	100.00
Total	2	100.00

- **cb037_4_ : Solar or Lunar Calendar**

	No	%
2 Lunar calendar	2	100.00
Total	2	100.00

- **cb037_5_ : Solar or Lunar Calendar**

	No	%
2 Lunar calendar	1	100.00
Total	1	100.00

- **cb038_1_ : Gender of this Child**

	No	%
1 Male	11	47.83

2 Female	12	52.17
Total	23	100.00

- **cb038_2_ : Gender of this Child**

	No	%
1 Male	3	75.00
2 Female	1	25.00
Total	4	100.00

- **cb038_3_ : Gender of this Child**

	No	%
1 Male	1	50.00
2 Female	1	50.00
Total	2	100.00

- **cb038_4_ : Gender of this Child**

	No	%
2 Female	2	100.00
Total	2	100.00

- **cb038_5_ : Gender of this Child**

	No	%
1 Male	1	100.00
Total	1	100.00

- **cb039_1_1_ : Year this Child Passed Away**

Mean	Min	Max	OBS
1,986.3	1,964.0	2,004.0	10

- **cb039_1_2_ : Year this Child Passed Away**

Mean	Min	Max	OBS
1,976.5	1,965.0	1,988.0	2

- **cb039_1_3_ : Year this Child Passed Away**

Mean	Min	Max	OBS
1,981.0	1,973.0	1,989.0	2

- **cb039_1_4_ : Year this Child Passed Away**

Mean	Min	Max	OBS
1,982.0	1,974.0	1,990.0	2

- **cb039_1_5_ : Year this Child Passed Away**

Mean	Min	Max	OBS
1,977.0	1,977.0	1,977.0	1

- **cb039_2_1_ : Month this Child Passed Away**

	No	%
1	1	10.00
2	1	10.00
5	2	20.00
6	1	10.00
7	1	10.00
9	1	10.00
12	3	30.00
Total	10	100.00

- **cb039_2_2_ : Month this Child Passed Away**

	No	%
4	1	50.00
11	1	50.00
Total	2	100.00

- **cb039_2_3_ : Month this Child Passed Away**

	No	%
1	1	50.00
4	1	50.00
Total	2	100.00

- **cb039_2_4_ : Month this Child Passed Away**

	No	%
5	1	50.00
12	1	50.00
Total	2	100.00

- **cb039_2_5_ : Month this Child Passed Away**

	No	%
10	1	100.00
Total	1	100.00

- **cb040_1_ : Solar or Lunar Calendar**

	No	%
1 Solar calendar	4	40.00
2 Lunar calendar	6	60.00
Total	10	100.00

- **cb040_2_ : Solar or Lunar Calendar**

	No	%
2 Lunar calendar	2	100.00
Total	2	100.00

- **cb040_3_ : Solar or Lunar Calendar**

	No	%
2 Lunar calendar	2	100.00
Total	2	100.00

- **cb040_4_ : Solar or Lunar Calendar**

	No	%
2 Lunar calendar	2	100.00
Total	2	100.00

- **cb040_5_ : Solar or Lunar Calendar**

	No	%

2 Lunar calendar	1	100.00
Total	1	100.00

- **cb041 : Num. of Adopted or Foster Children Currently Living but Not Living with You**

	No	%
0	7,824	98.79
1	88	1.11
2	6	0.08
3	2	0.03
Total	7,920	100.00

- **cb043 : Num. of Adopted or Forster Children Who are Died**

	No	%
0	7,913	99.91
1	6	0.08
2	1	0.01
Total	7,920	100.00

- **cb044_1_1_ : Year this Child Was Born**

Mean	Min	Max	OBS
1,988.0	1,980.0	1,994.0	3

- **cb044_1_2_ : Year this Child Was Born**

No Observations

- **cb044_2_1_ : Month this Child Was Born**

	No	%
2	1	33.33
5	1	33.33
8	1	33.33
Total	3	100.00

- **cb044_2_2_ : Month this Child Was Born**

No Observations

- **cb045_1_ : Solar or Lunar Calendar**

	No	%
2 Lunar calendar	3	100.00
Total	3	100.00

- **cb046_1_ : Gender of this Child**

	No	%
2 Female	6	100.00
Total	6	100.00

- **cb046_2_ : Gender of this Child**

	No	%
2 Female	1	100.00
Total	1	100.00

- **cb047_1_1_ : Year this Child Passed Away**

Mean	Min	Max	OBS
2,004.8	1,997.0	2,011.0	4

- **cb047_1_2_ : Year this Child Passed Away**

No Observations

- **cb047_2_1_ : Month this Child Passed Away**

	No	%
1	1	25.00
3	1	25.00
5	1	25.00
8	1	25.00
Total	4	100.00

- **cb047_2_2_ : Month this Child Passed Away**

No Observations

- **cb048_1_ : Solar or Lunar Calendar**

	No	%
1 Solar calendar	1	25.00
2 Lunar calendar	3	75.00
Total	4	100.00

- **cb049_1_ : Gender of this Child**

	No	%
1 Boy Son	2,559	61.75
2 Girl Daughter	1,585	38.25
Total	4,144	100.00

- **cb049_2_ : Gender of this Child**

	No	%
1 Boy Son	3,171	50.10
2 Girl Daughter	3,158	49.90
Total	6,329	100.00

- **cb049_3_ : Gender of this Child**

	No	%
1 Boy Son	1,761	40.19
2 Girl Daughter	2,621	59.81
Total	4,382	100.00

- **cb049_4_ : Gender of this Child**

	No	%
1 Boy Son	829	33.62
2 Girl Daughter	1,637	66.38
Total	2,466	100.00

- **cb049_5_ : Gender of this Child**

	No	%
1 Boy Son	329	27.21
2 Girl Daughter	880	72.79
Total	1,209	100.00

- **cb049_6_ : Gender of this Child**

	No	%
1 Boy Son	129	25.70
2 Girl Daughter	373	74.30
Total	502	100.00

- **cb049_7_ : Gender of this Child**

	No	%
1 Boy Son	56	29.79
2 Girl Daughter	132	70.21
Total	188	100.00

- **cb049_8_ : Gender of this Child**

	No	%
1 Boy Son	17	26.98
2 Girl Daughter	46	73.02
Total	63	100.00

- **cb049_9_ : Gender of this Child**

	No	%
1 Boy Son	6	27.27
2 Girl Daughter	16	72.73
Total	22	100.00

- **cb049_10_ : Gender of this Child**

	No	%
1 Boy Son	3	50.00
2 Girl Daughter	3	50.00
Total	6	100.00

- **cb049_11_ : Gender of this Child**

No Observations

- **cb049_12_ : Gender of this Child**

No Observations

- **cb049_13_ : Gender of this Child**

No Observations

- **cb049_14_ : Gender of this Child**

No Observations

- **cb050_1_ : Chinese Zodiac**

	No	%
1 Rat	285	7.04
2 Ox	309	7.63
3 Tiger	361	8.92
4 Rabbit	357	8.82
5 Dragon	361	8.92
6 Snake	321	7.93
7 Horse	316	7.80
8 Goat	304	7.51
9 Monkey	332	8.20
10 Rooster	346	8.55
11 Dog	401	9.90
12 Pig	356	8.79
Total	4,049	100.00

- **cb050_2_ : Chinese Zodiac**

	No	%
1 Rat	482	7.83
2 Ox	483	7.85

3 Tiger	576	9.36
4 Rabbit	584	9.49
5 Dragon	529	8.60
6 Snake	447	7.27
7 Horse	508	8.26
8 Goat	514	8.36
9 Monkey	479	7.79
10 Rooster	499	8.11
11 Dog	536	8.71
12 Pig	515	8.37
Total	6,152	100.00

- **cb050_3_ : Chinese Zodiac**

	No	%
1 Rat	349	8.28
2 Ox	346	8.20
3 Tiger	414	9.82
4 Rabbit	382	9.06
5 Dragon	369	8.75
6 Snake	350	8.30
7 Horse	360	8.54
8 Goat	312	7.40
9 Monkey	327	7.75
10 Rooster	334	7.92
11 Dog	334	7.92
12 Pig	340	8.06
Total	4,217	100.00

- **cb050_4_ : Chinese Zodiac**

	No	%
1 Rat	182	7.83
2 Ox	177	7.62
3 Tiger	230	9.90
4 Rabbit	215	9.25
5 Dragon	170	7.31
6 Snake	190	8.18
7 Horse	208	8.95
8 Goat	189	8.13
9 Monkey	182	7.83
10 Rooster	157	6.76
11 Dog	204	8.78
12 Pig	220	9.47
Total	2,324	100.00

- **cb050_5_ : Chinese Zodiac**

	No	%
1 Rat	88	7.82
2 Ox	89	7.90
3 Tiger	87	7.73
4 Rabbit	86	7.64
5 Dragon	106	9.41
6 Snake	96	8.53
7 Horse	88	7.82
8 Goat	91	8.08
9 Monkey	104	9.24
10 Rooster	106	9.41
11 Dog	78	6.93
12 Pig	107	9.50
Total	1,126	100.00

- **cb050_6_ : Chinese Zodiac**

	No	%
1 Rat	45	9.76
2 Ox	30	6.51
3 Tiger	46	9.98
4 Rabbit	45	9.76
5 Dragon	38	8.24
6 Snake	31	6.72
7 Horse	25	5.42
8 Goat	42	9.11
9 Monkey	31	6.72
10 Rooster	48	10.41
11 Dog	42	9.11
12 Pig	38	8.24
Total	461	100.00

- **cb050_7_ : Chinese Zodiac**

	No	%
1 Rat	20	11.76
2 Ox	8	4.71
3 Tiger	16	9.41
4 Rabbit	16	9.41
5 Dragon	8	4.71
6 Snake	17	10.00
7 Horse	24	14.12
8 Goat	13	7.65
9 Monkey	13	7.65
10 Rooster	12	7.06
11 Dog	7	4.12
12 Pig	16	9.41

Total	170	100.00

- **cb050_8_ : Chinese Zodiac**

	No	%
1 Rat	2	4.00
2 Ox	3	6.00
3 Tiger	4	8.00
4 Rabbit	5	10.00
5 Dragon	6	12.00
6 Snake	3	6.00
7 Horse	1	2.00
8 Goat	4	8.00
9 Monkey	4	8.00
10 Rooster	5	10.00
11 Dog	7	14.00
12 Pig	6	12.00
Total	50	100.00

- **cb050_9_ : Chinese Zodiac**

	No	%
2 Ox	1	6.67
3 Tiger	1	6.67
5 Dragon	3	20.00
6 Snake	2	13.33
7 Horse	1	6.67
8 Goat	2	13.33
9 Monkey	2	13.33
11 Dog	1	6.67
12 Pig	2	13.33
Total	15	100.00

- **cb050_10_ : Chinese Zodiac**

	No	%
2 Ox	1	33.33
8 Goat	1	33.33
11 Dog	1	33.33
Total	3	100.00

- **cb050_11_ : Chinese Zodiac**

No Observations

- **cb050_12_ : Chinese Zodiac**

No Observations

- **cb050_13_ : Chinese Zodiac**

No Observations

- **cb050_14_ : Chinese Zodiac**

No Observations

- **cb051_1_1_ : Birth Year of this Child**

Mean	Min	Max	OBS
1,977.5	1,900.0	2,010.0	9,609

- **cb051_1_2_ : Birth Year of this Child**

Mean	Min	Max	OBS
1,977.2	1,900.0	2,011.0	7,889

- **cb051_1_3_ : Birth Year of this Child**

Mean	Min	Max	OBS
1,974.9	1,900.0	2,010.0	4,579

- **cb051_1_4_ : Birth Year of this Child**

Mean	Min	Max	OBS
1,972.9	1,921.0	2,011.0	2,382

- **cb051_1_5_ : Birth Year of this Child**

Mean	Min	Max	OBS
1,971.5	1,939.0	2,006.0	1,133

- **cb051_1_6_ : Birth Year of this Child**

Mean	Min	Max	OBS
1,971.0	1,900.0	1,999.0	451

- **cb051_1_7_ : Birth Year of this Child**

Mean	Min	Max	OBS
1,970.5	1,900.0	1,993.0	167

- **cb051_1_8_ : Birth Year of this Child**

Mean	Min	Max	OBS
1,973.0	1,956.0	2,002.0	52

- **cb051_1_9_ : Birth Year of this Child**

Mean	Min	Max	OBS
1,975.4	1,959.0	2,004.0	16

- **cb051_1_10_ : Birth Year of this Child**

Mean	Min	Max	OBS
1,965.7	1,960.0	1,970.0	3

- **cb051_1_11_ : Birth Year of this Child**

No Observations

- **cb051_1_12_ : Birth Year of this Child**

No Observations

- **cb051_1_13_ : Birth Year of this Child**

No Observations

- **cb051_1_14_ : Birth Year of this Child**

No Observations

- **cb051_2_1_ : Birth Month of this Child**

	No	%
0	371	9.19
1	319	7.90
2	265	6.57
3	258	6.39
4	271	6.71
5	260	6.44
6	276	6.84
7	280	6.94
8	339	8.40
9	379	9.39
10	375	9.29
11	309	7.66
12	334	8.28
Total	4,036	100.00

- **cb051_2_2_ : Birth Month of this Child**

	No	%
0	651	10.64
1	464	7.58
2	462	7.55
3	395	6.45
4	382	6.24
5	394	6.44
6	449	7.34
7	423	6.91
8	515	8.41
9	523	8.54
10	533	8.71
11	472	7.71
12	458	7.48
Total	6,121	100.00

- **cb051_2_3_ : Birth Month of this Child**

	No	%
0	512	12.23
1	284	6.79
2	297	7.10
3	285	6.81
4	295	7.05
5	269	6.43
6	298	7.12
7	273	6.52
8	346	8.27
9	325	7.77
10	368	8.79
11	304	7.26
12	329	7.86
Total	4,185	100.00

- **cb051_2_4_ : Birth Month of this Child**

	No	%
0	346	15.00
1	168	7.29
2	173	7.50
3	173	7.50
4	134	5.81
5	111	4.81
6	160	6.94
7	153	6.63
8	195	8.46
9	195	8.46
10	168	7.29
11	138	5.98
12	192	8.33
Total	2,306	100.00

- **cb051_2_5_ : Birth Month of this Child**

	No	%
0	213	19.03
1	94	8.40
2	62	5.54
3	70	6.26
4	75	6.70
5	75	6.70
6	64	5.72
7	75	6.70
8	73	6.52

9	83	7.42
10	91	8.13
11	71	6.34
12	73	6.52
Total	1,119	100.00

- **cb051_2_6_ : Birth Month of this Child**

	No	%
0	95	21.06
1	36	7.98
2	35	7.76
3	31	6.87
4	32	7.10
5	32	7.10
6	30	6.65
7	23	5.10
8	29	6.43
9	26	5.76
10	35	7.76
11	21	4.66
12	26	5.76
Total	451	100.00

- **cb051_2_7_ : Birth Month of this Child**

	No	%
0	39	23.35
1	10	5.99
2	12	7.19
3	16	9.58
4	14	8.38
5	6	3.59
6	7	4.19
7	10	5.99
8	9	5.39
9	10	5.99
10	13	7.78
11	10	5.99
12	11	6.59
Total	167	100.00

- **cb051_2_8_ : Birth Month of this Child**

	No	%
0	8	15.38
1	4	7.69

2	6	11.54
3	4	7.69
4	3	5.77
5	1	1.92
6	7	13.46
7	5	9.62
8	3	5.77
9	4	7.69
10	4	7.69
12	3	5.77
Total	52	100.00

- **cb051_2_9_ : Birth Month of this Child**

	No	%
0	2	12.50
1	2	12.50
2	1	6.25
3	2	12.50
4	1	6.25
5	1	6.25
6	1	6.25
8	3	18.75
9	2	12.50
12	1	6.25
Total	16	100.00

- **cb051_2_10_ : Birth Month of this Child**

	No	%
0	2	66.67
4	1	33.33
Total	3	100.00

- **cb051_2_11_ : Birth Month of this Child**

No Observations

- **cb051_2_12_ : Birth Month of this Child**

No Observations

- **cb051_2_13_ : Birth Month of this Child**

No Observations

- **cb051_2_14_ : Birth Month of this Child**

No Observations

- **cb052_1_ : Solar or Lunar Calendar**

	No	%
1 Solar Calendar	770	18.90
2 Lunar Calendar	3,303	81.10
Total	4,073	100.00

- **cb052_2_ : Solar or Lunar Calendar**

	No	%
1 Solar Calendar	992	15.99
2 Lunar Calendar	5,213	84.01
Total	6,205	100.00

- **cb052_3_ : Solar or Lunar Calendar**

	No	%
1 Solar Calendar	539	12.62
2 Lunar Calendar	3,732	87.38
Total	4,271	100.00

- **cb052_4_ : Solar or Lunar Calendar**

	No	%
1 Solar Calendar	276	11.58
2 Lunar Calendar	2,107	88.42
Total	2,383	100.00

- **cb052_5_ : Solar or Lunar Calendar**

	No	%

1 Solar Calendar	113	9.75
2 Lunar Calendar	1,046	90.25
Total	1,159	100.00

- **cb052_6_ : Solar or Lunar Calendar**

	No	%
1 Solar Calendar	47	9.87
2 Lunar Calendar	429	90.13
Total	476	100.00

- **cb052_7_ : Solar or Lunar Calendar**

	No	%
1 Solar Calendar	18	10.11
2 Lunar Calendar	160	89.89
Total	178	100.00

- **cb052_8_ : Solar or Lunar Calendar**

	No	%
1 Solar Calendar	8	14.29
2 Lunar Calendar	48	85.71
Total	56	100.00

- **cb052_9_ : Solar or Lunar Calendar**

	No	%
1 Solar Calendar	2	11.11
2 Lunar Calendar	16	88.89
Total	18	100.00

- **cb052_10_ : Solar or Lunar Calendar**

	No	%
1 Solar Calendar	1	33.33
2 Lunar Calendar	2	66.67
Total	3	100.00

- **cb052_11_ : Solar or Lunar Calendar**

No Observations

- **cb052_12_ : Solar or Lunar Calendar**

	No	%
2 Lunar Calendar	1	100.00
Total	1	100.00

- **cb052_13_ : Solar or Lunar Calendar**

No Observations

- **cb052_14_ : Solar or Lunar Calendar**

No Observations

- **cb053_1_ : Where does this Child Live Now**

	No	%
1 This Household, but economicly independent	163	4.01
2 The same or adjacent dwelling/courtyard with me	264	6.49
3 Another Household in this Village/Neighborhood	1,038	25.52
4 Another Village/Neighborhood in this County/City	1,234	30.33
5 Another County/City in this Province	569	13.99
6 Another Province	786	19.32
7 Abroad	14	0.34
Total	4,068	100.00

- **cb053_1_1_ : Distance of Another Village**

Mean	Min	Max	OBS
100.6	0.0	99,999.0	1,231

- **cb053_1_2_ : Distance of Another Village**

Mean	Min	Max	OBS

19.9	0.0	2,000.0	2,103

- **cb053_1_3_ : Distance of Another Village**

Mean	Min	Max	OBS
17.3	0.0	700.0	1,701

- **cb053_1_4_ : Distance of Another Village**

Mean	Min	Max	OBS
16.4	0.0	600.0	995

- **cb053_1_5_ : Distance of Another Village**

Mean	Min	Max	OBS
17.3	0.0	300.0	530

- **cb053_1_6_ : Distance of Another Village**

Mean	Min	Max	OBS
13.5	0.0	100.0	207

- **cb053_1_7_ : Distance of Another Village**

Mean	Min	Max	OBS
20.4	1.0	200.0	78

- **cb053_1_8_ : Distance of Another Village**

Mean	Min	Max	OBS
19.9	1.0	150.0	22

- **cb053_1_9_ : Distance of Another Village**

	No	%
1	1	11.11
2	2	22.22
5	1	11.11
10	2	22.22

16	1	11.11
35	1	11.11
40	1	11.11
Total	9	100.00

- **cb053_1_10_ : Distance of Another Village**

Mean	Min	Max	OBS
183.3	100.0	300.0	3

- **cb053_1_11_ : Distance of Another Village**

No Observations

- **cb053_1_12_ : Distance of Another Village**

No Observations

- **cb053_1_13_ : Distance of Another Village**

No Observations

- **cb053_1_14_ : Distance of Another Village**

No Observations

- **cb053_2_ : Where does this Child Live Now**

	No	%
1 This Household, but economicly independent	210	3.37
2 The same or adjacent dwelling/courtyard with me	333	5.34
3 Another Household in this Village/Neighborhood	1,746	28.01
4 Another Village/Neighborhood in this County/City	2,102	33.72
5 Another County/City in this Province	803	12.88
6 Another Province	1,019	16.35
7 Abroad	21	0.34
Total	6,234	100.00

- **cb053_2_1_ : City**

	No	%
01	32	5.61
04	28	4.91
05	1	0.18
11	37	6.49
16	7	1.23
17	2	0.35
18	2	0.35
24	37	6.49
27	8	1.40
35	3	0.53
38	1	0.18
40	228	40.00
42	4	0.70
46	16	2.81
49	7	1.23
51	2	0.35
52	2	0.35
53	18	3.16
55	10	1.75
56	1	0.18
60	14	2.46
63	8	1.40
66	1	0.18
74	27	4.74
77	1	0.18
82	50	8.77
83	1	0.18
84	13	2.28
86	8	1.40
99	1	0.18
Total	570	100.00

- **cb053_2_2_ : City**

	No	%
01	45	5.60
02	5	0.62
04	43	5.35
05	5	0.62
11	55	6.84
16	6	0.75
17	3	0.37
18	3	0.37
24	43	5.35
27	12	1.49
28	1	0.12
35	2	0.25

40	293	36.44
42	5	0.62
46	33	4.10
49	14	1.74
51	6	0.75
53	31	3.86
55	30	3.73
56	2	0.25
60	27	3.36
63	19	2.36
66	3	0.37
67	2	0.25
74	26	3.23
76	2	0.25
77	1	0.12
82	53	6.59
83	1	0.12
84	15	1.87
86	12	1.49
96	3	0.37
99	3	0.37
Total	804	100.00

- **cb053_2_3_ : City**

	No	%
01	24	4.93
02	5	1.03
04	24	4.93
05	1	0.21
11	35	7.19
16	5	1.03
17	5	1.03
18	2	0.41
24	27	5.54
27	15	3.08
35	1	0.21
38	1	0.21
40	170	34.91
42	2	0.41
46	23	4.72
49	4	0.82
50	1	0.21
51	3	0.62
52	2	0.41
53	24	4.93
55	16	3.29
60	21	4.31
63	10	2.05
66	2	0.41
67	1	0.21

74	14	2.87
76	1	0.21
82	27	5.54
84	9	1.85
86	4	0.82
96	5	1.03
99	3	0.62
Total	487	100.00

- **cb053_2_4_ : City**

	No	%
01	17	6.44
02	1	0.38
04	15	5.68
11	27	10.23
16	5	1.89
17	3	1.14
24	14	5.30
27	6	2.27
35	1	0.38
40	85	32.20
42	2	0.76
46	16	6.06
49	4	1.52
51	2	0.76
52	1	0.38
53	9	3.41
55	8	3.03
56	1	0.38
60	2	0.76
63	3	1.14
66	1	0.38
67	1	0.38
74	14	5.30
77	2	0.76
82	11	4.17
83	1	0.38
84	4	1.52
86	4	1.52
96	2	0.76
99	2	0.76
Total	264	100.00

- **cb053_2_5_ : City**

	No	%
01	3	2.48
02	2	1.65

04	6	4.96
05	1	0.83
11	5	4.13
16	2	1.65
24	12	9.92
27	5	4.13
40	43	35.54
46	6	4.96
49	3	2.48
53	4	3.31
55	3	2.48
60	6	4.96
63	2	1.65
66	1	0.83
67	1	0.83
74	6	4.96
82	6	4.96
84	1	0.83
86	2	1.65
96	1	0.83
Total	121	100.00

- **cb053_2_6_ : City**

	No	%
01	4	8.00
02	1	2.00
05	1	2.00
11	3	6.00
17	1	2.00
18	1	2.00
27	2	4.00
35	1	2.00
40	19	38.00
46	1	2.00
53	6	12.00
60	1	2.00
63	1	2.00
74	2	4.00
82	4	8.00
84	1	2.00
86	1	2.00
Total	50	100.00

- **cb053_2_7_ : City**

	No	%
01	2	8.70
02	1	4.35

04	1	4.35
11	1	4.35
24	1	4.35
27	1	4.35
40	5	21.74
46	3	13.04
49	1	4.35
51	1	4.35
52	1	4.35
55	1	4.35
60	3	13.04
82	1	4.35
Total	23	100.00

- **cb053_2_8_ : City**

	No	%
01	1	12.50
11	1	12.50
40	2	25.00
49	1	12.50
52	1	12.50
55	1	12.50
84	1	12.50
Total	8	100.00

- **cb053_2_9_ : City**

	No	%
04	1	100.00
Total	1	100.00

- **cb053_3_ : Where does this Child Live Now**

	No	%
1 This Household, but economicly independent	106	2.46
2 The same or adjacent dwelling/courtyard with me	173	4.01
3 Another Household in this Village/Neighborhood	1,191	27.61
4 Another Village/Neighborhood in this County/City	1,705	39.53
5 Another County/City in this Province	486	11.27
6 Another Province	638	14.79
7 Abroad	14	0.32
Total	4,313	100.00

- **cb053_3_1_ : County**

	No	%
02	8	1.70
03	2	0.43
04	33	7.02
06	21	4.47
07	1	0.21
08	2	0.43
13	4	0.85
16	16	3.40
28	21	4.47
31	24	5.11
33	2	0.43
36	1	0.21
37	1	0.21
38	1	0.21
39	6	1.28
43	8	1.70
44	6	1.28
45	2	0.43
46	2	0.43
51	5	1.06
54	14	2.98
56	11	2.34
57	14	2.98
58	4	0.85
59	7	1.49
63	199	42.34
71	1	0.21
75	3	0.64
76	10	2.13
78	21	4.47
81	1	0.21
90	2	0.43
91	2	0.43
92	12	2.55
99	3	0.64
Total	470	100.00

- **cb053_3_2_ : County**

	No	%
02	20	2.93
03	7	1.02
04	56	8.20
06	27	3.95
07	2	0.29
08	4	0.59
13	2	0.29
16	7	1.02
28	30	4.39

31	38	5.56
33	2	0.29
37	15	2.20
38	3	0.44
39	10	1.46
43	11	1.61
44	6	0.88
46	13	1.90
49	1	0.15
51	3	0.44
54	27	3.95
56	9	1.32
57	10	1.46
58	5	0.73
59	33	4.83
63	256	37.48
75	4	0.59
76	25	3.66
78	17	2.49
81	5	0.73
89	1	0.15
90	2	0.29
91	1	0.15
92	26	3.81
97	1	0.15
99	4	0.59
Total	683	100.00

- **cb053_3_3_ : County**

	No	%
02	13	3.22
03	3	0.74
04	27	6.68
06	20	4.95
08	2	0.50
13	1	0.25
16	11	2.72
28	19	4.70
31	26	6.44
33	1	0.25
36	1	0.25
37	5	1.24
39	8	1.98
43	7	1.73
44	2	0.50
45	1	0.25
46	6	1.49
50	1	0.25
51	6	1.49
54	14	3.47

56	8	1.98
57	7	1.73
58	2	0.50
59	24	5.94
63	137	33.91
71	2	0.50
75	3	0.74
76	13	3.22
78	6	1.49
81	4	0.99
89	1	0.25
90	2	0.50
91	1	0.25
92	13	3.22
99	7	1.73
Total	404	100.00

- **cb053_3_4_ : County**

	No	%
02	4	1.87
03	3	1.40
04	17	7.94
06	17	7.94
08	2	0.93
13	1	0.47
16	8	3.74
23	1	0.47
28	8	3.74
31	11	5.14
37	2	0.93
39	1	0.47
43	4	1.87
44	1	0.47
46	5	2.34
51	3	1.40
54	5	2.34
57	6	2.80
58	2	0.93
59	12	5.61
63	69	32.24
75	1	0.47
76	7	3.27
78	7	3.27
81	5	2.34
89	1	0.47
91	1	0.47
92	6	2.80
99	4	1.87
Total	214	100.00

- **cb053_3_5_ : County**

	No	%
02	7	6.73
03	1	0.96
04	6	5.77
06	6	5.77
16	3	2.88
28	4	3.85
31	9	8.65
37	1	0.96
43	2	1.92
44	1	0.96
46	1	0.96
51	1	0.96
54	1	0.96
56	2	1.92
57	7	6.73
58	1	0.96
59	7	6.73
63	31	29.81
76	3	2.88
78	4	3.85
91	3	2.88
92	2	1.92
99	1	0.96
Total	104	100.00

- **cb053_3_6_ : County**

	No	%
02	1	2.33
04	3	6.98
06	1	2.33
13	1	2.33
16	2	4.65
28	3	6.98
31	4	9.30
43	1	2.33
44	1	2.33
46	1	2.33
51	1	2.33
57	2	4.65
59	4	9.30
63	11	25.58
76	3	6.98
78	2	4.65
91	1	2.33
92	1	2.33
Total	43	100.00

- **cb053_3_7_ : County**

	No	%
03	1	4.76
06	5	23.81
31	1	4.76
56	1	4.76
57	1	4.76
59	2	9.52
63	8	38.10
76	1	4.76
81	1	4.76
Total	21	100.00

- **cb053_3_8_ : County**

	No	%
03	1	16.67
16	1	16.67
56	1	16.67
63	3	50.00
Total	6	100.00

- **cb053_3_9_ : County**

	No	%
63	1	100.00
Total	1	100.00

- **cb053_4_ : Where does this Child Live Now**

	No	%
1 This Household, but economicly independent	58	2.39
2 The same or adjacent dwelling/courtyard with me	85	3.50
3 Another Household in this Village/Neighborhood	656	27.02
4 Another Village/Neighborhood in this County/City	993	40.90
5 Another County/City in this Province	267	11.00
6 Another Province	361	14.87
7 Abroad	8	0.33
Total	2,428	100.00

- **cb053_4_1_ : Distance of Another County**

Mean	Min	Max	OBS

361,468.0	-9,999.0	200,000,000.0	554

- **cb053_4_2_ : Distance of Another County**

Mean	Min	Max	OBS
518.0	-9,999.0	115,000.0	784

- **cb053_4_3_ : Distance of Another County**

Mean	Min	Max	OBS
263.3	-9,999.0	10,000.0	470

- **cb053_4_4_ : Distance of Another County**

Mean	Min	Max	OBS
518.6	-9,999.0	50,000.0	256

- **cb053_4_5_ : Distance of Another County**

Mean	Min	Max	OBS
202.8	0.0	1,500.0	116

- **cb053_4_6_ : Distance of Another County**

Mean	Min	Max	OBS
164.0	-999.0	750.0	48

- **cb053_4_7_ : Distance of Another County**

Mean	Min	Max	OBS
311.1	4.0	2,500.0	23

- **cb053_4_8_ : Distance of Another County**

Mean	Min	Max	OBS
194.9	4.0	500.0	7

- **cb053_4_9_ : Distance of Another County**

Mean	Min	Max	OBS
200.0	200.0	200.0	1

- **cb053_4_10_ : Distance of Another County**

No Observations

- **cb053_4_11_ : Distance of Another County**

No Observations

- **cb053_4_12_ : Distance of Another County**

No Observations

- **cb053_4_13_ : Distance of Another County**

No Observations

- **cb053_4_14_ : Distance of Another County**

Mean	Min	Max	OBS
150.0	150.0	150.0	1

- **cb053_5_ : Where does this Child Live Now**

	No	%
1 This Household, but economicly independent	22	1.84
2 The same or adjacent dwelling/courtyard with me	40	3.34
3 Another Household in this Village/Neighborhood	322	26.90
4 Another Village/Neighborhood in this County/City	530	44.28
5 Another County/City in this Province	120	10.03
6 Another Province	162	13.53
7 Abroad	1	0.08
Total	1,197	100.00

- **cb053_5_1_ : Province**

	No	%
01	9	1.14
02	6	0.76
03	26	3.30
04	1	0.13
05	9	1.14
06	34	4.31
07	4	0.51
08	22	2.79
09	83	10.52
10	21	2.66
11	41	5.20
12	16	2.03
13	5	0.63
14	6	0.76
15	208	26.36
16	19	2.41
17	7	0.89
18	59	7.48
19	16	2.03
20	79	10.01
21	4	0.51
23	9	1.14
24	12	1.52
25	7	0.89
26	7	0.89
27	12	1.52
28	13	1.65
29	18	2.28
32	15	1.90
33	11	1.39
34	10	1.27
Total	789	100.00

- **cb053_5_2_ : Province**

	No	%
01	14	1.36
02	6	0.58
03	40	3.90
05	21	2.05
06	21	2.05
07	11	1.07
08	26	2.53
09	84	8.19
10	20	1.95
11	72	7.02
12	24	2.34

13	8	0.78
14	19	1.85
15	246	23.98
16	39	3.80
17	10	0.97
18	86	8.38
19	24	2.34
20	111	10.82
21	11	1.07
23	12	1.17
24	14	1.36
25	4	0.39
26	14	1.36
27	15	1.46
28	18	1.75
29	20	1.95
32	14	1.36
33	14	1.36
34	8	0.78
Total	1,026	100.00

- **cb053_5_3_ : Province**

	No	%
01	6	0.94
02	1	0.16
03	19	2.99
05	15	2.36
06	27	4.25
07	2	0.31
08	24	3.77
09	45	7.08
10	12	1.89
11	40	6.29
12	14	2.20
13	6	0.94
14	13	2.04
15	155	24.37
16	21	3.30
17	5	0.79
18	54	8.49
19	9	1.42
20	67	10.53
21	13	2.04
23	4	0.63
24	9	1.42
26	15	2.36
27	11	1.73
28	7	1.10
29	19	2.99
32	12	1.89

33	4	0.63
34	7	1.10
Total	636	100.00

- **cb053_5_4_ : Province**

	No	%
01	5	1.36
02	2	0.54
03	14	3.80
05	7	1.90
06	15	4.08
07	2	0.54
08	13	3.53
09	35	9.51
10	8	2.17
11	30	8.15
12	7	1.90
13	2	0.54
14	5	1.36
15	60	16.30
16	11	2.99
17	5	1.36
18	29	7.88
19	9	2.45
20	47	12.77
21	5	1.36
23	3	0.82
24	7	1.90
25	7	1.90
26	5	1.36
27	5	1.36
28	4	1.09
29	14	3.80
32	6	1.63
33	5	1.36
34	1	0.27
Total	368	100.00

- **cb053_5_5_ : Province**

	No	%
01	2	1.24
02	1	0.62
03	7	4.35
05	5	3.11
06	6	3.73
08	2	1.24
09	7	4.35

11	12	7.45
12	5	3.11
13	1	0.62
14	5	3.11
15	35	21.74
16	2	1.24
17	3	1.86
18	17	10.56
19	1	0.62
20	19	11.80
21	5	3.11
23	1	0.62
24	4	2.48
25	2	1.24
26	2	1.24
27	3	1.86
28	5	3.11
29	6	3.73
33	1	0.62
34	2	1.24
Total	161	100.00

- **cb053_5_6_ : Province**

	No	%
03	1	1.69
05	1	1.69
06	1	1.69
09	5	8.47
10	1	1.69
11	1	1.69
12	2	3.39
14	2	3.39
15	11	18.64
17	2	3.39
18	7	11.86
19	1	1.69
20	13	22.03
21	2	3.39
23	1	1.69
24	1	1.69
25	2	3.39
27	1	1.69
29	2	3.39
32	1	1.69
33	1	1.69
Total	59	100.00

- **cb053_5_7_ : Province**

	No	%
01	1	5.56
03	1	5.56
09	1	5.56
13	1	5.56
14	1	5.56
15	1	5.56
16	1	5.56
17	1	5.56
18	1	5.56
20	3	16.67
21	1	5.56
26	1	5.56
27	1	5.56
29	1	5.56
33	1	5.56
34	1	5.56
Total	18	100.00

- **cb053_5_8_ : Province**

	No	%
09	1	12.50
11	1	12.50
13	1	12.50
15	2	25.00
21	1	12.50
23	1	12.50
26	1	12.50
Total	8	100.00

- **cb053_5_9_ : Province**

	No	%
15	1	50.00
26	1	50.00
Total	2	100.00

- **cb053_6_ : Where does this Child Live Now**

	No	%
1 This Household, but economicly independent	14	2.83
2 The same or adjacent dwelling/courtyard with me	16	3.24
3 Another Household in this Village/Neighborhood	150	30.36
4 Another Village/Neighborhood in this County/City	206	41.70

5 Another County/City in this Province	50	10.12
6 Another Province	58	11.74
Total	494	100.00

- **cb053_6_1_ : City**

	No	%
01	23	3.41
02	1	0.15
04	33	4.89
05	2	0.30
11	91	13.48
16	3	0.44
17	1	0.15
18	2	0.30
24	66	9.78
27	2	0.30
28	2	0.30
38	3	0.44
40	298	44.15
46	10	1.48
49	2	0.30
53	19	2.81
55	9	1.33
56	2	0.30
60	12	1.78
63	4	0.59
66	7	1.04
74	21	3.11
76	1	0.15
77	1	0.15
82	19	2.81
83	1	0.15
84	22	3.26
86	5	0.74
96	7	1.04
99	6	0.89
Total	675	100.00

- **cb053_6_2_ : City**

	No	%
01	55	6.52
02	2	0.24
04	30	3.56
07	1	0.12
11	116	13.76
16	5	0.59
18	6	0.71

24	84	9.96
25	1	0.12
27	10	1.19
28	3	0.36
38	5	0.59
40	352	41.76
46	10	1.19
49	6	0.71
51	2	0.24
53	28	3.32
55	9	1.07
60	9	1.07
63	7	0.83
66	5	0.59
74	14	1.66
82	31	3.68
84	34	4.03
86	8	0.95
89	2	0.24
96	4	0.47
99	4	0.47
Total	843	100.00

- **cb053_6_3_ : City**

	No	%
01	26	4.62
02	1	0.18
04	27	4.80
05	2	0.36
11	67	11.90
16	2	0.36
18	2	0.36
24	60	10.66
25	3	0.53
27	7	1.24
28	2	0.36
38	3	0.53
40	219	38.90
46	9	1.60
49	4	0.71
51	3	0.53
52	2	0.36
53	25	4.44
55	9	1.60
56	1	0.18
60	11	1.95
63	2	0.36
66	4	0.71
74	14	2.49
82	23	4.09

84	19	3.37
86	2	0.36
96	7	1.24
99	7	1.24
Total	563	100.00

- **cb053_6_4_ : City**

	No	%
01	11	3.51
02	1	0.32
04	12	3.83
11	33	10.54
16	2	0.64
18	1	0.32
24	38	12.14
38	2	0.64
40	137	43.77
46	11	3.51
49	2	0.64
51	1	0.32
52	1	0.32
53	12	3.83
55	10	3.19
60	4	1.28
63	2	0.64
66	4	1.28
69	1	0.32
74	6	1.92
82	7	2.24
83	1	0.32
84	6	1.92
86	2	0.64
88	1	0.32
96	1	0.32
99	4	1.28
Total	313	100.00

- **cb053_6_5_ : City**

	No	%
01	7	5.00
04	6	4.29
11	13	9.29
18	1	0.71
24	15	10.71
27	1	0.71
28	1	0.71
40	57	40.71

46	4	2.86
51	1	0.71
52	1	0.71
53	7	5.00
55	4	2.86
60	2	1.43
63	2	1.43
66	2	1.43
74	3	2.14
82	5	3.57
84	7	5.00
99	1	0.71
Total	140	100.00

- **cb053_6_6_ : City**

	No	%
04	1	2.17
05	1	2.17
11	2	4.35
24	5	10.87
40	24	52.17
46	1	2.17
49	2	4.35
53	2	4.35
55	2	4.35
60	1	2.17
74	2	4.35
82	1	2.17
86	2	4.35
Total	46	100.00

- **cb053_6_7_ : City**

	No	%
01	1	7.69
04	1	7.69
11	1	7.69
18	1	7.69
24	2	15.38
40	6	46.15
60	1	7.69
Total	13	100.00

- **cb053_6_8_ : City**

	No	%

04	1	14.29
24	1	14.29
40	2	28.57
60	1	14.29
63	1	14.29
82	1	14.29
Total	7	100.00

- **cb053_6_9_ : City**

	No	%
60	1	100.00
Total	1	100.00

- **cb053_7_ : Where does this Child Live Now**

	No	%
1 This Household, but economicly independent	6	3.23
2 The same or adjacent dwelling/courtyard with me	8	4.30
3 Another Household in this Village/Neighborhood	52	27.96
4 Another Village/Neighborhood in this County/City	78	41.94
5 Another County/City in this Province	23	12.37
6 Another Province	19	10.22
Total	186	100.00

- **cb053_7_1_ : County**

	No	%
02	7	1.35
03	1	0.19
04	6	1.16
06	8	1.55
08	8	1.55
10	1	0.19
13	6	1.16
16	6	1.16
23	4	0.77
28	54	10.44
31	19	3.68
37	3	0.58
38	5	0.97
39	6	1.16
43	9	1.74
44	5	0.97
46	4	0.77
50	1	0.19
51	2	0.39

54	18	3.48
56	9	1.74
57	7	1.35
58	2	0.39
59	9	1.74
63	245	47.39
73	1	0.19
75	7	1.35
76	8	1.55
78	16	3.09
81	6	1.16
89	1	0.19
91	1	0.19
92	24	4.64
99	8	1.55
Total	517	100.00

- **cb053_7_2_ : County**

	No	%
02	6	1.00
03	2	0.33
04	8	1.33
06	16	2.66
07	1	0.17
08	7	1.16
13	12	2.00
16	9	1.50
23	5	0.83
28	16	2.66
31	30	4.99
33	1	0.17
37	6	1.00
38	9	1.50
39	12	2.00
43	4	0.67
44	7	1.16
45	2	0.33
46	11	1.83
50	3	0.50
51	6	1.00
54	21	3.49
56	13	2.16
57	5	0.83
58	6	1.00
59	22	3.66
63	287	47.75
67	1	0.17
73	4	0.67
75	14	2.33
76	2	0.33

78	22	3.66
81	4	0.67
91	2	0.33
92	17	2.83
97	2	0.33
99	6	1.00
Total	601	100.00

- **cb053_7_3_ : County**

	No	%
02	5	1.22
03	2	0.49
04	10	2.43
06	9	2.19
08	6	1.46
10	1	0.24
13	5	1.22
16	10	2.43
23	3	0.73
28	6	1.46
31	20	4.87
37	4	0.97
38	4	0.97
39	9	2.19
43	5	1.22
44	4	0.97
46	8	1.95
51	6	1.46
54	13	3.16
56	8	1.95
57	4	0.97
58	8	1.95
59	13	3.16
63	202	49.15
73	1	0.24
75	10	2.43
76	6	1.46
78	10	2.43
81	5	1.22
89	1	0.24
90	1	0.24
92	10	2.43
97	1	0.24
99	1	0.24
Total	411	100.00

- **cb053_7_4_ : County**

	No	%
02	1	0.46
03	4	1.84
04	1	0.46
06	7	3.23
08	6	2.76
10	1	0.46
13	6	2.76
16	5	2.30
19	1	0.46
23	1	0.46
28	5	2.30
31	11	5.07
37	4	1.84
38	4	1.84
39	2	0.92
43	4	1.84
44	1	0.46
46	3	1.38
51	4	1.84
54	8	3.69
56	3	1.38
58	4	1.84
59	12	5.53
63	92	42.40
71	1	0.46
75	3	1.38
76	6	2.76
78	8	3.69
81	1	0.46
92	6	2.76
99	2	0.92
Total	217	100.00

- **cb053_7_5_ : County**

	No	%
04	1	0.94
06	4	3.77
08	1	0.94
13	3	2.83
23	3	2.83
28	3	2.83
31	9	8.49
39	3	2.83
43	3	2.83
44	1	0.94
46	4	3.77
49	1	0.94
51	1	0.94

54	5	4.72
56	2	1.89
58	2	1.89
59	6	5.66
63	38	35.85
73	1	0.94
75	4	3.77
76	4	3.77
78	3	2.83
92	3	2.83
99	1	0.94
Total	106	100.00

- **cb053_7_6_ : County**

	No	%
06	2	5.41
13	1	2.70
16	1	2.70
28	1	2.70
31	2	5.41
38	1	2.70
46	1	2.70
54	2	5.41
58	1	2.70
59	1	2.70
63	19	51.35
75	2	5.41
76	1	2.70
91	1	2.70
92	1	2.70
Total	37	100.00

- **cb053_7_7_ : County**

	No	%
04	1	11.11
06	1	11.11
51	1	11.11
63	5	55.56
76	1	11.11
Total	9	100.00

- **cb053_7_8_ : County**

	No	%
06	2	28.57

13	1	14.29
31	1	14.29
59	1	14.29
63	1	14.29
76	1	14.29
Total	7	100.00

- **cb053_7_9_ : County**

	No	%
06	1	100.00
Total	1	100.00

- **cb053_8_ : Where does this Child Live Now**

	No	%
1 This Household, but economicly independent	1	1.64
2 The same or adjacent dwelling/courtyard with me	3	4.92
3 Another Household in this Village/Neighborhood	19	31.15
4 Another Village/Neighborhood in this County/City	22	36.07
5 Another County/City in this Province	8	13.11
6 Another Province	8	13.11
Total	61	100.00

- **cb053_8_1_ : Distance of Another Province**

Mean	Min	Max	OBS
41,193.6	-9,999.0	20,000,000.0	681

- **cb053_8_2_ : Distance of Another Province**

Mean	Min	Max	OBS
4,731.3	-9,999.0	600,000.0	885

- **cb053_8_3_ : Distance of Another Province**

Mean	Min	Max	OBS
5,502,067.5	-9,999.0	3000000000.0	550

- **cb053_8_4_ : Distance of Another Province**

Mean	Min	Max	OBS

132,352.0	-9,999.0	40,000,000.0	313

- **cb053_8_5_ : Distance of Another Province**

Mean	Min	Max	OBS
1,493.5	0.0	20,000.0	141

- **cb053_8_6_ : Distance of Another Province**

Mean	Min	Max	OBS
2,225.1	0.0	20,000.0	50

- **cb053_8_7_ : Distance of Another Province**

Mean	Min	Max	OBS
1,570.6	-999.0	9,000.0	17

- **cb053_8_8_ : Distance of Another Province**

Mean	Min	Max	OBS
1,433.3	200.0	2,500.0	6

- **cb053_8_9_ : Distance of Another Province**

Mean	Min	Max	OBS
1,400.0	800.0	2,000.0	2

- **cb053_8_10_ : Distance of Another Province**

No Observations

- **cb053_8_11_ : Distance of Another Province**

No Observations

- **cb053_8_12_ : Distance of Another Province**

No Observations

- **cb053_8_13_ : Distance of Another Province**

No Observations

- **cb053_8_14_ : Distance of Another Province**

No Observations

- **cb053_9_ : Where does this Child Live Now**

	No	%
1 This Household, but economicly independent	1	4.55
2 The same or adjacent dwelling/courtyard with me	1	4.55
3 Another Household in this Village/Neighborhood	8	36.36
4 Another Village/Neighborhood in this County/City	9	40.91
5 Another County/City in this Province	1	4.55
6 Another Province	2	9.09
Total	22	100.00

- **cb053_10_ : Where does this Child Live Now**

	No	%
3 Another Household in this Village/Neighborhood	3	50.00
4 Another Village/Neighborhood in this County/City	3	50.00
Total	6	100.00

- **cb053_11_ : Where does this Child Live Now**

No Observations

- **cb053_12_ : Where does this Child Live Now**

No Observations

- **cb053_13_ : Where does this Child Live Now**

	No	%
5 Another County/City in this Province	1	100.00
Total	1	100.00

- **cb053_14_ : Where does this Child Live Now**

No Observations

- **cb054_1_ : Type of Location this Child Live**

	No	%
1 City	1,575	38.41
2 County	551	13.44
3 Town	268	6.54
4 Village	1,706	41.61
Total	4,100	100.00

- **cb054_2_ : Type of Location this Child Live**

	No	%
1 City	1,858	29.58
2 County	827	13.16
3 Town	413	6.57
4 Village	3,184	50.68
Total	6,282	100.00

- **cb054_3_ : Type of Location this Child Live**

	No	%
1 City	1,068	24.55
2 County	506	11.63
3 Town	320	7.36
4 Village	2,456	56.46
Total	4,350	100.00

- **cb054_4_ : Type of Location this Child Live**

	No	%
1 City	560	22.96
2 County	281	11.52
3 Town	155	6.36
4 Village	1,443	59.16
Total	2,439	100.00

- **cb054_5_ : Type of Location this Child Live**

	No	%
1 City	248	20.63
2 County	118	9.82
3 Town	77	6.41
4 Village	759	63.14
Total	1,202	100.00

- **cb054_6_ : Type of Location this Child Live**

	No	%
1 City	93	18.83
2 County	44	8.91
3 Town	33	6.68
4 Village	324	65.59
Total	494	100.00

- **cb054_7_ : Type of Location this Child Live**

	No	%
1 City	26	13.90
2 County	20	10.70
3 Town	10	5.35
4 Village	131	70.05
Total	187	100.00

- **cb054_8_ : Type of Location this Child Live**

	No	%
1 City	10	16.39
2 County	5	8.20
3 Town	2	3.28
4 Village	44	72.13
Total	61	100.00

- **cb054_9_ : Type of Location this Child Live**

	No	%
1 City	2	9.09
2 County	1	4.55
3 Town	2	9.09
4 Village	17	77.27
Total	22	100.00

- **cb054_10_ : Type of Location this Child Live**

	No	%
1 City	2	33.33
2 County	1	16.67
3 Town	1	16.67
4 Village	2	33.33
Total	6	100.00

- **cb054_11_ : Type of Location this Child Live**

No Observations

- **cb054_12_ : Type of Location this Child Live**

No Observations

- **cb054_13_ : Type of Location this Child Live**

No Observations

- **cb054_14_ : Type of Location this Child Live**

No Observations

- **cb055_1_ : Current Hukou Status of this Child**

	No	%

1 Agriculture Hukou	2,795	67.76
2 Non-Agriculture Hukou	1,290	31.27
3 Unified Residency Hukou	34	0.82
4 Do not have Hukou	6	0.15
Total	4,125	100.00

- **cb055_2_ : Current Hukou Status of this Child**

	No	%
1 Agriculture Hukou	4,694	74.50
2 Non-Agriculture Hukou	1,548	24.57
3 Unified Residency Hukou	46	0.73
4 Do not have Hukou	13	0.21
Total	6,301	100.00

- **cb055_3_ : Current Hukou Status of this Child**

	No	%
1 Agriculture Hukou	3,425	78.54
2 Non-Agriculture Hukou	903	20.71
3 Unified Residency Hukou	27	0.62
4 Do not have Hukou	6	0.14
Total	4,361	100.00

- **cb055_4_ : Current Hukou Status of this Child**

	No	%
1 Agriculture Hukou	1,957	79.94
2 Non-Agriculture Hukou	475	19.40
3 Unified Residency Hukou	14	0.57
4 Do not have Hukou	2	0.08
Total	2,448	100.00

- **cb055_5_ : Current Hukou Status of this Child**

	No	%
1 Agriculture Hukou	981	81.55
2 Non-Agriculture Hukou	212	17.62
3 Unified Residency Hukou	6	0.50
4 Do not have Hukou	4	0.33
Total	1,203	100.00

- **cb055_6_ : Current Hukou Status of this Child**

	No	%
1 Agriculture Hukou	419	84.14
2 Non-Agriculture Hukou	76	15.26
3 Unified Residency Hukou	2	0.40
4 Do not have Hukou	1	0.20
Total	498	100.00

- **cb055_7_ : Current Hukou Status of this Child**

	No	%
1 Agriculture Hukou	155	82.89
2 Non-Agriculture Hukou	32	17.11
Total	187	100.00

- **cb055_8_ : Current Hukou Status of this Child**

	No	%
1 Agriculture Hukou	53	85.48
2 Non-Agriculture Hukou	9	14.52
Total	62	100.00

- **cb055_9_ : Current Hukou Status of this Child**

	No	%
1 Agriculture Hukou	17	77.27
2 Non-Agriculture Hukou	5	22.73
Total	22	100.00

- **cb055_10_ : Current Hukou Status of this Child**

	No	%
1 Agriculture Hukou	3	50.00
2 Non-Agriculture Hukou	3	50.00
Total	6	100.00

- **cb055_11_ : Current Hukou Status of this Child**

No Observations

- **cb055_12_ : Current Hukou Status of this Child**

No Observations

- **cb055_13_ : Current Hukou Status of this Child**

No Observations

- **cb055_14_ : Current Hukou Status of this Child**

No Observations

- **cb056_1_ : Is this Child's Hukou Status same as His/Her Place of Residence**

	No	%
1 Yes	2,698	66.27
2 No	1,373	33.73
Total	4,071	100.00

- **cb056_2_ : Is this Child's Hukou Status same as His/Her Place of Residence**

	No	%
1 Yes	4,369	70.05
2 No	1,868	29.95
Total	6,237	100.00

- **cb056_3_ : Is this Child's Hukou Status same as His/Her Place of Residence**

	No	%
1 Yes	3,132	72.52
2 No	1,187	27.48
Total	4,319	100.00

- **cb056_4_ : Is this Child's Hukou Status same as His/Her Place of Resi-**

dence

	No	%
1 Yes	1,772	72.92
2 No	658	27.08
Total	2,430	100.00

- **cb056_5_ : Is this Child's Hukou Status same as His/Her Place of Residence**

	No	%
1 Yes	887	74.54
2 No	303	25.46
Total	1,190	100.00

- **cb056_6_ : Is this Child's Hukou Status same as His/Her Place of Residence**

	No	%
1 Yes	378	76.52
2 No	116	23.48
Total	494	100.00

- **cb056_7_ : Is this Child's Hukou Status same as His/Her Place of Residence**

	No	%
1 Yes	151	80.75
2 No	36	19.25
Total	187	100.00

- **cb056_8_ : Is this Child's Hukou Status same as His/Her Place of Residence**

	No	%
1 Yes	47	77.05
2 No	14	22.95
Total	61	100.00

- **cb056_9_ : Is this Child's Hukou Status same as His/Her Place of Residence**

	No	%
1 Yes	17	77.27
2 No	5	22.73
Total	22	100.00

- **cb056_10_ : Is this Child's Hukou Status same as His/Her Place of Residence**

	No	%
1 Yes	6	100.00
Total	6	100.00

- **cb057_1_ : The Current Hukou Location of this Child**

	No	%
1 This Household	791	57.11
2 This village/Neighborhood	278	20.07
3 Another Village/Neighborhood in this County/City	222	16.03
4 Another County/City in this Province	56	4.04
5 Another Province	36	2.60
6 Other	2	0.14
Total	1,385	100.00

- **cb057_1_1_ : City and County**

	No	%
01	3	5.17
0106	1	1.72
04	1	1.72
0476	2	3.45
0563	2	3.45
11	4	6.90
17	1	1.72
24	7	12.07
27	1	1.72
40	13	22.41
4028	1	1.72
4038	1	1.72
46	1	1.72
4631	2	3.45
4659	1	1.72
51	1	1.72
55	3	5.17
5581	1	1.72
6059	1	1.72

66	1	1.72
6631	2	3.45
74	1	1.72
82	2	3.45
8202	1	1.72
8204	1	1.72
8228	1	1.72
8254	1	1.72
9931	1	1.72
Total	58	100.00

- **cb057_1_2_ : City and County**

	No	%
01	3	3.57
0106	1	1.19
0143	1	1.19
0159	1	1.19
0431	1	1.19
0444	1	1.19
0446	1	1.19
0454	1	1.19
11	4	4.76
1102	1	1.19
1131	1	1.19
1159	1	1.19
1659	1	1.19
1863	1	1.19
24	3	3.57
2402	1	1.19
2439	1	1.19
2444	1	1.19
2454	1	1.19
27	1	1.19
2704	1	1.19
2731	1	1.19
2759	1	1.19
2846	1	1.19
40	14	16.67
4004	2	2.38
4008	1	1.19
4046	1	1.19
4058	1	1.19
4063	1	1.19
46	3	3.57
4643	1	1.19
4954	1	1.19
50	1	1.19
53	5	5.95
5303	1	1.19
5381	1	1.19

55	2	2.38
6031	1	1.19
6054	1	1.19
66	1	1.19
74	1	1.19
7443	1	1.19
7451	1	1.19
7454	2	2.38
7702	1	1.19
82	3	3.57
8206	2	2.38
8243	1	1.19
8246	1	1.19
8276	1	1.19
86	1	1.19
Total	84	100.00

- **cb057_1_3_ : City and County**

	No	%
01	1	2.08
0143	1	2.08
04	1	2.08
0451	1	2.08
11	1	2.08
1131	1	2.08
1159	1	2.08
1631	1	2.08
1863	1	2.08
24	3	6.25
2431	1	2.08
27	2	4.17
2704	2	4.17
40	6	12.50
4059	1	2.08
4063	1	2.08
46	3	6.25
4628	1	2.08
4659	1	2.08
53	4	8.33
5381	1	2.08
60	2	4.17
6004	1	2.08
6031	1	2.08
6054	1	2.08
6343	1	2.08
6376	1	2.08
74	2	4.17
7404	1	2.08
82	1	2.08
8257	1	2.08

8259	1	2.08
Total	48	100.00

- **cb057_1_4_ : City and County**

	No	%
0106	1	3.57
0445	1	3.57
11	2	7.14
1131	1	3.57
16	1	3.57
2431	1	3.57
2704	1	3.57
40	6	21.43
4031	1	3.57
46	1	3.57
49	1	3.57
50	1	3.57
5263	1	3.57
53	1	3.57
6043	2	7.14
6059	1	3.57
7763	1	3.57
8206	1	3.57
8239	1	3.57
8276	1	3.57
9663	1	3.57
Total	28	100.00

- **cb057_1_5_ : City and County**

	No	%
0143	1	4.17
0159	1	4.17
0202	1	4.17
0476	1	4.17
2759	1	4.17
40	2	8.33
4031	1	4.17
46	1	4.17
4628	1	4.17
4631	1	4.17
53	1	4.17
55	2	8.33
6002	1	4.17
6004	1	4.17
6328	1	4.17
6759	1	4.17
74	2	8.33

82	1	4.17
8257	2	8.33
8602	1	4.17
Total	24	100.00

- **cb057_1_6_ : City and County**

	No	%
1131	1	33.33
1159	1	33.33
7404	1	33.33
Total	3	100.00

- **cb057_1_7_ : City and County**

	No	%
55	1	100.00
Total	1	100.00

- **cb057_1_8_ : City and County**

	No	%
5203	1	100.00
Total	1	100.00

- **cb057_2_ : The Current Hukou Location of this Child**

	No	%
1 This Household	989	52.75
2 This village/Neighborhood	345	18.40
3 Another Village/Neighborhood in this County/City	384	20.48
4 Another County/City in this Province	85	4.53
5 Another Province	69	3.68
6 Other	3	0.16
Total	1,875	100.00

- **cb057_2_1_ : Province**

	No	%
03	1	2.78
05	1	2.78
06	4	11.11
07	2	5.56

08	2	5.56
09	1	2.78
11	1	2.78
12	1	2.78
13	1	2.78
14	1	2.78
15	4	11.11
16	1	2.78
17	1	2.78
18	4	11.11
19	1	2.78
20	3	8.33
21	1	2.78
26	1	2.78
27	1	2.78
28	1	2.78
32	2	5.56
33	1	2.78
Total	36	100.00

- **cb057_2_2_ : Province**

	No	%
01	1	1.43
05	4	5.71
06	3	4.29
07	5	7.14
08	1	1.43
09	5	7.14
11	5	7.14
12	6	8.57
13	2	2.86
14	4	5.71
15	7	10.00
16	2	2.86
17	1	1.43
18	1	1.43
20	4	5.71
23	1	1.43
24	2	2.86
26	2	2.86
27	1	1.43
28	1	1.43
29	3	4.29
32	2	2.86
33	5	7.14
34	2	2.86
Total	70	100.00

- **cb057_2_3_ : Province**

	No	%
05	3	8.33
06	3	8.33
07	3	8.33
09	1	2.78
10	2	5.56
12	4	11.11
13	1	2.78
14	2	5.56
15	2	5.56
16	1	2.78
18	1	2.78
20	2	5.56
21	1	2.78
23	1	2.78
24	1	2.78
27	3	8.33
28	1	2.78
29	1	2.78
33	2	5.56
34	1	2.78
Total	36	100.00

- **cb057_2_4_ : Province**

	No	%
01	1	4.00
05	1	4.00
08	2	8.00
09	2	8.00
10	1	4.00
11	4	16.00
12	2	8.00
15	3	12.00
16	1	4.00
20	1	4.00
24	1	4.00
32	2	8.00
33	1	4.00
34	3	12.00
Total	25	100.00

- **cb057_2_5_ : Province**

	No	%
01	1	9.09

05	3	27.27
09	2	18.18
12	2	18.18
15	1	9.09
16	1	9.09
26	1	9.09
Total	11	100.00

- **cb057_2_6_ : Province**

	No	%
10	1	33.33
17	2	66.67
Total	3	100.00

- **cb057_2_8_ : Province**

	No	%
15	1	100.00
Total	1	100.00

- **cb057_3_ : The Current Hukou Location of this Child**

	No	%
1 This Household	547	45.58
2 This village/Neighborhood	256	21.33
3 Another Village/Neighborhood in this County/City	312	26.00
4 Another County/City in this Province	48	4.00
5 Another Province	35	2.92
6 Other	2	0.17
Total	1,200	100.00

- **cb057_4_ : The Current Hukou Location of this Child**

	No	%
1 This Household	262	39.46
2 This village/Neighborhood	136	20.48
3 Another Village/Neighborhood in this County/City	211	31.78
4 Another County/City in this Province	29	4.37
5 Another Province	24	3.61
6 Other	2	0.30
Total	664	100.00

- **cb057_5_ : The Current Hukou Location of this Child**

	No	%
1 This Household	89	28.90
2 This village/Neighborhood	69	22.40
3 Another Village/Neighborhood in this County/City	114	37.01
4 Another County/City in this Province	24	7.79
5 Another Province	11	3.57
6 Other	1	0.32
Total	308	100.00

- **cb057_6_ : The Current Hukou Location of this Child**

	No	%
1 This Household	35	30.43
2 This village/Neighborhood	30	26.09
3 Another Village/Neighborhood in this County/City	44	38.26
4 Another County/City in this Province	3	2.61
5 Another Province	3	2.61
Total	115	100.00

- **cb057_7_ : The Current Hukou Location of this Child**

	No	%
1 This Household	13	36.11
2 This village/Neighborhood	8	22.22
3 Another Village/Neighborhood in this County/City	14	38.89
4 Another County/City in this Province	1	2.78
Total	36	100.00

- **cb057_8_ : The Current Hukou Location of this Child**

	No	%
1 This Household	3	23.08
2 This village/Neighborhood	2	15.38
3 Another Village/Neighborhood in this County/City	6	46.15
4 Another County/City in this Province	1	7.69
5 Another Province	1	7.69
Total	13	100.00

- **cb057_9_ : The Current Hukou Location of this Child**

	No	%
1 This Household	2	33.33

3 Another Village/Neighborhood in this County/City	4	66.67
Total	6	100.00

- **cb058_1_ : Is this Child Still in School Now**

	No	%
1 Yes	738	7.62
2 No	8,953	92.38
Total	9,691	100.00

- **cb058_2_ : Is this Child Still in School Now**

	No	%
1 Yes	497	6.18
2 No	7,547	93.82
Total	8,044	100.00

- **cb058_3_ : Is this Child Still in School Now**

	No	%
1 Yes	133	2.80
2 No	4,611	97.20
Total	4,744	100.00

- **cb058_4_ : Is this Child Still in School Now**

	No	%
1 Yes	31	1.23
2 No	2,494	98.77
Total	2,525	100.00

- **cb058_5_ : Is this Child Still in School Now**

	No	%
1 Yes	10	0.82
2 No	1,207	99.18
Total	1,217	100.00

- **cb058_6_ : Is this Child Still in School Now**

	No	%

1 Yes	2	0.40
2 No	493	99.60
Total	495	100.00

- **cb058_7_ : Is this Child Still in School Now**

	No	%
2 No	186	100.00
Total	186	100.00

- **cb058_8_ : Is this Child Still in School Now**

	No	%
1 Yes	1	1.64
2 No	60	98.36
Total	61	100.00

- **cb058_9_ : Is this Child Still in School Now**

	No	%
1 Yes	1	4.55
2 No	21	95.45
Total	22	100.00

- **cb058_10_ : Is this Child Still in School Now**

	No	%
2 No	6	100.00
Total	6	100.00

- **cb058_11_ : Is this Child Still in School Now**

No Observations

- **cb058_12_ : Is this Child Still in School Now**

No Observations

- **cb058_13_ : Is this Child Still in School Now**

No Observations

- **cb058_14_ : Is this Child Still in School Now**

No Observations

- **cb059_1_ : Level of Schooling this Child Currently Enrolled in**

	No	%
1 Primary school grade 1	23	3.08
2 Primary school grade 2	15	2.01
3 Primary school grade 3	12	1.61
4 Primary school grade 4	12	1.61
5 Primary school grade 5	20	2.68
6 Primary school grade 6	30	4.02
7 Middle school grade 1	37	4.95
8 Middle school grade 2	43	5.76
9 Middle school grade 3	53	7.10
10 Middle school grade 4	2	0.27
11 High school, grade 1	39	5.22
12 High school, grade 2	48	6.43
13 High school, grade 3	79	10.58
14 Vocational/technical high school year 1	10	1.34
15 Vocational/technical high school year 2	9	1.20
16 Vocational/technical high school year 3	10	1.34
17 College year 1	57	7.63
18 College year 2	76	10.17
19 College year 3	90	12.05
20 College year 4	40	5.35
21 College year 5	3	0.40
23 Masters degree	34	4.55
24 Doctoral degree/Ph.D. degree	5	0.67
Total	747	100.00

- **cb059_2_ : Level of Schooling this Child Currently Enrolled in**

	No	%
1 Primary school grade 1	25	4.96
2 Primary school grade 2	18	3.57
3 Primary school grade 3	6	1.19
4 Primary school grade 4	17	3.37
5 Primary school grade 5	17	3.37

6 Primary school grade 6	19	3.77
7 Middle school grade 1	29	5.75
8 Middle school grade 2	29	5.75
9 Middle school grade 3	32	6.35
10 Middle school grade 4	1	0.20
11 High school, grade 1	27	5.36
12 High school, grade 2	26	5.16
13 High school, grade 3	42	8.33
14 Vocational/technical high school year 1	8	1.59
15 Vocational/technical high school year 2	10	1.98
16 Vocational/technical high school year 3	12	2.38
17 College year 1	39	7.74
18 College year 2	55	10.91
19 College year 3	56	11.11
20 College year 4	20	3.97
21 College year 5	1	0.20
23 Masters degree	13	2.58
24 Doctoral degree/Ph.D. degree	2	0.40
Total	504	100.00

- **cb059_3_ : Level of Schooling this Child Currently Enrolled in**

	No	%
1 Primary school grade 1	4	3.01
2 Primary school grade 2	5	3.76
3 Primary school grade 3	6	4.51
4 Primary school grade 4	5	3.76
5 Primary school grade 5	6	4.51
6 Primary school grade 6	4	3.01
7 Middle school grade 1	8	6.02
8 Middle school grade 2	6	4.51
9 Middle school grade 3	7	5.26
11 High school, grade 1	5	3.76
12 High school, grade 2	5	3.76
13 High school, grade 3	10	7.52
15 Vocational/technical high school year 2	2	1.50
16 Vocational/technical high school year 3	3	2.26
17 College year 1	14	10.53
18 College year 2	18	13.53
19 College year 3	15	11.28
20 College year 4	1	0.75
21 College year 5	3	2.26
22 College year 6	1	0.75
23 Masters degree	4	3.01
24 Doctoral degree/Ph.D. degree	1	0.75
Total	133	100.00

- **cb059_4_ : Level of Schooling this Child Currently Enrolled in**

	No	%
1 Primary school grade 1	2	6.90
2 Primary school grade 2	2	6.90
3 Primary school grade 3	3	10.34
4 Primary school grade 4	1	3.45
5 Primary school grade 5	1	3.45
7 Middle school grade 1	2	6.90
8 Middle school grade 2	4	13.79
11 High school, grade 1	1	3.45
13 High school, grade 3	2	6.90
15 Vocational/technical high school year 2	2	6.90
17 College year 1	3	10.34
18 College year 2	1	3.45
19 College year 3	2	6.90
23 Masters degree	2	6.90
24 Doctoral degree/Ph.D. degree	1	3.45
Total	29	100.00

- **cb059_5_ : Level of Schooling this Child Currently Enrolled in**

	No	%
4 Primary school grade 4	1	10.00
6 Primary school grade 6	1	10.00
7 Middle school grade 1	2	20.00
8 Middle school grade 2	1	10.00
12 High school, grade 2	1	10.00
17 College year 1	1	10.00
18 College year 2	1	10.00
20 College year 4	2	20.00
Total	10	100.00

- **cb059_6_ : Level of Schooling this Child Currently Enrolled in**

	No	%
6 Primary school grade 6	1	50.00
13 High school, grade 3	1	50.00
Total	2	100.00

- **cb059_8_ : Level of Schooling this Child Currently Enrolled in**

	No	%
3 Primary school grade 3	1	100.00
Total	1	100.00

- **cb059_9_ : Level of Schooling this Child Currently Enrolled in**

	No	%
1 Primary school grade 1	1	100.00
Total	1	100.00

- **cb060_1_ : Highest Level of Education this Child Completed**

	No	%
1 No formal education illiterate	343	3.84
2 Did not finish primary school but capable of reading or writing	686	7.67
3 Sishu/home school	4	0.04
4 Elementary school	1,823	20.39
5 Middle school	3,230	36.12
6 High school	870	9.73
7 Vocational school	668	7.47
8 Two-/Three-Year College / Associate degree	656	7.34
9 Four-Year College / Bachelors degree	599	6.70
10 Post-graduate, Masters degree	55	0.62
11 Post-graduate, doctoral degree/Ph.D.	8	0.09
Total	8,942	100.00

- **cb060_2_ : Highest Level of Education this Child Completed**

	No	%
1 No formal education illiterate	355	4.73
2 Did not finish primary school but capable of reading or writing	705	9.40
3 Sishu/home school	6	0.08
4 Elementary school	1,699	22.64
5 Middle school	2,740	36.52
6 High school	673	8.97
7 Vocational school	479	6.38
8 Two-/Three-Year College / Associate degree	395	5.26
9 Four-Year College / Bachelors degree	410	5.46
10 Post-graduate, Masters degree	34	0.45
11 Post-graduate, doctoral degree/Ph.D.	7	0.09
Total	7,503	100.00

- **cb060_3_ : Highest Level of Education this Child Completed**

	No	%
1 No formal education illiterate	358	7.83
2 Did not finish primary school but capable of reading or writing	522	11.41
4 Elementary school	1,221	26.70
5 Middle school	1,575	34.44
6 High school	360	7.87
7 Vocational school	200	4.37

8 Two-/Three-Year College / Associate degree	179	3.91
9 Four-Year College / Bachelors degree	140	3.06
10 Post-graduate, Masters degree	14	0.31
11 Post-graduate, doctoral degree/Ph.D.	4	0.09
Total	4,573	100.00

- **cb060_4_ : Highest Level of Education this Child Completed**

	No	%
1 No formal education illiterate	285	11.52
2 Did not finish primary school but capable of reading or writing	354	14.31
4 Elementary school	713	28.82
5 Middle school	734	29.67
6 High school	159	6.43
7 Vocational school	101	4.08
8 Two-/Three-Year College / Associate degree	66	2.67
9 Four-Year College / Bachelors degree	57	2.30
10 Post-graduate, Masters degree	5	0.20
Total	2,474	100.00

- **cb060_5_ : Highest Level of Education this Child Completed**

	No	%
1 No formal education illiterate	202	16.92
2 Did not finish primary school but capable of reading or writing	174	14.57
3 Sishu/home school	1	0.08
4 Elementary school	350	29.31
5 Middle school	326	27.30
6 High school	60	5.03
7 Vocational school	27	2.26
8 Two-/Three-Year College / Associate degree	19	1.59
9 Four-Year College / Bachelors degree	30	2.51
10 Post-graduate, Masters degree	4	0.34
11 Post-graduate, doctoral degree/Ph.D.	1	0.08
Total	1,194	100.00

- **cb060_6_ : Highest Level of Education this Child Completed**

	No	%
1 No formal education illiterate	96	19.79
2 Did not finish primary school but capable of reading or writing	79	16.29
3 Sishu/home school	1	0.21
4 Elementary school	124	25.57
5 Middle school	143	29.48
6 High school	22	4.54
7 Vocational school	10	2.06
8 Two-/Three-Year College / Associate degree	4	0.82

9 Four-Year College / Bachelors degree	5	1.03
10 Post-graduate, Masters degree	1	0.21
Total	485	100.00

- **cb060_7_ : Highest Level of Education this Child Completed**

	No	%
1 No formal education illiterate	47	25.68
2 Did not finish primary school but capable of reading or writing	28	15.30
4 Elementary school	44	24.04
5 Middle school	41	22.40
6 High school	9	4.92
7 Vocational school	8	4.37
8 Two-/Three-Year College / Associate degree	5	2.73
9 Four-Year College / Bachelors degree	1	0.55
Total	183	100.00

- **cb060_8_ : Highest Level of Education this Child Completed**

	No	%
1 No formal education illiterate	11	18.97
2 Did not finish primary school but capable of reading or writing	10	17.24
4 Elementary school	14	24.14
5 Middle school	14	24.14
6 High school	6	10.34
7 Vocational school	1	1.72
9 Four-Year College / Bachelors degree	2	3.45
Total	58	100.00

- **cb060_9_ : Highest Level of Education this Child Completed**

	No	%
1 No formal education illiterate	3	15.00
2 Did not finish primary school but capable of reading or writing	5	25.00
4 Elementary school	3	15.00
5 Middle school	7	35.00
6 High school	1	5.00
9 Four-Year College / Bachelors degree	1	5.00
Total	20	100.00

- **cb060_10_ : Highest Level of Education this Child Completed**

	No	%
1 No formal education illiterate	1	20.00
2 Did not finish primary school but capable of reading or writing	1	20.00

5 Middle school	1	20.00
7 Vocational school	1	20.00
9 Four-Year College / Bachelors degree	1	20.00
Total	5	100.00

- **cb061_1_ : Years this Child Spend in Primary School**

	No	%
0	8	2.18
1	30	8.17
2	64	17.44
3	91	24.80
4	96	26.16
5	66	17.98
6	7	1.91
7	3	0.82
8	1	0.27
9	1	0.27
Total	367	100.00

- **cb061_2_ : Years this Child Spend in Primary School**

	No	%
0	9	1.40
1	46	7.18
2	126	19.66
3	181	28.24
4	154	24.02
5	111	17.32
6	10	1.56
7	2	0.31
8	2	0.31
Total	641	100.00

- **cb061_3_ : Years this Child Spend in Primary School**

	No	%
0	8	1.57
1	42	8.27
2	84	16.54
3	163	32.09
4	124	24.41
5	75	14.76
6	6	1.18
7	4	0.79
8	2	0.39
Total	508	100.00

- **cb061_4_ : Years this Child Spend in Primary School**

	No	%
0	14	4.08
1	30	8.75
2	76	22.16
3	99	28.86
4	79	23.03
5	40	11.66
6	3	0.87
7	1	0.29
8	1	0.29
Total	343	100.00

- **cb061_5_ : Years this Child Spend in Primary School**

	No	%
0	7	4.07
1	13	7.56
2	47	27.33
3	53	30.81
4	35	20.35
5	15	8.72
6	2	1.16
Total	172	100.00

- **cb061_6_ : Years this Child Spend in Primary School**

	No	%
0	2	2.56
1	4	5.13
2	23	29.49
3	23	29.49
4	13	16.67
5	11	14.10
6	2	2.56
Total	78	100.00

- **cb061_7_ : Years this Child Spend in Primary School**

	No	%
0	1	3.70
1	2	7.41
2	4	14.81

3	11	40.74
4	5	18.52
5	4	14.81
Total	27	100.00

- **cb061_8_ : Years this Child Spend in Primary School**

	No	%
3	4	40.00
4	5	50.00
5	1	10.00
Total	10	100.00

- **cb061_9_ : Years this Child Spend in Primary School**

	No	%
2	1	25.00
4	2	50.00
5	1	25.00
Total	4	100.00

- **cb061_10_ : Years this Child Spend in Primary School**

No Observations

- **cb062_1_ : Additional Years this Child Receive after the Answer in cb060**

	No	%
0	3,116	89.18
1	166	4.75
2	151	4.32
3	50	1.43
4	5	0.14
5	2	0.06
6	4	0.11
Total	3,494	100.00

- **cb062_2_ : Additional Years this Child Receive after the Answer in cb060**

	No	%
0	4,686	89.43
1	219	4.18

2	255	4.87
3	67	1.28
4	6	0.11
5	3	0.06
6	2	0.04
7	1	0.02
8	1	0.02
Total	5,240	100.00

- **cb062_3_ : Additional Years this Child Receive after the Answer in cb060**

	No	%
0	3,041	88.40
1	163	4.74
2	177	5.15
3	47	1.37
4	7	0.20
5	3	0.09
7	1	0.03
8	1	0.03
Total	3,440	100.00

- **cb062_4_ : Additional Years this Child Receive after the Answer in cb060**

	No	%
0	1,620	90.55
1	70	3.91
2	73	4.08
3	21	1.17
4	3	0.17
5	1	0.06
8	1	0.06
Total	1,789	100.00

- **cb062_5_ : Additional Years this Child Receive after the Answer in cb060**

	No	%
0	740	91.25
1	30	3.70
2	31	3.82
3	9	1.11
5	1	0.12
Total	811	100.00

- **cb062_6_ : Additional Years this Child Receive after the Answer in cb060**

	No	%
0	288	92.90
1	13	4.19
2	7	2.26
3	1	0.32
4	1	0.32
Total	310	100.00

- **cb062_7_ : Additional Years this Child Receive after the Answer in cb060**

	No	%
0	104	96.30
1	1	0.93
2	2	1.85
3	1	0.93
Total	108	100.00

- **cb062_8_ : Additional Years this Child Receive after the Answer in cb060**

	No	%
0	35	94.59
2	1	2.70
3	1	2.70
Total	37	100.00

- **cb062_9_ : Additional Years this Child Receive after the Answer in cb060**

	No	%
0	12	100.00
Total	12	100.00

- **cb062_10_ : Additional Years this Child Receive after the Answer in cb060**

	No	%
0	3	100.00
Total	3	100.00

- **cb063_1_ : Marital Status of this Child**

	No	%

1 Married with spouse present or living with a partner as if they were married	6,140	64.99
2 Married but not living with spouse temporarily for reasons such as work	494	5.23
3 Separated	37	0.39
4 Divorced	208	2.20
5 Widowed	99	1.05
6 Never married	2,469	26.14
Total	9,447	100.00

- **cb063_2_ : Marital Status of this Child**

	No	%
1 Married with spouse present or living with a partner as if they were married	5,650	71.95
2 Married but not living with spouse temporarily for reasons such as work	302	3.85
3 Separated	25	0.32
4 Divorced	125	1.59
5 Widowed	60	0.76
6 Never married	1,691	21.53
Total	7,853	100.00

- **cb063_3_ : Marital Status of this Child**

	No	%
1 Married with spouse present or living with a partner as if they were married	3,799	81.38
2 Married but not living with spouse temporarily for reasons such as work	182	3.90
3 Separated	10	0.21
4 Divorced	62	1.33
5 Widowed	35	0.75
6 Never married	580	12.43
Total	4,668	100.00

- **cb063_4_ : Marital Status of this Child**

	No	%
1 Married with spouse present or living with a partner as if they were married	2,148	85.89
2 Married but not living with spouse temporarily for reasons such as work	104	4.16
3 Separated	2	0.08
4 Divorced	44	1.76
5 Widowed	22	0.88
6 Never married	181	7.24
Total	2,501	100.00

- **cb063_5_ : Marital Status of this Child**

	No	%
1 Married with spouse present or living with a partner as if they were married	1,064	88.15

	No	%
2 Married but not living with spouse temporarily for reasons such as work	49	4.06
3 Separated	5	0.41
4 Divorced	13	1.08
5 Widowed	16	1.33
6 Never married	60	4.97
Total	1,207	100.00

- **cb063_6_ : Marital Status of this Child**

	No	%
1 Married with spouse present or living with a partner as if they were married	436	88.80
2 Married but not living with spouse temporarily for reasons such as work	20	4.07
4 Divorced	7	1.43
5 Widowed	5	1.02
6 Never married	23	4.68
Total	491	100.00

- **cb063_7_ : Marital Status of this Child**

	No	%
1 Married with spouse present or living with a partner as if they were married	171	91.94
2 Married but not living with spouse temporarily for reasons such as work	4	2.15
3 Separated	3	1.61
4 Divorced	1	0.54
6 Never married	7	3.76
Total	186	100.00

- **cb063_8_ : Marital Status of this Child**

	No	%
1 Married with spouse present or living with a partner as if they were married	54	90.00
5 Widowed	2	3.33
6 Never married	4	6.67
Total	60	100.00

- **cb063_9_ : Marital Status of this Child**

	No	%
1 Married with spouse present or living with a partner as if they were married	19	90.48
6 Never married	2	9.52
Total	21	100.00

- **cb063_10_ : Marital Status of this Child**

	No	%
1 Married with spouse present or living with a partner as if they were married	5	100.00
Total	5	100.00

- **cb063_11_ : Marital Status of this Child**

No Observations

- **cb063_12_ : Marital Status of this Child**

No Observations

- **cb063_13_ : Marital Status of this Child**

No Observations

- **cb063_14_ : Marital Status of this Child**

No Observations

- **cb064_1_ : Money Spend to Support this Child's College Education**

Mean	Min	Max	OBS
41,356.4	0.0	600,000.0	1,530

- **cb064_2_ : Money Spend to Support this Child's College Education**

Mean	Min	Max	OBS
39,533.6	0.0	600,000.0	971

- **cb064_3_ : Money Spend to Support this Child's College Education**

Mean	Min	Max	OBS

31,102.7	0.0	400,000.0	354

- **cb064_4_ : Money Spend to Support this Child's College Education**

Mean	Min	Max	OBS
26,884.7	0.0	100,000.0	115

- **cb064_5_ : Money Spend to Support this Child's College Education**

Mean	Min	Max	OBS
23,971.2	0.0	150,000.0	50

- **cb064_6_ : Money Spend to Support this Child's College Education**

Mean	Min	Max	OBS
28,466.7	0.0	80,000.0	6

- **cb064_7_ : Money Spend to Support this Child's College Education**

Mean	Min	Max	OBS
10,000.0	0.0	30,000.0	3

- **cb064_8_ : Money Spend to Support this Child's College Education**

No Observations

- **cb064_9_ : Money Spend to Support this Child's College Education**

Mean	Min	Max	OBS
10,000.0	10,000.0	10,000.0	1

- **cb064_10_ : Money Spend to Support this Child's College Education**

No Observations

- **cb065_1_ : Num. of Sons this Child has**

	No	%
0	2,416	34.09
1	3,765	53.13
2	826	11.66
3	69	0.97
4	8	0.11
5	3	0.04
Total	7,087	100.00

- **cb065_2_ : Num. of Sons this Child has**

	No	%
0	2,065	33.38
1	3,396	54.90
2	664	10.73
3	56	0.91
4	4	0.06
6	1	0.02
Total	6,186	100.00

- **cb065_3_ : Num. of Sons this Child has**

	No	%
0	1,205	29.38
1	2,327	56.74
2	523	12.75
3	40	0.98
4	5	0.12
5	1	0.02
Total	4,101	100.00

- **cb065_4_ : Num. of Sons this Child has**

	No	%
0	588	25.34
1	1,364	58.79
2	339	14.61
3	26	1.12
4	3	0.13
Total	2,320	100.00

- **cb065_5_ : Num. of Sons this Child has**

	No	%

0	280	24.31
1	703	61.02
2	153	13.28
3	16	1.39
Total	1,152	100.00

- **cb065_6_ : Num. of Sons this Child has**

	No	%
0	91	19.40
1	292	62.26
2	75	15.99
3	9	1.92
4	2	0.43
Total	469	100.00

- **cb065_7_ : Num. of Sons this Child has**

	No	%
0	42	23.33
1	110	61.11
2	25	13.89
3	3	1.67
Total	180	100.00

- **cb065_8_ : Num. of Sons this Child has**

	No	%
0	10	17.86
1	36	64.29
2	9	16.07
3	1	1.79
Total	56	100.00

- **cb065_9_ : Num. of Sons this Child has**

	No	%
0	3	16.67
1	12	66.67
2	2	11.11
3	1	5.56
Total	18	100.00

- **cb065_10_ : Num. of Sons this Child has**

	No	%
0	1	20.00
1	4	80.00
Total	5	100.00

- **cb065_11_ : Num. of Sons this Child has**

	No	%
0	1	100.00
Total	1	100.00

- **cb065_12_ : Num. of Sons this Child has**

No Observations

- **cb065_13_ : Num. of Sons this Child has**

No Observations

- **cb065_14_ : Num. of Sons this Child has**

No Observations

- **cb066_1_ : Num. of Adult Sons this Child has**

	No	%
0	2,969	62.64
1	1,357	28.63
2	359	7.57
3	47	0.99
4	6	0.13
5	2	0.04
Total	4,740	100.00

- **cb066_2_ : Num. of Adult Sons this Child has**

	No	%
0	2,479	59.78
1	1,290	31.11
2	334	8.05
3	40	0.96
4	3	0.07
6	1	0.02
Total	4,147	100.00

- **cb066_3_ : Num. of Adult Sons this Child has**

	No	%
0	1,665	57.26
1	973	33.46
2	237	8.15
3	29	1.00
4	3	0.10
5	1	0.03
Total	2,908	100.00

- **cb066_4_ : Num. of Adult Sons this Child has**

	No	%
0	942	54.26
1	622	35.83
2	154	8.87
3	17	0.98
4	1	0.06
Total	1,736	100.00

- **cb066_5_ : Num. of Adult Sons this Child has**

	No	%
0	425	48.68
1	370	42.38
2	69	7.90
3	9	1.03
Total	873	100.00

- **cb066_6_ : Num. of Adult Sons this Child has**

	No	%
0	184	48.42

1	149	39.21
2	38	10.00
3	7	1.84
4	2	0.53
Total	380	100.00

- **cb066_7_ : Num. of Adult Sons this Child has**

	No	%
0	59	42.75
1	67	48.55
2	9	6.52
3	3	2.17
Total	138	100.00

- **cb066_8_ : Num. of Adult Sons this Child has**

	No	%
0	21	45.65
1	21	45.65
2	3	6.52
3	1	2.17
Total	46	100.00

- **cb066_9_ : Num. of Adult Sons this Child has**

	No	%
0	9	60.00
1	4	26.67
2	1	6.67
3	1	6.67
Total	15	100.00

- **cb066_10_ : Num. of Adult Sons this Child has**

	No	%
0	1	25.00
1	3	75.00
Total	4	100.00

- **cb066_23_ : Num. of Adult Sons this Child has**

	No	%

0	1	100.00
Total	1	100.00

- **cb067_1_ : Num. of Daughters this Child has**

	No	%
0	3,306	46.69
1	3,009	42.50
2	654	9.24
3	86	1.21
4	18	0.25
5	5	0.07
6	1	0.01
8	1	0.01
Total	7,080	100.00

- **cb067_2_ : Num. of Daughters this Child has**

	No	%
0	2,732	44.18
1	2,699	43.64
2	672	10.87
3	64	1.03
4	16	0.26
5	1	0.02
Total	6,184	100.00

- **cb067_3_ : Num. of Daughters this Child has**

	No	%
0	1,734	42.27
1	1,828	44.56
2	478	11.65
3	50	1.22
4	9	0.22
5	2	0.05
6	1	0.02
Total	4,102	100.00

- **cb067_4_ : Num. of Daughters this Child has**

	No	%
0	923	39.75
1	1,051	45.26
2	284	12.23

3	47	2.02
4	15	0.65
5	1	0.04
7	1	0.04
Total	2,322	100.00

- **cb067_5_ : Num. of Daughters this Child has**

	No	%
0	429	37.30
1	538	46.78
2	154	13.39
3	24	2.09
4	3	0.26
5	1	0.09
6	1	0.09
Total	1,150	100.00

- **cb067_6_ : Num. of Daughters this Child has**

	No	%
0	175	37.23
1	213	45.32
2	71	15.11
3	7	1.49
4	3	0.64
5	1	0.21
Total	470	100.00

- **cb067_7_ : Num. of Daughters this Child has**

	No	%
0	59	33.15
1	93	52.25
2	21	11.80
3	5	2.81
Total	178	100.00

- **cb067_8_ : Num. of Daughters this Child has**

	No	%
0	22	38.60
1	27	47.37
2	7	12.28
3	1	1.75

Total	57	100.00

- **cb067_9_ : Num. of Daughters this Child has**

	No	%
0	10	55.56
1	7	38.89
2	1	5.56
Total	18	100.00

- **cb067_10_ : Num. of Daughters this Child has**

	No	%
0	2	40.00
1	2	40.00
2	1	20.00
Total	5	100.00

- **cb067_11_ : Num. of Daughters this Child has**

No Observations

- **cb067_12_ : Num. of Daughters this Child has**

No Observations

- **cb067_13_ : Num. of Daughters this Child has**

	No	%
0	1	100.00
Total	1	100.00

- **cb067_14_ : Num. of Daughters this Child has**

No Observations

- **cb067_23_ : Num. of Daughters this Child has**

	No	%
0	1	100.00
Total	1	100.00

- **cb068_1_ : Num. of Adult Daughters this Child has**

	No	%
0	2,310	60.77
1	1,163	30.60
2	261	6.87
3	49	1.29
4	12	0.32
5	4	0.11
6	1	0.03
8	1	0.03
Total	3,801	100.00

- **cb068_2_ : Num. of Adult Daughters this Child has**

	No	%
0	2,007	58.07
1	1,097	31.74
2	300	8.68
3	40	1.16
4	11	0.32
5	1	0.03
Total	3,456	100.00

- **cb068_3_ : Num. of Adult Daughters this Child has**

	No	%
0	1,308	55.31
1	843	35.64
2	179	7.57
3	26	1.10
4	8	0.34
5	1	0.04
Total	2,365	100.00

- **cb068_4_ : Num. of Adult Daughters this Child has**

	No	%
0	718	51.51

1	511	36.66
2	132	9.47
3	22	1.58
4	10	0.72
5	1	0.07
Total	1,394	100.00

- **cb068_5_ : Num. of Adult Daughters this Child has**

	No	%
0	324	45.06
1	294	40.89
2	84	11.68
3	14	1.95
4	3	0.42
Total	719	100.00

- **cb068_6_ : Num. of Adult Daughters this Child has**

	No	%
0	120	41.10
1	125	42.81
2	41	14.04
3	3	1.03
4	3	1.03
Total	292	100.00

- **cb068_7_ : Num. of Adult Daughters this Child has**

	No	%
0	50	42.02
1	59	49.58
2	9	7.56
3	1	0.84
Total	119	100.00

- **cb068_8_ : Num. of Adult Daughters this Child has**

	No	%
0	13	37.14
1	18	51.43
2	3	8.57
3	1	2.86
Total	35	100.00

- **cb068_9_ : Num. of Adult Daughters this Child has**

	No	%
0	4	50.00
1	4	50.00
Total	8	100.00

- **cb068_10_ : Num. of Adult Daughters this Child has**

	No	%
0	1	33.33
1	1	33.33
2	1	33.33
Total	3	100.00

- **cb069_1_ : The Total Income of this Child and His/Her Spouse Last Year**

	No	%
1 None	1,240	14.79
2 Under 2,000 yuan	211	2.52
3 2,000-5,000 yuan	588	7.02
4 5,000-10,000 yuan	1,122	13.39
5 10,000 - 20,000 yuan	2,439	29.10
6 20,000 - 50,000 yuan	2,194	26.18
7 50,000 - 100,000 yuan	433	5.17
8 100,000 - 150,000 yuan	107	1.28
9 150,000 - 200,000 yuan	15	0.18
10 200,000 - 300,000 yuan	13	0.16
11 Above 300,000 yuan	20	0.24
Total	8,382	100.00

- **cb069_2_ : The Total Income of this Child and His/Her Spouse Last Year**

	No	%
1 None	775	11.71
2 Under 2,000 yuan	147	2.22
3 2,000-5,000 yuan	440	6.65
4 5,000-10,000 yuan	878	13.26
5 10,000 - 20,000 yuan	2,053	31.01
6 20,000 - 50,000 yuan	1,846	27.89
7 50,000 - 100,000 yuan	353	5.33
8 100,000 - 150,000 yuan	76	1.15
9 150,000 - 200,000 yuan	21	0.32
10 200,000 - 300,000 yuan	11	0.17
11 Above 300,000 yuan	20	0.30
Total	6,620	100.00

- **cb069_3_ : The Total Income of this Child and His/Her Spouse Last Year**

	No	%
1 None	265	7.13
2 Under 2,000 yuan	86	2.31
3 2,000-5,000 yuan	256	6.89
4 5,000-10,000 yuan	528	14.20
5 10,000 - 20,000 yuan	1,206	32.44
6 20,000 - 50,000 yuan	1,080	29.05
7 50,000 - 100,000 yuan	215	5.78
8 100,000 - 150,000 yuan	62	1.67
9 150,000 - 200,000 yuan	6	0.16
10 200,000 - 300,000 yuan	6	0.16
11 Above 300,000 yuan	8	0.22
Total	3,718	100.00

- **cb069_4_ : The Total Income of this Child and His/Her Spouse Last Year**

	No	%
1 None	95	4.97
2 Under 2,000 yuan	49	2.56
3 2,000-5,000 yuan	139	7.27
4 5,000-10,000 yuan	273	14.27
5 10,000 - 20,000 yuan	637	33.30
6 20,000 - 50,000 yuan	574	30.01
7 50,000 - 100,000 yuan	108	5.65
8 100,000 - 150,000 yuan	25	1.31
9 150,000 - 200,000 yuan	2	0.10
10 200,000 - 300,000 yuan	6	0.31
11 Above 300,000 yuan	5	0.26
Total	1,913	100.00

- **cb069_5_ : The Total Income of this Child and His/Her Spouse Last Year**

	No	%
1 None	44	4.95
2 Under 2,000 yuan	29	3.27
3 2,000-5,000 yuan	57	6.42
4 5,000-10,000 yuan	142	15.99
5 10,000 - 20,000 yuan	293	33.00
6 20,000 - 50,000 yuan	262	29.50
7 50,000 - 100,000 yuan	49	5.52
8 100,000 - 150,000 yuan	7	0.79
9 150,000 - 200,000 yuan	3	0.34
11 Above 300,000 yuan	2	0.23
Total	888	100.00

- **cb069_6_ : The Total Income of this Child and His/Her Spouse Last Year**

	No	%
1 None	16	4.49
2 Under 2,000 yuan	8	2.25
3 2,000-5,000 yuan	30	8.43
4 5,000-10,000 yuan	45	12.64
5 10,000 - 20,000 yuan	139	39.04
6 20,000 - 50,000 yuan	97	27.25
7 50,000 - 100,000 yuan	15	4.21
8 100,000 - 150,000 yuan	4	1.12
9 150,000 - 200,000 yuan	1	0.28
11 Above 300,000 yuan	1	0.28
Total	356	100.00

- **cb069_7_ : The Total Income of this Child and His/Her Spouse Last Year**

	No	%
1 None	6	4.32
2 Under 2,000 yuan	2	1.44
3 2,000-5,000 yuan	14	10.07
4 5,000-10,000 yuan	24	17.27
5 10,000 - 20,000 yuan	48	34.53
6 20,000 - 50,000 yuan	34	24.46
7 50,000 - 100,000 yuan	4	2.88
8 100,000 - 150,000 yuan	4	2.88
10 200,000 - 300,000 yuan	3	2.16
Total	139	100.00

- **cb069_8_ : The Total Income of this Child and His/Her Spouse Last Year**

	No	%
1 None	2	4.65
2 Under 2,000 yuan	2	4.65
3 2,000-5,000 yuan	2	4.65
4 5,000-10,000 yuan	8	18.60
5 10,000 - 20,000 yuan	12	27.91
6 20,000 - 50,000 yuan	12	27.91
7 50,000 - 100,000 yuan	5	11.63
Total	43	100.00

- **cb069_9_ : The Total Income of this Child and His/Her Spouse Last Year**

	No	%
2 Under 2,000 yuan	2	13.33
3 2,000-5,000 yuan	1	6.67

4 5,000-10,000 yuan	2	13.33
5 10,000 - 20,000 yuan	5	33.33
6 20,000 - 50,000 yuan	3	20.00
7 50,000 - 100,000 yuan	1	6.67
8 100,000 - 150,000 yuan	1	6.67
Total	15	100.00

- **cb069_10_ : The Total Income of this Child and His/Her Spouse Last Year**

	No	%
1 None	1	25.00
5 10,000 - 20,000 yuan	1	25.00
7 50,000 - 100,000 yuan	2	50.00
Total	4	100.00

- **cb069_11_ : The Total Income of this Child and His/Her Spouse Last Year**

No Observations

- **cb069_12_ : The Total Income of this Child and His/Her Spouse Last Year**

No Observations

- **cb069_13_ : The Total Income of this Child and His/Her Spouse Last Year**

No Observations

- **cb069_14_ : The Total Income of this Child and His/Her Spouse Last Year**

No Observations

- **cb069_15_ : The Total Income of this Child and His/Her Spouse Last**

Year

	No	%
5 10,000 - 20,000 yuan	1	100.00
Total	1	100.00

- **cb069_23_ : The Total Income of this Child and His/Her Spouse Last Year**

	No	%
5 10,000 - 20,000 yuan	1	100.00
Total	1	100.00

- **cb069_25_ : The Total Income of this Child and His/Her Spouse Last Year**

	No	%
2 Under 2,000 yuan	1	100.00
Total	1	100.00

- **cb070_1_ : IS this Child Working Now**

	No	%
1 Yes	7,790	81.78
2 No	1,736	18.22
Total	9,526	100.00

- **cb070_2_ : IS this Child Working Now**

	No	%
1 Yes	6,488	82.49
2 No	1,377	17.51
Total	7,865	100.00

- **cb070_3_ : IS this Child Working Now**

	No	%
1 Yes	3,946	84.44
2 No	727	15.56
Total	4,673	100.00

- **cb070_4_ : IS this Child Working Now**

	No	%
1 Yes	2,162	86.55
2 No	336	13.45
Total	2,498	100.00

- **cb070_5_ : IS this Child Working Now**

	No	%
1 Yes	1,053	87.39
2 No	152	12.61
Total	1,205	100.00

- **cb070_6_ : IS this Child Working Now**

	No	%
1 Yes	426	86.76
2 No	65	13.24
Total	491	100.00

- **cb070_7_ : IS this Child Working Now**

	No	%
1 Yes	160	87.43
2 No	23	12.57
Total	183	100.00

- **cb070_8_ : IS this Child Working Now**

	No	%
1 Yes	51	85.00
2 No	9	15.00
Total	60	100.00

- **cb070_9_ : IS this Child Working Now**

	No	%
1 Yes	20	90.91
2 No	2	9.09
Total	22	100.00

- **cb070_10_ : IS this Child Working Now**

	No	%
1 Yes	5	83.33
2 No	1	16.67
Total	6	100.00

- **cb070_11_ : IS this Child Working Now**

	No	%
1 Yes	2	100.00
Total	2	100.00

- **cb070_12_ : IS this Child Working Now**

	No	%
1 Yes	2	66.67
2 No	1	33.33
Total	3	100.00

- **cb070_13_ : IS this Child Working Now**

No Observations

- **cb070_14_ : IS this Child Working Now**

	No	%
1 Yes	1	100.00
Total	1	100.00

- **cb070_17_ : IS this Child Working Now**

	No	%
1 Yes	1	100.00
Total	1	100.00

- **cb070_18_ : IS this Child Working Now**

	No	%

1 Yes	1	100.00
Total	1	100.00

- **cb070_20_ : IS this Child Working Now**

	No	%
2 No	1	100.00
Total	1	100.00

- **cb070_23_ : IS this Child Working Now**

	No	%
1 Yes	1	100.00
Total	1	100.00

- **cb070_25_ : IS this Child Working Now**

	No	%
1 Yes	1	100.00
Total	1	100.00

- **cb071_1_ : This Child's Main Occupation**

	No	%
1 Managers	622	8.05
2 Professionals and technicians	733	9.49
3 Clerks	352	4.56
4 Commercial and service workers	1,792	23.19
5 Agricultural, forestry, husbandry and fishery producers	1,922	24.88
6 Production and transportation workers	2,305	29.83
Total	7,726	100.00

- **cb071_2_ : This Child's Main Occupation**

	No	%
1 Managers	477	7.42
2 Professionals and technicians	506	7.87
3 Clerks	240	3.73
4 Commercial and service workers	1,548	24.09
5 Agricultural, forestry, husbandry and fishery producers	1,820	28.32
6 Production and transportation workers	1,835	28.56
Total	6,426	100.00

- **cb071_3_ : This Child's Main Occupation**

	No	%
1 Managers	238	6.08
2 Professionals and technicians	222	5.67
3 Clerks	115	2.94
4 Commercial and service workers	878	22.43
5 Agricultural, forestry, husbandry and fishery producers	1,432	36.59
6 Production and transportation workers	1,029	26.29
Total	3,914	100.00

- **cb071_4_ : This Child's Main Occupation**

	No	%
1 Managers	106	4.97
2 Professionals and technicians	97	4.55
3 Clerks	47	2.20
4 Commercial and service workers	437	20.48
5 Agricultural, forestry, husbandry and fishery producers	896	41.99
6 Production and transportation workers	551	25.82
Total	2,134	100.00

- **cb071_5_ : This Child's Main Occupation**

	No	%
1 Managers	50	4.77
2 Professionals and technicians	37	3.53
3 Clerks	21	2.00
4 Commercial and service workers	217	20.69
5 Agricultural, forestry, husbandry and fishery producers	495	47.19
6 Production and transportation workers	229	21.83
Total	1,049	100.00

- **cb071_6_ : This Child's Main Occupation**

	No	%
1 Managers	11	2.61
2 Professionals and technicians	10	2.38
3 Clerks	4	0.95
4 Commercial and service workers	75	17.81
5 Agricultural, forestry, husbandry and fishery producers	229	54.39
6 Production and transportation workers	92	21.85
Total	421	100.00

- **cb071_7_ : This Child's Main Occupation**

	No	%
1 Managers	6	3.77
2 Professionals and technicians	5	3.14
3 Clerks	3	1.89
4 Commercial and service workers	29	18.24
5 Agricultural, forestry, husbandry and fishery producers	78	49.06
6 Production and transportation workers	38	23.90
Total	159	100.00

- **cb071_8_ : This Child's Main Occupation**

	No	%
1 Managers	2	3.77
2 Professionals and technicians	2	3.77
4 Commercial and service workers	9	16.98
5 Agricultural, forestry, husbandry and fishery producers	29	54.72
6 Production and transportation workers	11	20.75
Total	53	100.00

- **cb071_9_ : This Child's Main Occupation**

	No	%
2 Professionals and technicians	1	5.00
4 Commercial and service workers	2	10.00
5 Agricultural, forestry, husbandry and fishery producers	14	70.00
6 Production and transportation workers	3	15.00
Total	20	100.00

- **cb071_10_ : This Child's Main Occupation**

	No	%
1 Managers	2	40.00
4 Commercial and service workers	1	20.00
5 Agricultural, forestry, husbandry and fishery producers	1	20.00
6 Production and transportation workers	1	20.00
Total	5	100.00

- **cb071_17_ : This Child's Main Occupation**

	No	%
6 Production and transportation workers	1	100.00
Total	1	100.00

- **cb071_23_ : This Child's Main Occupation**

	No	%
5 Agricultural, forestry, husbandry and fishery producers	1	100.00
Total	1	100.00

- **cb071_25_ : This Child's Main Occupation**

	No	%
5 Agricultural, forestry, husbandry and fishery producers	1	100.00
Total	1	100.00

- **cb072_1_ : This Child's Highest Administrative Level**

	No	%
1 Team Leader	95	1.23
2 Ke	75	0.97
3 Chu	34	0.44
4 Ju and above	18	0.23
5 Township Leader	42	0.54
6 None	7,469	96.59
Total	7,733	100.00

- **cb072_2_ : This Child's Highest Administrative Level**

	No	%
1 Team Leader	70	1.09
2 Ke	67	1.04
3 Chu	19	0.30
4 Ju and above	11	0.17
5 Township Leader	13	0.20
6 None	6,252	97.20
Total	6,432	100.00

- **cb072_3_ : This Child's Highest Administrative Level**

	No	%
1 Team Leader	31	0.79
2 Ke	34	0.87
3 Chu	6	0.15
4 Ju and above	3	0.08
5 Township Leader	7	0.18
6 None	3,830	97.93
Total	3,911	100.00

- **cb072_4_ : This Child's Highest Administrative Level**

	No	%
1 Team Leader	13	0.61
2 Ke	10	0.47
3 Chu	3	0.14
4 Ju and above	7	0.33
5 Township Leader	4	0.19
6 None	2,110	98.28
Total	2,147	100.00

- **cb072_5_ : This Child's Highest Administrative Level**

	No	%
1 Team Leader	2	0.19
2 Ke	2	0.19
4 Ju and above	2	0.19
5 Township Leader	3	0.29
6 None	1,037	99.14
Total	1,046	100.00

- **cb072_6_ : This Child's Highest Administrative Level**

	No	%
6 None	424	100.00
Total	424	100.00

- **cb072_7_ : This Child's Highest Administrative Level**

	No	%
1 Team Leader	1	0.63
5 Township Leader	1	0.63
6 None	157	98.74
Total	159	100.00

- **cb072_8_ : This Child's Highest Administrative Level**

	No	%
6 None	51	100.00
Total	51	100.00

- **cb072_9_ : This Child's Highest Administrative Level**

	No	%
6 None	20	100.00
Total	20	100.00

- **cb072_10_ : This Child's Highest Administrative Level**

	No	%
1 Team Leader	1	20.00
2 Ke	1	20.00
6 None	3	60.00
Total	5	100.00

- **cb072_17_ : This Child's Highest Administrative Level**

	No	%
6 None	1	100.00
Total	1	100.00

- **cb072_23_ : This Child's Highest Administrative Level**

	No	%
6 None	1	100.00
Total	1	100.00

- **cb072_25_ : This Child's Highest Administrative Level**

	No	%
6 None	1	100.00
Total	1	100.00

- **cb073_1_ : This Child's Highest Professional/Technical Level**

	No	%
1 Technician	200	2.61
2 Primary level	115	1.50
3 Intermediate level	164	2.14
4 Advanced level	83	1.08
5 None	7,095	92.66
Total	7,657	100.00

- **cb073_2_ : This Child's Highest Professional/Technical Level**

	No	%
1 Technician	118	1.86
2 Primary level	74	1.16
3 Intermediate level	96	1.51
4 Advanced level	66	1.04
5 None	6,001	94.43
Total	6,355	100.00

- **cb073_3_ : This Child's Highest Professional/Technical Level**

	No	%
1 Technician	52	1.34
2 Primary level	30	0.77
3 Intermediate level	49	1.27
4 Advanced level	30	0.77
5 None	3,712	95.84
Total	3,873	100.00

- **cb073_4_ : This Child's Highest Professional/Technical Level**

	No	%
1 Technician	19	0.89
2 Primary level	7	0.33
3 Intermediate level	14	0.66
4 Advanced level	13	0.61
5 None	2,075	97.51
Total	2,128	100.00

- **cb073_5_ : This Child's Highest Professional/Technical Level**

	No	%
1 Technician	8	0.76
2 Primary level	3	0.29
3 Intermediate level	4	0.38
4 Advanced level	6	0.57
5 None	1,025	97.99
Total	1,046	100.00

- **cb073_6_ : This Child's Highest Professional/Technical Level**

	No	%
1 Technician	2	0.47

	No	%
2 Primary level	1	0.24
4 Advanced level	1	0.24
5 None	418	99.05
Total	422	100.00

- **cb073_7_ : This Child's Highest Professional/Technical Level**

	No	%
2 Primary level	1	0.63
4 Advanced level	1	0.63
5 None	157	98.74
Total	159	100.00

- **cb073_8_ : This Child's Highest Professional/Technical Level**

	No	%
3 Intermediate level	1	1.96
4 Advanced level	1	1.96
5 None	49	96.08
Total	51	100.00

- **cb073_9_ : This Child's Highest Professional/Technical Level**

	No	%
3 Intermediate level	1	5.00
5 None	19	95.00
Total	20	100.00

- **cb073_10_ : This Child's Highest Professional/Technical Level**

	No	%
4 Advanced level	1	25.00
5 None	3	75.00
Total	4	100.00

- **cb073_17_ : This Child's Highest Professional/Technical Level**

	No	%
5 None	1	100.00
Total	1	100.00

- **cb073_23_ : This Child's Highest Professional/Technical Level**

	No	%
5 None	1	100.00
Total	1	100.00

- **cb073_25_ : This Child's Highest Professional/Technical Level**

	No	%
5 None	1	100.00
Total	1	100.00

- **cb074_1_ : Has this Child Ever Worked before**

	No	%
1 Yes	723	40.76
2 No	1,051	59.24
Total	1,774	100.00

- **cb074_2_ : Has this Child Ever Worked before**

	No	%
1 Yes	662	46.55
2 No	760	53.45
Total	1,422	100.00

- **cb074_3_ : Has this Child Ever Worked before**

	No	%
1 Yes	408	54.40
2 No	342	45.60
Total	750	100.00

- **cb074_4_ : Has this Child Ever Worked before**

	No	%
1 Yes	201	57.76
2 No	147	42.24
Total	348	100.00

- **cb074_5_ : Has this Child Ever Worked before**

	No	%
1 Yes	103	62.42
2 No	62	37.58
Total	165	100.00

- **cb074_6_ : Has this Child Ever Worked before**

	No	%
1 Yes	42	62.69
2 No	25	37.31
Total	67	100.00

- **cb074_7_ : Has this Child Ever Worked before**

	No	%
1 Yes	19	73.08
2 No	7	26.92
Total	26	100.00

- **cb074_8_ : Has this Child Ever Worked before**

	No	%
1 Yes	6	75.00
2 No	2	25.00
Total	8	100.00

- **cb074_9_ : Has this Child Ever Worked before**

	No	%
1 Yes	2	100.00
Total	2	100.00

- **cb074_10_ : Has this Child Ever Worked before**

No Observations

- **cb074_11_ : Has this Child Ever Worked before**

No Observations

- **cb074_12_ : Has this Child Ever Worked before**

	No	%
2 No	1	100.00
Total	1	100.00

- **cb074_13_ : Has this Child Ever Worked before**

	No	%
1 Yes	1	100.00
Total	1	100.00

- **cb074_14_ : Has this Child Ever Worked before**

	No	%
1 Yes	1	33.33
2 No	2	66.67
Total	3	100.00

- **cb074_15_ : Has this Child Ever Worked before**

	No	%
1 Yes	1	100.00
Total	1	100.00

- **cb074_17_ : Has this Child Ever Worked before**

	No	%
1 Yes	1	100.00
Total	1	100.00

- **cb074_20_ : Has this Child Ever Worked before**

	No	%
1 Yes	1	100.00
Total	1	100.00

- **cb075_1_ : This Child's Sort of Work**

	No	%
1 Managers	55	7.72
2 Professionals and technicians	61	8.57
3 Clerks	28	3.93
4 Commercial and service workers	173	24.30
5 Agricultural, forestry, husbandry and fishery producers	181	25.42
6 Production and transportation workers	214	30.06
Total	712	100.00

- **cb075_2_ : This Child's Sort of Work**

	No	%
1 Managers	44	6.85
2 Professionals and technicians	40	6.23
3 Clerks	32	4.98
4 Commercial and service workers	179	27.88
5 Agricultural, forestry, husbandry and fishery producers	172	26.79
6 Production and transportation workers	175	27.26
Total	642	100.00

- **cb075_3_ : This Child's Sort of Work**

	No	%
1 Managers	36	9.00
2 Professionals and technicians	14	3.50
3 Clerks	17	4.25
4 Commercial and service workers	94	23.50
5 Agricultural, forestry, husbandry and fishery producers	119	29.75
6 Production and transportation workers	120	30.00
Total	400	100.00

- **cb075_4_ : This Child's Sort of Work**

	No	%
1 Managers	16	8.08
2 Professionals and technicians	9	4.55
3 Clerks	13	6.57
4 Commercial and service workers	42	21.21
5 Agricultural, forestry, husbandry and fishery producers	60	30.30
6 Production and transportation workers	58	29.29
Total	198	100.00

- **cb075_5_ : This Child's Sort of Work**

	No	%
1 Managers	8	8.33
2 Professionals and technicians	3	3.13
3 Clerks	2	2.08
4 Commercial and service workers	19	19.79
5 Agricultural, forestry, husbandry and fishery producers	35	36.46
6 Production and transportation workers	29	30.21
Total	96	100.00

- **cb075_6_ : This Child's Sort of Work**

	No	%
1 Managers	5	12.50
2 Professionals and technicians	1	2.50
3 Clerks	1	2.50
4 Commercial and service workers	6	15.00
5 Agricultural, forestry, husbandry and fishery producers	17	42.50
6 Production and transportation workers	10	25.00
Total	40	100.00

- **cb075_7_ : This Child's Sort of Work**

	No	%
1 Managers	1	5.56
3 Clerks	1	5.56
4 Commercial and service workers	1	5.56
5 Agricultural, forestry, husbandry and fishery producers	10	55.56
6 Production and transportation workers	5	27.78
Total	18	100.00

- **cb075_8_ : This Child's Sort of Work**

	No	%
1 Managers	1	16.67
4 Commercial and service workers	1	16.67
5 Agricultural, forestry, husbandry and fishery producers	4	66.67
Total	6	100.00

- **cb075_9_ : This Child's Sort of Work**

	No	%
5 Agricultural, forestry, husbandry and fishery producers	1	100.00
Total	1	100.00

- **cb076_1_ : Dis this Child Live with Others, Away from You, before Age 16 for more than 6 Mo**

	No	%
1 Yes	303	3.18
2 No	9,234	96.82
Total	9,537	100.00

- **cb076_2_ : Dis this Child Live with Others, Away from You, before Age 16 for more than 6 Mo**

	No	%
1 Yes	177	2.25
2 No	7,697	97.75
Total	7,874	100.00

- **cb076_3_ : Dis this Child Live with Others, Away from You, before Age 16 for more than 6 Mo**

	No	%
1 Yes	96	2.05
2 No	4,581	97.95
Total	4,677	100.00

- **cb076_4_ : Dis this Child Live with Others, Away from You, before Age 16 for more than 6 Mo**

	No	%
1 Yes	41	1.64
2 No	2,462	98.36
Total	2,503	100.00

- **cb076_5_ : Dis this Child Live with Others, Away from You, before Age 16 for more than 6 Mo**

	No	%
1 Yes	20	1.66
2 No	1,187	98.34
Total	1,207	100.00

- **cb076_6_ : Dis this Child Live with Others, Away from You, before Age 16 for more than 6 Mo**

	No	%
1 Yes	10	2.02
2 No	485	97.98
Total	495	100.00

- **cb076_7_ : Dis this Child Live with Others, Away from You, before Age 16 for more than 6 Mo**

	No	%
1 Yes	3	1.62
2 No	182	98.38
Total	185	100.00

- **cb076_8_ : Dis this Child Live with Others, Away from You, before Age 16 for more than 6 Mo**

	No	%
1 Yes	2	3.33
2 No	58	96.67
Total	60	100.00

- **cb076_9_ : Dis this Child Live with Others, Away from You, before Age 16 for more than 6 Mo**

	No	%
1 Yes	1	4.55
2 No	21	95.45
Total	22	100.00

- **cb076_10_ : Dis this Child Live with Others, Away from You, before Age 16 for more than 6 Mo**

	No	%
1 Yes	1	16.67
2 No	5	83.33
Total	6	100.00

- **cb076_11_ : Dis this Child Live with Others, Away from You, before Age 16 for more than 6 Mo**

No Observations

- **cb076_12_ : Dis this Child Live with Others, Away from You, before Age 16 for more than 6 Mo**

	No	%
2 No	1	100.00
Total	1	100.00

- **cb076_13_ : Dis this Child Live with Others, Away from You, before Age 16 for more than 6 Mo**

No Observations

- **cb076_14_ : Dis this Child Live with Others, Away from You, before Age 16 for more than 6 Mo**

No Observations

- **cb076_17_ : Dis this Child Live with Others, Away from You, before Age 16 for more than 6 Mo**

	No	%
2 No	2	100.00
Total	2	100.00

- **cb076_18_ : Dis this Child Live with Others, Away from You, before Age 16 for more than 6 Mo**

	No	%
1 Yes	2	100.00
Total	2	100.00

- **cb076_19_ : Dis this Child Live with Others, Away from You, before Age 16 for more than 6 Mo**

	No	%

2 No	1	100.00
Total	1	100.00

- **cb076_20_ : Dis this Child Live with Others, Away from You, before Age 16 for more than 6 Mo**

	No	%
2 No	1	100.00
Total	1	100.00

- **cb076_23_ : Dis this Child Live with Others, Away from You, before Age 16 for more than 6 Mo**

	No	%
2 No	1	100.00
Total	1	100.00

- **cb076_25_ : Dis this Child Live with Others, Away from You, before Age 16 for more than 6 Mo**

	No	%
2 No	1	100.00
Total	1	100.00

- **cb077_1_ : Earliest Age this Child Lived Separated from You**

	No	%
1	29	9.60
2	10	3.31
3	7	2.32
4	8	2.65
5	3	0.99
6	6	1.99
7	9	2.98
8	11	3.64
9	7	2.32
10	8	2.65
11	5	1.66
12	21	6.95
13	35	11.59
14	38	12.58
15	72	23.84
16	33	10.93

Total	302	100.00

- **cb077_2_ : Earliest Age this Child Lived Separated from You**

	No	%
1	24	13.71
2	5	2.86
3	6	3.43
4	3	1.71
5	3	1.71
6	4	2.29
7	7	4.00
8	6	3.43
9	5	2.86
11	4	2.29
12	9	5.14
13	22	12.57
14	27	15.43
15	31	17.71
16	19	10.86
Total	175	100.00

- **cb077_3_ : Earliest Age this Child Lived Separated from You**

	No	%
1	36	34.95
2	5	4.85
3	6	5.83
4	1	0.97
5	1	0.97
6	2	1.94
7	1	0.97
8	3	2.91
9	1	0.97
10	3	2.91
11	1	0.97
12	6	5.83
13	7	6.80
14	8	7.77
15	17	16.50
16	5	4.85
Total	103	100.00

- **cb077_4_ : Earliest Age this Child Lived Separated from You**

	No	%
1	14	34.15

3	3	7.32
4	1	2.44
5	1	2.44
6	1	2.44
9	1	2.44
10	1	2.44
11	1	2.44
12	3	7.32
13	1	2.44
14	5	12.20
15	8	19.51
16	1	2.44
Total	41	100.00

- **cb077_5_ : Earliest Age this Child Lived Separated from You**

	No	%
1	7	36.84
2	1	5.26
5	1	5.26
6	1	5.26
9	3	15.79
11	2	10.53
14	2	10.53
16	2	10.53
Total	19	100.00

- **cb077_6_ : Earliest Age this Child Lived Separated from You**

	No	%
1	5	55.56
4	1	11.11
6	1	11.11
7	1	11.11
14	1	11.11
Total	9	100.00

- **cb077_7_ : Earliest Age this Child Lived Separated from You**

	No	%
1	2	66.67
6	1	33.33
Total	3	100.00

- **cb077_8_ : Earliest Age this Child Lived Separated from You**

	No	%
1	1	50.00
13	1	50.00
Total	2	100.00

- **cb077_9_ : Earliest Age this Child Lived Separated from You**

	No	%
1	1	100.00
Total	1	100.00

- **cb077_10_ : Earliest Age this Child Lived Separated from You**

	No	%
1	1	100.00
Total	1	100.00

- **cb078_1_ : The Person Live with for the Longest Time When not Living With You**

	No	%
1 Your parents	28	8.92
2 Your spouses parents	37	11.78
3 Your brothers or sisters family	15	4.78
4 Other family	23	7.32
5 Dormitory	99	31.53
6 Other	112	35.67
Total	314	100.00

- **cb078_2_ : The Person Live with for the Longest Time When not Living With You**

	No	%
1 Your parents	16	9.14
2 Your spouses parents	19	10.86
3 Your brothers or sisters family	11	6.29
4 Other family	16	9.14
5 Dormitory	54	30.86
6 Other	59	33.71
Total	175	100.00

- **cb078_3_ : The Person Live with for the Longest Time When not Living**

With You

	No	%
1 Your parents	9	8.91
2 Your spouses parents	13	12.87
3 Your brothers or sisters family	6	5.94
4 Other family	25	24.75
5 Dormitory	17	16.83
6 Other	31	30.69
Total	101	100.00

- **cb078_4_ : The Person Live with for the Longest Time When not Living With You**

	No	%
1 Your parents	1	2.44
2 Your spouses parents	2	4.88
3 Your brothers or sisters family	3	7.32
4 Other family	15	36.59
5 Dormitory	4	9.76
6 Other	16	39.02
Total	41	100.00

- **cb078_5_ : The Person Live with for the Longest Time When not Living With You**

	No	%
1 Your parents	2	10.00
2 Your spouses parents	1	5.00
3 Your brothers or sisters family	1	5.00
4 Other family	7	35.00
5 Dormitory	1	5.00
6 Other	8	40.00
Total	20	100.00

- **cb078_6_ : The Person Live with for the Longest Time When not Living With You**

	No	%
1 Your parents	1	10.00
4 Other family	3	30.00
6 Other	6	60.00
Total	10	100.00

- **cb078_7_ : The Person Live with for the Longest Time When not Living With You**

	No	%
4 Other family	2	66.67
6 Other	1	33.33
Total	3	100.00

- **cb078_8_ : The Person Live with for the Longest Time When not Living With You**

	No	%
4 Other family	1	50.00
6 Other	1	50.00
Total	2	100.00

- **cb078_9_ : The Person Live with for the Longest Time When not Living With You**

	No	%
4 Other family	1	100.00
Total	1	100.00

- **cb078_10_ : The Person Live with for the Longest Time When not Living With You**

	No	%
4 Other family	1	100.00
Total	1	100.00

- **cb079_1_ : Num. of Years Living with Others**

Mean	Min	Max	OBS
4.6	0.0	16.0	280

- **cb079_1_1_ : Num. of Months Living with Others**

	No	%
0	123	77.85
1	3	1.90
2	5	3.16

3	4	2.53
4	2	1.27
5	4	2.53
6	12	7.59
7	2	1.27
8	1	0.63
10	2	1.27
Total	158	100.00

- **cb079_1_2_ : Num. of Months Living with Others**

	No	%
0	73	81.11
1	2	2.22
2	4	4.44
3	1	1.11
5	1	1.11
6	6	6.67
7	3	3.33
Total	90	100.00

- **cb079_1_3_ : Num. of Months Living with Others**

	No	%
0	38	77.55
1	2	4.08
2	1	2.04
6	4	8.16
9	1	2.04
10	3	6.12
Total	49	100.00

- **cb079_1_4_ : Num. of Months Living with Others**

	No	%
0	18	90.00
6	2	10.00
Total	20	100.00

- **cb079_1_5_ : Num. of Months Living with Others**

	No	%
0	11	100.00
Total	11	100.00

- **cb079_1_6_ : Num. of Months Living with Others**

	No	%
0	5	100.00
Total	5	100.00

- **cb079_1_7_ : Num. of Months Living with Others**

	No	%
0	3	100.00
Total	3	100.00

- **cb079_1_8_ : Num. of Months Living with Others**

	No	%
0	1	100.00
Total	1	100.00

- **cb079_1_9_ : Num. of Months Living with Others**

	No	%
0	1	100.00
Total	1	100.00

- **cb079_1_10_ : Num. of Months Living with Others**

	No	%
0	1	100.00
Total	1	100.00

- **cb079_2_ : Num. of Years Living with Others**

Mean	Min	Max	OBS
5.2	0.0	16.0	164

- **cb079_3_ : Num. of Years Living with Others**

	No	%
0	3	3.19
1	13	13.83

2	11	11.70
3	9	9.57
4	6	6.38
5	2	2.13
6	1	1.06
8	2	2.13
9	1	1.06
10	5	5.32
11	1	1.06
12	2	2.13
13	8	8.51
14	3	3.19
15	8	8.51
16	19	20.21
Total	94	100.00

- **cb079_4_ : Num. of Years Living with Others**

	No	%
0	1	2.78
1	6	16.67
2	4	11.11
3	4	11.11
4	1	2.78
5	4	11.11
6	1	2.78
11	1	2.78
12	1	2.78
13	2	5.56
15	3	8.33
16	8	22.22
Total	36	100.00

- **cb079_5_ : Num. of Years Living with Others**

	No	%
0	1	5.26
1	1	5.26
2	1	5.26
3	1	5.26
5	3	15.79
7	2	10.53
8	1	5.26
9	1	5.26
10	2	10.53
14	2	10.53
16	4	21.05
Total	19	100.00

- **cb079_6_ : Num. of Years Living with Others**

Mean	Min	Max	OBS
12.1	0.5	16.0	9

- **cb079_7_ : Num. of Years Living with Others**

	No	%
10	1	33.33
15	2	66.67
Total	3	100.00

- **cb079_8_ : Num. of Years Living with Others**

	No	%
2	1	50.00
15	1	50.00
Total	2	100.00

- **cb079_9_ : Num. of Years Living with Others**

	No	%
15	1	100.00
Total	1	100.00

- **cb079_10_ : Num. of Years Living with Others**

	No	%
16	1	100.00
Total	1	100.00

- **cb080_1_s1 : Parents of this Grandchild**

	No	%
1 BC002[1]	2,869	100.00
Total	2,869	100.00

- **cb080_1_s2 : Parents of this Grandchild**

	No	%

	No	%
2 BC002[2]	382	100.00
Total	382	100.00

- **cb080_1_s3 : Parents of this Grandchild**

	No	%
3 BC002[3]	120	100.00
Total	120	100.00

- **cb080_1_s4 : Parents of this Grandchild**

	No	%
4 BC002[4]	52	100.00
Total	52	100.00

- **cb080_1_s5 : Parents of this Grandchild**

	No	%
5 BC002[5]	20	100.00
Total	20	100.00

- **cb080_1_s6 : Parents of this Grandchild**

	No	%
6 BC002[6]	5	100.00
Total	5	100.00

- **cb080_1_s7 : Parents of this Grandchild**

No Observations

- **cb080_1_s8 : Parents of this Grandchild**

	No	%
8 BC002[8]	1	100.00
Total	1	100.00

- **cb080_1_s9 : Parents of this Grandchild**

No Observations

- **cb080_1_s10 : Parents of this Grandchild**

No Observations

- **cb080_1_s11 : Parents of this Grandchild**

No Observations

- **cb080_1_s12 : Parents of this Grandchild**

No Observations

- **cb080_1_s13 : Parents of this Grandchild**

No Observations

- **cb080_1_s14 : Parents of this Grandchild**

No Observations

- **cb080_1_s15 : Parents of this Grandchild**

No Observations

- **cb080_1_s16 : Parents of this Grandchild**

No Observations

- **cb080_1_s17 : Parents of this Grandchild**

No Observations

- **cb080_1_s18 : Parents of this Grandchild**

No Observations

- **cb080_1_s19 : Parents of this Grandchild**

No Observations

- **cb080_1_s20 : Parents of this Grandchild**

No Observations

- **cb080_1_s21 : Parents of this Grandchild**

No Observations

- **cb080_1_s22 : Parents of this Grandchild**

No Observations

- **cb080_1_s23 : Parents of this Grandchild**

No Observations

- **cb080_1_s24 : Parents of this Grandchild**

No Observations

- **cb080_1_s25 : Parents of this Grandchild**

No Observations

- **cb080_1_s26 : Parents of this Grandchild**

	No	%
26 Other:	349	100.00
Total	349	100.00

- **cb080_2_s1 : Parents of this Grandchild**

	No	%
1 BC002[1]	1,080	100.00
Total	1,080	100.00

- **cb080_2_s2 : Parents of this Grandchild**

	No	%
2 BC002[2]	216	100.00
Total	216	100.00

- **cb080_2_s3 : Parents of this Grandchild**

	No	%
3 BC002[3]	43	100.00
Total	43	100.00

- **cb080_2_s4 : Parents of this Grandchild**

	No	%
4 BC002[4]	29	100.00
Total	29	100.00

- **cb080_2_s5 : Parents of this Grandchild**

	No	%
5 BC002[5]	7	100.00
Total	7	100.00

- **cb080_2_s6 : Parents of this Grandchild**

	No	%
6 BC002[6]	3	100.00
Total	3	100.00

- **cb080_2_s7 : Parents of this Grandchild**

	No	%
7 BC002[7]	2	100.00
Total	2	100.00

- **cb080_2_s8 : Parents of this Grandchild**

No Observations

- **cb080_2_s9 : Parents of this Grandchild**

No Observations

- **cb080_2_s10 : Parents of this Grandchild**

No Observations

- **cb080_2_s11 : Parents of this Grandchild**

No Observations

- **cb080_2_s12 : Parents of this Grandchild**

No Observations

- **cb080_2_s13 : Parents of this Grandchild**

No Observations

- **cb080_2_s14 : Parents of this Grandchild**

No Observations

- **cb080_2_s15 : Parents of this Grandchild**

No Observations

- **cb080_2_s16 : Parents of this Grandchild**

No Observations

- **cb080_2_s17 : Parents of this Grandchild**

No Observations

- **cb080_2_s18 : Parents of this Grandchild**

No Observations

- **cb080_2_s19 : Parents of this Grandchild**

No Observations

- **cb080_2_s20 : Parents of this Grandchild**

No Observations

- **cb080_2_s21 : Parents of this Grandchild**

No Observations

- **cb080_2_s22 : Parents of this Grandchild**

No Observations

- **cb080_2_s23 : Parents of this Grandchild**

No Observations

- **cb080_2_s24 : Parents of this Grandchild**

No Observations

- **cb080_2_s25 : Parents of this Grandchild**

No Observations

- **cb080_2_s26 : Parents of this Grandchild**

	No	%
26 Other:	142	100.00
Total	142	100.00

- **cb080_3_s1 : Parents of this Grandchild**

	No	%

1 BC002[1]	149	100.00
Total	149	100.00

- **cb080_3_s2 : Parents of this Grandchild**

	No	%
2 BC002[2]	54	100.00
Total	54	100.00

- **cb080_3_s3 : Parents of this Grandchild**

	No	%
3 BC002[3]	26	100.00
Total	26	100.00

- **cb080_3_s4 : Parents of this Grandchild**

	No	%
4 BC002[4]	5	100.00
Total	5	100.00

- **cb080_3_s5 : Parents of this Grandchild**

	No	%
5 BC002[5]	2	100.00
Total	2	100.00

- **cb080_3_s6 : Parents of this Grandchild**

	No	%
6 BC002[6]	2	100.00
Total	2	100.00

- **cb080_3_s7 : Parents of this Grandchild**

	No	%
7 BC002[7]	1	100.00
Total	1	100.00

- **cb080_3_s8 : Parents of this Grandchild**

No Observations

- **cb080_3_s9 : Parents of this Grandchild**

No Observations

- **cb080_3_s10 : Parents of this Grandchild**

No Observations

- **cb080_3_s11 : Parents of this Grandchild**

No Observations

- **cb080_3_s12 : Parents of this Grandchild**

No Observations

- **cb080_3_s13 : Parents of this Grandchild**

No Observations

- **cb080_3_s14 : Parents of this Grandchild**

No Observations

- **cb080_3_s15 : Parents of this Grandchild**

No Observations

- **cb080_3_s16 : Parents of this Grandchild**

No Observations

- **cb080_3_s17 : Parents of this Grandchild**

No Observations

- **cb080_3_s18 : Parents of this Grandchild**

No Observations

- **cb080_3_s19 : Parents of this Grandchild**

No Observations

- **cb080_3_s20 : Parents of this Grandchild**

No Observations

- **cb080_3_s21 : Parents of this Grandchild**

No Observations

- **cb080_3_s22 : Parents of this Grandchild**

No Observations

- **cb080_3_s23 : Parents of this Grandchild**

No Observations

- **cb080_3_s24 : Parents of this Grandchild**

No Observations

- **cb080_3_s25 : Parents of this Grandchild**

No Observations

- **cb080_3_s26 : Parents of this Grandchild**

	No	%
26 Other:	21	100.00
Total	21	100.00

- **cb080_4_s1 : Parents of this Grandchild**

	No	%
1 BC002[1]	21	100.00
Total	21	100.00

- **cb080_4_s2 : Parents of this Grandchild**

	No	%
2 BC002[2]	18	100.00
Total	18	100.00

- **cb080_4_s3 : Parents of this Grandchild**

	No	%
3 BC002[3]	8	100.00
Total	8	100.00

- **cb080_4_s4 : Parents of this Grandchild**

	No	%
4 BC002[4]	1	100.00
Total	1	100.00

- **cb080_4_s5 : Parents of this Grandchild**

	No	%
5 BC002[5]	1	100.00
Total	1	100.00

- **cb080_4_s6 : Parents of this Grandchild**

No Observations

- **cb080_4_s7 : Parents of this Grandchild**

No Observations

- **cb080_4_s8 : Parents of this Grandchild**

No Observations

- **cb080_4_s9 : Parents of this Grandchild**

No Observations

- **cb080_4_s10 : Parents of this Grandchild**

No Observations

- **cb080_4_s11 : Parents of this Grandchild**

No Observations

- **cb080_4_s12 : Parents of this Grandchild**

No Observations

- **cb080_4_s13 : Parents of this Grandchild**

No Observations

- **cb080_4_s14 : Parents of this Grandchild**

No Observations

- **cb080_4_s15 : Parents of this Grandchild**

No Observations

- **cb080_4_s16 : Parents of this Grandchild**

No Observations

- **cb080_4_s17 : Parents of this Grandchild**

No Observations

- **cb080_4_s18 : Parents of this Grandchild**

No Observations

- **cb080_4_s19 : Parents of this Grandchild**

No Observations

- **cb080_4_s20 : Parents of this Grandchild**

No Observations

- **cb080_4_s21 : Parents of this Grandchild**

No Observations

- **cb080_4_s22 : Parents of this Grandchild**

No Observations

- **cb080_4_s23 : Parents of this Grandchild**

No Observations

- **cb080_4_s24 : Parents of this Grandchild**

No Observations

- **cb080_4_s25 : Parents of this Grandchild**

No Observations

- **cb080_4_s26 : Parents of this Grandchild**

	No	%

26 Other:	5	100.00
Total	5	100.00

- **cb080_5_s1 : Parents of this Grandchild**

	No	%
1 BC002[1]	2	100.00
Total	2	100.00

- **cb080_5_s2 : Parents of this Grandchild**

	No	%
2 BC002[2]	3	100.00
Total	3	100.00

- **cb080_5_s3 : Parents of this Grandchild**

No Observations

- **cb080_5_s4 : Parents of this Grandchild**

No Observations

- **cb080_5_s5 : Parents of this Grandchild**

	No	%
5 BC002[5]	1	100.00
Total	1	100.00

- **cb080_5_s6 : Parents of this Grandchild**

No Observations

- **cb080_5_s7 : Parents of this Grandchild**

No Observations

- **cb080_5_s8 : Parents of this Grandchild**

No Observations

- **cb080_5_s9 : Parents of this Grandchild**

No Observations

- **cb080_5_s10 : Parents of this Grandchild**

No Observations

- **cb080_5_s11 : Parents of this Grandchild**

No Observations

- **cb080_5_s12 : Parents of this Grandchild**

No Observations

- **cb080_5_s13 : Parents of this Grandchild**

No Observations

- **cb080_5_s14 : Parents of this Grandchild**

No Observations

- **cb080_5_s15 : Parents of this Grandchild**

No Observations

- **cb080_5_s16 : Parents of this Grandchild**

No Observations

- **cb080_5_s17 : Parents of this Grandchild**

No Observations

- **cb080_5_s18 : Parents of this Grandchild**

No Observations

- **cb080_5_s19 : Parents of this Grandchild**

No Observations

- **cb080_5_s20 : Parents of this Grandchild**

No Observations

- **cb080_5_s21 : Parents of this Grandchild**

No Observations

- **cb080_5_s22 : Parents of this Grandchild**

No Observations

- **cb080_5_s23 : Parents of this Grandchild**

No Observations

- **cb080_5_s24 : Parents of this Grandchild**

No Observations

- **cb080_5_s25 : Parents of this Grandchild**

No Observations

- **cb080_5_s26 : Parents of this Grandchild**

	No	%
26 Other:	2	100.00
Total	2	100.00

- **cb080_6_s1 : Parents of this Grandchild**

	No	%
1 BC002[1]	1	100.00
Total	1	100.00

- **cb080_6_s2 : Parents of this Grandchild**

No Observations

- **cb080_6_s3 : Parents of this Grandchild**

	No	%
3 BC002[3]	1	100.00
Total	1	100.00

- **cb080_6_s4 : Parents of this Grandchild**

No Observations

- **cb080_6_s5 : Parents of this Grandchild**

No Observations

- **cb080_6_s6 : Parents of this Grandchild**

No Observations

- **cb080_6_s7 : Parents of this Grandchild**

No Observations

- **cb080_6_s8 : Parents of this Grandchild**

No Observations

- **cb080_6_s9 : Parents of this Grandchild**

No Observations

- **cb080_6_s10 : Parents of this Grandchild**

No Observations

- **cb080_6_s11 : Parents of this Grandchild**

No Observations

- **cb080_6_s12 : Parents of this Grandchild**

No Observations

- **cb080_6_s13 : Parents of this Grandchild**

No Observations

- **cb080_6_s14 : Parents of this Grandchild**

No Observations

- **cb080_6_s15 : Parents of this Grandchild**

No Observations

- **cb080_6_s16 : Parents of this Grandchild**

No Observations

- **cb080_6_s17 : Parents of this Grandchild**

No Observations

- **cb080_6_s18 : Parents of this Grandchild**

No Observations

- **cb080_6_s19 : Parents of this Grandchild**

No Observations

- **cb080_6_s20 : Parents of this Grandchild**

No Observations

- **cb080_6_s21 : Parents of this Grandchild**

No Observations

- **cb080_6_s22 : Parents of this Grandchild**

No Observations

- **cb080_6_s23 : Parents of this Grandchild**

No Observations

- **cb080_6_s24 : Parents of this Grandchild**

No Observations

- **cb080_6_s25 : Parents of this Grandchild**

No Observations

- **cb080_6_s26 : Parents of this Grandchild**

No Observations

- **cb081_1_ : Birth Place of this Child**

	No	%
1 This Village/Neighborhood	3,400	82.24
2 Another Village/Neighborhood in this County/City	521	12.60
3 Another County/City in this Province	104	2.52
4 Another Province	107	2.59
5 Abroad	2	0.05
Total	4,134	100.00

- **cb081_1_1_ : City**

	No	%
01	6	5.77
02	1	0.96
04	6	5.77
05	1	0.96
11	10	9.62
16	2	1.92
17	3	2.88
24	8	7.69
27	3	2.88
35	1	0.96
40	16	15.38
46	9	8.65
49	6	5.77
51	1	0.96
53	4	3.85
55	7	6.73
60	5	4.81
63	1	0.96
74	5	4.81
82	6	5.77
96	3	2.88
Total	104	100.00

- **cb081_1_2_ : City**

	No	%
01	8	5.56
02	1	0.69
04	7	4.86
07	1	0.69
11	14	9.72
16	2	1.39

17	1	0.69
24	7	4.86
27	4	2.78
28	2	1.39
35	2	1.39
40	17	11.81
46	20	13.89
49	3	2.08
50	1	0.69
51	2	1.39
53	4	2.78
55	8	5.56
60	5	3.47
63	6	4.17
74	5	3.47
76	1	0.69
77	1	0.69
82	15	10.42
86	3	2.08
96	2	1.39
99	2	1.39
Total	144	100.00

- **cb081_1_3_ : City**

	No	%
01	8	9.41
04	2	2.35
05	1	1.18
07	1	1.18
11	6	7.06
16	2	2.35
17	2	2.35
24	5	5.88
28	2	2.35
40	11	12.94
46	9	10.59
49	2	2.35
50	1	1.18
51	1	1.18
53	2	2.35
55	4	4.71
60	6	7.06
63	3	3.53
74	4	4.71
82	10	11.76
86	1	1.18
96	1	1.18
99	1	1.18
Total	85	100.00

- **cb081_1_4_ : City**

	No	%
01	2	4.26
04	1	2.13
07	1	2.13
11	5	10.64
16	1	2.13
17	1	2.13
24	2	4.26
28	1	2.13
40	6	12.77
46	7	14.89
49	2	4.26
50	1	2.13
55	3	6.38
60	2	4.26
63	1	2.13
74	1	2.13
82	8	17.02
86	1	2.13
96	1	2.13
Total	47	100.00

- **cb081_1_5_ : City**

	No	%
01	1	5.88
11	1	5.88
24	1	5.88
27	1	5.88
40	4	23.53
46	3	17.65
53	2	11.76
63	1	5.88
82	2	11.76
86	1	5.88
Total	17	100.00

- **cb081_1_6_ : City**

	No	%
40	1	50.00
46	1	50.00
Total	2	100.00

- **cb081_1_7_ : City**

	No	%
40	1	100.00
Total	1	100.00

- **cb081_2_ : Birth Place of this Child**

	No	%
1 This Village/Neighborhood	5,380	85.19
2 Another Village/Neighborhood in this County/City	654	10.36
3 Another County/City in this Province	143	2.26
4 Another Province	138	2.19
Total	6,315	100.00

- **cb081_2_1_ : County**

	No	%
02	5	5.26
03	1	1.05
04	2	2.11
06	7	7.37
28	4	4.21
31	9	9.47
33	4	4.21
38	2	2.11
39	2	2.11
43	4	4.21
44	2	2.11
46	3	3.16
51	3	3.16
54	4	4.21
56	1	1.05
57	1	1.05
59	8	8.42
63	16	16.84
76	6	6.32
78	3	3.16
81	5	5.26
91	1	1.05
92	1	1.05
99	1	1.05
Total	95	100.00

- **cb081_2_2_ : County**

	No	%

02	9	6.67
03	2	1.48
04	2	1.48
06	9	6.67
08	1	0.74
13	1	0.74
28	6	4.44
31	15	11.11
33	3	2.22
37	1	0.74
38	1	0.74
39	2	1.48
43	8	5.93
44	2	1.48
46	4	2.96
51	3	2.22
54	1	0.74
56	1	0.74
59	18	13.33
63	26	19.26
76	11	8.15
78	1	0.74
81	5	3.70
90	1	0.74
91	1	0.74
92	1	0.74
Total	135	100.00

- **cb081_2_3_ : County**

	No	%
02	5	6.41
03	2	2.56
04	2	2.56
06	4	5.13
28	5	6.41
31	9	11.54
33	1	1.28
43	5	6.41
44	3	3.85
46	5	6.41
51	2	2.56
54	2	2.56
56	1	1.28
59	6	7.69
63	19	24.36
76	3	3.85
81	4	5.13
Total	78	100.00

- **cb081_2_4_ : County**

	No	%
02	3	7.14
03	1	2.38
04	2	4.76
06	2	4.76
28	2	4.76
31	2	4.76
33	1	2.38
43	3	7.14
44	3	7.14
45	1	2.38
46	3	7.14
51	2	4.76
54	1	2.38
59	3	7.14
63	7	16.67
76	3	7.14
81	3	7.14
Total	42	100.00

- **cb081_2_5_ : County**

	No	%
02	1	6.25
04	1	6.25
28	2	12.50
43	1	6.25
44	1	6.25
51	1	6.25
54	2	12.50
59	2	12.50
63	3	18.75
76	1	6.25
81	1	6.25
Total	16	100.00

- **cb081_2_6_ : County**

	No	%
28	1	50.00
81	1	50.00
Total	2	100.00

- **cb081_2_7_ : County**

	No	%
81	1	100.00
Total	1	100.00

- **cb081_3_ : Birth Place of this Child**

	No	%
1 This Village/Neighborhood	3,782	86.68
2 Another Village/Neighborhood in this County/City	420	9.63
3 Another County/City in this Province	86	1.97
4 Another Province	75	1.72
Total	4,363	100.00

- **cb081_3_1_ : Province**

	No	%
01	2	1.89
02	2	1.89
03	2	1.89
04	1	0.94
05	6	5.66
06	8	7.55
07	2	1.89
08	1	0.94
09	3	2.83
10	4	3.77
11	5	4.72
12	3	2.83
13	2	1.89
14	10	9.43
16	5	4.72
17	4	3.77
18	3	2.83
19	3	2.83
20	2	1.89
21	9	8.49
25	2	1.89
26	1	0.94
28	2	1.89
29	10	9.43
32	3	2.83
33	7	6.60
34	4	3.77
Total	106	100.00

- **cb081_3_2_ : Province**

	No	%
01	1	0.73
02	1	0.73
03	3	2.19
05	13	9.49
06	8	5.84
07	4	2.92
08	1	0.73
09	3	2.19
10	8	5.84
11	2	1.46
12	4	2.92
13	3	2.19
14	15	10.95
15	2	1.46
16	7	5.11
17	4	2.92
18	1	0.73
19	1	0.73
20	4	2.92
21	14	10.22
24	1	0.73
25	3	2.19
26	2	1.46
27	4	2.92
28	2	1.46
29	10	7.30
32	5	3.65
33	9	6.57
34	2	1.46
Total	137	100.00

- **cb081_3_3_ : Province**

	No	%
03	1	1.33
05	3	4.00
06	3	4.00
07	3	4.00
08	1	1.33
10	5	6.67
12	3	4.00
13	1	1.33
14	9	12.00
15	2	2.67
16	5	6.67
17	1	1.33
20	1	1.33
21	8	10.67
24	2	2.67

25	2	2.67
26	1	1.33
27	4	5.33
28	2	2.67
29	5	6.67
32	5	6.67
33	4	5.33
34	4	5.33
Total	75	100.00

- **cb081_3_4_ : Province**

	No	%
05	3	7.50
06	1	2.50
09	1	2.50
10	3	7.50
14	6	15.00
16	3	7.50
17	2	5.00
21	7	17.50
25	1	2.50
26	1	2.50
27	3	7.50
28	1	2.50
29	4	10.00
32	1	2.50
34	3	7.50
Total	40	100.00

- **cb081_3_5_ : Province**

	No	%
05	1	5.88
10	1	5.88
14	5	29.41
16	1	5.88
17	1	5.88
21	4	23.53
27	2	11.76
29	1	5.88
32	1	5.88
Total	17	100.00

- **cb081_3_6_ : Province**

	No	%

14	2	40.00
21	3	60.00
Total	5	100.00

- **cb081_3_7_ : Province**

	No	%
21	2	100.00
Total	2	100.00

- **cb081_4_ : Birth Place of this Child**

	No	%
1 This Village/Neighborhood	2,137	86.76
2 Another Village/Neighborhood in this County/City	243	9.87
3 Another County/City in this Province	46	1.87
4 Another Province	37	1.50
Total	2,463	100.00

- **cb081_4_1_ : City**

	No	%
01	5	5.10
02	1	1.02
04	1	1.02
05	1	1.02
11	11	11.22
16	4	4.08
24	11	11.22
27	4	4.08
28	1	1.02
40	25	25.51
46	3	3.06
49	2	2.04
51	1	1.02
53	1	1.02
55	8	8.16
63	1	1.02
74	2	2.04
82	8	8.16
86	1	1.02
88	1	1.02
96	1	1.02
99	5	5.10
Total	98	100.00

- **cb081_4_2_ : City**

	No	%
01	9	6.98
02	1	0.78
04	5	3.88
05	2	1.55
11	9	6.98
16	4	3.10
24	14	10.85
27	6	4.65
28	2	1.55
40	18	13.95
46	4	3.10
49	6	4.65
51	1	0.78
53	7	5.43
55	12	9.30
60	1	0.78
63	1	0.78
74	4	3.10
82	15	11.63
86	2	1.55
88	1	0.78
99	5	3.88
Total	129	100.00

- **cb081_4_3_ : City**

	No	%
01	5	7.14
04	2	2.86
05	2	2.86
11	3	4.29
16	2	2.86
24	7	10.00
27	2	2.86
28	3	4.29
40	14	20.00
46	2	2.86
49	4	5.71
51	1	1.43
53	5	7.14
55	6	8.57
60	1	1.43
74	2	2.86
82	5	7.14
86	1	1.43
88	1	1.43
99	2	2.86

Total	70	100.00

- **cb081_4_4_ : City**

	No	%
01	4	11.11
04	2	5.56
05	2	5.56
11	2	5.56
16	1	2.78
24	3	8.33
27	2	5.56
28	2	5.56
40	6	16.67
51	1	2.78
53	2	5.56
55	2	5.56
63	1	2.78
82	5	13.89
99	1	2.78
Total	36	100.00

- **cb081_4_5_ : City**

	No	%
01	1	6.25
11	1	6.25
18	1	6.25
24	1	6.25
27	2	12.50
40	6	37.50
51	1	6.25
55	3	18.75
Total	16	100.00

- **cb081_4_6_ : City**

	No	%
27	1	20.00
40	1	20.00
55	3	60.00
Total	5	100.00

- **cb081_4_7_ : City**

	No	%
40	1	50.00
55	1	50.00
Total	2	100.00

- **cb081_5_ : Birth Place of this Child**

	No	%
1 This Village/Neighborhood	1,053	87.17
2 Another Village/Neighborhood in this County/City	121	10.02
3 Another County/City in this Province	17	1.41
4 Another Province	17	1.41
Total	1,208	100.00

- **cb081_5_1_ : County**

	No	%
02	2	2.38
04	2	2.38
06	10	11.90
16	1	1.19
23	1	1.19
28	2	2.38
31	10	11.90
33	1	1.19
37	1	1.19
43	5	5.95
44	4	4.76
45	2	2.38
46	4	4.76
49	1	1.19
51	2	2.38
54	8	9.52
56	1	1.19
58	1	1.19
59	6	7.14
63	14	16.67
76	2	2.38
78	2	2.38
81	2	2.38
Total	84	100.00

- **cb081_5_2_ : County**

	No	%
02	9	7.76

03	4	3.45
04	3	2.59
06	10	8.62
23	1	0.86
28	1	0.86
31	12	10.34
33	1	0.86
38	1	0.86
39	4	3.45
43	3	2.59
44	3	2.59
45	2	1.72
46	5	4.31
49	1	0.86
51	5	4.31
54	9	7.76
59	11	9.48
63	14	12.07
75	1	0.86
76	7	6.03
78	4	3.45
81	4	3.45
91	1	0.86
Total	116	100.00

- **cb081_5_3_ : County**

	No	%
02	3	4.69
03	1	1.56
04	5	7.81
06	3	4.69
23	1	1.56
28	3	4.69
31	9	14.06
39	1	1.56
43	3	4.69
44	3	4.69
45	1	1.56
51	2	3.13
54	2	3.13
57	1	1.56
59	5	7.81
63	12	18.75
76	1	1.56
78	2	3.13
81	4	6.25
91	2	3.13
Total	64	100.00

- **cb081_5_4_ : County**

	No	%
02	3	9.09
03	2	6.06
06	4	12.12
16	1	3.03
28	1	3.03
31	2	6.06
44	2	6.06
51	2	6.06
54	2	6.06
57	1	3.03
59	4	12.12
63	3	9.09
76	1	3.03
78	1	3.03
81	2	6.06
91	1	3.03
92	1	3.03
Total	33	100.00

- **cb081_5_5_ : County**

	No	%
02	1	7.14
03	1	7.14
06	1	7.14
28	1	7.14
31	1	7.14
37	1	7.14
44	1	7.14
54	2	14.29
57	1	7.14
59	1	7.14
63	2	14.29
76	1	7.14
Total	14	100.00

- **cb081_5_6_ : County**

	No	%
31	1	25.00
37	1	25.00
54	1	25.00
59	1	25.00
Total	4	100.00

- **cb081_5_7_ : County**

	No	%
37	1	100.00
Total	1	100.00

- **cb081_6_ : Birth Place of this Child**

	No	%
1 This Village/Neighborhood	439	87.80
2 Another Village/Neighborhood in this County/City	54	10.80
3 Another County/City in this Province	2	0.40
4 Another Province	5	1.00
Total	500	100.00

- **cb081_7_ : Birth Place of this Child**

	No	%
1 This Village/Neighborhood	166	88.30
2 Another Village/Neighborhood in this County/City	19	10.11
3 Another County/City in this Province	1	0.53
4 Another Province	2	1.06
Total	188	100.00

- **cb081_8_ : Birth Place of this Child**

	No	%
1 This Village/Neighborhood	50	80.65
2 Another Village/Neighborhood in this County/City	10	16.13
4 Another Province	2	3.23
Total	62	100.00

- **cb081_9_ : Birth Place of this Child**

	No	%
1 This Village/Neighborhood	16	72.73
2 Another Village/Neighborhood in this County/City	5	22.73
5 Abroad	1	4.55
Total	22	100.00

- **cb081_10_ : Birth Place of this Child**

	No	%

1 This Village/Neighborhood	6	100.00
Total	6	100.00

- **cb081_11_ : Birth Place of this Child**

No Observations

- **cb081_12_ : Birth Place of this Child**

No Observations

- **cb081_13_ : Birth Place of this Child**

No Observations

- **cb081_14_ : Birth Place of this Child**

No Observations

- **cb081_23_ : Birth Place of this Child**

	No	%
1 This Village/Neighborhood	1	100.00
Total	1	100.00

- **cb081_26_ : Birth Place of this Child**

	No	%
2 Another Village/Neighborhood in this County/City	1	100.00
Total	1	100.00

- **cb082_1_ : Is Her/His Current Hukou Status the same as Born**

	No	%

1 Yes	2,631	63.80
2 No	1,493	36.20
Total	4,124	100.00

- **cb082_2_ : Is Her/His Current Hukou Status the same as Born**

	No	%
1 Yes	3,617	57.37
2 No	2,688	42.63
Total	6,305	100.00

- **cb082_3_ : Is Her/His Current Hukou Status the same as Born**

	No	%
1 Yes	2,182	50.06
2 No	2,177	49.94
Total	4,359	100.00

- **cb082_4_ : Is Her/His Current Hukou Status the same as Born**

	No	%
1 Yes	1,115	45.44
2 No	1,339	54.56
Total	2,454	100.00

- **cb082_5_ : Is Her/His Current Hukou Status the same as Born**

	No	%
1 Yes	517	42.94
2 No	687	57.06
Total	1,204	100.00

- **cb082_6_ : Is Her/His Current Hukou Status the same as Born**

	No	%
1 Yes	212	42.57
2 No	286	57.43
Total	498	100.00

- **cb082_7_ : Is Her/His Current Hukou Status the same as Born**

	No	%
1 Yes	83	44.15
2 No	105	55.85
Total	188	100.00

- **cb082_8_ : Is Her/His Current Hukou Status the same as Born**

	No	%
1 Yes	25	40.98
2 No	36	59.02
Total	61	100.00

- **cb082_9_ : Is Her/His Current Hukou Status the same as Born**

	No	%
1 Yes	11	50.00
2 No	11	50.00
Total	22	100.00

- **cb082_10_ : Is Her/His Current Hukou Status the same as Born**

	No	%
1 Yes	3	50.00
2 No	3	50.00
Total	6	100.00

- **cb082_11_ : Is Her/His Current Hukou Status the same as Born**

No Observations

- **cb082_12_ : Is Her/His Current Hukou Status the same as Born**

No Observations

- **cb082_13_ : Is Her/His Current Hukou Status the same as Born**

No Observations

- **cb082_14_ : Is Her/His Current Hukou Status the same as Born**

No Observations

- **cb082_23_ : Is Her/His Current Hukou Status the same as Born**

	No	%
2 No	1	100.00
Total	1	100.00

- **cb083_1_ : How did this Child's Hukou Status or Location Change**

	No	%
1 Both Hukou status and location have changed	520	33.92
2 Only Hukou status has changed	59	3.85
3 Only Hukou location has changed	954	62.23
Total	1,533	100.00

- **cb083_2_ : How did this Child's Hukou Status or Location Change**

	No	%
1 Both Hukou status and location have changed	694	25.44
2 Only Hukou status has changed	85	3.12
3 Only Hukou location has changed	1,949	71.44
Total	2,728	100.00

- **cb083_3_ : How did this Child's Hukou Status or Location Change**

	No	%
1 Both Hukou status and location have changed	462	20.93
2 Only Hukou status has changed	34	1.54
3 Only Hukou location has changed	1,711	77.53
Total	2,207	100.00

- **cb083_4_ : How did this Child's Hukou Status or Location Change**

	No	%
1 Both Hukou status and location have changed	233	17.26

2 Only Hukou status has changed	19	1.41
3 Only Hukou location has changed	1,098	81.33
Total	1,350	100.00

- **cb083_5_ : How did this Child's Hukou Status or Location Change**

	No	%
1 Both Hukou status and location have changed	117	16.91
2 Only Hukou status has changed	7	1.01
3 Only Hukou location has changed	568	82.08
Total	692	100.00

- **cb083_6_ : How did this Child's Hukou Status or Location Change**

	No	%
1 Both Hukou status and location have changed	39	13.59
2 Only Hukou status has changed	3	1.05
3 Only Hukou location has changed	245	85.37
Total	287	100.00

- **cb083_7_ : How did this Child's Hukou Status or Location Change**

	No	%
1 Both Hukou status and location have changed	15	14.02
2 Only Hukou status has changed	2	1.87
3 Only Hukou location has changed	90	84.11
Total	107	100.00

- **cb083_8_ : How did this Child's Hukou Status or Location Change**

	No	%
1 Both Hukou status and location have changed	6	16.67
3 Only Hukou location has changed	30	83.33
Total	36	100.00

- **cb083_9_ : How did this Child's Hukou Status or Location Change**

	No	%
1 Both Hukou status and location have changed	2	18.18
2 Only Hukou status has changed	1	9.09
3 Only Hukou location has changed	8	72.73
Total	11	100.00

- **cb083_10_ : How did this Child's Hukou Status or Location Change**

	No	%
1 Both Hukou status and location have changed	2	66.67
2 Only Hukou status has changed	1	33.33
Total	3	100.00

- **cb083_23_ : How did this Child's Hukou Status or Location Change**

	No	%
3 Only Hukou location has changed	1	100.00
Total	1	100.00

- **cb084_1_ : This Child's First Hukou Status**

	No	%
1 Agricultural Hukou	537	95.89
2 Non-agricultual Hukou	23	4.11
Total	560	100.00

- **cb084_2_ : This Child's First Hukou Status**

	No	%
1 Agricultural Hukou	742	96.74
2 Non-agricultual Hukou	25	3.26
Total	767	100.00

- **cb084_3_ : This Child's First Hukou Status**

	No	%
1 Agricultural Hukou	473	96.53
2 Non-agricultual Hukou	17	3.47
Total	490	100.00

- **cb084_4_ : This Child's First Hukou Status**

	No	%
1 Agricultural Hukou	242	97.19
2 Non-agricultual Hukou	7	2.81
Total	249	100.00

- **cb084_5_ : This Child's First Hukou Status**

	No	%
1 Agricultural Hukou	117	96.69
2 Non-agricultual Hukou	4	3.31
Total	121	100.00

- **cb084_6_ : This Child's First Hukou Status**

	No	%
1 Agricultural Hukou	37	90.24
2 Non-agricultual Hukou	4	9.76
Total	41	100.00

- **cb084_7_ : This Child's First Hukou Status**

	No	%
1 Agricultural Hukou	17	100.00
Total	17	100.00

- **cb084_8_ : This Child's First Hukou Status**

	No	%
1 Agricultural Hukou	6	100.00
Total	6	100.00

- **cb084_9_ : This Child's First Hukou Status**

	No	%
1 Agricultural Hukou	3	100.00
Total	3	100.00

- **cb084_10_ : This Child's First Hukou Status**

	No	%
1 Agricultural Hukou	3	100.00
Total	3	100.00

- **cb085_1_ : This Child's First Hukou Location**

	No	%

1 This Village/Neighborhood	1,091	75.29
2 Another Village/Neighborhood in this County/City	239	16.49
3 Another County/City in this Province	60	4.14
4 Another Province	58	4.00
5 Abroad	1	0.07
Total	1,449	100.00

- **cb085_1_1_ : City**

	No	%
01	2	3.33
02	1	1.67
04	2	3.33
05	1	1.67
11	8	13.33
16	1	1.67
24	3	5.00
27	2	3.33
40	10	16.67
46	4	6.67
49	5	8.33
53	1	1.67
55	4	6.67
60	5	8.33
63	1	1.67
74	5	8.33
82	3	5.00
96	2	3.33
Total	60	100.00

- **cb085_1_2_ : City**

	No	%
01	5	5.81
02	1	1.16
04	5	5.81
11	8	9.30
16	1	1.16
24	2	2.33
27	2	2.33
28	1	1.16
35	1	1.16
40	11	12.79
46	16	18.60
49	3	3.49
51	1	1.16
53	3	3.49
55	3	3.49

60	5	5.81
63	4	4.65
74	2	2.33
82	9	10.47
86	1	1.16
96	2	2.33
Total	86	100.00

- **cb085_1_3_ : City**

	No	%
01	3	5.88
04	1	1.96
05	1	1.96
07	1	1.96
11	4	7.84
16	1	1.96
17	1	1.96
24	2	3.92
28	1	1.96
40	6	11.76
46	5	9.80
49	1	1.96
53	2	3.92
55	2	3.92
60	5	9.80
63	3	5.88
74	3	5.88
82	7	13.73
86	1	1.96
96	1	1.96
Total	51	100.00

- **cb085_1_4_ : City**

	No	%
01	2	8.33
07	1	4.17
11	2	8.33
40	5	20.83
46	3	12.50
55	2	8.33
60	1	4.17
63	1	4.17
74	1	4.17
82	4	16.67
86	1	4.17
96	1	4.17
Total	24	100.00

- **cb085_1_5_ : City**

	No	%
11	1	14.29
27	1	14.29
40	3	42.86
46	1	14.29
86	1	14.29
Total	7	100.00

- **cb085_1_6_ : City**

	No	%
40	1	50.00
46	1	50.00
Total	2	100.00

- **cb085_2_ : This Child's First Hukou Location**

	No	%
1 This Village/Neighborhood	2,121	80.86
2 Another Village/Neighborhood in this County/City	331	12.62
3 Another County/City in this Province	86	3.28
4 Another Province	85	3.24
Total	2,623	100.00

- **cb085_2_1_ : County**

	No	%
02	2	3.57
03	1	1.79
04	2	3.57
06	3	5.36
28	2	3.57
31	7	12.50
33	3	5.36
38	1	1.79
43	2	3.57
44	1	1.79
46	3	5.36
51	2	3.57
54	3	5.36
59	3	5.36
63	12	21.43

73	1	1.79
76	3	5.36
81	4	7.14
92	1	1.79
Total	56	100.00

- **cb085_2_2_ : County**

	No	%
02	5	6.17
03	2	2.47
06	4	4.94
08	1	1.23
13	1	1.23
28	4	4.94
31	9	11.11
33	1	1.23
38	1	1.23
43	3	3.70
44	1	1.23
46	3	3.70
51	1	1.23
54	2	2.47
59	13	16.05
63	18	22.22
76	6	7.41
78	1	1.23
81	4	4.94
90	1	1.23
Total	81	100.00

- **cb085_2_3_ : County**

	No	%
02	3	6.38
03	1	2.13
06	2	4.26
28	2	4.26
31	3	6.38
33	1	2.13
39	1	2.13
43	5	10.64
44	2	4.26
46	3	6.38
51	1	2.13
54	2	4.26
59	1	2.13
63	14	29.79
76	2	4.26

81	4	8.51
Total	47	100.00

- **cb085_2_4_ : County**

	No	%
02	2	10.00
04	1	5.00
31	1	5.00
43	1	5.00
46	3	15.00
49	1	5.00
59	2	10.00
63	5	25.00
76	1	5.00
81	3	15.00
Total	20	100.00

- **cb085_2_5_ : County**

	No	%
28	1	14.29
43	1	14.29
54	1	14.29
63	3	42.86
81	1	14.29
Total	7	100.00

- **cb085_2_6_ : County**

	No	%
28	1	50.00
81	1	50.00
Total	2	100.00

- **cb085_3_ : This Child's First Hukou Location**

	No	%
1 This Village/Neighborhood	1,813	84.44
2 Another Village/Neighborhood in this County/City	233	10.85
3 Another County/City in this Province	53	2.47
4 Another Province	48	2.24
Total	2,147	100.00

- **cb085_3_1_ : Province**

	No	%
02	1	1.69
05	3	5.08
06	4	6.78
08	1	1.69
09	2	3.39
10	3	5.08
11	1	1.69
12	3	5.08
13	2	3.39
14	6	10.17
16	3	5.08
17	2	3.39
18	2	3.39
19	2	3.39
20	2	3.39
21	6	10.17
25	2	3.39
26	1	1.69
29	6	10.17
32	1	1.69
33	3	5.08
34	3	5.08
Total	59	100.00

- **cb085_3_2_ : Province**

	No	%
02	1	1.18
03	1	1.18
05	7	8.24
06	5	5.88
07	1	1.18
08	2	2.35
09	3	3.53
10	5	5.88
11	1	1.18
12	3	3.53
13	4	4.71
14	11	12.94
15	2	2.35
16	5	5.88
17	1	1.18
19	1	1.18
20	2	2.35
21	8	9.41
24	1	1.18
25	2	2.35

26	1	1.18
27	1	1.18
28	1	1.18
29	7	8.24
32	2	2.35
33	4	4.71
34	3	3.53
Total	85	100.00

- **cb085_3_3_ : Province**

	No	%
03	1	2.08
05	2	4.17
06	3	6.25
07	1	2.08
08	1	2.08
10	2	4.17
12	2	4.17
13	1	2.08
14	5	10.42
15	2	4.17
16	4	8.33
17	1	2.08
21	4	8.33
24	1	2.08
25	2	4.17
26	1	2.08
27	1	2.08
28	2	4.17
29	4	8.33
32	4	8.33
33	1	2.08
34	3	6.25
Total	48	100.00

- **cb085_3_4_ : Province**

	No	%
03	1	3.57
05	2	7.14
10	2	7.14
14	6	21.43
16	3	10.71
17	2	7.14
21	5	17.86
25	1	3.57
26	1	3.57
27	1	3.57

28	1	3.57
29	1	3.57
32	1	3.57
34	1	3.57
Total	28	100.00

- **cb085_3_5_ : Province**

	No	%
05	1	10.00
10	1	10.00
14	2	20.00
16	1	10.00
21	3	30.00
27	1	10.00
32	1	10.00
Total	10	100.00

- **cb085_3_6_ : Province**

	No	%
14	1	25.00
21	3	75.00
Total	4	100.00

- **cb085_3_7_ : Province**

	No	%
21	2	100.00
Total	2	100.00

- **cb085_4_ : This Child's First Hukou Location**

	No	%
1 This Village/Neighborhood	1,146	86.69
2 Another Village/Neighborhood in this County/City	124	9.38
3 Another County/City in this Province	24	1.82
4 Another Province	28	2.12
Total	1,322	100.00

- **cb085_4_1_ : City**

	No	%

01	3	5.88
02	1	1.96
04	1	1.96
05	1	1.96
11	7	13.73
16	1	1.96
24	4	7.84
27	1	1.96
40	14	27.45
46	1	1.96
49	1	1.96
51	1	1.96
53	2	3.92
55	3	5.88
63	1	1.96
82	5	9.80
88	1	1.96
99	3	5.88
Total	51	100.00

- **cb085_4_2_ : City**

	No	%
01	4	5.00
02	1	1.25
04	3	3.75
05	1	1.25
11	6	7.50
16	1	1.25
18	1	1.25
24	9	11.25
27	4	5.00
28	1	1.25
40	16	20.00
46	4	5.00
49	3	3.75
51	1	1.25
53	3	3.75
55	7	8.75
63	1	1.25
82	7	8.75
86	1	1.25
88	1	1.25
99	5	6.25
Total	80	100.00

- **cb085_4_3_ : City**

	No	%

01	4	9.30
04	1	2.33
05	2	4.65
11	2	4.65
24	3	6.98
28	2	4.65
40	8	18.60
46	1	2.33
49	2	4.65
51	1	2.33
53	4	9.30
55	5	11.63
60	1	2.33
74	1	2.33
82	2	4.65
86	1	2.33
88	1	2.33
99	2	4.65
Total	43	100.00

- **cb085_4_4_ : City**

	No	%
01	4	16.67
04	1	4.17
11	2	8.33
24	1	4.17
27	2	8.33
28	1	4.17
40	5	20.83
51	1	4.17
53	2	8.33
55	1	4.17
63	1	4.17
82	3	12.50
Total	24	100.00

- **cb085_4_5_ : City**

	No	%
01	1	10.00
11	1	10.00
27	1	10.00
40	3	30.00
51	1	10.00
53	1	10.00
55	2	20.00
Total	10	100.00

- **cb085_4_6_ : City**

	No	%
27	1	25.00
55	2	50.00
82	1	25.00
Total	4	100.00

- **cb085_4_7_ : City**

	No	%
55	1	50.00
82	1	50.00
Total	2	100.00

- **cb085_5_ : This Child's First Hukou Location**

	No	%
1 This Village/Neighborhood	606	88.86
2 Another Village/Neighborhood in this County/City	58	8.50
3 Another County/City in this Province	7	1.03
4 Another Province	11	1.61
Total	682	100.00

- **cb085_5_1_ : County**

	No	%
02	1	2.33
04	2	4.65
06	5	11.63
23	1	2.33
31	4	9.30
33	1	2.33
43	3	6.98
44	1	2.33
46	3	6.98
51	1	2.33
54	5	11.63
56	1	2.33
58	1	2.33
59	1	2.33
63	6	13.95
75	1	2.33
76	3	6.98
78	2	4.65
81	1	2.33

Total	43	100.00

- **cb085_5_2_ : County**

	No	%
02	4	5.80
03	3	4.35
04	2	2.90
06	5	7.25
13	1	1.45
16	1	1.45
23	1	1.45
28	1	1.45
31	12	17.39
33	1	1.45
39	2	2.90
43	3	4.35
44	1	1.45
45	1	1.45
46	2	2.90
49	1	1.45
51	2	2.90
54	3	4.35
57	1	1.45
59	4	5.80
63	10	14.49
75	1	1.45
76	3	4.35
78	2	2.90
81	2	2.90
Total	69	100.00

- **cb085_5_3_ : County**

	No	%
02	3	8.11
03	1	2.70
04	2	5.41
06	1	2.70
16	1	2.70
23	1	2.70
31	6	16.22
39	1	2.70
43	4	10.81
51	1	2.70
57	2	5.41
59	1	2.70
63	7	18.92
76	1	2.70

78	1	2.70
81	2	5.41
91	2	5.41
Total	37	100.00

- **cb085_5_4_ : County**

	No	%
02	3	13.64
03	2	9.09
06	3	13.64
16	1	4.55
31	3	13.64
43	1	4.55
57	2	9.09
59	2	9.09
63	2	9.09
76	1	4.55
81	1	4.55
91	1	4.55
Total	22	100.00

- **cb085_5_5_ : County**

	No	%
02	1	11.11
03	1	11.11
06	1	11.11
28	1	11.11
31	1	11.11
43	1	11.11
54	1	11.11
57	1	11.11
63	1	11.11
Total	9	100.00

- **cb085_5_6_ : County**

	No	%
06	1	33.33
59	2	66.67
Total	3	100.00

- **cb085_5_7_ : County**

	No	%
06	1	100.00
Total	1	100.00

- **cb085_6_ : This Child's First Hukou Location**

	No	%
1 This Village/Neighborhood	251	88.69
2 Another Village/Neighborhood in this County/City	26	9.19
3 Another County/City in this Province	2	0.71
4 Another Province	4	1.41
Total	283	100.00

- **cb085_7_ : This Child's First Hukou Location**

	No	%
1 This Village/Neighborhood	91	88.35
2 Another Village/Neighborhood in this County/City	10	9.71
4 Another Province	2	1.94
Total	103	100.00

- **cb085_8_ : This Child's First Hukou Location**

	No	%
1 This Village/Neighborhood	30	83.33
2 Another Village/Neighborhood in this County/City	5	13.89
4 Another Province	1	2.78
Total	36	100.00

- **cb085_9_ : This Child's First Hukou Location**

	No	%
1 This Village/Neighborhood	7	70.00
2 Another Village/Neighborhood in this County/City	3	30.00
Total	10	100.00

- **cb085_10_ : This Child's First Hukou Location**

	No	%
1 This Village/Neighborhood	2	100.00
Total	2	100.00

- **cb085_23_ : This Child's First Hukou Location**

	No	%
1 This Village/Neighborhood	1	100.00
Total	1	100.00

- **cc001 : Num. of Your Siblings are Still Alive**

	No	%
0	1,122	11.11
1	1,287	12.75
2	1,666	16.50
3	1,942	19.24
4	1,705	16.89
5	1,260	12.48
6	700	6.93
7	286	2.83
8	94	0.93
9	24	0.24
10	6	0.06
11	3	0.03
Total	10,095	100.00

- **cc002_1 : Num. of Living Older Brothers**

	No	%
0	4,994	55.54
1	2,650	29.47
2	957	10.64
3	297	3.30
4	75	0.83
5	13	0.14
6	4	0.04
7	1	0.01
8	1	0.01
Total	8,992	100.00

- **cc002_2 : Num. of Living Younger Brothers**

	No	%
0	3,266	36.33
1	3,049	33.92
2	1,706	18.98
3	677	7.53
4	214	2.38
5	57	0.63

6	11	0.12
7	8	0.09
8	1	0.01
Total	8,989	100.00

- **cc002_3 : Num. of Living Older Sisters**

	No	%
0	4,915	54.69
1	2,614	29.09
2	1,011	11.25
3	326	3.63
4	89	0.99
5	28	0.31
6	3	0.03
7	1	0.01
Total	8,987	100.00

- **cc002_4 : Num. of Living Younger Sisters**

	No	%
0	3,718	41.38
1	2,813	31.30
2	1,496	16.65
3	668	7.43
4	206	2.29
5	63	0.70
6	15	0.17
7	6	0.07
8	1	0.01
Total	8,986	100.00

- **cc003 : Num. of Dead Biological Siblings**

	No	%
0	5,978	59.48
1	2,365	23.53
2	928	9.23
3	436	4.34
4	178	1.77
5	83	0.83
6	45	0.45
7	16	0.16
8	7	0.07
9	6	0.06
10	4	0.04
11	3	0.03

12	1	0.01
14	1	0.01
Total	10,051	100.00

- **cc004_1 : Num. of Dead Older Brothers**

	No	%
0	2,038	49.95
1	1,513	37.08
2	386	9.46
3	100	2.45
4	25	0.61
5	10	0.25
6	7	0.17
12	1	0.02
Total	4,080	100.00

- **cc004_2 : Num. of Dead Younger Brothers**

	No	%
0	2,938	72.06
1	958	23.50
2	141	3.46
3	31	0.76
4	6	0.15
5	2	0.05
11	1	0.02
Total	4,077	100.00

- **cc004_3 : Num. of Dead Older Sisters**

	No	%
0	2,500	61.32
1	1,200	29.43
2	256	6.28
3	85	2.08
4	25	0.61
5	8	0.20
6	1	0.02
7	1	0.02
8	1	0.02
Total	4,077	100.00

- **cc004_4 : Num. of Dead Younger Sisters**

	No	%
0	3,298	80.89
1	660	16.19
2	96	2.35
3	13	0.32
4	9	0.22
5	1	0.02
Total	4,077	100.00

- **cc005 : Who Answers Questions about Spouse's Siblings**

	No	%
1 The family respondent	6,880	70.53
2 The spouse	2,875	29.47
Total	9,755	100.00

- **cc006 : Num. of Your Spouse's Siblings are Still Alive**

	No	%
0	1,142	11.88
1	1,281	13.32
2	1,505	15.65
3	1,857	19.31
4	1,639	17.05
5	1,211	12.59
6	581	6.04
7	265	2.76
8	90	0.94
9	39	0.41
10	4	0.04
11	1	0.01
Total	9,615	100.00

- **cc007_1 : Num. of Spouse's Living Older Brothers**

	No	%
0	4,737	55.83
1	2,447	28.84
2	925	10.90
3	298	3.51
4	61	0.72
5	13	0.15
6	3	0.04
7	1	0.01
Total	8,485	100.00

- **cc007_2 : Num. of Spouse's Living Younger Brothers**

	No	%
0	3,079	36.31
1	2,790	32.90
2	1,629	19.21
3	712	8.40
4	200	2.36
5	56	0.66
6	12	0.14
7	2	0.02
Total	8,480	100.00

- **cc007_3 : Num. of Spouse's Living Older Sisters**

	No	%
0	4,666	55.04
1	2,445	28.84
2	959	11.31
3	313	3.69
4	72	0.85
5	18	0.21
6	3	0.04
7	1	0.01
9	1	0.01
Total	8,478	100.00

- **cc007_4 : Num. of Spouse's Living Younger Sisters**

	No	%
0	3,542	41.78
1	2,698	31.82
2	1,411	16.64
3	556	6.56
4	191	2.25
5	71	0.84
6	9	0.11
Total	8,478	100.00

- **cc008 : Num. of Spouse's Dead Biological Siblings**

	No	%
0	5,855	61.31
1	2,180	22.83
2	876	9.17
3	360	3.77

4	161	1.69
5	58	0.61
6	38	0.40
7	9	0.09
8	6	0.06
9	1	0.01
10	2	0.02
11	1	0.01
13	1	0.01
14	2	0.02
Total	9,550	100.00

- **cc009_1 : Num. of Spouse's Dead Older Brothers**

	No	%
0	1,906	51.54
1	1,342	36.29
2	328	8.87
3	87	2.35
4	24	0.65
5	4	0.11
6	4	0.11
7	1	0.03
8	1	0.03
14	1	0.03
Total	3,698	100.00

- **cc009_2 : Num. of Spouse's Dead Younger Brothers**

	No	%
0	2,646	71.57
1	870	23.53
2	153	4.14
3	23	0.62
4	5	0.14
Total	3,697	100.00

- **cc009_3 : Num. of Spouse's Dead Older Sisters**

	No	%
0	2,360	63.87
1	990	26.79
2	267	7.23
3	57	1.54
4	16	0.43
5	3	0.08
9	1	0.03

12	1	0.03
Total	3,695	100.00

- **cc009_4 : Num. of Spouse's Dead Younger Sisters**

	No	%
0	2,974	80.53
1	621	16.82
2	81	2.19
3	11	0.30
4	5	0.14
7	1	0.03
Total	3,693	100.00

- **cd001_1_ : Whom Does Your Parent Live with**

	No	%
1 By him/herself	783	58.26
2 With my/my spouse's older brother	94	6.99
3 With my/my spouse's younger brother	348	25.89
4 With my/my spouse's older sister	15	1.12
5 With my/my spouse's younger sister	30	2.23
6 Take turns in children's homes	34	2.53
7 Nursing home	3	0.22
8 Other	37	2.75
Total	1,344	100.00

- **cd001_2_ : Whom Does Your Parent Live with**

	No	%
1 By him/herself	1,101	47.40
2 With my/my spouse's older brother	228	9.81
3 With my/my spouse's younger brother	768	33.06
4 With my/my spouse's older sister	29	1.25
5 With my/my spouse's younger sister	60	2.58
6 Take turns in children's homes	84	3.62
7 Nursing home	9	0.39
8 Other	44	1.89
Total	2,323	100.00

- **cd001_3_ : Whom Does Your Parent Live with**

	No	%
1 By him/herself	785	59.51
2 With my/my spouse's older brother	85	6.44

	No	%
3 With my/my spouse's younger brother	356	26.99
4 With my/my spouse's older sister	5	0.38
5 With my/my spouse's younger sister	23	1.74
6 Take turns in children's homes	31	2.35
7 Nursing home	6	0.45
8 Other	28	2.12
Total	1,319	100.00

- **cd001_4_ : Whom Does Your Parent Live with**

	No	%
1 By him/herself	1,112	48.64
2 With my/my spouse's older brother	213	9.32
3 With my/my spouse's younger brother	746	32.63
4 With my/my spouse's older sister	17	0.74
5 With my/my spouse's younger sister	55	2.41
6 Take turns in children's homes	82	3.59
7 Nursing home	8	0.35
8 Other	53	2.32
Total	2,286	100.00

- **cd002_1_ : How Often do You/Your Spouse See Your Parent**

	No	%
1 Almost every day	367	27.37
2 2-3 times a week	130	9.69
3 Once a week	135	10.07
4 Every two weeks	126	9.40
5 Once a month	197	14.69
6 Once every three months	140	10.44
7 Once every six months	97	7.23
8 Once a year	75	5.59
9 Almost never	30	2.24
10 Other	44	3.28
Total	1,341	100.00

- **cd002_2_ : How Often do You/Your Spouse See Your Parent**

	No	%
1 Almost every day	650	27.97
2 2-3 times a week	238	10.24
3 Once a week	224	9.64
4 Every two weeks	225	9.68
5 Once a month	348	14.97
6 Once every three months	240	10.33
7 Once every six months	142	6.11
8 Once a year	146	6.28

9 Almost never	54	2.32
10 Other	57	2.45
Total	2,324	100.00

- **cd002_3_ : How Often do You/Your Spouse See Your Parent**

	No	%
1 Almost every day	370	28.14
2 2-3 times a week	116	8.82
3 Once a week	107	8.14
4 Every two weeks	105	7.98
5 Once a month	193	14.68
6 Once every three months	139	10.57
7 Once every six months	95	7.22
8 Once a year	105	7.98
9 Almost never	47	3.57
10 Other	38	2.89
Total	1,315	100.00

- **cd002_4_ : How Often do You/Your Spouse See Your Parent**

	No	%
1 Almost every day	616	26.93
2 2-3 times a week	208	9.09
3 Once a week	223	9.75
4 Every two weeks	221	9.66
5 Once a month	340	14.87
6 Once every three months	230	10.06
7 Once every six months	159	6.95
8 Once a year	169	7.39
9 Almost never	61	2.67
10 Other	60	2.62
Total	2,287	100.00

- **cd002_5_ : How Often do You/Your Spouse See Your Parent**

	No	%
1 Almost every day	4	25.00
2 2-3 times a week	3	18.75
3 Once a week	1	6.25
4 Every two weeks	3	18.75
5 Once a month	2	12.50
6 Once every three months	1	6.25
7 Once every six months	1	6.25
10 Other	1	6.25
Total	16	100.00

- **cd002_6_ : How Often do You/Your Spouse See Your Parent**

	No	%
5 Once a month	2	100.00
Total	2	100.00

- **cd002_7_ : How Often do You/Your Spouse See Your Parent**

	No	%
5 Once a month	2	100.00
Total	2	100.00

- **cd002_8_ : How Often do You/Your Spouse See Your Parent**

	No	%
3 Once a week	1	100.00
Total	1	100.00

- **cd002_9_ : How Often do You/Your Spouse See Your Parent**

	No	%
5 Once a month	1	100.00
Total	1	100.00

- **cd003_1_ : How Often do You See Child**

	No	%
1 Almost every day	928	22.54
2 2-3 times a week	239	5.81
3 Once a week	295	7.17
4 Every two weeks	221	5.37
5 Once a month	384	9.33
6 Once every three months	405	9.84
7 Once every six months	446	10.83
8 Once a year	842	20.45
9 Almost never	171	4.15
10 Other	186	4.52
Total	4,117	100.00

- **cd003_2_ : How Often do You See Child**

	No	%

1 Almost every day	1,331	21.14
2 2-3 times a week	393	6.24
3 Once a week	483	7.67
4 Every two weeks	407	6.46
5 Once a month	673	10.69
6 Once every three months	595	9.45
7 Once every six months	660	10.48
8 Once a year	1,303	20.69
9 Almost never	217	3.45
10 Other	235	3.73
Total	6,297	100.00

- **cd003_3_ : How Often do You See Child**

	No	%
1 Almost every day	800	18.31
2 2-3 times a week	262	6.00
3 Once a week	344	7.87
4 Every two weeks	329	7.53
5 Once a month	488	11.17
6 Once every three months	421	9.64
7 Once every six months	463	10.60
8 Once a year	904	20.69
9 Almost never	198	4.53
10 Other	160	3.66
Total	4,369	100.00

- **cd003_4_ : How Often do You See Child**

	No	%
1 Almost every day	397	16.13
2 2-3 times a week	142	5.77
3 Once a week	181	7.35
4 Every two weeks	208	8.45
5 Once a month	275	11.17
6 Once every three months	256	10.40
7 Once every six months	265	10.77
8 Once a year	494	20.07
9 Almost never	107	4.35
10 Other	136	5.53
Total	2,461	100.00

- **cd003_5_ : How Often do You See Child**

	No	%
1 Almost every day	195	16.17
2 2-3 times a week	65	5.39

3 Once a week	92	7.63
4 Every two weeks	106	8.79
5 Once a month	148	12.27
6 Once every three months	143	11.86
7 Once every six months	116	9.62
8 Once a year	227	18.82
9 Almost never	57	4.73
10 Other	57	4.73
Total	1,206	100.00

- **cd003_6_ : How Often do You See Child**

	No	%
1 Almost every day	68	13.52
2 2-3 times a week	34	6.76
3 Once a week	44	8.75
4 Every two weeks	45	8.95
5 Once a month	49	9.74
6 Once every three months	62	12.33
7 Once every six months	43	8.55
8 Once a year	99	19.68
9 Almost never	36	7.16
10 Other	23	4.57
Total	503	100.00

- **cd003_7_ : How Often do You See Child**

	No	%
1 Almost every day	37	19.58
2 2-3 times a week	8	4.23
3 Once a week	15	7.94
4 Every two weeks	12	6.35
5 Once a month	16	8.47
6 Once every three months	19	10.05
7 Once every six months	19	10.05
8 Once a year	40	21.16
9 Almost never	16	8.47
10 Other	7	3.70
Total	189	100.00

- **cd003_8_ : How Often do You See Child**

	No	%
1 Almost every day	8	12.90
2 2-3 times a week	3	4.84
3 Once a week	6	9.68
4 Every two weeks	2	3.23

	No	%
5 Once a month	5	8.06
6 Once every three months	9	14.52
7 Once every six months	8	12.90
8 Once a year	8	12.90
9 Almost never	9	14.52
10 Other	4	6.45
Total	62	100.00

- **cd003_9_ : How Often do You See Child**

	No	%
1 Almost every day	6	28.57
3 Once a week	5	23.81
5 Once a month	2	9.52
6 Once every three months	2	9.52
8 Once a year	4	19.05
9 Almost never	1	4.76
10 Other	1	4.76
Total	21	100.00

- **cd003_10_ : How Often do You See Child**

	No	%
1 Almost every day	2	33.33
3 Once a week	1	16.67
6 Once every three months	1	16.67
7 Once every six months	2	33.33
Total	6	100.00

- **cd003_11_ : How Often do You See Child**

	No	%
4 Every two weeks	1	100.00
Total	1	100.00

- **cd003_12_ : How Often do You See Child**

	No	%
5 Once a month	1	100.00
Total	1	100.00

- **cd003_13_ : How Often do You See Child**

	No	%
6 Once every three months	1	100.00
Total	1	100.00

- **cd003_14_ : How Often do You See Child**

	No	%
5 Once a month	1	100.00
Total	1	100.00

- **cd004_1_ : How Often do You Contact with Child**

	No	%
1 Almost every day	534	13.01
2 2-3 times a week	558	13.60
3 Once a week	689	16.79
4 Every two weeks	466	11.36
5 Once a month	458	11.16
6 Once every three months	171	4.17
7 Once every six months	73	1.78
8 Once a year	35	0.85
9 Almost never	828	20.18
10 Other	291	7.09
Total	4,103	100.00

- **cd004_2_ : How Often do You Contact with Child**

	No	%
1 Almost every day	657	10.46
2 2-3 times a week	813	12.94
3 Once a week	1,020	16.23
4 Every two weeks	769	12.24
5 Once a month	813	12.94
6 Once every three months	303	4.82
7 Once every six months	116	1.85
8 Once a year	69	1.10
9 Almost never	1,320	21.01
10 Other	404	6.43
Total	6,284	100.00

- **cd004_3_ : How Often do You Contact with Child**

	No	%
1 Almost every day	319	7.29

2 2-3 times a week	495	11.32
3 Once a week	682	15.60
4 Every two weeks	504	11.53
5 Once a month	643	14.70
6 Once every three months	222	5.08
7 Once every six months	102	2.33
8 Once a year	48	1.10
9 Almost never	1,075	24.58
10 Other	283	6.47
Total	4,373	100.00

- **cd004_4_ : How Often do You Contact with Child**

	No	%
1 Almost every day	124	5.03
2 2-3 times a week	252	10.22
3 Once a week	339	13.75
4 Every two weeks	279	11.32
5 Once a month	367	14.89
6 Once every three months	150	6.09
7 Once every six months	60	2.43
8 Once a year	41	1.66
9 Almost never	681	27.63
10 Other	172	6.98
Total	2,465	100.00

- **cd004_5_ : How Often do You Contact with Child**

	No	%
1 Almost every day	66	5.42
2 2-3 times a week	116	9.53
3 Once a week	137	11.26
4 Every two weeks	132	10.85
5 Once a month	179	14.71
6 Once every three months	63	5.18
7 Once every six months	22	1.81
8 Once a year	21	1.73
9 Almost never	377	30.98
10 Other	104	8.55
Total	1,217	100.00

- **cd004_6_ : How Often do You Contact with Child**

	No	%
1 Almost every day	27	5.31
2 2-3 times a week	46	9.06
3 Once a week	50	9.84

	No	%
4 Every two weeks	44	8.66
5 Once a month	62	12.20
6 Once every three months	43	8.46
7 Once every six months	16	3.15
8 Once a year	6	1.18
9 Almost never	164	32.28
10 Other	50	9.84
Total	508	100.00

- **cd004_7_ : How Often do You Contact with Child**

	No	%
1 Almost every day	12	6.28
2 2-3 times a week	14	7.33
3 Once a week	14	7.33
4 Every two weeks	11	5.76
5 Once a month	28	14.66
6 Once every three months	16	8.38
7 Once every six months	5	2.62
8 Once a year	1	0.52
9 Almost never	76	39.79
10 Other	14	7.33
Total	191	100.00

- **cd004_8_ : How Often do You Contact with Child**

	No	%
1 Almost every day	2	3.23
2 2-3 times a week	6	9.68
3 Once a week	5	8.06
4 Every two weeks	2	3.23
5 Once a month	9	14.52
6 Once every three months	5	8.06
7 Once every six months	1	1.61
8 Once a year	2	3.23
9 Almost never	26	41.94
10 Other	4	6.45
Total	62	100.00

- **cd004_9_ : How Often do You Contact with Child**

	No	%
3 Once a week	2	9.52
4 Every two weeks	2	9.52
5 Once a month	2	9.52
6 Once every three months	2	9.52
9 Almost never	9	42.86

10 Other	4	19.05
Total	21	100.00

- **cd004_10_ : How Often do You Contact with Child**

	No	%
3 Once a week	2	33.33
5 Once a month	1	16.67
7 Once every six months	1	16.67
9 Almost never	1	16.67
10 Other	1	16.67
Total	6	100.00

- **cd004_11_ : How Often do You Contact with Child**

	No	%
7 Once every six months	1	100.00
Total	1	100.00

- **cd004_12_ : How Often do You Contact with Child**

	No	%
4 Every two weeks	1	100.00
Total	1	100.00

- **cd004_13_ : How Often do You Contact with Child**

	No	%
6 Once every three months	1	100.00
Total	1	100.00

- **cd004_14_ : How Often do You Contact with Child**

	No	%
5 Once a month	1	100.00
Total	1	100.00

- **proxy : Interview Down By Proxy**

	No	%
0 No	9,912	97.20

1 Yes	286	2.80
Total	10,198	100.00

4 Family Transfer

- **householdID : Household ID**

A String Variable	
OBS:	10,122

- **ID : Individual ID**

A String Variable	
OBS:	10,122

- **communityID : Community ID**

A String Variable	
OBS:	10,122

- **ce001 : Any Transfer from Non-coresident Parents**

	No	%
1 Yes	91	2.63
2 No	3,370	97.37
Total	3,461	100.00

- **ce002_1 : Regular Monetary Support**

Mean	Min	Max	OBS
1,350.0	100.0	5,000.0	18

- **ce002_1_every : Time Interval**

	No	%
1 Per month	3	17.65
3 Per half year	1	5.88
4 Per year	13	76.47
Total	17	100.00

- **ce002_2 : Regular In-Kind Support**

	No	%

0	1	100.00
Total	1	100.00

- **ce002_3 : Non-Regular Monetary Support**

Mean	Min	Max	OBS
3,695.7	-9,999.0	60,000.0	83

- **ce002_4 : Non-Regular In-Kind Support**

Mean	Min	Max	OBS
1,782.1	0.0	20,000.0	28

- **ce004 : Any Transfer from Non-coresident Parents-in-Law**

	No	%
1 Yes	86	2.65
2 No	3,156	97.35
Total	3,242	100.00

- **ce005_1 : Regular Monetary Support**

Mean	Min	Max	OBS
979.4	0.0	6,000.0	17

- **ce005_1_every : Time Interval**

	No	%
	1	5.88
1 Per month	7	41.18
3 Per half year	1	5.88
4 Per year	8	47.06
Total	17	100.00

- **ce005_2 : Regular In-Kind Support**

Mean	Min	Max	OBS
700.0	700.0	700.0	1

- **ce005_2_every : Time Interval**

	No	%
2 Per season	1	50.00
	1	50.00
Total	2	100.00

- **ce005_3 : Non-Regular Monetary Support**

Mean	Min	Max	OBS
2,124.1	0.0	30,000.0	75

- **ce005_4 : Non-Regular In-Kind Support**

Mean	Min	Max	OBS
384.5	0.0	2,000.0	29

- **ce007 : Any Transfer from Non-Coresident Children**

	No	%
1 Yes	3,502	46.58
2 No	4,016	53.42
Total	7,518	100.00

- **ce008s1 : Which Child(ren)**

	No	%
1 FLSepChild[1]	1,739	100.00
Total	1,739	100.00

- **ce008s2 : Which Child(ren)**

	No	%
2 FLSepChild[2]	2,640	100.00
Total	2,640	100.00

- **ce008s3 : Which Child(ren)**

	No	%
3 FLSepChild[3]	1,978	100.00
Total	1,978	100.00

- **ce008s4 : Which Child(ren)**

	No	%
4 FLSepChild[4]	1,195	100.00
Total	1,195	100.00

- **ce008s5 : Which Child(ren)**

	No	%
5 FLSepChild[5]	614	100.00
Total	614	100.00

- **ce008s6 : Which Child(ren)**

	No	%
6 FLSepChild[6]	248	100.00
Total	248	100.00

- **ce008s7 : Which Child(ren)**

	No	%
7 FLSepChild[7]	101	100.00
Total	101	100.00

- **ce008s8 : Which Child(ren)**

	No	%
8 FLSepChild[8]	27	100.00
Total	27	100.00

- **ce008s9 : Which Child(ren)**

	No	%
9 FLSepChild[9]	10	100.00
Total	10	100.00

- **ce008s10 : Which Child(ren)**

	No	%
10 FLSepChild[10]	2	100.00

Total	2	100.00

- **ce008s11 : Which Child(ren)**

No Observations

- **ce008s12 : Which Child(ren)**

No Observations

- **ce008s13 : Which Child(ren)**

No Observations

- **ce008s14 : Which Child(ren)**

No Observations

- **ce008s15 : Which Child(ren)**

No Observations

- **ce008s16 : Which Child(ren)**

No Observations

- **ce008s17 : Which Child(ren)**

No Observations

- **ce008s18 : Which Child(ren)**

No Observations

- **ce008s19 : Which Child(ren)**

No Observations

- **ce008s20 : Which Child(ren)**

No Observations

- **ce008s21 : Which Child(ren)**

No Observations

- **ce008s22 : Which Child(ren)**

No Observations

- **ce008s23 : Which Child(ren)**

No Observations

- **ce008s24 : Which Child(ren)**

No Observations

- **ce008s25 : Which Child(ren)**

No Observations

- **ce009_1_1 : Regular Monetary Support**

Mean	Min	Max	OBS
1,604.5	0.0	39,000.0	304

- **ce009_1_1_a : Min Bracket**

Mean	Min	Max	OBS
801.0	801.0	801.0	1

- **ce009_1_1_b : Max Bracket**

Mean	Min	Max	OBS
1,599.0	1,599.0	1,599.0	1

- **ce009_1_1_every : Time Interval**

	No	%
	1	0.33
1 Per month	76	24.84
2 Per season	11	3.59
3 Per half year	15	4.90
4 Per year	203	66.34
Total	306	100.00

- **ce009_1_2 : Regular In-Kind Support**

Mean	Min	Max	OBS
854.8	0.0	10,000.0	63

- **ce009_1_2_a : Min Bracket**

No Observations

- **ce009_1_2_b : Max Bracket**

No Observations

- **ce009_1_2_every : Time Interval**

	No	%
	1	1.61
1 Per month	8	12.90
2 Per season	4	6.45
3 Per half year	7	11.29
4 Per year	42	67.74
Total	62	100.00

- **ce009_1_3 : Non-Regular Monetary Support**

Mean	Min	Max	OBS
2,061.1	-9,999.0	50,000.0	1,139

- **ce009_1_3_a : Min Bracket**

No Observations

- **ce009_1_3_b : Max Bracket**

No Observations

- **ce009_1_4 : Non-Regular In-Kind Support**

Mean	Min	Max	OBS
777.4	0.0	8,800.0	474

- **ce009_1_4_a : Min Bracket**

No Observations

- **ce009_1_4_b : Max Bracket**

No Observations

- **ce009_2_1 : Regular Monetary Support**

Mean	Min	Max	OBS
1,411.6	0.0	40,000.0	421

- **ce009_2_1_a : Min Bracket**

No Observations

- **ce009_2_1_b : Max Bracket**

No Observations

- **ce009_2_1_every : Time Interval**

	No	%
1 Per month	82	19.48
2 Per season	20	4.75
3 Per half year	21	4.99
4 Per year	297	70.55
	1	0.24
Total	421	100.00

- **ce009_2_2 : Regular In-Kind Support**

Mean	Min	Max	OBS
702.6	0.0	20,000.0	82

- **ce009_2_2_a : Min Bracket**

Mean	Min	Max	OBS
100.7	0.0	201.0	3

- **ce009_2_2_b : Max Bracket**

Mean	Min	Max	OBS
33,333,531.3	199.0	99,999,996.0	3

- **ce009_2_2_every : Time Interval**

	No	%
	1	1.19
1 Per month	8	9.52
2 Per season	6	7.14
3 Per half year	6	7.14
4 Per year	63	75.00
Total	84	100.00

- **ce009_2_3 : Non-Regular Monetary Support**

Mean	Min	Max	OBS
1,621.7	-9,999.0	150,000.0	1,736

- **ce009_2_3_a : Min Bracket**

No Observations

- **ce009_2_3_b : Max Bracket**

No Observations

- **ce009_2_4 : Non-Regular In-Kind Support**

Mean	Min	Max	OBS
676.8	0.0	45,000.0	811

- **ce009_2_4_a : Min Bracket**

No Observations

- **ce009_2_4_b : Max Bracket**

No Observations

- **ce009_3_1 : Regular Monetary Support**

Mean	Min	Max	OBS
1,120.7	0.0	40,000.0	314

- **ce009_3_1_a : Min Bracket**

No Observations

- **ce009_3_1_b : Max Bracket**

No Observations

- **ce009_3_1_every : Time Interval**

	No	%
1 Per month	60	19.17
2 Per season	13	4.15
3 Per half year	19	6.07
4 Per year	221	70.61
Total	313	100.00

- **ce009_3_2 : Regular In-Kind Support**

Mean	Min	Max	OBS
495.1	0.0	4,000.0	53

- **ce009_3_2_a : Min Bracket**

No Observations

- **ce009_3_2_b : Max Bracket**

No Observations

- **ce009_3_2_every : Time Interval**

	No	%
1 Per month	3	5.66
2 Per season	4	7.55
3 Per half year	5	9.43
4 Per year	40	75.47
	1	1.89
Total	53	100.00

- **ce009_3_3 : Non-Regular Monetary Support**

Mean	Min	Max	OBS
1,275.9	0.0	100,000.0	1,298

- **ce009_3_3_a : Min Bracket**

Mean	Min	Max	OBS
600.3	0.0	1,601.0	29

- **ce009_3_3_b : Max Bracket**

Mean	Min	Max	OBS
55,172,711.5	99.0	99,999,996.0	29

- **ce009_3_4 : Non-Regular In-Kind Support**

Mean	Min	Max	OBS
573.5	0.0	10,000.0	617

- **ce009_3_4_a : Min Bracket**

Mean	Min	Max	OBS
600.3	0.0	1,601.0	6

- **ce009_3_4_b : Max Bracket**

Mean	Min	Max	OBS
83,333,396.7	400.0	99,999,996.0	6

- **ce009_4_1 : Regular Monetary Support**

Mean	Min	Max	OBS
773.7	10.0	6,000.0	186

- **ce009_4_1_a : Min Bracket**

No Observations

- **ce009_4_1_b : Max Bracket**

No Observations

- **ce009_4_1_every : Time Interval**

	No	%
1 Per month	31	16.76
2 Per season	7	3.78
3 Per half year	10	5.41
4 Per year	137	74.05
Total	185	100.00

- **ce009_4_2 : Regular In-Kind Support**

Mean	Min	Max	OBS
665.4	0.0	4,000.0	37

- **ce009_4_2_a : Min Bracket**

No Observations

- **ce009_4_2_b : Max Bracket**

No Observations

- **ce009_4_2_every : Time Interval**

	No	%
1 Per month	3	7.89
2 Per season	8	21.05
3 Per half year	3	7.89
4 Per year	23	60.53
	1	2.63
Total	38	100.00

- **ce009_4_3 : Non-Regular Monetary Support**

Mean	Min	Max	OBS
1,081.5	-9,999.0	100,000.0	785

- **ce009_4_3_a : Min Bracket**

No Observations

- **ce009_4_3_b : Max Bracket**

No Observations

- **ce009_4_4 : Non-Regular In-Kind Support**

Mean	Min	Max	OBS
432.0	0.0	8,000.0	369

- **ce009_4_4_a : Min Bracket**

Mean	Min	Max	OBS
337.8	0.0	1,601.0	16

- **ce009_4_4_b : Max Bracket**

Mean	Min	Max	OBS
68,750,159.7	200.0	99,999,996.0	16

- **ce009_5_1 : Regular Monetary Support**

Mean	Min	Max	OBS
1,023.2	30.0	10,500.0	101

- **ce009_5_1_a : Min Bracket**

No Observations

- **ce009_5_1_b : Max Bracket**

No Observations

- **ce009_5_1_every : Time Interval**

	No	%
1 Per month	20	19.80
2 Per season	2	1.98
3 Per half year	6	5.94
4 Per year	72	71.29
	1	0.99
Total	101	100.00

- **ce009_5_2 : Regular In-Kind Support**

Mean	Min	Max	OBS
213.1	30.0	600.0	16

- **ce009_5_2_a : Min Bracket**

No Observations

- **ce009_5_2_b : Max Bracket**

No Observations

- **ce009_5_2_every : Time Interval**

	No	%
1 Per month	2	11.76
2 Per season	1	5.88
3 Per half year	1	5.88
4 Per year	12	70.59
	1	5.88
Total	17	100.00

- **ce009_5_3 : Non-Regular Monetary Support**

Mean	Min	Max	OBS
785.9	-9,999.0	15,000.0	399

- **ce009_5_3_a : Min Bracket**

No Observations

- **ce009_5_3_b : Max Bracket**

No Observations

- **ce009_5_4 : Non-Regular In-Kind Support**

Mean	Min	Max	OBS
373.0	0.0	6,000.0	196

- **ce009_5_4_a : Min Bracket**

No Observations

- **ce009_5_4_b : Max Bracket**

No Observations

- **ce009_6_1 : Regular Monetary Support**

Mean	Min	Max	OBS
528.1	0.0	3,600.0	36

- **ce009_6_1_a : Min Bracket**

No Observations

- **ce009_6_1_b : Max Bracket**

No Observations

- **ce009_6_1_every : Time Interval**

	No	%
1 Per month	5	13.89
2 Per season	1	2.78
3 Per half year	1	2.78
4 Per year	29	80.56
Total	36	100.00

- **ce009_6_2 : Regular In-Kind Support**

Mean	Min	Max	OBS
266.7	100.0	500.0	6

- **ce009_6_2_a : Min Bracket**

No Observations

- **ce009_6_2_b : Max Bracket**

No Observations

- **ce009_6_2_every : Time Interval**

	No	%
1 Per month	1	16.67
4 Per year	5	83.33
Total	6	100.00

- **ce009_6_3 : Non-Regular Monetary Support**

Mean	Min	Max	OBS
589.4	0.0	15,000.0	162

- **ce009_6_3_a : Min Bracket**

No Observations

- **ce009_6_3_b : Max Bracket**

No Observations

- **ce009_6_4 : Non-Regular In-Kind Support**

Mean	Min	Max	OBS
396.2	10.0	5,000.0	73

- **ce009_6_4_a : Min Bracket**

No Observations

- **ce009_6_4_b : Max Bracket**

No Observations

- **ce009_7_1 : Regular Monetary Support**

Mean	Min	Max	OBS
546.4	100.0	2,000.0	14

- **ce009_7_1_a : Min Bracket**

No Observations

- **ce009_7_1_b : Max Bracket**

No Observations

- **ce009_7_1_every : Time Interval**

	No	%
1 Per month	2	13.33
2 Per season	1	6.67
3 Per half year	1	6.67
4 Per year	10	66.67
	1	6.67
Total	15	100.00

- **ce009_7_2 : Regular In-Kind Support**

Mean	Min	Max	OBS
680.0	200.0	1,800.0	5

- **ce009_7_2_a : Min Bracket**

No Observations

- **ce009_7_2_b : Max Bracket**

No Observations

- **ce009_7_2_every : Time Interval**

	No	%
	1	16.67
1 Per month	1	16.67
2 Per season	2	33.33
4 Per year	2	33.33
Total	6	100.00

- **ce009_7_3 : Non-Regular Monetary Support**

Mean	Min	Max	OBS
836.2	0.0	10,000.0	61

- **ce009_7_3_a : Min Bracket**

No Observations

- **ce009_7_3_b : Max Bracket**

No Observations

- **ce009_7_4 : Non-Regular In-Kind Support**

Mean	Min	Max	OBS
298.6	50.0	1,000.0	35

- **ce009_7_4_a : Min Bracket**

No Observations

- **ce009_7_4_b : Max Bracket**

No Observations

- **ce009_8_1 : Regular Monetary Support**

Mean	Min	Max	OBS
483.3	150.0	800.0	3

- **ce009_8_1_a : Min Bracket**

No Observations

- **ce009_8_1_b : Max Bracket**

No Observations

- **ce009_8_1_every : Time Interval**

	No	%
	1	25.00
4 Per year	3	75.00
Total	4	100.00

- **ce009_8_2 : Regular In-Kind Support**

Mean	Min	Max	OBS
1,800.0	1,800.0	1,800.0	1

- **ce009_8_2_a : Min Bracket**

No Observations

- **ce009_8_2_b : Max Bracket**

No Observations

- **ce009_8_2_every : Time Interval**

	No	%
	1	50.00
2 Per season	1	50.00
Total	2	100.00

- **ce009_8_3 : Non-Regular Monetary Support**

Mean	Min	Max	OBS
500.0	0.0	2,000.0	13

- **ce009_8_3_a : Min Bracket**

No Observations

- **ce009_8_3_b : Max Bracket**

No Observations

- **ce009_8_4 : Non-Regular In-Kind Support**

Mean	Min	Max	OBS
256.3	30.0	600.0	12

- **ce009_8_4_a : Min Bracket**

No Observations

- **ce009_8_4_b : Max Bracket**

No Observations

- **ce009_9_1 : Regular Monetary Support**

Mean	Min	Max	OBS

150.0	150.0	150.0	1

- **ce009_9_1_a : Min Bracket**

No Observations

- **ce009_9_1_b : Max Bracket**

No Observations

- **ce009_9_1_every : Time Interval**

	No	%
4 Per year	1	50.00
	1	50.00
Total	2	100.00

- **ce009_9_2 : Regular In-Kind Support**

No Observations

- **ce009_9_2_a : Min Bracket**

No Observations

- **ce009_9_2_b : Max Bracket**

No Observations

- **ce009_9_2_every : Time Interval**

No Observations

- **ce009_9_3 : Non-Regular Monetary Support**

Mean	Min	Max	OBS
341.7	50.0	1,000.0	6

- **ce009_9_3_a : Min Bracket**

No Observations

- **ce009_9_3_b : Max Bracket**

No Observations

- **ce009_9_4 : Non-Regular In-Kind Support**

Mean	Min	Max	OBS
200.0	100.0	400.0	4

- **ce009_9_4_a : Min Bracket**

No Observations

- **ce009_9_4_b : Max Bracket**

No Observations

- **ce009_10_1 : Regular Monetary Support**

No Observations

- **ce009_10_1_a : Min Bracket**

No Observations

- **ce009_10_1_b : Max Bracket**

No Observations

- **ce009_10_1_every : Time Interval**

No Observations

- **ce009_10_2 : Regular In-Kind Support**

No Observations

- **ce009_10_2_a : Min Bracket**

No Observations

- **ce009_10_2_b : Max Bracket**

No Observations

- **ce009_10_2_every : Time Interval**

No Observations

- **ce009_10_3 : Non-Regular Monetary Support**

Mean	Min	Max	OBS
1,250.0	500.0	2,000.0	2

- **ce009_10_3_a : Min Bracket**

No Observations

- **ce009_10_3_b : Max Bracket**

No Observations

- **ce009_10_4 : Non-Regular In-Kind Support**

Mean	Min	Max	OBS
500.0	500.0	500.0	1

- **ce009_10_4_a : Min Bracket**

No Observations

- **ce009_10_4_b : Max Bracket**

No Observations

- **ce011 : Any Transfer from Non-Coresident Grandchildren**

	No	%
1 Yes	419	6.27
2 No	6,265	93.73
Total	6,684	100.00

- **ce012s1 : Parent of the Grandchildren**

	No	%
1 HMemberChildren[1]	262	100.00
Total	262	100.00

- **ce012s2 : Parent of the Grandchildren**

	No	%
2 HMemberChildren[2]	202	100.00
Total	202	100.00

- **ce012s3 : Parent of the Grandchildren**

	No	%
3 HMemberChildren[3]	141	100.00
Total	141	100.00

- **ce012s4 : Parent of the Grandchildren**

	No	%
4 HMemberChildren[4]	79	100.00
Total	79	100.00

- **ce012s5 : Parent of the Grandchildren**

	No	%
5 HMemberChildren[5]	36	100.00
Total	36	100.00

- **ce012s6 : Parent of the Grandchildren**

	No	%
6 HMemberChildren[6]	20	100.00
Total	20	100.00

- **ce012s7 : Parent of the Grandchildren**

	No	%
7 HMemberChildren[7]	7	100.00
Total	7	100.00

- **ce012s8 : Parent of the Grandchildren**

No Observations

- **ce012s9 : Parent of the Grandchildren**

No Observations

- **ce012s10 : Parent of the Grandchildren**

	No	%
10 HMemberChildren[10]	1	100.00
Total	1	100.00

- **ce012s11 : Parent of the Grandchildren**

No Observations

- **ce012s12 : Parent of the Grandchildren**

No Observations

- **ce012s13 : Parent of the Grandchildren**

No Observations

- **ce012s14 : Parent of the Grandchildren**

No Observations

- **ce012s15 : Parent of the Grandchildren**

No Observations

- **ce012s16 : Parent of the Grandchildren**

No Observations

- **ce012s17 : Parent of the Grandchildren**

No Observations

- **ce012s18 : Parent of the Grandchildren**

No Observations

- **ce012s19 : Parent of the Grandchildren**

No Observations

- **ce012s20 : Parent of the Grandchildren**

No Observations

- **ce012s21 : Parent of the Grandchildren**

No Observations

- **ce012s22 : Parent of the Grandchildren**

No Observations

- **ce012s23 : Parent of the Grandchildren**

No Observations

- **ce012s24 : Parent of the Grandchildren**

No Observations

- **ce012s25 : Parent of the Grandchildren**

No Observations

- **ce013_1_1 : Regular Monetary Support**

Mean	Min	Max	OBS
405.0	50.0	2,000.0	20

- **ce013_1_1_a : Min Bracket**

No Observations

- **ce013_1_1_b : Max Bracket**

No Observations

- **ce013_1_1_every : Time Interval**

	No	%
	1	4.76
1 Per month	5	23.81
4 Per year	15	71.43
Total	21	100.00

- **ce013_1_2 : Regular In-Kind Support**

Mean	Min	Max	OBS
125.0	50.0	200.0	2

- **ce013_1_2_a : Min Bracket**

No Observations

- **ce013_1_2_b : Max Bracket**

No Observations

- **ce013_1_2_every : Time Interval**

	No	%
4 Per year	2	66.67
	1	33.33
Total	3	100.00

- **ce013_1_3 : Non-Regular Monetary Support**

Mean	Min	Max	OBS
545.5	0.0	8,000.0	157

- **ce013_1_3_a : Min Bracket**

No Observations

- **ce013_1_3_b : Max Bracket**

No Observations

- **ce013_1_4 : Non-Regular In-Kind Support**

Mean	Min	Max	OBS
465.5	0.0	5,000.0	93

- **ce013_1_4_a : Min Bracket**

No Observations

- **ce013_1_4_b : Max Bracket**

No Observations

- **ce013_2_1 : Regular Monetary Support**

Mean	Min	Max	OBS
401.1	50.0	2,000.0	19

- **ce013_2_1_a : Min Bracket**

No Observations

- **ce013_2_1_b : Max Bracket**

No Observations

- **ce013_2_1_every : Time Interval**

	No	%
1 Per month	2	10.00
3 Per half year	2	10.00
4 Per year	15	75.00
	1	5.00
Total	20	100.00

- **ce013_2_2 : Regular In-Kind Support**

	No	%
100	1	100.00
Total	1	100.00

- **ce013_2_2_a : Min Bracket**

No Observations

- **ce013_2_2_b : Max Bracket**

No Observations

- **ce013_2_2_every : Time Interval**

	No	%
4 Per year	1	50.00
	1	50.00
Total	2	100.00

- **ce013_2_3 : Non-Regular Monetary Support**

Mean	Min	Max	OBS
458.0	0.0	10,000.0	126

- **ce013_2_3_a : Min Bracket**

No Observations

- **ce013_2_3_b : Max Bracket**

No Observations

- **ce013_2_4 : Non-Regular In-Kind Support**

Mean	Min	Max	OBS
208.6	0.0	1,500.0	67

- **ce013_2_4_a : Min Bracket**

No Observations

- **ce013_2_4_b : Max Bracket**

No Observations

- **ce013_3_1 : Regular Monetary Support**

Mean	Min	Max	OBS
331.4	100.0	1,000.0	7

- **ce013_3_1_a : Min Bracket**

No Observations

- **ce013_3_1_b : Max Bracket**

No Observations

- **ce013_3_1_every : Time Interval**

	No	%
4 Per year	7	87.50
	1	12.50
Total	8	100.00

- **ce013_3_2 : Regular In-Kind Support**

	No	%
100	1	100.00
Total	1	100.00

- **ce013_3_2_a : Min Bracket**

No Observations

- **ce013_3_2_b : Max Bracket**

No Observations

- **ce013_3_2_every : Time Interval**

	No	%
	1	50.00
4 Per year	1	50.00
Total	2	100.00

- **ce013_3_3 : Non-Regular Monetary Support**

Mean	Min	Max	OBS
348.8	10.0	3,000.0	100

- **ce013_3_3_a : Min Bracket**

Mean	Min	Max	OBS
800.0	0.0	1,600.0	2

- **ce013_3_3_b : Max Bracket**

Mean	Min	Max	OBS
50,000,798.0	1,600.0	99,999,996.0	2

- **ce013_3_4 : Non-Regular In-Kind Support**

Mean	Min	Max	OBS
161.3	5.0	400.0	44

- **ce013_3_4_a : Min Bracket**

Mean	Min	Max	OBS
1,600.0	1,600.0	1,600.0	1

- **ce013_3_4_b : Max Bracket**

Mean	Min	Max	OBS
1,600.0	1,600.0	1,600.0	1

- **ce013_4_1 : Regular Monetary Support**

Mean	Min	Max	OBS
275.0	100.0	500.0	4

- **ce013_4_1_a : Min Bracket**

No Observations

- **ce013_4_1_b : Max Bracket**

No Observations

- **ce013_4_1_every : Time Interval**

	No	%
4 Per year	4	100.00
Total	4	100.00

- **ce013_4_2 : Regular In-Kind Support**

	No	%
100	1	100.00
Total	1	100.00

- **ce013_4_2_a : Min Bracket**

No Observations

- **ce013_4_2_b : Max Bracket**

No Observations

- **ce013_4_2_every : Time Interval**

	No	%
4 Per year	1	50.00
	1	50.00
Total	2	100.00

- **ce013_4_3 : Non-Regular Monetary Support**

Mean	Min	Max	OBS
263.2	0.0	1,000.0	53

- **ce013_4_3_a : Min Bracket**

No Observations

- **ce013_4_3_b : Max Bracket**

No Observations

- **ce013_4_4 : Non-Regular In-Kind Support**

Mean	Min	Max	OBS
143.9	0.0	500.0	23

- **ce013_4_4_a : Min Bracket**

	No	%
0	1	100.00
Total	1	100.00

- **ce013_4_4_b : Max Bracket**

Mean	Min	Max	OBS
99,999,996.0	99,999,996.0	99,999,996.0	1

- **ce013_5_1 : Regular Monetary Support**

	No	%
0	1	100.00
Total	1	100.00

- **ce013_5_1_a : Min Bracket**

No Observations

- **ce013_5_1_b : Max Bracket**

No Observations

- **ce013_5_1_every : Time Interval**

	No	%
	1	50.00
3 Per half year	1	50.00
Total	2	100.00

- **ce013_5_2 : Regular In-Kind Support**

No Observations

- **ce013_5_2_a : Min Bracket**

No Observations

- **ce013_5_2_b : Max Bracket**

No Observations

- **ce013_5_2_every : Time Interval**

No Observations

- **ce013_5_3 : Non-Regular Monetary Support**

Mean	Min	Max	OBS
407.7	50.0	3,000.0	26

- **ce013_5_3_a : Min Bracket**

No Observations

- **ce013_5_3_b : Max Bracket**

No Observations

- **ce013_5_4 : Non-Regular In-Kind Support**

Mean	Min	Max	OBS
198.2	0.0	500.0	11

- **ce013_5_4_a : Min Bracket**

No Observations

- **ce013_5_4_b : Max Bracket**

No Observations

- **ce013_6_1 : Regular Monetary Support**

	No	%
100	3	100.00
Total	3	100.00

- **ce013_6_1_a : Min Bracket**

No Observations

- **ce013_6_1_b : Max Bracket**

No Observations

- **ce013_6_1_every : Time Interval**

	No	%
	1	25.00
4 Per year	3	75.00
Total	4	100.00

- **ce013_6_2 : Regular In-Kind Support**

No Observations

- **ce013_6_2_a : Min Bracket**

No Observations

- **ce013_6_2_b : Max Bracket**

No Observations

- **ce013_6_2_every : Time Interval**

No Observations

- **ce013_6_3 : Non-Regular Monetary Support**

Mean	Min	Max	OBS
375.0	50.0	1,500.0	14

- **ce013_6_3_a : Min Bracket**

No Observations

- **ce013_6_3_b : Max Bracket**

No Observations

- **ce013_6_4 : Non-Regular In-Kind Support**

Mean	Min	Max	OBS
325.0	100.0	500.0	4

- **ce013_6_4_a : Min Bracket**

No Observations

- **ce013_6_4_b : Max Bracket**

No Observations

- **ce013_7_1 : Regular Monetary Support**

No Observations

- **ce013_7_1_a : Min Bracket**

No Observations

- **ce013_7_1_b : Max Bracket**

No Observations

- **ce013_7_1_every : Time Interval**

No Observations

- **ce013_7_2 : Regular In-Kind Support**

No Observations

- **ce013_7_2_a : Min Bracket**

No Observations

- **ce013_7_2_b : Max Bracket**

No Observations

- **ce013_7_2_every : Time Interval**

No Observations

- **ce013_7_3 : Non-Regular Monetary Support**

Mean	Min	Max	OBS

214.0	20.0	600.0	5

- **ce013_7_3_a : Min Bracket**

No Observations

- **ce013_7_3_b : Max Bracket**

No Observations

- **ce013_7_4 : Non-Regular In-Kind Support**

	No	%
100	1	100.00
Total	1	100.00

- **ce013_7_4_a : Min Bracket**

No Observations

- **ce013_7_4_b : Max Bracket**

No Observations

- **ce013_8_1 : Regular Monetary Support**

No Observations

- **ce013_8_1_a : Min Bracket**

No Observations

- **ce013_8_1_b : Max Bracket**

No Observations

- **ce013_8_1_every : Time Interval**

No Observations

- **ce013_8_2 : Regular In-Kind Support**

No Observations

- **ce013_8_2_a : Min Bracket**

No Observations

- **ce013_8_2_b : Max Bracket**

No Observations

- **ce013_8_2_every : Time Interval**

No Observations

- **ce013_8_3 : Non-Regular Monetary Support**

No Observations

- **ce013_8_3_a : Min Bracket**

No Observations

- **ce013_8_3_b : Max Bracket**

No Observations

- **ce013_8_4 : Non-Regular In-Kind Support**

No Observations

- **ce013_8_4_a : Min Bracket**

No Observations

- **ce013_8_4_b : Max Bracket**

No Observations

- **ce013_9_1 : Regular Monetary Support**

No Observations

- **ce013_9_1_a : Min Bracket**

No Observations

- **ce013_9_1_b : Max Bracket**

No Observations

- **ce013_9_1_every : Time Interval**

No Observations

- **ce013_9_2 : Regular In-Kind Support**

No Observations

- **ce013_9_2_a : Min Bracket**

No Observations

- **ce013_9_2_b : Max Bracket**

No Observations

- **ce013_9_2_every : Time Interval**

No Observations

- **ce013_9_3 : Non-Regular Monetary Support**

No Observations

- **ce013_9_3_a : Min Bracket**

No Observations

- **ce013_9_3_b : Max Bracket**

No Observations

- **ce013_9_4 : Non-Regular In-Kind Support**

No Observations

- **ce013_9_4_a : Min Bracket**

No Observations

- **ce013_9_4_b : Max Bracket**

No Observations

- **ce013_10_1 : Regular Monetary Support**

No Observations

- **ce013_10_1_a : Min Bracket**

No Observations

- **ce013_10_1_b : Max Bracket**

No Observations

- **ce013_10_1_every : Time Interval**

No Observations

- **ce013_10_2 : Regular In-Kind Support**

No Observations

- **ce013_10_2_a : Min Bracket**

No Observations

- **ce013_10_2_b : Max Bracket**

No Observations

- **ce013_10_2_every : Time Interval**

No Observations

- **ce013_10_3 : Non-Regular Monetary Support**

Mean	Min	Max	OBS
500.0	500.0	500.0	1

- **ce013_10_3_a : Min Bracket**

No Observations

- **ce013_10_3_b : Max Bracket**

No Observations

- **ce013_10_4 : Non-Regular In-Kind Support**

Mean	Min	Max	OBS

300.0	300.0	300.0	1

- **ce013_10_4_a : Min Bracket**

No Observations

- **ce013_10_4_b : Max Bracket**

No Observations

- **ce015 : Any Transfer from Non-Coresident Other Relatives**

	No	%
1 Yes	638	6.32
2 No	9,452	93.68
Total	10,090	100.00

- **ce016_1 : Regular Monetary Support**

Mean	Min	Max	OBS
1,555.1	0.0	30,000.0	37

- **ce016_1_a : Min Bracket**

No Observations

- **ce016_1_b : Max Bracket**

No Observations

- **ce016_1_every : Time Interval**

	No	%
	1	2.63
1 Per month	7	18.42

3 Per half year	1	2.63
4 Per year	29	76.32
Total	38	100.00

- **ce016_2 : Regular In-Kind Support**

Mean	Min	Max	OBS
369.2	15.0	1,000.0	6

- **ce016_2_a : Min Bracket**

No Observations

- **ce016_2_b : Max Bracket**

No Observations

- **ce016_2_every : Time Interval**

	No	%
	1	14.29
1 Per month	2	28.57
2 Per season	1	14.29
4 Per year	3	42.86
Total	7	100.00

- **ce016_3 : Non-Regular Monetary Support**

Mean	Min	Max	OBS
5,480.1	0.0	700,000.0	504

- **ce016_3_a : Min Bracket**

Mean	Min	Max	OBS
801.0	801.0	801.0	1

- **ce016_3_b : Max Bracket**

Mean	Min	Max	OBS
1,599.0	1,599.0	1,599.0	1

- **ce016_4 : Non-Regular In-Kind Support**

Mean	Min	Max	OBS
887.4	0.0	40,000.0	147

- **ce016_4_a : Min Bracket**

No Observations

- **ce016_4_b : Max Bracket**

No Observations

- **ce018 : Any Transfer from Friends**

	No	%
1 Yes	102	1.01
2 No	9,989	98.99
Total	10,091	100.00

- **ce019_1 : Regular Monetary Support**

Mean	Min	Max	OBS
2,308.3	50.0	10,000.0	6

- **ce019_1_a : Min Bracket**

No Observations

- **ce019_1_b : Max Bracket**

No Observations

- **ce019_1_every : Time Interval**

	No	%
	1	14.29
1 Per month	1	14.29
4 Per year	5	71.43
Total	7	100.00

- **ce019_2 : Regular In-Kind Support**

	No	%
100	1	100.00
Total	1	100.00

- **ce019_2_a : Min Bracket**

No Observations

- **ce019_2_b : Max Bracket**

No Observations

- **ce019_2_every : Time Interval**

	No	%
	1	50.00
2 Per season	1	50.00
Total	2	100.00

- **ce019_3 : Non-Regular Monetary Support**

Mean	Min	Max	OBS
11,098.7	0.0	150,000.0	77

- **ce019_3_a : Min Bracket**

No Observations

- **ce019_3_b : Max Bracket**

No Observations

- **ce019_4 : Non-Regular In-Kind Support**

Mean	Min	Max	OBS
758.6	0.0	8,000.0	28

- **ce019_4_a : Min Bracket**

No Observations

- **ce019_4_b : Max Bracket**

No Observations

- **ce021 : Any Transfer to Non-Coresident Parents**

	No	%
1 Yes	1,537	44.42
2 No	1,923	55.58
Total	3,460	100.00

- **ce022_1 : Regular Monetary Support**

Mean	Min	Max	OBS
652.2	0.0	10,000.0	290

- **ce022_1_a : Min Bracket**

No Observations

- **ce022_1_b : Max Bracket**

No Observations

- **ce022_1_every : Time Interval**

	No	%
1 Per month	105	36.21
2 Per season	7	2.41
3 Per half year	13	4.48
4 Per year	165	56.90
Total	290	100.00

- **ce022_2 : Regular In-Kind Support**

Mean	Min	Max	OBS
488.6	0.0	3,000.0	65

- **ce022_2_a : Min Bracket**

No Observations

- **ce022_2_b : Max Bracket**

No Observations

- **ce022_2_every : Time Interval**

	No	%
	1	1.47
1 Per month	17	25.00
2 Per season	11	16.18
3 Per half year	7	10.29

4 Per year	32	47.06
Total	68	100.00

- **ce022_3 : Non-Regular Monetary Support**

Mean	Min	Max	OBS
1,047.5	-9,999.0	60,000.0	974

- **ce022_3_a : Min Bracket**

No Observations

- **ce022_3_b : Max Bracket**

No Observations

- **ce022_4 : Non-Regular In-Kind Support**

Mean	Min	Max	OBS
688.3	0.0	20,000.0	548

- **ce022_4_a : Min Bracket**

No Observations

- **ce022_4_b : Max Bracket**

No Observations

- **ce024 : Any Transfer to Non-Coresident Parents-in-Law**

	No	%
1 Yes	1,278	39.44
2 No	1,962	60.56
Total	3,240	100.00

- **ce025_1 : Regular Monetary Support**

Mean	Min	Max	OBS
708.1	10.0	4,800.0	226

- **ce025_1_a : Min Bracket**

No Observations

- **ce025_1_b : Max Bracket**

No Observations

- **ce025_1_every : Time Interval**

	No	%
	1	0.45
1 Per month	63	28.13
2 Per season	7	3.13
3 Per half year	11	4.91
4 Per year	142	63.39
Total	224	100.00

- **ce025_2 : Regular In-Kind Support**

Mean	Min	Max	OBS
943.3	0.0	20,000.0	50

- **ce025_2_a : Min Bracket**

No Observations

- **ce025_2_b : Max Bracket**

No Observations

- **ce025_2_every : Time Interval**

	No	%
1 Per month	14	29.17
2 Per season	6	12.50
3 Per half year	4	8.33
4 Per year	23	47.92
	1	2.08
Total	48	100.00

- **ce025_3 : Non-Regular Monetary Support**

Mean	Min	Max	OBS
1,188.2	0.0	200,000.0	805

- **ce025_3_a : Min Bracket**

Mean	Min	Max	OBS
400.0	400.0	400.0	1

- **ce025_3_b : Max Bracket**

Mean	Min	Max	OBS
400.0	400.0	400.0	1

- **ce025_4 : Non-Regular In-Kind Support**

Mean	Min	Max	OBS
604.2	0.0	10,000.0	442

- **ce025_4_a : Min Bracket**

Mean	Min	Max	OBS
134.0	0.0	201.0	3

- **ce025_4_b : Max Bracket**

Mean	Min	Max	OBS
33,333,731.3	399.0	99,999,996.0	3

- **ce027 : Any Transfer to Non-Coresident Children**

	No	%
1 Yes	663	8.82
2 No	6,851	91.18
Total	7,514	100.00

- **ce028s1 : Which Child(ren)**

	No	%
1 FLSepChild[1]	339	100.00
Total	339	100.00

- **ce028s2 : Which Child(ren)**

	No	%
2 FLSepChild[2]	432	100.00
Total	432	100.00

- **ce028s3 : Which Child(ren)**

	No	%
3 FLSepChild[3]	180	100.00
Total	180	100.00

- **ce028s4 : Which Child(ren)**

	No	%
4 FLSepChild[4]	68	100.00
Total	68	100.00

- **ce028s5 : Which Child(ren)**

	No	%
5 FLSepChild[5]	20	100.00
Total	20	100.00

- **ce028s6 : Which Child(ren)**

	No	%
6 FLSepChild[6]	6	100.00
Total	6	100.00

- **ce028s7 : Which Child(ren)**

	No	%
7 FLSepChild[7]	2	100.00
Total	2	100.00

- **ce028s8 : Which Child(ren)**

	No	%
8 FLSepChild[8]	1	100.00
Total	1	100.00

- **ce028s9 : Which Child(ren)**

No Observations

- **ce028s10 : Which Child(ren)**

No Observations

- **ce028s11 : Which Child(ren)**

No Observations

- **ce028s12 : Which Child(ren)**

No Observations

- **ce028s13 : Which Child(ren)**

No Observations

- **ce028s14 : Which Child(ren)**

No Observations

- **ce028s15 : Which Child(ren)**

No Observations

- **ce028s16 : Which Child(ren)**

No Observations

- **ce028s17 : Which Child(ren)**

No Observations

- **ce028s18 : Which Child(ren)**

No Observations

- **ce028s19 : Which Child(ren)**

No Observations

- **ce028s20 : Which Child(ren)**

No Observations

- **ce028s21 : Which Child(ren)**

No Observations

- **ce028s22 : Which Child(ren)**

No Observations

- **ce028s23 : Which Child(ren)**

No Observations

- **ce028s24 : Which Child(ren)**

No Observations

- **ce028s25 : Which Child(ren)**

No Observations

- **ce029_1_1 : Regular Monetary Support**

Mean	Min	Max	OBS
6,560.6	100.0	50,000.0	33

- **ce029_1_1_a : Min Bracket**

No Observations

- **ce029_1_1_b : Max Bracket**

No Observations

- **ce029_1_1_every : Time Interval**

	No	%
1 Per month	22	66.67
3 Per half year	1	3.03
4 Per year	10	30.30
Total	33	100.00

- **ce029_1_2 : Regular In-Kind Support**

Mean	Min	Max	OBS
2,500.0	2,500.0	2,500.0	1

- **ce029_1_2_a : Min Bracket**

No Observations

- **ce029_1_2_b : Max Bracket**

No Observations

- **ce029_1_2_every : Time Interval**

	No	%
1 Per month	1	50.00
	1	50.00
Total	2	100.00

- **ce029_1_3 : Non-Regular Monetary Support**

Mean	Min	Max	OBS
15,860.9	-9,999.0	240,000.0	227

- **ce029_1_3_a : Min Bracket**

No Observations

- **ce029_1_3_b : Max Bracket**

No Observations

- **ce029_1_4 : Non-Regular In-Kind Support**

Mean	Min	Max	OBS
6,593.0	0.0	130,000.0	43

- **ce029_1_4_a : Min Bracket**

No Observations

- **ce029_1_4_b : Max Bracket**

No Observations

- **ce029_2_1 : Regular Monetary Support**

Mean	Min	Max	OBS
5,125.1	5.0	20,000.0	40

- **ce029_2_1_a : Min Bracket**

No Observations

- **ce029_2_1_b : Max Bracket**

No Observations

- **ce029_2_1_every : Time Interval**

	No	%
	1	2.44
1 Per month	25	60.98
2 Per season	2	4.88
3 Per half year	1	2.44
4 Per year	12	29.27
Total	41	100.00

- **ce029_2_2 : Regular In-Kind Support**

Mean	Min	Max	OBS
808.3	100.0	3,500.0	6

- **ce029_2_2_a : Min Bracket**

No Observations

- **ce029_2_2_b : Max Bracket**

No Observations

- **ce029_2_2_every : Time Interval**

	No	%
	1	14.29
1 Per month	3	42.86
4 Per year	3	42.86
Total	7	100.00

- **ce029_2_3 : Non-Regular Monetary Support**

Mean	Min	Max	OBS
7,710.8	0.0	350,000.0	288

- **ce029_2_3_a : Min Bracket**

No Observations

- **ce029_2_3_b : Max Bracket**

No Observations

- **ce029_2_4 : Non-Regular In-Kind Support**

Mean	Min	Max	OBS
1,321.8	0.0	20,000.0	49

- **ce029_2_4_a : Min Bracket**

No Observations

- **ce029_2_4_b : Max Bracket**

No Observations

- **ce029_3_1 : Regular Monetary Support**

Mean	Min	Max	OBS
4,213.9	5.0	16,000.0	8

- **ce029_3_1_a : Min Bracket**

No Observations

- **ce029_3_1_b : Max Bracket**

No Observations

- **ce029_3_1_every : Time Interval**

	No	%
	1	12.50
1 Per month	3	37.50
4 Per year	4	50.00
Total	8	100.00

- **ce029_3_2 : Regular In-Kind Support**

	No	%
100	1	100.00
Total	1	100.00

- **ce029_3_2_a : Min Bracket**

No Observations

- **ce029_3_2_b : Max Bracket**

No Observations

- **ce029_3_2_every : Time Interval**

	No	%
	1	50.00
4 Per year	1	50.00
Total	2	100.00

- **ce029_3_3 : Non-Regular Monetary Support**

Mean	Min	Max	OBS
6,550.4	0.0	70,000.0	110

- **ce029_3_3_a : Min Bracket**

Mean	Min	Max	OBS
1,120.6	0.0	1,601.0	5

- **ce029_3_3_b : Max Bracket**

Mean	Min	Max	OBS
60,000,637.4	1,599.0	99,999,996.0	5

- **ce029_3_4 : Non-Regular In-Kind Support**

Mean	Min	Max	OBS
816.0	30.0	8,000.0	30

- **ce029_3_4_a : Min Bracket**

	No	%
0	1	100.00
Total	1	100.00

- **ce029_3_4_b : Max Bracket**

Mean	Min	Max	OBS
99,999,996.0	99,999,996.0	99,999,996.0	1

- **ce029_4_1 : Regular Monetary Support**

Mean	Min	Max	OBS
7,729.3	5.0	20,000.0	7

- **ce029_4_1_a : Min Bracket**

No Observations

- **ce029_4_1_b : Max Bracket**

No Observations

- **ce029_4_1_every : Time Interval**

	No	%

	1	12.50
1 Per month	4	50.00
2 Per season	1	12.50
4 Per year	2	25.00
Total	8	100.00

- **ce029_4_2 : Regular In-Kind Support**

Mean	Min	Max	OBS
1,050.0	100.0	2,000.0	2

- **ce029_4_2_a : Min Bracket**

No Observations

- **ce029_4_2_b : Max Bracket**

No Observations

- **ce029_4_2_every : Time Interval**

	No	%
1 Per month	1	33.33
4 Per year	1	33.33
	1	33.33
Total	3	100.00

- **ce029_4_3 : Non-Regular Monetary Support**

Mean	Min	Max	OBS
7,596.9	100.0	100,000.0	36

- **ce029_4_3_a : Min Bracket**

No Observations

- **ce029_4_3_b : Max Bracket**

No Observations

- **ce029_4_4 : Non-Regular In-Kind Support**

Mean	Min	Max	OBS
322.2	100.0	1,000.0	9

- **ce029_4_4_a : Min Bracket**

	No	%
0	1	100.00
Total	1	100.00

- **ce029_4_4_b : Max Bracket**

Mean	Min	Max	OBS
99,999,996.0	99,999,996.0	99,999,996.0	1

- **ce029_5_1 : Regular Monetary Support**

Mean	Min	Max	OBS
10,235.0	5.0	30,000.0	3

- **ce029_5_1_a : Min Bracket**

No Observations

- **ce029_5_1_b : Max Bracket**

No Observations

- **ce029_5_1_every : Time Interval**

	No	%

	1	25.00
1 Per month	1	25.00
4 Per year	2	50.00
Total	4	100.00

- **ce029_5_2 : Regular In-Kind Support**

No Observations

- **ce029_5_2_a : Min Bracket**

No Observations

- **ce029_5_2_b : Max Bracket**

No Observations

- **ce029_5_2_every : Time Interval**

No Observations

- **ce029_5_3 : Non-Regular Monetary Support**

Mean	Min	Max	OBS
2,457.8	100.0	10,000.0	9

- **ce029_5_3_a : Min Bracket**

No Observations

- **ce029_5_3_b : Max Bracket**

No Observations

- **ce029_5_4 : Non-Regular In-Kind Support**

Mean	Min	Max	OBS
166.7	100.0	300.0	3

- **ce029_5_4_a : Min Bracket**

No Observations

- **ce029_5_4_b : Max Bracket**

No Observations

- **ce029_6_1 : Regular Monetary Support**

No Observations

- **ce029_6_1_a : Min Bracket**

No Observations

- **ce029_6_1_b : Max Bracket**

No Observations

- **ce029_6_1_every : Time Interval**

No Observations

- **ce029_6_2 : Regular In-Kind Support**

No Observations

- **ce029_6_2_a : Min Bracket**

No Observations

- **ce029_6_2_b : Max Bracket**

No Observations

- **ce029_6_2_every : Time Interval**

No Observations

- **ce029_6_3 : Non-Regular Monetary Support**

Mean	Min	Max	OBS
444.0	100.0	1,200.0	5

- **ce029_6_3_a : Min Bracket**

No Observations

- **ce029_6_3_b : Max Bracket**

No Observations

- **ce029_6_4 : Non-Regular In-Kind Support**

	No	%

100	1	100.00
Total	1	100.00

- **ce029_6_4_a : Min Bracket**

No Observations

- **ce029_6_4_b : Max Bracket**

No Observations

- **ce029_7_1 : Regular Monetary Support**

No Observations

- **ce029_7_1_a : Min Bracket**

No Observations

- **ce029_7_1_b : Max Bracket**

No Observations

- **ce029_7_1_every : Time Interval**

No Observations

- **ce029_7_2 : Regular In-Kind Support**

No Observations

- **ce029_7_2_a : Min Bracket**

No Observations

- **ce029_7_2_b : Max Bracket**

No Observations

- **ce029_7_2_every : Time Interval**

No Observations

- **ce029_7_3 : Non-Regular Monetary Support**

Mean	Min	Max	OBS
110.0	100.0	120.0	2

- **ce029_7_3_a : Min Bracket**

No Observations

- **ce029_7_3_b : Max Bracket**

No Observations

- **ce029_7_4 : Non-Regular In-Kind Support**

	No	%
100	1	100.00
Total	1	100.00

- **ce029_7_4_a : Min Bracket**

No Observations

- **ce029_7_4_b : Max Bracket**

No Observations

- **ce029_8_1 : Regular Monetary Support**

No Observations

- **ce029_8_1_a : Min Bracket**

No Observations

- **ce029_8_1_b : Max Bracket**

No Observations

- **ce029_8_1_every : Time Interval**

No Observations

- **ce029_8_2 : Regular In-Kind Support**

No Observations

- **ce029_8_2_a : Min Bracket**

No Observations

- **ce029_8_2_b : Max Bracket**

No Observations

- **ce029_8_2_every : Time Interval**

No Observations

- **ce029_8_3 : Non-Regular Monetary Support**

No Observations

- **ce029_8_3_a : Min Bracket**

No Observations

- **ce029_8_3_b : Max Bracket**

No Observations

- **ce029_8_4 : Non-Regular In-Kind Support**

Mean	Min	Max	OBS
300.0	300.0	300.0	1

- **ce029_8_4_a : Min Bracket**

No Observations

- **ce029_8_4_b : Max Bracket**

No Observations

- **ce029_9_1 : Regular Monetary Support**

No Observations

- **ce029_9_1_a : Min Bracket**

No Observations

- **ce029_9_1_b : Max Bracket**

No Observations

- **ce029_9_1_every : Time Interval**

No Observations

- **ce029_9_2 : Regular In-Kind Support**

No Observations

- **ce029_9_2_a : Min Bracket**

No Observations

- **ce029_9_2_b : Max Bracket**

No Observations

- **ce029_9_2_every : Time Interval**

No Observations

- **ce029_9_3 : Non-Regular Monetary Support**

No Observations

- **ce029_9_3_a : Min Bracket**

No Observations

- **ce029_9_3_b : Max Bracket**

No Observations

- **ce029_9_4 : Non-Regular In-Kind Support**

No Observations

- **ce029_9_4_a : Min Bracket**

No Observations

- **ce029_9_4_b : Max Bracket**

No Observations

- **ce029_10_1 : Regular Monetary Support**

No Observations

- **ce029_10_1_a : Min Bracket**

No Observations

- **ce029_10_1_b : Max Bracket**

No Observations

- **ce029_10_1_every : Time Interval**

No Observations

- **ce029_10_2 : Regular In-Kind Support**

No Observations

- **ce029_10_2_a : Min Bracket**

No Observations

- **ce029_10_2_b : Max Bracket**

No Observations

- **ce029_10_2_every : Time Interval**

No Observations

- **ce029_10_3 : Non-Regular Monetary Support**

No Observations

- **ce029_10_3_a : Min Bracket**

No Observations

- **ce029_10_3_b : Max Bracket**

No Observations

- **ce029_10_4 : Non-Regular In-Kind Support**

No Observations

- **ce029_10_4_a : Min Bracket**

No Observations

- **ce029_10_4_b : Max Bracket**

No Observations

- **ce031 : Any Transfer to Non-coresident Grandchildren**

	No	%
1 Yes	1,154	17.26
2 No	5,531	82.74
Total	6,685	100.00

- **ce032s1 : Parents of the Grandchildren**

	No	%

1 HMemberChildren[1]	790	100.00
Total	790	100.00

- **ce032s2 : Parents of the Grandchildren**

	No	%
2 HMemberChildren[2]	693	100.00
Total	693	100.00

- **ce032s3 : Parents of the Grandchildren**

	No	%
3 HMemberChildren[3]	374	100.00
Total	374	100.00

- **ce032s4 : Parents of the Grandchildren**

	No	%
4 HMemberChildren[4]	171	100.00
Total	171	100.00

- **ce032s5 : Parents of the Grandchildren**

	No	%
5 HMemberChildren[5]	61	100.00
Total	61	100.00

- **ce032s6 : Parents of the Grandchildren**

	No	%
6 HMemberChildren[6]	23	100.00
Total	23	100.00

- **ce032s7 : Parents of the Grandchildren**

	No	%
7 HMemberChildren[7]	9	100.00
Total	9	100.00

- **ce032s8 : Parents of the Grandchildren**

	No	%
8 HMemberChildren[8]	3	100.00
Total	3	100.00

- **ce032s9 : Parents of the Grandchildren**

No Observations

- **ce032s10 : Parents of the Grandchildren**

	No	%
10 HMemberChildren[10]	1	100.00
Total	1	100.00

- **ce032s11 : Parents of the Grandchildren**

No Observations

- **ce032s12 : Parents of the Grandchildren**

No Observations

- **ce032s13 : Parents of the Grandchildren**

No Observations

- **ce032s14 : Parents of the Grandchildren**

No Observations

- **ce032s15 : Parents of the Grandchildren**

No Observations

- **ce032s16 : Parents of the Grandchildren**

No Observations

- **ce032s17 : Parents of the Grandchildren**

No Observations

- **ce032s18 : Parents of the Grandchildren**

No Observations

- **ce032s19 : Parents of the Grandchildren**

No Observations

- **ce032s20 : Parents of the Grandchildren**

No Observations

- **ce032s21 : Parents of the Grandchildren**

No Observations

- **ce032s22 : Parents of the Grandchildren**

No Observations

- **ce032s23 : Parents of the Grandchildren**

No Observations

- **ce032s24 : Parents of the Grandchildren**

No Observations

- **ce032s25 : Parents of the Grandchildren**

No Observations

- **ce033_1_1 : Regular Monetary Support**

Mean	Min	Max	OBS
498.8	3.0	5,000.0	138

- **ce033_1_1_a : Min Bracket**

No Observations

- **ce033_1_1_b : Max Bracket**

No Observations

- **ce033_1_1_every : Time Interval**

	No	%
1 Per month	19	13.67
2 Per season	2	1.44
3 Per half year	4	2.88
4 Per year	113	81.29
	1	0.72
Total	139	100.00

- **ce033_1_2 : Regular In-Kind Support**

Mean	Min	Max	OBS
720.0	50.0	2,000.0	5

- **ce033_1_2_a : Min Bracket**

No Observations

- **ce033_1_2_b : Max Bracket**

No Observations

- **ce033_1_2_every : Time Interval**

	No	%
1 Per month	4	57.14
2 Per season	1	14.29
4 Per year	1	14.29
	1	14.29
Total	7	100.00

- **ce033_1_3 : Non-Regular Monetary Support**

Mean	Min	Max	OBS
788.1	1.0	15,000.0	569

- **ce033_1_3_a : Min Bracket**

No Observations

- **ce033_1_3_b : Max Bracket**

No Observations

- **ce033_1_4 : Non-Regular In-Kind Support**

Mean	Min	Max	OBS
1,042.7	0.0	10,000.0	106

- **ce033_1_4_a : Min Bracket**

No Observations

- **ce033_1_4_b : Max Bracket**

No Observations

- **ce033_2_1 : Regular Monetary Support**

Mean	Min	Max	OBS
290.1	0.0	6,000.0	117

- **ce033_2_1_a : Min Bracket**

No Observations

- **ce033_2_1_b : Max Bracket**

No Observations

- **ce033_2_1_every : Time Interval**

	No	%
	1	0.86
1 Per month	4	3.45
3 Per half year	3	2.59
4 Per year	108	93.10
Total	116	100.00

- **ce033_2_2 : Regular In-Kind Support**

Mean	Min	Max	OBS
2,150.0	300.0	4,000.0	2

- **ce033_2_2_a : Min Bracket**

No Observations

- **ce033_2_2_b : Max Bracket**

No Observations

- **ce033_2_2_every : Time Interval**

	No	%
1 Per month	1	50.00
4 Per year	1	50.00
Total	2	100.00

- **ce033_2_3 : Non-Regular Monetary Support**

Mean	Min	Max	OBS
559.7	1.0	12,000.0	493

- **ce033_2_3_a : Min Bracket**

No Observations

- **ce033_2_3_b : Max Bracket**

No Observations

- **ce033_2_4 : Non-Regular In-Kind Support**

Mean	Min	Max	OBS
907.8	0.0	10,000.0	84

- **ce033_2_4_a : Min Bracket**

No Observations

- **ce033_2_4_b : Max Bracket**

No Observations

- **ce033_3_1 : Regular Monetary Support**

Mean	Min	Max	OBS
266.9	0.0	3,000.0	69

- **ce033_3_1_a : Min Bracket**

No Observations

- **ce033_3_1_b : Max Bracket**

No Observations

- **ce033_3_1_every : Time Interval**

	No	%
1 Per month	3	4.29
2 Per season	1	1.43
4 Per year	66	94.29
Total	70	100.00

- **ce033_3_2 : Regular In-Kind Support**

Mean	Min	Max	OBS
300.0	300.0	300.0	1

- **ce033_3_2_a : Min Bracket**

No Observations

- **ce033_3_2_b : Max Bracket**

No Observations

- **ce033_3_2_every : Time Interval**

	No	%
	1	50.00
4 Per year	1	50.00
Total	2	100.00

- **ce033_3_3 : Non-Regular Monetary Support**

Mean	Min	Max	OBS
437.8	0.0	20,000.0	271

- **ce033_3_3_a : Min Bracket**

Mean	Min	Max	OBS
101.0	101.0	101.0	1

- **ce033_3_3_b : Max Bracket**

Mean	Min	Max	OBS
199.0	199.0	199.0	1

- **ce033_3_4 : Non-Regular In-Kind Support**

Mean	Min	Max	OBS

336.1	0.0	1,500.0	31

- **ce033_3_4_a : Min Bracket**

No Observations

- **ce033_3_4_b : Max Bracket**

No Observations

- **ce033_4_1 : Regular Monetary Support**

Mean	Min	Max	OBS
169.1	0.0	1,000.0	33

- **ce033_4_1_a : Min Bracket**

No Observations

- **ce033_4_1_b : Max Bracket**

No Observations

- **ce033_4_1_every : Time Interval**

	No	%
	1	2.94
1 Per month	2	5.88
4 Per year	31	91.18
Total	34	100.00

- **ce033_4_2 : Regular In-Kind Support**

No Observations

- **ce033_4_2_a : Min Bracket**

No Observations

- **ce033_4_2_b : Max Bracket**

No Observations

- **ce033_4_2_every : Time Interval**

No Observations

- **ce033_4_3 : Non-Regular Monetary Support**

Mean	Min	Max	OBS
320.8	0.0	10,000.0	120

- **ce033_4_3_a : Min Bracket**

No Observations

- **ce033_4_3_b : Max Bracket**

No Observations

- **ce033_4_4 : Non-Regular In-Kind Support**

Mean	Min	Max	OBS
318.3	20.0	1,000.0	12

- **ce033_4_4_a : Min Bracket**

No Observations

- **ce033_4_4_b : Max Bracket**

No Observations

- **ce033_5_1 : Regular Monetary Support**

Mean	Min	Max	OBS
294.6	5.0	1,000.0	14

- **ce033_5_1_a : Min Bracket**

No Observations

- **ce033_5_1_b : Max Bracket**

No Observations

- **ce033_5_1_every : Time Interval**

	No	%
1 Per month	1	6.67
3 Per half year	1	6.67
4 Per year	12	80.00
	1	6.67
Total	15	100.00

- **ce033_5_2 : Regular In-Kind Support**

	No	%
0	1	100.00
Total	1	100.00

- **ce033_5_2_a : Min Bracket**

No Observations

- **ce033_5_2_b : Max Bracket**

No Observations

- **ce033_5_2_every : Time Interval**

	No	%
4 Per year	1	50.00
	1	50.00
Total	2	100.00

- **ce033_5_3 : Non-Regular Monetary Support**

Mean	Min	Max	OBS
232.2	5.0	4,000.0	43

- **ce033_5_3_a : Min Bracket**

No Observations

- **ce033_5_3_b : Max Bracket**

No Observations

- **ce033_5_4 : Non-Regular In-Kind Support**

Mean	Min	Max	OBS
140.0	20.0	200.0	3

- **ce033_5_4_a : Min Bracket**

No Observations

- **ce033_5_4_b : Max Bracket**

No Observations

- **ce033_6_1 : Regular Monetary Support**

Mean	Min	Max	OBS
110.0	30.0	200.0	3

- **ce033_6_1_a : Min Bracket**

No Observations

- **ce033_6_1_b : Max Bracket**

No Observations

- **ce033_6_1_every : Time Interval**

	No	%
	1	25.00
1 Per month	1	25.00
4 Per year	2	50.00
Total	4	100.00

- **ce033_6_2 : Regular In-Kind Support**

No Observations

- **ce033_6_2_a : Min Bracket**

No Observations

- **ce033_6_2_b : Max Bracket**

No Observations

- **ce033_6_2_every : Time Interval**

No Observations

- **ce033_6_3 : Non-Regular Monetary Support**

Mean	Min	Max	OBS
229.4	10.0	1,000.0	16

- **ce033_6_3_a : Min Bracket**

No Observations

- **ce033_6_3_b : Max Bracket**

No Observations

- **ce033_6_4 : Non-Regular In-Kind Support**

Mean	Min	Max	OBS
343.3	0.0	1,000.0	3

- **ce033_6_4_a : Min Bracket**

No Observations

- **ce033_6_4_b : Max Bracket**

No Observations

- **ce033_7_1 : Regular Monetary Support**

Mean	Min	Max	OBS
200.0	200.0	200.0	1

- **ce033_7_1_a : Min Bracket**

No Observations

- **ce033_7_1_b : Max Bracket**

No Observations

- **ce033_7_1_every : Time Interval**

	No	%
4 Per year	1	50.00
	1	50.00
Total	2	100.00

- **ce033_7_2 : Regular In-Kind Support**

No Observations

- **ce033_7_2_a : Min Bracket**

No Observations

- **ce033_7_2_b : Max Bracket**

No Observations

- **ce033_7_2_every : Time Interval**

No Observations

- **ce033_7_3 : Non-Regular Monetary Support**

Mean	Min	Max	OBS
78.0	20.0	200.0	5

- **ce033_7_3_a : Min Bracket**

No Observations

- **ce033_7_3_b : Max Bracket**

No Observations

- **ce033_7_4 : Non-Regular In-Kind Support**

Mean	Min	Max	OBS
500.0	0.0	1,000.0	2

- **ce033_7_4_a : Min Bracket**

No Observations

- **ce033_7_4_b : Max Bracket**

No Observations

- **ce033_8_1 : Regular Monetary Support**

No Observations

- **ce033_8_1_a : Min Bracket**

No Observations

- **ce033_8_1_b : Max Bracket**

No Observations

- **ce033_8_1_every : Time Interval**

No Observations

- **ce033_8_2 : Regular In-Kind Support**

No Observations

- **ce033_8_2_a : Min Bracket**

No Observations

- **ce033_8_2_b : Max Bracket**

No Observations

- **ce033_8_2_every : Time Interval**

No Observations

- **ce033_8_3 : Non-Regular Monetary Support**

Mean	Min	Max	OBS
100.0	0.0	200.0	2

- **ce033_8_3_a : Min Bracket**

No Observations

- **ce033_8_3_b : Max Bracket**

No Observations

- **ce033_8_4 : Non-Regular In-Kind Support**

No Observations

- **ce033_8_4_a : Min Bracket**

No Observations

- **ce033_8_4_b : Max Bracket**

No Observations

- **ce033_9_1 : Regular Monetary Support**

No Observations

- **ce033_9_1_a : Min Bracket**

No Observations

- **ce033_9_1_b : Max Bracket**

No Observations

- **ce033_9_1_every : Time Interval**

No Observations

- **ce033_9_2 : Regular In-Kind Support**

No Observations

- **ce033_9_2_a : Min Bracket**

No Observations

- **ce033_9_2_b : Max Bracket**

No Observations

- **ce033_9_2_every : Time Interval**

No Observations

- **ce033_9_3 : Non-Regular Monetary Support**

No Observations

- **ce033_9_3_a : Min Bracket**

No Observations

- **ce033_9_3_b : Max Bracket**

No Observations

- **ce033_9_4 : Non-Regular In-Kind Support**

No Observations

- **ce033_9_4_a : Min Bracket**

No Observations

- **ce033_9_4_b : Max Bracket**

No Observations

- **ce033_10_1 : Regular Monetary Support**

No Observations

- **ce033_10_1_a : Min Bracket**

No Observations

- **ce033_10_1_b : Max Bracket**

No Observations

- **ce033_10_1_every : Time Interval**

No Observations

- **ce033_10_2 : Regular In-Kind Support**

No Observations

- **ce033_10_2_a : Min Bracket**

No Observations

- **ce033_10_2_b : Max Bracket**

No Observations

- **ce033_10_2_every : Time Interval**

No Observations

- **ce033_10_3 : Non-Regular Monetary Support**

No Observations

- **ce033_10_3_a : Min Bracket**

No Observations

- **ce033_10_3_b : Max Bracket**

No Observations

- **ce033_10_4 : Non-Regular In-Kind Support**

Mean	Min	Max	OBS
300.0	300.0	300.0	1

- **ce033_10_4_a : Min Bracket**

No Observations

- **ce033_10_4_b : Max Bracket**

No Observations

- **ce035 : Any Transfer to Other Non-Coresident Relatives**

	No	%
1 Yes	370	3.67
2 No	9,720	96.33
Total	10,090	100.00

- **ce036_1 : Regular Monetary Support**

Mean	Min	Max	OBS
800.1	1.0	2,000.0	10

- **ce036_1_a : Min Bracket**

No Observations

- **ce036_1_b : Max Bracket**

No Observations

- **ce036_1_every : Time Interval**

	No	%
1 Per month	1	10.00
3 Per half year	1	10.00
4 Per year	8	80.00
Total	10	100.00

- **ce036_2 : Regular In-Kind Support**

Mean	Min	Max	OBS
7,550.0	100.0	15,000.0	2

- **ce036_2_a : Min Bracket**

No Observations

- **ce036_2_b : Max Bracket**

No Observations

- **ce036_2_every : Time Interval**

	No	%
	1	33.33
1 Per month	1	33.33
4 Per year	1	33.33
Total	3	100.00

- **ce036_3 : Non-Regular Monetary Support**

Mean	Min	Max	OBS
3,435.4	0.3	100,000.0	314

- **ce036_3_a : Min Bracket**

No Observations

- **ce036_3_b : Max Bracket**

No Observations

- **ce036_4 : Non-Regular In-Kind Support**

Mean	Min	Max	OBS
2,741.9	1.0	130,000.0	70

- **ce036_4_a : Min Bracket**

No Observations

- **ce036_4_b : Max Bracket**

No Observations

- **ce038 : Any Transfer to Non-Coresident Non-Relative (e.g. Friends)**

	No	%
1 Yes	104	1.03
2 No	9,988	98.97
Total	10,092	100.00

- **ce039_1 : Regular Monetary Support**

Mean	Min	Max	OBS
2,100.0	200.0	4,000.0	2

- **ce039_1_a : Min Bracket**

No Observations

- **ce039_1_b : Max Bracket**

No Observations

- **ce039_1_every : Time Interval**

	No	%
	1	33.33
4 Per year	2	66.67
Total	3	100.00

- **ce039_2 : Regular In-Kind Support**

No Observations

- **ce039_2_a : Min Bracket**

No Observations

- **ce039_2_b : Max Bracket**

No Observations

- **ce039_2_every : Time Interval**

No Observations

- **ce039_3 : Non-Regular Monetary Support**

Mean	Min	Max	OBS

2,889.8	1.0	60,000.0	82

- **ce039_3_a : Min Bracket**

No Observations

- **ce039_3_b : Max Bracket**

No Observations

- **ce039_4 : Non-Regular In-Kind Support**

Mean	Min	Max	OBS
952.8	0.0	5,000.0	18

- **ce039_4_a : Min Bracket**

No Observations

- **ce039_4_b : Max Bracket**

No Observations

- **ce041 : Ever Given a Large Amount of Money to Any of Your Children**

	No	%
1 Yes	1,380	13.70
2 No	8,696	86.30
Total	10,076	100.00

- **ce042s1 : Which Child(ren)**

	No	%
1 HMemberChildren[1]	1,043	100.00

Total	1,043	100.00

- **ce042s2 : Which Child(ren)**

	No	%
2 HMemberChildren[2]	604	100.00
Total	604	100.00

- **ce042s3 : Which Child(ren)**

	No	%
3 HMemberChildren[3]	237	100.00
Total	237	100.00

- **ce042s4 : Which Child(ren)**

	No	%
4 HMemberChildren[4]	84	100.00
Total	84	100.00

- **ce042s5 : Which Child(ren)**

	No	%
5 HMemberChildren[5]	26	100.00
Total	26	100.00

- **ce042s6 : Which Child(ren)**

	No	%
6 HMemberChildren[6]	7	100.00
Total	7	100.00

- **ce042s7 : Which Child(ren)**

	No	%
7 HMemberChildren[7]	1	100.00
Total	1	100.00

- **ce042s8 : Which Child(ren)**

	No	%
8 HMemberChildren[8]	1	100.00
Total	1	100.00

- **ce042s9 : Which Child(ren)**

No Observations

- **ce042s10 : Which Child(ren)**

No Observations

- **ce042s11 : Which Child(ren)**

No Observations

- **ce042s12 : Which Child(ren)**

No Observations

- **ce042s13 : Which Child(ren)**

No Observations

- **ce042s14 : Which Child(ren)**

No Observations

- **ce042s15 : Which Child(ren)**

No Observations

- **ce042s16 : Which Child(ren)**

No Observations

- **ce042s17 : Which Child(ren)**

No Observations

- **ce042s18 : Which Child(ren)**

No Observations

- **ce042s19 : Which Child(ren)**

No Observations

- **ce042s20 : Which Child(ren)**

No Observations

- **ce042s21 : Which Child(ren)**

No Observations

- **ce042s22 : Which Child(ren)**

No Observations

- **ce042s23 : Which Child(ren)**

No Observations

- **ce042s24 : Which Child(ren)**

No Observations

- **ce042s25 : Which Child(ren)**

No Observations

- **ce043_1_ : Value of Gift to Children**

Mean	Min	Max	OBS
43,739.0	-9,999.0	800,000.0	993

- **ce043_2_ : Value of Gift to Children**

Mean	Min	Max	OBS
29,925.1	0.0	400,000.0	567

- **ce043_3_ : Value of Gift to Children**

Mean	Min	Max	OBS
25,390.1	1,000.0	600,000.0	223

- **ce043_4_ : Value of Gift to Children**

Mean	Min	Max	OBS
21,171.4	5,000.0	120,000.0	77

- **ce043_5_ : Value of Gift to Children**

Mean	Min	Max	OBS
17,116.7	5,000.0	80,000.0	24

- **ce043_6_ : Value of Gift to Children**

Mean	Min	Max	OBS
54,833.3	7,000.0	130,000.0	6

- **ce043_7_ : Value of Gift to Children**

Mean	Min	Max	OBS
20,000.0	20,000.0	20,000.0	1

- **ce043_8_ : Value of Gift to Children**

No Observations

- **ce044_1_ : Year of Gift to Children**

Mean	Min	Max	OBS
2,005.1	1,963.0	2,011.0	965

- **ce044_2_ : Year of Gift to Children**

Mean	Min	Max	OBS
2,004.4	1,971.0	2,011.0	544

- **ce044_3_ : Year of Gift to Children**

Mean	Min	Max	OBS
2,003.7	1,977.0	2,011.0	212

- **ce044_4_ : Year of Gift to Children**

Mean	Min	Max	OBS
2,002.2	1,972.0	2,011.0	73

- **ce044_5_ : Year of Gift to Children**

Mean	Min	Max	OBS

1,999.5	1,970.0	2,011.0	23

- **ce044_6_ : Year of Gift to Children**

Mean	Min	Max	OBS
2,005.0	2,000.0	2,008.0	4

- **ce044_7_ : Year of Gift to Children**

Mean	Min	Max	OBS
2,000.0	2,000.0	2,000.0	1

- **ce044_8_ : Year of Gift to Children**

No Observations

- **ce045_1_ : Reason of Gift to Children**

	No	%
1 Pay for medical care or other emergency expense	43	4.30
2 Pay for house	193	19.32
4 Inheritance	22	2.20
5 Support grandchildrens education or welfare	21	2.10
6 Other	720	72.07
Total	999	100.00

- **ce045_2_ : Reason of Gift to Children**

	No	%
1 Pay for medical care or other emergency expense	13	2.25
2 Pay for house	113	19.55
4 Inheritance	12	2.08
5 Support grandchildrens education or welfare	6	1.04
6 Other	434	75.09
Total	578	100.00

- **ce045_3_ : Reason of Gift to Children**

	No	%
1 Pay for medical care or other emergency expense	10	4.41

	No	%
2 Pay for house	30	13.22
4 Inheritance	9	3.96
5 Support grandchildrens education or welfare	3	1.32
6 Other	175	77.09
Total	227	100.00

- **ce045_4_ : Reason of Gift to Children**

	No	%
1 Pay for medical care or other emergency expense	2	2.53
2 Pay for house	17	21.52
5 Support grandchildrens education or welfare	1	1.27
6 Other	59	74.68
Total	79	100.00

- **ce045_5_ : Reason of Gift to Children**

	No	%
2 Pay for house	4	16.67
6 Other	20	83.33
Total	24	100.00

- **ce045_6_ : Reason of Gift to Children**

	No	%
2 Pay for house	3	50.00
6 Other	3	50.00
Total	6	100.00

- **ce045_7_ : Reason of Gift to Children**

	No	%
2 Pay for house	1	100.00
Total	1	100.00

- **ce045_8_ : Reason of Gift to Children**

	No	%
6 Other	1	100.00
Total	1	100.00

- **ce046 : Ever Received Large Amount of Money from Children**

	No	%
1 Yes	422	4.19
2 No	9,657	95.81
Total	10,079	100.00

- **ce047s1 : Which Child(ren)**

	No	%
1 HMemberChildren[1]	305	100.00
Total	305	100.00

- **ce047s2 : Which Child(ren)**

	No	%
2 HMemberChildren[2]	165	100.00
Total	165	100.00

- **ce047s3 : Which Child(ren)**

	No	%
3 HMemberChildren[3]	67	100.00
Total	67	100.00

- **ce047s4 : Which Child(ren)**

	No	%
4 HMemberChildren[4]	30	100.00
Total	30	100.00

- **ce047s5 : Which Child(ren)**

	No	%
5 HMemberChildren[5]	8	100.00
Total	8	100.00

- **ce047s6 : Which Child(ren)**

	No	%

6 HMemberChildren[6]	2	100.00
Total	2	100.00

- **ce047s7 : Which Child(ren)**

	No	%
7 HMemberChildren[7]	2	100.00
Total	2	100.00

- **ce047s8 : Which Child(ren)**

No Observations

- **ce047s9 : Which Child(ren)**

No Observations

- **ce047s10 : Which Child(ren)**

No Observations

- **ce047s11 : Which Child(ren)**

No Observations

- **ce047s12 : Which Child(ren)**

No Observations

- **ce047s13 : Which Child(ren)**

No Observations

- **ce047s14 : Which Child(ren)**

No Observations

- **ce047s15 : Which Child(ren)**

No Observations

- **ce047s16 : Which Child(ren)**

No Observations

- **ce047s17 : Which Child(ren)**

No Observations

- **ce047s18 : Which Child(ren)**

No Observations

- **ce047s19 : Which Child(ren)**

No Observations

- **ce047s20 : Which Child(ren)**

No Observations

- **ce047s21 : Which Child(ren)**

No Observations

- **ce047s22 : Which Child(ren)**

No Observations

- **ce047s23 : Which Child(ren)**

No Observations

- **ce047s24 : Which Child(ren)**

No Observations

- **ce047s25 : Which Child(ren)**

No Observations

- **ce048_1_ : Value from this Child**

Mean	Min	Max	OBS
16,162.7	10.0	230,000.0	286

- **ce048_2_ : Value from this Child**

Mean	Min	Max	OBS
16,434.9	2.0	400,000.0	149

- **ce048_3_ : Value from this Child**

Mean	Min	Max	OBS
14,546.7	2.0	200,000.0	60

- **ce048_4_ : Value from this Child**

Mean	Min	Max	OBS
12,559.6	100.0	93,000.0	27

- **ce048_5_ : Value from this Child**

Mean	Min	Max	OBS
11,000.0	0.0	30,000.0	7

- **ce048_6_ : Value from this Child**

Mean	Min	Max	OBS
8,000.0	6,000.0	10,000.0	2

- **ce048_7_ : Value from this Child**

Mean	Min	Max	OBS
7,500.0	5,000.0	10,000.0	2

- **ce049_1_ : Year Received**

Mean	Min	Max	OBS
2,008.3	1,900.0	2,011.0	283

- **ce049_2_ : Year Received**

Mean	Min	Max	OBS
2,008.8	1,999.0	2,011.0	145

- **ce049_3_ : Year Received**

Mean	Min	Max	OBS
2,008.7	2,001.0	2,011.0	60

- **ce049_4_ : Year Received**

Mean	Min	Max	OBS
2,008.4	1,998.0	2,011.0	26

- **ce049_5_ : Year Received**

Mean	Min	Max	OBS
2,007.3	1,994.0	2,011.0	7

- **ce049_6_ : Year Received**

Mean	Min	Max	OBS
2,010.5	2,010.0	2,011.0	2

- **ce049_7_ : Year Received**

Mean	Min	Max	OBS
2,010.5	2,010.0	2,011.0	2

- **ce050_1_ : Why Received from this Child**

	No	%
1 Pay for medical care or other emergency expense	85	29.21
2 Pay for house	22	7.56
4 Other	184	63.23
Total	291	100.00

- **ce050_2_ : Why Received from this Child**

	No	%
1 Pay for medical care or other emergency expense	56	37.33
2 Pay for house	9	6.00
4 Other	85	56.67
Total	150	100.00

- **ce050_3_ : Why Received from this Child**

	No	%
1 Pay for medical care or other emergency expense	34	55.74
2 Pay for house	1	1.64
4 Other	26	42.62
Total	61	100.00

- **ce050_4_ : Why Received from this Child**

	No	%
1 Pay for medical care or other emergency expense	13	46.43
2 Pay for house	2	7.14
4 Other	13	46.43
Total	28	100.00

- **ce050_5_ : Why Received from this Child**

	No	%
1 Pay for medical care or other emergency expense	5	71.43
4 Other	2	28.57
Total	7	100.00

- **ce050_6_ : Why Received from this Child**

	No	%
1 Pay for medical care or other emergency expense	1	33.33
4 Other	2	66.67
Total	3	100.00

- **ce050_7_ : Why Received from this Child**

	No	%
1 Pay for medical care or other emergency expense	1	50.00
4 Other	1	50.00
Total	2	100.00

- **ce051 : Ever Given a Large Amount of Money to Your Parents**

	No	%
1 Yes	48	1.01
2 No	4,698	98.99
Total	4,746	100.00

- **ce052 : Value of Money to Your Parents**

Mean	Min	Max	OBS
18,780.4	2,000.0	100,000.0	51

- **ce053 : Year the Money to Your Parents**

Mean	Min	Max	OBS
2,005.0	1,986.0	2,010.0	47

- **ce054 : Reason for the Gift to Parents**

	No	%
1 pay for medical care or other emergency expense	34	65.38
2 pay for house	4	7.69
5 other	14	26.92
Total	52	100.00

- **ce055 : Ever Received a Large Amount of Money from Your Parents**

	No	%
1 Yes	50	1.05
2 No	4,694	98.95
Total	4,744	100.00

- **ce056 : Vaule of Money from Parents**

Mean	Min	Max	OBS
20,187.5	0.0	200,000.0	55

- **ce057 : Year the Money from Parents**

Mean	Min	Max	OBS
2,002.4	1,967.0	2,010.0	48

- **ce058 : Reason the Money from Parents**

	No	%
6 Pay for medical care or other emergency expense	3	5.66
7 Pay for house	7	13.21
9 Inheritance	19	35.85
10 Other	24	45.28
Total	53	100.00

- **ce059 : Ever Gave a Large Amount of Money to Your Parents-in-Law**

	No	%

1 Yes	27	0.60
2 No	4,489	99.40
Total	4,516	100.00

- **ce060 : Value of Money to Parents-in-Law**

Mean	Min	Max	OBS
14,492.6	0.0	80,000.0	27

- **ce061 : Year the Money to Parents-in-Law**

Mean	Min	Max	OBS
2,005.6	1,969.0	2,010.0	26

- **ce062 : Reason the Money to Parents-in_Law**

	No	%
1 pay for medical care or other emergency expense	16	57.14
2 pay for house	4	14.29
5 other	8	28.57
Total	28	100.00

- **ce063 : Ever Received a Large Amount of Money from Your Parents-in-Law**

	No	%
1 Yes	27	0.60
2 No	4,493	99.40
Total	4,520	100.00

- **ce064 : Value of Money from Parents-in-Law**

Mean	Min	Max	OBS
52,729.2	42.0	820,000.0	22

- **ce065 : Year the Money from Parents-in-Law**

Mean	Min	Max	OBS
2,003.8	1,979.0	2,010.0	23

- **ce066 : Reason the Money from Parents-in-Law**

	No	%
1 pay for medical care or other emergency expense	3	11.11
2 pay for house	3	11.11
4 Inheritance	6	22.22
5 other	15	55.56
Total	27	100.00

- **cf001 : Spend any Time Taking Care of Grandchildren**

	No	%
1 Yes	2,923	48.10
2 No	3,154	51.90
Total	6,077	100.00

- **cf002s1 : Which Child(ren)'s Children**

	No	%
1 HMemberChildren[1]	2,078	100.00
Total	2,078	100.00

- **cf002s2 : Which Child(ren)'s Children**

	No	%
2 HMemberChildren[2]	870	100.00
Total	870	100.00

- **cf002s3 : Which Child(ren)'s Children**

	No	%
3 HMemberChildren[3]	336	100.00
Total	336	100.00

- **cf002s4 : Which Child(ren)'s Children**

	No	%
4 HMemberChildren[4]	146	100.00
Total	146	100.00

- **cf002s5 : Which Child(ren)'s Children**

	No	%
5 HMemberChildren[5]	52	100.00
Total	52	100.00

- **cf002s6 : Which Child(ren)'s Children**

	No	%
6 HMemberChildren[6]	12	100.00
Total	12	100.00

- **cf002s7 : Which Child(ren)'s Children**

	No	%
7 HMemberChildren[7]	5	100.00
Total	5	100.00

- **cf002s8 : Which Child(ren)'s Children**

	No	%
8 HMemberChildren[8]	1	100.00
Total	1	100.00

- **cf002s9 : Which Child(ren)'s Children**

No Observations

- **cf002s10 : Which Child(ren)'s Children**

No Observations

- **cf002s11 : Which Child(ren)'s Children**

No Observations

- **cf002s12 : Which Child(ren)'s Children**

No Observations

- **cf002s13 : Which Child(ren)'s Children**

No Observations

- **cf002s14 : Which Child(ren)'s Children**

No Observations

- **cf002s15 : Which Child(ren)'s Children**

No Observations

- **cf002s16 : Which Child(ren)'s Children**

No Observations

- **cf002s17 : Which Child(ren)'s Children**

No Observations

- **cf002s18 : Which Child(ren)'s Children**

No Observations

- **cf002s19 : Which Child(ren)'s Children**

No Observations

- **cf002s20 : Which Child(ren)'s Children**

No Observations

- **cf002s21 : Which Child(ren)'s Children**

No Observations

- **cf002s22 : Which Child(ren)'s Children**

No Observations

- **cf002s23 : Which Child(ren)'s Children**

No Observations

- **cf002s24 : Which Child(ren)'s Children**

No Observations

- **cf002s25 : Which Child(ren)'s Children**

No Observations

- **cf002s26 : Which Child(ren)'s Children**

	No	%
26 Deceased Children	16	100.00
Total	16	100.00

- **cf002s27 : Which Child(ren)'s Children**

	No	%

27 Others	44	100.00
Total	44	100.00

- **cf003_1_1_ : Weeks**

	No	%
0	15	0.81
1	49	2.66
2	18	0.98
3	9	0.49
4	45	2.44
5	14	0.76
6	7	0.38
7	31	1.68
8	37	2.01
9	1	0.05
10	32	1.74
12	46	2.49
13	2	0.11
14	2	0.11
15	6	0.33
16	15	0.81
17	3	0.16
18	1	0.05
20	60	3.25
21	2	0.11
23	3	0.16
24	37	2.01
25	27	1.46
26	31	1.68
27	2	0.11
28	11	0.60
30	40	2.17
32	8	0.43
34	2	0.11
35	2	0.11
36	8	0.43
38	2	0.11
40	134	7.27
42	12	0.65
43	2	0.11
44	11	0.60
45	14	0.76
46	2	0.11
48	315	17.08
49	6	0.33
50	227	12.31
51	28	1.52
52	522	28.31
54	1	0.05

58	1	0.05
84	1	0.05
Total	1,844	100.00

- **cf003_1_2_ : Weeks**

Mean	Min	Max	OBS
34.9	0.0	110.0	728

- **cf003_1_3_ : Weeks**

	No	%
0	3	1.06
1	15	5.32
2	7	2.48
3	1	0.35
4	11	3.90
5	5	1.77
7	1	0.35
8	17	6.03
9	1	0.35
10	8	2.84
11	1	0.35
12	4	1.42
13	2	0.71
15	1	0.35
16	4	1.42
17	1	0.35
20	6	2.13
24	12	4.26
25	2	0.71
26	5	1.77
28	4	1.42
30	7	2.48
32	3	1.06
34	1	0.35
35	1	0.35
36	1	0.35
38	1	0.35
40	22	7.80
42	2	0.71
44	2	0.71
45	1	0.35
48	38	13.48
50	28	9.93
51	3	1.06
52	61	21.63
Total	282	100.00

- **cf003_1_4_ : Weeks**

	No	%
1	6	5.13
2	6	5.13
3	3	2.56
4	2	1.71
5	1	0.85
6	1	0.85
7	1	0.85
8	2	1.71
10	3	2.56
11	1	0.85
12	1	0.85
13	1	0.85
16	1	0.85
20	8	6.84
22	1	0.85
24	3	2.56
25	1	0.85
26	1	0.85
30	4	3.42
32	1	0.85
35	1	0.85
36	1	0.85
40	9	7.69
42	1	0.85
45	2	1.71
48	13	11.11
50	13	11.11
51	2	1.71
52	27	23.08
Total	117	100.00

- **cf003_1_5_ : Weeks**

	No	%
0	1	2.33
1	3	6.98
2	1	2.33
10	1	2.33
20	2	4.65
24	3	6.98
26	2	4.65
36	1	2.33
40	3	6.98
48	9	20.93
50	2	4.65
52	15	34.88
Total	43	100.00

- **cf003_1_6_ : Weeks**

	No	%
1	2	22.22
12	1	11.11
21	1	11.11
44	1	11.11
50	1	11.11
52	3	33.33
Total	9	100.00

- **cf003_1_7_ : Weeks**

	No	%
2	1	33.33
15	1	33.33
44	1	33.33
Total	3	100.00

- **cf003_1_8_ : Weeks**

	No	%
2	1	100.00
Total	1	100.00

- **cf003_1_26_ : Weeks**

	No	%
7	2	15.38
30	1	7.69
48	4	30.77
50	3	23.08
52	3	23.08
Total	13	100.00

- **cf003_1_27_ : Weeks**

	No	%
1	2	6.06
3	1	3.03
7	1	3.03
10	1	3.03
12	1	3.03
15	1	3.03

20	1	3.03
24	1	3.03
26	1	3.03
30	2	6.06
40	2	6.06
48	5	15.15
50	5	15.15
52	9	27.27
Total	33	100.00

- **cf003_1_28_ : Weeks**

	No	%
12	1	25.00
20	1	25.00
24	1	25.00
52	1	25.00
Total	4	100.00

- **cf003_1_s1 : Me**

	No	%
1 Myself: weeks; hours per week	1,882	100.00
Total	1,882	100.00

- **cf003_1_s2 : My Wife**

	No	%
2 My spouse: weeks; hours per week	1,191	100.00
Total	1,191	100.00

- **cf003_2_1_ : Hours Per Week**

Mean	Min	Max	OBS
53.9	0.0	5,000.0	1,791

- **cf003_2_2_ : Hours Per Week**

Mean	Min	Max	OBS
47.4	0.0	400.0	708

- **cf003_2_3_ : Hours Per Week**

Mean	Min	Max	OBS
46.1	0.0	140.0	276

- **cf003_2_4_ : Hours Per Week**

Mean	Min	Max	OBS
42.4	0.0	140.0	114

- **cf003_2_5_ : Hours Per Week**

Mean	Min	Max	OBS
56.0	0.0	160.0	40

- **cf003_2_6_ : Hours Per Week**

Mean	Min	Max	OBS
63.6	5.0	140.0	8

- **cf003_2_7_ : Hours Per Week**

	No	%
2	1	33.33
8	1	33.33
14	1	33.33
Total	3	100.00

- **cf003_2_8_ : Hours Per Week**

	No	%
4	1	100.00
Total	1	100.00

- **cf003_2_26_ : Hours Per Week**

Mean	Min	Max	OBS
47.2	1.0	140.0	13

- **cf003_2_27_ : Hours Per Week**

Mean	Min	Max	OBS
42.9	1.0	140.0	32

- **cf003_2_28_ : Hours Per Week**

	No	%
0	2	50.00
10	1	25.00
21	1	25.00
Total	4	100.00

- **cf003_2_s1 : Me**

	No	%
1 Myself: weeks; hours per week	745	100.00
Total	745	100.00

- **cf003_2_s2 : My Wife**

	No	%
2 My spouse: weeks; hours per week	519	100.00
Total	519	100.00

- **cf003_3_1_ : Weeks**

Mean	Min	Max	OBS
37.0	0.0	521.0	1,170

- **cf003_3_2_ : Weeks**

	No	%
0	17	3.37
1	17	3.37
2	13	2.57
3	4	0.79
4	16	3.17
5	3	0.59
6	1	0.20
7	6	1.19
8	14	2.77

9	1	0.20
10	10	1.98
11	1	0.20
12	15	2.97
13	2	0.40
15	4	0.79
16	6	1.19
17	1	0.20
20	19	3.76
24	25	4.95
25	10	1.98
26	8	1.58
27	1	0.20
28	5	0.99
30	10	1.98
32	6	1.19
35	1	0.20
36	4	0.79
40	36	7.13
42	5	0.99
44	2	0.40
45	2	0.40
48	68	13.47
49	1	0.20
50	37	7.33
51	6	1.19
52	128	25.35
Total	505	100.00

- **cf003_3_3_ : Weeks**

	No	%
0	4	2.15
1	7	3.76
2	4	2.15
3	2	1.08
4	7	3.76
5	2	1.08
7	1	0.54
8	6	3.23
9	1	0.54
10	7	3.76
12	4	2.15
13	1	0.54
15	1	0.54
16	2	1.08
20	8	4.30
24	8	4.30
25	1	0.54
26	4	2.15
28	2	1.08

30	5	2.69
32	4	2.15
38	1	0.54
40	14	7.53
42	3	1.61
44	2	1.08
45	1	0.54
46	1	0.54
48	24	12.90
50	16	8.60
51	2	1.08
52	41	22.04
Total	186	100.00

- **cf003_3_4_ : Weeks**

	No	%
0	4	5.06
1	3	3.80
2	5	6.33
3	1	1.27
4	3	3.80
6	1	1.27
8	1	1.27
10	2	2.53
11	1	1.27
12	1	1.27
13	1	1.27
20	6	7.59
22	1	1.27
24	2	2.53
25	1	1.27
40	6	7.59
42	1	1.27
45	1	1.27
48	8	10.13
49	1	1.27
50	12	15.19
51	2	2.53
52	15	18.99
Total	79	100.00

- **cf003_3_5_ : Weeks**

	No	%
2	1	4.00
8	1	4.00
20	2	8.00
26	1	4.00

36	1	4.00
40	2	8.00
48	7	28.00
52	10	40.00
Total	25	100.00

- **cf003_3_6_ : Weeks**

	No	%
1	3	42.86
12	1	14.29
44	1	14.29
52	2	28.57
Total	7	100.00

- **cf003_3_7_ : Weeks**

	No	%
13	1	33.33
15	1	33.33
44	1	33.33
Total	3	100.00

- **cf003_3_8_ : Weeks**

No Observations

- **cf003_3_26_ : Weeks**

	No	%
7	1	12.50
25	1	12.50
30	1	12.50
48	2	25.00
50	2	25.00
52	1	12.50
Total	8	100.00

- **cf003_3_27_ : Weeks**

	No	%
0	1	5.88

1	1	5.88
2	1	5.88
7	1	5.88
12	1	5.88
15	1	5.88
16	1	5.88
20	1	5.88
26	1	5.88
50	3	17.65
52	5	29.41
Total	17	100.00

- **cf003_3_28_ : Weeks**

	No	%
12	1	25.00
20	1	25.00
24	1	25.00
52	1	25.00
Total	4	100.00

- **cf003_3_s1 : Me**

	No	%
1 Myself: weeks; hours per week	285	100.00
Total	285	100.00

- **cf003_3_s2 : My Wife**

	No	%
2 My spouse: weeks; hours per week	187	100.00
Total	187	100.00

- **cf003_4_1_ : Hours Per Week**

Mean	Min	Max	OBS
51.7	0.0	5,000.0	1,124

- **cf003_4_2_ : Hours Per Week**

Mean	Min	Max	OBS
45.2	0.0	400.0	484

- **cf003_4_3_ : Hours Per Week**

Mean	Min	Max	OBS
50.5	0.0	168.0	180

- **cf003_4_4_ : Hours Per Week**

Mean	Min	Max	OBS
47.3	0.0	140.0	76

- **cf003_4_5_ : Hours Per Week**

Mean	Min	Max	OBS
62.6	3.0	140.0	21

- **cf003_4_6_ : Hours Per Week**

Mean	Min	Max	OBS
74.7	5.0	140.0	6

- **cf003_4_7_ : Hours Per Week**

	No	%
8	1	50.00
14	1	50.00
Total	2	100.00

- **cf003_4_8_ : Hours Per Week**

No Observations

- **cf003_4_26_ : Hours Per Week**

Mean	Min	Max	OBS
56.6	3.0	140.0	8

- **cf003_4_27_ : Hours Per Week**

	No	%
0	1	6.25
5	1	6.25
6	2	12.50
10	2	12.50
14	1	6.25
42	1	6.25
45	1	6.25
50	1	6.25
56	2	12.50
70	3	18.75
100	1	6.25
Total	16	100.00

- **cf003_4_28_ : Hours Per Week**

	No	%
0	2	50.00
10	1	25.00
21	1	25.00
Total	4	100.00

- **cf003_4_s1 : Me**

	No	%
1 Myself: weeks; hours per week	120	100.00
Total	120	100.00

- **cf003_4_s2 : My Wife**

	No	%
2 My spouse: weeks; hours per week	83	100.00
Total	83	100.00

- **cf003_5_s1 : Me**

	No	%
1 Myself: weeks; hours per week	42	100.00
Total	42	100.00

- **cf003_5_s2 : My Wife**

	No	%
2 My spouse: weeks; hours per week	24	100.00
Total	24	100.00

- **cf003_6_s1 : Me**

	No	%
1 Myself: weeks; hours per week	9	100.00
Total	9	100.00

- **cf003_6_s2 : My Wife**

	No	%
2 My spouse: weeks; hours per week	7	100.00
Total	7	100.00

- **cf003_7_s1 : Me**

	No	%
1 Myself: weeks; hours per week	3	100.00
Total	3	100.00

- **cf003_7_s2 : My Wife**

	No	%
2 My spouse: weeks; hours per week	3	100.00
Total	3	100.00

- **cf003_8_s1 : Me**

	No	%
1 Myself: weeks; hours per week	1	100.00
Total	1	100.00

- **cf003_8_s2 : My Wife**

No Observations

- **cf003_26_s1 : Me**

	No	%
1 Myself: weeks; hours per week	13	100.00
Total	13	100.00

- **cf003_26_s2 : My Wife**

	No	%
2 My spouse: weeks; hours per week	8	100.00
Total	8	100.00

- **cf003_27_s1 : Me**

	No	%
1 Myself: weeks; hours per week	32	100.00
Total	32	100.00

- **cf003_27_s2 : My Wife**

	No	%
2 My spouse: weeks; hours per week	18	100.00
Total	18	100.00

- **cf003_28_s1 : Me**

	No	%
1 Myself: weeks; hours per week	5	100.00
Total	5	100.00

- **cf003_28_s2 : My Wife**

	No	%
2 My spouse: weeks; hours per week	4	100.00
Total	4	100.00

- **cf004 : Take Care of Your Parents or Parents-in-Law**

	No	%
1 Yes	1,315	13.03

2 No	8,775	86.97
Total	10,090	100.00

- **cf005_1 : Weeks**

	No	%
0	41	10.12
1	42	10.37
2	38	9.38
3	20	4.94
4	28	6.91
5	8	1.98
6	4	0.99
7	3	0.74
8	16	3.95
10	12	2.96
12	18	4.44
13	2	0.49
15	2	0.49
16	6	1.48
17	1	0.25
20	12	2.96
24	19	4.69
25	2	0.49
26	3	0.74
27	2	0.49
30	2	0.49
32	2	0.49
34	1	0.25
36	1	0.25
39	1	0.25
40	8	1.98
42	1	0.25
48	33	8.15
50	27	6.67
51	1	0.25
52	49	12.10
Total	405	100.00

- **cf005_2 : Hours Per Week**

Mean	Min	Max	OBS
22.0	0.0	148.0	396

- **cf005_3 : Weeks**

	No	%

0	36	5.25
1	56	8.16
2	61	8.89
3	30	4.37
4	35	5.10
5	14	2.04
6	7	1.02
7	10	1.46
8	29	4.23
10	19	2.77
11	1	0.15
12	42	6.12
13	1	0.15
15	6	0.87
16	11	1.60
18	1	0.15
20	26	3.79
24	15	2.19
25	6	0.87
26	7	1.02
27	2	0.29
28	3	0.44
30	8	1.17
33	1	0.15
34	2	0.29
36	2	0.29
39	1	0.15
40	20	2.92
42	1	0.15
43	1	0.15
44	1	0.15
48	81	11.81
49	1	0.15
50	48	7.00
51	4	0.58
52	97	14.14
Total	686	100.00

- **cf005_4 : Hours Per Week**

Mean	Min	Max	OBS
25.3	0.0	168.0	679

- **cf005_5 : Weeks**

	No	%
0	52	16.46
1	37	11.71
2	32	10.13

3	11	3.48
4	15	4.75
5	10	3.16
6	3	0.95
7	2	0.63
8	8	2.53
10	6	1.90
11	1	0.32
12	18	5.70
13	1	0.32
14	2	0.63
15	2	0.63
16	4	1.27
17	3	0.95
20	10	3.16
24	4	1.27
25	6	1.90
26	5	1.58
27	4	1.27
30	6	1.90
35	1	0.32
36	1	0.32
40	10	3.16
44	1	0.32
48	14	4.43
50	10	3.16
51	1	0.32
52	36	11.39
Total	316	100.00

- **cf005_6 : Hours Per Week**

Mean	Min	Max	OBS
18.4	0.0	140.0	301

- **cf005_7 : Weeks**

	No	%
0	53	10.11
1	60	11.45
2	53	10.11
3	17	3.24
4	24	4.58
5	17	3.24
6	4	0.76
7	4	0.76
8	15	2.86
10	17	3.24
11	1	0.19

12	30	5.73
13	3	0.57
14	2	0.38
15	3	0.57
16	11	2.10
17	2	0.38
18	2	0.38
20	20	3.82
21	1	0.19
23	1	0.19
24	11	2.10
25	7	1.34
26	7	1.34
27	4	0.76
28	1	0.19
30	7	1.34
31	1	0.19
32	1	0.19
33	1	0.19
36	3	0.57
40	15	2.86
45	1	0.19
48	28	5.34
50	26	4.96
51	4	0.76
52	67	12.79
Total	524	100.00

- **cf005_8 : Hours Per Week**

Mean	Min	Max	OBS
22.8	0.0	168.0	513

- **cf005s1 : Which Parents or Parents-in-Law**

	No	%
1 Your father	415	100.00
Total	415	100.00

- **cf005s2 : Which Parents or Parents-in-Law**

	No	%
2 Your mother	706	100.00
Total	706	100.00

- **cf005s3 : Which Parents or Parents-in-Law**

	No	%
3 Your father-in-law	321	100.00
Total	321	100.00

- **cf005s4 : Which Parents or Parents-in-Law**

	No	%
4 Your mother-in-law	526	100.00
Total	526	100.00

- **cf006_1 : Weeks**

	No	%
0	79	25.40
1	35	11.25
2	36	11.58
3	8	2.57
4	17	5.47
5	4	1.29
7	2	0.64
8	10	3.22
10	4	1.29
12	11	3.54
13	3	0.96
16	4	1.29
17	1	0.32
20	8	2.57
24	14	4.50
25	1	0.32
26	5	1.61
27	3	0.96
30	3	0.96
32	2	0.64
36	1	0.32
40	3	0.96
42	1	0.32
45	1	0.32
48	17	5.47
50	14	4.50
52	24	7.72
Total	311	100.00

- **cf006_2 : Hours Per Week**

Mean	Min	Max	OBS

17.2	0.0	148.0	304

- **cf006_3 : Weeks**

	No	%
0	88	18.45
1	52	10.90
2	37	7.76
3	17	3.56
4	17	3.56
5	7	1.47
6	2	0.42
7	5	1.05
8	18	3.77
10	15	3.14
12	30	6.29
13	1	0.21
15	2	0.42
16	11	2.31
17	1	0.21
20	14	2.94
24	12	2.52
25	4	0.84
26	7	1.47
27	2	0.42
30	6	1.26
32	2	0.42
34	1	0.21
35	1	0.21
36	1	0.21
40	6	1.26
42	1	0.21
45	1	0.21
48	50	10.48
50	16	3.35
51	1	0.21
52	49	10.27
Total	477	100.00

- **cf006_4 : Hours Per Week**

Mean	Min	Max	OBS
21.3	0.0	200.0	468

- **cf006_5 : Weeks**

	No	%
0	60	19.74
1	37	12.17
2	26	8.55
3	15	4.93
4	13	4.28
5	5	1.64
6	1	0.33
7	1	0.33
8	6	1.97
10	12	3.95
11	1	0.33
12	18	5.92
13	1	0.33
14	2	0.66
15	2	0.66
16	6	1.97
17	2	0.66
20	3	0.99
24	6	1.97
25	6	1.97
26	7	2.30
27	3	0.99
30	6	1.97
33	1	0.33
34	1	0.33
36	1	0.33
40	7	2.30
48	12	3.95
50	11	3.62
52	32	10.53
Total	304	100.00

- **cf006_6 : Hours Per Week**

Mean	Min	Max	OBS
16.3	0.0	140.0	294

- **cf006_7 : Weeks**

	No	%
0	53	10.71
1	60	12.12
2	45	9.09
3	18	3.64
4	19	3.84
5	17	3.43
6	4	0.81

7	3	0.61
8	13	2.63
9	1	0.20
10	24	4.85
11	1	0.20
12	29	5.86
13	2	0.40
14	2	0.40
15	3	0.61
16	11	2.22
17	1	0.20
18	2	0.40
20	18	3.64
24	13	2.63
25	6	1.21
26	11	2.22
27	3	0.61
30	13	2.63
31	1	0.20
32	1	0.20
35	1	0.20
36	2	0.40
40	11	2.22
42	1	0.20
48	28	5.66
50	25	5.05
51	3	0.61
52	50	10.10
Total	495	100.00

- **cf006_8 : Hours Per Week**

Mean	Min	Max	OBS
20.7	0.0	140.0	486

- **cf006s1 : Which Parents or Parents-in-Law**

	No	%
1 Your father	303	100.00
Total	303	100.00

- **cf006s2 : Which Parents or Parents-in-Law**

	No	%
2 Your mother	472	100.00
Total	472	100.00

- **cf006s3 : Which Parents or Parents-in-Law**

	No	%
3 Your father-in-law	301	100.00
Total	301	100.00

- **cf006s4 : Which Parents or Parents-in-Law**

	No	%
4 Your mother-in-law	493	100.00
Total	493	100.00

- **cg001_1_ : Best Living Arrangement**

	No	%
1 Live with adult children	5,294	54.55
2 Dont live with them in the same house, but live in the same community or village	3,665	37.77
3 Dont live with them in the same house and the same community or village	391	4.03
4 Live in a nursing house	223	2.30
5 Other	131	1.35
Total	9,704	100.00

- **cg001_2_ : Best Living Arrangement**

	No	%
1 Live with adult children	3,924	53.99
2 Dont live with them in the same house, but live in the same community or village	2,720	37.42
3 Dont live with them in the same house and the same community or village	231	3.18
4 Live in a nursing house	126	1.73
5 Other	267	3.67
Total	7,268	100.00

- **cg001_3_ : Best Living Arrangement**

	No	%
3 Dont live with them in the same house and the same community or village	1	100.00
Total	1	100.00

- **cg002_1_ : Best Living Arrangement**

	No	%
1 Live with adult children	6,601	68.19

	No	%
2 Dont live with them in the same house, but live in the same community or village.	2,342	24.19
3 Dont live with them in the same house and the same community or village.	223	2.30
4 Live in the nursing house	354	3.66
5 Other	160	1.65
Total	9,680	100.00

- **cg002_2_ : Best Living Arrangement**

	No	%
1 Live with adult children	4,907	67.62
2 Dont live with them in the same house, but live in the same community or village.	1,700	23.43
3 Dont live with them in the same house and the same community or village.	148	2.04
4 Live in the nursing house	217	2.99
5 Other	285	3.93
Total	7,257	100.00

- **cg002_3_ : Best Living Arrangement**

	No	%
1 Live with adult children	8	66.67
2 Dont live with them in the same house, but live in the same community or village.	2	16.67
3 Dont live with them in the same house and the same community or village.	1	8.33
5 Other	1	8.33
Total	12	100.00

- **cg003 : Receive Assistance in Interview**

	No	%
1 Never	8,082	80.08
2 A few times	1,754	17.38
3 Most or all the time	256	2.54
Total	10,092	100.00

- **proxy : Interview Down By Proxy**

	No	%
0 No	9,836	97.19
1 Yes	284	2.81
Total	10,120	100.00

5 Health Status and Functioning

- **ID : Individual ID**

A String Variable	
OBS:	17,596

- **householdID : Household ID**

A String Variable	
OBS:	17,596

- **communityID : Community ID**

A String Variable	
OBS:	17,596

- **da001 : Self Comment of Your Health**

	No	%
1 Excellent	63	0.72
2 Very good	748	8.58
3 Good	1,427	16.38
4 Fair	4,031	46.26
5 Poor	2,444	28.05
Total	8,713	100.00

- **da002 : Self Comment of Your Health**

	No	%
1 Very good	527	5.94
2 Good	1,437	16.21
3 Fair	4,139	46.68
4 Poor	2,280	25.72
5 Very poor	483	5.45
Total	8,866	100.00

- **da003 : Pain on the Left Side of Your Chest**

	No	%
1 Yes	3,030	18.63
2 No	13,237	81.37

Total	16,267	100.00

- **da004 : Chest Pains When Climbing Stairs/Uphill or Walking Quickly**

	No	%
1 Yes	2,119	13.03
2 No	13,607	83.66
3 Not applicable	538	3.31
Total	16,264	100.00

- **da005_1_ : Physical Disabilities**

	No	%
1 Yes	746	4.24
2 No	16,830	95.76
Total	17,576	100.00

- **da005_2_ : Brain Damage/Mental Retardation**

	No	%
1 Yes	523	2.98
2 No	17,042	97.02
Total	17,565	100.00

- **da005_3_ : Vision Problem**

	No	%
1 Yes	1,093	6.22
2 No	16,485	93.78
Total	17,578	100.00

- **da005_4_ : Hearing Problem**

	No	%
1 Yes	1,570	8.93
2 No	16,002	91.07
Total	17,572	100.00

- **da005_5_ : Speech Impediment**

	No	%

1 Yes	101	0.58
2 No	17,458	99.42
Total	17,559	100.00

- **da006_1_ : The Year Becoming Physical Disabilities**

Mean	Min	Max	OBS
1,989.2	1,918.0	2,011.0	730

- **da006_2_ : The Year Becoming Brain Damage/Mental Retardation**

Mean	Min	Max	OBS
1,993.6	1,936.0	2,011.0	500

- **da006_3_ : The Year Becoming Vision Problem**

Mean	Min	Max	OBS
2,000.7	1,900.0	2,011.0	1,067

- **da006_4_ : The Year Becoming Hearing Problem**

Mean	Min	Max	OBS
2,000.2	1,900.0	2,011.0	1,492

- **da006_5_ : The Year Becoming Speech Impediment**

Mean	Min	Max	OBS
1,984.6	1,930.0	2,011.0	96

- **da007_1_ : Hypertension**

	No	%
1 Yes	4,284	24.52
2 No	13,186	75.48
Total	17,470	100.00

- **da007_2_ : Dyslipidemia**

	No	%

1 Yes	1,595	9.27
2 No	15,618	90.73
Total	17,213	100.00

- **da007_3_ : Disabetes or High Blood Sugar**

	No	%
1 Yes	993	5.70
2 No	16,415	94.30
Total	17,408	100.00

- **da007_4_ : Cancer or Malignant Tumor**

	No	%
1 Yes	180	1.03
2 No	17,306	98.97
Total	17,486	100.00

- **da007_5_ : Chronic Lung Diseases**

	No	%
1 Yes	1,781	10.18
2 No	15,715	89.82
Total	17,496	100.00

- **da007_6_ : Liver Disease**

	No	%
1 Yes	676	3.88
2 No	16,762	96.12
Total	17,438	100.00

- **da007_7_ : Heart Problems**

	No	%
1 Yes	2,093	11.98
2 No	15,374	88.02
Total	17,467	100.00

- **da007_8_ : Stroke**

	No	%

1 Yes	413	2.36
2 No	17,113	97.64
Total	17,526	100.00

- **da007_9_ : Kidney Diease**

	No	%
1 Yes	1,106	6.34
2 No	16,346	93.66
Total	17,452	100.00

- **da007_10_ : Stomach or Other Digestive Disease**

	No	%
1 Yes	3,902	22.28
2 No	13,615	77.72
Total	17,517	100.00

- **da007_11_ : Emotional, Nervous, or Psychiatric Problems**

	No	%
1 Yes	251	1.44
2 No	17,239	98.56
Total	17,490	100.00

- **da007_12_ : Memory-Related Disease**

	No	%
1 Yes	277	1.58
2 No	17,230	98.42
Total	17,507	100.00

- **da007_13_ : Arthritis or Rheumatism**

	No	%
1 Yes	5,773	32.94
2 No	11,751	67.06
Total	17,524	100.00

- **da007_14_ : Asthma**

	No	%
1 Yes	637	3.64
2 No	16,860	96.36
Total	17,497	100.00

- **da008_1_ : Do You Konw If You Have Hypertension**

	No	%
1 Yes	143	1.08
2 No	11,529	86.87
3 Dont know	1,600	12.06
Total	13,272	100.00

- **da008_5_ : Do You Konw If You Have Chronic Lung Diseases**

	No	%
1 Yes	304	1.93
2 No	13,896	88.00
3 Dont know	1,591	10.08
Total	15,791	100.00

- **da008_11_ : Do You Konw If You Have Emotional, Nervous,or Psychiatric Problems**

	No	%
1 Yes	111	0.64
2 No	15,836	91.82
3 Dont know	1,299	7.53
Total	17,246	100.00

- **da009_1_ : The Time of Being Diagnosed of Hypertension**

	No	%
1 Year	3,465	79.09
2 Age	916	20.91
Total	4,381	100.00

- **da009_1_1_ : The Year of Being Diagnosed of Hypertension**

Mean	Min	Max	OBS
2,005.7	1,945.0	2,011.0	3,444

- **da009_1_2_ : The Year of Being Diagnosed of Dyslipidemia**

Mean	Min	Max	OBS
2,006.7	1,965.0	2,011.0	1,364

- **da009_1_3_ : The Year of Being Diagnosed of Disabetes or High Blood Sugar**

Mean	Min	Max	OBS
2,006.5	1,971.0	2,011.0	843

- **da009_1_4_ : The Year of Being Diagnosed of Cancer or Malignant Tumor**

Mean	Min	Max	OBS
2,004.7	1,970.0	2,011.0	148

- **da009_1_5_ : The Year of Being Diagnosed of Chronic Lung Diseases**

Mean	Min	Max	OBS
2,002.2	1,948.0	2,011.0	1,296

- **da009_1_6_ : The Year of Being Diagnosed of Liver Disease**

Mean	Min	Max	OBS
2,002.5	1,900.0	2,011.0	480

- **da009_1_7_ : The Year of Being Diagnosed of Heart Problems**

Mean	Min	Max	OBS
2,003.9	1,940.0	2,011.0	1,586

- **da009_1_8_ : The Year of Being Diagnosed of Stroke**

Mean	Min	Max	OBS
2,004.1	1,937.0	2,011.0	313

- **da009_1_9_ : The Year of Being Diagnosed of Kidney Diease**

Mean	Min	Max	OBS
2,003.3	1,900.0	2,011.0	842

- **da009_1_10_ : The Year of Being Diagnosed of Stomach or Other Digestive Disease**

Mean	Min	Max	OBS
2,001.3	1,900.0	2,011.0	2,529

- **da009_1_11_ : The Year of Being Diagnosed of Emotional, Nervous, or Psychiatric Problems**

Mean	Min	Max	OBS
1,998.8	1,950.0	2,011.0	248

- **da009_1_12_ : The Year of Being Diagnosed of Memory-Related Disease**

Mean	Min	Max	OBS
2,006.2	1,960.0	2,011.0	247

- **da009_1_13_ : The Year of Being Diagnosed of Arthritis or Rheumatism**

Mean	Min	Max	OBS
2,001.6	1,900.0	2,011.0	3,718

- **da009_1_14_ : The Year of Being Diagnosed of Asthma**

Mean	Min	Max	OBS
2,001.0	1,948.0	2,011.0	388

- **da009_2_ : The Time of Being Diagnosed of Dyslipidemia**

	No	%
1 Year	1,378	85.96
2 Age	225	14.04
Total	1,603	100.00

- **da009_2_1_ : The Age of Being Diagnosed of Hypertension**

	No	%
3	1	0.11
6	1	0.11
15	1	0.11
16	5	0.55
17	2	0.22
18	7	0.77
19	4	0.44
20	9	0.99
21	2	0.22
22	2	0.22
23	3	0.33
24	5	0.55
25	5	0.55
26	4	0.44
27	1	0.11
28	4	0.44
29	1	0.11
30	20	2.21
31	4	0.44
32	4	0.44
33	3	0.33
34	3	0.33
35	21	2.32
36	13	1.43
37	14	1.55
38	8	0.88
39	3	0.33
40	82	9.05
41	5	0.55
42	23	2.54
43	11	1.21
44	10	1.10
45	66	7.28
46	20	2.21
47	23	2.54
48	21	2.32
49	16	1.77
50	110	12.14
51	14	1.55
52	21	2.32
53	18	1.99
54	21	2.32
55	36	3.97
56	19	2.10
57	10	1.10
58	14	1.55
59	12	1.32
60	72	7.95
61	9	0.99
62	7	0.77

63	4	0.44
64	11	1.21
65	16	1.77
66	4	0.44
67	5	0.55
68	6	0.66
69	4	0.44
70	26	2.87
71	6	0.66
72	4	0.44
73	7	0.77
74	2	0.22
75	4	0.44
76	2	0.22
77	4	0.44
78	1	0.11
79	3	0.33
80	3	0.33
81	1	0.11
86	1	0.11
87	2	0.22
Total	906	100.00

- **da009_2_2_ : The Age of Being Diagnosed of Dyslipidemia**

	No	%
20	1	0.46
23	1	0.46
25	1	0.46
26	1	0.46
28	1	0.46
30	4	1.84
31	1	0.46
32	1	0.46
34	3	1.38
36	3	1.38
37	6	2.76
38	2	0.92
39	2	0.92
40	14	6.45
41	1	0.46
42	8	3.69
43	1	0.46
44	3	1.38
45	13	5.99
46	6	2.76
47	5	2.30
48	4	1.84
49	5	2.30
50	28	12.90
51	3	1.38

52	13	5.99
53	5	2.30
54	4	1.84
55	19	8.76
56	7	3.23
57	6	2.76
58	4	1.84
59	3	1.38
60	11	5.07
61	4	1.84
62	1	0.46
63	7	3.23
64	3	1.38
65	4	1.84
67	2	0.92
70	2	0.92
72	1	0.46
74	1	0.46
75	1	0.46
79	1	0.46
Total	217	100.00

- **da009_2_3_ : The Age of Being Diagnosed of Disabetes or High Blood Sugar**

	No	%
6	1	0.65
19	1	0.65
20	2	1.29
30	2	1.29
31	1	0.65
33	2	1.29
35	1	0.65
36	1	0.65
37	1	0.65
38	1	0.65
39	3	1.94
40	9	5.81
41	3	1.94
42	5	3.23
43	2	1.29
44	1	0.65
45	13	8.39
46	6	3.87
47	5	3.23
48	4	2.58
49	2	1.29
50	12	7.74
51	2	1.29
52	6	3.87
53	4	2.58

54	1	0.65
55	10	6.45
56	7	4.52
57	3	1.94
58	8	5.16
59	2	1.29
60	11	7.10
61	3	1.94
62	3	1.94
63	2	1.29
64	1	0.65
65	4	2.58
66	2	1.29
70	2	1.29
71	1	0.65
72	1	0.65
78	2	1.29
80	1	0.65
81	1	0.65
Total	155	100.00

- **da009_2_4_ : The Age of Being Diagnosed of Cancer or Malignant Tumor**

	No	%
24	1	2.86
27	1	2.86
30	2	5.71
31	1	2.86
33	2	5.71
37	1	2.86
38	1	2.86
39	2	5.71
40	5	14.29
42	1	2.86
45	2	5.71
46	3	8.57
47	1	2.86
48	1	2.86
51	2	5.71
54	2	5.71
56	2	5.71
60	1	2.86
61	1	2.86
67	1	2.86
71	1	2.86
76	1	2.86
Total	35	100.00

- **da009_2_5_ : The Age of Being Diagnosed of Chronic Lung Diseases**

	No	%
1	18	2.47
2	3	0.41
3	8	1.10
4	7	0.96
5	10	1.37
6	11	1.51
7	10	1.37
8	9	1.23
9	3	0.41
10	14	1.92
11	2	0.27
12	8	1.10
13	11	1.51
14	3	0.41
15	10	1.37
16	8	1.10
17	8	1.10
18	15	2.06
19	2	0.27
20	27	3.70
21	1	0.14
22	5	0.69
23	10	1.37
24	8	1.10
25	23	3.16
26	8	1.10
27	3	0.41
28	3	0.41
29	1	0.14
30	60	8.23
31	1	0.14
32	7	0.96
33	7	0.96
34	14	1.92
35	16	2.19
36	9	1.23
37	5	0.69
38	11	1.51
39	3	0.41
40	75	10.29
41	3	0.41
42	5	0.69
43	4	0.55
44	1	0.14
45	27	3.70
46	10	1.37
47	5	0.69
48	10	1.37
49	4	0.55
50	59	8.09

51	4	0.55
52	7	0.96
53	8	1.10
54	7	0.96
55	7	0.96
56	3	0.41
57	7	0.96
58	6	0.82
59	2	0.27
60	40	5.49
61	6	0.82
62	4	0.55
63	3	0.41
64	5	0.69
65	4	0.55
66	3	0.41
67	4	0.55
69	2	0.27
70	11	1.51
71	3	0.41
73	3	0.41
75	2	0.27
77	1	0.14
80	1	0.14
86	1	0.14
Total	729	100.00

- **da009_2_6_ : The Age of Being Diagnosed of Liver Disease**

	No	%
1	2	1.11
5	1	0.56
7	1	0.56
8	1	0.56
9	1	0.56
10	4	2.22
12	4	2.22
13	2	1.11
15	2	1.11
16	3	1.67
18	4	2.22
19	2	1.11
20	6	3.33
21	3	1.67
22	2	1.11
23	3	1.67
24	3	1.67
25	4	2.22
26	3	1.67
27	1	0.56
28	4	2.22

30	12	6.67
31	3	1.67
32	7	3.89
33	5	2.78
35	7	3.89
36	4	2.22
37	8	4.44
38	5	2.78
39	2	1.11
40	13	7.22
41	2	1.11
43	3	1.67
44	2	1.11
45	7	3.89
46	4	2.22
47	1	0.56
50	9	5.00
51	1	0.56
52	4	2.22
53	1	0.56
54	2	1.11
55	4	2.22
56	1	0.56
57	1	0.56
58	1	0.56
59	1	0.56
60	4	2.22
61	1	0.56
62	2	1.11
63	1	0.56
64	1	0.56
67	1	0.56
70	2	1.11
78	1	0.56
80	1	0.56
Total	180	100.00

- **da009_2_7_ : The Age of Being Diagnosed of Heart Problems**

	No	%
1	5	1.04
2	1	0.21
5	1	0.21
6	1	0.21
10	3	0.63
12	1	0.21
13	2	0.42
14	3	0.63
15	1	0.21
16	3	0.63
17	2	0.42

18	5	1.04
19	1	0.21
20	3	0.63
21	1	0.21
22	1	0.21
23	4	0.84
24	2	0.42
25	10	2.09
26	2	0.42
27	2	0.42
28	2	0.42
29	2	0.42
30	24	5.01
31	6	1.25
32	4	0.84
33	5	1.04
34	5	1.04
35	17	3.55
36	8	1.67
37	17	3.55
38	11	2.30
39	3	0.63
40	41	8.56
41	6	1.25
42	7	1.46
43	11	2.30
44	5	1.04
45	28	5.85
46	8	1.67
47	5	1.04
48	8	1.67
49	7	1.46
50	41	8.56
51	5	1.04
52	17	3.55
53	4	0.84
54	3	0.63
55	19	3.97
56	9	1.88
57	7	1.46
58	3	0.63
59	6	1.25
60	34	7.10
61	2	0.42
62	2	0.42
63	4	0.84
64	4	0.84
65	5	1.04
66	2	0.42
67	2	0.42
68	4	0.84
69	1	0.21

70	9	1.88
73	1	0.21
74	1	0.21
75	2	0.42
76	1	0.21
77	2	0.42
78	1	0.21
79	1	0.21
81	1	0.21
82	1	0.21
85	1	0.21
Total	479	100.00

- **da009_2_8_ : The Age of Being Diagnosed of Stroke**

	No	%
5	1	1.02
7	1	1.02
10	1	1.02
15	2	2.04
16	1	1.02
18	1	1.02
20	3	3.06
23	1	1.02
25	2	2.04
26	2	2.04
29	2	2.04
30	3	3.06
33	1	1.02
35	5	5.10
36	2	2.04
37	5	5.10
40	7	7.14
41	1	1.02
42	2	2.04
44	1	1.02
45	1	1.02
46	3	3.06
47	2	2.04
48	1	1.02
49	4	4.08
50	9	9.18
51	1	1.02
53	1	1.02
54	2	2.04
55	1	1.02
57	3	3.06
58	4	4.08
59	2	2.04
61	1	1.02
62	1	1.02

65	2	2.04
66	2	2.04
67	2	2.04
68	1	1.02
70	3	3.06
72	2	2.04
73	1	1.02
75	2	2.04
77	1	1.02
80	1	1.02
81	1	1.02
Total	98	100.00

- **da009_2_9_ : The Age of Being Diagnosed of Kidney Diease**

	No	%
3	1	0.39
8	1	0.39
10	1	0.39
15	2	0.78
16	2	0.78
17	2	0.78
18	1	0.39
19	1	0.39
20	8	3.14
22	4	1.57
23	1	0.39
25	7	2.75
26	3	1.18
27	4	1.57
28	3	1.18
30	37	14.51
31	2	0.78
32	3	1.18
33	1	0.39
34	3	1.18
35	12	4.71
36	5	1.96
37	5	1.96
38	4	1.57
39	2	0.78
40	23	9.02
41	1	0.39
42	4	1.57
43	2	0.78
45	17	6.67
46	5	1.96
47	4	1.57
48	6	2.35
50	21	8.24
51	6	2.35

52	5	1.96
53	1	0.39
54	4	1.57
56	2	0.78
57	2	0.78
58	2	0.78
59	3	1.18
60	13	5.10
61	1	0.39
62	2	0.78
63	1	0.39
65	3	1.18
66	3	1.18
67	4	1.57
68	1	0.39
70	2	0.78
77	1	0.39
81	1	0.39
Total	255	100.00

- **da009_2_10_ : The Age of Being Diagnosed of Stomach or Other Digestive Disease**

	No	%
1	1	0.08
2	1	0.08
3	2	0.16
5	1	0.08
6	5	0.40
7	1	0.08
8	2	0.16
9	2	0.16
10	14	1.13
11	2	0.16
12	4	0.32
13	6	0.48
14	5	0.40
15	15	1.21
16	26	2.09
17	15	1.21
18	28	2.25
19	8	0.64
20	69	5.55
21	14	1.13
22	19	1.53
23	23	1.85
24	23	1.85
25	59	4.75
26	23	1.85
27	23	1.85
28	11	0.88

29	7	0.56
30	160	12.87
31	5	0.40
32	15	1.21
33	12	0.97
34	21	1.69
35	62	4.99
36	19	1.53
37	9	0.72
38	20	1.61
39	4	0.32
40	148	11.91
41	13	1.05
42	14	1.13
43	15	1.21
44	10	0.80
45	44	3.54
46	18	1.45
47	7	0.56
48	16	1.29
49	6	0.48
50	72	5.79
51	5	0.40
52	13	1.05
53	8	0.64
54	12	0.97
55	20	1.61
56	13	1.05
57	7	0.56
58	8	0.64
59	2	0.16
60	22	1.77
61	2	0.16
62	4	0.32
63	2	0.16
64	4	0.32
65	4	0.32
66	1	0.08
68	3	0.24
69	1	0.08
70	6	0.48
73	1	0.08
74	3	0.24
76	2	0.16
78	1	0.08
Total	1,243	100.00

- **da009_2_11_ : The Age of Being Diagnosed of Emotional, Nervous, or Psychiatric Problems**

	No	%

1	2	1.98
5	2	1.98
6	1	0.99
7	1	0.99
9	1	0.99
10	4	3.96
11	1	0.99
13	2	1.98
15	2	1.98
16	4	3.96
17	2	1.98
18	3	2.97
20	2	1.98
22	1	0.99
23	2	1.98
24	2	1.98
25	3	2.97
26	1	0.99
27	2	1.98
28	3	2.97
30	7	6.93
31	2	1.98
33	1	0.99
35	2	1.98
38	3	2.97
40	8	7.92
41	2	1.98
42	1	0.99
43	2	1.98
44	1	0.99
45	6	5.94
47	1	0.99
49	1	0.99
50	6	5.94
51	1	0.99
53	4	3.96
55	1	0.99
56	1	0.99
57	1	0.99
58	1	0.99
59	1	0.99
60	3	2.97
62	1	0.99
63	1	0.99
72	1	0.99
74	1	0.99
Total	101	100.00

- **da009_2_12_ : The Age of Being Diagnosed of Memory-Related Disease**

	No	%
15	1	3.45
35	1	3.45
40	3	10.34
43	1	3.45
44	1	3.45
48	1	3.45
50	1	3.45
52	2	6.90
57	1	3.45
58	3	10.34
60	2	6.90
67	2	6.90
70	2	6.90
72	1	3.45
73	1	3.45
75	1	3.45
76	1	3.45
77	1	3.45
78	1	3.45
80	2	6.90
Total	29	100.00

- **da009_2_13_ : The Age of Being Diagnosed of Arthritis or Rheumatism**

	No	%
1	1	0.05
6	2	0.11
7	5	0.27
8	7	0.37
9	5	0.27
10	15	0.80
11	1	0.05
12	12	0.64
13	9	0.48
14	9	0.48
15	13	0.69
16	22	1.17
17	18	0.96
18	26	1.38
19	10	0.53
20	81	4.31
21	6	0.32
22	19	1.01
23	8	0.43
24	17	0.90
25	50	2.66
26	27	1.44
27	14	0.74
28	17	0.90

29	4	0.21
30	186	9.89
31	13	0.69
32	10	0.53
33	5	0.27
34	14	0.74
35	78	4.15
36	28	1.49
37	27	1.44
38	20	1.06
39	6	0.32
40	274	14.57
41	8	0.43
42	38	2.02
43	30	1.59
44	19	1.01
45	114	6.06
46	30	1.59
47	19	1.01
48	33	1.75
49	11	0.58
50	183	9.73
51	12	0.64
52	26	1.38
53	17	0.90
54	14	0.74
55	44	2.34
56	14	0.74
57	25	1.33
58	14	0.74
59	11	0.58
60	64	3.40
61	5	0.27
62	8	0.43
63	7	0.37
64	7	0.37
65	16	0.85
66	4	0.21
67	4	0.21
68	5	0.27
69	2	0.11
70	13	0.69
71	1	0.05
72	3	0.16
73	4	0.21
74	2	0.11
75	6	0.32
76	2	0.11
77	1	0.05
78	1	0.05
80	1	0.05
82	2	0.11

83	1	0.05
84	1	0.05
Total	1,881	100.00

- **da009_2_14_ : The Age of Being Diagnosed of Asthma**

	No	%
1	8	3.51
3	4	1.75
5	5	2.19
6	5	2.19
7	3	1.32
8	2	0.88
10	6	2.63
11	1	0.44
12	2	0.88
13	4	1.75
14	2	0.88
15	5	2.19
16	6	2.63
17	2	0.88
18	6	2.63
20	6	2.63
22	3	1.32
23	1	0.44
24	1	0.44
25	4	1.75
28	2	0.88
29	2	0.88
30	20	8.77
32	2	0.88
33	2	0.88
34	1	0.44
35	6	2.63
36	3	1.32
37	2	0.88
38	2	0.88
39	1	0.44
40	17	7.46
41	1	0.44
42	1	0.44
43	1	0.44
45	12	5.26
46	2	0.88
47	2	0.88
48	3	1.32
50	20	8.77
51	1	0.44
52	1	0.44
53	2	0.88
54	2	0.88

55	12	5.26
56	1	0.44
57	2	0.88
59	1	0.44
60	8	3.51
61	1	0.44
62	2	0.88
65	1	0.44
66	1	0.44
67	1	0.44
68	2	0.88
69	1	0.44
70	7	3.07
72	1	0.44
73	1	0.44
74	1	0.44
75	1	0.44
Total	228	100.00

- **da009_3_ : The Time of Being Diagnosed of Disabetes or High Blood Sugar**

	No	%
1 Year	850	84.49
2 Age	156	15.51
Total	1,006	100.00

- **da009_4_ : The Time of Being Diagnosed of Cancer or Malignant Tumor**

	No	%
1 Year	152	80.85
2 Age	36	19.15
Total	188	100.00

- **da009_5_ : The Time of Being Diagnosed of Chronic Lung Diseases**

	No	%
1 Year	1,306	63.96
2 Age	736	36.04
Total	2,042	100.00

- **da009_6_ : The Time of Being Diagnosed of Liver Disease**

	No	%
1 Year	487	72.36

2 Age	186	27.64
Total	673	100.00

- **da009_7_ : The Time of Being Diagnosed of Heart Problems**

	No	%
1 Year	1,599	76.69
2 Age	486	23.31
Total	2,085	100.00

- **da009_8_ : The Time of Being Diagnosed of Stroke**

	No	%
1 Year	314	75.85
2 Age	100	24.15
Total	414	100.00

- **da009_9_ : The Time of Being Diagnosed of Kidney Diease**

	No	%
1 Year	848	76.67
2 Age	258	23.33
Total	1,106	100.00

- **da009_10_ : The Time of Being Diagnosed of Stomach or Other Digestive Disease**

	No	%
1 Year	2,549	67.19
2 Age	1,245	32.81
Total	3,794	100.00

- **da009_11_ : The Time of Being Diagnosed of Emotional, Nervous, or Psychiatric Problems**

	No	%
1 Year	253	70.08
2 Age	108	29.92
Total	361	100.00

- **da009_12_ : The Time of Being Diagnosed of Memory-Related Disease**

	No	%
1 Year	252	88.73
2 Age	32	11.27
Total	284	100.00

- **da009_13_ : The Time of Being Diagnosed of Arthritis or Rheumatism**

	No	%
1 Year	3,744	66.43
2 Age	1,892	33.57
Total	5,636	100.00

- **da009_14_ : The Time of Being Diagnosed of Asthma**

	No	%
1 Year	394	63.04
2 Age	231	36.96
Total	625	100.00

- **da010_2_s1 : Take Chinese Traditional Medicine for Dyslipidemia**

	No	%
1 Taking Chinese traditional medicine	193	100.00
Total	193	100.00

- **da010_2_s2 : Take Western Morden Medicine for Dyslipidemia**

	No	%
2 taking Western morden medicine ,	780	100.00
Total	780	100.00

- **da010_2_s3 : Other Treatments for Dyslipidemia**

	No	%
3 Other treatments	109	100.00
Total	109	100.00

- **da010_2_s4 : None of the Above for Dyslipide**

	No	%

4 None of the above	681	100.00
Total	681	100.00

- **da010_5_s1 : Take Chinese Traditional Medicine for Chronic Lung Diseases**

	No	%
1 Taking Chinese traditional medicine	358	100.00
Total	358	100.00

- **da010_5_s2 : Take Western Morden Medicine for Chronic Lung Diseases**

	No	%
2 taking Western morden medicine ,	1,058	100.00
Total	1,058	100.00

- **da010_5_s3 : Other Treatments for Chronic Lung Diseases**

	No	%
3 Other treatments	169	100.00
Total	169	100.00

- **da010_5_s4 : None of the Above for Chronic Lung Diseases**

	No	%
4 None of the above	832	100.00
Total	832	100.00

- **da010_6_s1 : Take Chinese Traditional Medicine for Liver Disease**

	No	%
1 Taking Chinese traditional medicine	138	100.00
Total	138	100.00

- **da010_6_s2 : Take Western Morden Medicine for Liver Disease**

	No	%
2 taking Western morden medicine ,	254	100.00
Total	254	100.00

- **da010_6_s3 : Other Treatments for Liver Disease**

	No	%
3 Other treatments	51	100.00
Total	51	100.00

- **da010_6_s4 : None of the Above for Liver Disease**

	No	%
4 None of the above	344	100.00
Total	344	100.00

- **da010_7_s1 : Take Chinese Traditional Medicine for Heart Problems**

	No	%
1 Taking Chinese traditional medicine	438	100.00
Total	438	100.00

- **da010_7_s2 : Take Western Morden Medicine for Heart Problems**

	No	%
2 taking Western morden medicine ,	1,219	100.00
Total	1,219	100.00

- **da010_7_s3 : Other Treatments for Heart Problems**

	No	%
3 Other treatments	127	100.00
Total	127	100.00

- **da010_7_s4 : None of the Above for Heart Problems**

	No	%
4 None of the above	647	100.00
Total	647	100.00

- **da010_9_s1 : Take Chinese Traditional Medicine for Kidney Diease**

	No	%
1 Taking Chinese traditional medicine	268	100.00

Total	268	100.00

- **da010_9_s2 : Take Western Morden Medicine for Kidney Diease**

	No	%
2 taking Western morden medicine ,	415	100.00
Total	415	100.00

- **da010_9_s3 : Other Treatments for Kidney Diease**

	No	%
3 Other treatments	142	100.00
Total	142	100.00

- **da010_9_s4 : None of the Above for Kidney Diease**

	No	%
4 None of the above	511	100.00
Total	511	100.00

- **da010_10_s1 : Take Chinese Traditional Medicine for Stomach or Other Digestive Disease**

	No	%
1 Taking Chinese traditional medicine	786	100.00
Total	786	100.00

- **da010_10_s2 : Take Western Morden Medicine for Stomach or Other Digestive Disease**

	No	%
2 taking Western morden medicine ,	2,149	100.00
Total	2,149	100.00

- **da010_10_s3 : Other Treatments for Stomach or Other Digestive Disease**

	No	%
3 Other treatments	209	100.00
Total	209	100.00

- **da010_10_s4 : None of the Above for Stomach or Other Digestive Disease**

	No	%
4 None of the above	1,389	100.00
Total	1,389	100.00

- **da010_12_s1 : Take Chinese Traditional Medicine for Memory-Related Disease**

	No	%
1 Taking Chinese traditional medicine	42	100.00
Total	42	100.00

- **da010_12_s2 : Take Western Morden Medicine for Memory-Related Disease**

	No	%
2 taking Western morden medicine ,	117	100.00
Total	117	100.00

- **da010_12_s3 : Other Treatments for Memory-Related Disease**

	No	%
3 Other treatments	18	100.00
Total	18	100.00

- **da010_12_s4 : None of the Above for Memory-Related Disease**

	No	%
4 None of the above	141	100.00
Total	141	100.00

- **da010_13_s1 : Take Chinese Traditional Medicine for Arthritis or Rheumatism**

	No	%
1 Taking Chinese traditional medicine	1,099	100.00
Total	1,099	100.00

- **da010_13_s2 : Take Western Morden Medicine for Arthritis or Rheuma-**

tism

	No	%
2 taking Western morden medicine ,	2,216	100.00
Total	2,216	100.00

- **da010_13_s3 : Other Treatments for Arthritis or Rheumatism**

	No	%
3 Other treatments	1,290	100.00
Total	1,290	100.00

- **da010_13_s4 : None of the Above for Arthritis or Rheumatism**

	No	%
4 None of the above	2,329	100.00
Total	2,329	100.00

- **da011s1 : Take Chinese Traditional Medicine for Hypertension**

	No	%
1 Taking Chinese traditional medicine	477	100.00
Total	477	100.00

- **da011s2 : Take Western Morden Medicine for Hypertension**

	No	%
2 Taking Western morden medicine	3,201	100.00
Total	3,201	100.00

- **da011s3 : None of the Above for Hypertension**

	No	%
3 None of the above	993	100.00
Total	993	100.00

- **da012 : Times of Blood Pressure Examination Last Year**

Mean	Min	Max	OBS
26.4	0.0	999.0	4,286

- **da013s1 : Got Any Health Advice from doctor of Weight Control**

	No	%
1 Weight control	1,074	100.00
Total	1,074	100.00

- **da013s2 : Got Any Health Advice from doctor of Exercise**

	No	%
2 Exercise	1,420	100.00
Total	1,420	100.00

- **da013s3 : Got Any Health Advice from doctor of Diet**

	No	%
3 Diet	2,065	100.00
Total	2,065	100.00

- **da013s4 : Got Any Health Advice from doctor of Smoking Control**

	No	%
4 Smoking control	964	100.00
Total	964	100.00

- **da013s5 : Got Any Health Advice from doctor of None of the Above**

	No	%
5 None of the above	1,938	100.00
Total	1,938	100.00

- **da014s1 : Take Chinese Traditional Medicine for Diabetes**

	No	%
1 Taking Chinese traditional medicine	123	100.00
Total	123	100.00

- **da014s2 : Take Western Morden Medicine for Diabetes**

	No	%
2 Taking Western morden medicine	581	100.00

Total	581	100.00

- **da014s3 : Taking Insulin Injections for Diabetes**

	No	%
3 Taking insulin injections	137	100.00
Total	137	100.00

- **da014s4 : None of the Above for Diabetes**

	No	%
4 None of the above	287	100.00
Total	287	100.00

- **da015_1_a : How Many Times Have You Had Diebetes Tests of Blood Glucose Test**

Mean	Min	Max	OBS
11.8	0.0	365.0	758

- **da015_2_a : How Many Times Have You Had Diebetes Tests of Urine Glucose Test**

	No	%
0	46	11.47
1	151	37.66
2	66	16.46
3	37	9.23
4	17	4.24
5	11	2.74
6	14	3.49
7	2	0.50
10	11	2.74
11	1	0.25
12	25	6.23
20	1	0.25
24	2	0.50
30	5	1.25
36	2	0.50
48	4	1.00
50	1	0.25
52	2	0.50
72	1	0.25
84	1	0.25
96	1	0.25

Total	401	100.00

- **da015_3_a : How Many Times Have You Had Diebetes Tests of Fundus Examination**

	No	%
0	74	31.22
1	91	38.40
2	27	11.39
3	13	5.49
4	5	2.11
5	6	2.53
6	3	1.27
10	2	0.84
12	10	4.22
24	1	0.42
30	4	1.69
50	1	0.42
Total	237	100.00

- **da015_4_a : How Many Times Have You Had Diebetes Tests of Micro-albuminuria Test**

	No	%
0	55	25.00
1	85	38.64
2	30	13.64
3	12	5.45
4	8	3.64
5	6	2.73
6	1	0.45
10	2	0.91
12	13	5.91
24	1	0.45
30	4	1.82
48	1	0.45
50	1	0.45
84	1	0.45
Total	220	100.00

- **da015s1 : Have You Had Diebetes Tests of Blood Glucose Test**

	No	%
1 Blood glucose test Times	757	100.00
Total	757	100.00

- **da015s2 : Have You Had Diebetes Tests of Urine Glucose Test**

	No	%
2 Urine glucose test Times	400	100.00
Total	400	100.00

- **da015s3 : Have You Had Diebetes Tests of Fundus Examination**

	No	%
3 fundus examination Times	233	100.00
Total	233	100.00

- **da015s4 : Have You Had Diebetes Tests of Micro-albuminuria Test**

	No	%
4 micro-albuminuria test Times	218	100.00
Total	218	100.00

- **da015s5 : None Diebetes Tests Have Had**

	No	%
5 None of the above	205	100.00
Total	205	100.00

- **da016s1 : Got Any Health Advice from doctor of Weight Control**

	No	%
1 Weight control	363	100.00
Total	363	100.00

- **da016s2 : Got Any Health Advice from doctor of Exercise**

	No	%
2 Exercise	468	100.00
Total	468	100.00

- **da016s3 : Got Any Health Advice from doctor of Diet**

	No	%
3 Diet	697	100.00

Total	697	100.00

- **da016s4 : Got Any Health Advice from doctor of Smoking Control**

	No	%
4 Smoking control	263	100.00
Total	263	100.00

- **da016s5 : Got Any Health Advice from doctor of Foot Self-care**

	No	%
5 Foot self-care	143	100.00
Total	143	100.00

- **da016s6 : Got Any Health Advice from doctor of None of the Above**

	No	%
6 None of the above	236	100.00
Total	236	100.00

- **da017s1 : The Brain Had or Having Cancer**

	No	%
1 Brain	8	100.00
Total	8	100.00

- **da017s2 : The Oral Had or Cavity Having Cancer**

	No	%
2 Oral cavity	2	100.00
Total	2	100.00

- **da017s3 : The Larynx Had or Having Cancer**

	No	%
3 Larynx	3	100.00
Total	3	100.00

- **da017s4 : The Other Pharynx Had or Having Cancer**

	No	%
4 Other pharynx	3	100.00
Total	3	100.00

- **da017s5 : The Thyroid Had or Having Cancer**

	No	%
5 Thyroid	9	100.00
Total	9	100.00

- **da017s6 : The Lung Had or Having Cancer**

	No	%
6 Lung	13	100.00
Total	13	100.00

- **da017s7 : The Breast Had or Having Cancer**

	No	%
7 Breast	25	100.00
Total	25	100.00

- **da017s8 : The Oesophagus Had or Having Cancer**

	No	%
8 Oesophagus	10	100.00
Total	10	100.00

- **da017s9 : The Stomach Had or Having Cancer**

	No	%
9 Stomach	11	100.00
Total	11	100.00

- **da017s10 : The Liver Had or Having Cancer**

	No	%
10 Liver	7	100.00
Total	7	100.00

- **da017s11 : The Procreas Had or Having Cancer**

	No	%
11 Pancreas	1	100.00
Total	1	100.00

- **da017s12 : The Kidney Had or Having Cancer**

	No	%
12 Kidney	3	100.00
Total	3	100.00

- **da017s13 : The Prostate Had or Having Cancer**

No Observations

- **da017s14 : The Testicle Had or Having Cancer**

No Observations

- **da017s15 : The Ovary Had or Having Cancer**

	No	%
15 Ovary	8	100.00
Total	8	100.00

- **da017s16 : The Cervix Had or Having Cancer**

	No	%
16 Cervix	28	100.00
Total	28	100.00

- **da017s17 : The Endometrium Had or Having Cancer**

	No	%
17 Endometrium	26	100.00
Total	26	100.00

- **da017s18 : The Colon or Rectum Had or Having Cancer**

	No	%
18 Colon or rectum	13	100.00
Total	13	100.00

- **da017s19 : The Bladder Had or Having Cancer**

	No	%
19 Bladder	1	100.00
Total	1	100.00

- **da017s20 : The Skin Had or Having Cancer**

	No	%
20 Skin	4	100.00
Total	4	100.00

- **da017s21 : The Non-Hodgkin Lymphoma Had or Having Cancer**

	No	%
21 Non-Hodgkin lymphoma	1	100.00
Total	1	100.00

- **da017s22 : The Leukemia Had or Having Cancer**

	No	%
22 Leukemia	1	100.00
Total	1	100.00

- **da017s23 : The Other Organ Had or Having Cancer**

	No	%
23 Other organ	22	100.00
Total	22	100.00

- **da018s1 : Y/N Treat Cancer by Take Chinese Traditional Medicine**

	No	%
1 Taking Chinese traditional medicine	32	100.00

Total	32	100.00

- **da018s2 : Y/N Treat Cancer by Take Western Morden Medicine**

	No	%
2 Taking Western morden medicine	83	100.00
Total	83	100.00

- **da018s3 : Y/N Treat Cancer by Chemotherapy**

	No	%
3 Chemotherapy	30	100.00
Total	30	100.00

- **da018s4 : Y/N Treat Cancer by Surgery**

	No	%
4 Surgery	69	100.00
Total	69	100.00

- **da018s5 : Y/N Treat Cancer by Radiation Therapy**

	No	%
5 Radiation therapy	13	100.00
Total	13	100.00

- **da018s6 : Y/N Treat Cancer by None of the Above**

	No	%
6 None of the above	53	100.00
Total	53	100.00

- **da019s1 : Y/N Treat Stroke by Take Chinese Traditional Medicine**

	No	%
1 Taking Chinese traditional medicine	120	100.00
Total	120	100.00

- **da019s2 : Y/N Treat Stroke by Take Western Morden Medicine**

	No	%
2 Taking Western morden medicine	222	100.00
Total	222	100.00

- **da019s3 : Y/N Treat Stroke by Physical Therapy**

	No	%
3 Physical therapy	37	100.00
Total	37	100.00

- **da019s4 : Y/N Treat Stroke by Acupuncture and Moxibustion**

	No	%
4 Acupuncture and moxibustion	53	100.00
Total	53	100.00

- **da019s5 : Y/N Treat Stroke by Occupational Therapy**

	No	%
5 Occupational therapy	12	100.00
Total	12	100.00

- **da019s6 : Y/N Treat Stroke by None of the Above**

	No	%
6 None of the above	127	100.00
Total	127	100.00

- **da020s1 : Y/N Treat Emotional or Psychiatric Problems by Psychological Treatment**

	No	%
1 Receiving psychiatric or psychological treatment	42	100.00
Total	42	100.00

- **da020s2 : Y/N Treat Emotional or Psychiatric Problems by Taking Anti Depressants**

	No	%

2 Taking anti depressants	49	100.00
Total	49	100.00

- **da020s3 : Y/N Treat Emotional or Psychiatric Problems by Taking Sleeping Pills**

	No	%
3 Taking tranquilizers or sleeping pills	87	100.00
Total	87	100.00

- **da020s4 : Y/N Treat Emotional or Psychiatric Problems by None of the Above**

	No	%
4 None of the above	222	100.00
Total	222	100.00

- **da021 : Traffic Accident or Major Accidental Injury**

	No	%
1 Yes	1,603	9.87
2 No	14,641	90.13
Total	16,244	100.00

- **da022 : Y/N Injury Limit Your Daily Activities**

	No	%
1 Yes	837	51.73
2 No	781	48.27
Total	1,618	100.00

- **da023 : Y/N Fallen Down**

	No	%
1 Yes	2,549	15.70
2 No	13,691	84.30
Total	16,240	100.00

- **da024 : The Number of Times of Falling**

Mean	Min	Max	OBS

1.6	0.0	1,460.0	2,546

- **da025 : Y/N Fractured Hip**

	No	%
1 Yes	262	1.61
2 No	15,980	98.39
Total	16,242	100.00

- **da026_1 : The Year When You Began the Menarche**

Mean	Min	Max	OBS
237.4	0.0	1,996.0	669

- **da026_2 : The Age When You Began the Menarche**

Mean	Min	Max	OBS
16.9	0.0	1,982.0	7,528

- **da027 : Y/N Started Menopause**

	No	%
1 Yes	6,091	72.08
2 No	2,359	27.92
Total	8,450	100.00

- **da028_1 : The Year Started Menopause**

Mean	Min	Max	OBS
1,741.2	0.0	2,011.0	816

- **da028_2 : The Age Started Menopause**

Mean	Min	Max	OBS
54.0	11.0	2,011.0	5,089

- **da029 : Y/N Diagnosed With A Prostate Illness**

	No	%
1 Yes	765	10.11
2 No	6,801	89.89
Total	7,566	100.00

- **da030_1 : The Year First Diagnosed With A Prostate Illness**

Mean	Min	Max	OBS
2,005.8	1,970.0	2,011.0	633

- **da030_2 : The Age First Diagnosed With A Prostate Illness**

	No	%
15	1	0.81
20	1	0.81
23	2	1.61
25	1	0.81
30	2	1.61
32	2	1.61
35	2	1.61
36	1	0.81
38	1	0.81
39	1	0.81
40	8	6.45
42	4	3.23
44	2	1.61
45	9	7.26
46	2	1.61
47	2	1.61
48	1	0.81
50	14	11.29
51	1	0.81
52	7	5.65
53	3	2.42
54	2	1.61
55	6	4.84
56	2	1.61
57	3	2.42
58	1	0.81
59	1	0.81
60	15	12.10
61	1	0.81
63	1	0.81
64	4	3.23
65	3	2.42
66	1	0.81
68	1	0.81
69	1	0.81
70	3	2.42

71	1	0.81
72	1	0.81
74	1	0.81
75	3	2.42
77	2	1.61
79	2	1.61
80	2	1.61
Total	124	100.00

- **da031 : Taking Mediation or Other Treatment for Your Prostate Illness**

	No	%
1 Yes	254	33.20
2 No	511	66.80
Total	765	100.00

- **da032 : Y/N Wearing Glasses or Corrective Lens**

	No	%
1 Yes	1,968	11.20
2 Legally blind	116	0.66
3 No	15,485	88.14
Total	17,569	100.00

- **da033 : Your Eyesight for Seeing Things at A Distance**

	No	%
1 Excellent	268	1.66
2 Very good	1,981	12.28
3 Good	4,017	24.90
4 Fair	6,296	39.03
5 Poor	3,570	22.13
Total	16,132	100.00

- **da034 : Your Eyesight for Seeing Things Up Close**

	No	%
1 Excellent	191	1.18
2 Very good	1,419	8.80
3 Good	3,862	23.95
4 Fair	6,907	42.83
5 Poor	3,748	23.24
Total	16,127	100.00

- **da035 : Cataract Surgery**

	No	%
1 Yes	329	1.89
2 No	17,117	98.11
Total	17,446	100.00

- **da036 : Cataract Surgery on Both Eyes or Just One**

	No	%
1 One eye only	201	59.47
2 Both eyes	137	40.53
Total	338	100.00

- **da037 : Glaucoma**

	No	%
1 Yes	168	0.96
2 No	17,257	99.04
Total	17,425	100.00

- **da038 : Wearing A Hearing Aid**

	No	%
1 Yes	98	0.56
2 No	17,470	99.44
Total	17,568	100.00

- **da039 : Hearing With A Hearing Aid**

	No	%
1 Excellent	253	1.44
2 Very good	2,451	13.96
3 Good	5,333	30.37
4 Fair	7,028	40.03
5 Poor	2,494	14.20
Total	17,559	100.00

- **da040 : Wearing Dentures,Fixed or Removable**

	No	%
1 Yes	1,516	8.63

2 No	16,052	91.37
Total	17,568	100.00

- **da041 : Currently Feel Any Body Pains**

	No	%
1 Yes	5,665	32.32
2 No	11,862	67.68
Total	17,527	100.00

- **da042s1 : Y/N the Head Pains**

	No	%
1 Head Headache	2,342	100.00
Total	2,342	100.00

- **da042s2 : Y/N the Shoulder Pains**

	No	%
2 Shoulder	2,107	100.00
Total	2,107	100.00

- **da042s3 : Y/N the Arm Pains**

	No	%
3 Arm	1,715	100.00
Total	1,715	100.00

- **da042s4 : Y/N the Wrist Pains**

	No	%
4 Wrist	1,094	100.00
Total	1,094	100.00

- **da042s5 : Y/N the Fingers Pains**

	No	%
5 Fingers	1,024	100.00
Total	1,024	100.00

- **da042s6 : Y/N the Chest Pains**

	No	%
6 Chest	1,112	100.00
Total	1,112	100.00

- **da042s7 : Y/N the Stomach Pains**

	No	%
7 Stomach Stomachache	1,504	100.00
Total	1,504	100.00

- **da042s8 : Y/N the Back Pains**

	No	%
8 Back	1,597	100.00
Total	1,597	100.00

- **da042s9 : Y/N the Waist Pains**

	No	%
9 Waist	3,379	100.00
Total	3,379	100.00

- **da042s10 : Y/N the Buttocks Pains**

	No	%
10 Buttocks	649	100.00
Total	649	100.00

- **da042s11 : Y/N the Leg Pains**

	No	%
11 Leg	2,554	100.00
Total	2,554	100.00

- **da042s12 : Y/N the Knees Pains**

	No	%
12 Knees	2,253	100.00

Total	2,253	100.00

- **da042s13 : Y/N the Ankle Pains**

	No	%
13 Ankle	997	100.00
Total	997	100.00

- **da042s14 : Y/N the Toes Pains**

	No	%
14 Toes	578	100.00
Total	578	100.00

- **da042s15 : Y/N the Neck Pains**

	No	%
15 Neck	1,179	100.00
Total	1,179	100.00

- **da043 : How Bad Is Your Pain**

	No	%
1 Mild	1,421	25.18
2 Moderate	2,054	36.39
3 Severe	2,169	38.43
Total	5,644	100.00

- **da044s1 : Y/N Been Diagnosed With Tuberculosis**

	No	%
1 Tuberculosis	113	100.00
Total	113	100.00

- **da044s2 : Y/N Been Diagnosed With Hepatitis B**

	No	%
2 hepatitis B	103	100.00
Total	103	100.00

- **da044s3 : Y/N Been Diagnosed With Malaria**

	No	%
3 Malaria	16	100.00
Total	16	100.00

- **da044s4 : Y/N Been Diagnosed With Influenza**

	No	%
4 Influenza	442	100.00
Total	442	100.00

- **da044s5 : Y/N Been Diagnosed With Rabies**

	No	%
5 Rabies	3	100.00
Total	3	100.00

- **da044s6 : Y/N Been Diagnosed With Schistosomiasis**

	No	%
6 Schistosomiasis	109	100.00
Total	109	100.00

- **da044s7 : Y/N Been Diagnosed With AIDS Patients and Infecious Ones**

	No	%
7 AIDS patients and infecious ones	1	100.00
Total	1	100.00

- **da044s8 : Y/N Been Diagnosed With Encephalitis B**

	No	%
8 Encephalitis B	4	100.00
Total	4	100.00

- **da044s9 : Y/N Been Diagnosed With Dysentery**

	No	%
9 Dysentery	157	100.00

Total	157	100.00

- **da044s10 : Y/N Been Diagnosed With Measles**

	No	%
10 Measles	36	100.00
Total	36	100.00

- **da044s11 : Y/N Been Diagnosed With Brucellosis**

	No	%
11 Brucellosis	7	100.00
Total	7	100.00

- **da044s12 : Y/N Been Diagnosed With Gonorrhea**

	No	%
12 Gonorrhea	1	100.00
Total	1	100.00

- **da044s13 : Y/N Been Diagnosed With Syphilis**

	No	%
13 Syphilis	4	100.00
Total	4	100.00

- **da044s14 : Y/N Been Diagnosed With Others**

	No	%
14 Others Pls specify:	135	100.00
Total	135	100.00

- **da044s15 : Y/N Been Diagnosed With None**

	No	%
15 none of these	16,573	100.00
Total	16,573	100.00

- **da045 : Any Other Medical Diseases**

	No	%
1 Yes	2,747	15.65
2 No	14,806	84.35
Total	17,553	100.00

- **da047 : Any Weigh Change**

	No	%
1 Yes, I only gained weight	534	3.04
2 Yes, I only lost weight	1,251	7.13
3 Yes, I gained and lost weight	109	0.62
4 Yes, I lost and gained weight	106	0.60
5 No	14,559	83.00
6 I dont Know	983	5.60
Total	17,542	100.00

- **da048 : Your Health During Childhood**

	No	%
1 Excellent	1,663	9.64
2 Very good	6,465	37.46
3 Good	4,949	28.68
4 Fair	2,991	17.33
5 Poor	1,190	6.90
Total	17,258	100.00

- **da049 : Average Hours for One Night Sleeping Time During the Past Month**

Mean	Min	Max	OBS
6.4	0.0	15.0	16,091

- **da050 : How Long Did You Take A Nap During the Past Month**

Mean	Min	Max	OBS
32.3	0.0	180.0	16,185

- **da051_1_ : Y/N Do Vigorous Activities At Least 10 Minutes Continuously**

	No	%

1 Yes	2,361	34.13
2 No	4,557	65.87
Total	6,918	100.00

- **da051_2_ : Y/N Do Moderate Physical Effort At Least 10 Minutes Continuously**

	No	%
1 Yes	3,884	56.18
2 No	3,030	43.82
Total	6,914	100.00

- **da051_3_ : Y/N Walking At Least 10 Minutes Continuously**

	No	%
1 Yes	5,495	79.65
2 No	1,404	20.35
Total	6,899	100.00

- **da052_1_ : Days Do Vigorous Activities At Least 10 Minutes Continuously**

	No	%
1	87	3.69
2	160	6.79
3	187	7.94
4	160	6.79
5	163	6.92
6	102	4.33
7	1,497	63.54
Total	2,356	100.00

- **da052_2_ : Days Do Moderate Physical Effort At Least 10 Minutes Continuously**

	No	%
1	87	2.25
2	160	4.13
3	218	5.63
4	153	3.95
5	208	5.37
6	119	3.07
7	2,929	75.61
Total	3,874	100.00

- **da052_3_ : Days Walking At Least 10 Minutes Continuously**

	No	%
1	68	1.24
2	97	1.77
3	162	2.96
4	110	2.01
5	157	2.87
6	72	1.32
7	4,802	87.82
Total	5,468	100.00

- **da053_1_ : 2 Hours Everyday Do Vigorous Activities At Least 10 Minutes Continuously**

	No	%
1 Less than 2 hours	416	17.57
More than 2 hours	1,951	82.43
Total	2,367	100.00

- **da053_2_ : 2 Hours Everyday Do Moderate Physical Effort At Least 10 Minutes Continuously**

	No	%
1 Less than 2 hours	1,493	38.56
More than 2 hours	2,379	61.44
Total	3,872	100.00

- **da053_3_ : 2 Hours Everyday Walking At Least 10 Minutes Continuously**

	No	%
1 Less than 2 hours	3,418	62.42
More than 2 hours	2,058	37.58
Total	5,476	100.00

- **da054_1_ : Minutes Everyday Do Vigorous Activities At Least 10 Minutes Continuously**

	No	%
1 Less than 30 minutes	110	26.32
More than 30 minutes	308	73.68
Total	418	100.00

- **da054_2_ : Minutes Everyday Do Moderate Physical Effort At Least 10 Minutes Continuously**

	No	%
1 Less than 30 minutes	326	21.73
More than 30 minutes	1,174	78.27
Total	1,500	100.00

- **da054_3_ : Minutes Everyday Walking At Least 10 Minutes Continuously**

	No	%
1 Less than 30 minutes	948	27.65
More than 30 minutes	2,481	72.35
Total	3,429	100.00

- **da055_1_ : 4 Hours Everyday Do Vigorous Activities At Least 10 Minutes Continuously**

	No	%
1 Less than 4 hours	485	24.88
More than 4 hours	1,464	75.12
Total	1,949	100.00

- **da055_2_ : 4 Hours Everyday Do Moderate Physical Effort At Least 10 Minutes Continuously**

	No	%
1 Less than 4 hours	925	38.88
More than 4 hours	1,454	61.12
Total	2,379	100.00

- **da055_3_ : 4 Hours Everyday Walking At Least 10 Minutes Continuously**

	No	%
1 Less than 4 hours	1,190	57.29
More than 4 hours	887	42.71
Total	2,077	100.00

- **da056s1 : Do Activities LM of Interacted With Friends**

	No	%
1 Interacted with friends	5,718	100.00

Total	5,718	100.00

- **da056s2 : Do Activities LM of Played Ma-jong/Cards/Chess or Went to Community Club**

	No	%
2 Played Ma-jong, etc, or went to community club	2,976	100.00
Total	2,976	100.00

- **da056s3 : Do Activities LM of Provided Help to People Who Lived Apart**

	No	%
3 Provided help to family, friends, or neighbors	1,102	100.00
Total	1,102	100.00

- **da056s4 : Do Activities LM of Went to Club**

	No	%
4 Went to a sport, social, or other kind of club	1,052	100.00
Total	1,052	100.00

- **da056s5 : Do Activities LM of Took Part In A Community-related Organization**

	No	%
5 Took part in a community-related organization	239	100.00
Total	239	100.00

- **da056s6 : Do Activities LM of Voluntary or Charity**

	No	%
6 Done voluntary or charity work	102	100.00
Total	102	100.00

- **da056s7 : Do Activities LM of Cared for A Sick or Disabled Adult Who Lived Apart**

	No	%
7 Cared for a sick or disabled adult	122	100.00
Total	122	100.00

- **da056s8 : Do Activities LM of Attended An Educational or Training Course**

	No	%
8 Attended an educational or training course	61	100.00
Total	61	100.00

- **da056s9 : Do Activities LM of Stock Investment**

	No	%
9 Stock investment	92	100.00
Total	92	100.00

- **da056s10 : Do Activities LM of Used the Internet**

	No	%
10 Used the Internet	452	100.00
Total	452	100.00

- **da056s11 : Do Activities LM of Other**

	No	%
11 Other	127	100.00
Total	127	100.00

- **da056s12 : Do Activities LM of None of These**

	No	%
12 None of these	8,097	100.00
Total	8,097	100.00

- **da057_1_ : How Often Do Activities LM of Interacted With Friends**

	No	%
1 Almost daily	2,771	48.45
2 Almost every week	1,280	22.38
3 Less often	1,668	29.17
Total	5,719	100.00

- **da057_2_ : How Often Do Activities LM of Played Ma-jong/Cards/Chess**

	No	%
1 Almost daily	776	26.00
2 Almost every week	1,000	33.50
3 Less often	1,209	40.50
Total	2,985	100.00

- **da057_3_ : How Often Do Activities LM of Provided Help to People Who Lived Apart**

	No	%
1 Almost daily	122	11.00
2 Almost every week	207	18.67
3 Less often	780	70.33
Total	1,109	100.00

- **da057_4_ : How Often Do Activities LM of Went to Club**

	No	%
1 Almost daily	688	65.40
2 Almost every week	211	20.06
3 Less often	153	14.54
Total	1,052	100.00

- **da057_5_ : How Often Do Activities LM of Took Part In A Community-related Organization**

	No	%
1 Almost daily	32	13.39
2 Almost every week	79	33.05
3 Less often	128	53.56
Total	239	100.00

- **da057_6_ : How Often Do Activities LM of Voluntary or Charity**

	No	%
1 Almost daily	5	4.90
2 Almost every week	17	16.67
3 Less often	80	78.43
Total	102	100.00

- **da057_7_ : How Often Do Activities LM of Cared for A Sick or Disabled Adult Who Lived Apart**

	No	%
1 Almost daily	29	23.39
2 Almost every week	36	29.03
3 Less often	59	47.58
Total	124	100.00

- **da057_8_ : How Often Do Activities LM of Attended An Educational or Training Course**

	No	%
1 Almost daily	5	8.20
2 Almost every week	12	19.67
3 Less often	44	72.13
Total	61	100.00

- **da057_9_ : How Often Do Activities LM of Stock Investment**

	No	%
1 Almost daily	49	53.85
2 Almost every week	12	13.19
3 Less often	30	32.97
Total	91	100.00

- **da057_10_ : How Often Do Activities LM of Used the Internet**

	No	%
1 Almost daily	313	69.40
2 Almost every week	71	15.74
3 Less often	67	14.86
Total	451	100.00

- **da057_11_ : How Often Do Activities LM of Other**

	No	%
1 Almost daily	1	100.00
Total	1	100.00

- **da058 : The Number of Times for Eating**

	No	%
1 More than 4 meals per day	57	0.35

2 4 meals per day	216	1.33
3 3 meals per day	13,783	84.95
4 2 meals per day	2,142	13.20
5 1 meal per day	23	0.14
6 Less than 1 meal per day	3	0.02
Total	16,224	100.00

- **da059 : Smoke or Not**

	No	%
1 Yes	6,931	39.47
2 No	10,628	60.53
Total	17,559	100.00

- **da060 : Product Type for Smoking**

	No	%
1 Smoking a pipe	576	9.09
2 Smoking self-rolled cigarettes	803	12.67
3 Filtered cigarette	4,501	70.99
4 Unfiltered cigarette	201	3.17
5 Cigar	18	0.28
6 Water cigarettes	241	3.80
Total	6,340	100.00

- **da061 : Still Smoking or Not**

	No	%
1 Still have	4,871	77.18
2 Quit	1,440	22.82
Total	6,311	100.00

- **da062_1 : The Age You Totally Quit Smoking**

	No	%
13	3	0.61
14	3	0.61
15	7	1.43
16	6	1.23
17	3	0.61
18	11	2.25
19	4	0.82
20	31	6.34
21	6	1.23
22	2	0.41

23	5	1.02
24	3	0.61
25	12	2.45
26	1	0.20
27	2	0.41
28	7	1.43
29	3	0.61
30	32	6.54
31	1	0.20
32	5	1.02
33	3	0.61
34	2	0.41
35	7	1.43
36	6	1.23
37	4	0.82
38	5	1.02
39	2	0.41
40	34	6.95
41	4	0.82
42	8	1.64
43	3	0.61
44	4	0.82
45	23	4.70
46	10	2.04
47	5	1.02
48	6	1.23
49	6	1.23
50	41	8.38
51	3	0.61
52	5	1.02
53	7	1.43
54	6	1.23
55	16	3.27
56	11	2.25
57	4	0.82
58	9	1.84
59	7	1.43
60	39	7.98
61	2	0.41
62	11	2.25
63	3	0.61
64	2	0.41
65	5	1.02
66	3	0.61
67	4	0.82
68	4	0.82
69	3	0.61
70	11	2.25
71	1	0.20
72	1	0.20
73	5	1.02
75	3	0.61

76	1	0.20
78	1	0.20
80	2	0.41
Total	489	100.00

- **da062_2 : The Year You Totally Quit Smoking**

Mean	Min	Max	OBS
2,003.9	1,950.0	2,011.0	927

- **da062s1 : The Age**

	No	%
1 Age	544	100.00
Total	544	100.00

- **da062s2 : The Year**

	No	%
2 Year	935	100.00
Total	935	100.00

- **da063 : The Number of Cigarettes You Consume**

Mean	Min	Max	OBS
19.0	1.0	120.0	4,656

- **da064 : Cigarettes Price**

Mean	Min	Max	OBS
4.6	0.0	150.0	4,628

- **da065_1 : The Age Beginning Smoking**

	No	%
1	2	0.03
2	1	0.02
3	1	0.02
4	1	0.02
5	7	0.12
6	8	0.14

7	13	0.22
8	37	0.64
9	23	0.39
10	51	0.88
11	17	0.29
12	78	1.34
13	84	1.44
14	95	1.63
15	321	5.51
16	356	6.11
17	361	6.20
18	586	10.06
19	160	2.75
20	1,283	22.03
21	145	2.49
22	203	3.48
23	182	3.12
24	166	2.85
25	414	7.11
26	92	1.58
27	79	1.36
28	61	1.05
29	12	0.21
30	461	7.91
31	9	0.15
32	16	0.27
33	24	0.41
34	11	0.19
35	81	1.39
36	21	0.36
37	12	0.21
38	13	0.22
39	6	0.10
40	159	2.73
41	4	0.07
42	10	0.17
43	6	0.10
45	34	0.58
46	4	0.07
47	4	0.07
48	4	0.07
49	3	0.05
50	55	0.94
52	5	0.09
53	1	0.02
54	4	0.07
55	7	0.12
56	4	0.07
57	2	0.03
58	1	0.02
59	1	0.02
60	12	0.21

65	1	0.02
66	1	0.02
67	2	0.03
68	1	0.02
70	3	0.05
72	1	0.02
74	1	0.02
76	1	0.02
77	1	0.02
Total	5,825	100.00

- **da065_2 : The Year Beginning Smoking**

Mean	Min	Max	OBS
1,982.3	1,950.0	2,011.0	387

- **da065s1 : The Age**

	No	%
1 Age	5,843	100.00
Total	5,843	100.00

- **da065s2 : The Year**

	No	%
2 Year	388	100.00
Total	388	100.00

- **da066 : How Soon After You Wake Up You Smoke**

	No	%
1 Within 5 minutes	2,065	33.18
2 Within 6-30 minutes	1,042	16.74
3 Within 31-60 minutes	480	7.71
4 More than 1 hour	2,636	42.36
Total	6,223	100.00

- **da067 : Did You Drink Any Alcoholic Beverages Last Year**

	No	%
1 Drink more than once a month	4,383	24.97
2 Drink but less than once a month	1,384	7.89
3 None of these	11,785	67.14

Total	17,552	100.00

- **da068s1 : Did You Drink Liqor**

	No	%
1 Liqor, including white liquor, whisky, and others	3,256	100.00
Total	3,256	100.00

- **da068s2 : Did You Drink Beer**

	No	%
2 Beer	1,773	100.00
Total	1,773	100.00

- **da068s3 : Did You Drink Wine**

	No	%
3 Wine or rice wine	618	100.00
Total	618	100.00

- **da069 : Did You Drink Alcoholic Beverages Ever**

	No	%
1 I never had a drink.	10,718	81.08
2 I used to drink less than once a month.	1,424	10.77
3 I used to drink more than once a month.	1,077	8.15
Total	13,219	100.00

- **da070 : Year or Age**

	No	%
1 Age	699	70.82
2 Year	288	29.18
Total	987	100.00

- **da070_1 : The Year for Quiting Drinking**

Mean	Min	Max	OBS
2,004.4	1,950.0	2,011.0	685

- **da070_2 : The Age for Quiting Drinking**

	No	%
14	1	0.36
15	1	0.36
16	1	0.36
17	6	2.14
18	2	0.71
19	2	0.71
20	22	7.83
21	2	0.71
22	1	0.36
23	2	0.71
25	5	1.78
26	2	0.71
27	2	0.71
28	2	0.71
29	2	0.71
30	25	8.90
33	2	0.71
34	1	0.36
35	2	0.71
36	1	0.36
37	4	1.42
38	1	0.36
40	20	7.12
41	3	1.07
42	3	1.07
43	2	0.71
44	2	0.71
45	18	6.41
46	1	0.36
47	1	0.36
48	3	1.07
49	3	1.07
50	36	12.81
51	3	1.07
52	7	2.49
53	4	1.42
54	3	1.07
55	13	4.63
56	3	1.07
57	5	1.78
58	8	2.85
59	2	0.71
60	18	6.41
61	3	1.07
63	2	0.71
64	1	0.36
65	3	1.07
66	1	0.36
67	1	0.36

68	3	1.07
69	2	0.71
70	8	2.85
71	2	0.71
72	1	0.36
73	1	0.36
74	1	0.36
78	2	0.71
80	1	0.36
88	1	0.36
100	1	0.36
Total	281	100.00

- **da071 : Year or Age**

	No	%
1 Age	585	11.29
2 Year	4,595	88.71
Total	5,180	100.00

- **da071_1 : The Year for Starting Drinking**

Mean	Min	Max	OBS
1,990.5	1,919.0	2,011.0	589

- **da071_2 : The Age for Starting Drinking**

	No	%
3	1	0.02
4	2	0.04
5	24	0.52
6	11	0.24
7	18	0.39
8	25	0.54
9	6	0.13
10	48	1.04
11	4	0.09
12	43	0.94
13	21	0.46
14	30	0.65
15	180	3.92
16	176	3.83
17	188	4.09
18	351	7.64
19	114	2.48
20	1,169	25.44
21	99	2.15

22	139	3.02
23	139	3.02
24	148	3.22
25	340	7.40
26	85	1.85
27	71	1.54
28	53	1.15
29	3	0.07
30	504	10.97
31	11	0.24
32	24	0.52
33	8	0.17
34	14	0.30
35	84	1.83
36	20	0.44
37	19	0.41
38	9	0.20
39	6	0.13
40	164	3.57
41	3	0.07
42	7	0.15
43	4	0.09
44	8	0.17
45	46	1.00
46	5	0.11
47	5	0.11
48	2	0.04
49	2	0.04
50	78	1.70
51	1	0.02
52	7	0.15
53	3	0.07
54	4	0.09
55	11	0.24
56	3	0.07
57	7	0.15
58	5	0.11
60	26	0.57
64	2	0.04
65	1	0.02
67	1	0.02
68	1	0.02
69	1	0.02
70	9	0.20
72	2	0.04
77	1	0.02
Total	4,596	100.00

- **da072 : The Frequency for Drink Liquor**

	No	%

1 Once a month	180	7.04
2 2-3 times a month	322	12.59
3 Once a week	197	7.70
4 2-3 times a week	312	12.20
5 4-6 times a week	113	4.42
6 Once a day	782	30.58
7 Twice a day	485	18.97
8 More than twice a day	166	6.49
Total	2,557	100.00

- **da073 : How Many Liquor Did You Drink LY**

Mean	Min	Max	OBS
2.5	0.0	73.0	2,552

- **da074 : How Many Times Per Month Did You Drink Beer LY**

	No	%
1 Once a month	175	13.03
2 2-3 times a month	256	19.06
3 Once a week	165	12.29
4 2-3 times a week	227	16.90
5 4-6 times a week	90	6.70
6 Once a day	274	20.40
7 Twice a day	120	8.94
8 More than twice a day	36	2.68
Total	1,343	100.00

- **da075 : Bottles or Mugs**

	No	%
1 Bottles	1,161	86.06
2 Mugs	188	13.94
Total	1,349	100.00

- **da075_1 : How Many Bottles of Beer Did You Drink LY**

Mean	Min	Max	OBS
2.1	0.0	240.0	1,162

- **da075_2 : How Many Mugs of Beer Did You Drink LY**

	No	%

1	114	60.64
2	65	34.57
3	3	1.60
4	1	0.53
5	4	2.13
6	1	0.53
Total	188	100.00

- **da076 : How Often Did You Drink Wine Per Month LY**

	No	%
1 Once a month	13	31.71
2 2-3 times a month	4	9.76
3 Once a week	3	7.32
4 2-3 times a week	2	4.88
5 4-6 times a week	2	4.88
6 Once a day	9	21.95
7 Twice a day	7	17.07
8 More than twice a day	1	2.44
Total	41	100.00

- **da077 : How Many Liang of Wine Did You Drink the Last Time You Drank LY**

Mean	Min	Max	OBS
6.2	0.0	100.0	42

- **da078 : Taking A Drink First Thing In the Morning Ever LY**

	No	%
1 Yes	171	4.94
2 No	3,291	95.06
Total	3,462	100.00

- **da079 : Self Comment of Your Health By Comparing With Others**

	No	%
1 Very good	840	6.15
2 Good	2,406	17.63
3 Fair	6,942	50.86
4 Poor	2,830	20.73
5 Very poor	631	4.62
Total	13,649	100.00

- **da080 : Self Comment of Your Health**

	No	%
1 Excellent	43	1.10
2 Very good	398	10.20
3 Good	847	21.71
4 Fair	1,888	48.40
5 Poor	725	18.58
Total	3,901	100.00

- **da081 : The Probability of Self Expected Age**

	No	%
1 Almost impossible	1,199	8.50
2 Not very likely	2,871	20.35
3 Maybe	5,139	36.43
4 Very likely	2,329	16.51
5 Almost certain	2,568	18.21
Total	14,106	100.00

- **db001 : Have Difficulty Running or Jogging About 1 Km**

	No	%
1 No, I dont have any difficulty	8,282	48.04
2 I have difficulty but can still do it.	1,816	10.53
3 Yes, I have difficulty and need help.	444	2.58
4 I can not do it.	6,699	38.86
Total	17,241	100.00

- **db002 : Have Difficulty Walking 1 KM**

	No	%
1 No, I dont have any difficulty	6,526	70.38
2 I have difficulty but can still do it.	1,244	13.42
3 Yes, I have difficulty and need help.	232	2.50
4 I can not do it.	1,270	13.70
Total	9,272	100.00

- **db003 : Have Difficulty Walking 100 Metres**

	No	%
1 No, I dont have any difficulty	1,228	82.09
2 I have difficulty but can still do it.	217	14.51
3 Yes, I have difficulty and need help.	44	2.94

4 I can not do it.	7	0.47
Total	1,496	100.00

- **db004 : Have Difficulty Getting Up From A Chair After Sitting for Long Periods**

	No	%
1 No, I dont have any difficulty	12,781	72.90
2 I have difficulty but can still do it.	4,102	23.40
3 Yes, I have difficulty and need help.	382	2.18
4 I can not do it.	268	1.53
Total	17,533	100.00

- **db005 : Have Difficulty Climbing Several Flights of Stairs Without Resting**

	No	%
1 No, I dont have any difficulty	10,458	60.05
2 I have difficulty but can still do it.	4,307	24.73
3 Yes, I have difficulty and need help.	586	3.36
4 I can not do it.	2,065	11.86
Total	17,416	100.00

- **db006 : Have Difficulty Stooping, Kneeling, or Crouching**

	No	%
1 No, I dont have any difficulty	12,388	70.67
2 I have difficulty but can still do it.	3,478	19.84
3 Yes, I have difficulty and need help.	505	2.88
4 I can not do it.	1,158	6.61
Total	17,529	100.00

- **db007 : Have Difficulty Reaching or Extending Your Arms Above Shoulder Level**

	No	%
1 No, I dont have any difficulty	15,717	89.62
2 I have difficulty but can still do it.	910	5.19
3 Yes, I have difficulty and need help.	168	0.96
4 I can not do it.	743	4.24
Total	17,538	100.00

- **db008 : Have Difficulty Lifting or Carrying Weights Over 10 Jin**

	No	%
1 No, I dont have any difficulty	15,438	88.04
2 I have difficulty but can still do it.	803	4.58
3 Yes, I have difficulty and need help.	193	1.10
4 I can not do it.	1,102	6.28
Total	17,536	100.00

- **db009 : Have Difficulty Picking Up A Small Coin From A Table**

	No	%
1 No, I dont have any difficulty	16,812	95.89
2 I have difficulty but can still do it.	336	1.92
3 Yes, I have difficulty and need help.	70	0.40
4 I can not do it.	315	1.80
Total	17,533	100.00

- **db010 : Have Any Difficulty With Dressing Because of Health And Memory Problem**

	No	%
1 No, I dont have any difficulty	10,568	92.10
2 I have difficulty but can still do it.	601	5.24
3 Yes, I have difficulty and need help.	167	1.46
4 I can not do it.	138	1.20
Total	11,474	100.00

- **db011 : Have Any Difficulty With Bathing or Showering**

	No	%
1 No, I dont have any difficulty	10,282	89.70
2 I have difficulty but can still do it.	585	5.10
3 Yes, I have difficulty and need help.	335	2.92
4 I can not do it.	261	2.28
Total	11,463	100.00

- **db012 : Have Any Difficulty With Eating Because of Health And Memory Problem**

	No	%
1 No, I dont have any difficulty	10,945	95.37
2 I have difficulty but can still do it.	325	2.83
3 Yes, I have difficulty and need help.	100	0.87
4 I can not do it.	106	0.92

Total	11,476	100.00

- **db013 : Have Any Difficulty With Getting Out of Bed and Walking Across A Room**

	No	%
1 No, I dont have any difficulty	10,501	91.52
2 I have difficulty but can still do it.	719	6.27
3 Yes, I have difficulty and need help.	139	1.21
4 I can not do it.	115	1.00
Total	11,474	100.00

- **db014 : Have Any Difficulty With Using the Toilet, Getting Up and Down**

	No	%
1 No, I dont have any difficulty	9,461	82.46
2 I have difficulty but can still do it.	1,491	12.99
3 Yes, I have difficulty and need help.	273	2.38
4 I can not do it.	249	2.17
Total	11,474	100.00

- **db015 : Have Any Difficulty With Controlling Urination and Defecation**

	No	%
1 No, I dont have any difficulty	10,713	93.40
2 I have difficulty but can still do it.	490	4.27
3 Yes, I have difficulty and need help.	94	0.82
4 I can not do it.	173	1.51
Total	11,470	100.00

- **db016 : Have Any Difficulty With Doing Household Chores**

	No	%
1 No, I dont have any difficulty	15,856	90.42
2 I have difficulty but can still do it.	801	4.57
3 Yes, I have difficulty and need help.	234	1.33
4 I can not do it.	644	3.67
Total	17,535	100.00

- **db017 : Have Any Difficulty With Preparing Hot Meals**

	No	%

1 No, I dont have any difficulty	15,939	90.89
2 I have difficulty but can still do it.	638	3.64
3 Yes, I have difficulty and need help.	201	1.15
4 I can not do it.	758	4.32
Total	17,536	100.00

- **db018 : Have Any Difficulty With Shopping Because of Health And Memory Problem**

	No	%
1 No, I dont have any difficulty	15,886	90.66
2 I have difficulty but can still do it.	639	3.65
3 Yes, I have difficulty and need help.	243	1.39
4 I can not do it.	754	4.30
Total	17,522	100.00

- **db019 : Have Any Difficulty With Managing Assets Because of Health And Memory Problem**

	No	%
1 No, I dont have any difficulty	15,254	87.16
2 I have difficulty but can still do it.	749	4.28
3 Yes, I have difficulty and need help.	370	2.11
4 I can not do it.	1,129	6.45
Total	17,502	100.00

- **db020 : Have Any Difficulty With Taking Medications Because of Health And Memory Problem**

	No	%
1 No, I dont have any difficulty	16,298	93.02
2 I have difficulty but can still do it.	561	3.20
3 Yes, I have difficulty and need help.	386	2.20
4 I can not do it.	276	1.58
Total	17,521	100.00

- **db021s1 : Do You Use Walking Stick**

	No	%
1 Walking stick	634	100.00
Total	634	100.00

- **db021s2 : Do You Use Travel Device**

	No	%
2 Travel device	12	100.00
Total	12	100.00

- **db021s3 : Do You Use Manual Wheelchair**

	No	%
3 Manual wheelchair	71	100.00
Total	71	100.00

- **db021s4 : Do You Use Electric Wheelchair**

	No	%
4 Electric Wheelchair	5	100.00
Total	5	100.00

- **db021s5 : Do Not Use Any Auxiliay**

	No	%
5 None	1,917	100.00
Total	1,917	100.00

- **db022_a_6_ : Y/N Children Him/herself Taking the Time**

	No	%
1 Yes	429	95.76
2 No	19	4.24
Total	448	100.00

- **db022_a_7_ : Y/N Children Him/herself Taking the Time**

	No	%
1 Yes	100	89.29
2 No	12	10.71
Total	112	100.00

- **db022_a_8_ : Y/N Children Him/herself Taking the Time**

	No	%

1 Yes	64	95.52
2 No	3	4.48
Total	67	100.00

- **db022_a_9_ : Y/N Children Him/herself Taking the Time**

	No	%
1 Yes	32	88.89
2 No	4	11.11
Total	36	100.00

- **db022_a_10_ : Y/N Children Him/herself Taking the Time**

	No	%
1 Yes	17	94.44
2 No	1	5.56
Total	18	100.00

- **db022_a_11_ : Y/N Children Him/herself Taking the Time**

	No	%
1 Yes	8	88.89
2 No	1	11.11
Total	9	100.00

- **db022_a_12_ : Y/N Children Him/herself Taking the Time**

	No	%
1 Yes	1	50.00
2 No	1	50.00
Total	2	100.00

- **db022_a_13_ : Y/N Children Him/herself Taking the Time**

	No	%
1 Yes	1	100.00
Total	1	100.00

- **db022_a_34_ : Y/N Children's Spouse Him/herself Taking the Time**

	No	%
1 Yes	277	96.52
2 No	10	3.48
Total	287	100.00

- **db022s1 : Spouse Helps You Often With Functional Difficulties**

	No	%
1 Spouse	1,488	100.00
Total	1,488	100.00

- **db022s2 : Mother Helps You Often With Functional Difficulties**

	No	%
2 Mother	7	100.00
Total	7	100.00

- **db022s3 : Father Helps You Often With Functional Difficulties**

No Observations

- **db022s4 : Mother-in-law Helps You Often With Functional Difficulties**

	No	%
4 Mother-in-law	3	100.00
Total	3	100.00

- **db022s5 : Father-in-law Helps You Often With Functional Difficulties**

	No	%
5 Father-in-law	2	100.00
Total	2	100.00

- **db022s6 : Children Helps You Often With Functional Difficulties**

	No	%
6 HMemberChildren[1]	433	100.00
Total	433	100.00

- **db022s7 : Children Helps You Often With Functional Difficulties**

	No	%
7 HMemberChildren[2]	107	100.00
Total	107	100.00

- **db022s8 : Children Helps You Often With Functional Difficulties**

	No	%
8 HMemberChildren[3]	61	100.00
Total	61	100.00

- **db022s9 : Children Helps You Often With Functional Difficulties**

	No	%
9 HMemberChildren[4]	35	100.00
Total	35	100.00

- **db022s10 : Children Helps You Often With Functional Difficulties**

	No	%
10 HMemberChildren[5]	15	100.00
Total	15	100.00

- **db022s11 : Children Helps You Often With Functional Difficulties**

	No	%
11 HMemberChildren[6]	9	100.00
Total	9	100.00

- **db022s12 : Children Helps You Often With Functional Difficulties**

	No	%
12 HMemberChildren[7]	2	100.00
Total	2	100.00

- **db022s13 : Children Helps You Often With Functional Difficulties**

No Observations

- **db022s14 : Children Helps You Often With Functional Difficulties**

No Observations

- **db022s15 : Children Helps You Often With Functional Difficulties**

No Observations

- **db022s16 : Children Helps You Often With Functional Difficulties**

No Observations

- **db022s17 : Children Helps You Often With Functional Difficulties**

No Observations

- **db022s18 : Children Helps You Often With Functional Difficulties**

No Observations

- **db022s19 : Children Helps You Often With Functional Difficulties**

No Observations

- **db022s20 : Children Helps You Often With Functional Difficulties**

No Observations

- **db022s21 : Children Helps You Often With Functional Difficulties**

No Observations

- **db022s22 : Children Helps You Often With Functional Difficulties**

No Observations

- **db022s23 : Children Helps You Often With Functional Difficulties**

No Observations

- **db022s24 : Children Helps You Often With Functional Difficulties**

No Observations

- **db022s25 : Children Helps You Often With Functional Difficulties**

No Observations

- **db022s26 : Children Helps You Often With Functional Difficulties**

No Observations

- **db022s27 : Children Helps You Often With Functional Difficulties**

No Observations

- **db022s28 : Children Helps You Often With Functional Difficulties**

No Observations

- **db022s29 : Children Helps You Often With Functional Difficulties**

No Observations

- **db022s30 : Children Helps You Often With Functional Difficulties**

No Observations

- **db022s31 : Sibling Helps You Often With Functional Difficulties**

	No	%
31 Sibling	29	100.00
Total	29	100.00

- **db022s32 : Sibling of Spouse Helps You Often With Functional Difficulties**

	No	%
32 Sibling of spouse	6	100.00
Total	6	100.00

- **db022s33 : Brother-in-law,Sister in law Helps You Often With Functional Difficulties**

	No	%
33 Brother-in-law, sister-in-law	4	100.00
Total	4	100.00

- **db022s34 : Children's Spouse Helps You Often With Functional Difficulties**

	No	%
34 Spouse of child	278	100.00
Total	278	100.00

- **db022s35 : Grandchild Helps You Often With Functional Difficulties**

	No	%
35 Grandchild	90	100.00

Total	90	100.00

- **db022s36 : Other Relative Helps You Often With Functional Difficulties**

	No	%
36 Other relative	29	100.00
Total	29	100.00

- **db022s37 : Paid Helper Helps You Often With Functional Difficulties**

	No	%
37 Paid helpersuch as nanny	20	100.00
Total	20	100.00

- **db022s38 : Volunteer Helps You Often With Functional Difficulties**

No Observations

- **db022s39 : Other Helps You Often With Functional Difficulties**

	No	%
39 Other	46	100.00
Total	46	100.00

- **db022s40 : No One Helps You Often With Functional Difficulties**

	No	%
40 No one helped	367	100.00
Total	367	100.00

- **db023_1_ : How Many Days Did Spouse Help You LM**

	No	%
1	71	4.90
2	23	1.59
3	31	2.14
4	15	1.04
5	26	1.80
6	4	0.28
7	62	4.28

8	8	0.55
9	1	0.07
10	65	4.49
11	1	0.07
12	1	0.07
13	2	0.14
14	1	0.07
15	42	2.90
16	1	0.07
17	2	0.14
20	40	2.76
23	6	0.41
24	1	0.07
25	8	0.55
26	5	0.35
27	1	0.07
28	2	0.14
29	1	0.07
30	1,009	69.68
31	19	1.31
Total	1,448	100.00

- **db023_2_ : How Many Days Did Mother Help You LM**

	No	%
30	7	100.00
Total	7	100.00

- **db023_4_ : How Many Days Did Mother-in-law Help You LM**

	No	%
10	1	33.33
30	2	66.67
Total	3	100.00

- **db023_5_ : How Many Days Did Father-in-law Help You LM**

	No	%
10	1	50.00
30	1	50.00
Total	2	100.00

- **db023_6_ : How Many Days Did Children Help You LM**

	No	%

1	26	6.27
2	13	3.13
3	9	2.17
4	4	0.96
5	12	2.89
6	8	1.93
7	18	4.34
8	5	1.20
10	39	9.40
11	1	0.24
12	2	0.48
13	1	0.24
15	19	4.58
16	1	0.24
18	1	0.24
20	26	6.27
23	1	0.24
25	7	1.69
26	2	0.48
28	1	0.24
30	214	51.57
31	5	1.20
Total	415	100.00

- **db023_7_ : How Many Days Did Children Help You LM**

	No	%
1	11	11.00
2	10	10.00
3	2	2.00
4	7	7.00
5	4	4.00
6	1	1.00
7	6	6.00
10	12	12.00
13	1	1.00
15	2	2.00
18	1	1.00
20	8	8.00
28	1	1.00
30	34	34.00
Total	100	100.00

- **db023_8_ : How Many Days Did Children Help You LM**

	No	%
1	7	11.29
2	6	9.68
3	4	6.45

4	1	1.61
5	2	3.23
10	9	14.52
15	4	6.45
20	4	6.45
25	1	1.61
30	24	38.71
Total	62	100.00

- **db023_9_ : How Many Days Did Children Help You LM**

	No	%
1	5	15.15
2	4	12.12
3	4	12.12
10	2	6.06
15	1	3.03
20	1	3.03
24	1	3.03
30	15	45.45
Total	33	100.00

- **db023_10_ : How Many Days Did Children Help You LM**

	No	%
1	3	20.00
5	1	6.67
8	1	6.67
10	2	13.33
12	1	6.67
15	1	6.67
30	6	40.00
Total	15	100.00

- **db023_11_ : How Many Days Did Children Help You LM**

	No	%
2	3	33.33
3	1	11.11
5	1	11.11
10	1	11.11
15	1	11.11
30	2	22.22
Total	9	100.00

- **db023_12_ : How Many Days Did Children Help You LM**

	No	%
10	1	50.00
30	1	50.00
Total	2	100.00

- **db023_31_ : How Many Days Did Sibling Help You LM**

	No	%
1	3	11.11
2	2	7.41
3	3	11.11
4	1	3.70
6	1	3.70
14	1	3.70
15	1	3.70
30	13	48.15
31	2	7.41
Total	27	100.00

- **db023_32_ : How Many Days Did Sibling of Spouse Help You LM**

	No	%
2	2	33.33
3	1	16.67
10	1	16.67
30	2	33.33
Total	6	100.00

- **db023_33_ : How Many Days Did Brother-in-law,Sister in law Help You LM**

	No	%
2	1	25.00
3	1	25.00
30	1	25.00
31	1	25.00
Total	4	100.00

- **db023_34_ : How Many Days Did Children's Spouse Help You LM**

	No	%
1	8	2.94
2	3	1.10

3	10	3.68
4	4	1.47
5	8	2.94
6	2	0.74
7	7	2.57
8	3	1.10
10	12	4.41
13	1	0.37
14	1	0.37
15	13	4.78
20	16	5.88
25	1	0.37
30	182	66.91
31	1	0.37
Total	272	100.00

- **db023_35_ : How Many Days Did Grandchild Help You LM**

	No	%
1	2	2.20
2	8	8.79
3	6	6.59
4	4	4.40
5	3	3.30
6	2	2.20
7	1	1.10
8	3	3.30
10	6	6.59
13	1	1.10
14	2	2.20
15	5	5.49
20	2	2.20
23	2	2.20
28	1	1.10
30	43	47.25
Total	91	100.00

- **db023_36_ : How Many Days Did Other Relative Help You LM**

	No	%
1	5	17.86
4	1	3.57
5	5	17.86
6	1	3.57
7	1	3.57
8	1	3.57
10	2	7.14
15	3	10.71
30	8	28.57

31	1	3.57
Total	28	100.00

- **db023_37_ : How Many Days Did Paid Helper Help You LM**

	No	%
4	1	5.00
20	2	10.00
28	1	5.00
30	16	80.00
Total	20	100.00

- **db023_38_ : How Many Days Did Volunteer Help You LM**

	No	%
1	1	100.00
Total	1	100.00

- **db023_39_ : How Many Days Did Other Help You LM**

	No	%
1	10	23.81
3	4	9.52
4	1	2.38
5	1	2.38
6	3	7.14
7	1	2.38
10	3	7.14
15	2	4.76
18	1	2.38
20	1	2.38
25	1	2.38
30	14	33.33
Total	42	100.00

- **db024_1_ : How Many Hours Per Day Did Spouse Help You LM**

	No	%
1	463	32.72
2	219	15.48
3	189	13.36
4	82	5.80
5	63	4.45
6	62	4.38
7	13	0.92

8	60	4.24
9	5	0.35
10	65	4.59
11	1	0.07
12	72	5.09
14	5	0.35
15	6	0.42
16	3	0.21
17	1	0.07
18	2	0.14
20	16	1.13
24	88	6.22
Total	1,415	100.00

- **db024_2_ : How Many Hours Per Day Did Mother Help You LM**

	No	%
1	1	14.29
3	2	28.57
5	2	28.57
12	1	14.29
24	1	14.29
Total	7	100.00

- **db024_4_ : How Many Hours Per Day Did Mother-in-law Help You LM**

	No	%
1	1	50.00
4	1	50.00
Total	2	100.00

- **db024_5_ : How Many Hours Per Day Did Father-in-law Help You LM**

	No	%
1	2	100.00
Total	2	100.00

- **db024_6_ : How Many Hours Per Day Did Children Help You LM**

	No	%
1	135	33.58
2	90	22.39
3	54	13.43
4	27	6.72
5	14	3.48

6	13	3.23
7	5	1.24
8	14	3.48
9	1	0.25
10	5	1.24
12	16	3.98
15	1	0.25
16	4	1.00
20	2	0.50
24	21	5.22
Total	402	100.00

- **db024_7_ : How Many Hours Per Day Did Children Help You LM**

	No	%
1	34	33.66
2	16	15.84
3	16	15.84
4	11	10.89
5	7	6.93
6	4	3.96
7	1	0.99
8	4	3.96
10	3	2.97
12	3	2.97
16	1	0.99
24	1	0.99
Total	101	100.00

- **db024_8_ : How Many Hours Per Day Did Children Help You LM**

	No	%
1	17	28.81
2	8	13.56
3	14	23.73
4	4	6.78
5	3	5.08
6	3	5.08
8	4	6.78
9	1	1.69
10	2	3.39
12	1	1.69
24	2	3.39
Total	59	100.00

- **db024_9_ : How Many Hours Per Day Did Children Help You LM**

	No	%
1	9	27.27
2	3	9.09
3	7	21.21
4	1	3.03
5	3	9.09
8	2	6.06
10	1	3.03
12	4	12.12
18	1	3.03
24	2	6.06
Total	33	100.00

- **db024_10_ : How Many Hours Per Day Did Children Help You LM**

	No	%
1	5	33.33
2	3	20.00
3	2	13.33
5	1	6.67
6	3	20.00
8	1	6.67
Total	15	100.00

- **db024_11_ : How Many Hours Per Day Did Children Help You LM**

	No	%
1	2	22.22
2	2	22.22
4	1	11.11
6	2	22.22
12	1	11.11
24	1	11.11
Total	9	100.00

- **db024_12_ : How Many Hours Per Day Did Children Help You LM**

	No	%
1	1	50.00
2	1	50.00
Total	2	100.00

- **db024_31_ : How Many Hours Per Day Did Sibling Help You LM**

	No	%

1	5	20.00
2	4	16.00
3	4	16.00
5	2	8.00
8	4	16.00
10	3	12.00
12	3	12.00
Total	25	100.00

- **db024_32_ : How Many Hours Per Day Did Sibling of Spouse Help You LM**

	No	%
2	4	66.67
7	1	16.67
12	1	16.67
Total	6	100.00

- **db024_33_ : How Many Hours Per Day Did Brother-in-law,Sister in law Help You LM**

	No	%
1	1	25.00
2	1	25.00
8	1	25.00
10	1	25.00
Total	4	100.00

- **db024_34_ : How Many Hours Per Day Did Children's Spouse Help You LM**

	No	%
1	72	27.69
2	49	18.85
3	54	20.77
4	21	8.08
5	14	5.38
6	7	2.69
7	1	0.38
8	17	6.54
9	1	0.38
10	7	2.69
12	7	2.69
15	1	0.38
16	1	0.38
18	1	0.38

24	7	2.69
Total	260	100.00

- **db024_35_ : How Many Hours Per Day Did Grandchild Help You LM**

	No	%
1	36	40.91
2	22	25.00
3	6	6.82
4	6	6.82
5	2	2.27
6	3	3.41
7	1	1.14
8	2	2.27
10	2	2.27
12	6	6.82
24	2	2.27
Total	88	100.00

- **db024_36_ : How Many Hours Per Day Did Other Relative Help You LM**

	No	%
1	8	30.77
2	4	15.38
3	2	7.69
4	6	23.08
5	3	11.54
7	1	3.85
8	1	3.85
20	1	3.85
Total	26	100.00

- **db024_37_ : How Many Hours Per Day Did Paid Helper Help You LM**

	No	%
3	2	10.00
4	2	10.00
5	1	5.00
8	4	20.00
10	3	15.00
15	1	5.00
24	7	35.00
Total	20	100.00

- **db024_39_ : How Many Hours Per Day Did Other Help You LM**

	No	%
1	21	48.84
2	5	11.63
3	5	11.63
4	2	4.65
5	2	4.65
6	1	2.33
8	3	6.98
10	1	2.33
11	1	2.33
12	1	2.33
24	1	2.33
Total	43	100.00

- **db025_1_ : Is Spouse Living In Your Home**

	No	%
1 Yes	1,449	97.77
2 No	33	2.23
Total	1,482	100.00

- **db025_2_ : Is Mother Living In Your Home**

	No	%
1 Yes	7	100.00
Total	7	100.00

- **db025_4_ : Is Mother-in-law Living In Your Home**

	No	%
1 Yes	1	33.33
2 No	2	66.67
Total	3	100.00

- **db025_5_ : Is Father-in-law Living In Your Home**

	No	%
2 No	2	100.00
Total	2	100.00

- **db025_6_ : Is Children Living In Your Home**

	No	%
1 Yes	348	81.12
2 No	81	18.88
Total	429	100.00

- **db025_7_ : Is Children Living In Your Home**

	No	%
1 Yes	39	36.45
2 No	68	63.55
Total	107	100.00

- **db025_8_ : Is Children Living In Your Home**

	No	%
1 Yes	20	32.79
2 No	41	67.21
Total	61	100.00

- **db025_9_ : Is Children Living In Your Home**

	No	%
1 Yes	13	38.24
2 No	21	61.76
Total	34	100.00

- **db025_10_ : Is Children Living In Your Home**

	No	%
1 Yes	5	33.33
2 No	10	66.67
Total	15	100.00

- **db025_11_ : Is Children Living In Your Home**

	No	%
1 Yes	2	22.22
2 No	7	77.78
Total	9	100.00

- **db025_12_ : Is Children Living In Your Home**

	No	%
1 Yes	1	50.00
2 No	1	50.00
Total	2	100.00

- **db025_31_ : Is Sibling Living In Your Home**

	No	%
1 Yes	11	37.93
2 No	18	62.07
Total	29	100.00

- **db025_32_ : Is Sibling of Spouse Living In Your Home**

	No	%
1 Yes	2	33.33
2 No	4	66.67
Total	6	100.00

- **db025_33_ : Is Brother-in-law,Sister in law Living In Your Home**

	No	%
1 Yes	3	75.00
2 No	1	25.00
Total	4	100.00

- **db025_34_ : Is Children's Spouse Living In Your Home**

	No	%
1 Yes	226	81.59
2 No	51	18.41
Total	277	100.00

- **db025_35_ : Is Grandchild Living In Your Home**

	No	%
1 Yes	72	79.12
2 No	19	20.88
Total	91	100.00

- **db025_36_ : Is Other Relative Living In Your Home**

	No	%
1 Yes	11	39.29
2 No	17	60.71
Total	28	100.00

- **db025_37_ : Is Paid Helper Living In Your Home**

	No	%
1 Yes	13	65.00
2 No	7	35.00
Total	20	100.00

- **db025_39_ : Is Other Living In Your Home**

	No	%
1 Yes	22	48.89
2 No	23	51.11
Total	45	100.00

- **db026_1_ : Is Spouse Paid to Help You**

	No	%
1 Yes	6	0.41
2 No	1,474	99.59
Total	1,480	100.00

- **db026_2_ : Is Mother Paid to Help You**

	No	%
2 No	7	100.00
Total	7	100.00

- **db026_4_ : Is Mother-in-law Paid to Help You**

	No	%
2 No	3	100.00
Total	3	100.00

- **db026_5_ : Is Father-in-law Paid to Help You**

	No	%
2 No	2	100.00
Total	2	100.00

- **db026_6_ : Is Children Paid to Help You**

	No	%
1 Yes	5	1.17
2 No	423	98.83
Total	428	100.00

- **db026_7_ : Is Children Paid to Help You**

	No	%
2 No	107	100.00
Total	107	100.00

- **db026_8_ : Is Children Paid to Help You**

	No	%
1 Yes	1	1.64
2 No	60	98.36
Total	61	100.00

- **db026_9_ : Is Children Paid to Help You**

	No	%
2 No	34	100.00
Total	34	100.00

- **db026_10_ : Is Children Paid to Help You**

	No	%
2 No	15	100.00
Total	15	100.00

- **db026_11_ : Is Children Paid to Help You**

	No	%

2 No	9	100.00
Total	9	100.00

- **db026_12_ : Is Children Paid to Help You**

	No	%
2 No	2	100.00
Total	2	100.00

- **db026_31_ : Is Sibling Paid to Help You**

	No	%
2 No	29	100.00
Total	29	100.00

- **db026_32_ : Is Sibling of Spouse Paid to Help You**

	No	%
2 No	6	100.00
Total	6	100.00

- **db026_33_ : Is Brother-in-law,Sister in law Paid to Help You**

	No	%
2 No	4	100.00
Total	4	100.00

- **db026_34_ : Is Children's Spouse Paid to Help You**

	No	%
1 Yes	1	0.36
2 No	276	99.64
Total	277	100.00

- **db026_35_ : Is Grandchild Paid to Help You**

	No	%
2 No	91	100.00
Total	91	100.00

- **db026_36_ : Is Other Relative Paid to Help You**

	No	%
1 Yes	1	3.57
2 No	27	96.43
Total	28	100.00

- **db026_37_ : Is Paid Helper Paid to Help You**

	No	%
1 Yes	18	90.00
2 No	2	10.00
Total	20	100.00

- **db026_39_ : Is Other Paid to Help You**

	No	%
1 Yes	1	2.22
2 No	44	97.78
Total	45	100.00

- **db027 : Your Total Pay for the Help**

Mean	Min	Max	OBS
735.6	0.0	3,000.0	32

- **db028 : Who Paid Most of This Cost for the Help You Got**

	No	%
1 Yourself	9	29.03
2 Spouse	9	29.03
7 HMemberChildren[1]	6	19.35
8 HMemberChildren[2]	3	9.68
9 HMemberChildren[3]	2	6.45
11 HMemberChildren[5]	1	3.23
38 Other	1	3.23
Total	31	100.00

- **db029s1 : Do You Use Walking Stick**

	No	%
1 Walking stick	965	100.00

Total	965	100.00

- **db029s2 : Do You Use Travel Device**

	No	%
2 Travel device	13	100.00
Total	13	100.00

- **db029s3 : Do You Use Manual Wheelchair**

	No	%
3 Manual wheelchair	69	100.00
Total	69	100.00

- **db029s4 : Do You Use Electric Wheelchair**

	No	%
4 Electric Wheelchair	6	100.00
Total	6	100.00

- **db029s5 : Do You Use Catheter, Urine Collection Bag**

	No	%
5 catheter, urine collection bag	29	100.00
Total	29	100.00

- **db029s6 : Do You Use Toilet Series**

	No	%
6 Toilet Series	193	100.00
Total	193	100.00

- **db029s7 : Use None of the Above**

	No	%
7 None of the above	16,420	100.00
Total	16,420	100.00

- **db030 : Is There Any Other One to Help You In The Future**

	No	%
1 Yes	11,671	69.64
2 No	5,088	30.36
Total	16,759	100.00

- **db031s1 : Mother Is The One to Help You In The Future**

	No	%
1 Mother	22	100.00
Total	22	100.00

- **db031s2 : Father Is The One to Help You In The Future**

	No	%
2 Father	11	100.00
Total	11	100.00

- **db031s3 : Mother-in-law Is The One to Help You In The Future**

	No	%
3 Mother-in-law	7	100.00
Total	7	100.00

- **db031s4 : Father-in-law Is The One to Help You In The Future**

	No	%
4 Father-in-law	9	100.00
Total	9	100.00

- **db031s5 : Children Is The One to Help You In The Future**

	No	%
5 HMemberChildren[1]	8,444	100.00
Total	8,444	100.00

- **db031s6 : Children Is The One to Help You In The Future**

	No	%
6 HMemberChildren[2]	6,024	100.00

Total	6,024	100.00

- **db031s7 : Children Is The One to Help You In The Future**

	No	%
7 HMemberChildren[3]	3,098	100.00
Total	3,098	100.00

- **db031s8 : Children Is The One to Help You In The Future**

	No	%
8 HMemberChildren[4]	1,460	100.00
Total	1,460	100.00

- **db031s9 : Children Is The One to Help You In The Future**

	No	%
9 HMemberChildren[5]	597	100.00
Total	597	100.00

- **db031s10 : Children Is The One to Help You In The Future**

	No	%
10 HMemberChildren[6]	224	100.00
Total	224	100.00

- **db031s11 : Children Is The One to Help You In The Future**

	No	%
11 HMemberChildren[7]	82	100.00
Total	82	100.00

- **db031s12 : Children Is The One to Help You In The Future**

	No	%
12 HMemberChildren[8]	20	100.00
Total	20	100.00

- **db031s13 : Children Is The One to Help You In The Future**

	No	%
13 HMemberChildren[9]	5	100.00
Total	5	100.00

- **db031s14 : Children Is The One to Help You In The Future**

	No	%
14 HMemberChildren[10]	2	100.00
Total	2	100.00

- **db031s15 : Children Is The One to Help You In The Future**

No Observations

- **db031s16 : Children Is The One to Help You In The Future**

No Observations

- **db031s17 : Children Is The One to Help You In The Future**

No Observations

- **db031s18 : Children Is The One to Help You In The Future**

No Observations

- **db031s19 : Children Is The One to Help You In The Future**

No Observations

- **db031s20 : Children Is The One to Help You In The Future**

No Observations

- **db031s21 : Children Is The One to Help You In The Future**

No Observations

- **db031s22 : Children Is The One to Help You In The Future**

No Observations

- **db031s23 : Children Is The One to Help You In The Future**

No Observations

- **db031s24 : Children Is The One to Help You In The Future**

No Observations

- **db031s25 : Children Is The One to Help You In The Future**

No Observations

- **db031s26 : Children Is The One to Help You In The Future**

No Observations

- **db031s27 : Children Is The One to Help You In The Future**

No Observations

- **db031s28 : Children Is The One to Help You In The Future**

No Observations

- **db031s29 : Children Is The One to Help You In The Future**

No Observations

- **db031s30 : Sibling Is The One to Help You In The Future**

	No	%
30 Your Siblings	203	100.00
Total	203	100.00

- **db031s31 : Sibling of Spouse Is The One to Help You In The Future**

	No	%
31 Siblings of your spouse	50	100.00
Total	50	100.00

- **db031s32 : Brother-in-law,Sister in law Is The One to Help You In The Future**

	No	%
32 Brother-in-law, sister-in-law	27	100.00
Total	27	100.00

- **db031s33 : Children's Spouse Is The One to Help You In The Future**

	No	%
33 Spouse of child	3,811	100.00
Total	3,811	100.00

- **db031s34 : Grandchild Is The One to Help You In The Future**

	No	%
34 Grandchild	800	100.00
Total	800	100.00

- **db031s35 : Other Relative Is The One to Help You In The Future**

	No	%
35 Other relative	315	100.00
Total	315	100.00

- **db031s36 : Paid Helper Is The One to Help You In The Future**

	No	%
36 Paid helpersuch as nanny	77	100.00
Total	77	100.00

- **db031s37 : Volunteer Is The One to Help You In The Future**

	No	%
37 Volunteer or Employee of facility	16	100.00
Total	16	100.00

- **db031s38 : Other Is The One to Help You In The Future**

	No	%
38 Other	463	100.00
Total	463	100.00

- **db032 : How Often Did the Respondent Receive Assistance**

	No	%
1 Never	13,769	78.46
2 A few times	2,956	16.85
3 Most or all of the time	469	2.67
4 The section was done by a proxy reporter	354	2.02
Total	17,548	100.00

- **db033 : What Is Your Relationship to Respondent**

	No	%
1 Spouse	269	68.62
2 Mother	3	0.77
3 Father	2	0.51
4 Mother-in-law	3	0.77

6 Sibling	4	1.02
7 Brother-in-law, sister-in-law	2	0.51
8 Child	72	18.37
9 Spouse of child	29	7.40
10 Grandchild	4	1.02
11 Other relative	2	0.51
12 Helper or other non-relative	2	0.51
Total	392	100.00

- **db034 : What Is the Main Reason for Proxy**

	No	%
1 The respondent has serious physical handicaps	24	10.48
2 The respondent has serious mental handicaps	10	4.37
3 The respondent has rejected this interview	12	5.24
4 Other	183	79.91
Total	229	100.00

- **dc001s1 : Year Is Correct**

	No	%
1 Year is correct	11,569	100.00
Total	11,569	100.00

- **dc001s2 : Month Is Correct**

	No	%
2 Month is correct	13,526	100.00
Total	13,526	100.00

- **dc001s3 : Day Is Correct**

	No	%
3 Day is correct	11,679	100.00
Total	11,679	100.00

- **dc002 : Checking Day of Week**

	No	%
1 Day of week OK	9,282	68.57
2 Day of week not OK	4,255	31.43
Total	13,537	100.00

- **dc003 : Checking Season**

	No	%
1 Season OK	13,543	89.12
2 Season not OK	1,654	10.88
Total	15,197	100.00

- **dc004 : Self Comment of Memory**

	No	%
1 Excellent	78	0.49
2 Very good	871	5.43
3 Good	2,245	13.99
4 Fair	7,201	44.89
5 Poor	5,648	35.21
Total	16,043	100.00

- **dc005 : Need More Explanation**

	No	%
1 Yes	14,439	91.35
2 No	1,368	8.65
Total	15,807	100.00

- **dc006_version : Wordlist Version**

	No	%
1	3,601	24.78
2	3,667	25.24
3	3,612	24.86
4	3,651	25.13
Total	14,531	100.00

- **dc006s1 : Words Recall**

	No	%
1 DC006_Wordlist[1]	10,572	100.00
Total	10,572	100.00

- **dc006s2 : Words Recall**

	No	%

2 DC006_Wordlist[2]	6,576	100.00
Total	6,576	100.00

- **dc006s3 : Words Recall**

	No	%
3 DC006_Wordlist[3]	3,866	100.00
Total	3,866	100.00

- **dc006s4 : Words Recall**

	No	%
4 DC006_Wordlist[4]	5,311	100.00
Total	5,311	100.00

- **dc006s5 : Words Recall**

	No	%
5 DC006_Wordlist[5]	5,012	100.00
Total	5,012	100.00

- **dc006s6 : Words Recall**

	No	%
6 DC006_Wordlist[6]	5,041	100.00
Total	5,041	100.00

- **dc006s7 : Words Recall**

	No	%
7 DC006_Wordlist[7]	4,676	100.00
Total	4,676	100.00

- **dc006s8 : Words Recall**

	No	%
8 DC006_Wordlist[8]	5,286	100.00
Total	5,286	100.00

- **dc006s9 : Words Recall**

	No	%
9 DC006_Wordlist[9]	5,478	100.00
Total	5,478	100.00

- **dc006s10 : Words Recall**

	No	%
10 DC006_Wordlist[10]	6,109	100.00
Total	6,109	100.00

- **dc006s11 : Words Recall**

	No	%
11 None	200	100.00
Total	200	100.00

- **dc008_1 : Automatically Record Hour**

	No	%
0	8	0.06
1	4	0.03
2	6	0.04
3	2	0.01
4	5	0.03
5	6	0.04
6	23	0.16
7	189	1.31
8	634	4.39
9	1,412	9.78
10	1,690	11.70
11	1,287	8.91
12	727	5.03
13	722	5.00
14	1,134	7.85
15	1,493	10.34
16	1,509	10.45
17	1,157	8.01
18	708	4.90
19	633	4.38
20	611	4.23
21	352	2.44
22	110	0.76
23	19	0.13
Total	14,441	100.00

- **dc008_2 : Automatically Record Minute**

	No	%
0	259	1.79
1	247	1.71
2	235	1.63
3	265	1.84
4	214	1.48
5	217	1.50
6	239	1.66
7	224	1.55
8	246	1.70
9	263	1.82
10	240	1.66
11	252	1.75
12	234	1.62
13	233	1.61
14	200	1.38
15	211	1.46
16	227	1.57
17	246	1.70
18	241	1.67
19	242	1.68
20	240	1.66
21	210	1.45
22	259	1.79
23	267	1.85
24	247	1.71
25	248	1.72
26	246	1.70
27	229	1.59
28	256	1.77
29	259	1.79
30	235	1.63
31	228	1.58
32	249	1.72
33	239	1.66
34	219	1.52
35	257	1.78
36	262	1.81
37	261	1.81
38	237	1.64
39	253	1.75
40	239	1.66
41	246	1.70
42	237	1.64
43	234	1.62
44	223	1.54
45	239	1.66
46	266	1.84
47	241	1.67
48	228	1.58

49	227	1.57
50	237	1.64
51	221	1.53
52	252	1.75
53	249	1.72
54	242	1.68
55	230	1.59
56	240	1.66
57	239	1.66
58	261	1.81
59	254	1.76
Total	14,441	100.00

- **dc009 : Bothered by Things**

	No	%
1 Rarely or none of the time ¡1 day	7,180	45.10
2 Some or a little of the time 1-2 days	3,481	21.86
3 Occasionally or a moderate amount of the time 3-4 days	2,860	17.96
4 Most or all of the time 5-7 days	2,400	15.07
Total	15,921	100.00

- **dc010 : Had Trouble Keeping Mind**

	No	%
1 Rarely or none of the time ¡1 day	7,978	50.41
2 Some or a little of the time 1-2 days	3,200	20.22
3 Occasionally or a moderate amount of the time 3-4 days	2,626	16.59
4 Most or all of the time 5-7 days	2,021	12.77
Total	15,825	100.00

- **dc011 : Felt Depressed**

	No	%
1 Rarely or none of the time ¡1 day	7,240	45.65
2 Some or a little of the time 1-2 days	3,673	23.16
3 Occasionally or a moderate amount of the time 3-4 days	2,988	18.84
4 Most or all of the time 5-7 days	1,959	12.35
Total	15,860	100.00

- **dc012 : Felt Everything I Did Was An Effort**

	No	%
1 Rarely or none of the time ¡1 day	7,710	48.52
2 Some or a little of the time 1-2 days	2,951	18.57

3 Occasionally or a moderate amount of the time 3-4 days	2,624	16.51
4 Most or all of the time 5-7 days	2,605	16.39
Total	15,890	100.00

- **dc013 : Felt Hopeful About the Future**

	No	%
1 Rarely or none of the time ¡1 day	3,490	22.33
2 Some or a little of the time 1-2 days	2,365	15.13
3 Occasionally or a moderate amount of the time 3-4 days	3,116	19.93
4 Most or all of the time 5-7 days	6,660	42.61
Total	15,631	100.00

- **dc014 : Felt fearful**

	No	%
1 Rarely or none of the time ¡1 day	12,606	78.93
2 Some or a little of the time 1-2 days	1,673	10.47
3 Occasionally or a moderate amount of the time 3-4 days	1,013	6.34
4 Most or all of the time 5-7 days	680	4.26
Total	15,972	100.00

- **dc015 : Sleep Was Restless**

	No	%
1 Rarely or none of the time ¡1 day	7,791	48.72
2 Some or a little of the time 1-2 days	2,682	16.77
3 Occasionally or a moderate amount of the time 3-4 days	2,379	14.88
4 Most or all of the time 5-7 days	3,139	19.63
Total	15,991	100.00

- **dc016 : Happy**

	No	%
1 Rarely or none of the time ¡1 day	2,637	16.53
2 Some or a little of the time 1-2 days	2,449	15.36
3 Occasionally or a moderate amount of the time 3-4 days	4,065	25.49
4 Most or all of the time 5-7 days	6,797	42.62
Total	15,948	100.00

- **dc017 : Felt Lonely**

	No	%

1 Rarely or none of the time ¡1 day	11,211	70.40
2 Some or a little of the time 1-2 days	2,020	12.69
3 Occasionally or a moderate amount of the time 3-4 days	1,444	9.07
4 Most or all of the time 5-7 days	1,249	7.84
Total	15,924	100.00

- **dc018 : Could Not Get Going**

	No	%
1 Rarely or none of the time ¡1 day	12,454	78.40
2 Some or a little of the time 1-2 days	1,722	10.84
3 Occasionally or a moderate amount of the time 3-4 days	996	6.27
4 Most or all of the time 5-7 days	714	4.49
Total	15,886	100.00

- **dc019 : 100 Minus 7**

Mean	Min	Max	OBS
89.9	0.0	1,300.0	14,278

- **dc020 : Minus 7**

Mean	Min	Max	OBS
80.3	0.0	1,400.0	13,414

- **dc021 : Minus 7**

Mean	Min	Max	OBS
72.4	0.0	7,972.0	12,947

- **dc022 : Minus 7**

Mean	Min	Max	OBS
63.8	0.0	522.0	12,714

- **dc023 : Minus 7**

Mean	Min	Max	OBS
56.6	-1.0	165.0	12,525

- **dc024 : Use aid for the Number Substraction**

	No	%
1 Yes	404	2.53
2 No	15,535	97.47
Total	15,939	100.00

- **dc025 : Drew the Picture or Failed**

	No	%
1 Drew the picture	10,004	63.20
2 Failed to draw the picture	5,824	36.80
Total	15,828	100.00

- **dc026_1 : Automatically Record Hour**

	No	%
0	7	0.05
1	5	0.03
2	6	0.04
3	2	0.01
4	4	0.03
5	7	0.05
6	18	0.12
7	169	1.17
8	580	4.02
9	1,358	9.41
10	1,712	11.86
11	1,318	9.13
12	743	5.15
13	715	4.95
14	1,091	7.56
15	1,480	10.26
16	1,528	10.59
17	1,193	8.27
18	729	5.05
19	637	4.41
20	616	4.27
21	375	2.60
22	117	0.81
23	21	0.15
Total	14,431	100.00

- **dc026_2 : Automatically Record Minute**

	No	%

0	261	1.81
1	256	1.77
2	241	1.67
3	271	1.88
4	235	1.63
5	254	1.76
6	232	1.61
7	235	1.63
8	211	1.46
9	253	1.75
10	252	1.75
11	221	1.53
12	234	1.62
13	252	1.75
14	245	1.70
15	231	1.60
16	240	1.66
17	223	1.55
18	232	1.61
19	232	1.61
20	235	1.63
21	232	1.61
22	224	1.55
23	240	1.66
24	242	1.68
25	252	1.75
26	240	1.66
27	224	1.55
28	266	1.84
29	226	1.57
30	258	1.79
31	260	1.80
32	247	1.71
33	241	1.67
34	232	1.61
35	257	1.78
36	256	1.77
37	215	1.49
38	238	1.65
39	254	1.76
40	251	1.74
41	236	1.64
42	228	1.58
43	256	1.77
44	234	1.62
45	252	1.75
46	232	1.61
47	237	1.64
48	209	1.45
49	250	1.73
50	253	1.75
51	246	1.70

52	256	1.77
53	235	1.63
54	222	1.54
55	250	1.73
56	221	1.53
57	219	1.52
58	249	1.73
59	245	1.70
Total	14,431	100.00

- **dc027s1 : Recall Words**

	No	%
1 DC006_Wordlist[1]	8,674	100.00
Total	8,674	100.00

- **dc027s2 : Recall Words**

	No	%
2 DC006_Wordlist[2]	5,357	100.00
Total	5,357	100.00

- **dc027s3 : Recall Words**

	No	%
3 DC006_Wordlist[3]	3,290	100.00
Total	3,290	100.00

- **dc027s4 : Recall Words**

	No	%
4 DC006_Wordlist[4]	4,439	100.00
Total	4,439	100.00

- **dc027s5 : Recall Words**

	No	%
5 DC006_Wordlist[5]	3,965	100.00
Total	3,965	100.00

- **dc027s6 : Recall Words**

	No	%
6 DC006_Wordlist[6]	3,887	100.00
Total	3,887	100.00

- **dc027s7 : Recall Words**

	No	%
7 DC006_Wordlist[7]	3,724	100.00
Total	3,724	100.00

- **dc027s8 : Recall Words**

	No	%
8 DC006_Wordlist[8]	3,826	100.00
Total	3,826	100.00

- **dc027s9 : Recall Words**

	No	%
9 DC006_Wordlist[9]	3,673	100.00
Total	3,673	100.00

- **dc027s10 : Recall Words**

	No	%
10 DC006_Wordlist[10]	3,421	100.00
Total	3,421	100.00

- **dc027s11 : Recall Words**

	No	%
11 None	1,915	100.00
Total	1,915	100.00

- **dc028 : Self Comment of Your Life**

	No	%
1 Completely satisfied	265	1.85
2 Very satisfied	2,879	20.05

3 Somewhat satisfied	8,942	62.26
4 Not very satisfied	1,911	13.31
5 Not at all satisfied	365	2.54
Total	14,362	100.00

- **de001 : Bodily Aches or Pains**

	No	%
1 None	1,968	52.78
2 Mild	726	19.47
3 Moderate	539	14.45
4 Severe	426	11.42
5 Extreme	70	1.88
Total	3,729	100.00

- **de002 : The Difficulty of Falling Asleep and Waking Up**

	No	%
1 None	2,188	58.72
2 Mild	668	17.93
3 Moderate	490	13.15
4 Severe	304	8.16
5 Extreme	76	2.04
Total	3,726	100.00

- **de003 : Moving Around**

	No	%
1 None	3,140	84.09
2 Mild	251	6.72
3 Moderate	167	4.47
4 Severe	131	3.51
5 Extreme	45	1.21
Total	3,734	100.00

- **de004 : The Difficulty of Concentrating or Remembering Things**

	No	%
1 None	2,085	56.09
2 Mild	749	20.15
3 Moderate	540	14.53
4 Severe	297	7.99
5 Extreme	46	1.24
Total	3,717	100.00

- **de005 : A Problem Because of Shortness of Breath**

	No	%
1 None	2,964	79.40
2 Mild	416	11.14
3 Moderate	198	5.30
4 Severe	120	3.21
5 Extreme	35	0.94
Total	3,733	100.00

- **de006 : Feeling Sad,Low,or Depressed**

	No	%
1 None	2,601	69.86
2 Mild	586	15.74
3 Moderate	331	8.89
4 Severe	157	4.22
5 Extreme	48	1.29
Total	3,723	100.00

- **de007 : Evaluate Pain 1**

	No	%
1 None	248	22.18
2 Mild	609	54.47
3 Moderate	199	17.80
4 Severe	55	4.92
5 Extreme	7	0.63
Total	1,118	100.00

- **de008 : Evaluate Pain 2**

	No	%
1 None	245	21.97
2 Mild	592	53.09
3 Moderate	207	18.57
4 Severe	63	5.65
5 Extreme	8	0.72
Total	1,115	100.00

- **de009 : Evaluate Pain 3**

	No	%
1 None	58	5.16

2 Mild	170	15.14
3 Moderate	384	34.19
4 Severe	427	38.02
5 Extreme	84	7.48
Total	1,123	100.00

- **de010 : Evaluate Sleep 1**

	No	%
1 None	244	19.92
2 Mild	512	41.80
3 Moderate	329	26.86
4 Severe	128	10.45
5 Extreme	12	0.98
Total	1,225	100.00

- **de011 : Evaluate Sleep 2**

	No	%
1 None	99	8.18
2 Mild	251	20.73
3 Moderate	457	37.74
4 Severe	355	29.31
5 Extreme	49	4.05
Total	1,211	100.00

- **de012 : Evaluate Sleep 3**

	No	%
1 None	114	9.44
2 Mild	205	16.97
3 Moderate	350	28.97
4 Severe	416	34.44
5 Extreme	123	10.18
Total	1,208	100.00

- **de013 : Evaluate Mobility 1**

	No	%
1 None	541	48.26
2 Mild	337	30.06
3 Moderate	176	15.70
4 Severe	63	5.62
5 Extreme	4	0.36
Total	1,121	100.00

- **de014 : Evaluate Mobility 2**

	No	%
1 None	262	23.46
2 Mild	332	29.72
3 Moderate	330	29.54
4 Severe	168	15.04
5 Extreme	25	2.24
Total	1,117	100.00

- **de015 : Evaluate Mobility 3**

	No	%
1 None	57	5.08
2 Mild	100	8.90
3 Moderate	297	26.45
4 Severe	519	46.22
5 Extreme	150	13.36
Total	1,123	100.00

- **de016 : Evaluate Cognition 1**

	No	%
1 None	468	39.03
2 Mild	495	41.28
3 Moderate	167	13.93
4 Severe	63	5.25
5 Extreme	6	0.50
Total	1,199	100.00

- **de017 : Evaluate Cognition 2**

	No	%
1 None	190	15.82
2 Mild	444	36.97
3 Moderate	382	31.81
4 Severe	180	14.99
5 Extreme	5	0.42
Total	1,201	100.00

- **de018 : Evaluate Cognition 3**

	No	%
1 None	200	16.76
2 Mild	313	26.24
3 Moderate	398	33.36
4 Severe	247	20.70
5 Extreme	35	2.93
Total	1,193	100.00

- **de019 : Evaluate Breathing 1**

	No	%
1 None	188	16.29
2 Mild	327	28.34
3 Moderate	307	26.60
4 Severe	301	26.08
5 Extreme	31	2.69
Total	1,154	100.00

- **de020 : Evaluate Breathing 2**

	No	%
1 None	83	7.26
2 Mild	117	10.24
3 Moderate	289	25.28
4 Severe	522	45.67
5 Extreme	132	11.55
Total	1,143	100.00

- **de021 : Evaluate Breathing 3**

	No	%
1 None	72	6.30
2 Mild	53	4.64
3 Moderate	135	11.82
4 Severe	513	44.92
5 Extreme	369	32.31
Total	1,142	100.00

- **de022 : Evaluate Affect 1**

	No	%
1 None	299	25.15
2 Mild	538	45.25
3 Moderate	272	22.88
4 Severe	76	6.39

	No	%
5 Extreme	4	0.34
Total	1,189	100.00

- **de023 : Evaluate Affect 2**

	No	%
1 None	129	11.00
2 Mild	339	28.90
3 Moderate	478	40.75
4 Severe	205	17.48
5 Extreme	22	1.88
Total	1,173	100.00

- **de024 : Evaluate Affect 3**

	No	%
1 None	62	5.27
2 Mild	88	7.48
3 Moderate	197	16.74
4 Severe	492	41.80
5 Extreme	338	28.72
Total	1,177	100.00

- **flda081_1 : The Number of Self Expectation Age**

	No	%
.E	4	0.02
100	104	0.64
105	31	0.19
110	4	0.02
115	19	0.12
75	12,273	75.61
80	1,556	9.59
85	1,145	7.05
90	751	4.63
95	345	2.13
Total	16,232	100.00

- **proxy : Interview Down By Proxy**

	No	%
0 No	16,267	92.47
1 Yes	1,325	7.53
Total	17,592	100.00

6 Health Care and Insurance

- **ID : Individual ID**

A String Variable	
OBS:	17,545

- **householdID : Household ID**

A String Variable	
OBS:	17,545

- **communityID : Community ID**

A String Variable	
OBS:	17,545

- **ea001s1 : Y/N Urban Employee Medical Insurance**

	No	%
1 Urban employee medical insurance yi-bao	1,912	100.00
Total	1,912	100.00

- **ea001s2 : Y/N Urban Resident Medical Insurance**

	No	%
2 Urban resident medical insurance	793	100.00
Total	793	100.00

- **ea001s3 : Y/N New Cooperative Medical Insurance**

	No	%
3 New cooperative medical insurance he-zuo-yi-liao	12,902	100.00
Total	12,902	100.00

- **ea001s4 : Y/N Urban and Rural Resident Medical Insurance**

	No	%
4 Urban and rural resident medical insurance	224	100.00
Total	224	100.00

- **ea001s5 : Y/N Government Medical Insurance**

	No	%
5 Government medical insurance gong-fei	369	100.00
Total	369	100.00

- **ea001s6 : Y/N Medical Aid**

	No	%
6 Medical aid	14	100.00
Total	14	100.00

- **ea001s7 : Y/N Private Medical Insurance: Purchased By R's Union**

	No	%
7 Private medical Insurance: Purchased by Rs union	119	100.00
Total	119	100.00

- **ea001s8 : Y/N Private Medical Insurance: Purchased By Individual**

	No	%
8 Private medical Insurance: Purchased by Individual	321	100.00
Total	321	100.00

- **ea001s9 : Y/N Other Medical Insurance**

	No	%
9 Other medical insurance specify	130	100.00
Total	130	100.00

- **ea001s10 : Y/N No Insurance**

	No	%
10 No insurance	1,180	100.00
Total	1,180	100.00

- **ea002_1_ : Do You Have Supplemental Insurance to This Plan**

	No	%
1 Yes	112	5.82
2 No	1,813	94.18
Total	1,925	100.00

- **ea002_2_ : Do You Have Supplemental Insurance to This Plan**

	No	%
1 Yes	35	4.27
2 No	784	95.73
Total	819	100.00

- **ea002_3_ : Do You Have Supplemental Insurance to This Plan**

	No	%
1 Yes	446	3.44
2 No	12,513	96.56
Total	12,959	100.00

- **ea002_4_ : Do You Have Supplemental Insurance to This Plan**

	No	%
1 Yes	9	3.93
2 No	220	96.07
Total	229	100.00

- **ea002_5_ : Do You Have Supplemental Insurance to This Plan**

	No	%
1 Yes	13	3.45
2 No	364	96.55
Total	377	100.00

- **ea002_6_ : Do You Have Supplemental Insurance to This Plan**

	No	%
2 No	14	100.00
Total	14	100.00

- **ea002_7_ : Do You Have Supplemental Insurance to This Plan**

	No	%
1 Yes	9	7.26
2 No	115	92.74
Total	124	100.00

- **ea002_8_ : Do You Have Supplemental Insurance to This Plan**

	No	%
1 Yes	51	14.91
2 No	291	85.09
Total	342	100.00

- **ea003_1_ : Where Did You Set Up Your Insurance Account**

	No	%
1 This County	1,776	92.45
2 (If it is not in this county) the place of your Hukou	106	5.52
3 Other	39	2.03
Total	1,921	100.00

- **ea003_1_1 : Other Province Set Up Your Insurance Account**

	No	%
03	2	5.00
05	1	2.50
06	2	5.00
07	2	5.00
10	2	5.00
11	3	7.50
13	3	7.50
14	2	5.00
15	4	10.00
17	4	10.00
18	1	2.50
27	1	2.50
28	1	2.50
29	2	5.00
32	4	10.00
33	4	10.00
34	2	5.00
Total	40	100.00

- **ea003_1_2 : Other Province Set Up Your Insurance Account**

	No	%

11	1	33.33
20	2	66.67
Total	3	100.00

- **ea003_1_3 : Other Province Set Up Your Insurance Account**

	No	%
03	2	6.25
05	1	3.13
06	2	6.25
07	1	3.13
12	1	3.13
13	1	3.13
14	6	18.75
20	1	3.13
21	3	9.38
24	1	3.13
26	4	12.50
27	1	3.13
29	1	3.13
32	4	12.50
33	3	9.38
Total	32	100.00

- **ea003_1_4 : Other Province Set Up Your Insurance Account**

	No	%
05	1	50.00
12	1	50.00
Total	2	100.00

- **ea003_1_5 : Other Province Set Up Your Insurance Account**

	No	%
01	1	7.14
03	1	7.14
04	1	7.14
05	2	14.29
07	1	7.14
10	2	14.29
11	1	7.14
14	1	7.14
15	1	7.14
17	1	7.14
26	1	7.14
32	1	7.14

Total	14	100.00

- **ea003_1_6 : Other Province Set Up Your Insurance Account**

No Observations

- **ea003_1_7 : Other Province Set Up Your Insurance Account**

	No	%
05	1	20.00
12	1	20.00
15	2	40.00
19	1	20.00
Total	5	100.00

- **ea003_1_8 : Other Province Set Up Your Insurance Account**

	No	%
05	1	12.50
07	1	12.50
09	1	12.50
15	1	12.50
19	1	12.50
29	1	12.50
33	2	25.00
Total	8	100.00

- **ea003_2_ : Where Did You Set Up Your Insurance Account**

	No	%
1 This County	763	93.62
2 (If it is not in this county) the place of your Hukou	49	6.01
3 Other	3	0.37
Total	815	100.00

- **ea003_2_1 : Other County Set Up Your Insurance Account**

	No	%
04	1	2.78
0444	1	2.78
11	1	2.78
16	1	2.78

24	5	13.89
40	7	19.44
4628	1	2.78
4631	1	2.78
4654	1	2.78
53	5	13.89
5359	1	2.78
5531	1	2.78
60	2	5.56
74	1	2.78
7454	1	2.78
82	2	5.56
8244	1	2.78
84	1	2.78
99	2	5.56
Total	36	100.00

- **ea003_2_2 : Other County Set Up Your Insurance Account**

	No	%
2454	2	66.67
53	1	33.33
Total	3	100.00

- **ea003_2_3 : Other County Set Up Your Insurance Account**

	No	%
01	2	6.25
0159	2	6.25
04	1	3.13
0459	1	3.13
16	1	3.13
1659	1	3.13
2431	2	6.25
2439	1	3.13
2481	1	3.13
2759	3	9.38
2781	1	3.13
40	5	15.63
49	3	9.38
4976	1	3.13
5306	2	6.25
55	1	3.13
74	2	6.25
82	1	3.13
8259	1	3.13
Total	32	100.00

- **ea003_2_4 : Other County Set Up Your Insurance Account**

	No	%
40	1	50.00
4078	1	50.00
Total	2	100.00

- **ea003_2_5 : Other County Set Up Your Insurance Account**

	No	%
04	1	7.14
11	2	14.29
24	2	14.29
40	5	35.71
46	1	7.14
53	1	7.14
55	1	7.14
83	1	7.14
Total	14	100.00

- **ea003_2_6 : Other County Set Up Your Insurance Account**

No Observations

- **ea003_2_7 : Other County Set Up Your Insurance Account**

	No	%
4038	1	33.33
53	1	33.33
84	1	33.33
Total	3	100.00

- **ea003_2_8 : Other County Set Up Your Insurance Account**

	No	%
11	1	14.29
40	3	42.86
4008	1	14.29
74	1	14.29
84	1	14.29
Total	7	100.00

- **ea003_3_ : Where Did You Set Up Your Insurance Account**

	No	%
1 This County	12,317	95.09
2 (If it is not in this county) the place of your Hukou	604	4.66
3 Other	32	0.25
Total	12,953	100.00

- **ea003_4_ : Where Did You Set Up Your Insurance Account**

	No	%
1 This County	216	95.15
2 (If it is not in this county) the place of your Hukou	9	3.96
3 Other	2	0.88
Total	227	100.00

- **ea003_5_ : Where Did You Set Up Your Insurance Account**

	No	%
1 This County	352	92.88
2 (If it is not in this county) the place of your Hukou	13	3.43
3 Other	14	3.69
Total	379	100.00

- **ea003_6_ : Where Did You Set Up Your Insurance Account**

	No	%
1 This County	11	78.57
2 (If it is not in this county) the place of your Hukou	3	21.43
Total	14	100.00

- **ea003_7_ : Where Did You Set Up Your Insurance Account**

	No	%
1 This County	116	94.31
2 (If it is not in this county) the place of your Hukou	2	1.63
3 Other	5	4.07
Total	123	100.00

- **ea003_8_ : Where Did You Set Up Your Insurance Account**

	No	%

1 This County	331	96.50
2 (If it is not in this county) the place of your Hukou	5	1.46
3 Other	7	2.04
Total	343	100.00

- **ea003_9_ : Where Did You Set Up Your Insurance Account**

	No	%
1 This County	1	100.00
Total	1	100.00

- **ea004_1_ : Ways of Reimbursement**

	No	%
1 Get the reimbursement immediately	898	48.83
2 You should pay it first and then get the reimbursement later.	941	51.17
Total	1,839	100.00

- **ea004_2_ : Ways of Reimbursement**

	No	%
1 Get the reimbursement immediately	233	31.23
2 You should pay it first and then get the reimbursement later.	513	68.77
Total	746	100.00

- **ea004_3_ : Ways of Reimbursement**

	No	%
1 Get the reimbursement immediately	3,927	31.05
2 You should pay it first and then get the reimbursement later.	8,720	68.95
Total	12,647	100.00

- **ea004_4_ : Ways of Reimbursement**

	No	%
1 Get the reimbursement immediately	109	50.46
2 You should pay it first and then get the reimbursement later.	107	49.54
Total	216	100.00

- **ea004_5_ : Ways of Reimbursement**

	No	%
1 Get the reimbursement immediately	206	55.98
2 You should pay it first and then get the reimbursement later.	162	44.02
Total	368	100.00

- **ea004_6_ : Ways of Reimbursement**

	No	%
1 Get the reimbursement immediately	8	66.67
2 You should pay it first and then get the reimbursement later.	4	33.33
Total	12	100.00

- **ea004_7_ : Ways of Reimbursement**

	No	%
1 Get the reimbursement immediately	74	62.71
2 You should pay it first and then get the reimbursement later.	44	37.29
Total	118	100.00

- **ea004_8_ : Ways of Reimbursement**

	No	%
1 Get the reimbursement immediately	51	16.56
2 You should pay it first and then get the reimbursement later.	257	83.44
Total	308	100.00

- **ea004_9_ : Ways of Reimbursement**

	No	%
2 You should pay it first and then get the reimbursement later.	1	100.00
Total	1	100.00

- **ea005_1_ : Through Which Agency Did You Purchase Your Primary Plan**

	No	%
1 Community committee/ village committee	45	2.35
2 Rname s union	1,622	84.57
3 Agency of Social insurance	235	12.25
4 Private insurance company	4	0.21
5 Other	12	0.63
Total	1,918	100.00

- **ea005_2_ : Through Which Agency Did You Purchase Your Primary Plan**

	No	%
1 Community committee/ village committee	557	69.19
2 Rname s union	73	9.07
3 Agency of Social insurance	146	18.14
4 Private insurance company	2	0.25
5 Other	27	3.35
Total	805	100.00

- **ea005_3_ : Through Which Agency Did You Purchase Your Primary Plan**

	No	%
1 Community committee/ village committee	12,477	96.56
2 Rname s union	23	0.18
3 Agency of Social insurance	367	2.84
4 Private insurance company	3	0.02
5 Other	51	0.39
Total	12,921	100.00

- **ea005_4_ : Through Which Agency Did You Purchase Your Primary Plan**

	No	%
1 Community committee/ village committee	198	88.39
2 Rname s union	9	4.02
3 Agency of Social insurance	16	7.14
5 Other	1	0.45
Total	224	100.00

- **ea005_5_ : Through Which Agency Did You Purchase Your Primary Plan**

	No	%
1 Community committee/ village committee	16	4.24
2 Rname s union	317	84.08
3 Agency of Social insurance	35	9.28
5 Other	9	2.39
Total	377	100.00

- **ea005_6_ : Through Which Agency Did You Purchase Your Primary Plan**

	No	%
1 Community committee/ village committee	3	21.43
2 Rname s union	1	7.14
3 Agency of Social insurance	8	57.14

	No	%
5 Other	2	14.29
Total	14	100.00

- **ea005_7_ : Through Which Agency Did You Purchase Your Primary Plan**

	No	%
1 Community committee/ village committee	7	5.79
2 Rname s union	99	81.82
3 Agency of Social insurance	3	2.48
4 Private insurance company	12	9.92
Total	121	100.00

- **ea005_8_ : Through Which Agency Did You Purchase Your Primary Plan**

	No	%
1 Community committee/ village committee	33	10.28
2 Rname s union	7	2.18
3 Agency of Social insurance	3	0.93
4 Private insurance company	259	80.69
5 Other	19	5.92
Total	321	100.00

- **ea005_9_ : Through Which Agency Did You Purchase Your Primary Plan**

	No	%
1 Community committee/ village committee	1	100.00
Total	1	100.00

- **ea006_1_ : Your Out-of-Pocket Yearly Premium**

Mean	Min	Max	OBS
469.6	0.0	16,000.0	1,575

- **ea006_2_ : Your Out-of-Pocket Yearly Premium**

Mean	Min	Max	OBS
251.6	0.0	7,200.0	768

- **ea006_3_ : Your Out-of-Pocket Yearly Premium**

Mean	Min	Max	OBS

41.0	0.0	34,200.0	12,733

- **ea006_4_ : Your Out-of-Pocket Yearly Premium**

Mean	Min	Max	OBS
206.0	0.0	29,000.0	219

- **ea006_5_ : Your Out-of-Pocket Yearly Premium**

Mean	Min	Max	OBS
161.2	0.0	4,680.0	315

- **ea006_6_ : Your Out-of-Pocket Yearly Premium**

Mean	Min	Max	OBS
46.4	0.0	150.0	11

- **ea006_7_ : Your Out-of-Pocket Yearly Premium**

Mean	Min	Max	OBS
880.1	0.0	4,990.0	89

- **ea006_8_ : Your Out-of-Pocket Yearly Premium**

Mean	Min	Max	OBS
2,012.8	0.0	50,000.0	312

- **ea006_9_ : Your Out-of-Pocket Yearly Premium**

	No	%
30	1	100.00
Total	1	100.00

- **ea007_1_s1 : Yourself Pay the Premium for You**

	No	%
1 Myself	1,058	100.00
Total	1,058	100.00

- **ea007_1_s2 : Children Pay the Premium for You**

	No	%
2 Children	6	100.00
Total	6	100.00

- **ea007_1_s3 : Relatives Pay the Premium for You**

	No	%
3 Relatives	4	100.00
Total	4	100.00

- **ea007_1_s4 : Government Pay the Premium for You**

	No	%
4 Government	78	100.00
Total	78	100.00

- **ea007_1_s5 : R's Union Pay the Premium for You**

	No	%
5 Rsunion	972	100.00
Total	972	100.00

- **ea007_1_s6 : Loan Pay the Premium for You**

No Observations

- **ea007_1_s7 : Donate Pay the Premium for You**

No Observations

- **ea007_1_s8 : Others Pay the Premium for You**

	No	%
8 Others Specify:	84	100.00
Total	84	100.00

- **ea007_2_s1 : Yourself Pay the Premium for You**

	No	%
1 Myself	618	100.00
Total	618	100.00

- **ea007_2_s2 : Children Pay the Premium for You**

	No	%
2 Children	53	100.00
Total	53	100.00

- **ea007_2_s3 : Relatives Pay the Premium for You**

	No	%
3 Relatives	9	100.00
Total	9	100.00

- **ea007_2_s4 : Government Pay the Premium for You**

	No	%
4 Government	60	100.00
Total	60	100.00

- **ea007_2_s5 : R's Union Pay the Premium for You**

	No	%
5 Rsunion	46	100.00
Total	46	100.00

- **ea007_2_s6 : Loan Pay the Premium for You**

No Observations

- **ea007_2_s7 : Donate Pay the Premium for You**

No Observations

- **ea007_2_s8 : Others Pay the Premium for You**

	No	%
8 Others Specify:	27	100.00
Total	27	100.00

- **ea007_3_s1 : Yourself Pay the Premium for You**

	No	%
1 Myself	10,183	100.00
Total	10,183	100.00

- **ea007_3_s2 : Children Pay the Premium for You**

	No	%
2 Children	1,750	100.00
Total	1,750	100.00

- **ea007_3_s3 : Relatives Pay the Premium for You**

	No	%
3 Relatives	173	100.00
Total	173	100.00

- **ea007_3_s4 : Government Pay the Premium for You**

	No	%
4 Government	226	100.00
Total	226	100.00

- **ea007_3_s5 : R's Union Pay the Premium for You**

	No	%
5 Rsunion	608	100.00
Total	608	100.00

- **ea007_3_s6 : Loan Pay the Premium for You**

No Observations

- **ea007_3_s7 : Donate Pay the Premium for You**

	No	%
7 Donate	3	100.00
Total	3	100.00

- **ea007_3_s8 : Others Pay the Premium for You**

	No	%
8 Others Specify:	127	100.00
Total	127	100.00

- **ea007_4_s1 : Yourself Pay the Premium for You**

	No	%
1 Myself	170	100.00
Total	170	100.00

- **ea007_4_s2 : Children Pay the Premium for You**

	No	%
2 Children	21	100.00
Total	21	100.00

- **ea007_4_s3 : Relatives Pay the Premium for You**

	No	%
3 Relatives	1	100.00
Total	1	100.00

- **ea007_4_s4 : Government Pay the Premium for You**

	No	%
4 Government	10	100.00
Total	10	100.00

- **ea007_4_s5 : R's Union Pay the Premium for You**

	No	%
5 Rsunion	27	100.00

Total	27	100.00

- **ea007_4_s6 : Loan Pay the Premium for You**

No Observations

- **ea007_4_s7 : Donate Pay the Premium for You**

No Observations

- **ea007_4_s8 : Others Pay the Premium for You**

	No	%
8 Others Specify:	4	100.00
Total	4	100.00

- **ea007_5_s1 : Yourself Pay the Premium for You**

	No	%
1 Myself	175	100.00
Total	175	100.00

- **ea007_5_s2 : Children Pay the Premium for You**

No Observations

- **ea007_5_s3 : Relatives Pay the Premium for You**

No Observations

- **ea007_5_s4 : Government Pay the Premium for You**

	No	%
4 Government	36	100.00
Total	36	100.00

- **ea007_5_s5 : R's Union Pay the Premium for You**

	No	%
5 Rsunion	176	100.00
Total	176	100.00

- **ea007_5_s6 : Loan Pay the Premium for You**

No Observations

- **ea007_5_s7 : Donate Pay the Premium for You**

No Observations

- **ea007_5_s8 : Others Pay the Premium for You**

	No	%
8 Others Specify:	26	100.00
Total	26	100.00

- **ea007_6_s1 : Yourself Pay the Premium for You**

	No	%
1 Myself	7	100.00
Total	7	100.00

- **ea007_6_s2 : Children Pay the Premium for You**

No Observations

- **ea007_6_s3 : Relatives Pay the Premium for You**

No Observations

- **ea007_6_s4 : Government Pay the Premium for You**

	No	%
4 Government	6	100.00
Total	6	100.00

- **ea007_6_s5 : R's Union Pay the Premium for You**

	No	%
5 Rsunion	2	100.00
Total	2	100.00

- **ea007_6_s6 : Loan Pay the Premium for You**

No Observations

- **ea007_6_s7 : Donate Pay the Premium for You**

No Observations

- **ea007_6_s8 : Others Pay the Premium for You**

No Observations

- **ea007_7_s1 : Yourself Pay the Premium for You**

	No	%
1 Myself	46	100.00
Total	46	100.00

- **ea007_7_s2 : Children Pay the Premium for You**

No Observations

- **ea007_7_s3 : Relatives Pay the Premium for You**

No Observations

- **ea007_7_s4 : Government Pay the Premium for You**

	No	%
4 Government	1	100.00
Total	1	100.00

- **ea007_7_s5 : R's Union Pay the Premium for You**

	No	%
5 Rsunion	77	100.00
Total	77	100.00

- **ea007_7_s6 : Loan Pay the Premium for You**

No Observations

- **ea007_7_s7 : Donate Pay the Premium for You**

No Observations

- **ea007_7_s8 : Others Pay the Premium for You**

	No	%
8 Others Specify:	1	100.00
Total	1	100.00

- **ea007_8_s1 : Yourself Pay the Premium for You**

	No	%
1 Myself	290	100.00
Total	290	100.00

- **ea007_8_s2 : Children Pay the Premium for You**

	No	%
2 Children	30	100.00
Total	30	100.00

- **ea007_8_s3 : Relatives Pay the Premium for You**

	No	%
3 Relatives	1	100.00
Total	1	100.00

- **ea007_8_s4 : Government Pay the Premium for You**

No Observations

- **ea007_8_s5 : R's Union Pay the Premium for You**

	No	%
5 Rsunion	1	100.00
Total	1	100.00

- **ea007_8_s6 : Loan Pay the Premium for You**

No Observations

- **ea007_8_s7 : Donate Pay the Premium for You**

No Observations

- **ea007_8_s8 : Others Pay the Premium for You**

	No	%
8 Others Specify:	2	100.00
Total	2	100.00

- **ea007_9_s1 : Yourself Pay the Premium for You**

	No	%
1 Myself	2	100.00
Total	2	100.00

- **ea007_9_s2 : Children Pay the Premium for You**

No Observations

- **ea007_9_s3 : Relatives Pay the Premium for You**

No Observations

- **ea007_9_s4 : Government Pay the Premium for You**

No Observations

- **ea007_9_s5 : R's Union Pay the Premium for You**

No Observations

- **ea007_9_s6 : Loan Pay the Premium for You**

No Observations

- **ea007_9_s7 : Donate Pay the Premium for You**

No Observations

- **ea007_9_s8 : Others Pay the Premium for You**

No Observations

- **ea008_1_1_ : Year of This Benefit Begin**

Mean	Min	Max	OBS
1,996.5	1,900.0	2,011.0	1,423

- **ea008_1_2_ : Year of This Benefit Begin**

Mean	Min	Max	OBS
2,006.9	1,958.0	2,011.0	729

- **ea008_1_3_ : Year of This Benefit Begin**

Mean	Min	Max	OBS
2,006.6	1,900.0	2,011.0	11,838

- **ea008_1_4_ : Year of This Benefit Begin**

Mean	Min	Max	OBS
2,007.2	1,980.0	2,011.0	206

- **ea008_1_5_ : Year of This Benefit Begin**

Mean	Min	Max	OBS
1,985.3	1,946.0	2,011.0	303

- **ea008_1_6_ : Year of This Benefit Begin**

Mean	Min	Max	OBS
2,005.4	1,978.0	2,011.0	11

- **ea008_1_7_ : Year of This Benefit Begin**

Mean	Min	Max	OBS
1,995.7	1,950.0	2,011.0	85

- **ea008_1_8_ : Year of This Benefit Begin**

Mean	Min	Max	OBS
2,005.0	1,981.0	2,011.0	309

- **ea008_1_9_ : Year of This Benefit Begin**

Mean	Min	Max	OBS
2,002.7	1,971.0	2,011.0	66

- **ea008_1_10_ : Year of This Benefit Begin**

Mean	Min	Max	OBS
2,006.0	2,006.0	2,006.0	2

- **ea008_2_1_ : Month of This Benefit Begin**

	No	%
0	674	47.36
1	175	12.30
2	20	1.41
3	33	2.32
4	49	3.44
5	62	4.36
6	53	3.72
7	70	4.92
8	49	3.44
9	61	4.29
10	66	4.64
11	37	2.60
12	74	5.20
Total	1,423	100.00

- **ea008_2_2_ : Month of This Benefit Begin**

	No	%
0	280	38.41
1	75	10.29
2	8	1.10
3	43	5.90
4	32	4.39
5	29	3.98
6	26	3.57
7	55	7.54
8	26	3.57

9	35	4.80
10	40	5.49
11	32	4.39
12	48	6.58
Total	729	100.00

- **ea008_2_3_ : Month of This Benefit Begin**

	No	%
0	4,343	36.69
1	2,555	21.59
2	324	2.74
3	655	5.53
4	290	2.45
5	158	1.33
6	226	1.91
7	368	3.11
8	304	2.57
9	274	2.31
10	660	5.58
11	600	5.07
12	1,079	9.12
Total	11,836	100.00

- **ea008_2_4_ : Month of This Benefit Begin**

	No	%
0	74	35.92
1	41	19.90
2	3	1.46
3	12	5.83
5	2	0.97
6	3	1.46
7	40	19.42
8	1	0.49
9	1	0.49
10	5	2.43
11	6	2.91
12	18	8.74
Total	206	100.00

- **ea008_2_5_ : Month of This Benefit Begin**

	No	%
0	127	41.91
1	23	7.59
2	11	3.63

3	18	5.94
4	8	2.64
5	16	5.28
6	8	2.64
7	21	6.93
8	28	9.24
9	15	4.95
10	12	3.96
11	5	1.65
12	11	3.63
Total	303	100.00

- **ea008_2_6_ : Month of This Benefit Begin**

	No	%
0	2	18.18
1	2	18.18
3	1	9.09
6	1	9.09
7	2	18.18
8	1	9.09
9	2	18.18
Total	11	100.00

- **ea008_2_7_ : Month of This Benefit Begin**

	No	%
0	22	25.88
1	3	3.53
3	3	3.53
4	7	8.24
5	13	15.29
6	8	9.41
7	9	10.59
8	9	10.59
9	2	2.35
10	4	4.71
11	2	2.35
12	3	3.53
Total	85	100.00

- **ea008_2_8_ : Month of This Benefit Begin**

	No	%
0	111	35.92
1	25	8.09
2	7	2.27

3	19	6.15
4	15	4.85
5	23	7.44
6	25	8.09
7	12	3.88
8	16	5.18
9	13	4.21
10	14	4.53
11	15	4.85
12	14	4.53
Total	309	100.00

- **ea008_2_9_ : Month of This Benefit Begin**

	No	%
0	52	78.79
1	4	6.06
3	2	3.03
4	1	1.52
5	1	1.52
6	1	1.52
7	1	1.52
8	2	3.03
9	1	1.52
10	1	1.52
Total	66	100.00

- **ea008_2_10_ : Month of This Benefit Begin**

	No	%
0	2	100.00
Total	2	100.00

- **ea009 : Reason for Not Having A Health Insurance**

	No	%
1 I do not need it	13	16.25
2 Can not afford it	23	28.75
3 Do not know where or from whom to get it	8	10.00
5 Do not have suitable programs for me to buy	3	3.75
6 Do not know/never thought of it	2	2.50
7 Others	31	38.75
Total	80	100.00

- **eb001 : Have Health Insurance Before**

	No	%
1 Yes	10	12.82
2 No	68	87.18
Total	78	100.00

- **eb002s1 : Did You Have Urban Employee Medical Insurance**

No Observations

- **eb002s2 : Did You Have Urban Resident Medical Insurance**

No Observations

- **eb002s3 : Did You Have New Cooperative Medical Insurance**

	No	%
3 New cooperative medical insurance he-zuo-yi-liao	6	100.00
Total	6	100.00

- **eb002s4 : Did You Have Urban and Rural Resident Medical Insurance**

No Observations

- **eb002s5 : Did You Have Government Medical Insurance**

No Observations

- **eb002s6 : Did You Have Medical Aid**

No Observations

- **eb002s7 : Did You Have Private Medical Insurance: Purchased By R's Union**

No Observations

- **eb002s8 : Did You Have Private Medical Insurance: Purchased By Individual**

	No	%
8 Private medical Insurance: Purchased by Individual	1	100.00
Total	1	100.00

- **eb002s9 : Did You Have Other Medical Insurance**

	No	%
9 Other medical insurance specify	1	100.00
Total	1	100.00

- **eb002s10 : Did You Have No Insurance**

No Observations

- **eb003_1_3_ : Year**

Mean	Min	Max	OBS
2,008.6	1,999.0	2,011.0	5

- **eb003_1_7_ : Year**

No Observations

- **eb003_1_8_ : Year**

No Observations

- **eb003_2_3_ : Month**

	No	%
0	2	40.00
10	2	40.00
12	1	20.00
Total	5	100.00

- **eb003_2_7_ : Month**

No Observations

- **eb003_2_8_ : Month**

No Observations

- **eb004_3_ : Reason that Your Lose it**

	No	%
2 Such insurance will not be proviced locally any more	2	33.33
4 Other:	4	66.67
Total	6	100.00

- **ec001_1 : The Year You Take the Last Examination**

Mean	Min	Max	OBS
1,911.1	-9,999.0	20,111.0	13,196

- **ec001_2 : The Month You Take the Last Examination**

Mean	Min	Max	OBS
22.3	0.0	9,999.0	13,196

- **ec002 : Who pay the physical examination cost?**

	No	%
1 Myself	5,325	32.08
2 Children	745	4.49
3 Relatives	77	0.46

4 Government	1,168	7.04
5 Rs union	1,772	10.68
6 Rs insurance	225	1.36
7 Loan	6	0.04
8 Donate	111	0.67
9 Others Specify:	7,169	43.19
Total	16,598	100.00

- **ed001 : Visited Hospital Last Month**

	No	%
1 Yes	3,367	19.33
2 No	14,055	80.67
Total	17,422	100.00

- **ed002 : Whether Ill In the Last Month**

	No	%
1 Yes	1,467	10.18
2 No	12,939	89.82
Total	14,406	100.00

- **ed003 : Reason for Not Seeking A Visit to Hospital**

	No	%
1 Already under treatment.	181	13.63
2 Illness is not serious. Dont need treatment	647	48.72
3 Poor	231	17.39
4 No time	27	2.03
5 Inconvenient traffic	42	3.16
6 Poor service	7	0.53
7 No available treatment	27	2.03
8 Other	166	12.50
Total	1,328	100.00

- **ed004s1 : Have Visited General Hospital**

	No	%
1 General Hospital	954	100.00
Total	954	100.00

- **ed004s2 : Have Visited Specialized Hospital**

	No	%
2 Specialized hospital	118	100.00
Total	118	100.00

- **ed004s3 : Have Visited Chinese Medical Hospital**

	No	%
3 Chinese Medicine Hospital	193	100.00
Total	193	100.00

- **ed004s4 : Have Visited Community Healthcare Center**

	No	%
4 Community Healthcare Center	149	100.00
Total	149	100.00

- **ed004s5 : Have Visited Township Hospital**

	No	%
5 Township hospital	660	100.00
Total	660	100.00

- **ed004s6 : Have Visited Health Care Post**

	No	%
6 Healthcare Post	194	100.00
Total	194	100.00

- **ed004s7 : Have Visited Village Clinic**

	No	%
7 Village clinic/Private clinic	1,342	100.00
Total	1,342	100.00

- **ed004s8 : Have Visited Other**

	No	%
8 Other	53	100.00
Total	53	100.00

- **ed005_1_ : How Many Times did You Visit the Medical Facility**

	No	%
1	664	71.09
2	149	15.95
3	68	7.28
4	17	1.82
5	13	1.39
6	6	0.64
7	1	0.11
8	4	0.43
10	7	0.75
12	2	0.21
13	1	0.11
20	1	0.11
27	1	0.11
Total	934	100.00

- **ed005_2_ : How Many Times did You Visit the Medical Facility**

	No	%
1	79	66.95
2	17	14.41
3	13	11.02
4	3	2.54
5	4	3.39
8	1	0.85
30	1	0.85
Total	118	100.00

- **ed005_3_ : How Many Times did You Visit the Medical Facility**

	No	%
1	117	62.57
2	43	22.99
3	13	6.95
4	7	3.74
5	2	1.07
6	1	0.53
7	1	0.53
10	2	1.07
13	1	0.53
Total	187	100.00

- **ed005_4_ : How Many Times did You Visit the Medical Facility**

	No	%

1	81	52.94
2	35	22.88
3	17	11.11
4	10	6.54
5	2	1.31
6	1	0.65
7	1	0.65
8	3	1.96
10	2	1.31
12	1	0.65
Total	153	100.00

- **ed005_5_ : How Many Times did You Visit the Medical Facility**

	No	%
1	380	58.55
2	136	20.96
3	70	10.79
4	21	3.24
5	13	2.00
6	4	0.62
7	3	0.46
8	4	0.62
9	2	0.31
10	9	1.39
12	1	0.15
15	2	0.31
18	1	0.15
20	1	0.15
30	2	0.31
Total	649	100.00

- **ed005_6_ : How Many Times did You Visit the Medical Facility**

	No	%
1	97	51.32
2	44	23.28
3	17	8.99
4	3	1.59
5	13	6.88
6	3	1.59
7	2	1.06
8	5	2.65
10	2	1.06
15	1	0.53
20	1	0.53
30	1	0.53
Total	189	100.00

- **ed005_7_ : How Many Times did You Visit the Medical Facility**

	No	%
1	614	47.01
2	295	22.59
3	186	14.24
4	75	5.74
5	52	3.98
6	24	1.84
7	11	0.84
8	9	0.69
9	2	0.15
10	20	1.53
12	5	0.38
14	2	0.15
15	5	0.38
20	5	0.38
30	1	0.08
Total	1,306	100.00

- **ed006_1_ : Whether Pay**

	No	%
1 Total cost yuan	300	97.40
2 Didnt pay anything	8	2.60
Total	308	100.00

- **ed006_1_1_ : Total Cost**

Mean	Min	Max	OBS
1,480.3	0.0	25,000.0	300

- **ed006_1_2_ : Total Cost**

Mean	Min	Max	OBS
3,204.9	100.0	50,000.0	51

- **ed006_1_3_ : Total Cost**

Mean	Min	Max	OBS
1,034.0	0.0	15,000.0	75

- **ed006_1_4_ : Total Cost**

Mean	Min	Max	OBS
397.6	13.0	3,000.0	73

- **ed006_1_5_ : Total Cost**

Mean	Min	Max	OBS
386.1	0.0	7,000.0	292

- **ed006_1_6_ : Total Cost**

Mean	Min	Max	OBS
184.1	10.0	1,500.0	93

- **ed006_1_7_ : Total Cost**

Mean	Min	Max	OBS
192.0	0.0	4,000.0	690

- **ed006_1_8_ : Total Cost**

Mean	Min	Max	OBS
200.0	200.0	200.0	1

- **ed006_1_9_ : Total Cost**

Mean	Min	Max	OBS
354.5	30.0	1,000.0	4

- **ed006_2_ : Whether Pay**

	No	%
1 Total cost yuan	51	98.08
2 Didnt pay anything	1	1.92
Total	52	100.00

- **ed006_3_ : Whether Pay**

	No	%
1 Total cost yuan	75	97.40
2 Didnt pay anything	2	2.60
Total	77	100.00

- **ed006_4_ : Whether Pay**

	No	%
1 Total cost yuan	73	97.33
2 Didnt pay anything	2	2.67
Total	75	100.00

- **ed006_5_ : Whether Pay**

	No	%
1 Total cost yuan	295	98.66
2 Didnt pay anything	4	1.34
Total	299	100.00

- **ed006_6_ : Whether Pay**

	No	%
1 Total cost yuan	93	95.88
2 Didnt pay anything	4	4.12
Total	97	100.00

- **ed006_7_ : Whether Pay**

	No	%
1 Total cost yuan	690	98.15
2 Didnt pay anything	13	1.85
Total	703	100.00

- **ed006_8_ : Whether Pay**

	No	%
1 Total cost yuan	1	100.00
Total	1	100.00

- **ed006_9_ : Whether Pay**

	No	%
1 Total cost yuan	4	100.00
Total	4	100.00

- **ed007_1_ : Self Paid Part or Pay Nothing**

	No	%
1 Self-paid part yuan	291	97.32
2 Didnt pay anything	8	2.68
Total	299	100.00

- **ed007_1_1_ : The Amount of Self Paid Part**

Mean	Min	Max	OBS
1,128.0	0.0	18,000.0	290

- **ed007_1_2_ : The Amount of Self Paid Part**

Mean	Min	Max	OBS
2,106.6	0.0	40,000.0	50

- **ed007_1_3_ : The Amount of Self Paid Part**

Mean	Min	Max	OBS
745.5	1.0	6,200.0	72

- **ed007_1_4_ : The Amount of Self Paid Part**

Mean	Min	Max	OBS
317.9	0.0	3,000.0	65

- **ed007_1_5_ : The Amount of Self Paid Part**

Mean	Min	Max	OBS
296.6	10.0	5,700.0	281

- **ed007_1_6_ : The Amount of Self Paid Part**

Mean	Min	Max	OBS
156.1	10.0	1,500.0	92

- **ed007_1_7_ : The Amount of Self Paid Part**

Mean	Min	Max	OBS
185.1	0.0	4,000.0	678

- **ed007_1_8_ : The Amount of Self Paid Part**

Mean	Min	Max	OBS
200.0	200.0	200.0	1

- **ed007_1_9_ : The Amount of Self Paid Part**

Mean	Min	Max	OBS
160.0	30.0	500.0	4

- **ed007_2_ : Self Paid Part or Pay Nothing**

	No	%
1 Self-paid part yuan	50	98.04
2 Didnt pay anything	1	1.96
Total	51	100.00

- **ed007_3_ : Self Paid Part or Pay Nothing**

	No	%
1 Self-paid part yuan	72	97.30
2 Didnt pay anything	2	2.70
Total	74	100.00

- **ed007_4_ : Self Paid Part or Pay Nothing**

	No	%
1 Self-paid part yuan	64	90.14
2 Didnt pay anything	7	9.86
Total	71	100.00

- **ed007_5_ : Self Paid Part or Pay Nothing**

	No	%
1 Self-paid part yuan	281	98.94
2 Didnt pay anything	3	1.06
Total	284	100.00

- **ed007_6_ : Self Paid Part or Pay Nothing**

	No	%
1 Self-paid part yuan	92	100.00
Total	92	100.00

- **ed007_7_ : Self Paid Part or Pay Nothing**

	No	%
1 Self-paid part yuan	682	98.98
2 Didnt pay anything	7	1.02
Total	689	100.00

- **ed007_8_ : Self Paid Part or Pay Nothing**

	No	%
1 Self-paid part yuan	1	100.00
Total	1	100.00

- **ed007_9_ : Self Paid Part or Pay Nothing**

	No	%
1 Self-paid part yuan	4	80.00
2 Didnt pay anything	1	20.00
Total	5	100.00

- **ed008 : The Health Care Provider Last Time**

	No	%
1 FLSelectFacility[1]	871	25.77
2 FLSelectFacility[2]	102	3.02
3 FLSelectFacility[3]	176	5.21
4 FLSelectFacility[4]	137	4.05
5 FLSelectFacility[5]	596	17.63
6 FLSelectFacility[6]	174	5.15

7 FLSelectFacility[7]	1,275	37.72
8 FLSelectFacility[8]	49	1.45
Total	3,380	100.00

- **ed009 : The Health Care Provider Public or Private**

	No	%
1 Public	1,887	89.81
2 Private	214	10.19
Total	2,101	100.00

- **ed010 : What is the Level of The Health Care Provider**

	No	%
1 county/district	867	75.65
2 Regional /city	195	17.02
3 Provincial/ affiliated to a ministry	56	4.89
4 Military	14	1.22
5 Others	11	0.96
6 Not applicable	3	0.26
Total	1,146	100.00

- **ed012 : Did the Provider Visit You at Home**

	No	%
1 Yes	150	4.43
2 No	3,236	95.57
Total	3,386	100.00

- **ed013 : How Many Kilometers is it the Provider from Your Home**

Mean	Min	Max	OBS
15.5	0.0	2,000.0	3,175

- **ed014 : Travel Time to That Facility**

Mean	Min	Max	OBS
19.1	0.0	360.0	2,680

- **ed014_1 : Unit of Time to That Facility**

Mean	Min	Max	OBS
2.4	0.0	120.0	667

- **ed014_2 : The Method to That Facility**

	No	%
1 Walk	1,465	46.08
2 Bus	739	23.25
3 Car	289	9.09
4 Ambulance	8	0.25
5 Bicycle or other manual vehicles	183	5.76
6 Electric bicycle/electric tricycle	217	6.83
7 Motorcycle	251	7.90
8 Tractor	7	0.22
9 Train	20	0.63
Total	3,179	100.00

- **ed015 : The Total Transportation Cost to the Facility**

Mean	Min	Max	OBS
13.3	0.0	900.0	1,667

- **ed016_1 : The Health Care Provider Located Province**

	No	%
1 This province	2,568	98.24
2 Other province, specify	46	1.76
Total	2,614	100.00

- **ed016_1_1 : The Health Care Provider Located Other Province**

	No	%
01	1	1.67
02	1	1.67
05	1	1.67
06	2	3.33
07	1	1.67
08	1	1.67
09	5	8.33
10	3	5.00
11	1	1.67
12	5	8.33
13	1	1.67
14	4	6.67
15	9	15.00

18	4	6.67
19	1	1.67
20	4	6.67
21	1	1.67
23	1	1.67
26	3	5.00
29	4	6.67
33	7	11.67
Total	60	100.00

- **ed016_2 : The Health Care Provider Located County**

	No	%
1 This county / city	2,625	95.80
2 Other county / city, specify	115	4.20
Total	2,740	100.00

- **ed016_2_1 : The Health Care Provider Located Other County**

	No	%
01	28	4.18
02	2	0.30
04	24	3.58
05	1	0.15
11	59	8.81
16	32	4.78
17	1	0.15
18	1	0.15
24	17	2.54
27	13	1.94
40	113	16.87
42	1	0.15
46	66	9.85
49	21	3.13
51	1	0.15
52	1	0.15
53	23	3.43
55	39	5.82
56	6	0.90
60	34	5.07
63	17	2.54
66	16	2.39
67	7	1.04
74	53	7.91
77	2	0.30
82	55	8.21
84	1	0.15
86	19	2.84
96	3	0.45

99	14	2.09
Total	670	100.00

- **ed016_3 : The Health Care Provider Located Township**

	No	%
1 This township/district	1,875	87.54
2 Other township/district	267	12.46
Total	2,142	100.00

- **ed016_4 : The Health Care Provider Located Village**

	No	%
1 This village/street	1,369	73.33
2 Other village/street	498	26.67
Total	1,867	100.00

- **ed017s1 : Visit the Health Care Provider for Immunization**

	No	%
1 Immunization	6	100.00
Total	6	100.00

- **ed017s2 : Visit the Health Care Provider for Consultation**

	No	%
2 Consultation	77	100.00
Total	77	100.00

- **ed017s3 : Visit the Health Care Provider for Medical Chech-up**

	No	%
3 Medical check-up	277	100.00
Total	277	100.00

- **ed017s4 : Visit the Health Care Provider for Treatment for Illness**

	No	%
4 Treatment for Illness	3,094	100.00
Total	3,094	100.00

- **ed017s5 : Visit the Health Care Provider for Other**

	No	%
5 Other	155	100.00
Total	155	100.00

- **ed018 : The Disease Name**

Chinese Character String

- **ed019 : A First Visit/A Follow-up Visit**

	No	%
1 First	1,572	50.91
2 Follow-up	1,516	49.09
Total	3,088	100.00

- **ed020 : Ordinary Outpatient Service/An Emergency**

	No	%
1 Normal	3,003	97.28
2 Emergency	84	2.72
Total	3,087	100.00

- **ed021s1 : Receive Injection Treatment**

	No	%
1 Injection	686	100.00
Total	686	100.00

- **ed021s2 : Receive Laboratorium Test Treatment**

	No	%
2 Laboratory test	405	100.00
Total	405	100.00

- **ed021s3 : Receive Surgery Treatment**

	No	%

3 Surgery	63	100.00
Total	63	100.00

- **ed021s4 : Receive X-ray,CT,B Ultrasonic Treatment**

	No	%
4 X-ray, CT, B ultrasonic, MRI	521	100.00
Total	521	100.00

- **ed021s5 : Receive Medications and Purchase Medical Treatment**

	No	%
5 Medications and purchase medical	2,506	100.00
Total	2,506	100.00

- **ed021s6 : Receive IV Treatment**

	No	%
6 IV Drip Infusion	1,231	100.00
Total	1,231	100.00

- **ed021s7 : Receive Traditional Treatment**

	No	%
7 Traditional treatment ,eg massage, acupuncture	85	100.00
Total	85	100.00

- **ed021s8 : Receive Other Treatment**

	No	%
8 Other	96	100.00
Total	96	100.00

- **ed022 : Wait for How Long to be Examined**

Mean	Min	Max	OBS
4.5	0.0	90.0	3,155

- **ed022_1 : Unit of Time of Waiting to be Examined**

	No	%
1 Minute	2,873	91.06
2 Hour	282	8.94
Total	3,155	100.00

- **ed023 : Cost/No Cost of the Hospital Visit**

	No	%
1 yuan	3,137	96.37
2 There was no cost	118	3.63
Total	3,255	100.00

- **ed023_1 : The Amount of Total Cost of the Hospital Visit**

Mean	Min	Max	OBS
802.3	-9,999.0	200,000.0	3,137

- **ed024 : Self Paid Part or Pay Nothing**

	No	%
1 RMB	3,041	97.09
2 Did not pay anything	91	2.91
Total	3,132	100.00

- **ed024_1 : Self Paid Amount**

Mean	Min	Max	OBS
561.4	-9,999.0	170,000.0	3,039

- **ed025 : Who Paid Most of the Hospital Visit Fee**

	No	%
1 Myself	2,643	86.94
2 Children	348	11.45
3 Relatives	17	0.56
4 Government	5	0.16
5 Rs union	11	0.36
6 Rs insurance	3	0.10
8 Donate	13	0.43
Total	3,040	100.00

- **ed026 : The Total Medication Cost or Other Situation**

	No	%
1 RMB	2,498	87.62
2 Didnt receive	318	11.15
3 Didnt fill prescription	35	1.23
Total	2,851	100.00

- **ed026_1 : The Amount of The Total Medication Cost**

Mean	Min	Max	OBS
309.0	-9,999.0	50,000.0	2,506

- **ed027 : Self Payment of The Total Medication Cost or Other Situation**

	No	%
1 RMB	2,416	97.11
2 Didnt pay anything	72	2.89
Total	2,488	100.00

- **ed027_1 : The Amount of Self Payment of The Total Medication Cost**

Mean	Min	Max	OBS
233.2	-9,999.0	10,000.0	2,410

- **ed028s1 : Did You Have Urban Employee Medical Insurance**

	No	%
1 FLInsurance_1[1]	155	100.00
Total	155	100.00

- **ed028s2 : Did You Have Urban Resident Medical Insurance**

	No	%
2 FLInsurance_1[2]	36	100.00
Total	36	100.00

- **ed028s3 : Did You Have New Cooperative Medical Insurance**

	No	%

3 FLInsurance_1[3]	661	100.00
Total	661	100.00

- **ed028s4 : Did You Have Urban and Rural Resident Medical Insurance**

	No	%
4 FLInsurance_1[4]	12	100.00
Total	12	100.00

- **ed028s5 : Did You Have Government Medical Insurance**

	No	%
5 FLInsurance_1[5]	37	100.00
Total	37	100.00

- **ed028s6 : Did You Have Medical Aid**

	No	%
6 FLInsurance_1[6]	1	100.00
Total	1	100.00

- **ed028s7 : Did You Have Private Medical Insurance:Purchased By R's Union**

	No	%
7 FLInsurance_1[7]	6	100.00
Total	6	100.00

- **ed028s8 : Did You Have Private Medical Insurance:Purchased By Individual**

	No	%
8 FLInsurance_1[8]	1	100.00
Total	1	100.00

- **ed028s9 : Did You Have Other Medical Insurance**

	No	%
9 FLInsurance_1[9]	1	100.00
Total	1	100.00

- **ed028s10 : Did You Have Reimbursed by R's Union**

	No	%
10 No insurance	188	100.00
Total	188	100.00

- **ed028s11 : Did You Have No Insurance**

	No	%
11 Reimbursed by Rs union	7	100.00
Total	7	100.00

- **ed028s12 : The Insurance Not Revelent to You**

	No	%
12 Not revelent to me	2,039	100.00
Total	2,039	100.00

- **ed029 : Y/N Give Red Envelopes to the Doctors**

	No	%
1 Yes	24	0.72
2 No	3,330	99.28
Total	3,354	100.00

- **ee001 : Y/N Need Inpatient Care But You Did Not Get**

	No	%
1 Yes	711	4.40
2 No	15,464	95.60
Total	16,175	100.00

- **ee002 : The Reason for Not Seeking Hospitalization**

	No	%
1 Not enough money	446	60.76
2 Not willing to go to the hospital	158	21.53
3 Hospital quality poor	17	2.32
4 Problem too serious	12	1.63
5 No ward available	4	0.54
6 Other	97	13.22
Total	734	100.00

- **ee003 : Have You Received Inpatient Care LY**

	No	%
1 Yes	1,587	9.06
2 No	15,923	90.94
Total	17,510	100.00

- **ee004 : How Many Times Have You Received Inpatient Care LY**

Mean	Min	Max	OBS
2.6	0.0	2,011.0	1,663

- **ee005 : Cost/No Cost of the Hospitalizations**

	No	%
1 Total cost Yuan	365	98.92
2 Didnt pay anything	4	1.08
Total	369	100.00

- **ee005_1 : The Amount of Total Cost of the Hospitalizations**

Mean	Min	Max	OBS
13,790.3	180.0	160,000.0	358

- **ee006 : Y/N Have Self Payment of Cost of the Hospitalizations**

	No	%
1 Self-paid part Yuan	338	97.13
2 Didnt pay anything	10	2.87
Total	348	100.00

- **ee006_1 : The Amount of Self Payment of Total Cost of the Hospitalizations**

Mean	Min	Max	OBS
7,667.6	0.0	140,000.0	339

- **ee007 : Whether The Hospital Same With ed011**

	No	%

1 Yes	296	52.86
2 No	264	47.14
Total	560	100.00

- **ee008 : The Type of The Facility for Your Hospitalization LY**

	No	%
1 General Hospital	783	58.61
2 Specialized hospital	92	6.89
3 Chinese Medicine Hospital	114	8.53
4 Community Healthcare Center	15	1.12
5 Township Healthcare Clinic	279	20.88
6 Other	32	2.40
	21	1.57
Total	1,336	100.00

- **ee009 : Public/Private The Facility for Your Hospitalization LY**

	No	%
1 Public	1,249	93.63
2 Private	85	6.37
Total	1,334	100.00

- **ee010 : The Level of the Facility**

	No	%
1 county/district	725	73.45
2 Regional /city	180	18.24
3 Provincial/ affiliated to a ministry	54	5.47
4 Military	16	1.62
5 Others	12	1.22
Total	987	100.00

- **ee012_1_1 : The Health Care Provider Located Province**

	No	%
1 This province	1,061	96.19
2 Other province, specify	42	3.81
Total	1,103	100.00

- **ee012_1_2 : The Health Care Provider Located Other Province**

	No	%

02	4	8.70
04	1	2.17
05	1	2.17
06	2	4.35
07	1	2.17
08	1	2.17
09	6	13.04
10	2	4.35
11	3	6.52
12	2	4.35
14	11	23.91
15	4	8.70
16	1	2.17
17	1	2.17
18	2	4.35
20	1	2.17
26	1	2.17
28	1	2.17
32	1	2.17
Total	46	100.00

- **ee012_2_1 : The Health Care Provider Located County**

	No	%
1 This county / city	1,103	92.69
2 Other county / city, specify	87	7.31
Total	1,190	100.00

- **ee012_2_2 : The Health Care Provider Located Other County**

	No	%
01	24	6.52
02	2	0.54
04	12	3.26
11	28	7.61
16	15	4.08
18	1	0.27
24	16	4.35
27	7	1.90
40	76	20.65
42	2	0.54
46	36	9.78
49	7	1.90
51	3	0.82
52	1	0.27
53	20	5.43
55	15	4.08
56	1	0.27
60	13	3.53

63	12	3.26
66	10	2.72
67	7	1.90
74	16	4.35
82	30	8.15
83	1	0.27
84	1	0.27
86	11	2.99
99	1	0.27
Total	368	100.00

- **ee012_3_1 : The Health Care Provider Located Township**

	No	%
1 This township/district	514	75.48
2 2 Other township/district	167	24.52
Total	681	100.00

- **ee012_4_1 : The Health Care Provider Located Village**

	No	%
1 This village/street	166	39.52
2 Other village/street	254	60.48
Total	420	100.00

- **ee013 : How Many Kilometers is it the Provider from Your Home**

Mean	Min	Max	OBS
51.7	0.0	3,000.0	1,305

- **ee014 : Travel Time to that Facility**

Mean	Min	Max	OBS
15.9	0.0	90.0	1,304

- **ee014_1 : Unit of Time Travel to That Facility**

	No	%
1 Minute	876	67.18
2 Hour	428	32.82
Total	1,304	100.00

- **ee014_2 : The Method Travel to That Facility**

	No	%
1 Walk	187	14.33
2 Bus	503	38.54
3 Car	345	26.44
4 Ambulance	53	4.06
5 Bicycle or other manual vehicles	29	2.22
6 Electric bicycle/electric tricycle	76	5.82
7 Motorcycle	85	6.51
8 Tractor	2	0.15
9 Train	25	1.92
Total	1,305	100.00

- **ee015 : The Total Transportation Cost to the Facility**

Mean	Min	Max	OBS
45.2	0.0	8,000.0	1,070

- **ee016 : Nights of Hospitalized There**

Mean	Min	Max	OBS
11.9	0.0	120.0	1,613

- **ee017_1 : Year of the Starting Date of Your Hospital Stay**

Mean	Min	Max	OBS
2,010.4	1,997.0	2,012.0	1,411

- **ee017_2 : Month of the Starting Date of Your Hospital Stay**

	No	%
0	65	4.64
1	87	6.21
2	100	7.14
3	140	10.00
4	126	9.00
5	137	9.79
6	138	9.86
7	168	12.00
8	89	6.36
9	67	4.79
10	102	7.29
11	88	6.29

12	93	6.64
Total	1,400	100.00

- **ee017_3 : Day of the Starting Date of Your Hospital Stay**

	No	%
0	516	36.86
1	102	7.29
2	36	2.57
3	32	2.29
4	36	2.57
5	28	2.00
6	35	2.50
7	36	2.57
8	29	2.07
9	17	1.21
10	59	4.21
11	24	1.71
12	37	2.64
13	22	1.57
14	16	1.14
15	46	3.29
16	31	2.21
17	21	1.50
18	28	2.00
19	17	1.21
20	54	3.86
21	17	1.21
22	14	1.00
23	23	1.64
24	15	1.07
25	22	1.57
26	14	1.00
27	21	1.50
28	20	1.43
29	13	0.93
30	15	1.07
31	4	0.29
Total	1,400	100.00

- **ee018 : Still There/Exit**

	No	%
1 year month day	1,368	98.21
2 still there	25	1.79
Total	1,393	100.00

- **ee018_1 : The Year of Exit**

Mean	Min	Max	OBS
2,010.4	1,998.0	2,019.0	1,366

- **ee018_2 : The Month of Exit**

	No	%
0	60	4.39
1	80	5.86
2	99	7.25
3	117	8.57
4	125	9.15
5	136	9.96
6	127	9.30
7	166	12.15
8	107	7.83
9	68	4.98
10	92	6.73
11	94	6.88
12	95	6.95
Total	1,366	100.00

- **ee018_3 : The Day of Exit**

	No	%
0	458	33.90
1	28	2.07
2	31	2.29
3	23	1.70
4	31	2.29
5	32	2.37
6	27	2.00
7	38	2.81
8	39	2.89
9	24	1.78
10	33	2.44
11	25	1.85
12	23	1.70
13	31	2.29
14	33	2.44
15	62	4.59
16	32	2.37
17	24	1.78
18	29	2.15
19	22	1.63
20	47	3.48
21	25	1.85
22	21	1.55

23	24	1.78
24	19	1.41
25	34	2.52
26	25	1.85
27	24	1.78
28	28	2.07
29	16	1.18
30	31	2.29
31	12	0.89
Total	1,351	100.00

- **ee019 : The Reason for Hospitalization**

	No	%
1 Sickness	1,390	87.37
2 Accident	173	10.87
3 Violence	9	0.57
4 Other	19	1.19
Total	1,591	100.00

- **ee020 : The Name of the Disease**

Chinese Character String

- **ee021s1 : Receive Medical Check-up/Consultation Treatment During Hospitalization**

	No	%
1 Medical check-up/consultation	592	100.00
Total	592	100.00

- **ee021s2 : Receive Injection Treatment During Hospitalization**

	No	%
2 Injection	756	100.00
Total	756	100.00

- **ee021s3 : Receive Laboratory Test Treatment During Hospitalization**

	No	%
3 Laboratory test	807	100.00
Total	807	100.00

- **ee021s4 : Receive Surgery Treatment During Hospitalization**

	No	%
4 Surgery	393	100.00
Total	393	100.00

- **ee021s5 : Receive X-ray,CT,B Ultrasonic ,MRI Treatment During Hospitalization**

	No	%
5 X-ray, CT, B ultrasonic, MRI	893	100.00
Total	893	100.00

- **ee021s6 : Receive Medications Treatment During Hospitalization**

	No	%
6 Medications and purchase medical	1,292	100.00
Total	1,292	100.00

- **ee021s7 : Receive IV Treatment During Hospitalization**

	No	%
7 IV Drip Infusion	1,394	100.00
Total	1,394	100.00

- **ee021s8 : Receive Traditional Treatment Treatment During Hospitalization**

	No	%
8 Traditional treatment ,eg massage, acupuncture	81	100.00
Total	81	100.00

- **ee021s9 : Receive Delivery Treatment During Hospitalization**

	No	%
9 Delivery	1	100.00
Total	1	100.00

- **ee021s10 : Receive Other Treatment During Hospitalization**

	No	%
10 Other	26	100.00
Total	26	100.00

- **ee022 : Reasons Leave Hospital**

	No	%
1 Fully recovered from illness, received doctors approval	550	40.44
2 Didnt recover from illness, but received doctors suggestion to leave	324	23.82
3 Didnt recover from illness, requested to leave without doctors suggestion	453	33.31
4 Other reasons	33	2.43
Total	1,360	100.00

- **ee023 : Reasons Leave Hospital Before Recovered**

	No	%
1 Cant recover from illness	55	11.98
2 Poor	282	61.44
3 No space in the hospital	2	0.44
4 Limited hospital conditions	19	4.14
5 Poor quality and service from health care providers	5	1.09
6 Other reasons	96	20.92
Total	459	100.00

- **ee024 : Total Medical Cost for Hospitalization/No Cost**

	No	%
1 yuan	1,514	99.08
2 There was no cost	14	0.92
Total	1,528	100.00

- **ee024_1 : The Amount of Total Medical Cost for Hospitalization**

Mean	Min	Max	OBS
7,398.2	40.0	160,000.0	1,513

- **ee025 : Total Cost for Hired Nurse/No Cost**

	No	%
1 yuan	252	16.28

	No	%
2 There was no cost	1,296	83.72
Total	1,548	100.00

- **ee025_1 : The Amount of Total Cost for Hired Nurse**

Mean	Min	Max	OBS
290.7	0.0	10,000.0	261

- **ee026 : Total Cost for Transportation, Food, Accommodation/No Cost**

	No	%
1 yuan	948	68.15
2 There was no cost	443	31.85
Total	1,391	100.00

- **ee026_1 : The Amount of Total Cost for Transportation, Food, Accommodation**

Mean	Min	Max	OBS
1,063.8	-9,999.0	30,000.0	950

- **ee027 : Total Pay Out of Pocket for Cost of Hospitalization/No Cost**

	No	%
1 RMB	1,428	95.07
2 Didnt pay anything	74	4.93
Total	1,502	100.00

- **ee027_1 : The Amount of Total Pay Out of Pocket for Cost of Hospitalization**

Mean	Min	Max	OBS
4,897.5	0.0	200,000.0	1,428

- **ee028 : Who Contributes Most for Cost for Pay Out of Pocket Cost**

	No	%
1 Myself	1,024	64.69
2 Children	487	30.76
3 Relatives	12	0.76
4 Government	4	0.25

5 Rs union	24	1.52
6 Loan	6	0.38
8 Others Specify :	26	1.64
Total	1,583	100.00

- **ee029 : Total Medical Cost for Visit/No Cost**

	No	%
1 RMB	925	89.20
2 Didnt receive	112	10.80
Total	1,037	100.00

- **ee029_1 : The Amount of Total Medical Cost for Visit**

Mean	Min	Max	OBS
3,582.4	-9,999.0	70,000.0	924

- **ee030 : Total Pay Out of Pocket of Medical Cost for Hospitalization/No Cost**

	No	%
1 RMB	859	97.06
2 Didnt receive	26	2.94
Total	885	100.00

- **ee030_1 : The Amount of Total Pay Out of Pocket of Medical Cost for Hospitalization**

Mean	Min	Max	OBS
2,318.3	-9,999.0	60,000.0	855

- **ee031s1 : Did/Will You Use Urban Employee Medical Insurance**

	No	%
1 FLInsurance_1[1]	216	100.00
Total	216	100.00

- **ee031s2 : Did/Will You Use Urban Resident Medical Insurance**

	No	%

	No	%
2 FLInsurance_1[2]	59	100.00
Total	59	100.00

- **ee031s3 : Did/Will You Use New Cooperative Medical Insurance**

	No	%
3 FLInsurance_1[3]	922	100.00
Total	922	100.00

- **ee031s4 : Did/Will You Use Urban and Rural Resident Medical Insurance**

	No	%
4 FLInsurance_1[4]	16	100.00
Total	16	100.00

- **ee031s5 : Did/Will You Use Government Medical Insurance**

	No	%
5 FLInsurance_1[5]	42	100.00
Total	42	100.00

- **ee031s6 : Did/Will You Use Medical Aid**

	No	%
6 FLInsurance_1[6]	4	100.00
Total	4	100.00

- **ee031s7 : Did/Will You Use Private Medical Insurance: Purchased By R's Union**

	No	%
7 FLInsurance_1[7]	5	100.00
Total	5	100.00

- **ee031s8 : Did/Will You Use Private Medical Insurance: Purchased By Individual**

	No	%
8 FLInsurance_1[8]	7	100.00
Total	7	100.00

- **ee031s9 : Did/Will You Use Other Medical Insurance**

	No	%
9 FLInsurance_1[9]	3	100.00
Total	3	100.00

- **ee031s10 : Did/Will You Use Reimbursed by R's Union**

	No	%
10 No insurance	53	100.00
Total	53	100.00

- **ee031s11 : Did/Will You Use No Insurance**

	No	%
11 Reimbursed by Rs union	24	100.00
Total	24	100.00

- **ee031s12 : The Insurance Not Revelent to You**

	No	%
12 Not revelent to me	230	100.00
Total	230	100.00

- **ee032 : Whether Pay Any Red Bags**

	No	%
1 Yes	35	2.22
2 No	1,540	97.78
Total	1,575	100.00

- **ef001s1 : LY Treat Yourself By Consumed Over-the-counter Modern Medicines**

	No	%
1 Consumed over-the-counter modern medicines	5,359	100.00
Total	5,359	100.00

- **ef001s2 : LY Treat Yourself By Consumed Prescription Medicines**

	No	%
2 Consumed prescription medicines	2,326	100.00
Total	2,326	100.00

- **ef001s3 : LY Treat Yourself By Consumed Traditional Herbs/Medicines**

	No	%
3 Consumed traditional herbs or traditional medicines as treatment	1,013	100.00
Total	1,013	100.00

- **ef001s4 : LY Treat Yourself By Tonic/Health Supplement**

	No	%
4 Vitamin/ Refresher	593	100.00
Total	593	100.00

- **ef001s5 : LY Treat Yourself By Use Health Care Equipment**

	No	%
5 Use health care equipment	68	100.00
Total	68	100.00

- **ef001s6 : LY Treat Yourself By Other**

	No	%
6 Other	147	100.00
Total	147	100.00

- **ef001s7 : LY Treat Yourself By None**

	No	%
7 None	9,085	100.00
Total	9,085	100.00

- **ef002_1_ : The Total Cost for Consumed Over-the-counter Modern Medicines/No Cost**

	No	%

1 RMB	5,163	97.30
2 There was no cost	143	2.70
Total	5,306	100.00

- **ef002_1_1_ : The Amount of The Total Cost for Consumed Over-the-counter Modern Medicines**

Mean	Min	Max	OBS
118.2	0.0	5,600.0	5,068

- **ef002_1_2_ : The Amount of The Total Cost for Consumed Prescription Medicines**

Mean	Min	Max	OBS
186.4	0.0	6,000.0	2,144

- **ef002_1_3_ : The Amount of The Total Cost for Consumed Traditional Herbs/Medicines**

Mean	Min	Max	OBS
184.5	0.0	2,000.0	881

- **ef002_1_4_ : The Amount of The Total Cost for Tonic/Health Supplement**

Mean	Min	Max	OBS
134.6	0.0	2,000.0	481

- **ef002_1_5_ : The Amount of The Total Cost for Use Health Care Equipment**

Mean	Min	Max	OBS
518.8	0.0	5,000.0	41

- **ef002_1_6_ : The Amount of The Total Cost for Other**

Mean	Min	Max	OBS
238.3	30.0	1,000.0	6

- **ef002_2_ : The Total Cost for Consumed Prescription Medicines/No Cost**

	No	%
1 RMB	2,197	96.36
2 There was no cost	83	3.64
Total	2,280	100.00

- **ef002_3_ : The Total Cost for /No Cost Consumed Traditional Herbs/Medicines**

	No	%
1 RMB	891	90.55
2 There was no cost	93	9.45
Total	984	100.00

- **ef002_4_ : The Total Cost for /No Cost Tonic/Health Supplement**

	No	%
1 RMB	484	87.84
2 There was no cost	67	12.16
Total	551	100.00

- **ef002_5_ : The Total Cost for /No Cost Use Health Care Equipment**

	No	%
1 RMB	42	67.74
2 There was no cost	20	32.26
Total	62	100.00

- **ef002_6_ : The Total Cost for /No Cost Other**

	No	%
1 RMB	10	100.00
Total	10	100.00

- **ef003_1_ : Pay Out of Pocket Cost for Over-the-counter Modern Medicines/No Cost**

	No	%
1 RMB	4,820	95.41
2 Didnt pay anything	232	4.59
Total	5,052	100.00

- **ef003_1_1_ : The Amount of Pay Out of Pocket Cost for Over-the-counter Modern Medicines**

Mean	Min	Max	OBS
111.4	0.0	5,600.0	4,818

- **ef003_1_2_ : The Amount of The Pay Out of Pocket Cost for Consumed Prescription Medicines**

Mean	Min	Max	OBS
172.7	0.0	6,000.0	2,046

- **ef003_1_3_ : The Amount of Pay Out of Pocket Cost for Traditional Herbs/Medicines**

Mean	Min	Max	OBS
182.1	0.0	2,000.0	847

- **ef003_1_4_ : The Amount of The Pay Out of Pocket Cost for Tonic/Health Supplement**

Mean	Min	Max	OBS
135.2	0.0	2,000.0	432

- **ef003_1_5_ : The Amount of The Pay Out of Pocket Cost for Use Health Care Equipment**

Mean	Min	Max	OBS
508.2	0.0	5,000.0	39

- **ef003_1_6_ : The Amount of The Pay Out of Pocket Cost for Other Self Treatment**

	No	%
50	2	66.67
100	1	33.33
Total	3	100.00

- **ef003_2_ : The Pay Out of Pocket Cost for Consumed Prescription Medicines/No**

Cost

	No	%
1 RMB	2,047	96.28
2 Didnt pay anything	79	3.72
Total	2,126	100.00

- **ef003_3_ : Pay Out of Pocket Cost for Traditional Herbs/Medicines/No Cost**

	No	%
1 RMB	848	96.58
2 Didnt pay anything	30	3.42
Total	878	100.00

- **ef003_4_ : Pay Out of Pocket Cost for Tonic/Health Supplement/No Cost**

	No	%
1 RMB	433	90.21
2 Didnt pay anything	47	9.79
Total	480	100.00

- **ef003_5_ : The Total Cost for Use Health Care Equipment/No Cost**

	No	%
1 RMB	39	95.12
2 Didnt pay anything	2	4.88
Total	41	100.00

- **ef003_6_ : The Pay Out of Pocket Cost for Other Self Treatment/No Cost**

	No	%
1 RMB	3	75.00
2 Didnt pay anything	1	25.00
Total	4	100.00

- **ef004_1_ : Who Pay the Premium for Consumed Over-the-counter Modern Medicines**

	No	%
1 Myself	4,422	91.80
2 Children	343	7.12

3 Relatives	24	0.50
4 Government	3	0.06
5 Rs union	9	0.19
6 Loan	3	0.06
8 Others Specify :	13	0.27
Total	4,817	100.00

- **ef004_2_ : Who Pay the Premium for Consumed Prescription Medicines**

	No	%
1 Myself	1,804	88.17
2 Children	207	10.12
3 Relatives	14	0.68
4 Government	4	0.20
5 Rs union	4	0.20
6 Loan	1	0.05
8 Others Specify :	12	0.59
Total	2,046	100.00

- **ef004_3_ : Who Pay the Premium for Consumed Consumed Traditional Herbs/Medicines**

	No	%
1 Myself	744	87.84
2 Children	87	10.27
3 Relatives	11	1.30
6 Loan	1	0.12
8 Others Specify :	4	0.47
Total	847	100.00

- **ef004_4_ : Who Pay the Premium for Tonic/Health Supplement**

	No	%
1 Myself	338	78.24
2 Children	81	18.75
3 Relatives	5	1.16
4 Government	1	0.23
5 Rs union	3	0.69
6 Loan	1	0.23
8 Others Specify :	3	0.69
Total	432	100.00

- **ef004_5_ : Who Pay the Premium for Use Health Care Equipment**

	No	%

1 Myself	29	74.36
2 Children	9	23.08
8 Others Specify :	1	2.56
Total	39	100.00

- **ef004_6_ : Who Pay the Premium for Other Self Treatment**

	No	%
1 Myself	3	100.00
Total	3	100.00

- **ef005_1_s1 : Insurance Used**

	No	%
1 FLInsurance_1[1]	256	100.00
Total	256	100.00

- **ef005_1_s2 : Insurance Used**

	No	%
2 FLInsurance_1[2]	33	100.00
Total	33	100.00

- **ef005_1_s3 : Insurance Used**

	No	%
3 FLInsurance_1[3]	422	100.00
Total	422	100.00

- **ef005_1_s4 : Insurance Used**

	No	%
4 FLInsurance_1[4]	16	100.00
Total	16	100.00

- **ef005_1_s5 : Insurance Used**

	No	%
5 FLInsurance_1[5]	47	100.00
Total	47	100.00

- **ef005_1_s6 : Insurance Used**

No Observations

- **ef005_1_s7 : Insurance Used**

	No	%
7 FLInsurance_1[7]	4	100.00
Total	4	100.00

- **ef005_1_s8 : Insurance Used**

	No	%
8 FLInsurance_1[8]	2	100.00
Total	2	100.00

- **ef005_1_s9 : Insurance Used**

	No	%
9 FLInsurance_1[9]	2	100.00
Total	2	100.00

- **ef005_1_s10 : Insurance Used**

	No	%
10 No insurance	391	100.00
Total	391	100.00

- **ef005_1_s11 : Insurance Used**

	No	%
11 Reimbursed by Rs union	13	100.00
Total	13	100.00

- **ef005_1_s12 : Insurance Used**

	No	%
12 Not revelent to me	3,892	100.00
Total	3,892	100.00

- **ef005_2_s1 : Insurance Used**

	No	%
1 FLInsurance_1[1]	129	100.00
Total	129	100.00

- **ef005_2_s2 : Insurance Used**

	No	%
2 FLInsurance_1[2]	18	100.00
Total	18	100.00

- **ef005_2_s3 : Insurance Used**

	No	%
3 FLInsurance_1[3]	384	100.00
Total	384	100.00

- **ef005_2_s4 : Insurance Used**

	No	%
4 FLInsurance_1[4]	4	100.00
Total	4	100.00

- **ef005_2_s5 : Insurance Used**

	No	%
5 FLInsurance_1[5]	23	100.00
Total	23	100.00

- **ef005_2_s6 : Insurance Used**

No Observations

- **ef005_2_s7 : Insurance Used**

	No	%
7 FLInsurance_1[7]	4	100.00
Total	4	100.00

- **ef005_2_s8 : Insurance Used**

	No	%
8 FLInsurance_1[8]	1	100.00
Total	1	100.00

- **ef005_2_s9 : Insurance Used**

No Observations

- **ef005_2_s10 : Insurance Used**

	No	%
10 No insurance	151	100.00
Total	151	100.00

- **ef005_2_s11 : Insurance Used**

	No	%
11 Reimbursed by Rs union	2	100.00
Total	2	100.00

- **ef005_2_s12 : Insurance Used**

	No	%
12 Not revelent to me	1,431	100.00
Total	1,431	100.00

- **ef005_3_s1 : Insurance Used**

	No	%
1 FLInsurance_1[1]	39	100.00
Total	39	100.00

- **ef005_3_s2 : Insurance Used**

	No	%
2 FLInsurance_1[2]	3	100.00
Total	3	100.00

- **ef005_3_s3 : Insurance Used**

	No	%
3 FLInsurance_1[3]	69	100.00
Total	69	100.00

- **ef005_3_s4 : Insurance Used**

	No	%
4 FLInsurance_1[4]	1	100.00
Total	1	100.00

- **ef005_3_s5 : Insurance Used**

	No	%
5 FLInsurance_1[5]	8	100.00
Total	8	100.00

- **ef005_3_s6 : Insurance Used**

No Observations

- **ef005_3_s7 : Insurance Used**

	No	%
7 FLInsurance_1[7]	1	100.00
Total	1	100.00

- **ef005_3_s8 : Insurance Used**

No Observations

- **ef005_3_s9 : Insurance Used**

No Observations

- **ef005_3_s10 : Insurance Used**

	No	%
10 No insurance	72	100.00
Total	72	100.00

- **ef005_3_s11 : Insurance Used**

	No	%
11 Reimbursed by Rs union	2	100.00
Total	2	100.00

- **ef005_3_s12 : Insurance Used**

	No	%
12 Not revelent to me	682	100.00
Total	682	100.00

- **ef005_4_s1 : Insurance Used**

	No	%
1 FLInsurance_1[1]	38	100.00
Total	38	100.00

- **ef005_4_s2 : Insurance Used**

	No	%
2 FLInsurance_1[2]	6	100.00
Total	6	100.00

- **ef005_4_s3 : Insurance Used**

	No	%
3 FLInsurance_1[3]	22	100.00
Total	22	100.00

- **ef005_4_s4 : Insurance Used**

No Observations

- **ef005_4_s5 : Insurance Used**

	No	%
5 FLInsurance_1[5]	9	100.00
Total	9	100.00

- **ef005_4_s6 : Insurance Used**

No Observations

- **ef005_4_s7 : Insurance Used**

No Observations

- **ef005_4_s8 : Insurance Used**

No Observations

- **ef005_4_s9 : Insurance Used**

No Observations

- **ef005_4_s10 : Insurance Used**

	No	%
10 No insurance	21	100.00
Total	21	100.00

- **ef005_4_s11 : Insurance Used**

	No	%
11 Reimbursed by Rs union	6	100.00
Total	6	100.00

- **ef005_4_s12 : Insurance Used**

	No	%
12 Not revelent to me	377	100.00
Total	377	100.00

- **ef005_5_s1 : Insurance Used**

	No	%
1 FLInsurance_1[1]	2	100.00
Total	2	100.00

- **ef005_5_s2 : Insurance Used**

No Observations

- **ef005_5_s3 : Insurance Used**

	No	%
3 FLInsurance_1[3]	6	100.00
Total	6	100.00

- **ef005_5_s4 : Insurance Used**

No Observations

- **ef005_5_s5 : Insurance Used**

No Observations

- **ef005_5_s6 : Insurance Used**

No Observations

- **ef005_5_s7 : Insurance Used**

No Observations

- **ef005_5_s8 : Insurance Used**

No Observations

- **ef005_5_s9 : Insurance Used**

No Observations

- **ef005_5_s10 : Insurance Used**

	No	%
10 No insurance	6	100.00
Total	6	100.00

- **ef005_5_s11 : Insurance Used**

	No	%
11 Reimbursed by Rs union	1	100.00
Total	1	100.00

- **ef005_5_s12 : Insurance Used**

	No	%
12 Not revelent to me	27	100.00
Total	27	100.00

- **ef005_6_s1 : Insurance Used**

	No	%
1 FLInsurance_1[1]	1	100.00
Total	1	100.00

- **ef005_6_s2 : Insurance Used**

No Observations

- **ef005_6_s3 : Insurance Used**

No Observations

- **ef005_6_s4 : Insurance Used**

No Observations

- **ef005_6_s5 : Insurance Used**

No Observations

- **ef005_6_s6 : Insurance Used**

No Observations

- **ef005_6_s7 : Insurance Used**

No Observations

- **ef005_6_s8 : Insurance Used**

No Observations

- **ef005_6_s9 : Insurance Used**

No Observations

- **ef005_6_s10 : Insurance Used**

No Observations

- **ef005_6_s11 : Insurance Used**

No Observations

- **ef005_6_s12 : Insurance Used**

	No	%
12 Not revelent to me	4	100.00
Total	4	100.00

- **ef006 : Respondent Receive Assistance In Answering Section D**

	No	%
1 Never	13,420	76.60
2 A few times	3,122	17.82
3 Most or all the time	542	3.09
4 The section was done by a proxy reporter	436	2.49
Total	17,520	100.00

- **ef007 : Your Relationship to R**

	No	%
1 Spouse	300	67.72
2 Mother	4	0.90
3 Father	5	1.13
4 Mother-in-law	2	0.45
5 Father-in-law	1	0.23
6 Sibling	6	1.35
7 Brother-in-law, sister-in-law	2	0.45
8 Child	77	17.38
9 Spouse of child	32	7.22
10 Grandchild	8	1.81
11 Other relative	3	0.68
12 Helper or other non-relative	3	0.68
Total	443	100.00

- **ef008 : The Reason for Proxy**

	No	%
1 The respondent has serious physical handicaps	59	13.47
2 The respondent has serious mental handicaps	22	5.02
3 The respondent has rejected this interview	43	9.82
4 Other	314	71.69
Total	438	100.00

- **proxy : Interview Down By Proxy**

	No	%
0 No	16,214	92.45
1 Yes	1,325	7.55
Total	17,539	100.00

7 Work, Retirement and Pension

- **ID : Individual ID**

A String Variable	
OBS:	17,524

- **householdID : Household ID**

A String Variable	
OBS:	17,524

- **communityID : Community ID**

A String Variable	
OBS:	17,524

- **fa001 : Engaged in Agricultural Work for More than 10 days**

	No	%
1 Yes	9,215	52.61
2 No	8,300	47.39
Total	17,515	100.00

- **fa002 : Work for at Least One Hour Last Week**

	No	%
1 Yes	2,565	30.46
2 No	5,856	69.54
Total	8,421	100.00

- **fa003 : Have a Job but are Temporarily Laid-off, on Sick or other Leave**

	No	%
1 Yes	93	1.58
2 No	5,789	98.42
Total	5,882	100.00

- **fa004_1 : Year**

Mean	Min	Max	OBS

2,008.9	1,995.0	2,012.0	79

- **fa004_2 : Month**

	No	%
0	6	7.69
1	8	10.26
2	1	1.28
3	6	7.69
4	1	1.28
5	5	6.41
6	10	12.82
7	26	33.33
8	6	7.69
9	1	1.28
10	3	3.85
11	1	1.28
12	4	5.13
Total	78	100.00

- **fa005 : Expect to Go Back to this Job in the Future**

	No	%
1 Yes	36	50.70
2 No	35	49.30
Total	71	100.00

- **fa006 : Still Receive Any Salary from this Job**

	No	%
1 Yes	13	36.11
2 No	23	63.89
Total	36	100.00

- **fa007 : Ever Worked for At Least three months**

	No	%
1 Yes	5,273	91.23
2 No	507	8.77
Total	5,780	100.00

- **fa008 : Are You Sure**

	No	%
1 Yes, never worked before	383	71.59
2 No, ever worked	152	28.41
Total	535	100.00

- **fa009 : The Reason for Not Work**

	No	%
1 Disabled physical or psychological	37	10.22
2 Homemaker	268	74.03
3 My family is too rich that I dont need to work	2	0.55
5 Other	55	15.19
Total	362	100.00

- **fb001 : Age or Year Start Working**

	No	%
1 Age: years	15,477	91.99
2 year [IWER: Mark the year in four digits.]	1,347	8.01
Total	16,824	100.00

- **fb001_1 : Age**

	No	%
1	6	0.04
3	1	0.01
4	7	0.05
5	15	0.10
6	70	0.45
7	139	0.90
8	263	1.70
9	133	0.86
10	431	2.78
11	205	1.32
12	943	6.09
13	1,105	7.13
14	1,197	7.73
15	2,170	14.01
16	2,303	14.87
17	1,571	10.14
18	1,783	11.51
19	638	4.12
20	1,204	7.77
21	248	1.60
22	256	1.65

23	205	1.32
24	141	0.91
25	155	1.00
26	59	0.38
27	36	0.23
28	27	0.17
29	7	0.05
30	76	0.49
31	10	0.06
32	13	0.08
33	4	0.03
34	5	0.03
35	5	0.03
36	3	0.02
37	3	0.02
38	3	0.02
39	3	0.02
40	16	0.10
41	3	0.02
42	1	0.01
43	2	0.01
44	1	0.01
45	5	0.03
46	1	0.01
47	1	0.01
49	1	0.01
50	3	0.02
52	3	0.02
53	1	0.01
55	1	0.01
57	1	0.01
60	2	0.01
61	1	0.01
64	1	0.01
84	1	0.01
Total	15,488	100.00

- **fb001_2 : Year**

Mean	Min	Max	OBS
1,973.1	1,934.0	2,011.0	1,368

- **fb002 : Type of Work Unit of Your First Job**

	No	%
1 Government	329	2.08
2 Institutions	778	4.91
3 NGO	48	0.30
4 Firm	1,647	10.39

5 Individual firm	386	2.44
6 Farmer	11,953	75.43
7 Individual household	72	0.45
8 Other	634	4.00
Total	15,847	100.00

- **fb003 : Ownership Type of the Business**

	No	%
1 100% State owned firm	996	60.22
2 State-controlled firm	21	1.27
3 100% Collective-owned firm	503	30.41
4 Collective-controlled firm	18	1.09
5 100% Private firm	79	4.78
6 Private-controlled firm	19	1.15
7 100% foreign-owned	2	0.12
8 Joint venture	1	0.06
9 Other joint-ownership	2	0.12
10 Other	13	0.79
Total	1,654	100.00

- **fb004 : Location of First Workplace**

	No	%
1 This village/community	8,704	54.96
2 Other village/community in this county/city	5,717	36.10
3 Another county/city in this province	807	5.10
4 Another province	609	3.85
Total	15,837	100.00

- **fb004_1 : City**

	No	%
01	42	5.18
02	3	0.37
04	35	4.32
05	15	1.85
11	53	6.54
16	20	2.47
17	8	0.99
18	4	0.49
24	59	7.27
27	26	3.21
28	4	0.49
35	2	0.25
40	135	16.65
42	7	0.86

46	70	8.63
49	16	1.97
50	1	0.12
51	5	0.62
52	3	0.37
53	41	5.06
55	33	4.07
56	6	0.74
60	41	5.06
63	30	3.70
66	2	0.25
74	46	5.67
76	2	0.25
77	6	0.74
82	66	8.14
86	16	1.97
96	9	1.11
99	5	0.62
Total	811	100.00

- **fb004_2 : County**

	No	%
02	55	7.34
03	13	1.74
04	30	4.01
06	55	7.34
07	2	0.27
08	6	0.80
13	3	0.40
16	15	2.00
19	3	0.40
28	30	4.01
31	65	8.68
33	6	0.80
37	6	0.80
38	2	0.27
39	9	1.20
43	39	5.21
44	11	1.47
45	1	0.13
46	42	5.61
49	4	0.53
50	1	0.13
51	15	2.00
54	32	4.27
56	2	0.27
57	5	0.67
58	3	0.40
59	57	7.61
63	117	15.62

73	1	0.13
75	3	0.40
76	59	7.88
78	5	0.67
81	25	3.34
90	6	0.80
91	12	1.60
92	6	0.80
99	3	0.40
Total	749	100.00

- **fb004_3 : Province**

	No	%
01	7	1.16
02	2	0.33
03	12	2.00
04	3	0.50
05	54	8.99
06	41	6.82
07	15	2.50
08	12	2.00
09	17	2.83
10	20	3.33
11	27	4.49
12	34	5.66
13	10	1.66
14	40	6.66
15	13	2.16
16	38	6.32
17	16	2.66
18	10	1.66
19	8	1.33
20	6	1.00
21	36	5.99
23	2	0.33
24	22	3.66
25	5	0.83
26	11	1.83
27	17	2.83
28	8	1.33
29	52	8.65
32	23	3.83
33	27	4.49
34	13	2.16
Total	601	100.00

- **fb004_4 : City**

	No	%
01	23	3.89
02	2	0.34
04	31	5.25
05	4	0.68
11	39	6.60
16	11	1.86
17	1	0.17
18	5	0.85
24	62	10.49
27	17	2.88
28	9	1.52
38	1	0.17
40	124	20.98
46	31	5.25
48	1	0.17
49	20	3.38
51	3	0.51
52	1	0.17
53	34	5.75
55	37	6.26
56	1	0.17
60	17	2.88
63	12	2.03
66	2	0.34
74	15	2.54
76	1	0.17
82	48	8.12
83	1	0.17
84	1	0.17
86	17	2.88
88	2	0.34
96	1	0.17
99	17	2.88
Total	591	100.00

- **fb004_5 : County**

	No	%
02	28	5.61
03	3	0.60
04	22	4.41
06	38	7.62
07	3	0.60
08	1	0.20
13	2	0.40
16	2	0.40
19	1	0.20
23	7	1.40
28	1	0.20

31	43	8.62
33	6	1.20
37	3	0.60
38	1	0.20
39	14	2.81
43	32	6.41
44	3	0.60
45	5	1.00
46	18	3.61
49	4	0.80
50	1	0.20
51	10	2.00
54	17	3.41
56	5	1.00
57	5	1.00
58	3	0.60
59	57	11.42
63	100	20.04
73	2	0.40
75	1	0.20
76	28	5.61
78	10	2.00
81	13	2.61
83	1	0.20
90	1	0.20
91	3	0.60
92	3	0.60
99	2	0.40
Total	499	100.00

- **fb005 : Ever Worked in a State Owned, State Controlled or Collectively Owned Enterprise**

	No	%
1 Yes	1,383	9.66
2 No	12,941	90.34
Total	14,324	100.00

- **fb006 : Specific Ownership Type**

	No	%
1 100% State owned firm	788	55.81
2 State-controlled firm	46	3.26
3 100% Collective-owned firm	557	39.45
4 Collective-controlled firm	21	1.49
Total	1,412	100.00

- **fb007 : Stop Working for an Extended Period of Time**

	No	%
1 Yes	805	5.08
2 No	15,029	94.92
Total	15,834	100.00

- **fb008_1 : Years**

	No	%
0	38	4.59
1	267	32.25
2	156	18.84
3	119	14.37
4	46	5.56
5	37	4.47
6	26	3.14
7	16	1.93
8	16	1.93
9	6	0.72
10	27	3.26
11	9	1.09
12	13	1.57
13	10	1.21
14	5	0.60
15	7	0.85
16	5	0.60
17	3	0.36
18	1	0.12
20	7	0.85
21	1	0.12
22	1	0.12
24	1	0.12
25	1	0.12
27	2	0.24
28	1	0.12
30	1	0.12
35	1	0.12
40	2	0.24
48	2	0.24
50	1	0.12
Total	828	100.00

- **fb008_2 : Months**

	No	%
0	617	74.97
1	28	3.40

2	22	2.67
3	27	3.28
4	10	1.22
5	11	1.34
6	82	9.96
7	7	0.85
8	8	0.97
9	2	0.24
10	5	0.61
11	4	0.49
Total	823	100.00

- **fb009_2 : Beginning Year**

Mean	Min	Max	OBS
1,993.1	1,946.0	2,011.0	756

- **fb009_3 : Begining Month**

	No	%
0	281	37.17
1	84	11.11
2	38	5.03
3	54	7.14
4	25	3.31
5	37	4.89
6	39	5.16
7	43	5.69
8	31	4.10
9	43	5.69
10	20	2.65
11	22	2.91
12	39	5.16
Total	756	100.00

- **fb009_5 : Ending Year**

Mean	Min	Max	OBS
1,997.0	1,949.0	2,012.0	755

- **fb009_6 : Ending Month**

	No	%
0	282	37.35
1	48	6.36

2	32	4.24
3	28	3.71
4	34	4.50
5	27	3.58
6	52	6.89
7	88	11.66
8	54	7.15
9	33	4.37
10	25	3.31
11	18	2.38
12	34	4.50
Total	755	100.00

- **fb010 : Reason for Interruptions**

	No	%
1 Family	130	16.03
2 Health	537	66.21
4 School	24	2.96
5 Unemployment/layoff	25	3.08
6 Other	95	11.71
Total	811	100.00

- **fb011 : Processed Retirement**

	No	%
1 Yes	2,089	12.20
2 No	15,036	87.80
Total	17,125	100.00

- **fb012 : Completed Receding Position Procedures**

	No	%
1 Yes	124	0.82
2 No	14,952	99.18
Total	15,076	100.00

- **fc001 : Worked for Other Famers**

	No	%
1 Yes	617	6.71
2 No	8,572	93.29
Total	9,189	100.00

- **fc002 : Num. of Employers Worked for in the Past Month**

Mean	Min	Max	OBS
2.8	0.0	400.0	650

- **fc003 : Workplace for Most Time**

	No	%
1 This village/community	517	77.98
2 Other village/community in this county/city	127	19.16
3 Another county/city in this province	13	1.96
4 Another province	6	0.90
Total	663	100.00

- **fc003_1 : City**

	No	%
11	1	7.69
16	1	7.69
40	2	15.38
46	2	15.38
49	3	23.08
51	1	7.69
74	1	7.69
82	1	7.69
96	1	7.69
Total	13	100.00

- **fc003_2 : County**

	No	%
02	1	8.33
04	2	16.67
06	3	25.00
51	1	8.33
54	1	8.33
57	1	8.33
63	2	16.67
76	1	8.33
Total	12	100.00

- **fc003_3 : Province**

	No	%

08	2	33.33
10	1	16.67
11	1	16.67
16	1	16.67
23	1	16.67
Total	6	100.00

- **fc003_4 : City**

	No	%
04	1	16.67
05	1	16.67
18	1	16.67
53	2	33.33
82	1	16.67
Total	6	100.00

- **fc003_5 : County**

	No	%
43	1	16.67
46	1	16.67
63	4	66.67
Total	6	100.00

- **fc004 : Months Worked on Cropping, Livestock, and Fishing for other Famers**

	No	%
0	81	13.00
1	252	40.45
2	74	11.88
3	52	8.35
4	19	3.05
5	12	1.93
6	35	5.62
7	4	0.64
8	18	2.89
9	8	1.28
10	12	1.93
11	11	1.77
12	45	7.22
Total	623	100.00

- **fc005 : Days Worked Per Week on Average**

	No	%
0	6	1.13
1	35	6.59
2	36	6.78
3	50	9.42
4	47	8.85
5	60	11.30
6	37	6.97
7	260	48.96
Total	531	100.00

- **fc006 : Hours Worked Per Day on Average**

	No	%
0	7	1.32
1	1	0.19
2	2	0.38
3	10	1.89
4	14	2.64
5	10	1.89
6	22	4.15
7	16	3.02
8	159	30.00
9	58	10.94
10	142	26.79
11	16	3.02
12	64	12.08
13	5	0.94
14	1	0.19
15	3	0.57
Total	530	100.00

- **fc007 : Average Monthly Wage**

Mean	Min	Max	OBS
1,130.6	0.0	30,000.0	595

- **fc008 : Worked for Your Own Household**

	No	%
1 Yes	7,956	86.60
2 No	1,231	13.40
Total	9,187	100.00

- **fc009 : Months Worked on Cropping etc. for Own Household**

	No	%
0	44	0.55
1	694	8.73
2	891	11.20
3	706	8.88
4	466	5.86
5	381	4.79
6	950	11.95
7	294	3.70
8	499	6.27
9	261	3.28
10	511	6.43
11	146	1.84
12	2,110	26.53
Total	7,953	100.00

- **fc010 : Num. of Days Worked Per Week**

	No	%
0	24	0.31
1	189	2.42
2	366	4.68
3	619	7.91
4	529	6.76
5	810	10.35
6	526	6.72
7	4,762	60.86
Total	7,825	100.00

- **fc011 : Num. of Hours Worked Per Day**

	No	%
0	17	0.22
1	112	1.44
2	338	4.34
3	485	6.23
4	674	8.65
5	660	8.47
6	960	12.32
7	557	7.15
8	1,827	23.45
9	317	4.07
10	1,418	18.20
11	87	1.12
12	260	3.34
13	31	0.40

14	21	0.27
15	11	0.14
16	7	0.09
18	3	0.04
19	2	0.03
20	2	0.03
24	1	0.01
Total	7,790	100.00

- **fc012 : Location of Workplace**

	No	%
1 This village/community	7,627	96.76
2 Other village/community in this county/city	196	2.49
3 Another county/city in this province	34	0.43
4 Another province	25	0.32
Total	7,882	100.00

- **fc012_1 : City**

	No	%
01	2	5.71
04	1	2.86
05	1	2.86
11	1	2.86
16	1	2.86
24	1	2.86
40	13	37.14
42	1	2.86
46	3	8.57
50	2	5.71
53	1	2.86
55	1	2.86
60	1	2.86
63	2	5.71
74	2	5.71
83	1	2.86
86	1	2.86
Total	35	100.00

- **fc012_2 : County**

	No	%
02	1	3.33
04	2	6.67
06	3	10.00
08	2	6.67

28	1	3.33
31	1	3.33
37	1	3.33
43	1	3.33
46	2	6.67
50	1	3.33
54	2	6.67
59	1	3.33
63	8	26.67
76	2	6.67
99	2	6.67
Total	30	100.00

- **fc012_3 : Province**

	No	%
01	1	3.85
08	1	3.85
09	5	19.23
14	1	3.85
15	7	26.92
18	1	3.85
19	2	7.69
20	1	3.85
23	1	3.85
26	1	3.85
27	1	3.85
28	1	3.85
29	1	3.85
32	1	3.85
33	1	3.85
Total	26	100.00

- **fc012_4 : City**

	No	%
01	1	4.17
04	1	4.17
11	2	8.33
16	1	4.17
24	1	4.17
40	13	54.17
55	1	4.17
66	1	4.17
74	1	4.17
82	1	4.17
96	1	4.17
Total	24	100.00

- **fc012_5 : County**

	No	%
13	2	13.33
37	1	6.67
39	2	13.33
57	2	13.33
59	1	6.67
63	6	40.00
78	1	6.67
Total	15	100.00

- **fc013 : Num. of Days Missed due to Health Problems**

Mean	Min	Max	OBS
16.7	0.0	365.0	8,977

- **fc014 : Worked in Wage or Self-Employed Work or Unpaid Family Business**

	No	%
1 Yes	1,706	18.63
2 No	7,452	81.37
Total	9,158	100.00

- **fc015 : Temporarily Laid-off**

	No	%
1 Yes	88	1.18
2 No	7,391	98.82
Total	7,479	100.00

- **fc016_1 : Beginning Year**

Mean	Min	Max	OBS
2,009.4	1,965.0	2,011.0	75

- **fc016_2 : Beginning Month**

	No	%
0	4	5.26
1	2	2.63

2	2	2.63
3	7	9.21
4	4	5.26
5	8	10.53
6	18	23.68
7	24	31.58
9	2	2.63
10	2	2.63
12	3	3.95
Total	76	100.00

- **fc017 : Expect to Go Back to Job in the Future**

	No	%
1 Yes	56	77.78
2 No	16	22.22
Total	72	100.00

- **fc018 : Age Plan to Stop Working**

Mean	Min	Max	OBS
19.4	0.0	2,016.0	6,980

- **fc019 : Have More than One Job**

	No	%
1 Yes	181	3.97
2 No	4,375	96.03
Total	4,556	100.00

- **fc020 : Main Job**

	No	%
1 Employed	137	68.16
2 Self-employed	51	25.37
3 unpaid family business	13	6.47
Total	201	100.00

- **fc021 : Describe Non-Agricultural Job**

	No	%
1 Employed	2,953	68.20
2 Self-employed	1,104	25.50

3 unpaid family business	273	6.30
Total	4,330	100.00

- **fd001 : Receive Wage from**

	No	%
1 Place of work	2,828	90.70
2 Dispatch Company	290	9.30
Total	3,118	100.00

- **fd002 : Type of the Work Unit**

	No	%
1 Government	179	5.88
2 Institutions	383	12.59
3 NGO	25	0.82
4 Firm	876	28.80
5 Individual firm	1,211	39.81
6 Farmer	126	4.14
7 Individual household	100	3.29
8 Other	142	4.67
Total	3,042	100.00

- **fd004 : Location of Workplace**

	No	%
1 This village/community	1,159	38.78
2 Other village/community in this county/city	1,361	45.53
3 Another county/city in this province	252	8.43
4 Another province	217	7.26
Total	2,989	100.00

- **fd004_1 : City**

	No	%
01	8	4.68
04	10	5.85
05	3	1.75
11	7	4.09
16	1	0.58
24	11	6.43
27	2	1.17
40	51	29.82
42	2	1.17
46	17	9.94

49	3	1.75
51	1	0.58
52	2	1.17
53	9	5.26
55	6	3.51
60	9	5.26
66	5	2.92
74	7	4.09
76	1	0.58
82	8	4.68
83	3	1.75
84	2	1.17
86	1	0.58
96	1	0.58
99	1	0.58
Total	171	100.00

- **fd004_2 : County**

	No	%
02	6	4.00
04	13	8.67
06	11	7.33
08	5	3.33
16	5	3.33
28	8	5.33
31	18	12.00
37	4	2.67
39	3	2.00
43	9	6.00
44	1	0.67
46	2	1.33
51	1	0.67
54	12	8.00
56	5	3.33
57	2	1.33
58	2	1.33
59	8	5.33
63	8	5.33
75	1	0.67
76	3	2.00
78	6	4.00
81	2	1.33
89	1	0.67
90	1	0.67
91	1	0.67
92	10	6.67
99	2	1.33
Total	150	100.00

- **fd004_3 : Province**

	No	%
04	1	1.06
05	1	1.06
06	9	9.57
07	3	3.19
08	2	2.13
09	8	8.51
10	2	2.13
11	9	9.57
12	1	1.06
14	2	2.13
15	16	17.02
17	3	3.19
18	9	9.57
19	9	9.57
20	6	6.38
23	2	2.13
24	1	1.06
26	2	2.13
28	1	1.06
29	2	2.13
32	2	2.13
33	2	2.13
34	1	1.06
Total	94	100.00

- **fd004_4 : City**

	No	%
01	5	7.25
04	5	7.25
05	1	1.45
07	1	1.45
11	7	10.14
16	1	1.45
24	5	7.25
27	1	1.45
38	1	1.45
40	20	28.99
53	8	11.59
55	3	4.35
60	3	4.35
63	3	4.35
74	1	1.45
82	2	2.90
83	1	1.45
84	1	1.45
Total	69	100.00

- **fd004_5 : County**

	No	%
02	1	2.08
03	2	4.17
04	2	4.17
06	1	2.08
08	2	4.17
13	5	10.42
16	1	2.08
23	1	2.08
28	2	4.17
31	2	4.17
37	3	6.25
38	1	2.08
39	1	2.08
43	2	4.17
44	1	2.08
46	1	2.08
54	4	8.33
56	2	4.17
59	5	10.42
63	4	8.33
76	2	4.17
78	2	4.17
99	1	2.08
Total	48	100.00

- **fd005_indc : Employed Industry Code**

	No	%
1	98	3.29
10	28	0.94
11	45	1.51
12	30	1.01
13	15	0.50
14	45	1.51
15	104	3.49
16	150	5.03
17	59	1.98
18	24	0.80
19	251	8.42
2	73	2.45
3	980	32.86
4	37	1.24
5	696	23.34
6	116	3.89
7	7	0.23
8	136	4.56
9	88	2.95

Total	2,982	100.00

- **fd006 : Civil Servant**

	No	%
1 Yes	48	26.82
2 No	131	73.18
Total	179	100.00

- **fd007 : Formal Employee of an Establishment**

	No	%
1 Yes	90	50.28
2 No	89	49.72
Total	179	100.00

- **fd008 : As Firm or Government Unit**

	No	%
1 As a firm	118	32.07
2 As a government unit	250	67.93
Total	368	100.00

- **fd009 : Regular Worker**

	No	%
1 Yes	269	70.60
2 No	112	29.40
Total	381	100.00

- **fd010 : Ownership Type of the Business**

	No	%
1 100% State owned firm	207	24.67
2 State-controlled firm	31	3.69
3 100% Collective-owned firm	72	8.58
4 Collective-controlled firm	24	2.86
5 100% Private firm	359	42.79
6 Private-controlled firm	111	13.23
7 100% foreign-owned	11	1.31
8 Joint venture	9	1.07
9 Other joint-ownership	4	0.48
10 Other	11	1.31

Total	839	100.00

- **fd011_1 : Began Year**

Mean	Min	Max	OBS
2,000.9	1,905.0	2,012.0	2,918

- **fd011_2 : Began Month**

	No	%
0	837	28.68
1	268	9.18
2	208	7.13
3	274	9.39
4	162	5.55
5	170	5.83
6	207	7.09
7	268	9.18
8	146	5.00
9	122	4.18
10	94	3.22
11	62	2.12
12	100	3.43
Total	2,918	100.00

- **fd012_gb : Employed Occupation Code (GB)**

	No	%
10202	1	0.03
10203	1	0.03
10205	8	0.27
10209	2	0.07
10302	2	0.07
10303	37	1.24
10401	4	0.13
10402	4	0.13
10403	2	0.07
10409	4	0.13
10510	6	0.20
10520	46	1.54
10530	22	0.74
10544	4	0.13
10546	2	0.07
20118	1	0.03
20201	1	0.03
20203	3	0.10
20206	1	0.03

20207	2	0.07
20213	1	0.03
20214	2	0.07
20215	1	0.03
20216	1	0.03
20222	1	0.03
20223	5	0.17
20225	1	0.03
20226	5	0.17
20229	2	0.07
20235	2	0.07
20236	3	0.10
20301	1	0.03
20501	22	0.74
20505	1	0.03
20506	6	0.20
20507	4	0.13
20508	3	0.10
20601	1	0.03
20602	3	0.10
20603	59	1.98
20701	3	0.10
20702	4	0.13
20803	1	0.03
20901	9	0.30
20902	2	0.07
20903	35	1.17
20904	49	1.64
20905	7	0.23
20909	3	0.10
21003	4	0.13
21004	1	0.03
21007	1	0.03
21009	1	0.03
21100	2	0.07
21202	1	0.03
21205	1	0.03
21206	4	0.13
21209	1	0.03
30101	81	2.72
30102	60	2.01
30201	17	0.57
30202	127	4.26
30209	1	0.03
30301	1	0.03
30303	4	0.13
40101	55	1.84
40102	15	0.50
40103	5	0.17
40105	18	0.60
40107	1	0.03
40109	1	0.03

40201	25	0.84
40202	9	0.30
40209	1	0.03
40301	87	2.92
40305	39	1.31
40401	10	0.34
40402	1	0.03
40403	2	0.07
40501	12	0.40
40502	7	0.23
40504	1	0.03
40600	1	0.03
40701	3	0.10
40702	7	0.23
40703	22	0.74
40704	1	0.03
40707	3	0.10
40708	4	0.13
40711	5	0.17
40712	1	0.03
40713	30	1.01
40714	156	5.23
40715	2	0.07
40719	6	0.20
40900	1	0.03
50101	31	1.04
50103	35	1.17
50104	1	0.03
50105	2	0.07
50106	6	0.20
50201	3	0.10
50202	2	0.07
50204	6	0.20
50301	4	0.13
50302	5	0.17
50305	6	0.20
50401	2	0.07
50402	2	0.07
50403	2	0.07
50501	1	0.03
50502	1	0.03
50504	1	0.03
60103	30	1.01
60104	12	0.40
60105	4	0.13
60106	3	0.10
60201	2	0.07
60202	9	0.30
60203	2	0.07
60205	2	0.07
60208	3	0.10
60301	4	0.13

60303	1	0.03
60304	10	0.34
60305	6	0.20
60309	1	0.03
60312	3	0.10
60317	2	0.07
60401	36	1.21
60402	21	0.70
60404	1	0.03
60405	7	0.23
60406	3	0.10
60409	1	0.03
60501	2	0.07
60502	4	0.13
60503	2	0.07
60504	3	0.10
60506	3	0.10
60507	3	0.10
60508	2	0.07
60511	3	0.10
60512	33	1.11
60519	6	0.20
60601	42	1.41
60701	3	0.10
60702	3	0.10
60705	3	0.10
60706	27	0.91
60801	4	0.13
60802	2	0.07
60803	2	0.07
60901	5	0.17
60902	31	1.04
61001	9	0.30
61002	10	0.34
61003	20	0.67
61004	10	0.34
61005	1	0.03
61009	1	0.03
61101	62	2.08
61102	27	0.91
61103	6	0.20
61109	1	0.03
61201	6	0.20
61202	2	0.07
61203	4	0.13
61204	4	0.13
61205	6	0.20
61206	1	0.03
61207	1	0.03
61501	12	0.40
61502	13	0.44
61503	55	1.84

61505	4	0.13
61506	8	0.27
61601	7	0.23
61602	31	1.04
61605	3	0.10
61607	1	0.03
61701	2	0.07
61702	2	0.07
61703	1	0.03
61704	8	0.27
61901	1	0.03
61902	4	0.13
61903	2	0.07
61909	1	0.03
62001	4	0.13
62003	8	0.27
62004	1	0.03
62005	7	0.23
62006	1	0.03
62007	1	0.03
62009	24	0.80
62101	5	0.17
62201	16	0.54
62202	379	12.71
62203	38	1.27
62204	12	0.40
62205	5	0.17
62206	1	0.03
62207	55	1.84
62209	38	1.27
62211	2	0.07
62301	84	2.82
62302	2	0.07
62304	5	0.17
62305	10	0.34
62309	2	0.07
62403	4	0.13
62501	18	0.60
62504	4	0.13
62901	45	1.51
62902	2	0.07
62903	199	6.67
Total	2,982	100.00

- **fd012_isco : Employed Occupation Code (ISCO)**

	No	%
1100	1	0.03
1140	1	0.03
1142	2	0.07
1143	37	1.24

1200	10	0.34
1210	20	0.67
1220	46	1.54
1230	22	0.74
1314	4	0.13
1316	2	0.07
2122	3	0.10
2130	2	0.07
2141	6	0.20
2143	1	0.03
2144	1	0.03
2145	7	0.23
2146	1	0.03
2147	4	0.13
2149	8	0.27
2211	1	0.03
2213	2	0.07
2221	23	0.77
2224	6	0.20
2230	3	0.10
2310	9	0.30
2320	37	1.24
2331	49	1.64
2332	7	0.23
2359	3	0.10
2411	59	1.98
2421	1	0.03
2431	4	0.13
2441	1	0.03
2444	1	0.03
2450	1	0.03
2451	1	0.03
2452	1	0.03
2453	1	0.03
2455	4	0.13
3110	4	0.13
3150	18	0.60
3220	4	0.13
3227	6	0.20
3412	4	0.13
3416	5	0.17
3419	3	0.10
3430	81	2.72
3439	1	0.03
3470	1	0.03
3475	2	0.07
4110	60	2.01
4130	1	0.03
4131	25	0.84
4133	9	0.30
4142	1	0.03
4210	4	0.13

5000	1	0.03
5110	20	0.67
5111	1	0.03
5121	29	0.97
5122	87	2.92
5123	39	1.31
5130	30	1.01
5132	22	0.74
5141	1	0.03
5143	2	0.07
5149	7	0.23
5162	17	0.57
5169	128	4.29
5200	1	0.03
5220	70	2.35
6111	31	1.04
6113	38	1.27
6121	4	0.13
6122	5	0.17
6141	11	0.37
6151	2	0.07
6152	2	0.07
7111	37	1.24
7121	16	0.54
7122	379	12.71
7123	38	1.27
7129	12	0.40
7130	5	0.17
7134	1	0.03
7141	55	1.84
7210	58	1.95
7213	1	0.03
7224	10	0.34
7230	50	1.68
7240	35	1.17
7241	9	0.30
7245	3	0.10
7300	24	0.80
7313	5	0.17
7321	4	0.13
7322	1	0.03
7324	8	0.27
7330	9	0.30
7331	1	0.03
7340	8	0.27
7400	5	0.17
7410	24	0.80
7412	2	0.07
7413	4	0.13
7430	21	0.70
7431	9	0.30
7432	22	0.74

7433	89	2.98
7434	6	0.20
7436	7	0.23
8112	12	0.40
8121	15	0.50
8122	3	0.10
8141	25	0.84
8143	4	0.13
8150	22	0.74
8160	2	0.07
8161	3	0.10
8212	38	1.27
8220	3	0.10
8229	2	0.07
8231	5	0.17
8232	31	1.04
8240	55	1.84
8253	8	0.27
8271	2	0.07
8280	4	0.13
8281	40	1.34
8282	5	0.17
8283	6	0.20
8290	4	0.13
8300	2	0.07
8310	2	0.07
8320	84	2.82
8334	10	0.34
8340	5	0.17
9000	199	6.67
9161	22	0.74
9162	156	5.23
9312	41	1.37
9322	45	1.51
Total	2,982	100.00

- **fd013 : Your Current Position**

	No	%
1 Clerk/worker	2,166	72.08
2 Team Leader	69	2.30
3 Ke	72	2.40
4 Chu	15	0.50
5 Ju and above	3	0.10
6 Village Leader	54	1.80
7 Township Leader	10	0.33
8 Division manager	22	0.73
9 Overall manager	9	0.30
10 Others	585	19.47
Total	3,005	100.00

- **fd014 : Your Current Professional/Technical Level**

	No	%
1 Technician	78	2.60
2 Primary level	60	2.00
3 Intermediate level	135	4.50
4 Advanced level	111	3.70
5 No professional/technical level	2,619	87.21
Total	3,003	100.00

- **fd015 : In a Postion to Supervise Others**

	No	%
1 Yes	149	5.41
2 No	2,604	94.59
Total	2,753	100.00

- **fd016 : Num. of People under Your Supervise**

	No	%
1 1 5 people	114	29.16
2 6 10 people	80	20.46
3 11 15 people	38	9.72
4 16 30 people	48	12.28
5 31 99 people	49	12.53
6 More than 100 people	62	15.86
Total	391	100.00

- **fd017 : Employment Type**

	No	%
1 Regular worker	241	8.61
2 Contract worker	926	33.07
3 Casual worker	1,633	58.32
Total	2,800	100.00

- **fd018 : Personnel File**

	No	%
1 Yes	782	27.28
2 No	2,085	72.72
Total	2,867	100.00

- **fd019 : Where is Your Personnel File Kept**

	No	%
1 With my current employer	497	64.05
2 Other work unit but not current employer	83	10.70
3 With the Job Service Center in this city	127	16.37
4 HuKou place in other city	62	7.99
5 Other city	7	0.90
Total	776	100.00

- **fd020 : Receive a Labor Contract**

	No	%
1 Yes	694	23.57
2 No	2,251	76.43
Total	2,945	100.00

- **fd021 : Agreed Period of Employment**

	No	%
1 Defined period years and months	333	48.54
2 Not defined	339	49.42
3 Same as the term of the project	14	2.04
Total	686	100.00

- **fd021_1 : Years**

	No	%
0	6	1.87
1	147	45.79
2	34	10.59
3	80	24.92
4	3	0.93
5	32	9.97
6	1	0.31
8	1	0.31
10	7	2.18
27	1	0.31
28	1	0.31
30	1	0.31
32	2	0.62
35	1	0.31
36	1	0.31
42	2	0.62
60	1	0.31
Total	321	100.00

- **fd021_2 : Months**

	No	%
0	301	96.17
1	2	0.64
3	2	0.64
5	1	0.32
6	4	1.28
8	1	0.32
9	1	0.32
11	1	0.32
Total	313	100.00

- **fd022 : Employment Contract Ever Been Renewed**

	No	%
1 Yes	242	72.67
2 No	91	27.33
Total	333	100.00

- **fd023 : Times the Contract been Renewed**

	No	%
1	17	8.10
2	59	28.10
3	49	23.33
4	19	9.05
5	19	9.05
6	11	5.24
7	11	5.24
8	5	2.38
9	1	0.48
10	4	1.90
11	2	0.95
12	1	0.48
14	1	0.48
15	2	0.95
16	1	0.48
17	1	0.48
20	2	0.95
21	1	0.48
26	1	0.48
30	2	0.95
50	1	0.48
Total	210	100.00

- **fd024 : How Long You Expect to Work at Current Workplace**

	No	%
1 Less than one year	864	32.85
2 One to two years	352	13.38
3 Two to three years	236	8.97
4 More than three years	1,178	44.79
Total	2,630	100.00

- **fd025 : Why You Expect So**

	No	%
1 Because the predefined contract period will expire	26	2.14
2 Because typically the contract expires although theres no written contract	44	3.62
3 Because I was hired under the condition that I would resign upon the request of employer	34	2.80
4 Because the current job/project will be completed	222	18.26
5 Because the person I am substituting will return to work	3	0.25
6 Because I can only work during certain seasons	45	3.70
7 Because I plan to find another job that better suits my job aptitude, ability, and preference	92	7.57
8 Because I will reach at retirement age set by regulations/practice	119	9.79
9 Because of family care responsibility, poor health, etc.	255	20.97
10 Other	376	30.92
Total	1,216	100.00

- **fd026 : Work Unit Going to Process Retirement for You**

	No	%
1 Yes	522	18.75
2 No	2,262	81.25
Total	2,784	100.00

- **fd027 : Age You will Process Retirement**

	No	%
45	2	0.39
46	1	0.19
50	55	10.64
51	5	0.97
52	1	0.19
53	1	0.19
54	2	0.39
55	110	21.28
56	1	0.19
58	1	0.19
59	1	0.19
60	317	61.32
61	10	1.93

62	1	0.19
65	7	1.35
70	2	0.39
Total	517	100.00

- **fd028 : Processed Retirement through this Work Unit**

	No	%
1 Yes	75	39.89
2 No	113	60.11
Total	188	100.00

- **fd029 : Num. of Days of Paid Vacation**

Mean	Min	Max	OBS
3.7	0.0	365.0	2,952

- **fd030 : Num. of Days Missed due to Health Problems**

Mean	Min	Max	OBS
2.4	0.0	365.0	2,930

- **fd031 : Num. of Days Not Deduct Wage or Bonus**

Mean	Min	Max	OBS
7.2	0.0	365.0	324

- **fe001 : Months Worked in the Past Year**

	No	%
0	211	7.11
1	125	4.21
2	103	3.47
3	109	3.67
4	104	3.51
5	94	3.17
6	176	5.93
7	93	3.14
8	146	4.92
9	113	3.81
10	265	8.93
11	158	5.33
12	1,269	42.78

Total	2,966	100.00

- **fe002 : Days Per Week Worked in the Past Year**

	No	%
0	137	4.69
1	33	1.13
2	43	1.47
3	71	2.43
4	138	4.72
5	618	21.16
6	384	13.15
7	1,497	51.25
Total	2,921	100.00

- **fe003 : Hours Per Day Worked in the Past Year**

	No	%
0	119	4.08
1	6	0.21
2	16	0.55
3	28	0.96
4	56	1.92
5	71	2.43
6	108	3.70
7	116	3.97
8	1,250	42.81
9	245	8.39
10	519	17.77
11	71	2.43
12	225	7.71
13	26	0.89
14	23	0.79
15	11	0.38
16	15	0.51
20	2	0.07
24	13	0.45
Total	2,920	100.00

- **ff001 : How is Your Wage Paid**

	No	%
1 Yearly salary	204	6.85
2 Monthly salary	1,775	59.62
3 Weekly salary	5	0.17
4 Daily salary	510	17.13
5 Hourly salary	28	0.94

6 Contract-based	143	4.80
7 Performance-based	173	5.81
8 Other	139	4.67
Total	2,977	100.00

- **ff002 : After-Tax Salary in the Last Year**

Mean	Min	Max	OBS
15,466.1	0.0	300,000.0	197

- **ff003_a : Min Bracket**

Mean	Min	Max	OBS
12,857.4	0.0	50,000.0	7

- **ff003_b : Max Bracket**

Mean	Min	Max	OBS
42,877,140.7	9,999.0	99,999,996.0	7

- **ff004 : After-Tax Salary in the Last Month**

Mean	Min	Max	OBS
1,689.5	0.0	15,000.0	1,748

- **ff005_a : Min Bracket**

Mean	Min	Max	OBS
1,145.5	0.0	5,000.0	31

- **ff005_b : Max Bracket**

Mean	Min	Max	OBS
25,808,143.7	499.0	99,999,996.0	31

- **ff006 : Wage in the Last Week**

Mean	Min	Max	OBS
490.0	200.0	1,000.0	5

- **ff007_a : Min Bracket**

No Observations

- **ff007_b : Max Bracket**

No Observations

- **ff008 : Usual Daily Wage**

Mean	Min	Max	OBS
76.9	0.0	1,000.0	512

- **ff009_a : Min Bracket**

	No	%
50	1	50.00
51	1	50.00
Total	2	100.00

- **ff009_b : Max Bracket**

	No	%
50	1	50.00
99	1	50.00
Total	2	100.00

- **ff010 : Hourly Wage**

Mean	Min	Max	OBS
10.2	2.0	45.0	28

- **ff011_a : Min Bracket**

No Observations

- **ff011_b : Max Bracket**

No Observations

- **ff012 : How Much on Average You Received Last Month**

Mean	Min	Max	OBS
1,519.1	0.0	199,990.0	960

- **ff013_a : Min Bracket**

Mean	Min	Max	OBS
648.8	0.0	5,000.0	37

- **ff013_b : Max Bracket**

Mean	Min	Max	OBS
27,028,701.1	999.0	99,999,996.0	37

- **ff014 : Value of All Ohter Bonuses You Received Last Year**

Mean	Min	Max	OBS
3,812.1	0.0	150,000.0	2,907

- **ff015_a : Min Bracket**

Mean	Min	Max	OBS
2,195.0	0.0	20,001.0	77

- **ff015_b : Max Bracket**

Mean	Min	Max	OBS
44,158,647.3	999.0	99,999,996.0	77

- **fg001s1 : Fringe Benefits**

	No	%
1 Free lunch	681	100.00
Total	681	100.00

- **fg001s2 : Fringe Benefits**

	No	%
2 Free breakfast	350	100.00
Total	350	100.00

- **fg001s3 : Fringe Benefits**

	No	%
3 Free dinner	394	100.00
Total	394	100.00

- **fg001s4 : Fringe Benefits**

	No	%
4 Meal cash subsidy	99	100.00
Total	99	100.00

- **fg001s5 : Fringe Benefits**

	No	%
5 Transportation cash subsidizations	88	100.00
Total	88	100.00

- **fg001s6 : Fringe Benefits**

	No	%
6 Free housing	375	100.00
Total	375	100.00

- **fg001s7 : Fringe Benefits**

	No	%
7 Subsidization of housing	50	100.00
Total	50	100.00

- **fg001s8 : Fringe Benefits**

	No	%
8 Company car	18	100.00
Total	18	100.00

- **fg001s9 : Fringe Benefits**

	No	%
9 Company bus	50	100.00
Total	50	100.00

- **fg001s10 : Fringe Benefits**

	No	%
10 Other subsidies	45	100.00
Total	45	100.00

- **fg001s11 : Fringe Benefits**

	No	%
11 None	1,953	100.00
Total	1,953	100.00

- **fg002_1_ : The Value of Subsidy**

Mean	Min	Max	OBS
108.8	0.0	600.0	623

- **fg002_2_ : The Value of Subsidy**

Mean	Min	Max	OBS
68.6	0.0	300.0	305

- **fg002_3_ : The Value of Subsidy**

Mean	Min	Max	OBS
104.5	0.0	1,000.0	340

- **fg002_4_ : The Value of Subsidy**

Mean	Min	Max	OBS
167.5	0.0	1,200.0	95

- **fg002_5_ : The Value of Subsidy**

Mean	Min	Max	OBS
151.4	0.0	2,000.0	75

- **fg002_6_ : The Value of Subsidy**

Mean	Min	Max	OBS
228.3	0.0	2,000.0	274

- **fg002_7_ : The Value of Subsidy**

Mean	Min	Max	OBS
309.4	0.0	1,500.0	41

- **fg002_8_ : The Value of Subsidy**

Mean	Min	Max	OBS
14,542.9	0.0	200,000.0	14

- **fg002_9_ : The Value of Subsidy**

Mean	Min	Max	OBS
133.9	0.0	2,000.0	47

- **fg002_10_ : The Value of Subsidy**

Mean	Min	Max	OBS
113.9	0.0	1,000.0	44

- **fg003s1 : Does Your Employer Provide Insurance**

	No	%

1 Pension	300	100.00
Total	300	100.00

- **fg003s2 : Does Your Employer Provide Insurance**

	No	%
2 Health insurance	278	100.00
Total	278	100.00

- **fg003s3 : Does Your Employer Provide Insurance**

	No	%
3 Unemployment insurance	163	100.00
Total	163	100.00

- **fg003s4 : Does Your Employer Provide Insurance**

	No	%
4 Workers injury insurance	284	100.00
Total	284	100.00

- **fg003s5 : Does Your Employer Provide Insurance**

	No	%
5 Maternity insurance	91	100.00
Total	91	100.00

- **fg003s6 : Does Your Employer Provide Insurance**

	No	%
6 None	2,022	100.00
Total	2,022	100.00

- **fg004s1 : The Reason for Not Having Insurance**

	No	%
1 There is no social insurance through employment at my local area	530	100.00
Total	530	100.00

- **fg004s2 : The Reason for Not Having Insurance**

	No	%
2 Social insurance through work is available at my local area but my employer does not provide it to me	1,154	10[illegible]
Total	1,154	1[illegible]

- **fg004s3 : The Reason for Not Having Insurance**

	No	%
3 My employer offers it but I am unwilling to join	25	100.00
Total	25	100.00

- **fg004s4 : The Reason for Not Having Insurance**

	No	%
4 I am in the trial period	80	100.00
Total	80	100.00

- **fg004s5 : The Reason for Not Having Insurance**

	No	%
5 I have social insurance from elsewhere	43	100.00
Total	43	100.00

- **fg004s6 : The Reason for Not Having Insurance**

	No	%
6 I have passed retirement age	50	100.00
Total	50	100.00

- **fg005 : From Where do You Have Insurance**

	No	%
1 I am covered through another work unit	37	77.08
2 I contribute through job service center	11	22.92
Total	48	100.00

- **fg006 : Why do You Contribute Insurance through Another Work Unit**

	No	%
1 I was laid-off by this work unit but employment contract is not terminated	11	35.48

	No	%
2 I am on leave from this employer	2	6.45
5 Other	18	58.06
Total	31	100.00

- **fg007 : Where is This Work Unit Located**

	No	%
1 Same city	27	96.43
2 Different city, same province	1	3.57
Total	28	100.00

- **fg008 : Who Pays**

	No	%
2 Myself	9	100.00
Total	9	100.00

- **fg009 : Is the Salary the Net Amount**

	No	%
1 Yes	332	66.53
2 No	167	33.47
Total	499	100.00

- **fg010 : Income Base**

Mean	Min	Max	OBS
1,297.3	0.0	15,000.0	300

- **fg011 : Do You Know How Much You or Your Employer Contributes**

	No	%
1 I know the amount by myself and the employer	126	27.88
2 I know the amount by myself but not the employer	111	24.56
3 I do not know either my contribution or my employers contribution at all.	215	47.57
Total	452	100.00

- **fg012 : Employer's Contribution**

Mean	Min	Max	OBS
283.8	0.0	3,710.0	112

- **fg013 : Your Own Contribution**

Mean	Min	Max	OBS
226.6	0.0	6,300.0	216

- **fg014 : Employer Provides Funding for Public Housing**

	No	%
1 Yes	146	5.86
2 No	2,346	94.14
Total	2,492	100.00

- **fg015 : Age You Plan to Stop Working**

	No	%
0	1,382	59.24
2	1	0.04
32	1	0.04
45	3	0.13
46	1	0.04
48	4	0.17
49	1	0.04
50	81	3.47
51	9	0.39
52	9	0.39
53	11	0.47
54	4	0.17
55	150	6.43
56	11	0.47
57	7	0.30
58	10	0.43
59	9	0.39
60	419	17.96
61	11	0.47
62	13	0.56
63	10	0.43
64	5	0.21
65	77	3.30
66	9	0.39
67	6	0.26
68	6	0.26
69	2	0.09
70	50	2.14
71	4	0.17
72	2	0.09
73	3	0.13

74	2	0.09
75	9	0.39
76	2	0.09
78	2	0.09
80	4	0.17
84	1	0.04
90	1	0.04
100	1	0.04
Total	2,333	100.00

- **fh001 : Num. Of Months Worked Last Year**

	No	%
0	34	2.42
1	49	3.49
2	48	3.42
3	53	3.77
4	37	2.63
5	33	2.35
6	90	6.41
7	23	1.64
8	54	3.84
9	28	1.99
10	110	7.83
11	78	5.55
12	768	54.66
Total	1,405	100.00

- **fh002 : Days Worked Per Week**

	No	%
0	31	2.24
1	19	1.37
2	41	2.96
3	77	5.55
4	64	4.61
5	108	7.79
6	85	6.13
7	950	68.49
8	2	0.14
10	1	0.07
15	1	0.07
20	4	0.29
30	4	0.29
Total	1,387	100.00

- **fh003 : Hours Worked Per Day**

	No	%
0	33	2.39
1	10	0.73
2	42	3.05
3	68	4.93
4	66	4.79
5	85	6.16
6	91	6.60
7	58	4.21
8	315	22.84
9	51	3.70
10	276	20.01
11	17	1.23
12	166	12.04
13	27	1.96
14	27	1.96
15	17	1.23
16	30	2.18
Total	1,379	100.00

- **fh004 : Days Missing for Health Problems**

Mean	Min	Max	OBS
8.2	0.0	350.0	1,376

- **fh005_gb : Self-Employed Occupation Code (GB)**

	No	%
10303	2	0.18
10402	1	0.09
10510	35	3.09
10520	6	0.53
10544	439	38.82
10545	50	4.42
10546	102	9.02
10547	4	0.35
10548	20	1.77
10549	21	1.86
10550	17	1.50
10551	2	0.18
10552	44	3.89
20229	1	0.09
20501	23	2.03
20502	1	0.09
20507	2	0.18
20603	1	0.09
20901	1	0.09

20905	1	0.09
21006	1	0.09
21007	3	0.27
30101	1	0.09
30102	2	0.18
30202	1	0.09
40101	2	0.18
40102	1	0.09
40104	1	0.09
40105	33	2.92
40301	7	0.62
40305	1	0.09
40402	1	0.09
40403	1	0.09
40701	2	0.18
40703	1	0.09
40704	10	0.88
40707	5	0.44
40711	16	1.41
40713	2	0.18
40714	2	0.18
40719	5	0.44
50101	16	1.41
50103	17	1.50
50105	1	0.09
50106	4	0.35
50204	2	0.18
50301	10	0.88
50302	4	0.35
50305	2	0.18
50309	1	0.09
50401	5	0.44
50402	4	0.35
50502	1	0.09
50901	1	0.09
60103	5	0.44
60104	2	0.18
60317	2	0.18
60402	2	0.18
60504	1	0.09
60512	5	0.44
60519	1	0.09
60601	3	0.27
60701	1	0.09
60706	3	0.27
60801	1	0.09
61002	1	0.09
61003	1	0.09
61004	2	0.18
61101	9	0.80
61102	11	0.97
61201	5	0.44

61205	11	0.97
61206	7	0.62
61207	2	0.18
61501	5	0.44
61503	16	1.41
61602	2	0.18
61607	1	0.09
62001	2	0.18
62003	1	0.09
62007	2	0.18
62009	7	0.62
62201	1	0.09
62202	38	3.36
62203	1	0.09
62205	1	0.09
62206	2	0.18
62207	15	1.33
62209	1	0.09
62301	10	0.88
62304	1	0.09
62305	1	0.09
62501	1	0.09
62901	1	0.09
62903	8	0.71
Total	1,131	100.00

- **fh005_isco : Self-Employed Occupation Code (ISCO)**

	No	%
1143	2	0.21
1210	36	3.70
1220	6	0.62
1314	439	45.12
1316	102	10.48
2149	1	0.10
2220	1	0.10
2221	23	2.36
2310	1	0.10
2332	1	0.10
2411	1	0.10
2452	4	0.41
3150	1	0.10
3220	2	0.21
3227	2	0.21
3417	1	0.10
3430	1	0.10
4110	2	0.21
5111	1	0.10
5121	1	0.10
5122	7	0.72
5123	1	0.10

5130	2	0.21
5132	8	0.82
5141	10	1.03
5149	5	0.51
5169	1	0.10
5220	3	0.31
6111	16	1.64
6113	18	1.85
6121	10	1.03
6122	4	0.41
6129	1	0.10
6141	2	0.21
6151	5	0.51
6152	4	0.41
7111	5	0.51
7121	1	0.10
7122	38	3.91
7123	1	0.10
7130	1	0.10
7134	2	0.21
7141	15	1.54
7210	2	0.21
7230	4	0.41
7240	4	0.41
7241	17	1.75
7300	7	0.72
7313	2	0.21
7330	3	0.31
7410	29	2.98
7430	1	0.10
7432	3	0.31
7433	20	2.06
8112	2	0.21
8141	5	0.51
8212	2	0.21
8229	2	0.21
8240	16	1.64
8281	5	0.51
8283	1	0.10
8290	1	0.10
8320	10	1.03
8331	1	0.10
8334	1	0.10
8340	1	0.10
9000	8	0.82
9161	33	3.39
9162	2	0.21
9312	2	0.21
9322	1	0.10
Total	973	100.00

- **fh006 : Location of Your Company or Workplace**

	No	%
1 This village/community	839	74.25
2 Other village/community in this county/city	243	21.50
3 Another county/city in this province	31	2.74
4 Another province	17	1.50
Total	1,130	100.00

- **fh006_1 : City**

	No	%
11	3	10.71
16	1	3.57
18	1	3.57
24	2	7.14
27	1	3.57
40	14	50.00
55	1	3.57
60	1	3.57
66	1	3.57
74	1	3.57
82	1	3.57
99	1	3.57
Total	28	100.00

- **fh006_2 : County**

	No	%
04	4	16.67
06	1	4.17
08	1	4.17
16	1	4.17
28	1	4.17
31	3	12.50
43	1	4.17
51	2	8.33
54	1	4.17
57	1	4.17
59	2	8.33
63	3	12.50
75	1	4.17
76	1	4.17
81	1	4.17
Total	24	100.00

- **fh006_3 : Province**

	No	%
01	1	5.88
03	2	11.76
05	1	5.88
09	1	5.88
10	1	5.88
11	1	5.88
13	1	5.88
15	2	11.76
18	2	11.76
20	1	5.88
29	2	11.76
32	1	5.88
33	1	5.88
Total	17	100.00

- **fh006_4 : City**

	No	%
01	1	7.14
17	1	7.14
24	3	21.43
40	5	35.71
60	1	7.14
74	1	7.14
76	1	7.14
99	1	7.14
Total	14	100.00

- **fh006_5 : County**

	No	%
04	1	9.09
28	1	9.09
38	1	9.09
46	1	9.09
58	1	9.09
63	6	54.55
Total	11	100.00

- **fh007_indc : Self-Employed Industry Code**

	No	%
1	61	5.39
11	3	0.27
12	10	0.88

13	3	0.27
14	1	0.09
15	69	6.10
16	2	0.18
17	28	2.48
18	18	1.59
19	4	0.35
2	7	0.62
3	145	12.82
4	7	0.62
5	84	7.43
6	115	10.17
7	4	0.35
8	512	45.27
9	58	5.13
Total	1,131	100.00

- **fh008_1 : Year**

Mean	Min	Max	OBS
1,998.5	1,900.0	2,011.0	1,060

- **fh008_2 : Month**

	No	%
0	505	47.64
1	88	8.30
2	49	4.62
3	65	6.13
4	46	4.34
5	50	4.72
6	45	4.25
7	49	4.62
8	45	4.25
9	31	2.92
10	40	3.77
11	21	1.98
12	26	2.45
Total	1,060	100.00

- **fh009 : Any Other Household Members Work in the Same Self-Employed Activity**

	No	%
1 Yes	370	32.80
2 No	758	67.20
Total	1,128	100.00

- **fh010 : Estimates of the Net Income Earned from this Activity Last Year**

Mean	Min	Max	OBS
18,741.9	10.0	1,000,000.0	749

- **fh011_a : Min Bracket**

No Observations

- **fh011_b : Max Bracket**

No Observations

- **fh013 : Location of this Company or Workplace**

	No	%
1 This village/community	198	82.50
2 Other village/community in this county/city	34	14.17
3 Another county/city in this province	5	2.08
4 Another province	3	1.25
Total	240	100.00

- **fh013_1 : City**

	No	%
01	2	40.00
40	3	60.00
Total	5	100.00

- **fh013_2 : County**

	No	%
31	2	40.00
39	2	40.00
63	1	20.00
Total	5	100.00

- **fh013_3 : Province**

	No	%
09	1	50.00
26	1	50.00
Total	2	100.00

- **fh013_4 : City**

	No	%
40	1	50.00
63	1	50.00
Total	2	100.00

- **fh013_5 : County**

	No	%
63	1	50.00
78	1	50.00
Total	2	100.00

- **fh014_indc : Family Business Industry Code**

	No	%
1	79	33.19
15	12	5.04
16	2	0.84
17	6	2.52
18	3	1.26
19	1	0.42
2	1	0.42
3	41	17.23
4	1	0.42
5	21	8.82
6	12	5.04
7	1	0.42
8	43	18.07
9	15	6.30
Total	238	100.00

- **fh015_gb : Family Business Occupation Code (GB)**

	No	%
10303	1	0.42

10510	1	0.42
10544	15	6.30
10545	1	0.42
10546	2	0.84
10548	1	0.42
20501	5	2.10
20904	1	0.42
20909	1	0.42
21007	1	0.42
30202	1	0.42
40101	27	11.34
40103	1	0.42
40105	2	0.84
40301	1	0.42
40305	11	4.62
40401	1	0.42
40501	1	0.42
40600	1	0.42
40703	1	0.42
40704	4	1.68
40706	1	0.42
40708	1	0.42
40713	3	1.26
40714	1	0.42
40719	1	0.42
50101	61	25.63
50103	2	0.84
50106	1	0.42
50201	1	0.42
50301	6	2.52
50302	2	0.84
50303	1	0.42
50306	1	0.42
50401	3	1.26
50402	4	1.68
50901	1	0.42
60103	1	0.42
60317	1	0.42
60401	2	0.84
60402	1	0.42
60405	1	0.42
60511	1	0.42
60512	2	0.84
60601	2	0.84
60702	1	0.42
60902	3	1.26
61003	1	0.42
61101	10	4.20
61201	2	0.84
61204	2	0.84
61205	3	1.26
61501	1	0.42

61502	1	0.42
61503	5	2.10
61602	1	0.42
62202	11	4.62
62207	4	1.68
62211	1	0.42
62301	7	2.94
62903	4	1.68
Total	238	100.00

- **fh015_isco : Family Business Occupation Code (ISCO)**

	No	%
1143	1	0.42
1210	1	0.42
1314	15	6.36
1316	2	0.85
2221	5	2.12
2331	1	0.42
2359	1	0.42
2452	1	0.42
3224	1	0.42
3416	1	0.42
5110	1	0.42
5121	1	0.42
5122	1	0.42
5123	11	4.66
5130	3	1.27
5132	3	1.27
5141	4	1.69
5149	1	0.42
5169	1	0.42
5220	27	11.44
6111	62	26.27
6113	2	0.85
6121	6	2.54
6122	2	0.85
6123	1	0.42
6141	1	0.42
6151	3	1.27
6152	4	1.69
7111	1	0.42
7122	11	4.66
7141	4	1.69
7210	3	1.27
7224	1	0.42
7230	3	1.27
7410	8	3.39
7430	1	0.42
7433	10	4.24
8141	2	0.85

8161	1	0.42
8212	1	0.42
8229	1	0.42
8232	3	1.27
8240	5	2.12
8281	2	0.85
8282	1	0.42
8320	7	2.97
8331	1	0.42
9000	4	1.69
9161	2	0.85
9162	1	0.42
Total	236	100.00

- **fh016 : Num. of Family Members, Relatives or Friends without Payment**

	No	%
0	75	32.75
1	77	33.62
2	57	24.89
3	9	3.93
4	6	2.62
5	3	1.31
6	2	0.87
Total	229	100.00

- **fh017_1 : Year**

Mean	Min	Max	OBS
1,997.6	1,900.0	2,010.0	159

- **fh017_2 : Month**

	No	%
0	58	36.25
1	25	15.63
2	9	5.63
3	9	5.63
4	4	2.50
5	12	7.50
6	4	2.50
7	8	5.00
8	5	3.13
9	6	3.75
10	13	8.13
11	3	1.88
12	4	2.50

Total	160	100.00

- **fh018_1 : Year**

Mean	Min	Max	OBS
1,999.5	1,900.0	2,010.0	166

- **fh018_2 : Month**

	No	%
0	49	29.34
1	23	13.77
2	11	6.59
3	12	7.19
4	6	3.59
5	10	5.99
6	5	2.99
7	6	3.59
8	8	4.79
9	8	4.79
10	15	8.98
11	3	1.80
12	11	6.59
Total	167	100.00

- **fh019 : Any other Household Member in the Same Company**

	No	%
1 Yes	76	35.35
2 No	139	64.65
Total	215	100.00

- **fh020 : Estimates of the Net Income Earned from this Activity Last Year**

Mean	Min	Max	OBS
372,746.5	0.0	50,000,000.0	142

- **fi001s1 : Covered by Insurance**

	No	%
1 Pension	105	100.00
Total	105	100.00

- **fi001s2 : Covered by Insurance**

	No	%
2 Health insurance	62	100.00
Total	62	100.00

- **fi001s3 : Covered by Insurance**

	No	%
3 Unemployment insurance	2	100.00
Total	2	100.00

- **fi001s4 : Covered by Insurance**

	No	%
4 Workers injury insurance	10	100.00
Total	10	100.00

- **fi001s5 : Covered by Insurance**

	No	%
5 Maternity Insurance	1	100.00
Total	1	100.00

- **fi001s6 : Covered by Insurance**

	No	%
6 None	1,114	100.00
Total	1,114	100.00

- **fi002 : The Reason for Not Covered by Insurance**

	No	%
1 It is not possible for self-employed to participate in social insurance at my local area	571	54.59
2 Social insurance is available to self employed but I do not participate	411	39.29
3 I have social insurance from elsewhere	25	2.39
4 I have passed retirement age	39	3.73
Total	1,046	100.00

- **fi003 : From Where Do You Have Insurance**

	No	%
1 I am covered through another work unit	28	75.68
2 I contribute through job service center	9	24.32
Total	37	100.00

- **fi004 : Why You Contribute through Another Work Unit**

	No	%
1 I was laid-off by this work unit but employment contract is not terminated	9	36.00
5 Other	16	64.00
Total	25	100.00

- **fi005 : Your Own Contribution**

Mean	Min	Max	OBS
2,692.7	0.0	50,150.0	184

- **fi006 : Is the Income the Net after Paying for the above Benefits**

	No	%
1 Yes	37	56.06
2 No	29	43.94
Total	66	100.00

- **fi007 : Income Base**

Mean	Min	Max	OBS
964.9	0.0	10,000.0	97

- **fi008 : Do You Know How Much You or Your Work Unit Contributes**

	No	%
1 I know the amount by myself and the work unit	3	17.65
2 I know the amount by myself but not the work unit	9	52.94
3 I do not know either my contribution or the work units contribution at all	5	29.41
Total	17	100.00

- **fi009 : Work Unit Contribution**

	No	%

0	3	75.00
7	1	25.00
Total	4	100.00

- **fi010 : Your Own Contribution**

Mean	Min	Max	OBS
194.1	0.0	500.0	10

- **fi011 : Expect to Process Retirement at this Business/Organization**

	No	%
1 Yes	23	13.77
2 No	144	86.23
Total	167	100.00

- **fi012 : Age You Plan to Stop Working**

	No	%
0	816	66.83
2	1	0.08
20	1	0.08
38	1	0.08
45	1	0.08
48	3	0.25
49	1	0.08
50	25	2.05
51	1	0.08
52	2	0.16
54	5	0.41
55	39	3.19
56	8	0.66
57	5	0.41
58	5	0.41
60	150	12.29
61	4	0.33
62	6	0.49
63	2	0.16
64	5	0.41
65	44	3.60
66	4	0.33
67	4	0.33
68	4	0.33
69	1	0.08
70	54	4.42
71	2	0.16

72	2	0.16
75	11	0.90
76	1	0.08
78	1	0.08
79	1	0.08
80	9	0.74
82	1	0.08
87	1	0.08
Total	1,221	100.00

- **fj001 : Num. of Jobs Excluding Main Job**

	No	%
1	131	99.24
2	1	0.76
Total	132	100.00

- **fj002 : Hours Per Week Worked Excluding Main Job**

Mean	Min	Max	OBS
11.9	0.0	105.0	144

- **fj003 : Average Monthly Income from Side Job**

Mean	Min	Max	OBS
1,959.2	0.0	180,000.0	140

- **fj004_a : Min Bracket**

Mean	Min	Max	OBS
260.1	0.0	5,001.0	25

- **fj004_b : Max Bracket**

Mean	Min	Max	OBS
76,000,536.8	499.0	99,999,996.0	25

- **fk001_1 : Year**

Mean	Min	Max	OBS
2,000.7	1,900.0	2,021.0	5,001

- **fk001_2 : Month**

	No	%
0	2,046	40.91
1	587	11.74
2	190	3.80
3	264	5.28
4	168	3.36
5	234	4.68
6	230	4.60
7	284	5.68
8	212	4.24
9	184	3.68
10	212	4.24
11	121	2.42
12	269	5.38
Total	5,001	100.00

- **fk002 : Search for a New Job**

	No	%
1 Yes	64	1.22
2 No	5,194	98.78
Total	5,258	100.00

- **fk003 : Age You Plan to Stop Working**

	No	%
0	33	57.89
50	4	7.02
55	3	5.26
56	1	1.75
60	13	22.81
65	2	3.51
72	1	1.75
Total	57	100.00

- **fl001 : Type of Work**

	No	%
1 Employed	2,099	39.84
2 Self employed	234	4.44
3 Unpaid family business	179	3.40
4 Farming	2,756	52.32
Total	5,268	100.00

- **fl002_1 : Year**

Mean	Min	Max	OBS
1,970.2	1,900.0	2,011.0	4,523

- **fl002_2 : Month**

	No	%
0	2,303	50.92
1	400	8.84
2	147	3.25
3	263	5.81
4	165	3.65
5	179	3.96
6	141	3.12
7	182	4.02
8	151	3.34
9	167	3.69
10	186	4.11
11	78	1.72
12	161	3.56
Total	4,523	100.00

- **fl003_1 : Year**

Mean	Min	Max	OBS
2,001.2	1,900.0	2,026.0	4,767

- **fl003_2 : Month**

	No	%
0	1,953	40.97
1	541	11.35
2	173	3.63
3	225	4.72
4	154	3.23
5	211	4.43
6	240	5.03
7	288	6.04
8	233	4.89
9	178	3.73
10	198	4.15
11	105	2.20
12	268	5.62
Total	4,767	100.00

- **fl004 : Location of Job**

	No	%
1 This village/community	3,009	60.96
2 Other village/community in this county/city	1,598	32.37
3 Another county/city in this province	193	3.91
4 Another province	136	2.76
Total	4,936	100.00

- **fl004_1 : City**

	No	%
01	14	5.02
02	1	0.36
04	10	3.58
11	13	4.66
16	7	2.51
17	2	0.72
24	22	7.89
27	7	2.51
28	2	0.72
40	94	33.69
46	28	10.04
49	1	0.36
52	1	0.36
53	13	4.66
55	12	4.30
60	7	2.51
63	6	2.15
74	16	5.73
82	14	5.02
84	1	0.36
86	2	0.72
96	2	0.72
99	4	1.43
Total	279	100.00

- **fl004_2 : County**

	No	%
02	13	5.04
03	2	0.78
04	11	4.26
06	5	1.94
08	1	0.39
13	1	0.39
16	4	1.55
28	16	6.20

31	17	6.59
33	2	0.78
37	12	4.65
38	1	0.39
39	4	1.55
43	9	3.49
44	6	2.33
46	6	2.33
49	1	0.39
54	6	2.33
56	2	0.78
57	4	1.55
59	10	3.88
63	95	36.82
73	1	0.39
75	2	0.78
76	6	2.33
78	5	1.94
81	5	1.94
90	1	0.39
91	4	1.55
92	5	1.94
99	1	0.39
Total	258	100.00

- **fl004_3 : Province**

	No	%
01	1	0.40
02	1	0.40
03	10	4.02
04	3	1.20
05	8	3.21
06	7	2.81
07	6	2.41
08	6	2.41
09	28	11.24
10	13	5.22
11	14	5.62
12	4	1.61
13	2	0.80
14	11	4.42
15	39	15.66
16	7	2.81
17	8	3.21
18	3	1.20
19	1	0.40
20	17	6.83
21	6	2.41
23	3	1.20
24	3	1.20

25	6	2.41
26	3	1.20
27	4	1.61
28	4	1.61
29	11	4.42
32	4	1.61
33	10	4.02
34	6	2.41
Total	249	100.00

- **fl004_4 : City**

	No	%
01	9	3.78
04	10	4.20
05	2	0.84
11	25	10.50
16	2	0.84
17	2	0.84
18	1	0.42
24	23	9.66
27	3	1.26
35	1	0.42
40	82	34.45
46	6	2.52
49	5	2.10
51	2	0.84
53	18	7.56
55	10	4.20
60	5	2.10
66	3	1.26
74	9	3.78
82	11	4.62
84	6	2.52
96	2	0.84
99	1	0.42
Total	238	100.00

- **fl004_5 : County**

	No	%
02	6	2.93
03	1	0.49
04	5	2.44
06	4	1.95
08	3	1.46
28	3	1.46
31	15	7.32
39	6	2.93

44	5	2.44
45	1	0.49
46	5	2.44
51	2	0.98
54	1	0.49
56	1	0.49
57	2	0.98
59	7	3.41
63	125	60.98
75	2	0.98
76	3	1.46
78	7	3.41
91	1	0.49
Total	205	100.00

- **fl006_indc : Most Recent Job Industry Code**

	No	%
1	126	5.23
10	27	1.12
11	10	0.42
12	14	0.58
13	40	1.66
14	11	0.46
15	36	1.50
16	168	6.98
17	74	3.07
18	16	0.66
19	239	9.93
2	55	2.28
3	869	36.09
4	39	1.62
5	190	7.89
6	143	5.94
7	8	0.33
8	271	11.25
9	72	2.99
Total	2,408	100.00

- **fl007 : Work Unit Still in Existence**

	No	%
1 Yes	1,354	57.94
2 No	983	42.06
Total	2,337	100.00

- **fl008 : Hours Per Week**

Mean	Min	Max	OBS
45.3	0.0	168.0	2,322

- **fl009 : Monthly Income**

Mean	Min	Max	OBS
1,747.8	-9,999.0	999,997.0	1,913

- **fl010_a : Min Bracket**

Mean	Min	Max	OBS
392.5	0.0	10,000.0	65

- **fl010_b : MAx Bracket**

Mean	Min	Max	OBS
18,462,544.7	499.0	99,999,996.0	65

- **fl011 : Value of other Bonuses**

Mean	Min	Max	OBS
2,095.3	0.0	150,000.0	1,914

- **fl012 : Do You Have Employees**

	No	%
1 Self-employed with employees	29	13.30
2 Self-employed without employees	135	61.93
3 Family business worker without pay	54	24.77
Total	218	100.00

- **fl013 : Type of Worker**

	No	%
1 Regular wage worker	1,446	73.66
2 Contract worker	109	5.55
3 Temporary wage worker	379	19.31
4 Casual wage worker	29	1.48
Total	1,963	100.00

- **fl014 : Type of Work Unit**

	No	%
1 Government	206	10.45
2 Institutions	437	22.16
3 NGO	16	0.81
4 Firm	1,076	54.56
5 Individual firm	160	8.11
6 Individual farmer	15	0.76
7 Individual household	16	0.81
8 Other	46	2.33
Total	1,972	100.00

- **fl015 : Were You a Civil Servant**

	No	%
1 Yes	100	47.17
2 No	112	52.83
Total	212	100.00

- **fl016 : Ownership Type of the Business**

	No	%
1 100% State owned firm	724	67.47
2 State-controlled firm	18	1.68
3 100% Collective-owned firm	233	21.71
4 Collective-controlled firm	14	1.30
5 100% Private firm	55	5.13
6 Private-controlled firm	15	1.40
7 100% foreign-owned	3	0.28
8 Joint venture	2	0.19
9 Other joint-ownership	4	0.37
10 Other	5	0.47
Total	1,073	100.00

- **fl017_gb : Most Recent Job Occupation Code (GB)**

	No	%
10100	1	0.04
10201	2	0.08
10202	2	0.08
10205	26	1.08
10209	1	0.04
10302	3	0.12
10303	13	0.54
10401	4	0.17

10402	5	0.21
10403	1	0.04
10409	11	0.46
10510	15	0.62
10520	58	2.41
10530	24	1.00
10544	2	0.08
10545	1	0.04
10546	3	0.12
20104	1	0.04
20105	1	0.04
20108	1	0.04
20113	1	0.04
20116	1	0.04
20117	1	0.04
20118	1	0.04
20201	2	0.08
20203	1	0.04
20204	1	0.04
20206	2	0.08
20207	4	0.17
20208	1	0.04
20211	1	0.04
20222	2	0.08
20223	5	0.21
20225	1	0.04
20226	6	0.25
20234	1	0.04
20236	1	0.04
20239	1	0.04
20303	1	0.04
20305	1	0.04
20501	41	1.70
20502	1	0.04
20505	1	0.04
20506	2	0.08
20507	2	0.08
20508	14	0.58
20602	6	0.25
20603	103	4.28
20701	8	0.33
20702	2	0.08
20801	2	0.08
20802	3	0.12
20803	1	0.04
20804	1	0.04
20901	8	0.33
20902	5	0.21
20903	40	1.66
20904	68	2.82
20905	10	0.42
20909	17	0.71

21003	2	0.08
21005	1	0.04
21007	1	0.04
21201	2	0.08
21202	2	0.08
21204	1	0.04
21205	2	0.08
21206	2	0.08
30101	65	2.70
30102	121	5.02
30109	1	0.04
30201	21	0.87
30202	24	1.00
30209	3	0.12
30301	2	0.08
30302	2	0.08
30303	6	0.25
30309	1	0.04
30900	5	0.21
40101	185	7.68
40102	23	0.96
40103	15	0.62
40105	9	0.37
40107	1	0.04
40109	2	0.08
40201	42	1.74
40202	6	0.25
40301	42	1.74
40305	42	1.74
40401	13	0.54
40403	3	0.12
40501	9	0.37
40502	9	0.37
40503	1	0.04
40504	5	0.21
40600	5	0.21
40701	10	0.42
40702	1	0.04
40703	11	0.46
40704	2	0.08
40705	1	0.04
40707	4	0.17
40708	4	0.17
40711	1	0.04
40712	1	0.04
40713	18	0.75
40714	39	1.62
40719	3	0.12
50101	90	3.74
50103	7	0.29
50106	6	0.25
50201	4	0.17

50204	2	0.08
50301	2	0.08
50302	2	0.08
50303	1	0.04
50305	1	0.04
50401	6	0.25
50402	1	0.04
50502	1	0.04
50901	3	0.12
60101	4	0.17
60103	25	1.04
60104	4	0.17
60105	1	0.04
60106	3	0.12
60201	2	0.08
60202	28	1.16
60203	3	0.12
60204	5	0.21
60205	2	0.08
60208	4	0.17
60301	8	0.33
60302	2	0.08
60304	4	0.17
60305	4	0.17
60309	5	0.21
60312	2	0.08
60317	5	0.21
60401	35	1.45
60402	24	1.00
60404	2	0.08
60405	2	0.08
60502	6	0.25
60503	2	0.08
60504	3	0.12
60506	2	0.08
60507	8	0.33
60508	1	0.04
60511	4	0.17
60512	39	1.62
60514	1	0.04
60519	4	0.17
60521	2	0.08
60601	40	1.66
60602	2	0.08
60702	3	0.12
60704	2	0.08
60705	3	0.12
60706	23	0.96
60801	1	0.04
60802	6	0.25
60803	1	0.04
60804	1	0.04

60805	1	0.04
60901	6	0.25
60902	6	0.25
61001	4	0.17
61002	30	1.25
61003	23	0.96
61004	5	0.21
61005	1	0.04
61101	53	2.20
61102	18	0.75
61103	6	0.25
61201	9	0.37
61202	2	0.08
61203	20	0.83
61204	3	0.12
61205	8	0.33
61206	2	0.08
61207	5	0.21
61302	4	0.17
61401	8	0.33
61502	4	0.17
61503	27	1.12
61504	4	0.17
61505	7	0.29
61506	5	0.21
61601	7	0.29
61602	9	0.37
61605	1	0.04
61606	6	0.25
61607	1	0.04
61701	4	0.17
61704	16	0.66
61803	2	0.08
61901	2	0.08
61902	13	0.54
61903	4	0.17
62005	2	0.08
62006	1	0.04
62007	1	0.04
62009	4	0.17
62101	1	0.04
62201	2	0.08
62202	66	2.74
62203	4	0.17
62204	4	0.17
62206	1	0.04
62207	9	0.37
62209	23	0.96
62211	3	0.12
62301	50	2.08
62302	8	0.33
62304	3	0.12

62305	6	0.25
62309	3	0.12
62501	32	1.33
62504	6	0.25
62901	25	1.04
62902	2	0.08
62903	56	2.33
Total	2,408	100.00

- **fl017_isco : Most Recent Job Occupation Code (ISCO)**

	No	%
1100	3	0.12
1140	2	0.08
1142	3	0.12
1143	13	0.54
1200	27	1.12
1210	36	1.50
1220	58	2.41
1230	24	1.00
1314	2	0.08
1316	3	0.12
2113	1	0.04
2122	6	0.25
2141	7	0.29
2145	12	0.50
2146	2	0.08
2147	4	0.17
2149	3	0.12
2211	2	0.08
2213	3	0.12
2220	1	0.04
2221	42	1.74
2223	1	0.04
2224	2	0.08
2230	14	0.58
2310	8	0.33
2320	45	1.87
2331	68	2.83
2332	10	0.42
2359	17	0.71
2411	103	4.28
2421	4	0.17
2422	2	0.08
2429	1	0.04
2431	2	0.08
2442	2	0.08
2443	1	0.04
2444	2	0.08
2451	4	0.17
2452	1	0.04

2455	3	0.12
3110	6	0.25
3117	4	0.17
3131	1	0.04
3132	2	0.08
3150	32	1.33
3220	2	0.08
3227	1	0.04
3412	2	0.08
3416	15	0.62
3419	8	0.33
3430	65	2.70
3439	1	0.04
3472	1	0.04
4110	122	5.07
4131	42	1.74
4133	6	0.25
4142	2	0.08
4190	5	0.21
4210	9	0.37
5110	24	1.00
5121	12	0.50
5122	42	1.74
5123	42	1.74
5130	18	0.75
5132	34	1.41
5141	2	0.08
5149	8	0.33
5162	21	0.87
5169	27	1.12
5200	2	0.08
5220	208	8.64
6111	90	3.74
6113	7	0.29
6121	2	0.08
6122	2	0.08
6123	1	0.04
6141	6	0.25
6151	6	0.25
6152	1	0.04
7111	29	1.20
7121	2	0.08
7122	66	2.74
7123	4	0.17
7129	4	0.17
7134	1	0.04
7141	9	0.37
7210	59	2.45
7213	2	0.08
7224	2	0.08
7230	49	2.04
7240	32	1.33

7241	4	0.17
7243	3	0.12
7245	3	0.12
7300	4	0.17
7313	1	0.04
7321	4	0.17
7324	16	0.66
7330	1	0.04
7340	19	0.79
7400	1	0.04
7410	33	1.37
7412	2	0.08
7413	20	0.83
7416	4	0.17
7430	23	0.96
7431	4	0.17
7432	36	1.50
7433	71	2.95
7434	6	0.25
7436	2	0.08
8112	4	0.17
8121	40	1.66
8122	4	0.17
8141	4	0.17
8142	4	0.17
8143	7	0.29
8150	21	0.87
8155	2	0.08
8160	2	0.08
8161	3	0.12
8212	16	0.66
8220	2	0.08
8221	8	0.33
8229	5	0.21
8231	6	0.25
8232	6	0.25
8240	27	1.12
8253	5	0.21
8280	2	0.08
8281	53	2.20
8282	6	0.25
8283	5	0.21
8290	8	0.33
8300	3	0.12
8310	8	0.33
8320	50	2.08
8331	3	0.12
8334	6	0.25
8340	3	0.12
9000	56	2.33
9161	9	0.37
9162	39	1.62

9312	24	1.00
9322	25	1.04
Total	2,407	100.00

- **fl018 : Is this the Business/Organization Where You Processed Retirement**

	No	%
1 Yes	1,364	87.89
2 No	188	12.11
Total	1,552	100.00

- **fl019 : Expect to Process Retirement from this Business/Organization**

	No	%
1 Yes	138	4.07
2 No	3,251	95.93
Total	3,389	100.00

- **fl020s1 : Reason for Leaving**

	No	%
1 Business closed	212	100.00
Total	212	100.00

- **fl020s2 : Reason for Leaving**

	No	%
2 Quit	79	100.00
Total	79	100.00

- **fl020s3 : Reason for Leaving**

	No	%
3 I was laid-off	59	100.00
Total	59	100.00

- **fl020s4 : Reason for Leaving**

	No	%
4 I was fired	45	100.00

Total	45	100.00

- **fl020s5 : Reason for Leaving**

No Observations

- **fl020s6 : Reason for Leaving**

No Observations

- **fl020s7 : Reason for Leaving**

	No	%
7 I stopped working for health reasons	1,471	100.00
Total	1,471	100.00

- **fl020s8 : Reason for Leaving**

	No	%
8 I stopped working for family reasons	396	100.00
Total	396	100.00

- **fl020s9 : Reason for Leaving**

	No	%
9 I was transferred to another job	25	100.00
Total	25	100.00

- **fl020s10 : Reason for Leaving**

	No	%
10 I was sent down to do manual labor	3	100.00
Total	3	100.00

- **fl020s11 : Reason for Leaving**

	No	%

11 I started working off-farm locally	19	100.00
Total	19	100.00

- **fl020s12 : Reason for Leaving**

	No	%
12 I went to work away from home	4	100.00
Total	4	100.00

- **fl020s13 : Reason for Leaving**

	No	%
13 Better job in local area	11	100.00
Total	11	100.00

- **fl020s14 : Reason for Leaving**

	No	%
14 Better job in another location	4	100.00
Total	4	100.00

- **fl020s15 : Reason for Leaving**

	No	%
15 I retired	176	100.00
Total	176	100.00

- **fl020s16 : Reason for Leaving**

	No	%
16 Other	1,199	100.00
Total	1,199	100.00

- **fl021 : Recived Compensation**

	No	%
1 Yes	172	3.48
2 No	4,766	96.52
Total	4,938	100.00

- **fl022_1 : Yuan**

Mean	Min	Max	OBS
13,511.5	0.0	125,000.0	168

- **fl022_2 : Years**

	No	%
0	17	10.12
1	27	16.07
2	3	1.79
3	2	1.19
5	1	0.60
6	1	0.60
8	1	0.60
10	3	1.79
11	1	0.60
12	4	2.38
13	3	1.79
14	1	0.60
15	8	4.76
16	3	1.79
17	3	1.79
18	2	1.19
19	1	0.60
20	14	8.33
21	4	2.38
22	5	2.98
23	3	1.79
24	2	1.19
25	3	1.79
26	4	2.38
27	3	1.79
28	4	2.38
29	1	0.60
30	9	5.36
31	6	3.57
32	3	1.79
33	4	2.38
34	2	1.19
35	6	3.57
37	4	2.38
38	5	2.98
41	1	0.60
43	2	1.19
44	2	1.19
Total	168	100.00

- **fl022_a : Min bracket**

Mean	Min	Max	OBS
457.7	0.0	20,001.0	839

- **fl022_b : Max Bracket**

Mean	Min	Max	OBS
10,370,786.6	999.0	99,999,996.0	839

- **fm001 : Work Unit Processed Your Retirement**

	No	%
1 Yes	1,788	80.22
2 No	441	19.78
Total	2,229	100.00

- **fm003 : Type of Work Unit**

	No	%
1 Government	80	18.39
2 Institutions	77	17.70
4 Firm	233	53.56
5 Ran own business	11	2.53
6 Other	34	7.82
Total	435	100.00

- **fm004 : Location of Work Unit**

	No	%
1 This village/community	130	30.09
2 Other village/community in this county/city	268	62.04
3 Another county/city in this province	21	4.86
4 Another province	13	3.01
Total	432	100.00

- **fm004_1 : City**

	No	%
04	1	4.76
11	3	14.29
27	1	4.76
40	6	28.57

46	2	9.52
53	1	4.76
55	1	4.76
63	1	4.76
74	1	4.76
82	1	4.76
83	1	4.76
86	2	9.52
Total	21	100.00

- **fm004_2 : County**

	No	%
02	1	5.26
06	1	5.26
28	4	21.05
31	2	10.53
37	1	5.26
46	2	10.53
59	1	5.26
63	4	21.05
75	1	5.26
76	1	5.26
78	1	5.26
Total	19	100.00

- **fm004_3 : Province**

	No	%
09	1	7.69
10	1	7.69
11	2	15.38
12	1	7.69
15	1	7.69
17	2	15.38
18	1	7.69
25	1	7.69
27	1	7.69
34	2	15.38
Total	13	100.00

- **fm004_4 : City**

	No	%
04	1	8.33
11	2	16.67
24	1	8.33

40	7	58.33
53	1	8.33
Total	12	100.00

- **fm004_5 : County**

	No	%
08	1	10.00
16	2	20.00
38	1	10.00
44	1	10.00
63	4	40.00
92	1	10.00
Total	10	100.00

- **fm005_1 : Year**

Mean	Min	Max	OBS
1,998.8	1,960.0	2,010.0	115

- **fm005_2 : Month**

	No	%
0	35	30.43
1	6	5.22
2	1	0.87
3	10	8.70
4	1	0.87
5	8	6.96
6	7	6.09
7	8	6.96
8	8	6.96
9	8	6.96
10	12	10.43
11	1	0.87
12	10	8.70
Total	115	100.00

- **fm006 : Reason You Receded from Your Position**

	No	%
1 Due to poor health	11	9.02
4 I receded from my position voluntary	30	24.59
5 Reach retirement age, but not eligible working age	5	4.10
6 Other	76	62.30

Total	122	100.00

- **fm007 : Salary Per Month**

Mean	Min	Max	OBS
862.3	80.0	3,000.0	13

- **fm008 : Received Any Payments for Leaving Job**

	No	%
1 Yes	58	47.93
2 No	63	52.07
Total	121	100.00

- **fm009 : Value of Compensation**

Mean	Min	Max	OBS
11,653.9	0.0	120,000.0	57

- **fm010 : Currently Receiving Pension**

	No	%
1 Yes	21	16.94
2 No	103	83.06
Total	124	100.00

- **fm011 : Type of Retirement**

	No	%
1 Normal retirement	1,630	77.62
2 Early retirement	303	14.43
3 Internal retirement first then regular retirement	126	6.00
4 Internal retirement and not yet regular retirement	41	1.95
Total	2,100	100.00

- **fm012 : Retired as Worker or Cadre**

	No	%
1 Worker	1,482	70.84
2 Cadre	610	29.16
Total	2,092	100.00

- **fm013 : Ordinary Retiree or Revolutionary Retiree**

	No	%
1 Ordinary retiree	161	91.48
2 Revolutionary retiree	15	8.52
Total	176	100.00

- **fm014_1 : Year**

Mean	Min	Max	OBS
1,999.3	1,958.0	2,019.0	1,906

- **fm014_2 : Month**

	No	%
0	390	20.48
1	155	8.14
2	70	3.68
3	119	6.25
4	85	4.46
5	124	6.51
6	104	5.46
7	161	8.46
8	133	6.99
9	120	6.30
10	137	7.20
11	78	4.10
12	228	11.97
Total	1,904	100.00

- **fm015 : Reason for Early Retirement**

	No	%
1 I have 30 years of job experience	26	8.61
2 My work unit belonged to the category of high-risk and hard manual labor	20	6.62
3 My work unit was restructuringbankrupt	55	18.21
4 Due to poor health	124	41.06
5 Due to family reason	21	6.95
6 Other	56	18.54
Total	302	100.00

- **fm016 : Pre-Retirement Salary**

Mean	Min	Max	OBS
720.7	-9,999.0	30,000.0	1,874

- **fm017_a : Min Bracket**

Mean	Min	Max	OBS
564.9	0.0	5,001.0	54

- **fm017_b : Max Bracket**

Mean	Min	Max	OBS
14,815,896.9	499.0	99,999,996.0	54

- **fm018_1 : Year**

Mean	Min	Max	OBS
1,999.9	1,962.0	2,020.0	1,914

- **fm018_2 : Month**

	No	%
0	352	18.40
1	264	13.80
2	91	4.76
3	95	4.97
4	98	5.12
5	115	6.01
6	94	4.91
7	145	7.58
8	138	7.21
9	132	6.90
10	140	7.32
11	93	4.86
12	156	8.15
Total	1,913	100.00

- **fm019s1 : Receiving Pension Benefits from**

	No	%
1 Government	358	100.00
Total	358	100.00

- **fm019s2 : Receiving Pension Benefits from**

	No	%
2 Work unit	741	100.00
Total	741	100.00

- **fm019s3 : Receiving Pension Benefits from**

	No	%
3 Social insurance agency	797	100.00
Total	797	100.00

- **fm019s4 : Receiving Pension Benefits from**

	No	%
4 Commerial insurance company	15	100.00
Total	15	100.00

- **fm019s5 : Receiving Pension Benefits from**

	No	%
5 Other	81	100.00
Total	81	100.00

- **fm020 : Benefis when You Retired**

Mean	Min	Max	OBS
821.7	-9,999.0	9,000.0	1,890

- **fm021_a : Min Brackets**

Mean	Min	Max	OBS
541.9	0.0	5,000.0	48

- **fm021_b : Max Brackets**

Mean	Min	Max	OBS
18,750,915.4	499.0	99,999,996.0	48

- **fm022 : Monthly Pension**

Mean	Min	Max	OBS
1,661.8	0.0	8,000.0	1,919

- **fm023_a : Min Brackets**

Mean	Min	Max	OBS
413.2	0.0	3,500.0	23

- **fm023_b : Max Brackets**

Mean	Min	Max	OBS
43,479,041.4	499.0	99,999,996.0	23

- **fm024 : Eligible Work Years at the Time of Retirement**

Mean	Min	Max	OBS
31.4	0.0	95.0	1,853

- **fm025_1 : Year**

Mean	Min	Max	OBS
2,001.2	1,982.0	2,011.0	162

- **fm025_2 : Month**

	No	%
0	52	32.10
1	12	7.41
2	5	3.09
3	13	8.02
4	11	6.79
5	5	3.09
6	10	6.17
7	8	4.94
8	11	6.79
9	9	5.56
10	8	4.94
11	6	3.70
12	12	7.41
Total	162	100.00

- **fm026 : Reason for Internal Retirement**

	No	%
1 I have 30 years of job experience	19	11.45
2 My work unit was restructuringbankrupt	50	30.12
3 Due to poor health	27	16.27
4 Due to family reason	5	3.01
5 Other	65	39.16
Total	166	100.00

- **fm027 : Pre-Internal Retirement Salary**

Mean	Min	Max	OBS
909.2	0.0	4,000.0	162

- **fm028 : Internal Retirement Wage**

	No	%
.D	4	2.40
.E	163	97.60
Total	167	100.00

- **fm029_a : Min Bracket**

	No	%
0	5	100.00
Total	5	100.00

- **fm029_b : Max Bracket**

Mean	Min	Max	OBS
60,000,297.2	499.0	99,999,996.0	5

- **fm030_1 : Year**

Mean	Min	Max	OBS
2,004.1	1,945.0	2,015.0	121

- **fm030_2 : Month**

	No	%

0	36	29.75
1	9	7.44
2	4	3.31
3	5	4.13
4	8	6.61
5	9	7.44
6	3	2.48
7	11	9.09
8	3	2.48
9	3	2.48
10	12	9.92
11	6	4.96
12	12	9.92
Total	121	100.00

- **fm031 : Benifis when Formally Retired**

No Observations

- **fm032_a : Min Bracket**

Mean	Min	Max	OBS
625.6	0.0	2,001.0	8

- **fm032_b : Max Bracket**

Mean	Min	Max	OBS
37,501,185.4	999.0	99,999,996.0	8

- **fm033 : You Receive Pension from**

	No	%
6 Government	13	10.40
7 Work unit	51	40.80
8 Social insurance agency	54	43.20
10 Other	7	5.60
Total	125	100.00

- **fm034 : Monthly Pension**

Mean	Min	Max	OBS

1,638.8	0.0	4,400.0	125

- **fm035_a : Min Bracket**

	No	%
0	2	100.00
Total	2	100.00

- **fm035_b : Max Bracket**

Mean	Min	Max	OBS
99,999,996.0	99,999,996.0	99,999,996.0	2

- **fm036 : Eligible Work Years for Formal Retirement**

	No	%
0	1	0.83
5	1	0.83
10	2	1.65
15	1	0.83
19	1	0.83
21	4	3.31
23	2	1.65
24	1	0.83
25	2	1.65
26	1	0.83
27	4	3.31
28	1	0.83
29	4	3.31
30	12	9.92
31	8	6.61
32	9	7.44
33	6	4.96
34	5	4.13
35	8	6.61
36	2	1.65
37	11	9.09
38	4	3.31
39	4	3.31
40	6	4.96
41	4	3.31
42	7	5.79
43	5	4.13
44	2	1.65
45	1	0.83
46	1	0.83
52	1	0.83

Total	121	100.00

- **fm037_1 : Year**

Mean	Min	Max	OBS
2,012.0	1,997.0	2,019.0	34

- **fm037_2 : Month**

	No	%
0	7	20.59
1	3	8.82
2	6	17.65
3	1	2.94
4	1	2.94
5	1	2.94
6	4	11.76
8	1	2.94
9	4	11.76
10	1	2.94
11	1	2.94
12	4	11.76
Total	34	100.00

- **fm038 : Amount or Pencent**

	No	%
1 Yuan per month	26	86.67
2 % of salary before retirement	4	13.33
Total	30	100.00

- **fm038_1 : Amount Per Month**

Mean	Min	Max	OBS
1,671.2	0.0	5,000.0	26

- **fm038_2 : Percentage**

	No	%
90	3	75.00
100	1	25.00
Total	4	100.00

- **fm039_a : Min Bracket**

Mean	Min	Max	OBS
1,318.5	0.0	3,500.0	11

- **fm039_b : Max Bracket**

Mean	Min	Max	OBS
9,092,635.6	499.0	99,999,996.0	11

- **fm040 : Eligible Work Years**

	No	%
0	1	2.63
10	1	2.63
15	1	2.63
20	2	5.26
26	1	2.63
29	2	5.26
30	3	7.89
31	1	2.63
33	2	5.26
34	3	7.89
35	1	2.63
36	2	5.26
37	1	2.63
40	2	5.26
41	2	5.26
42	3	7.89
43	5	13.16
44	4	10.53
46	1	2.63
Total	38	100.00

- **fm041 : Eligible Work Years Currently Have**

	No	%
8	1	2.63
10	1	2.63
14	1	2.63
20	2	5.26
22	1	2.63
24	1	2.63
26	1	2.63
29	4	10.53
30	1	2.63

31	1	2.63
32	2	5.26
34	2	5.26
35	3	7.89
36	3	7.89
37	2	5.26
38	3	7.89
39	1	2.63
40	2	5.26
41	1	2.63
42	3	7.89
46	2	5.26
Total	38	100.00

- **fm042 : Have a Spouse When Processed Retirement**

	No	%
1 Yes	1,863	92.46
2 No	152	7.54
Total	2,015	100.00

- **fm043 : Health When Processed Retirement**

	No	%
1 Excellent	42	2.25
2 Very good	437	23.43
3 Good	697	37.37
4 Fair	514	27.56
5 Poor	175	9.38
Total	1,865	100.00

- **fm044 : Spouse Already Processed Retirement When You Did**

	No	%
1 Yes	544	29.28
2 No	1,314	70.72
Total	1,858	100.00

- **fm045 : Spouse's Economic Activities When You Processed Retirement**

	No	%
1 Employed by another person or company and received a wage	943	51.11
2 Ran own business	77	4.17
3 Non-employed and looking for a job	22	1.19
4 Non-employed and not looking for a jobor only doing household	486	26.34

5 Farming	317	17.18
Total	1,845	100.00

- **fm046 : Spouse's Health When You Processed Retirement**

	No	%
1 Excellent	23	1.24
2 Very good	374	20.19
3 Good	711	38.39
4 Fair	530	28.62
5 Poor	214	11.56
Total	1,852	100.00

- **fm047 : Father Was Alive When You Processed Retirement**

	No	%
1 Yes	437	21.74
2 No	1,573	78.26
Total	2,010	100.00

- **fm048 : Father's Health When You Processed Retirement**

	No	%
1 Excellent	4	0.90
2 Very good	68	15.38
3 Good	134	30.32
4 Fair	136	30.77
5 Poor	100	22.62
Total	442	100.00

- **fm049 : Mother Was Alive When You Processed Retirement**

	No	%
1 Yes	722	35.90
2 No	1,289	64.10
Total	2,011	100.00

- **fm050 : Mother's Health When You Processed Retirement**

	No	%
1 Excellent	7	0.97
2 Very good	97	13.40
3 Good	229	31.63

4 Fair	237	32.73
5 Poor	154	21.27
Total	724	100.00

- **fm051 : Num. of Grandchildren below Age 6 When You Processed Retirement**

	No	%
0	1,257	63.04
1	402	20.16
2	203	10.18
3	68	3.41
4	37	1.86
5	14	0.70
6	4	0.20
7	5	0.25
8	1	0.05
10	2	0.10
11	1	0.05
Total	1,994	100.00

- **fm052 : Did You Work after Processed Retirement**

	No	%
1 Yes	262	16.82
2 No	1,296	83.18
Total	1,558	100.00

- **fm053 : How Long did You Start to Work Again after Processed Retirement**

Mean	Min	Max	OBS
1.3	0.0	25.0	702

- **fm054 : Currently Engaged in Paid Small Pastime Work**

	No	%
1 Yes	39	2.51
2 No	1,517	97.49
Total	1,556	100.00

- **fm055_gb : Pasttime Job Occupation Code (GB)**

	No	%
10544	3	8.82
10545	1	2.94
10546	2	5.88
10548	1	2.94
10552	1	2.94
20501	1	2.94
20603	1	2.94
20709	1	2.94
20909	2	5.88
21001	1	2.94
30101	1	2.94
30102	2	5.88
30202	1	2.94
30900	1	2.94
40101	3	8.82
40105	1	2.94
40109	1	2.94
40403	1	2.94
50101	1	2.94
50301	1	2.94
50302	1	2.94
50401	1	2.94
60405	1	2.94
61101	1	2.94
61506	1	2.94
62202	1	2.94
62501	1	2.94
Total	34	100.00

- **fm055_indc : Pasttime Job Industry Code**

	No	%
1	4	11.76
10	2	5.88
12	1	2.94
16	3	8.82
17	1	2.94
18	3	8.82
19	1	2.94
3	5	14.71
5	3	8.82
6	2	5.88
8	7	20.59
9	2	5.88
Total	34	100.00

- **fm055_isco : Pasttime Job Occupation Code (ISCO)**

	No	%
1314	3	9.68
1316	2	6.45
2221	1	3.23
2359	2	6.45
2411	1	3.23
2451	1	3.23
3150	1	3.23
3419	1	3.23
3430	1	3.23
4110	2	6.45
4190	1	3.23
5132	1	3.23
5169	1	3.23
5200	1	3.23
5220	3	9.68
6111	1	3.23
6121	1	3.23
6122	1	3.23
6151	1	3.23
7122	1	3.23
7224	1	3.23
7433	1	3.23
8253	1	3.23
9161	1	3.23
Total	31	100.00

- **fm056_1 : Year**

Mean	Min	Max	OBS
2,000.4	1,965.0	2,011.0	38

- **fm056_2 : Month**

	No	%
0	13	34.21
1	5	13.16
3	5	13.16
4	1	2.63
6	4	10.53
7	1	2.63
8	2	5.26
9	1	2.63
10	3	7.89
11	3	7.89
Total	38	100.00

- **fm057 : Days Worked Per Week**

	No	%
1	1	2.56
2	2	5.13
3	3	7.69
5	6	15.38
6	1	2.56
7	26	66.67
Total	39	100.00

- **fm058 : Hours Worked Per Week**

	No	%
1	2	5.41
2	3	8.11
4	1	2.70
5	1	2.70
6	5	13.51
8	6	16.22
10	3	8.11
12	3	8.11
15	2	5.41
20	1	2.70
30	1	2.70
35	1	2.70
42	1	2.70
48	1	2.70
56	2	5.41
60	1	2.70
70	2	5.41
84	1	2.70
Total	37	100.00

- **fm059 : Monthly Income**

Mean	Min	Max	OBS
815.0	0.0	3,000.0	36

- **fn001 : Currently Receiving at Least One Kind of Pensions**

	No	%
1 Yes	1,739	9.95
2 No	15,738	90.05
Total	17,477	100.00

- **fn002s1 : Types of Pension You Receive**

	No	%
1 Supplemental pension insurance of the firm	144	100.00
Total	144	100.00

- **fn002s2 : Types of Pension You Receive**

	No	%
2 Commercial pension	55	100.00
Total	55	100.00

- **fn002s3 : Types of Pension You Receive**

	No	%
3 Rural pension	806	100.00
Total	806	100.00

- **fn002s4 : Types of Pension You Receive**

	No	%
4 Residents pension	141	100.00
Total	141	100.00

- **fn002s5 : Types of Pension You Receive**

	No	%
5 Urban residents pension	291	100.00
Total	291	100.00

- **fn002s6 : Types of Pension You Receive**

	No	%
6 Pension subsidy to the oldest old	239	100.00
Total	239	100.00

- **fn002s7 : Types of Pension You Receive**

	No	%
7 Other	242	100.00

Total	242	100.00

- **fn003_1 : Year**

Mean	Min	Max	OBS
2,002.9	1,977.0	2,021.0	115

- **fn003_2 : Month**

	No	%
0	27	23.48
1	13	11.30
2	5	4.35
3	8	6.96
4	2	1.74
5	9	7.83
6	5	4.35
7	6	5.22
8	8	6.96
9	7	6.09
10	11	9.57
11	5	4.35
12	9	7.83
Total	115	100.00

- **fn004 : Monthly Benefit**

Mean	Min	Max	OBS
1,131.7	0.0	3,200.0	112

- **fn005_a : Min Bracket**

Mean	Min	Max	OBS
1,125.1	0.0	5,001.0	8

- **fn005_b : Max Bracket**

Mean	Min	Max	OBS
37,500,560.9	499.0	99,999,996.0	8

- **fn006_1 : Year**

Mean	Min	Max	OBS
2,005.7	1,983.0	2,026.0	42

- **fn006_2 : Month**

	No	%
0	18	41.86
1	4	9.30
3	2	4.65
4	1	2.33
5	2	4.65
6	1	2.33
7	1	2.33
8	2	4.65
9	4	9.30
10	2	4.65
11	4	9.30
12	2	4.65
Total	43	100.00

- **fn007 : Monthly Benefit**

Mean	Min	Max	OBS
933.4	0.0	3,000.0	34

- **fn008_a : Min Bracket**

Mean	Min	Max	OBS
333.3	0.0	1,000.0	3

- **fn008_b : Max Bracket**

Mean	Min	Max	OBS
33,333,831.7	499.0	99,999,996.0	3

- **fn009 : Have You Ever Contributed to Your Pension**

	No	%
1 Yes	655	53.51
2 No	569	46.49
Total	1,224	100.00

- **fn010_1 : Year**

Mean	Min	Max	OBS
2,005.4	1,965.0	2,011.0	571

- **fn010_2 : Month**

	No	%
0	246	43.23
1	61	10.72
2	13	2.28
3	33	5.80
4	20	3.51
5	17	2.99
6	20	3.51
7	31	5.45
8	18	3.16
9	21	3.69
10	30	5.27
11	23	4.04
12	36	6.33
Total	569	100.00

- **fn011 : Annual or Lump Sum**

	No	%
1 Annual	310	54.77
2 Lump Sum	256	45.23
Total	566	100.00

- **fn011_1 : Annual Contribution**

Mean	Min	Max	OBS
782.4	0.0	18,000.0	310

- **fn011_2 : Annual Subsidy from the Collective**

Mean	Min	Max	OBS
23.8	-9,999.0	4,200.0	310

- **fn011_3 : Annual Subsidy from the Government**

Mean	Min	Max	OBS

79.5	-9,999.0	7,200.0	310

- **fn011_4 : Lump Sum Contribution**

Mean	Min	Max	OBS
15,081.6	3.0	260,000.0	255

- **fn011_5 : Lump Sum Subsidy from the Collective**

Mean	Min	Max	OBS
388.3	0.0	30,000.0	255

- **fn011_6 : Lump Sum Subsidy from the Government**

Mean	Min	Max	OBS
313.5	0.0	40,000.0	255

- **fn012_1 : Year**

Mean	Min	Max	OBS
2,008.0	1,900.0	2,026.0	1,107

- **fn012_2 : Month**

	No	%
0	284	25.65
1	283	25.56
2	40	3.61
3	51	4.61
4	43	3.88
5	40	3.61
6	46	4.16
7	74	6.68
8	31	2.80
9	47	4.25
10	72	6.50
11	32	2.89
12	64	5.78
Total	1,107	100.00

- **fn013 : Amount Per Month Receive Now**

Mean	Min	Max	OBS
408.9	0.0	5,000.0	1,141

- **fn014_a : Min Bracket**

Mean	Min	Max	OBS
148.7	0.0	1,001.0	37

- **fn014_b : Max Bracket**

Mean	Min	Max	OBS
32,432,903.6	499.0	99,999,996.0	37

- **fn015 : Resident's Pension Was Transfered from other Pension**

	No	%
1 Yes	27	20.45
2 No	105	79.55
Total	132	100.00

- **fn016 : Transfered from**

	No	%
1 Rural pension	4	15.38
2 Basic pension of the firms	21	80.77
3 Other	1	3.85
Total	26	100.00

- **fn017_1 : Year**

Mean	Min	Max	OBS
2,009.7	1,996.0	2,011.0	227

- **fn017_2 : Month**

	No	%
0	45	19.82
1	96	42.29
2	7	3.08

3	3	1.32
4	7	3.08
5	6	2.64
6	5	2.20
7	15	6.61
8	2	0.88
9	5	2.20
10	23	10.13
11	2	0.88
12	11	4.85
Total	227	100.00

- **fn018 : Amount Per Month Received**

Mean	Min	Max	OBS
87.3	0.0	900.0	236

- **fn019_a : Min Bracket**

	No	%
0	1	100.00
Total	1	100.00

- **fn019_b : Max Bracket**

Mean	Min	Max	OBS
499.0	499.0	499.0	1

- **fn021_1 : Year**

Mean	Min	Max	OBS
2,003.0	1,900.0	2,022.0	185

- **fn021_2 : Month**

	No	%
0	48	25.95
1	25	13.51
2	11	5.95
3	10	5.41
4	6	3.24
5	9	4.86
6	4	2.16

7	11	5.95
8	12	6.49
9	10	5.41
10	18	9.73
11	4	2.16
12	17	9.19
Total	185	100.00

- **fn022 : Amount Per Month Received**

Mean	Min	Max	OBS
938.2	0.0	3,300.0	185

- **fn023_a : Min Bracket**

	No	%
0	2	100.00
Total	2	100.00

- **fn023_b : Max Bracket**

Mean	Min	Max	OBS
99,999,996.0	99,999,996.0	99,999,996.0	2

- **fn024 : Enrolled in Pension**

	No	%
1 Yes, pension program of the government and institutions	465	3.10
2 Yes, basic pension insurance of the firms	340	2.27
3 No	14,199	94.63
Total	15,004	100.00

- **fn025 : From Which did You Get Pension**

	No	%
1 Current work unit EP027	437	49.94
2 Last work unit EP146	137	15.66
3 The work unit that processed retirement for the respondent EP169	40	4.57
4 None of the above	261	29.83
Total	875	100.00

- **fn027 : Type of Work Unit**

	No	%
1 Government	167	72.61
2 Institutions	20	8.70
3 NGO	1	0.43
4 Firm	18	7.83
5 Individual	6	2.61
6 Other	18	7.83
Total	230	100.00

- **fn028_1 : Province**

	No	%
01	21	9.38
03	20	8.93
05	29	12.95
06	3	1.34
07	4	1.79
10	7	3.13
11	8	3.57
12	5	2.23
13	15	6.70
14	9	4.02
15	5	2.23
16	4	1.79
17	1	0.45
18	4	1.79
19	9	4.02
20	15	6.70
21	8	3.57
24	2	0.89
26	5	2.23
27	7	3.13
28	8	3.57
29	20	8.93
32	3	1.34
33	12	5.36
Total	224	100.00

- **fn028_2 : City**

	No	%
01	4	1.79
02	1	0.45
04	10	4.48
11	33	14.80
16	1	0.45

17	3	1.35
24	8	3.59
27	2	0.90
40	47	21.08
46	13	5.83
49	5	2.24
53	8	3.59
55	9	4.04
60	14	6.28
63	2	0.90
66	1	0.45
74	15	6.73
76	17	7.62
82	25	11.21
86	5	2.24
Total	223	100.00

- **fn028_3 : County**

	No	%
02	25	11.42
03	1	0.46
04	13	5.94
06	35	15.98
16	3	1.37
28	12	5.48
31	18	8.22
33	20	9.13
37	12	5.48
39	4	1.83
43	3	1.37
44	2	0.91
45	3	1.37
46	2	0.91
51	18	8.22
54	8	3.65
56	2	0.91
58	1	0.46
59	18	8.22
63	3	1.37
76	4	1.83
78	1	0.46
81	1	0.46
89	2	0.91
90	2	0.91
91	3	1.37
99	3	1.37
Total	219	100.00

- **fn029_1 : Year**

Mean	Min	Max	OBS
1,995.4	1,949.0	2,011.0	264

- **fn029_2 : Month**

	No	%
0	125	47.35
1	31	11.74
2	2	0.76
3	8	3.03
4	13	4.92
5	11	4.17
6	15	5.68
7	10	3.79
8	7	2.65
9	7	2.65
10	14	5.30
11	9	3.41
12	12	4.55
Total	264	100.00

- **fn030 : Ever Participated in Basic Pension Insurance for More Than 10 Years**

	No	%
1 Yes	21	6.18
2 No	319	93.82
Total	340	100.00

- **fn031 : Province**

	No	%
05	1	4.55
06	1	4.55
08	1	4.55
10	5	22.73
11	2	9.09
14	1	4.55
15	1	4.55
16	1	4.55
18	1	4.55
20	4	18.18
28	1	4.55
32	1	4.55
33	1	4.55

34	1	4.55
Total	22	100.00

- **fn032 : Years Included in this Program**

Mean	Min	Max	OBS
21.7	0.0	60.0	737

- **fn033 : Years Included in this Program When You Retire**

	No	%
0	61	8.46
1	1	0.14
2	3	0.42
3	4	0.55
4	5	0.69
5	7	0.97
6	5	0.69
7	5	0.69
9	3	0.42
10	10	1.39
11	3	0.42
12	5	0.69
13	7	0.97
14	5	0.69
15	54	7.49
16	5	0.69
17	8	1.11
18	13	1.80
19	9	1.25
20	25	3.47
21	4	0.55
22	4	0.55
23	6	0.83
24	7	0.97
25	19	2.64
26	9	1.25
27	5	0.69
28	9	1.25
29	10	1.39
30	50	6.93
31	17	2.36
32	29	4.02
33	15	2.08
34	21	2.91
35	30	4.16
36	27	3.74
37	19	2.64
38	27	3.74

39	13	1.80
40	49	6.80
41	20	2.77
42	30	4.16
43	29	4.02
44	14	1.94
45	14	1.94
46	4	0.55
50	1	0.14
60	1	0.14
Total	721	100.00

- **fn034 : Enough Years to Receive Pension**

	No	%
1 Yes	674	88.45
2 No	88	11.55
Total	762	100.00

- **fn035 : What do You Plan to Do**

	No	%
1 I will pay the remaining premiums all in one payment at retirement to qualify for pension	36	45.00
2 I will receive a one-time payment at retirement and not get pension	10	12.50
3 I will not receive pension	34	42.50
Total	80	100.00

- **fn036 : Amount or Percentage**

	No	%
1 Yuan per month	431	87.25
2 % of final pay	63	12.75
Total	494	100.00

- **fn036_1 : Amount**

Mean	Min	Max	OBS
1,588.5	0.0	10,000.0	428

- **fn036_2 : Percentage**

	No	%
30	1	1.59

50	2	3.17
60	4	6.35
65	1	1.59
70	2	3.17
80	11	17.46
85	8	12.70
90	14	22.22
95	7	11.11
97	1	1.59
100	12	19.05
Total	63	100.00

- **fn037_a : Min Bracket**

Mean	Min	Max	OBS
1,284.4	0.0	5,001.0	213

- **fn037_b : Max Bracket**

Mean	Min	Max	OBS
19,250,449.5	499.0	99,999,996.0	213

- **fn038 : Enrolled in Pension Programs**

	No	%
1 Yes	1,109	6.35
2 No	16,348	93.65
Total	17,457	100.00

- **fn039s1 : Enrolled in Pension Program**

	No	%
1 Supplemental pension insurance of the firm	36	100.00
Total	36	100.00

- **fn039s2 : Enrolled in Pension Program**

	No	%
2 Commercial pension	107	100.00
Total	107	100.00

- **fn039s3 : Enrolled in Pension Program**

	No	%
3 Rural pension	877	100.00
Total	877	100.00

- **fn039s4 : Enrolled in Pension Program**

	No	%
4 Residents pension	66	100.00
Total	66	100.00

- **fn039s5 : Enrolled in Pension Program**

	No	%
5 Urban residents pension	85	100.00
Total	85	100.00

- **fn039s6 : Enrolled in Pension Program**

	No	%
6 Other pension	36	100.00
Total	36	100.00

- **fn040 : Type of Retirement Pension**

	No	%
1 Defined Benefit DB Retirement Pension	12	38.71
2 Defined Contribution DC Retirement Pension	19	61.29
Total	31	100.00

- **fn041 : Years You Have Been Included in this Plan**

	No	%
0	1	3.70
1	4	14.81
2	1	3.70
3	1	3.70
4	2	7.41
5	1	3.70
6	1	3.70
9	1	3.70
10	1	3.70
15	4	14.81

16	1	3.70
18	1	3.70
19	1	3.70
20	1	3.70
23	1	3.70
28	1	3.70
29	1	3.70
30	1	3.70
40	2	7.41
Total	27	100.00

- **fn042 : When to Start Receiving Benefits**

	No	%
1 At age or in years	20	76.92
4 Other	6	23.08
Total	26	100.00

- **fn042_1 : Age**

	No	%
47	1	5.56
55	3	16.67
60	10	55.56
61	2	11.11
74	1	5.56
80	1	5.56
Total	18	100.00

- **fn042_2 : Years**

	No	%
0	1	100.00
Total	1	100.00

- **fn043_1 : Contribution from You and Your Employer**

Mean	Min	Max	OBS
9,528.2	0.0	36,000.0	11

- **fn043_2 : Contribution from You**

	No	%

0	2	18.18
30	1	9.09
50	1	9.09
60	1	9.09
90	1	9.09
100	5	45.45
Total	11	100.00

- **fn044_1 : Yuan Per Month**

Mean	Min	Max	OBS
1,240.0	600.0	1,800.0	5

- **fn044_2 : Percentage**

No Observations

- **fn044_3 : Lump Sum Amount**

No Observations

- **fn045_a : Min Bracket**

No Observations

- **fn045_b : Max Bracket**

No Observations

- **fn046 : Ever Checked Your Account Balance**

	No	%
2 No	11	100.00
Total	11	100.00

- **fn046_1 : Yuan**

No Observations

- **fn046_2 : Year**

No Observations

- **fn046_3 : Month**

No Observations

- **fn047 : Earliest Age**

	No	%
55	1	9.09
60	8	72.73
62	1	9.09
74	1	9.09
Total	11	100.00

- **fn048 : Amount or Percentage**

	No	%
1 By %	3	50.00
2 Yuan	3	50.00
Total	6	100.00

- **fn048_1 : Percentage**

	No	%
0	1	33.33
50	2	66.67
Total	3	100.00

- **fn048_2 : Amount**

Mean	Min	Max	OBS

933.3	200.0	1,600.0	3

- **fn049 : How Much Cash Settlements did You Receive**

No Observations

- **fn050 : Who Paid for the Commercial Pension Insurance**

	No	%
1 Myself	84	81.55
2 My employer	7	6.80
3 My family or relative	10	9.71
4 Other people	2	1.94
Total	103	100.00

- **fn051_1 : Year**

Mean	Min	Max	OBS
2,003.7	1,986.0	2,011.0	101

- **fn051_2 : Month**

	No	%
0	36	35.64
1	8	7.92
2	5	4.95
3	9	8.91
4	2	1.98
5	5	4.95
6	6	5.94
7	9	8.91
8	3	2.97
9	6	5.94
10	4	3.96
11	4	3.96
12	4	3.96
Total	101	100.00

- **fn052 : How do You Contribute to the Commercial Pension**

	No	%

1 Annual payment	84	83.17
2 Lump sum amount	17	16.83
Total	101	100.00

- **fn053 : Contribution from You**

Mean	Min	Max	OBS
3,206.2	0.0	36,000.0	81

- **fn054 : Years**

	No	%
0	1	1.28
3	2	2.56
5	3	3.85
7	3	3.85
8	1	1.28
10	22	28.21
12	4	5.13
15	12	15.38
17	1	1.28
20	27	34.62
23	1	1.28
30	1	1.28
Total	78	100.00

- **fn055 : Amount You Need to Pay in Total**

Mean	Min	Max	OBS
21,617.6	200.0	60,000.0	17

- **fn056 : How do You Receive the Pension**

	No	%
1 Lump sum amount	28	31.11
2 Yearly	22	24.44
3 Monthly	40	44.44
Total	90	100.00

- **fn057 : Amount You Expect to Receive after Retirement**

Mean	Min	Max	OBS
2,370.6	0.0	10,000.0	18

- **fn058 : Amount You Expect to Receive Per Month in the Future**

Mean	Min	Max	OBS
821.9	60.0	4,000.0	32

- **fn059 : Amount You Expect to Receive in Total**

Mean	Min	Max	OBS
84,019.3	0.0	600,000.0	51

- **fn060_1 : Year**

Mean	Min	Max	OBS
2,007.1	1,900.0	2,011.0	897

- **fn060_2 : Month**

	No	%
0	377	42.03
1	97	10.81
2	38	4.24
3	41	4.57
4	33	3.68
5	65	7.25
6	29	3.23
7	61	6.80
8	28	3.12
9	28	3.12
10	50	5.57
11	19	2.12
12	31	3.46
Total	897	100.00

- **fn061_1 : Your Annual Contribution**

Mean	Min	Max	OBS
1,354.3	0.0	42,000.0	915

- **fn061_2 : Subsidy from the Collective**

Mean	Min	Max	OBS
59.7	0.0	25,000.0	846

- **fn061_3 : Subsidy from the Government**

Mean	Min	Max	OBS
86.0	0.0	32,000.0	850

- **fn062 : Age or Years**

	No	%
1 At age years	932	96.28
2 In years	36	3.72
Total	968	100.00

- **fn062_1 : Age**

	No	%
50	26	2.79
51	5	0.54
53	1	0.11
54	2	0.21
55	55	5.90
56	10	1.07
57	1	0.11
58	2	0.21
59	3	0.32
60	709	76.07
61	37	3.97
62	6	0.64
63	11	1.18
64	3	0.32
65	11	1.18
66	2	0.21
67	3	0.32
68	4	0.43
69	3	0.32
70	7	0.75
71	4	0.43
73	2	0.21
74	4	0.43
75	6	0.64
77	1	0.11
78	2	0.21
80	7	0.75
82	1	0.11

83	1	0.11
85	1	0.11
90	1	0.11
100	1	0.11
Total	932	100.00

- **fn062_2 : Years**

	No	%
0	6	16.67
1	12	33.33
2	5	13.89
3	2	5.56
5	2	5.56
6	1	2.78
7	1	2.78
10	1	2.78
12	1	2.78
13	1	2.78
15	3	8.33
78	1	2.78
Total	36	100.00

- **fn063 : Yuan Per Month or Lump Sum Amount**

	No	%
1 Yuan per month	858	99.19
2 Yuan Lump sum amount	7	0.81
Total	865	100.00

- **fn063_1 : Amount Per Month**

Mean	Min	Max	OBS
183.6	0.0	3,600.0	858

- **fn063_2 : Lump Sum Amount**

Mean	Min	Max	OBS
4,650.7	100.0	30,000.0	7

- **fn064_1 : Unfolding Bracket**

	No	%

1 Less than 250 yuan	47	75.81
2 About 250 yuan	7	11.29
3 More than 250 yuan	8	12.90
Total	62	100.00

- **fn064_2 : Unfolding Bracket**

	No	%
1 Less than 500 yuan	72	68.57
2 About 500 yuan	12	11.43
3 More than 500 yuan	21	20.00
Total	105	100.00

- **fn064_3 : Unfolding Bracket**

	No	%
1 Less than 1,000 yuan	10	47.62
2 About 1,000 yuan	6	28.57
3 More than 1,000 yuan	5	23.81
Total	21	100.00

- **fn064_4 : Unfolding Bracket**

	No	%
1 Less than 2,000 yuan	5	100.00
Total	5	100.00

- **fn065 : Was Your Residents' Pension Transfered from other Pension**

	No	%
1 Yes	10	15.87
2 No	53	84.13
Total	63	100.00

- **fn066 : Transfered from**

	No	%
1 Rural pension	6	60.00
2 Basic pension insurance of the firms	3	30.00
3 Other	1	10.00
Total	10	100.00

- **fn068_1 : Age**

	No	%
50	2	8.00
55	5	20.00
60	17	68.00
65	1	4.00
Total	25	100.00

- **fn068_2 : Years**

Mean	Min	Max	OBS
678.0	8.0	2,011.0	3

- **fn069 : Yuan Per Month or Lump Sum Amount**

	No	%
1 Yuan per month	18	94.74
2 Yuan lump sum amount	1	5.26
Total	19	100.00

- **fn069_1 : Amount Per Month**

Mean	Min	Max	OBS
690.9	55.0	2,000.0	18

- **fn069_2 : Lump Sum Amount**

	No	%
26	1	100.00
Total	1	100.00

- **fn070_1 : Unfolding Bracket**

	No	%
2 About 250 yuan	1	100.00
Total	1	100.00

- **fn070_2 : Unfolding Bracket**

	No	%

1 Less than 500 yuan	1	16.67
3 More than 500 yuan	5	83.33
Total	6	100.00

- **fn070_3 : Unfolding Bracket**

	No	%
1 Less than 1,000 yuan	1	25.00
3 More than 1,000 yuan	3	75.00
Total	4	100.00

- **fn070_4 : Unfolding Bracket**

	No	%
1 Less than 2,000 yuan	3	75.00
3 More than 2,000 yuan	1	25.00
Total	4	100.00

- **fn071 : Participated in NRSP**

	No	%
1 Yes	3,763	21.59
2 No	13,665	78.41
Total	17,428	100.00

- **fn072s1 : Reason for Not Participate in NRSP**

	No	%
1 The New Rural Social Pension has not been introduced in my local area.	7,098	100.00
Total	7,098	100.00

- **fn072s2 : Reason for Not Participate in NRSP**

	No	%
2 I lack money.	2,934	100.00
Total	2,934	100.00

- **fn072s3 : Reason for Not Participate in NRSP**

	No	%

3 I am not satisfied with the benefits because it isnt cost-effective.	181	100.00
Total	181	100.00

- **fn072s4 : Reason for Not Participate in NRSP**

	No	%
4 The benefits are poor, and mean nothing to my life.	114	100.00
Total	114	100.00

- **fn072s5 : Reason for Not Participate in NRSP**

	No	%
5 Application and payment arrangements make it inconvenient to participate.	74	100.00
Total	74	100.00

- **fn072s6 : Reason for Not Participate in NRSP**

	No	%
6 The mechanism for making contributions is not justifiable.	23	100.00
Total	23	100.00

- **fn072s7 : Reason for Not Participate in NRSP**

	No	%
7 I do not have a local Hukou.	298	100.00
Total	298	100.00

- **fn072s8 : Reason for Not Participate in NRSP**

	No	%
8 I am already covered by other social pension insurance.	1,486	100.00
Total	1,486	100.00

- **fn072s9 : Reason for Not Participate in NRSP**

	No	%
9 Other .	2,344	100.00
Total	2,344	100.00

- **fn073_1 : Year**

Mean	Min	Max	OBS
2,010.0	2,008.0	2,011.0	3,693

- **fn073_2 : Month**

	No	%
0	826	22.26
1	442	11.91
2	107	2.88
3	232	6.25
4	203	5.47
5	148	3.99
6	262	7.06
7	455	12.26
8	127	3.42
9	77	2.08
10	460	12.40
11	173	4.66
12	198	5.34
Total	3,710	100.00

- **fn074 : How do You Contribute to NRSP**

	No	%
1 Annual payment	2,373	63.53
2 Lump sum amount	168	4.50
3 I don't need to pay myself	1,194	31.97
Total	3,735	100.00

- **fn074_1 : Annual Payment**

Mean	Min	Max	OBS
208.8	0.0	10,000.0	2,382

- **fn074_2 : Lump Sum Amount**

Mean	Min	Max	OBS
10,040.7	0.0	60,000.0	173

- **fn074_3 : Annual Payment**

Mean	Min	Max	OBS

913.2	0.0	60,000.0	169

- **fn075 : Num. Years Need to Pay to Receive Benefits**

	No	%
0	137	6.14
1	147	6.59
2	130	5.82
3	141	6.32
4	104	4.66
5	142	6.36
6	133	5.96
7	101	4.53
8	81	3.63
9	70	3.14
10	108	4.84
11	88	3.94
12	102	4.57
13	93	4.17
14	65	2.91
15	532	23.84
16	19	0.85
17	14	0.63
18	4	0.18
19	2	0.09
20	11	0.49
21	1	0.04
22	2	0.09
24	1	0.04
29	1	0.04
30	1	0.04
40	1	0.04
55	1	0.04
Total	2,232	100.00

- **fn076 : Who Pays for Your NRSP**

	No	%
1 Myself	2,120	84.03
2 My children	283	11.22
3 Other family member or relative	70	2.77
4 Others	50	1.98
Total	2,523	100.00

- **fn077 : Did You Start to Receive Benefits from NRSP**

	No	%

1 Yes	1,125	29.90
2 No	2,637	70.10
Total	3,762	100.00

- **fn078_1 : Year**

Mean	Min	Max	OBS
2,010.1	2,008.0	2,011.0	1,105

- **fn078_2 : Month**

	No	%
0	196	17.75
1	262	23.73
2	75	6.79
3	89	8.06
4	45	4.08
5	19	1.72
6	42	3.80
7	111	10.05
8	13	1.18
9	31	2.81
10	123	11.14
11	37	3.35
12	61	5.53
Total	1,104	100.00

- **fn079 : Amount Received Per Month**

Mean	Min	Max	OBS
84.9	0.0	1,100.0	1,118

- **fn080 : The Way You Can rely on for Old-Age Support**

	No	%
1 Children	11,671	68.28
2 Savings	692	4.05
3 Pension or retirement salary	3,687	21.57
4 Commercial pension insurance	89	0.52
5 Other	955	5.59
Total	17,094	100.00

- **fn081 : Assistance in Section E**

	No	%
1 Never	13,385	76.45
2 A few times	3,122	17.83
3 Most or all the time	566	3.23
4 The section was done by a proxy reporter	435	2.48
Total	17,508	100.00

- **fn082 : Relationship to R**

	No	%
1 Spouse	300	67.87
2 Mother	3	0.68
3 Father	4	0.90
4 Mother-in-law	4	0.90
6 Sibling	4	0.90
7 Brother-in-law, sister-in-law	2	0.45
8 Child	77	17.42
9 Spouse of child	35	7.92
10 Grandchild	7	1.58
11 Other relative	4	0.90
12 Helper or other non-relative	2	0.45
Total	442	100.00

- **fn083 : Reason for Proxy**

	No	%
1 The respondent has serious physical handicaps	69	16.24
2 The respondent has serious mental handicaps	22	5.18
3 The respondent has rejected this interview	45	10.59
4 Other	289	68.00
Total	425	100.00

- **proxy : Interview Down By Proxy**

	No	%
0 No	16,188	92.39
1 Yes	1,333	7.61
Total	17,521	100.00

8 Household Income

- **householdID : Household ID**

A String Variable	
OBS:	10,026

- **communityID : Community ID**

A String Variable	
OBS:	10,026

- **ga005_1_ : Did This Household Member Receive Any Wage And Bonus Income in the Past Year**

	No	%
1 Yes	201	26.21
2 No	566	73.79
Total	767	100.00

- **ga005_2_ : Did This Household Member Receive Any Wage And Bonus Income in the Past Year**

	No	%
1 Yes	554	31.16
2 No	1,224	68.84
Total	1,778	100.00

- **ga005_3_ : Did This Household Member Receive Any Wage And Bonus Income in the Past Year**

	No	%
1 Yes	2,090	36.19
2 No	3,685	63.81
Total	5,775	100.00

- **ga005_4_ : Did This Household Member Receive Any Wage And Bonus Income in the Past Year**

	No	%
1 Yes	1,254	30.02

2 No	2,923	69.98
Total	4,177	100.00

- **ga005_5_ : Did This Household Member Receive Any Wage And Bonus Income in the Past Year**

	No	%
1 Yes	586	21.66
2 No	2,120	78.34
Total	2,706	100.00

- **ga005_6_ : Did This Household Member Receive Any Wage And Bonus Income in the Past Year**

	No	%
1 Yes	311	23.31
2 No	1,023	76.69
Total	1,334	100.00

- **ga005_7_ : Did This Household Member Receive Any Wage And Bonus Income in the Past Year**

	No	%
1 Yes	144	28.02
2 No	370	71.98
Total	514	100.00

- **ga005_8_ : Did This Household Member Receive Any Wage And Bonus Income in the Past Year**

	No	%
1 Yes	71	29.34
2 No	171	70.66
Total	242	100.00

- **ga005_9_ : Did This Household Member Receive Any Wage And Bonus Income in the Past Year**

	No	%
1 Yes	35	25.93
2 No	100	74.07
Total	135	100.00

- **ga005_10_ : Did This Household Member Receive Any Wage And Bonus Income in the Past Year**

	No	%
1 Yes	11	16.67
2 No	55	83.33
Total	66	100.00

- **ga005_11_ : Did This Household Member Receive Any Wage And Bonus Income in the Past Year**

	No	%
1 Yes	6	18.18
2 No	27	81.82
Total	33	100.00

- **ga005_12_ : Did This Household Member Receive Any Wage And Bonus Income in the Past Year**

	No	%
1 Yes	5	26.32
2 No	14	73.68
Total	19	100.00

- **ga005_13_ : Did This Household Member Receive Any Wage And Bonus Income in the Past Year**

	No	%
1 Yes	2	20.00
2 No	8	80.00
Total	10	100.00

- **ga005_14_ : Did This Household Member Receive Any Wage And Bonus Income in the Past Year**

	No	%
1 Yes	2	40.00
2 No	3	60.00
Total	5	100.00

- **ga005_15_ : Did This Household Member Receive Any Wage And Bonus Income in the Past Year**

	No	%
1 Yes	1	50.00
2 No	1	50.00
Total	2	100.00

- **ga005_16_ : Did This Household Member Receive Any Wage And Bonus Income in the Past Year**

	No	%
2 No	1	100.00
Total	1	100.00

- **ga005_26_ : Did This Household Member Receive Any Wage And Bonus Income in the Past Year**

	No	%
2 No	2	100.00
Total	2	100.00

- **ga006_1_1_ : Salary Per Year**

Mean	Min	Max	OBS
22,335.0	1,000.0	200,000.0	40

- **ga006_1_2_ : Salary Per Year**

Mean	Min	Max	OBS
16,352.3	1.0	150,000.0	109

- **ga006_1_3_ : Salary Per Year**

Mean	Min	Max	OBS
15,837.0	0.0	200,000.0	429

- **ga006_1_4_ : Salary Per Year**

Mean	Min	Max	OBS

15,400.4	0.0	420,000.0	223

- **ga006_1_5_ : Salary Per Year**

Mean	Min	Max	OBS
14,793.8	0.0	200,000.0	97

- **ga006_1_6_ : Salary Per Year**

Mean	Min	Max	OBS
14,686.0	120.0	150,000.0	52

- **ga006_1_7_ : Salary Per Year**

Mean	Min	Max	OBS
13,551.9	150.0	60,000.0	16

- **ga006_1_8_ : Salary Per Year**

Mean	Min	Max	OBS
16,150.0	1,000.0	40,000.0	8

- **ga006_1_9_ : Salary Per Year**

Mean	Min	Max	OBS
10,000.0	2,000.0	18,000.0	3

- **ga006_1_10_ : Salary Per Year**

No Observations

- **ga006_1_11_ : Salary Per Year**

No Observations

- **ga006_1_12_ : Salary Per Year**

No Observations

- **ga006_1_13_ : Salary Per Year**

No Observations

- **ga006_1_14_ : Salary Per Year**

No Observations

- **ga006_1_15_ : Salary Per Year**

No Observations

- **ga006_2_1_ : Salary Per Month**

Mean	Min	Max	OBS
1,785.1	30.0	8,000.0	138

- **ga006_2_2_ : Salary Per Month**

Mean	Min	Max	OBS
1,735.1	0.0	15,000.0	399

- **ga006_2_3_ : Salary Per Month**

Mean	Min	Max	OBS
1,702.1	0.0	10,000.0	1,488

- **ga006_2_4_ : Salary Per Month**

Mean	Min	Max	OBS

1,633.1	0.0	8,000.0	922

- **ga006_2_5_ : Salary Per Month**

Mean	Min	Max	OBS
1,696.1	1.0	15,000.0	436

- **ga006_2_6_ : Salary Per Month**

Mean	Min	Max	OBS
1,673.0	0.0	8,000.0	237

- **ga006_2_7_ : Salary Per Month**

Mean	Min	Max	OBS
1,737.4	200.0	6,000.0	119

- **ga006_2_8_ : Salary Per Month**

Mean	Min	Max	OBS
1,883.9	400.0	8,000.0	56

- **ga006_2_9_ : Salary Per Month**

Mean	Min	Max	OBS
1,783.3	400.0	8,000.0	30

- **ga006_2_10_ : Salary Per Month**

Mean	Min	Max	OBS
2,181.8	1,100.0	7,000.0	11

- **ga006_2_11_ : Salary Per Month**

Mean	Min	Max	OBS
1,933.3	1,500.0	3,000.0	6

- **ga006_2_12_ : Salary Per Month**

Mean	Min	Max	OBS
1,666.7	1,000.0	2,000.0	3

- **ga006_2_13_ : Salary Per Month**

Mean	Min	Max	OBS
2,050.0	1,800.0	2,300.0	2

- **ga006_2_14_ : Salary Per Month**

Mean	Min	Max	OBS
1,700.0	1,500.0	1,900.0	2

- **ga006_2_15_ : Salary Per Month**

Mean	Min	Max	OBS
2,000.0	2,000.0	2,000.0	1

- **ga006_a_1_ : Min of Bracket**

Mean	Min	Max	OBS
9,166.9	0.0	100,001.0	24

- **ga006_a_2_ : Min of Bracket**

Mean	Min	Max	OBS
14,528.6	0.0	100,000.0	53

- **ga006_a_3_ : Min of Bracket**

Mean	Min	Max	OBS
10,995.0	0.0	100,001.0	191

- **ga006_a_4_ : Min of Bracket**

Mean	Min	Max	OBS
7,459.2	0.0	100,000.0	122

- **ga006_a_5_ : Min of Bracket**

Mean	Min	Max	OBS
12,931.2	0.0	100,001.0	58

- **ga006_a_6_ : Min of Bracket**

Mean	Min	Max	OBS
8,518.6	0.0	100,001.0	27

- **ga006_a_7_ : Min of Bracket**

Mean	Min	Max	OBS
6,000.3	0.0	30,001.0	10

- **ga006_a_8_ : Min of Bracket**

Mean	Min	Max	OBS
5,000.0	0.0	10,000.0	8

- **ga006_a_9_ : Min of Bracket**

Mean	Min	Max	OBS
6,667.0	0.0	10,001.0	3

- **ga006_a_12_ : Min of Bracket**

	No	%
0	2	100.00
Total	2	100.00

- **ga006_b_1_ : Max of Bracket**

Mean	Min	Max	OBS
29,188,331.6	9,999.0	99,999,996.0	24

- **ga006_b_2_ : Max of Bracket**

Mean	Min	Max	OBS

24,552,640.0	9,999.0	99,999,996.0	53

- **ga006_b_3_ : Max of Bracket**

Mean	Min	Max	OBS
24,105,548.3	9,999.0	99,999,996.0	191

- **ga006_b_4_ : Max of Bracket**

Mean	Min	Max	OBS
35,262,375.2	9,999.0	99,999,996.0	122

- **ga006_b_5_ : Max of Bracket**

Mean	Min	Max	OBS
32,777,756.9	9,999.0	99,999,996.0	58

- **ga006_b_6_ : Max of Bracket**

Mean	Min	Max	OBS
29,646,665.0	9,999.0	99,999,996.0	27

- **ga006_b_7_ : Max of Bracket**

Mean	Min	Max	OBS
30,014,998.2	9,999.0	99,999,996.0	10

- **ga006_b_8_ : Max of Bracket**

Mean	Min	Max	OBS
12,511,249.1	9,999.0	99,999,996.0	8

- **ga006_b_9_ : Max of Bracket**

Mean	Min	Max	OBS
33,346,665.0	10,000.0	99,999,996.0	3

- **ga006_b_12_ : Max of Bracket**

Mean	Min	Max	OBS
50,014,997.5	29,999.0	99,999,996.0	2

- **ga007_1_s1 : Did this Household Member Receive this Type of Individual Income in Past Year**

	No	%
1 Pensions	93	100.00
Total	93	100.00

- **ga007_1_s2 : Did this Household Member Receive this Type of Individual Income in Past Year**

No Observations

- **ga007_1_s3 : Did this Household Member Receive this Type of Individual Income in Past Year**

	No	%
3 Pension subsidy	3	100.00
Total	3	100.00

- **ga007_1_s4 : Did this Household Member Receive this Type of Individual Income in Past Year**

	No	%
4 Workers compensation	2	100.00
Total	2	100.00

- **ga007_1_s5 : Did this Household Member Receive this Type of Individual Income in Past Year**

	No	%
5 Elderly family planning subsidies	6	100.00
Total	6	100.00

- **ga007_1_s6 : Did this Household Member Receive this Type of Individual**

Income in Past Year

	No	%
6 Medical aid	5	100.00
Total	5	100.00

- **ga007_1_s7 : Did this Household Member Receive this Type of Individual Income in Past Year**

	No	%
7 Other government subsidies	29	100.00
Total	29	100.00

- **ga007_1_s8 : Did this Household Member Receive this Type of Individual Income in Past Year**

No Observations

- **ga007_1_s9 : Did this Household Member Receive this Type of Individual Income in Past Year**

	No	%
9 Other income sources	4	100.00
Total	4	100.00

- **ga007_1_s10 : Did this Household Member Receive this Type of Individual Income in Past Year**

	No	%
10 None of the above	631	100.00
Total	631	100.00

- **ga007_2_s1 : Did this Household Member Receive this Type of Individual Income in Past Year**

	No	%
1 Pensions	49	100.00
Total	49	100.00

- **ga007_2_s2 : Did this Household Member Receive this Type of Individual Income in Past Year**

No Observations

- **ga007_2_s3 : Did this Household Member Receive this Type of Individual Income in Past Year**

	No	%
3 Pension subsidy	5	100.00
Total	5	100.00

- **ga007_2_s4 : Did this Household Member Receive this Type of Individual Income in Past Year**

	No	%
4 Workers compensation	1	100.00
Total	1	100.00

- **ga007_2_s5 : Did this Household Member Receive this Type of Individual Income in Past Year**

	No	%
5 Elderly family planning subsidies	5	100.00
Total	5	100.00

- **ga007_2_s6 : Did this Household Member Receive this Type of Individual Income in Past Year**

	No	%
6 Medical aid	6	100.00
Total	6	100.00

- **ga007_2_s7 : Did this Household Member Receive this Type of Individual Income in Past Year**

	No	%
7 Other government subsidies	37	100.00
Total	37	100.00

- **ga007_2_s8 : Did this Household Member Receive this Type of Individual Income in Past Year**

	No	%
8 Social assistance	2	100.00
Total	2	100.00

- **ga007_2_s9 : Did this Household Member Receive this Type of Individual Income in Past Year**

	No	%
9 Other income sources	10	100.00
Total	10	100.00

- **ga007_2_s10 : Did this Household Member Receive this Type of Individual Income in Past Year**

	No	%
10 None of the above	1,653	100.00
Total	1,653	100.00

- **ga007_3_s1 : Did this Household Member Receive this Type of Individual Income in Past Year**

	No	%
1 Pensions	71	100.00
Total	71	100.00

- **ga007_3_s2 : Did this Household Member Receive this Type of Individual Income in Past Year**

	No	%
2 Unemployment compensation	2	100.00
Total	2	100.00

- **ga007_3_s3 : Did this Household Member Receive this Type of Individual Income in Past Year**

	No	%
3 Pension subsidy	13	100.00
Total	13	100.00

- **ga007_3_s4 : Did this Household Member Receive this Type of Individual Income in Past Year**

	No	%
4 Workers compensation	6	100.00
Total	6	100.00

- **ga007_3_s5 : Did this Household Member Receive this Type of Individual Income in Past Year**

	No	%
5 Elderly family planning subsidies	7	100.00
Total	7	100.00

- **ga007_3_s6 : Did this Household Member Receive this Type of Individual Income in Past Year**

	No	%
6 Medical aid	11	100.00
Total	11	100.00

- **ga007_3_s7 : Did this Household Member Receive this Type of Individual Income in Past Year**

	No	%
7 Other government subsidies	99	100.00
Total	99	100.00

- **ga007_3_s8 : Did this Household Member Receive this Type of Individual Income in Past Year**

	No	%
8 Social assistance	6	100.00
Total	6	100.00

- **ga007_3_s9 : Did this Household Member Receive this Type of Individual Income in Past Year**

	No	%
9 Other income sources	18	100.00
Total	18	100.00

- **ga007_3_s10 : Did this Household Member Receive this Type of Individual Income in Past Year**

	No	%
10 None of the above	5,521	100.00
Total	5,521	100.00

- **ga007_4_s1 : Did this Household Member Receive this Type of Individual Income in Past Year**

	No	%
1 Pensions	33	100.00
Total	33	100.00

- **ga007_4_s2 : Did this Household Member Receive this Type of Individual Income in Past Year**

	No	%
2 Unemployment compensation	1	100.00
Total	1	100.00

- **ga007_4_s3 : Did this Household Member Receive this Type of Individual Income in Past Year**

	No	%
3 Pension subsidy	2	100.00
Total	2	100.00

- **ga007_4_s4 : Did this Household Member Receive this Type of Individual Income in Past Year**

	No	%
4 Workers compensation	3	100.00
Total	3	100.00

- **ga007_4_s5 : Did this Household Member Receive this Type of Individual Income in Past Year**

	No	%
5 Elderly family planning subsidies	4	100.00
Total	4	100.00

- **ga007_4_s6 : Did this Household Member Receive this Type of Individual Income in Past Year**

	No	%
6 Medical aid	4	100.00
Total	4	100.00

- **ga007_4_s7 : Did this Household Member Receive this Type of Individual Income in Past Year**

	No	%
7 Other government subsidies	44	100.00
Total	44	100.00

- **ga007_4_s8 : Did this Household Member Receive this Type of Individual Income in Past Year**

	No	%
8 Social assistance	1	100.00
Total	1	100.00

- **ga007_4_s9 : Did this Household Member Receive this Type of Individual Income in Past Year**

	No	%
9 Other income sources	8	100.00
Total	8	100.00

- **ga007_4_s10 : Did this Household Member Receive this Type of Individual Income in Past Year**

	No	%
10 None of the above	4,066	100.00
Total	4,066	100.00

- **ga007_5_s1 : Did this Household Member Receive this Type of Individual Income in Past Year**

	No	%
1 Pensions	10	100.00
Total	10	100.00

- **ga007_5_s2 : Did this Household Member Receive this Type of Individual Income in Past Year**

No Observations

- **ga007_5_s3 : Did this Household Member Receive this Type of Individual Income in Past Year**

No Observations

- **ga007_5_s4 : Did this Household Member Receive this Type of Individual Income in Past Year**

No Observations

- **ga007_5_s5 : Did this Household Member Receive this Type of Individual Income in Past Year**

	No	%
5 Elderly family planning subsidies	5	100.00
Total	5	100.00

- **ga007_5_s6 : Did this Household Member Receive this Type of Individual Income in Past Year**

	No	%
6 Medical aid	2	100.00
Total	2	100.00

- **ga007_5_s7 : Did this Household Member Receive this Type of Individual Income in Past Year**

	No	%
7 Other government subsidies	25	100.00
Total	25	100.00

- **ga007_5_s8 : Did this Household Member Receive this Type of Individual**

Income in Past Year

	No	%
8 Social assistance	2	100.00
Total	2	100.00

- **ga007_5_s9 : Did this Household Member Receive this Type of Individual Income in Past Year**

	No	%
9 Other income sources	5	100.00
Total	5	100.00

- **ga007_5_s10 : Did this Household Member Receive this Type of Individual Income in Past Year**

	No	%
10 None of the above	2,647	100.00
Total	2,647	100.00

- **ga007_6_s1 : Did this Household Member Receive this Type of Individual Income in Past Year**

	No	%
1 Pensions	4	100.00
Total	4	100.00

- **ga007_6_s2 : Did this Household Member Receive this Type of Individual Income in Past Year**

No Observations

- **ga007_6_s3 : Did this Household Member Receive this Type of Individual Income in Past Year**

	No	%
3 Pension subsidy	3	100.00
Total	3	100.00

- **ga007_6_s4 : Did this Household Member Receive this Type of Individual Income in Past Year**

No Observations

- **ga007_6_s5 : Did this Household Member Receive this Type of Individual Income in Past Year**

	No	%
5 Elderly family planning subsidies	3	100.00
Total	3	100.00

- **ga007_6_s6 : Did this Household Member Receive this Type of Individual Income in Past Year**

No Observations

- **ga007_6_s7 : Did this Household Member Receive this Type of Individual Income in Past Year**

	No	%
7 Other government subsidies	17	100.00
Total	17	100.00

- **ga007_6_s8 : Did this Household Member Receive this Type of Individual Income in Past Year**

No Observations

- **ga007_6_s9 : Did this Household Member Receive this Type of Individual Income in Past Year**

	No	%
9 Other income sources	1	100.00
Total	1	100.00

- **ga007_6_s10 : Did this Household Member Receive this Type of Individ-**

ual Income in Past Year

	No	%
10 None of the above	1,303	100.00
Total	1,303	100.00

- **ga007_7_s1 : Did this Household Member Receive this Type of Individual Income in Past Year**

	No	%
1 Pensions	6	100.00
Total	6	100.00

- **ga007_7_s2 : Did this Household Member Receive this Type of Individual Income in Past Year**

No Observations

- **ga007_7_s3 : Did this Household Member Receive this Type of Individual Income in Past Year**

	No	%
3 Pension subsidy	1	100.00
Total	1	100.00

- **ga007_7_s4 : Did this Household Member Receive this Type of Individual Income in Past Year**

No Observations

- **ga007_7_s5 : Did this Household Member Receive this Type of Individual Income in Past Year**

	No	%
5 Elderly family planning subsidies	1	100.00
Total	1	100.00

- **ga007_7_s6 : Did this Household Member Receive this Type of Individual**

Income in Past Year

	No	%
6 Medical aid	2	100.00
Total	2	100.00

- **ga007_7_s7 : Did this Household Member Receive this Type of Individual Income in Past Year**

	No	%
7 Other government subsidies	2	100.00
Total	2	100.00

- **ga007_7_s8 : Did this Household Member Receive this Type of Individual Income in Past Year**

No Observations

- **ga007_7_s9 : Did this Household Member Receive this Type of Individual Income in Past Year**

No Observations

- **ga007_7_s10 : Did this Household Member Receive this Type of Individual Income in Past Year**

	No	%
10 None of the above	502	100.00
Total	502	100.00

- **ga007_8_s1 : Did this Household Member Receive this Type of Individual Income in Past Year**

	No	%
1 Pensions	2	100.00
Total	2	100.00

- **ga007_8_s2 : Did this Household Member Receive this Type of Individual**

Income in Past Year

No Observations

- **ga007_8_s3 : Did this Household Member Receive this Type of Individual Income in Past Year**

No Observations

- **ga007_8_s4 : Did this Household Member Receive this Type of Individual Income in Past Year**

No Observations

- **ga007_8_s5 : Did this Household Member Receive this Type of Individual Income in Past Year**

No Observations

- **ga007_8_s6 : Did this Household Member Receive this Type of Individual Income in Past Year**

No Observations

- **ga007_8_s7 : Did this Household Member Receive this Type of Individual Income in Past Year**

No Observations

- **ga007_8_s8 : Did this Household Member Receive this Type of Individual Income in Past Year**

No Observations

- **ga007_8_s9 : Did this Household Member Receive this Type of Individual Income in Past Year**

No Observations

- **ga007_8_s10 : Did this Household Member Receive this Type of Individual Income in Past Year**

	No	%
10 None of the above	238	100.00
Total	238	100.00

- **ga007_9_s1 : Did this Household Member Receive this Type of Individual Income in Past Year**

	No	%
1 Pensions	1	100.00
Total	1	100.00

- **ga007_9_s2 : Did this Household Member Receive this Type of Individual Income in Past Year**

No Observations

- **ga007_9_s3 : Did this Household Member Receive this Type of Individual Income in Past Year**

	No	%
3 Pension subsidy	1	100.00
Total	1	100.00

- **ga007_9_s4 : Did this Household Member Receive this Type of Individual Income in Past Year**

No Observations

- **ga007_9_s5 : Did this Household Member Receive this Type of Individual Income in Past Year**

No Observations

- **ga007_9_s6 : Did this Household Member Receive this Type of Individual Income in Past Year**

No Observations

- **ga007_9_s7 : Did this Household Member Receive this Type of Individual Income in Past Year**

No Observations

- **ga007_9_s8 : Did this Household Member Receive this Type of Individual Income in Past Year**

No Observations

- **ga007_9_s9 : Did this Household Member Receive this Type of Individual Income in Past Year**

No Observations

- **ga007_9_s10 : Did this Household Member Receive this Type of Individual Income in Past Year**

	No	%
10 None of the above	133	100.00
Total	133	100.00

- **ga007_10_s1 : Did this Household Member Receive this Type of Individual Income in Past Year**

No Observations

- **ga007_10_s2 : Did this Household Member Receive this Type of Individual Income in Past Year**

No Observations

- **ga007_10_s3 : Did this Household Member Receive this Type of Individual Income in Past Year**

No Observations

- **ga007_10_s4 : Did this Household Member Receive this Type of Individual Income in Past Year**

No Observations

- **ga007_10_s5 : Did this Household Member Receive this Type of Individual Income in Past Year**

No Observations

- **ga007_10_s6 : Did this Household Member Receive this Type of Individual Income in Past Year**

No Observations

- **ga007_10_s7 : Did this Household Member Receive this Type of Individual Income in Past Year**

No Observations

- **ga007_10_s8 : Did this Household Member Receive this Type of Individual Income in Past Year**

No Observations

- **ga007_10_s9 : Did this Household Member Receive this Type of Individual Income in Past Year**

No Observations

- **ga007_10_s10 : Did this Household Member Receive this Type of Individual Income in Past Year**

	No	%
10 None of the above	66	100.00
Total	66	100.00

- **ga007_11_s1 : Did this Household Member Receive this Type of Individual Income in Past Year**

No Observations

- **ga007_11_s2 : Did this Household Member Receive this Type of Individual Income in Past Year**

No Observations

- **ga007_11_s3 : Did this Household Member Receive this Type of Individual Income in Past Year**

	No	%
3 Pension subsidy	1	100.00

Total	1	100.00

- **ga007_11_s4 : Did this Household Member Receive this Type of Individual Income in Past Year**

No Observations

- **ga007_11_s5 : Did this Household Member Receive this Type of Individual Income in Past Year**

No Observations

- **ga007_11_s6 : Did this Household Member Receive this Type of Individual Income in Past Year**

No Observations

- **ga007_11_s7 : Did this Household Member Receive this Type of Individual Income in Past Year**

No Observations

- **ga007_11_s8 : Did this Household Member Receive this Type of Individual Income in Past Year**

No Observations

- **ga007_11_s9 : Did this Household Member Receive this Type of Individual Income in Past Year**

No Observations

- **ga007_11_s10 : Did this Household Member Receive this Type of Individual Income in Past Year**

	No	%
10 None of the above	32	100.00
Total	32	100.00

- **ga007_12_s1 : Did this Household Member Receive this Type of Individual Income in Past Year**

No Observations

- **ga007_12_s2 : Did this Household Member Receive this Type of Individual Income in Past Year**

No Observations

- **ga007_12_s3 : Did this Household Member Receive this Type of Individual Income in Past Year**

No Observations

- **ga007_12_s4 : Did this Household Member Receive this Type of Individual Income in Past Year**

No Observations

- **ga007_12_s5 : Did this Household Member Receive this Type of Individual Income in Past Year**

No Observations

- **ga007_12_s6 : Did this Household Member Receive this Type of Individual Income in Past Year**

No Observations

- **ga007_12_s7 : Did this Household Member Receive this Type of Individual Income in Past Year**

No Observations

- **ga007_12_s8 : Did this Household Member Receive this Type of Individual Income in Past Year**

No Observations

- **ga007_12_s9 : Did this Household Member Receive this Type of Individual Income in Past Year**

No Observations

- **ga007_12_s10 : Did this Household Member Receive this Type of Individual Income in Past Year**

	No	%
10 None of the above	19	100.00
Total	19	100.00

- **ga007_13_s1 : Did this Household Member Receive this Type of Individual Income in Past Year**

No Observations

- **ga007_13_s2 : Did this Household Member Receive this Type of Individual Income in Past Year**

No Observations

- **ga007_13_s3 : Did this Household Member Receive this Type of Individual Income in Past Year**

No Observations

- **ga007_13_s4 : Did this Household Member Receive this Type of Individual Income in Past Year**

No Observations

- **ga007_13_s5 : Did this Household Member Receive this Type of Individual Income in Past Year**

No Observations

- **ga007_13_s6 : Did this Household Member Receive this Type of Individual Income in Past Year**

No Observations

- **ga007_13_s7 : Did this Household Member Receive this Type of Individual Income in Past Year**

No Observations

- **ga007_13_s8 : Did this Household Member Receive this Type of Individual Income in Past Year**

No Observations

- **ga007_13_s9 : Did this Household Member Receive this Type of Individual Income in Past Year**

No Observations

- **ga007_13_s10 : Did this Household Member Receive this Type of Individual Income in Past Year**

	No	%
10 None of the above	10	100.00
Total	10	100.00

- **ga007_14_s1 : Did this Household Member Receive this Type of Individual Income in Past Year**

No Observations

- **ga007_14_s2 : Did this Household Member Receive this Type of Individual Income in Past Year**

No Observations

- **ga007_14_s3 : Did this Household Member Receive this Type of Individual Income in Past Year**

No Observations

- **ga007_14_s4 : Did this Household Member Receive this Type of Individual Income in Past Year**

No Observations

- **ga007_14_s5 : Did this Household Member Receive this Type of Individual Income in Past Year**

No Observations

- **ga007_14_s6 : Did this Household Member Receive this Type of Individual Income in Past Year**

No Observations

- **ga007_14_s7 : Did this Household Member Receive this Type of Individual Income in Past Year**

No Observations

- **ga007_14_s8 : Did this Household Member Receive this Type of Individual Income in Past Year**

No Observations

- **ga007_14_s9 : Did this Household Member Receive this Type of Individual Income in Past Year**

No Observations

- **ga007_14_s10 : Did this Household Member Receive this Type of Individual Income in Past Year**

	No	%
10 None of the above	5	100.00
Total	5	100.00

- **ga007_15_s1 : Did this Household Member Receive this Type of Individual Income in Past Year**

No Observations

- **ga007_15_s2 : Did this Household Member Receive this Type of Individual Income in Past Year**

No Observations

- **ga007_15_s3 : Did this Household Member Receive this Type of Individual Income in Past Year**

No Observations

- **ga007_15_s4 : Did this Household Member Receive this Type of Individual Income in Past Year**

No Observations

- **ga007_15_s5 : Did this Household Member Receive this Type of Individual Income in Past Year**

No Observations

- **ga007_15_s6 : Did this Household Member Receive this Type of Individual Income in Past Year**

No Observations

- **ga007_15_s7 : Did this Household Member Receive this Type of Individual Income in Past Year**

No Observations

- **ga007_15_s8 : Did this Household Member Receive this Type of Individual Income in Past Year**

No Observations

- **ga007_15_s9 : Did this Household Member Receive this Type of Individual Income in Past Year**

No Observations

- **ga007_15_s10 : Did this Household Member Receive this Type of Individual Income in Past Year**

	No	%
10 None of the above	2	100.00
Total	2	100.00

- **ga007_16_s1 : Did this Household Member Receive this Type of Individual Income in Past Year**

No Observations

- **ga007_16_s2 : Did this Household Member Receive this Type of Individual Income in Past Year**

No Observations

- **ga007_16_s3 : Did this Household Member Receive this Type of Individual Income in Past Year**

No Observations

- **ga007_16_s4 : Did this Household Member Receive this Type of Individual Income in Past Year**

No Observations

- **ga007_16_s5 : Did this Household Member Receive this Type of Individual Income in Past Year**

No Observations

- **ga007_16_s6 : Did this Household Member Receive this Type of Individual Income in Past Year**

No Observations

- **ga007_16_s7 : Did this Household Member Receive this Type of Individual Income in Past Year**

No Observations

- **ga007_16_s8 : Did this Household Member Receive this Type of Individual Income in Past Year**

No Observations

- **ga007_16_s9 : Did this Household Member Receive this Type of Individual Income in Past Year**

No Observations

- **ga007_16_s10 : Did this Household Member Receive this Type of Individual Income in Past Year**

	No	%
10 None of the above	1	100.00

Total	1	100.00

- **ga007_17_s1 : Did this Household Member Receive this Type of Individual Income in Past Year**

No Observations

- **ga007_17_s2 : Did this Household Member Receive this Type of Individual Income in Past Year**

No Observations

- **ga007_17_s3 : Did this Household Member Receive this Type of Individual Income in Past Year**

No Observations

- **ga007_17_s4 : Did this Household Member Receive this Type of Individual Income in Past Year**

No Observations

- **ga007_17_s5 : Did this Household Member Receive this Type of Individual Income in Past Year**

No Observations

- **ga007_17_s6 : Did this Household Member Receive this Type of Individual Income in Past Year**

No Observations

- **ga007_17_s7 : Did this Household Member Receive this Type of Individual Income in Past Year**

No Observations

- **ga007_17_s8 : Did this Household Member Receive this Type of Individual Income in Past Year**

No Observations

- **ga007_17_s9 : Did this Household Member Receive this Type of Individual Income in Past Year**

No Observations

- **ga007_17_s10 : Did this Household Member Receive this Type of Individual Income in Past Year**

	No	%
10 None of the above	1	100.00
Total	1	100.00

- **ga007_24_s1 : Did this Household Member Receive this Type of Individual Income in Past Year**

No Observations

- **ga007_24_s2 : Did this Household Member Receive this Type of Individual Income in Past Year**

No Observations

- **ga007_24_s3 : Did this Household Member Receive this Type of Individual Income in Past Year**

No Observations

- **ga007_24_s4 : Did this Household Member Receive this Type of Individual Income in Past Year**

No Observations

- **ga007_24_s5 : Did this Household Member Receive this Type of Individual Income in Past Year**

No Observations

- **ga007_24_s6 : Did this Household Member Receive this Type of Individual Income in Past Year**

No Observations

- **ga007_24_s7 : Did this Household Member Receive this Type of Individual Income in Past Year**

No Observations

- **ga007_24_s8 : Did this Household Member Receive this Type of Individual Income in Past Year**

No Observations

- **ga007_24_s9 : Did this Household Member Receive this Type of Individual Income in Past Year**

No Observations

- **ga007_24_s10 : Did this Household Member Receive this Type of Individual Income in Past Year**

	No	%
10 None of the above	1	100.00
Total	1	100.00

- **ga007_26_s1 : Did this Household Member Receive this Type of Individual Income in Past Year**

	No	%
1 Pensions	4	100.00
Total	4	100.00

- **ga007_26_s2 : Did this Household Member Receive this Type of Individual Income in Past Year**

No Observations

- **ga007_26_s3 : Did this Household Member Receive this Type of Individual Income in Past Year**

No Observations

- **ga007_26_s4 : Did this Household Member Receive this Type of Individual Income in Past Year**

No Observations

- **ga007_26_s5 : Did this Household Member Receive this Type of Individual Income in Past Year**

No Observations

- **ga007_26_s6 : Did this Household Member Receive this Type of Individ-**

ual Income in Past Year

	No	%
6 Medical aid	1	100.00
Total	1	100.00

- **ga007_26_s7 : Did this Household Member Receive this Type of Individual Income in Past Year**

No Observations

- **ga007_26_s8 : Did this Household Member Receive this Type of Individual Income in Past Year**

No Observations

- **ga007_26_s9 : Did this Household Member Receive this Type of Individual Income in Past Year**

No Observations

- **ga007_26_s10 : Did this Household Member Receive this Type of Individual Income in Past Year**

	No	%
10 None of the above	16	100.00
Total	16	100.00

- **ga008_1_a_1_ : Yuan/Year of Pension**

Mean	Min	Max	OBS
3,670.3	50.0	28,000.0	18

- **ga008_1_a_2_ : Yuan/Year of Pension**

Mean	Min	Max	OBS

6,053.3	200.0	25,000.0	6

- **ga008_1_a_3_ : Yuan/Year of Pension**

Mean	Min	Max	OBS
665.0	200.0	1,400.0	10

- **ga008_1_a_4_ : Yuan/Year of Pension**

Mean	Min	Max	OBS
6,640.0	720.0	18,000.0	3

- **ga008_1_a_5_ : Yuan/Year of Pension**

Mean	Min	Max	OBS
605.0	120.0	900.0	4

- **ga008_1_a_6_ : Yuan/Year of Pension**

Mean	Min	Max	OBS
720.0	720.0	720.0	1

- **ga008_1_a_7_ : Yuan/Year of Pension**

Mean	Min	Max	OBS
600.0	200.0	700.0	5

- **ga008_1_a_8_ : Yuan/Year of Pension**

No Observations

- **ga008_1_a_9_ : Yuan/Year of Pension**

No Observations

- **ga008_1_a_26_ : Yuan/Year of Pension**

No Observations

- **ga008_1_b_1_ : Yuan/Month of Pension**

Mean	Min	Max	OBS
657.2	50.0	6,000.0	73

- **ga008_1_b_2_ : Yuan/Month of Pension**

Mean	Min	Max	OBS
655.7	40.0	2,200.0	43

- **ga008_1_b_3_ : Yuan/Month of Pension**

Mean	Min	Max	OBS
638.3	0.0	4,000.0	62

- **ga008_1_b_4_ : Yuan/Month of Pension**

Mean	Min	Max	OBS
686.4	50.0	2,100.0	28

- **ga008_1_b_5_ : Yuan/Month of Pension**

Mean	Min	Max	OBS
821.7	60.0	2,000.0	6

- **ga008_1_b_6_ : Yuan/Month of Pension**

Mean	Min	Max	OBS
478.3	60.0	1,300.0	3

- **ga008_1_b_7_ : Yuan/Month of Pension**

Mean	Min	Max	OBS
1,700.0	1,700.0	1,700.0	1

- **ga008_1_b_8_ : Yuan/Month of Pension**

Mean	Min	Max	OBS
680.0	60.0	1,300.0	2

- **ga008_1_b_9_ : Yuan/Month of Pension**

Mean	Min	Max	OBS
1,300.0	1,300.0	1,300.0	1

- **ga008_1_b_26_ : Yuan/Month of Pension**

Mean	Min	Max	OBS
1,733.3	500.0	2,900.0	3

- **ga008_2_a_3_ : Yuan/Year of Unemployment Compensation**

Mean	Min	Max	OBS
1,000.0	1,000.0	1,000.0	1

- **ga008_2_a_4_ : Yuan/Year of Unemployment Compensation**

No Observations

- **ga008_2_b_3_ : Yuan/Month of Unemployment Compensation**

Mean	Min	Max	OBS
616.0	616.0	616.0	1

- **ga008_2_b_4_ : Yuan/Month of Unemployment Compensation**

Mean	Min	Max	OBS
600.0	600.0	600.0	1

- **ga008_3_a_1_ : Yuan/Year of Pension Subsidy**

Mean	Min	Max	OBS

1,390.0	480.0	2,300.0	2

- **ga008_3_a_2_ : Yuan/Year of Pension Subsidy**

Mean	Min	Max	OBS
862.5	725.0	1,000.0	2

- **ga008_3_a_3_ : Yuan/Year of Pension Subsidy**

Mean	Min	Max	OBS
792.5	100.0	1,800.0	10

- **ga008_3_a_4_ : Yuan/Year of Pension Subsidy**

No Observations

- **ga008_3_a_6_ : Yuan/Year of Pension Subsidy**

No Observations

- **ga008_3_a_7_ : Yuan/Year of Pension Subsidy**

No Observations

- **ga008_3_a_9_ : Yuan/Year of Pension Subsidy**

No Observations

- **ga008_3_a_11_ : Yuan/Year of Pension Subsidy**

Mean	Min	Max	OBS
480.0	480.0	480.0	1

- **ga008_3_b_1_ : Yuan/Month of Pension Subsidy**

	No	%
30	1	100.00
Total	1	100.00

- **ga008_3_b_2_ : Yuan/Month of Pension Subsidy**

	No	%
35	1	33.33
50	2	66.67
Total	3	100.00

- **ga008_3_b_3_ : Yuan/Month of Pension Subsidy**

Mean	Min	Max	OBS
87.5	30.0	120.0	4

- **ga008_3_b_4_ : Yuan/Month of Pension Subsidy**

Mean	Min	Max	OBS
140.0	40.0	240.0	2

- **ga008_3_b_6_ : Yuan/Month of Pension Subsidy**

Mean	Min	Max	OBS
133.3	40.0	200.0	3

- **ga008_3_b_7_ : Yuan/Month of Pension Subsidy**

	No	%
20	1	100.00
Total	1	100.00

- **ga008_3_b_9_ : Yuan/Month of Pension Subsidy**

	No	%
80	1	100.00
Total	1	100.00

- **ga008_3_b_11_ : Yuan/Month of Pension Subsidy**

No Observations

- **ga008_4_a_1_ : Yuan/Year of Worker's Compensation**

Mean	Min	Max	OBS
750.0	600.0	900.0	2

- **ga008_4_a_2_ : Yuan/Year of Worker's Compensation**

Mean	Min	Max	OBS
900.0	900.0	900.0	1

- **ga008_4_a_3_ : Yuan/Year of Worker's Compensation**

Mean	Min	Max	OBS
436.7	350.0	600.0	3

- **ga008_4_a_4_ : Yuan/Year of Worker's Compensation**

No Observations

- **ga008_4_a_6_ : Yuan/Year of Worker's Compensation**

No Observations

- **ga008_4_b_1_ : Yuan/Month of Worker's Compensation**

No Observations

- **ga008_4_b_2_ : Yuan/Month of Worker's Compensation**

No Observations

- **ga008_4_b_3_ : Yuan/Month of Worker's Compensation**

	No	%
80	2	100.00
Total	2	100.00

- **ga008_4_b_4_ : Yuan/Month of Worker's Compensation**

Mean	Min	Max	OBS
120.0	120.0	120.0	1

- **ga008_4_b_6_ : Yuan/Month of Worker's Compensation**

No Observations

- **ga008_5_a_1_ : Yuan/Year of Family Planning Subsidy**

Mean	Min	Max	OBS
90.0	60.0	120.0	2

- **ga008_5_a_2_ : Yuan/Year of Family Planning Subsidy**

Mean	Min	Max	OBS
945.0	60.0	3,000.0	4

- **ga008_5_a_3_ : Yuan/Year of Family Planning Subsidy**

Mean	Min	Max	OBS
192.0	60.0	600.0	5

- **ga008_5_a_4_ : Yuan/Year of Family Planning Subsidy**

Mean	Min	Max	OBS
273.3	20.0	600.0	3

- **ga008_5_a_5_ : Yuan/Year of Family Planning Subsidy**

Mean	Min	Max	OBS
876.7	50.0	2,400.0	3

- **ga008_5_a_6_ : Yuan/Year of Family Planning Subsidy**

Mean	Min	Max	OBS
296.7	50.0	720.0	3

- **ga008_5_a_7_ : Yuan/Year of Family Planning Subsidy**

Mean	Min	Max	OBS
420.0	420.0	420.0	1

- **ga008_5_b_1_ : Yuan/Month of Family Planning Subsidy**

	No	%
40	1	33.33
50	2	66.67
Total	3	100.00

- **ga008_5_b_2_ : Yuan/Month of Family Planning Subsidy**

	No	%
40	1	100.00
Total	1	100.00

- **ga008_5_b_3_ : Yuan/Month of Family Planning Subsidy**

	No	%
50	2	100.00
Total	2	100.00

- **ga008_5_b_4_ : Yuan/Month of Family Planning Subsidy**

	No	%
20	1	100.00
Total	1	100.00

- **ga008_5_b_5_ : Yuan/Month of Family Planning Subsidy**

	No	%
20	1	50.00
100	1	50.00
Total	2	100.00

- **ga008_5_b_6_ : Yuan/Month of Family Planning Subsidy**

No Observations

- **ga008_5_b_7_ : Yuan/Month of Family Planning Subsidy**

No Observations

- **ga008_6_a_1_ : Yuan/Year of Medical Aid**

	No	%
0	1	50.00
30	1	50.00
Total	2	100.00

- **ga008_6_a_2_ : Yuan/Year of Medical Aid**

Mean	Min	Max	OBS
650.0	300.0	1,000.0	2

- **ga008_6_a_3_ : Yuan/Year of Medical Aid**

Mean	Min	Max	OBS
1,332.9	30.0	5,000.0	7

- **ga008_6_a_4_ : Yuan/Year of Medical Aid**

Mean	Min	Max	OBS
500.0	500.0	500.0	2

- **ga008_6_a_5_ : Yuan/Year of Medical Aid**

No Observations

- **ga008_6_a_6_ : Yuan/Year of Medical Aid**

No Observations

- **ga008_6_a_7_ : Yuan/Year of Medical Aid**

No Observations

- **ga008_6_a_26_ : Yuan/Year of Medical Aid**

Mean	Min	Max	OBS
800.0	800.0	800.0	1

- **ga008_6_b_1_ : Yuan/Month of Medical Aid**

	No	%
20	1	25.00
30	1	25.00
40	1	25.00
73	1	25.00
Total	4	100.00

- **ga008_6_b_2_ : Yuan/Month of Medical Aid**

	No	%
0	1	33.33
40	1	33.33
100	1	33.33
Total	3	100.00

- **ga008_6_b_3_ : Yuan/Month of Medical Aid**

Mean	Min	Max	OBS

200.0	100.0	300.0	2

- **ga008_6_b_4_ : Yuan/Month of Medical Aid**

	No	%
70	1	100.00
Total	1	100.00

- **ga008_6_b_5_ : Yuan/Month of Medical Aid**

	No	%
30	1	100.00
Total	1	100.00

- **ga008_6_b_6_ : Yuan/Month of Medical Aid**

No Observations

- **ga008_6_b_7_ : Yuan/Month of Medical Aid**

	No	%
30	1	100.00
Total	1	100.00

- **ga008_6_b_26_ : Yuan/Month of Medical Aid**

No Observations

- **ga008_7_a_1_ : Yuan/Year of Other Government Subsidy**

Mean	Min	Max	OBS
519.3	0.0	2,000.0	15

- **ga008_7_a_2_ : Yuan/Year of Other Government Subsidy**

Mean	Min	Max	OBS
839.6	100.0	3,200.0	26

- **ga008_7_a_3_ : Yuan/Year of Other Government Subsidy**

Mean	Min	Max	OBS
919.3	0.0	5,000.0	67

- **ga008_7_a_4_ : Yuan/Year of Other Government Subsidy**

Mean	Min	Max	OBS
1,176.9	100.0	7,000.0	36

- **ga008_7_a_5_ : Yuan/Year of Other Government Subsidy**

Mean	Min	Max	OBS
902.0	100.0	4,700.0	22

- **ga008_7_a_6_ : Yuan/Year of Other Government Subsidy**

Mean	Min	Max	OBS
1,182.1	60.0	5,000.0	12

- **ga008_7_a_7_ : Yuan/Year of Other Government Subsidy**

Mean	Min	Max	OBS
430.0	360.0	500.0	2

- **ga008_7_b_1_ : Yuan/Month of Other Government Subsidy**

Mean	Min	Max	OBS
350.4	50.0	1,900.0	13

- **ga008_7_b_2_ : Yuan/Month of Other Government Subsidy**

Mean	Min	Max	OBS
113.7	20.0	300.0	12

- **ga008_7_b_3_ : Yuan/Month of Other Government Subsidy**

Mean	Min	Max	OBS
201.4	33.0	900.0	30

- **ga008_7_b_4_ : Yuan/Month of Other Government Subsidy**

Mean	Min	Max	OBS
197.5	30.0	500.0	8

- **ga008_7_b_5_ : Yuan/Month of Other Government Subsidy**

Mean	Min	Max	OBS
82.8	40.0	140.0	5

- **ga008_7_b_6_ : Yuan/Month of Other Government Subsidy**

Mean	Min	Max	OBS
153.5	30.0	300.0	4

- **ga008_7_b_7_ : Yuan/Month of Other Government Subsidy**

No Observations

- **ga008_8_a_1_ : Yuan/Year of Social Assistance**

No Observations

- **ga008_8_a_2_ : Yuan/Year of Social Assistance**

Mean	Min	Max	OBS
1,000.0	1,000.0	1,000.0	1

- **ga008_8_a_3_ : Yuan/Year of Social Assistance**

Mean	Min	Max	OBS
2,016.7	0.0	10,000.0	6

- **ga008_8_a_4_ : Yuan/Year of Social Assistance**

No Observations

- **ga008_8_a_5_ : Yuan/Year of Social Assistance**

	No	%
30	1	100.00
Total	1	100.00

- **ga008_8_a_6_ : Yuan/Year of Social Assistance**

No Observations

- **ga008_8_a_8_ : Yuan/Year of Social Assistance**

No Observations

- **ga008_8_b_1_ : Yuan/Month of Social Assistance**

No Observations

- **ga008_8_b_2_ : Yuan/Month of Social Assistance**

No Observations

- **ga008_8_b_3_ : Yuan/Month of Social Assistance**

No Observations

- **ga008_8_b_4_ : Yuan/Month of Social Assistance**

No Observations

- **ga008_8_b_5_ : Yuan/Month of Social Assistance**

No Observations

- **ga008_8_b_6_ : Yuan/Month of Social Assistance**

No Observations

- **ga008_8_b_8_ : Yuan/Month of Social Assistance**

No Observations

- **ga008_9_a_1_ : Yuan/Year of Other Income Source**

Mean	Min	Max	OBS
130.0	60.0	200.0	2

- **ga008_9_a_2_ : Yuan/Year of Other Income Source**

Mean	Min	Max	OBS
3,766.7	200.0	17,000.0	6

- **ga008_9_a_3_ : Yuan/Year of Other Income Source**

Mean	Min	Max	OBS
2,334.1	0.0	10,000.0	11

- **ga008_9_a_4_ : Yuan/Year of Other Income Source**

Mean	Min	Max	OBS
1,085.7	0.0	5,000.0	7

- **ga008_9_a_5_ : Yuan/Year of Other Income Source**

Mean	Min	Max	OBS
90.0	0.0	270.0	3

- **ga008_9_a_6_ : Yuan/Year of Other Income Source**

	No	%
0	1	100.00
Total	1	100.00

- **ga008_9_a_7_ : Yuan/Year of Other Income Source**

No Observations

- **ga008_9_b_1_ : Yuan/Month of Other Income Source**

Mean	Min	Max	OBS
175.0	50.0	300.0	2

- **ga008_9_b_2_ : Yuan/Month of Other Income Source**

Mean	Min	Max	OBS
306.7	200.0	420.0	3

- **ga008_9_b_3_ : Yuan/Month of Other Income Source**

Mean	Min	Max	OBS
281.3	0.0	600.0	8

- **ga008_9_b_4_ : Yuan/Month of Other Income Source**

Mean	Min	Max	OBS
290.0	0.0	420.0	4

- **ga008_9_b_5_ : Yuan/Month of Other Income Source**

Mean	Min	Max	OBS

500.0	0.0	2,000.0	4

- **ga008_9_b_6_ : Yuan/Month of Other Income Source**

	No	%
0	1	100.00
Total	1	100.00

- **ga008_9_b_7_ : Yuan/Month of Other Income Source**

No Observations

- **ga008_a_1_ : Min of Bracket**

Mean	Min	Max	OBS
1,111.2	0.0	10,001.0	18

- **ga008_a_2_ : Min of Bracket**

Mean	Min	Max	OBS
7,143.1	0.0	30,000.0	7

- **ga008_a_3_ : Min of Bracket**

Mean	Min	Max	OBS
23,333.4	0.0	200,000.0	9

- **ga008_a_4_ : Min of Bracket**

Mean	Min	Max	OBS
20,000.0	0.0	100,000.0	5

- **ga008_a_5_ : Min of Bracket**

	No	%
0	2	100.00
Total	2	100.00

- **ga008_a_8_ : Min of Bracket**

	No	%
0	1	100.00
Total	1	100.00

- **ga008_a_26_ : Min of Bracket**

Mean	Min	Max	OBS
76,666.7	0.0	200,000.0	3

- **ga008_b_1_ : Max of Bracket**

Mean	Min	Max	OBS
5,567,221.1	9,999.0	99,999,996.0	18

- **ga008_b_2_ : Max of Bracket**

Mean	Min	Max	OBS
18,570.6	9,999.0	30,000.0	7

- **ga008_b_3_ : Max of Bracket**

Mean	Min	Max	OBS
33,332.4	9,999.0	200,000.0	9

- **ga008_b_4_ : Max of Bracket**

Mean	Min	Max	OBS
20,025,998.6	9,999.0	99,999,996.0	5

- **ga008_b_5_ : Max of Bracket**

Mean	Min	Max	OBS
9,999.0	9,999.0	9,999.0	2

- **ga008_b_8_ : Max of Bracket**

Mean	Min	Max	OBS
9,999.0	9,999.0	9,999.0	1

- **ga008_b_26_ : Max of Bracket**

Mean	Min	Max	OBS
33,409,998.7	30,000.0	99,999,996.0	3

- **gb001 : Did Your Household Engage in Agricultural Work**

	No	%
1 Yes	5,915	59.05
2 No	4,102	40.95
Total	10,017	100.00

- **gb002_1_ : Did This Household Member Engage in Agricultural Work**

	No	%
1 Yes	325	66.87
2 No	161	33.13
Total	486	100.00

- **gb002_2_ : Did This Household Member Engage in Agricultural Work**

	No	%
1 Yes	602	55.43
2 No	484	44.57
Total	1,086	100.00

- **gb002_3_ : Did This Household Member Engage in Agricultural Work**

	No	%
1 Yes	1,196	31.71
2 No	2,576	68.29
Total	3,772	100.00

- **gb002_4_ : Did This Household Member Engage in Agricultural Work**

	No	%

1 Yes	730	25.29
2 No	2,157	74.71
Total	2,887	100.00

- **gb002_5_ : Did This Household Member Engage in Agricultural Work**

	No	%
1 Yes	240	12.37
2 No	1,700	87.63
Total	1,940	100.00

- **gb002_6_ : Did This Household Member Engage in Agricultural Work**

	No	%
1 Yes	109	10.72
2 No	908	89.28
Total	1,017	100.00

- **gb002_7_ : Did This Household Member Engage in Agricultural Work**

	No	%
1 Yes	59	14.60
2 No	345	85.40
Total	404	100.00

- **gb002_8_ : Did This Household Member Engage in Agricultural Work**

	No	%
1 Yes	26	13.00
2 No	174	87.00
Total	200	100.00

- **gb002_9_ : Did This Household Member Engage in Agricultural Work**

	No	%
1 Yes	17	14.91
2 No	97	85.09
Total	114	100.00

- **gb002_10_ : Did This Household Member Engage in Agricultural Work**

	No	%
1 Yes	6	10.34
2 No	52	89.66
Total	58	100.00

- **gb002_11_ : Did This Household Member Engage in Agricultural Work**

	No	%
1 Yes	2	6.90
2 No	27	93.10
Total	29	100.00

- **gb002_12_ : Did This Household Member Engage in Agricultural Work**

	No	%
1 Yes	1	6.25
2 No	15	93.75
Total	16	100.00

- **gb002_13_ : Did This Household Member Engage in Agricultural Work**

	No	%
2 No	10	100.00
Total	10	100.00

- **gb002_14_ : Did This Household Member Engage in Agricultural Work**

	No	%
2 No	6	100.00
Total	6	100.00

- **gb002_15_ : Did This Household Member Engage in Agricultural Work**

	No	%
2 No	2	100.00
Total	2	100.00

- **gb002_16_ : Did This Household Member Engage in Agricultural Work**

	No	%

2 No	1	100.00
Total	1	100.00

- **gb003 : Did Your Household Engage in Cropping or Forestry Last Year**

	No	%
1 Yes	3,950	66.42
2 No	1,997	33.58
Total	5,947	100.00

- **gb004_1 : Year**

Mean	Min	Max	OBS
2,010.7	2,009.0	2,011.0	3,867

- **gb004_2 : Month**

	No	%
0	58	1.50
1	22	0.57
2	22	0.57
3	46	1.19
4	159	4.11
5	674	17.43
6	795	20.56
7	578	14.95
8	555	14.35
9	469	12.13
10	388	10.03
11	75	1.94
12	26	0.67
Total	3,867	100.00

- **gb005 : Value of All Crops and Forestry Products**

Mean	Min	Max	OBS
6,650.3	0.0	100,000.0	3,837

- **gb005_a : Min of Bracket**

Mean	Min	Max	OBS
2,266.9	0.0	10,001.0	90

- **gb005_b : Max of Bracket**

Mean	Min	Max	OBS
18,891,554.4	999.0	99,999,996.0	90

- **gb006 : Total Cost of Producing Crops**

Mean	Min	Max	OBS
2,955.1	0.0	1,400,000.0	3,848

- **gb006_a : Min of Bracket**

Mean	Min	Max	OBS
1,152.9	0.0	5,001.0	78

- **gb006_b : Max of Bracket**

Mean	Min	Max	OBS
20,514,166.7	299.0	99,999,996.0	78

- **gb007 : Did Your Household Grow Any Livestock or Fish Last Year**

	No	%
1 Yes	2,162	36.54
2 No	3,755	63.46
Total	5,917	100.00

- **gb008 : Value of All Livestock**

Mean	Min	Max	OBS
10,676.2	0.0	700,000.0	2,124

- **gb008_a : Min of Bracket**

Mean	Min	Max	OBS
1,990.4	0.0	9,001.0	50

- **gb008_b : Max of Bracket**

Mean	Min	Max	OBS
34,001,558.2	499.0	99,999,996.0	50

- **gb009 : Value of All Livestock and Aquatic Life at the Same Time of Last Year**

Mean	Min	Max	OBS
9,787.3	-6,000.0	700,000.0	2,110

- **gb009_a : Min of Bracket**

Mean	Min	Max	OBS
2,219.0	0.0	8,001.0	64

- **gb009_b : Max of Bracket**

Mean	Min	Max	OBS
35,939,146.7	499.0	99,999,996.0	64

- **gb010 : How Much did You Spend Purchasing New Livestock and Qquatic Life Last Year**

Mean	Min	Max	OBS
3,157.0	0.0	1,000,000.0	2,127

- **gb011 : Value of Livestock and Qquatic Life Sold or Consumed Last Year**

Mean	Min	Max	OBS
7,642.5	0.0	2,000,000.0	2,099

- **gb011_a : Min of Bracket**

Mean	Min	Max	OBS
1,520.2	0.0	5,001.0	60

- **gb011_b : Max of Bracket**

Mean	Min	Max	OBS
40,001,003.2	199.0	99,999,996.0	60

- **gb012 : Value of All Livestock and Qquatic Life Produced**

Mean	Min	Max	OBS
934.9	0.0	170,000.0	2,101

- **gb012_a : Min of Bracket**

Mean	Min	Max	OBS
256.8	0.0	1,001.0	62

- **gb012_b : Max of Bracket**

Mean	Min	Max	OBS
33,871,199.9	99.0	99,999,996.0	62

- **gb013 : Cost of Producing Livestock and Qquatic Life Last Year**

Mean	Min	Max	OBS
4,368.5	0.0	1,000,000.0	2,063

- **gc001 : Engaged in Any Self-Employed Activities Last Year**

	No	%
1 Yes	845	8.44
2 No	9,170	91.56
Total	10,015	100.00

- **gc002 : Num. of Activities**

	No	%
0	25	2.85
1	803	91.46
2	48	5.47
3	2	0.23
Total	878	100.00

- **gc003_1_s1 : Who Engage in this Self-Employment Business in the Past Year**

	No	%
1 HMemberNameAll[1]	528	100.00
Total	528	100.00

- **gc003_1_s2 : Who Engage in this Self-Employment Business in the Past Year**

	No	%
2 HMemberNameAll[2]	416	100.00
Total	416	100.00

- **gc003_1_s3 : Who Engage in this Self-Employment Business in the Past Year**

	No	%
3 HMemberNameAll[3]	200	100.00
Total	200	100.00

- **gc003_1_s4 : Who Engage in this Self-Employment Business in the Past Year**

	No	%
4 HMemberNameAll[4]	115	100.00
Total	115	100.00

- **gc003_1_s5 : Who Engage in this Self-Employment Business in the Past Year**

	No	%
5 HMemberNameAll[5]	29	100.00
Total	29	100.00

- **gc003_1_s6 : Who Engage in this Self-Employment Business in the Past Year**

	No	%
6 HMemberNameAll[6]	9	100.00
Total	9	100.00

- **gc003_1_s7 : Who Engage in this Self-Employment Business in the Past Year**

	No	%
7 HMemberNameAll[7]	5	100.00
Total	5	100.00

- **gc003_1_s8 : Who Engage in this Self-Employment Business in the Past Year**

	No	%
8 HMemberNameAll[8]	2	100.00
Total	2	100.00

- **gc003_1_s9 : Who Engage in this Self-Employment Business in the Past Year**

	No	%
9 HMemberNameAll[9]	2	100.00
Total	2	100.00

- **gc003_1_s10 : Who Engage in this Self-Employment Business in the Past Year**

No Observations

- **gc003_1_s11 : Who Engage in this Self-Employment Business in the Past Year**

No Observations

- **gc003_1_s12 : Who Engage in this Self-Employment Business in the Past Year**

No Observations

- **gc003_1_s13 : Who Engage in this Self-Employment Business in the Past**

Year

No Observations

- **gc003_1_s14 : Who Engage in this Self-Employment Business in the Past Year**

No Observations

- **gc003_1_s15 : Who Engage in this Self-Employment Business in the Past Year**

No Observations

- **gc003_1_s16 : Who Engage in this Self-Employment Business in the Past Year**

No Observations

- **gc003_1_s17 : Who Engage in this Self-Employment Business in the Past Year**

No Observations

- **gc003_1_s18 : Who Engage in this Self-Employment Business in the Past Year**

No Observations

- **gc003_1_s19 : Who Engage in this Self-Employment Business in the Past Year**

No Observations

- **gc003_1_s20 : Who Engage in this Self-Employment Business in the Past Year**

No Observations

- **gc003_1_s21 : Who Engage in this Self-Employment Business in the Past Year**

No Observations

- **gc003_1_s22 : Who Engage in this Self-Employment Business in the Past Year**

No Observations

- **gc003_1_s23 : Who Engage in this Self-Employment Business in the Past Year**

No Observations

- **gc003_1_s24 : Who Engage in this Self-Employment Business in the Past Year**

No Observations

- **gc003_1_s25 : Who Engage in this Self-Employment Business in the Past Year**

No Observations

- **gc003_2_s1 : Who Engage in this Self-Employment Business in the Past Year**

	No	%
1 HMemberNameAll[1]	33	100.00
Total	33	100.00

- **gc003_2_s2 : Who Engage in this Self-Employment Business in the Past Year**

	No	%
2 HMemberNameAll[2]	28	100.00
Total	28	100.00

- **gc003_2_s3 : Who Engage in this Self-Employment Business in the Past Year**

	No	%
3 HMemberNameAll[3]	15	100.00
Total	15	100.00

- **gc003_2_s4 : Who Engage in this Self-Employment Business in the Past Year**

	No	%
4 HMemberNameAll[4]	9	100.00
Total	9	100.00

- **gc003_2_s5 : Who Engage in this Self-Employment Business in the Past Year**

	No	%
5 HMemberNameAll[5]	4	100.00
Total	4	100.00

- **gc003_2_s6 : Who Engage in this Self-Employment Business in the Past Year**

No Observations

- **gc003_2_s7 : Who Engage in this Self-Employment Business in the Past Year**

	No	%
7 HMemberNameAll[7]	2	100.00
Total	2	100.00

- **gc003_2_s8 : Who Engage in this Self-Employment Business in the Past Year**

No Observations

- **gc003_2_s9 : Who Engage in this Self-Employment Business in the Past Year**

	No	%
9 HMemberNameAll[9]	1	100.00
Total	1	100.00

- **gc003_2_s10 : Who Engage in this Self-Employment Business in the Past Year**

No Observations

- **gc003_2_s11 : Who Engage in this Self-Employment Business in the Past Year**

No Observations

- **gc003_2_s12 : Who Engage in this Self-Employment Business in the Past Year**

No Observations

- **gc003_2_s13 : Who Engage in this Self-Employment Business in the Past**

Year

No Observations

- **gc003_2_s14 : Who Engage in this Self-Employment Business in the Past Year**

No Observations

- **gc003_2_s15 : Who Engage in this Self-Employment Business in the Past Year**

No Observations

- **gc003_2_s16 : Who Engage in this Self-Employment Business in the Past Year**

No Observations

- **gc003_2_s17 : Who Engage in this Self-Employment Business in the Past Year**

No Observations

- **gc003_2_s18 : Who Engage in this Self-Employment Business in the Past Year**

No Observations

- **gc003_2_s19 : Who Engage in this Self-Employment Business in the Past Year**

No Observations

- **gc003_2_s20 : Who Engage in this Self-Employment Business in the Past Year**

No Observations

- **gc003_2_s21 : Who Engage in this Self-Employment Business in the Past Year**

No Observations

- **gc003_2_s22 : Who Engage in this Self-Employment Business in the Past Year**

No Observations

- **gc003_2_s23 : Who Engage in this Self-Employment Business in the Past Year**

No Observations

- **gc003_2_s24 : Who Engage in this Self-Employment Business in the Past Year**

No Observations

- **gc003_2_s25 : Who Engage in this Self-Employment Business in the Past Year**

No Observations

- **gc003_3_s1 : Who Engage in this Self-Employment Business in the Past Year**

	No	%
1 HMemberNameAll[1]	2	100.00
Total	2	100.00

- **gc003_3_s2 : Who Engage in this Self-Employment Business in the Past Year**

	No	%
2 HMemberNameAll[2]	1	100.00
Total	1	100.00

- **gc003_3_s3 : Who Engage in this Self-Employment Business in the Past Year**

No Observations

- **gc003_3_s4 : Who Engage in this Self-Employment Business in the Past Year**

No Observations

- **gc003_3_s5 : Who Engage in this Self-Employment Business in the Past Year**

No Observations

- **gc003_3_s6 : Who Engage in this Self-Employment Business in the Past Year**

No Observations

- **gc003_3_s7 : Who Engage in this Self-Employment Business in the Past**

Year

No Observations

- **gc003_3_s8 : Who Engage in this Self-Employment Business in the Past Year**

No Observations

- **gc003_3_s9 : Who Engage in this Self-Employment Business in the Past Year**

No Observations

- **gc003_3_s10 : Who Engage in this Self-Employment Business in the Past Year**

No Observations

- **gc003_3_s11 : Who Engage in this Self-Employment Business in the Past Year**

No Observations

- **gc003_3_s12 : Who Engage in this Self-Employment Business in the Past Year**

No Observations

- **gc003_3_s13 : Who Engage in this Self-Employment Business in the Past Year**

No Observations

- **gc003_3_s14 : Who Engage in this Self-Employment Business in the Past Year**

No Observations

- **gc003_3_s15 : Who Engage in this Self-Employment Business in the Past Year**

No Observations

- **gc003_3_s16 : Who Engage in this Self-Employment Business in the Past Year**

No Observations

- **gc003_3_s17 : Who Engage in this Self-Employment Business in the Past Year**

No Observations

- **gc003_3_s18 : Who Engage in this Self-Employment Business in the Past Year**

No Observations

- **gc003_3_s19 : Who Engage in this Self-Employment Business in the Past Year**

No Observations

- **gc003_3_s20 : Who Engage in this Self-Employment Business in the Past Year**

No Observations

- **gc003_3_s21 : Who Engage in this Self-Employment Business in the Past Year**

No Observations

- **gc003_3_s22 : Who Engage in this Self-Employment Business in the Past Year**

No Observations

- **gc003_3_s23 : Who Engage in this Self-Employment Business in the Past Year**

No Observations

- **gc003_3_s24 : Who Engage in this Self-Employment Business in the Past Year**

No Observations

- **gc003_3_s25 : Who Engage in this Self-Employment Business in the Past Year**

No Observations

- **gc004_1_s1 : Type of Activities**

	No	%

1 Services cooking, sewing, private clinic etc.	195	100.00
Total	195	100.00

- **gc004_1_s2 : Type of Activities**

	No	%
2 Transportation	77	100.00
Total	77	100.00

- **gc004_1_s3 : Type of Activities**

	No	%
3 Construction	32	100.00
Total	32	100.00

- **gc004_1_s4 : Type of Activities**

	No	%
4 Mining	1	100.00
Total	1	100.00

- **gc004_1_s5 : Type of Activities**

	No	%
5 Processing production	94	100.00
Total	94	100.00

- **gc004_1_s6 : Type of Activities**

	No	%
6 Business	364	100.00
Total	364	100.00

- **gc004_1_s7 : Type of Activities**

	No	%
7 Others	80	100.00
Total	80	100.00

- **gc004_2_s1 : Type of Activities**

	No	%
1 Services cooking, sewing, private clinic etc.	11	100.00
Total	11	100.00

- **gc004_2_s2 : Type of Activities**

	No	%
2 Transportation	2	100.00
Total	2	100.00

- **gc004_2_s3 : Type of Activities**

	No	%
3 Construction	4	100.00
Total	4	100.00

- **gc004_2_s4 : Type of Activities**

No Observations

- **gc004_2_s5 : Type of Activities**

	No	%
5 Processing production	9	100.00
Total	9	100.00

- **gc004_2_s6 : Type of Activities**

	No	%
6 Business	20	100.00
Total	20	100.00

- **gc004_2_s7 : Type of Activities**

	No	%
7 Others	6	100.00
Total	6	100.00

- **gc004_3_s1 : Type of Activities**

	No	%
1 Services cooking, sewing, private clinic etc.	1	100.00
Total	1	100.00

- **gc004_3_s2 : Type of Activities**

No Observations

- **gc004_3_s3 : Type of Activities**

No Observations

- **gc004_3_s4 : Type of Activities**

	No	%
4 Mining	1	100.00
Total	1	100.00

- **gc004_3_s5 : Type of Activities**

No Observations

- **gc004_3_s6 : Type of Activities**

No Observations

- **gc004_3_s7 : Type of Activities**

No Observations

- **gc005_1_ : Net Income from This Activity**

Mean	Min	Max	OBS
22,243.1	-30,000.0	600,000.0	762

- **gc005_2_ : Net Income from This Activity**

Mean	Min	Max	OBS
29,627.3	0.0	150,000.0	44

- **gc005_3_ : Net Income from This Activity**

Mean	Min	Max	OBS
25,000.0	0.0	50,000.0	2

- **gc005_a_1_ : Min of Bracket**

Mean	Min	Max	OBS
19,750.3	0.0	200,000.0	60

- **gc005_a_2_ : Min of Bracket**

Mean	Min	Max	OBS
14,000.0	0.0	50,000.0	5

- **gc005_b_1_ : Max of Bracket**

Mean	Min	Max	OBS
35,036,748.1	4,999.0	99,999,996.0	60

- **gc005_b_2_ : Max of Bracket**

Mean	Min	Max	OBS
40,013,998.4	10,000.0	99,999,996.0	5

- **gd001 : Did Your Household Receive Dibao Last Year**

	No	%
1 Yes	1,107	11.08

2 No	8,881	88.92
Total	9,988	100.00

- **gd001_c : Value of Dibao (Yuan)**

Mean	Min	Max	OBS
1,230.7	0.0	12,000.0	1,110

- **gd002_1 : Reforestation**

Mean	Min	Max	OBS
768.8	0.0	20,000.0	751

- **gd002_2 : Agricultural Subsidies**

Mean	Min	Max	OBS
432.9	-9,999.0	15,000.0	5,044

- **gd002_3 : Wubaohu**

Mean	Min	Max	OBS
1,460.8	0.0	5,160.0	100

- **gd002_4 : Tekunhu**

Mean	Min	Max	OBS
1,001.5	0.0	10,000.0	55

- **gd002_5 : Work Injury Subsidies**

Mean	Min	Max	OBS
2,416.4	40.0	10,000.0	14

- **gd002_6 : Emergency or Disaster Relief**

No Observations

- **gd002_7 : Others**

Mean	Min	Max	OBS
1,260.5	10.0	18,000.0	211

- **gd002s1 : Whether Receive This Subsidy**

	No	%
1 Reforestation	751	100.00
Total	751	100.00

- **gd002s2 : Whether Receive This Subsidy**

	No	%
2 Agricultural subsidies	5,051	100.00
Total	5,051	100.00

- **gd002s3 : Whether Receive This Subsidy**

	No	%
3 Wubaohu	98	100.00
Total	98	100.00

- **gd002s4 : Whether Receive This Subsidy**

	No	%
4 Tekunhu	56	100.00
Total	56	100.00

- **gd002s5 : Whether Receive This Subsidy**

	No	%
5 Work injury subsidies	14	100.00
Total	14	100.00

- **gd002s6 : Whether Receive This Subsidy**

	No	%
6 Emergency or disaster relief	14	100.00
Total	14	100.00

- **gd002s7 : Whether Receive This Subsidy**

	No	%
7 Other	210	100.00
Total	210	100.00

- **gd002s8 : Whether Receive This Subsidy**

	No	%
8 None	4,445	100.00
Total	4,445	100.00

- **gd003_1 : Donations from the Society**

Mean	Min	Max	OBS
970.6	10.0	20,000.0	49

- **gd003_2 : Compensation for Land Seizure**

Mean	Min	Max	OBS
14,731.8	0.0	600,000.0	240

- **gd003_3 : Compensation to Pulling Down Your House or Apartment**

Mean	Min	Max	OBS
41,840.9	46.0	330,000.0	39

- **gd003s1 : INCOME FROM THE FOLLOWING SOURCES**

	No	%
1 Donations from the society	49	100.00
Total	49	100.00

- **gd003s2 : INCOME FROM THE FOLLOWING SOURCES**

	No	%
2 Compensation for land seizure	241	100.00
Total	241	100.00

- **gd003s3 : INCOME FROM THE FOLLOWING SOURCES**

	No	%
3 Compensation to pulling down your house or apartment	38	100.00
Total	38	100.00

- **gd003s4 : INCOME FROM THE FOLLOWING SOURCES**

	No	%
4 None	9,676	100.00
Total	9,676	100.00

- **ge001 : You are the Primary Person Who Purchases Food for the Household**

	No	%
1 Yes	9,039	90.43
2 No	957	9.57
Total	9,996	100.00

- **ge002 : Who Purchasing Food**

	No	%
1 HMemberNameAll[1]	283	28.94
2 HMemberNameAll[2]	548	56.03
3 HMemberNameAll[3]	96	9.82
4 HMemberNameAll[4]	35	3.58
5 HMemberNameAll[5]	7	0.72
6 HMemberNameAll[6]	6	0.61
7 HMemberNameAll[7]	2	0.20
9 HMemberNameAll[9]	1	0.10
Total	978	100.00

- **ge003 : Who Answered Expenditure Questions**

	No	%
1 Primary person who purchases food for the household not Financial respondent	606	62.73
2 Financial respondent	287	29.71
3 Other	73	7.56
Total	966	100.00

- **ge004 : Num. of People Eating Together**

Mean	Min	Max	OBS
3.3	0.0	300.0	10,006

- **ge005 : Meals Provided to Guests**

Mean	Min	Max	OBS
1.5	0.0	750.0	9,971

- **ge006 : Expenditure on Food**

Mean	Min	Max	OBS
180.1	0.0	140,000.0	9,552

- **ge007 : Expenditure on Eating Out**

Mean	Min	Max	OBS
12.2	0.0	7,000.0	9,948

- **ge008 : Expenditure on Alcohol, Cigarettes, etc.**

Mean	Min	Max	OBS
34.0	0.0	9,000.0	9,825

- **ge009_1 : Communication Fees**

Mean	Min	Max	OBS
-76.4	-9,999.0	9,999.0	9,929

- **ge009_2 : Utilities Fees**

Mean	Min	Max	OBS
-0.3	-9,999.0	9,999.0	9,929

- **ge009_3 : Fuels Fees**

Mean	Min	Max	OBS
-15.4	-9,999.0	9,999.0	9,929

- **ge009_4 : Fees on Matron, Housekeepers and Servants**

Mean	Min	Max	OBS
-18.8	-9,999.0	9,999.0	9,929

- **ge009_5 : Local Transportaion Fees**

Mean	Min	Max	OBS
-49.8	-9,999.0	9,999.0	9,929

- **ge009_6 : Fees on Household Items**

Mean	Min	Max	OBS
-199.6	-9,999.0	9,999.0	9,929

- **ge009_7 : Entertainment Fees**

Mean	Min	Max	OBS
-37.5	-9,999.0	9,999.0	9,929

- **ge010_1 : Clothing and Bedding**

Mean	Min	Max	OBS
732.7	-99,990.0	100,000.0	9,883

- **ge010_2 : Long Distance Travelling Expenses**

Mean	Min	Max	OBS
276.8	-9,999.0	150,000.0	9,883

- **ge010_3 : Heating**

Mean	Min	Max	OBS
225.6	-9,999.0	25,000.0	9,883

- **ge010_4 : Furniture and Consumption of Durable Goods**

Mean	Min	Max	OBS

498.3	-9,999.0	230,000.0	9,883

- **ge010_5 : Education and Training**

Mean	Min	Max	OBS
49.2	-9,999.0	50,000.0	9,883

- **ge010_6 : Medical Expenditure**

Mean	Min	Max	OBS
-7.9	-9,999.0	20,000.0	9,883

- **ge010_7 : Fitness Expenditure**

Mean	Min	Max	OBS
322.1	-9,999.0	50,000.0	9,883

- **ge010_8 : Beauty**

Mean	Min	Max	OBS
62.4	-9,999.0	200,000.0	9,883

- **ge010_9 : Purchase, MAintenance and Repair**

Mean	Min	Max	OBS
1,557.2	-9,999.0	200,000.0	9,883

- **ge010_10 : Taxes and Fees Turned Over to the Government**

Mean	Min	Max	OBS
2,885.3	-9,999.0	200,000.0	9,883

- **ge010_11 : Automobiles**

Mean	Min	Max	OBS
1,204.3	-9,999.0	800,000.0	9,883

- **ge010_12 : Electronics**

Mean	Min	Max	OBS
139.9	-9,999.0	60,000.0	9,883

- **ge010_13 : Property Management Fees**

Mean	Min	Max	OBS
3.7	-9,999.0	15,000.0	9,883

- **ge010_14 : Donations to the Society**

Mean	Min	Max	OBS
-23.7	-9,999.0	15,000.0	9,883

- **ha001 : When did Your Household Start to Live at Your Current Residence**

Mean	Min	Max	OBS
1,993.2	1,900.0	2,011.0	9,735

- **ha002 : Do You Pay Rent for Your Current Residence**

	No	%
1 Yes	301	3.01
2 No	9,706	96.99
Total	10,007	100.00

- **ha003 : Rent Per Month**

Mean	Min	Max	OBS
348.7	0.0	3,500.0	309

- **ha004 : Pay Less than Market Rental Value**

	No	%
1 Yes	126	42.28
2 No	172	57.72
Total	298	100.00

- **ha005 : Market Rental Value**

Mean	Min	Max	OBS
693.3	50.0	4,500.0	124

- **ha006_1 : Subsidy Amount**

Mean	Min	Max	OBS
29.9	0.0	490.0	106

- **ha006_2 : Which Household Member**

	No	%
1 HMemberNameAll[1]	85	82.52
2 HMemberNameAll[2]	18	17.48
Total	103	100.00

- **ha007 : Ownship of Current Residence**

	No	%
1 Totally Belongs to Household Member	8,541	85.40
2 Partially Belongs to Household Member	426	4.26
3 Not Belongs to Household Member	1,034	10.34
Total	10,001	100.00

- **ha008s1 : Which Household Member(s)) Own the House**

	No	%
1 HMemberNameAll[1]	7,644	100.00
Total	7,644	100.00

- **ha008s2 : Which Household Member(s)) Own the House**

	No	%
2 HMemberNameAll[2]	4,742	100.00
Total	4,742	100.00

- **ha008s3 : Which Household Member(s)) Own the House**

	No	%

3 HMemberNameAll[3]	1,489	100.00
Total	1,489	100.00

- **ha008s4 : Which Household Member(s)) Own the House**

	No	%
4 HMemberNameAll[4]	854	100.00
Total	854	100.00

- **ha008s5 : Which Household Member(s)) Own the House**

	No	%
5 HMemberNameAll[5]	452	100.00
Total	452	100.00

- **ha008s6 : Which Household Member(s)) Own the House**

	No	%
6 HMemberNameAll[6]	194	100.00
Total	194	100.00

- **ha008s7 : Which Household Member(s)) Own the House**

	No	%
7 HMemberNameAll[7]	71	100.00
Total	71	100.00

- **ha008s8 : Which Household Member(s)) Own the House**

	No	%
8 HMemberNameAll[8]	36	100.00
Total	36	100.00

- **ha008s9 : Which Household Member(s)) Own the House**

	No	%
9 HMemberNameAll[9]	15	100.00
Total	15	100.00

- **ha008s10 : Which Household Member(s)) Own the House**

	No	%
10 HMemberNameAll[10]	5	100.00
Total	5	100.00

- **ha008s11 : Which Household Member(s)) Own the House**

	No	%
11 HMemberNameAll[11]	2	100.00
Total	2	100.00

- **ha008s12 : Which Household Member(s)) Own the House**

	No	%
12 HMemberNameAll[12]	1	100.00
Total	1	100.00

- **ha008s13 : Which Household Member(s)) Own the House**

	No	%
13 HMemberNameAll[13]	2	100.00
Total	2	100.00

- **ha008s14 : Which Household Member(s)) Own the House**

	No	%
14 HMemberNameAll[14]	2	100.00
Total	2	100.00

- **ha008s15 : Which Household Member(s)) Own the House**

No Observations

- **ha008s16 : Which Household Member(s)) Own the House**

No Observations

- **ha008s17 : Which Household Member(s)) Own the House**

No Observations

- **ha008s18 : Which Household Member(s)) Own the House**

No Observations

- **ha008s19 : Which Household Member(s)) Own the House**

No Observations

- **ha008s20 : Which Household Member(s)) Own the House**

No Observations

- **ha008s21 : Which Household Member(s)) Own the House**

No Observations

- **ha008s22 : Which Household Member(s)) Own the House**

No Observations

- **ha008s23 : Which Household Member(s)) Own the House**

No Observations

- **ha008s24 : Which Household Member(s)) Own the House**

No Observations

- **ha008s25 : Which Household Member(s)) Own the House**

No Observations

- **ha009_1_ : Percentage Owned by this Household Member**

Mean	Min	Max	OBS
68.1	0.0	100.0	7,572

- **ha009_2_ : Percentage Owned by this Household Member**

Mean	Min	Max	OBS
51.3	0.0	100.0	4,698

- **ha009_3_ : Percentage Owned by this Household Member**

Mean	Min	Max	OBS
45.3	0.0	100.0	1,463

- **ha009_4_ : Percentage Owned by this Household Member**

Mean	Min	Max	OBS
36.4	0.0	100.0	841

- **ha009_5_ : Percentage Owned by this Household Member**

Mean	Min	Max	OBS
34.6	0.0	100.0	437

- **ha009_6_ : Percentage Owned by this Household Member**

Mean	Min	Max	OBS
30.3	0.0	100.0	183

- **ha009_7_ : Percentage Owned by this Household Member**

Mean	Min	Max	OBS
22.4	0.0	100.0	64

- **ha009_8_ : Percentage Owned by this Household Member**

Mean	Min	Max	OBS
31.0	0.0	100.0	34

- **ha009_9_ : Percentage Owned by this Household Member**

Mean	Min	Max	OBS
19.7	10.0	50.0	15

- **ha009_10_ : Percentage Owned by this Household Member**

	No	%
10	2	40.00
16	2	40.00
25	1	20.00
Total	5	100.00

- **ha009_11_ : Percentage Owned by this Household Member**

	No	%
16	1	50.00
33	1	50.00
Total	2	100.00

- **ha009_12_ : Percentage Owned by this Household Member**

	No	%
20	1	100.00
Total	1	100.00

- **ha009_13_ : Percentage Owned by this Household Member**

	No	%
16	1	50.00
20	1	50.00

Total	2	100.00

- **ha009_14_ : Percentage Owned by this Household Member**

	No	%
20	1	50.00
25	1	50.00
Total	2	100.00

- **ha010_1 : Which Household Member**

	No	%
1 HMemberNameAll[1]	28	68.29
2 HMemberNameAll[2]	9	21.95
3 HMemberNameAll[3]	3	7.32
5 HMemberNameAll[5]	1	2.44
Total	41	100.00

- **ha010_2 : Which Child**

	No	%
1 FLSepChild[1]	190	35.45
2 FLSepChild[2]	163	30.41
3 FLSepChild[3]	99	18.47
4 FLSepChild[4]	44	8.21
5 FLSepChild[5]	21	3.92
6 FLSepChild[6]	11	2.05
7 FLSepChild[7]	5	0.93
8 FLSepChild[8]	3	0.56
Total	536	100.00

- **ha010_3 : Which Parent**

	No	%
1 FLSepLiveParent[1]	2	25.00
2 FLSepLiveParent[2]	2	25.00
3 FLSepLiveParent[3]	3	37.50
4 FLSepLiveParent[4]	1	12.50
Total	8	100.00

- **ha010s1 : Who Owns Your Current Residence**

	No	%

1 Working unit of household member	36	100.00
Total	36	100.00

- **ha010s2 : Who Owns Your Current Residence**

	No	%
2 Government indemnificatory housing	61	100.00
Total	61	100.00

- **ha010s3 : Who Owns Your Current Residence**

	No	%
3 Child (non-household member)	542	100.00
Total	542	100.00

- **ha010s4 : Who Owns Your Current Residence**

	No	%
4 Parent (non-household member)	13	100.00
Total	13	100.00

- **ha010s5 : Who Owns Your Current Residence**

	No	%
5 Nonresident other relatives	104	100.00
Total	104	100.00

- **ha010s6 : Who Owns Your Current Residence**

	No	%
6 Friends	33	100.00
Total	33	100.00

- **ha010s7 : Who Owns Your Current Residence**

	No	%
7 Other	258	100.00
Total	258	100.00

- **ha011_1 : Total Price**

Mean	Min	Max	OBS
1,547.0	-9,999.0	800,000.0	8,276

- **ha011_2 : Unit Price**

Mean	Min	Max	OBS
466.4	0.0	30,000.0	494

- **ha011_check : Are You Sure**

	No	%
1 Yes	185	98.40
2 No	3	1.60
Total	188	100.00

- **ha012_a : Min of Bracket**

Mean	Min	Max	OBS
63,525.6	0.0	500,001.0	1,302

- **ha012_b : Max of Bracket**

Mean	Min	Max	OBS
33,730,213.3	19,999.0	99,999,996.0	1,302

- **ha013 : Take Out a Bank Loan to Finance Your House**

	No	%
1 Yes	232	2.72
2 No	8,300	97.28
Total	8,532	100.00

- **ha014 : Amount of Loans**

Mean	Min	Max	OBS
50,018.8	0.0	600,000.0	256

- **ha015 : Monthly Mortgage Payment**

Mean	Min	Max	OBS
1,069.1	0.0	10,000.0	210

- **ha016 : How was this Housing Unit Obtained**

	No	%
1 Purchased from market	855	10.01
2 Purchased from working unit of household members	508	5.95
3 Purchased from Child	8	0.09
4 Purchased from Parents	12	0.14
5 Purchased from Other relatives	70	0.82
6 Self-built	6,390	74.82
7 Inherited, bequeathed, or given	356	4.17
8 Received home as compensation for demolition of old home	194	2.27
9 Other	148	1.73
Total	8,541	100.00

- **ha016_1s1 : Which Household Member**

	No	%
1 HMemberNameAll[1]	549	100.00
Total	549	100.00

- **ha016_1s2 : Which Household Member**

	No	%
2 HMemberNameAll[2]	173	100.00
Total	173	100.00

- **ha016_1s3 : Which Household Member**

	No	%
3 HMemberNameAll[3]	33	100.00
Total	33	100.00

- **ha016_1s4 : Which Household Member**

	No	%
4 HMemberNameAll[4]	16	100.00
Total	16	100.00

- **ha016_1s5 : Which Household Member**

	No	%
5 HMemberNameAll[5]	5	100.00
Total	5	100.00

- **ha016_1s6 : Which Household Member**

	No	%
6 HMemberNameAll[6]	1	100.00
Total	1	100.00

- **ha016_1s7 : Which Household Member**

No Observations

- **ha016_1s8 : Which Household Member**

No Observations

- **ha016_1s9 : Which Household Member**

No Observations

- **ha016_1s10 : Which Household Member**

No Observations

- **ha016_1s11 : Which Household Member**

No Observations

- **ha016_1s12 : Which Household Member**

No Observations

- **ha016_1s13 : Which Household Member**

No Observations

- **ha016_1s14 : Which Household Member**

No Observations

- **ha016_1s15 : Which Household Member**

No Observations

- **ha016_1s16 : Which Household Member**

No Observations

- **ha016_1s17 : Which Household Member**

No Observations

- **ha016_1s18 : Which Household Member**

No Observations

- **ha016_1s19 : Which Household Member**

No Observations

- **ha016_1s20 : Which Household Member**

No Observations

- **ha016_1s21 : Which Household Member**

No Observations

- **ha016_1s22 : Which Household Member**

No Observations

- **ha016_1s23 : Which Household Member**

No Observations

- **ha016_1s24 : Which Household Member**

No Observations

- **ha016_1s25 : Which Household Member**

No Observations

- **ha016_2 : Separated Child**

	No	%
1 FLSepChild[1]	4	36.36
2 FLSepChild[2]	1	9.09
3 FLSepChild[3]	3	27.27
4 FLSepChild[4]	2	18.18
5 FLSepChild[5]	1	9.09
Total	11	100.00

- **ha016_3 : R or Spouse**

	No	%
1 FLRespondents[1]	15	71.43
2 FLRespondents[2]	6	28.57
Total	21	100.00

- **ha017 : When did You Purchase It**

Mean	Min	Max	OBS
1,998.3	1,900.0	2,011.0	1,405

- **ha018 : Your Own Money Pay for the House**

Mean	Min	Max	OBS
2,877.6	0.0	600,000.0	1,397

- **ha019 : Price Type**

	No	%
1 Market price	823	56.64
2 Subsidized by working unit	519	35.72
3 Economic housing	35	2.41
4 Other	76	5.23
Total	1,453	100.00

- **ha020_1 : Total Price**

Mean	Min	Max	OBS
1,947.1	-9,999.0	300,000.0	746

- **ha020_2 : Unit Price**

Mean	Min	Max	OBS
466.3	0.0	9,000.0	144

- **ha020_check : Are You Sure**

	No	%
1 Yes	16	84.21

2 No	3	15.79
Total	19	100.00

- **ha021 : When did You Receive the House**

Mean	Min	Max	OBS
2,002.5	1,974.0	2,011.0	195

- **ha022 : Market Value at That Time**

Mean	Min	Max	OBS
614.6	0.1	100,000.0	165

- **ha022_check : Are You Sure**

	No	%
1 Yes	1	100.00
Total	1	100.00

- **ha023_1 : Total Price**

Mean	Min	Max	OBS
946.0	0.0	53,000.0	111

- **ha023_2 : Unit Price**

Mean	Min	Max	OBS
256.7	0.1	5,000.0	59

- **ha023_check : Are You Sure**

	No	%
1 Yes	2	100.00
Total	2	100.00

- **ha024 : Can You Sell the House Freely**

	No	%
1 Yes	166	86.91

2 No, restricted by work unit	25	13.09
Total	191	100.00

- **ha025 : Spending on Decorating or Renovating**

Mean	Min	Max	OBS
12,900.4	-9,999.0	800,000.0	7,974

- **ha026 : Year**

Mean	Min	Max	OBS
1,996.7	1,900.0	2,011.0	7,526

- **ha027 : Other Residential Properties**

	No	%
1 Yes	996	9.96
2 No	9,004	90.04
Total	10,000	100.00

- **ha028 : Num. Of other Housing Units**

	No	%
0	65	6.05
1	945	87.99
2	56	5.21
3	6	0.56
4	1	0.09
6	1	0.09
Total	1,074	100.00

- **ha029_1_1_ : Province**

	No	%
01	37	3.81
03	60	6.19
04	1	0.10
05	82	8.45
06	45	4.64
07	46	4.74
08	1	0.10
09	5	0.52
10	49	5.05

11	45	4.64
12	10	1.03
13	16	1.65
14	20	2.06
15	77	7.94
16	22	2.27
17	26	2.68
18	14	1.44
19	10	1.03
20	58	5.98
21	12	1.24
24	17	1.75
26	53	5.46
27	16	1.65
28	19	1.96
29	101	10.41
32	69	7.11
33	50	5.15
34	9	0.93
Total	970	100.00

- **ha029_1_2_ : Province**

	No	%
01	1	1.56
06	2	3.13
07	4	6.25
09	1	1.56
10	2	3.13
13	1	1.56
14	2	3.13
15	13	20.31
16	2	3.13
17	4	6.25
18	1	1.56
20	6	9.38
21	1	1.56
24	2	3.13
26	4	6.25
28	1	1.56
29	9	14.06
32	5	7.81
33	2	3.13
34	1	1.56
Total	64	100.00

- **ha029_1_3_ : Province**

	No	%

15	4	44.44
17	1	11.11
20	1	11.11
32	2	22.22
33	1	11.11
Total	9	100.00

- **ha029_1_4_ : Province**

	No	%
17	1	100.00
Total	1	100.00

- **ha029_2_1_ : City**

	No	%
01	47	4.85
02	5	0.52
04	64	6.60
11	62	6.40
16	40	4.13
17	10	1.03
18	3	0.31
24	37	3.82
27	35	3.61
38	7	0.72
40	178	18.37
42	2	0.21
46	66	6.81
49	19	1.96
51	3	0.31
52	6	0.62
53	43	4.44
55	41	4.23
56	16	1.65
60	52	5.37
63	33	3.41
66	12	1.24
67	1	0.10
74	49	5.06
76	2	0.21
77	3	0.31
82	114	11.76
86	17	1.75
96	1	0.10
99	1	0.10
Total	969	100.00

- **ha029_2_2_ : City**

	No	%
04	3	4.76
11	2	3.17
16	3	4.76
24	6	9.52
27	3	4.76
38	1	1.59
40	11	17.46
46	5	7.94
49	1	1.59
52	1	1.59
53	6	9.52
55	4	6.35
56	3	4.76
60	2	3.17
63	1	1.59
74	3	4.76
82	8	12.70
Total	63	100.00

- **ha029_2_3_ : City**

	No	%
40	3	33.33
46	3	33.33
53	1	11.11
56	1	11.11
82	1	11.11
Total	9	100.00

- **ha029_2_4_ : City**

	No	%
46	1	100.00
Total	1	100.00

- **ha030_1_ : Who Owns this Residence**

	No	%
1 Owned completely by your household members.	904	92.24
2 Owned partly by your household members.	76	7.76
Total	980	100.00

- **ha030_2_ : Who Owns this Residence**

	No	%
1 Owned completely by your household members.	60	95.24
2 Owned partly by your household members.	3	4.76
Total	63	100.00

- **ha030_3_ : Who Owns this Residence**

	No	%
1 Owned completely by your household members.	8	88.89
2 Owned partly by your household members.	1	11.11
Total	9	100.00

- **ha030_4_ : Who Owns this Residence**

	No	%
1 Owned completely by your household members.	1	100.00
Total	1	100.00

- **ha031_1_s1 : Which Household Member**

	No	%
1 HMemberNameAll[1]	721	100.00
Total	721	100.00

- **ha031_1_s2 : Which Household Member**

	No	%
2 HMemberNameAll[2]	466	100.00
Total	466	100.00

- **ha031_1_s3 : Which Household Member**

	No	%
3 HMemberNameAll[3]	170	100.00
Total	170	100.00

- **ha031_1_s4 : Which Household Member**

	No	%

4 HMemberNameAll[4]	98	100.00
Total	98	100.00

- **ha031_1_s5 : Which Household Member**

	No	%
5 HMemberNameAll[5]	48	100.00
Total	48	100.00

- **ha031_1_s6 : Which Household Member**

	No	%
6 HMemberNameAll[6]	21	100.00
Total	21	100.00

- **ha031_1_s7 : Which Household Member**

	No	%
7 HMemberNameAll[7]	7	100.00
Total	7	100.00

- **ha031_1_s8 : Which Household Member**

	No	%
8 HMemberNameAll[8]	4	100.00
Total	4	100.00

- **ha031_1_s9 : Which Household Member**

	No	%
9 HMemberNameAll[9]	3	100.00
Total	3	100.00

- **ha031_1_s10 : Which Household Member**

	No	%
10 HMemberNameAll[10]	2	100.00
Total	2	100.00

- **ha031_1_s11 : Which Household Member**

	No	%
11 HMemberNameAll[11]	1	100.00
Total	1	100.00

- **ha031_1_s12 : Which Household Member**

No Observations

- **ha031_1_s13 : Which Household Member**

No Observations

- **ha031_1_s14 : Which Household Member**

No Observations

- **ha031_1_s15 : Which Household Member**

No Observations

- **ha031_1_s16 : Which Household Member**

No Observations

- **ha031_1_s17 : Which Household Member**

No Observations

- **ha031_1_s18 : Which Household Member**

No Observations

- **ha031_1_s19 : Which Household Member**

No Observations

- **ha031_1_s20 : Which Household Member**

No Observations

- **ha031_1_s21 : Which Household Member**

No Observations

- **ha031_1_s22 : Which Household Member**

No Observations

- **ha031_1_s23 : Which Household Member**

No Observations

- **ha031_1_s24 : Which Household Member**

No Observations

- **ha031_1_s25 : Which Household Member**

No Observations

- **ha031_2_s1 : Which Household Member**

	No	%
1 HMemberNameAll[1]	41	100.00
Total	41	100.00

- **ha031_2_s2 : Which Household Member**

	No	%
2 HMemberNameAll[2]	26	100.00
Total	26	100.00

- **ha031_2_s3 : Which Household Member**

	No	%
3 HMemberNameAll[3]	15	100.00
Total	15	100.00

- **ha031_2_s4 : Which Household Member**

	No	%
4 HMemberNameAll[4]	9	100.00
Total	9	100.00

- **ha031_2_s5 : Which Household Member**

	No	%
5 HMemberNameAll[5]	9	100.00
Total	9	100.00

- **ha031_2_s6 : Which Household Member**

	No	%
6 HMemberNameAll[6]	6	100.00
Total	6	100.00

- **ha031_2_s7 : Which Household Member**

	No	%
7 HMemberNameAll[7]	3	100.00

Total	3	100.00

- **ha031_2_s8 : Which Household Member**

No Observations

- **ha031_2_s9 : Which Household Member**

No Observations

- **ha031_2_s10 : Which Household Member**

No Observations

- **ha031_2_s11 : Which Household Member**

No Observations

- **ha031_2_s12 : Which Household Member**

No Observations

- **ha031_2_s13 : Which Household Member**

No Observations

- **ha031_2_s14 : Which Household Member**

No Observations

- **ha031_2_s15 : Which Household Member**

No Observations

- **ha031_2_s16 : Which Household Member**

No Observations

- **ha031_2_s17 : Which Household Member**

No Observations

- **ha031_2_s18 : Which Household Member**

No Observations

- **ha031_2_s19 : Which Household Member**

No Observations

- **ha031_2_s20 : Which Household Member**

No Observations

- **ha031_2_s21 : Which Household Member**

No Observations

- **ha031_2_s22 : Which Household Member**

No Observations

- **ha031_2_s23 : Which Household Member**

No Observations

- **ha031_2_s24 : Which Household Member**

No Observations

- **ha031_2_s25 : Which Household Member**

No Observations

- **ha031_3_s1 : Which Household Member**

	No	%
1 HMemberNameAll[1]	4	100.00
Total	4	100.00

- **ha031_3_s2 : Which Household Member**

	No	%
2 HMemberNameAll[2]	5	100.00
Total	5	100.00

- **ha031_3_s3 : Which Household Member**

	No	%
3 HMemberNameAll[3]	2	100.00
Total	2	100.00

- **ha031_3_s4 : Which Household Member**

No Observations

- **ha031_3_s5 : Which Household Member**

	No	%
5 HMemberNameAll[5]	1	100.00
Total	1	100.00

- **ha031_3_s6 : Which Household Member**

No Observations

- **ha031_3_s7 : Which Household Member**

	No	%
7 HMemberNameAll[7]	1	100.00
Total	1	100.00

- **ha031_3_s8 : Which Household Member**

No Observations

- **ha031_3_s9 : Which Household Member**

No Observations

- **ha031_3_s10 : Which Household Member**

No Observations

- **ha031_3_s11 : Which Household Member**

No Observations

- **ha031_3_s12 : Which Household Member**

No Observations

- **ha031_3_s13 : Which Household Member**

No Observations

- **ha031_3_s14 : Which Household Member**

No Observations

- **ha031_3_s15 : Which Household Member**

No Observations

- **ha031_3_s16 : Which Household Member**

No Observations

- **ha031_3_s17 : Which Household Member**

No Observations

- **ha031_3_s18 : Which Household Member**

No Observations

- **ha031_3_s19 : Which Household Member**

No Observations

- **ha031_3_s20 : Which Household Member**

No Observations

- **ha031_3_s21 : Which Household Member**

No Observations

- **ha031_3_s22 : Which Household Member**

No Observations

- **ha031_3_s23 : Which Household Member**

No Observations

- **ha031_3_s24 : Which Household Member**

No Observations

- **ha031_3_s25 : Which Household Member**

No Observations

- **ha031_4_s1 : Which Household Member**

No Observations

- **ha031_4_s2 : Which Household Member**

No Observations

- **ha031_4_s3 : Which Household Member**

No Observations

- **ha031_4_s4 : Which Household Member**

No Observations

- **ha031_4_s5 : Which Household Member**

	No	%
5 HMemberNameAll[5]	1	100.00
Total	1	100.00

- **ha031_4_s6 : Which Household Member**

No Observations

- **ha031_4_s7 : Which Household Member**

No Observations

- **ha031_4_s8 : Which Household Member**

No Observations

- **ha031_4_s9 : Which Household Member**

No Observations

- **ha031_4_s10 : Which Household Member**

No Observations

- **ha031_4_s11 : Which Household Member**

No Observations

- **ha031_4_s12 : Which Household Member**

No Observations

- **ha031_4_s13 : Which Household Member**

No Observations

- **ha031_4_s14 : Which Household Member**

No Observations

- **ha031_4_s15 : Which Household Member**

No Observations

- **ha031_4_s16 : Which Household Member**

No Observations

- **ha031_4_s17 : Which Household Member**

No Observations

- **ha031_4_s18 : Which Household Member**

No Observations

- **ha031_4_s19 : Which Household Member**

No Observations

- **ha031_4_s20 : Which Household Member**

No Observations

- **ha031_4_s21 : Which Household Member**

No Observations

- **ha031_4_s22 : Which Household Member**

No Observations

- **ha031_4_s23 : Which Household Member**

No Observations

- **ha031_4_s24 : Which Household Member**

No Observations

- **ha031_4_s25 : Which Household Member**

No Observations

- **ha033_1_1_ : Which Household Member**

	No	%
1 HMemberNameAll[1]	7	53.85
2 HMemberNameAll[2]	1	7.69
3 HMemberNameAll[3]	5	38.46
Total	13	100.00

- **ha033_1_2_ : Which Household Member**

No Observations

- **ha033_1_3_ : Which Household Member**

No Observations

- **ha033_1_s1 : Ownership of House**

	No	%
1 Working unit of household member	14	100.00
Total	14	100.00

- **ha033_1_s2 : Ownership of House**

	No	%
2 Government indemnificatory housing	1	100.00
Total	1	100.00

- **ha033_1_s3 : Ownership of House**

	No	%
3 Child (non-household member) of main respondent or spouse	17	100.00
Total	17	100.00

- **ha033_1_s4 : Ownership of House**

	No	%
4 Parent (non-household member) of main respondent or spouse	2	100.00
Total	2	100.00

- **ha033_1_s5 : Ownership of House**

	No	%
5 Nonresident other relatives	22	100.00
Total	22	100.00

- **ha033_1_s6 : Ownership of House**

No Observations

- **ha033_1_s7 : Ownership of House**

	No	%
7 Other	13	100.00
Total	13	100.00

- **ha033_2_1_ : Child Not Living Together**

	No	%
1 FLSepChild[1]	3	17.65
2 FLSepChild[2]	11	64.71
3 FLSepChild[3]	2	11.76
5 FLSepChild[5]	1	5.88
Total	17	100.00

- **ha033_2_2_ : Child Not Living Together**

No Observations

- **ha033_2_3_ : Child Not Living Together**

No Observations

- **ha033_2_s1 : Ownership of House**

No Observations

- **ha033_2_s2 : Ownership of House**

No Observations

- **ha033_2_s3 : Ownership of House**

No Observations

- **ha033_2_s4 : Ownership of House**

	No	%
4 Parent (non-household member) of main respondent or spouse	1	100.00
Total	1	100.00

- **ha033_2_s5 : Ownership of House**

No Observations

- **ha033_2_s6 : Ownership of House**

No Observations

- **ha033_2_s7 : Ownership of House**

	No	%

7 Other	1	100.00
Total	1	100.00

- **ha033_3_1_ : Parents Not Living Together**

No Observations

- **ha033_3_2_ : Parents Not Living Together**

No Observations

- **ha033_3_3_ : Parents Not Living Together**

No Observations

- **ha033_3_s1 : Ownership of House**

No Observations

- **ha033_3_s2 : Ownership of House**

No Observations

- **ha033_3_s3 : Ownership of House**

No Observations

- **ha033_3_s4 : Ownership of House**

	No	%
4 Parent (non-household member) of main respondent or spouse	1	100.00
Total	1	100.00

- **ha033_3_s5 : Ownership of House**

No Observations

- **ha033_3_s6 : Ownership of House**

No Observations

- **ha033_3_s7 : Ownership of House**

No Observations

- **ha034_1_1_ : Total Price**

Mean	Min	Max	OBS
328.3	0.0	100,000.0	634

- **ha034_1_2_ : Total Price**

Mean	Min	Max	OBS
13.2	0.0	90.0	33

- **ha034_1_3_ : Total Price**

Mean	Min	Max	OBS
42.6	0.1	150.0	4

- **ha034_2_1_ : Unit Price**

Mean	Min	Max	OBS
533.0	0.2	8,000.0	40

- **ha034_2_2_ : Unit Price**

	No	%
2	1	20.00
3	2	40.00
4	1	20.00
18	1	20.00
Total	5	100.00

- **ha034_2_3_ : Unit Price**

No Observations

- **ha034_check : Are You Sure**

	No	%
1 Yes	18	94.74
2 No	1	5.26
Total	19	100.00

- **ha035_a_1_ : Min of Bracket**

Mean	Min	Max	OBS
65,180.9	0.0	500,001.0	83

- **ha035_a_2_ : Min of Bracket**

Mean	Min	Max	OBS
204,000.4	0.0	500,001.0	5

- **ha035_a_3_ : Min of Bracket**

	No	%
0	1	100.00
Total	1	100.00

- **ha035_b_1_ : Max of Bracket**

Mean	Min	Max	OBS
53,065,298.8	19,999.0	99,999,996.0	83

- **ha035_b_2_ : Max of Bracket**

Mean	Min	Max	OBS
40,129,998.0	49,999.0	99,999,996.0	5

- **ha035_b_3_ : Max of Bracket**

Mean	Min	Max	OBS
19,999.0	19,999.0	19,999.0	1

- **ha036_1_ : Take Out a Band Loan to Finance House**

	No	%
1 Yes	28	3.71
2 No	726	96.29
Total	754	100.00

- **ha036_2_ : Take Out a Band Loan to Finance House**

	No	%
1 Yes	3	7.14
2 No	39	92.86
Total	42	100.00

- **ha036_3_ : Take Out a Band Loan to Finance House**

	No	%
2 No	4	100.00
Total	4	100.00

- **ha037_1_ : Outstanding Amount of Loans**

	No	%
0	4	13.79
2	3	10.34
3	1	3.45
4	1	3.45
5	5	17.24
6	1	3.45
7	1	3.45
8	1	3.45
9	1	3.45

10	1	3.45
14	1	3.45
15	2	6.90
18	1	3.45
20	1	3.45
29	1	3.45
30	3	10.34
33	1	3.45
Total	29	100.00

- **ha037_2_ : Outstanding Amount of Loans**

	No	%
0	1	33.33
15	1	33.33
30	1	33.33
Total	3	100.00

- **ha038_1_ : Monthly Mortgage Payment**

Mean	Min	Max	OBS
1,105.6	0.0	4,000.0	26

- **ha038_2_ : Monthly Mortgage Payment**

Mean	Min	Max	OBS
1,833.3	0.0	3,500.0	3

- **ha039_1_ : How was this House Unit Obtained**

	No	%
1 Purchased from market	89	11.80
2 Purchased from working unit	50	6.63
3 Purchased from Child	1	0.13
4 Purchased from Parents	2	0.27
5 Purchased from Other relatives	6	0.80
6 Self-built	451	59.81
7 Inherited, bequeathed, or given	108	14.32
8 Received home as compensation for demolition of old home	27	3.58
9 Other	20	2.65
Total	754	100.00

- **ha039_1_1_ : R or Spouse**

	No	%
1 FLRespondents[1]	34	68.00
2 FLRespondents[2]	16	32.00
Total	50	100.00

- **ha039_1_2_ : R or Spouse**

No Observations

- **ha039_1_3_ : R or Spouse**

No Observations

- **ha039_2_ : How was this House Unit Obtained**

	No	%
1 Purchased from market	10	23.26
5 Purchased from Other relatives	1	2.33
6 Self-built	22	51.16
7 Inherited, bequeathed, or given	6	13.95
8 Received home as compensation for demolition of old home	3	6.98
9 Other	1	2.33
Total	43	100.00

- **ha039_2_1_ : Which Child**

	No	%
1 BC002[1]	1	100.00
Total	1	100.00

- **ha039_2_2_ : Which Child**

No Observations

- **ha039_2_3_ : Which Child**

No Observations

- **ha039_3_ : How was this House Unit Obtained**

	No	%
1 Purchased from market	2	50.00
5 Purchased from Other relatives	1	25.00
6 Self-built	1	25.00
Total	4	100.00

- **ha039_3_1_ : R or Spouse**

	No	%
1 FLRespondents[1]	2	100.00
Total	2	100.00

- **ha039_3_2_ : R or Spouse**

No Observations

- **ha039_3_3_ : R or Spouse**

No Observations

- **ha040_1_ : Price Type**

	No	%
1 Market price	84	57.14
2 Subsidized by working unit	52	35.37
3 Economic housing	2	1.36
4 Other	9	6.12
Total	147	100.00

- **ha040_2_ : Price Type**

	No	%
1 Market price	9	90.00
2 Subsidized by working unit	1	10.00

Total	10	100.00

- **ha040_3_ : Price Type**

	No	%
1 Market price	3	100.00
Total	3	100.00

- **ha041_1_ : When did You Purchase/Buid It**

Mean	Min	Max	OBS
1,993.0	1,930.0	2,011.0	480

- **ha041_2_ : When did You Purchase/Buid It**

Mean	Min	Max	OBS
1,997.4	1,978.0	2,011.0	19

- **ha041_3_ : When did You Purchase/Buid It**

No Observations

- **ha042_1_ : Can You Sell the House Freely**

	No	%
1 Yes	469	91.07
2 No, restricted by work unit	46	8.93
Total	515	100.00

- **ha042_2_ : Can You Sell the House Freely**

	No	%
1 Yes	23	95.83
2 No, restricted by work unit	1	4.17
Total	24	100.00

- **ha042_3_ : Can You Sell the House Freely**

	No	%
1 Yes	1	100.00
Total	1	100.00

- **ha043_1_ : Own Money Spend on the House**

Mean	Min	Max	OBS
897.5	-9,999.0	100,000.0	474

- **ha043_2_ : Own Money Spend on the House**

Mean	Min	Max	OBS
8.6	0.0	100.0	21

- **ha043_3_ : Own Money Spend on the House**

Mean	Min	Max	OBS
0.3	0.3	0.3	1

- **ha043_check : Are You Sure**

	No	%
1 Yes	10	90.91
2 No	1	9.09
Total	11	100.00

- **ha044_a_1_ : Min of Bracket**

Mean	Min	Max	OBS
16,097.7	0.0	100,001.0	41

- **ha044_a_2_ : Min of Bracket**

Mean	Min	Max	OBS
50,000.0	0.0	100,000.0	3

- **ha044_b_1_ : Max of Bracket**

Mean	Min	Max	OBS

39,057,071.1	19,999.0	99,999,996.0	41

- **ha044_b_2_ : Max of Bracket**

Mean	Min	Max	OBS
33,383,332.0	50,000.0	99,999,996.0	3

- **ha045_1_1_ : Total Price**

Mean	Min	Max	OBS
10.1	0.0	200.0	197

- **ha045_1_2_ : Total Price**

Mean	Min	Max	OBS
16.8	0.1	65.0	12

- **ha045_1_3_ : Total Price**

Mean	Min	Max	OBS
35.0	0.1	70.0	2

- **ha045_2_1_ : Unit Price**

Mean	Min	Max	OBS
402.8	0.2	8,000.0	22

- **ha045_2_2_ : Unit Price**

Mean	Min	Max	OBS
3.8	2.0	5.4	3

- **ha045_2_3_ : Unit Price**

Mean	Min	Max	OBS
1.2	1.2	1.2	1

- **ha045_check : Are You Sure**

	No	%
1 Yes	2	100.00
Total	2	100.00

- **ha046_1_ : Year**

Mean	Min	Max	OBS
2,001.1	1,958.0	2,011.0	27

- **ha046_2_ : Year**

Mean	Min	Max	OBS
2,007.3	2,007.0	2,008.0	3

- **ha047_1_ : Market Value of the Old House at That Time**

Mean	Min	Max	OBS
11.7	0.2	40.0	22

- **ha047_2_ : Market Value of the Old House at That Time**

	No	%
3	1	33.33
10	1	33.33
12	1	33.33
Total	3	100.00

- **ha047_check : Are You Sure**

No Observations

- **ha048_1_1_ : Total Price**

Mean	Min	Max	OBS
18.8	1.5	45.0	12

- **ha048_1_2_ : Total Price**

	No	%
20	1	100.00
Total	1	100.00

- **ha048_2_1_ : Unit Price**

Mean	Min	Max	OBS
1,265.1	1.6	4,500.0	11

- **ha048_2_2_ : Unit Price**

Mean	Min	Max	OBS
2,300.0	2,000.0	2,600.0	2

- **ha048_check : Are You Sure**

No Observations

- **ha049_1_ : Spending on Decorating or Renovating**

Mean	Min	Max	OBS
9,491.2	-9,999.0	400,000.0	687

- **ha049_2_ : Spending on Decorating or Renovating**

Mean	Min	Max	OBS
11,637.5	0.0	150,000.0	40

- **ha049_3_ : Spending on Decorating or Renovating**

Mean	Min	Max	OBS
62,500.0	0.0	200,000.0	4

- **ha050_1_ : Year**

Mean	Min	Max	OBS

1,992.6	1,900.0	2,012.0	569

- **ha050_2_ : Year**

Mean	Min	Max	OBS
1,999.7	1,900.0	2,011.0	30

- **ha050_3_ : Year**

Mean	Min	Max	OBS
2,004.5	2,000.0	2,009.0	2

- **ha051_1_ : Construction Area of the House**

Mean	Min	Max	OBS
102.5	0.0	1,300.0	726

- **ha051_2_ : Construction Area of the House**

Mean	Min	Max	OBS
123.8	20.0	1,400.0	39

- **ha051_3_ : Construction Area of the House**

Mean	Min	Max	OBS
137.0	30.0	400.0	5

- **ha052 : Any Rental Income**

	No	%
1 Yuan/month	100	10.10
2 Not applicable	890	89.90
Total	990	100.00

- **ha052_1 : Rental Income**

Mean	Min	Max	OBS
738.1	0.0	6,000.0	106

- **ha053 : Any Rental Income**

	No	%
1 Yuan/month	35	3.54
2 Not applicable	953	96.46
Total	988	100.00

- **ha053_1 : Rental Income**

Mean	Min	Max	OBS
607.1	0.0	4,000.0	35

- **ha054s1 : Does Your Household have Any**

	No	%
1 Cultivated land	6,796	100.00
Total	6,796	100.00

- **ha054s2 : Does Your Household have Any**

	No	%
2 Forest land	1,686	100.00
Total	1,686	100.00

- **ha054s3 : Does Your Household have Any**

	No	%
3 Pasture	76	100.00
Total	76	100.00

- **ha054s4 : Does Your Household have Any**

	No	%
4 Pond	97	100.00
Total	97	100.00

- **ha054s5 : Does Your Household have Any**

	No	%
5 None	3,080	100.00
Total	3,080	100.00

- **ha055_1_ : How Many Mu**

Mean	Min	Max	OBS
6.5	0.0	400.0	6,715

- **ha055_2_ : How Many Mu**

Mean	Min	Max	OBS
8.6	0.0	700.0	1,516

- **ha055_3_ : How Many Mu**

Mean	Min	Max	OBS
565.3	0.0	1,470.0	78

- **ha055_4_ : How Many Mu**

Mean	Min	Max	OBS
4.3	0.0	50.0	95

- **ha056_1_ : How Many Mu of Them are Irrigable**

Mean	Min	Max	OBS
2.8	0.0	200.0	6,705

- **ha057_1_ : Rent Per Mu**

Mean	Min	Max	OBS
304.5	0.0	50,000.0	6,023

- **ha057_2_ : Rent Per Mu**

Mean	Min	Max	OBS
150.1	0.0	10,000.0	1,241

- **ha057_3_ : Rent Per Mu**

Mean	Min	Max	OBS
85.6	0.0	800.0	65

- **ha057_4_ : Rent Per Mu**

Mean	Min	Max	OBS
1,561.1	0.0	100,000.0	83

- **ha058_1_ : Did You Rent Out**

	No	%
1 Yes	808	11.89
2 No	5,985	88.11
Total	6,793	100.00

- **ha058_2_ : Did You Rent Out**

	No	%
1 Yes	26	1.54
2 No	1,657	98.46
Total	1,683	100.00

- **ha058_3_ : Did You Rent Out**

	No	%
1 Yes	1	1.33
2 No	74	98.67
Total	75	100.00

- **ha058_4_ : Did You Rent Out**

	No	%
1 Yes	7	7.22
2 No	90	92.78
Total	97	100.00

- **ha059_1_ : How Much You Rent Out**

Mean	Min	Max	OBS

5.1	0.0	200.0	810

- **ha059_2_ : How Much You Rent Out**

Mean	Min	Max	OBS
6.9	0.0	45.0	25

- **ha059_3_ : How Much You Rent Out**

Mean	Min	Max	OBS
600.0	600.0	600.0	1

- **ha059_4_ : How Much You Rent Out**

	No	%
0	1	14.29
1	2	28.57
2	2	28.57
3	1	14.29
30	1	14.29
Total	7	100.00

- **ha060_1_ : Rental Income**

Mean	Min	Max	OBS
1,168.6	0.0	30,000.0	800

- **ha060_2_ : Rental Income**

Mean	Min	Max	OBS
771.1	0.0	10,000.0	26

- **ha060_3_ : Rental Income**

Mean	Min	Max	OBS
30,000.0	30,000.0	30,000.0	1

- **ha060_4_ : Rental Income**

Mean	Min	Max	OBS
43,385.7	0.0	300,000.0	7

- **ha061_1_ : Rent in Any Land**

	No	%
1 Yes	736	10.83
2 No	6,057	89.17
Total	6,793	100.00

- **ha061_2_ : Rent in Any Land**

	No	%
1 Yes	15	0.89
2 No	1,668	99.11
Total	1,683	100.00

- **ha061_3_ : Rent in Any Land**

	No	%
1 Yes	11	14.47
2 No	65	85.53
Total	76	100.00

- **ha061_4_ : Rent in Any Land**

	No	%
1 Yes	13	13.40
2 No	84	86.60
Total	97	100.00

- **ha062_1_ : How Much did You Rent**

Mean	Min	Max	OBS
7.7	0.0	200.0	736

- **ha062_2_ : How Much did You Rent**

Mean	Min	Max	OBS

89.2	0.0	1,200.0	18

- **ha062_3_ : How Much did You Rent**

Mean	Min	Max	OBS
809.1	300.0	3,500.0	11

- **ha062_4_ : How Much did You Rent**

Mean	Min	Max	OBS
12.1	0.1	40.0	13

- **ha063_1_ : How Much Rent Did You Pay**

Mean	Min	Max	OBS
1,529.6	0.0	70,000.0	730

- **ha063_2_ : How Much Rent Did You Pay**

Mean	Min	Max	OBS
994.7	0.0	6,000.0	15

- **ha063_3_ : How Much Rent Did You Pay**

Mean	Min	Max	OBS
5,254.5	600.0	16,000.0	11

- **ha063_4_ : How Much Rent Did You Pay**

Mean	Min	Max	OBS
8,609.2	100.0	40,000.0	13

- **ha064 : Any Rental Income**

	No	%
1 Yuan	78	0.78
2 Not applicable	9,925	99.22
Total	10,003	100.00

- **ha064_1 : Rental Income**

Mean	Min	Max	OBS
7,314.6	0.0	100,000.0	81

- **ha065_1_1_ : Current Value of Asset**

Mean	Min	Max	OBS
75,081.4	0.0	2,000,000.0	560

- **ha065_1_2_ : Current Value of Asset**

Mean	Min	Max	OBS
1,299.4	10.0	20,000.0	2,292

- **ha065_1_3_ : Current Value of Asset**

Mean	Min	Max	OBS
2,156.1	0.0	20,000.0	3,330

- **ha065_1_4_ : Current Value of Asset**

Mean	Min	Max	OBS
1,122.7	0.0	120,000.0	5,326

- **ha065_1_5_ : Current Value of Asset**

Mean	Min	Max	OBS
498.5	0.0	9,000.0	5,735

- **ha065_1_6_ : Current Value of Asset**

Mean	Min	Max	OBS
747.5	0.0	80,000.0	8,984

- **ha065_1_7_ : Current Value of Asset**

Mean	Min	Max	OBS

2,159.6	0.0	15,000.0	1,758

- **ha065_1_8_ : Current Value of Asset**

Mean	Min	Max	OBS
558.9	0.0	8,000.0	659

- **ha065_1_9_ : Current Value of Asset**

Mean	Min	Max	OBS
1,684.5	0.0	9,000.0	95

- **ha065_1_10_ : Current Value of Asset**

Mean	Min	Max	OBS
1,057.1	0.0	15,000.0	609

- **ha065_1_11_ : Current Value of Asset**

Mean	Min	Max	OBS
1,984.4	0.0	50,000.0	2,003

- **ha065_1_12_ : Current Value of Asset**

Mean	Min	Max	OBS
504.9	0.0	20,000.0	7,469

- **ha065_1_13_ : Current Value of Asset**

Mean	Min	Max	OBS
6,137.2	0.0	300,000.0	271

- **ha065_1_14_ : Current Value of Asset**

Mean	Min	Max	OBS
5,154.6	20.0	15,000.0	37

- **ha065_1_15_ : Current Value of Asset**

Mean	Min	Max	OBS
18,192.0	0.0	200,000.0	15

- **ha065_1_16_ : Current Value of Asset**

Mean	Min	Max	OBS
4,970.4	0.0	50,000.0	355

- **ha065_1_17_ : Current Value of Asset**

Mean	Min	Max	OBS
4,813.6	50.0	20,000.0	11

- **ha065s1 : Own this Asset**

	No	%
1 Automobile	578	100.00
Total	578	100.00

- **ha065s2 : Own this Asset**

	No	%
2 Electric Bicycle	2,305	100.00
Total	2,305	100.00

- **ha065s3 : Own this Asset**

	No	%
3 Motorcycle	3,380	100.00
Total	3,380	100.00

- **ha065s4 : Own this Asset**

	No	%
4 Refrigerator	5,485	100.00
Total	5,485	100.00

- **ha065s5 : Own this Asset**

	No	%
5 Washing machine	5,882	100.00
Total	5,882	100.00

- **ha065s6 : Own this Asset**

	No	%
6 TV	9,196	100.00
Total	9,196	100.00

- **ha065s7 : Own this Asset**

	No	%
7 Computer	1,853	100.00
Total	1,853	100.00

- **ha065s8 : Own this Asset**

	No	%
8 Stereo system	701	100.00
Total	701	100.00

- **ha065s9 : Own this Asset**

	No	%
9 Video camera	105	100.00
Total	105	100.00

- **ha065s10 : Own this Asset**

	No	%
10 Camera	644	100.00
Total	644	100.00

- **ha065s11 : Own this Asset**

	No	%
11 Air conditioner	2,074	100.00

Total	2,074	100.00

- **ha065s12 : Own this Asset**

	No	%
12 Mobile phone	7,679	100.00
Total	7,679	100.00

- **ha065s13 : Own this Asset**

	No	%
13 Furniture	279	100.00
Total	279	100.00

- **ha065s14 : Own this Asset**

	No	%
14 Music instrument	37	100.00
Total	37	100.00

- **ha065s15 : Own this Asset**

	No	%
15 Valuable decorations, ornaments, vases	18	100.00
Total	18	100.00

- **ha065s16 : Own this Asset**

	No	%
16 Treasures and precious metal	367	100.00
Total	367	100.00

- **ha065s17 : Own this Asset**

	No	%
17 Antiques, valuable paintings, etc	15	100.00
Total	15	100.00

- **ha065s18 : Own this Asset**

	No	%
18 None	437	100.00
Total	437	100.00

- **ha066_1_1_ : Current Value of Fixed Capital Assets**

Mean	Min	Max	OBS
5,240.6	100.0	600,000.0	945

- **ha066_1_2_ : Current Value of Fixed Capital Assets**

Mean	Min	Max	OBS
619.0	0.0	100,000.0	749

- **ha066_1_3_ : Current Value of Fixed Capital Assets**

Mean	Min	Max	OBS
2,015.1	15.0	50,000.0	325

- **ha066_1_4_ : Current Value of Fixed Capital Assets**

Mean	Min	Max	OBS
362.1	0.0	5,000.0	1,351

- **ha066_1_5_ : Current Value of Fixed Capital Assets**

Mean	Min	Max	OBS
2,506.0	10.0	300,000.0	423

- **ha066s1 : Own This Fixed Capital Asset**

	No	%
1 Tractor	957	100.00
Total	957	100.00

- **ha066s2 : Own This Fixed Capital Asset**

	No	%

2 Thresher	757	100.00
Total	757	100.00

- **ha066s3 : Own This Fixed Capital Asset**

	No	%
3 Tractor tools	326	100.00
Total	326	100.00

- **ha066s4 : Own This Fixed Capital Asset**

	No	%
4 Water pump	1,361	100.00
Total	1,361	100.00

- **ha066s5 : Own This Fixed Capital Asset**

	No	%
5 Processing equipment	422	100.00
Total	422	100.00

- **ha066s6 : Own This Fixed Capital Asset**

	No	%
6 None	7,369	100.00
Total	7,369	100.00

- **ha067 : Current Value of Other Fixed Capital Assets**

Mean	Min	Max	OBS
3,868.3	0.0	4,000,000.0	9,883

- **ha068 : Any Other Durable or Fixed Assets Worth 500 Yuan or More**

	No	%
1 Yes	562	5.64
2 No	9,401	94.36
Total	9,963	100.00

- **ha068_1 : Current Value**

Mean	Min	Max	OBS
5,281.3	0.0	200,000.0	572

- **ha069 : Lent to Other Families or Individuals and not Been Repaid**

	No	%
1 Yes	897	8.99
2 No	9,084	91.01
Total	9,981	100.00

- **ha070 : Total Amount of Loans**

Mean	Min	Max	OBS
33,771.5	0.0	10,000,000.0	919

- **ha071 : Interest Income from What You Lent to Others**

Mean	Min	Max	OBS
59.9	0.0	300,000.0	9,969

- **ha072 : Loans You are Still Owed to Others**

Mean	Min	Max	OBS
8,347.0	0.0	1,500,000.0	9,947

- **ha073_1 : 5,000**

	No	%
1 Less than 5,000 yuan	3	60.00
2 About 5,000 yuan	2	40.00
Total	5	100.00

- **ha073_2 : 10,000**

	No	%
1 Less than 10,000 yuan	5	27.78
2 About 10,000 yuan	4	22.22
3 More than 10,000 yuan	9	50.00

Total	18	100.00

- **ha073_3 : 50,000**

	No	%
1 Less than 50,000 yuan	19	55.88
2 About 50,000 yuan	9	26.47
3 More than 50,000 yuan	6	17.65
Total	34	100.00

- **ha073_4 : 100,000**

	No	%
1 Less than 100,000 yuan	2	40.00
2 About 100,000 yuan	2	40.00
3 More than 100,000 yuan	1	20.00
Total	5	100.00

- **ha073_5 : 200,000**

	No	%
1 Less than 200,000 yuan	1	100.00
Total	1	100.00

- **ha073_6 : 500,000**

No Observations

- **ha074_1_ : The Value of all Financial Assets of this Household Member**

Mean	Min	Max	OBS
5,610.9	0.0	400,000.0	712

- **ha074_2_ : The Value of all Financial Assets of this Household Member**

Mean	Min	Max	OBS
3,239.4	0.0	1,000,000.0	1,560

- **ha074_3_ : The Value of all Financial Assets of this Household Member**

Mean	Min	Max	OBS
2,447.1	0.0	500,000.0	5,085

- **ha074_4_ : The Value of all Financial Assets of this Household Member**

Mean	Min	Max	OBS
1,310.3	-10,000.0	200,000.0	3,634

- **ha074_5_ : The Value of all Financial Assets of this Household Member**

Mean	Min	Max	OBS
676.4	0.0	60,000.0	2,410

- **ha074_6_ : The Value of all Financial Assets of this Household Member**

Mean	Min	Max	OBS
1,093.9	0.0	100,000.0	1,201

- **ha074_7_ : The Value of all Financial Assets of this Household Member**

Mean	Min	Max	OBS
913.4	0.0	60,000.0	455

- **ha074_8_ : The Value of all Financial Assets of this Household Member**

Mean	Min	Max	OBS
2,470.6	0.0	150,000.0	214

- **ha074_9_ : The Value of all Financial Assets of this Household Member**

Mean	Min	Max	OBS
1,877.4	0.0	60,000.0	117

- **ha074_10_ : The Value of all Financial Assets of this Household Member**

Mean	Min	Max	OBS
264.4	0.0	10,000.0	59

- **ha074_11_ : The Value of all Financial Assets of this Household Member**

Mean	Min	Max	OBS
1,129.0	0.0	30,000.0	31

- **ha074_12_ : The Value of all Financial Assets of this Household Member**

Mean	Min	Max	OBS
55.6	0.0	1,000.0	18

- **ha074_13_ : The Value of all Financial Assets of this Household Member**

	No	%
0	10	100.00
Total	10	100.00

- **ha074_14_ : The Value of all Financial Assets of this Household Member**

Mean	Min	Max	OBS
1,666.7	0.0	10,000.0	6

- **ha074_15_ : The Value of all Financial Assets of this Household Member**

	No	%
0	2	100.00
Total	2	100.00

- **ha074_16_ : The Value of all Financial Assets of this Household Member**

	No	%
0	1	100.00
Total	1	100.00

- **ha074_check : Are You Sure**

No Observations

- **ha074_check_2_ : Are You Sure**

	No	%
1 Yes	1	100.00
Total	1	100.00

- **ha075_1_ : The Value of all Outstanding of this Household Member**

Mean	Min	Max	OBS
3,145.3	0.0	400,000.0	774

- **ha075_2_ : The Value of all Outstanding of this Household Member**

Mean	Min	Max	OBS
1,600.7	0.0	300,000.0	1,755

- **ha075_3_ : The Value of all Outstanding of this Household Member**

Mean	Min	Max	OBS
1,651.2	0.0	500,000.0	5,653

- **ha075_4_ : The Value of all Outstanding of this Household Member**

Mean	Min	Max	OBS
817.0	0.0	500,000.0	4,096

- **ha075_5_ : The Value of all Outstanding of this Household Member**

Mean	Min	Max	OBS
281.9	0.0	100,000.0	2,652

- **ha075_6_ : The Value of all Outstanding of this Household Member**

Mean	Min	Max	OBS
205.5	0.0	50,000.0	1,320

- **ha075_7_ : The Value of all Outstanding of this Household Member**

Mean	Min	Max	OBS

574.9	0.0	80,000.0	510

- **ha075_8_ : The Value of all Outstanding of this Household Member**

Mean	Min	Max	OBS
83.3	0.0	10,000.0	240

- **ha075_9_ : The Value of all Outstanding of this Household Member**

Mean	Min	Max	OBS
167.4	0.0	12,000.0	135

- **ha075_10_ : The Value of all Outstanding of this Household Member**

	No	%
0	67	100.00
Total	67	100.00

- **ha075_11_ : The Value of all Outstanding of this Household Member**

	No	%
0	34	100.00
Total	34	100.00

- **ha075_12_ : The Value of all Outstanding of this Household Member**

	No	%
0	19	100.00
Total	19	100.00

- **ha075_13_ : The Value of all Outstanding of this Household Member**

	No	%
0	10	100.00
Total	10	100.00

- **ha075_14_ : The Value of all Outstanding of this Household Member**

	No	%

0	6	100.00
Total	6	100.00

- **ha075_15_ : The Value of all Outstanding of this Household Member**

	No	%
0	2	100.00
Total	2	100.00

- **ha075_16_ : The Value of all Outstanding of this Household Member**

	No	%
0	1	100.00
Total	1	100.00

- **ha076 : Assistance in Section Household Income**

	No	%
1 Never	7,981	79.75
2 A few times	1,772	17.71
3 Most or all of the times	255	2.55
Total	10,008	100.00

- **proxy : Interview Down By Proxy**

	No	%
0 No	9,880	98.56
1 Yes	144	1.44
Total	10,024	100.00

9 Individual Income

- **ID : Individual ID**

A String Variable	
OBS:	17,424

- **householdID : Household ID**

A String Variable	
OBS:	17,424

- **communityID : Community ID**

A String Variable	
OBS:	17,424

- **g001 : Rate Standard of Living of Mr Wang**

	No	%
1 Very high	660	4.24
2 Relatively high	1,984	12.75
3 Average	8,220	52.84
4 Relatively poor	3,932	25.28
5 Poor	760	4.89
Total	15,556	100.00

- **g002 : Rate Standard of Living of Mr Zhang**

	No	%
1 Very high	137	0.87
2 Relatively high	1,189	7.59
3 Average	7,791	49.73
4 Relatively poor	5,157	32.91
5 Poor	1,394	8.90
Total	15,668	100.00

- **g003 : Rate Your Standard of Living**

	No	%
1 Very high	33	0.21
2 Relatively high	447	2.79

3 Average	8,470	52.82
4 Relatively poor	5,060	31.56
5 Poor	2,025	12.63
Total	16,035	100.00

- **ga001 : Receive Any Wage and Bonus Last Year**

	No	%
1 Yes	3,156	18.15
2 No	14,230	81.85
Total	17,386	100.00

- **ga002 : How Much did You Receive Last Year**

Mean	Min	Max	OBS
14,284.6	0.0	250,000.0	1,166

- **ga002_1 : Or Yuan/Month**

Mean	Min	Max	OBS
1,711.3	0.0	30,000.0	1,971

- **ga002_a : Min of Bracket**

Mean	Min	Max	OBS
7,692.5	0.0	100,001.0	39

- **ga002_b : Max of Bracket**

Mean	Min	Max	OBS
43,604,356.8	9,999.0	99,999,996.0	39

- **ga003s1 : Did You Receive this Type of Income**

	No	%
1 Pensions	3,459	100.00
Total	3,459	100.00

- **ga003s2 : Did You Receive this Type of Income**

	No	%
2 Unemployment compensation	18	100.00
Total	18	100.00

- **ga003s3 : Did You Receive this Type of Income**

	No	%
3 Pension subsidy	93	100.00
Total	93	100.00

- **ga003s4 : Did You Receive this Type of Income**

	No	%
4 Workers compensation	18	100.00
Total	18	100.00

- **ga003s5 : Did You Receive this Type of Income**

	No	%
5 Elderly family planning subsidies	80	100.00
Total	80	100.00

- **ga003s6 : Did You Receive this Type of Income**

	No	%
6 Medical aid	35	100.00
Total	35	100.00

- **ga003s7 : Did You Receive this Type of Income**

	No	%
7 Other government subsidies	510	100.00
Total	510	100.00

- **ga003s8 : Did You Receive this Type of Income**

	No	%
8 Social assistance	13	100.00
Total	13	100.00

- **ga003s9 : Did You Receive this Type of Income**

	No	%
9 Other income sources including alimony, child support	98	100.00
Total	98	100.00

- **ga003s10 : Did You Receive this Type of Income**

	No	%
10 None of the above	13,231	100.00
Total	13,231	100.00

- **ga004_1_1_ : Yuan/Year**

Mean	Min	Max	OBS
6,830.3	0.0	58,000.0	335

- **ga004_1_2_ : Yuan/Year**

Mean	Min	Max	OBS
4,257.5	40.0	8,400.0	6

- **ga004_1_3_ : Yuan/Year**

Mean	Min	Max	OBS
1,008.8	40.0	3,000.0	48

- **ga004_1_4_ : Yuan/Year**

Mean	Min	Max	OBS
1,553.3	0.0	10,000.0	9

- **ga004_1_5_ : Yuan/Year**

Mean	Min	Max	OBS
879.4	0.0	3,600.0	49

- **ga004_1_6_ : Yuan/Year**

Mean	Min	Max	OBS
2,812.7	50.0	15,000.0	20

- **ga004_1_7_ : Yuan/Year**

Mean	Min	Max	OBS
1,030.4	0.0	14,000.0	310

- **ga004_1_8_ : Yuan/Year**

Mean	Min	Max	OBS
6,305.0	0.0	70,000.0	12

- **ga004_1_9_ : Yuan/Year**

Mean	Min	Max	OBS
3,019.4	55.0	20,000.0	71

- **ga004_1_10_ : Yuan/Year**

Mean	Min	Max	OBS
1,000.0	1,000.0	1,000.0	1

- **ga004_1_11_ : Yuan/Year**

No Observations

- **ga004_2_1_ : Yuan/Month**

Mean	Min	Max	OBS
984.9	0.0	14,400.0	3,179

- **ga004_2_2_ : Yuan/Month**

Mean	Min	Max	OBS
452.9	0.0	940.0	11

- **ga004_2_3_ : Yuan/Month**

Mean	Min	Max	OBS
127.9	20.0	785.0	45

- **ga004_2_4_ : Yuan/Month**

Mean	Min	Max	OBS
361.9	60.0	1,000.0	9

- **ga004_2_5_ : Yuan/Month**

Mean	Min	Max	OBS
79.4	0.0	720.0	32

- **ga004_2_6_ : Yuan/Month**

Mean	Min	Max	OBS
180.0	10.0	500.0	10

- **ga004_2_7_ : Yuan/Month**

Mean	Min	Max	OBS
186.5	0.0	2,000.0	208

- **ga004_2_8_ : Yuan/Month**

	No	%
70	1	100.00
Total	1	100.00

- **ga004_2_9_ : Yuan/Month**

Mean	Min	Max	OBS
633.1	40.0	4,000.0	26

- **ga004_2_10_ : Yuan/Month**

No Observations

- **ga004_2_11_ : Yuan/Month**

No Observations

- **ga004_a_1_ : Min of Bracket**

Mean	Min	Max	OBS
7,070.6	0.0	100,000.0	611

- **ga004_b_1_ : Max of Bracket**

Mean	Min	Max	OBS
1,654,467.2	9,999.0	99,999,996.0	611

- **hb001 : Ever Purchased House Unit from Your Work Unit**

	No	%
1 Yes	757	4.35
2 No	16,631	95.65
Total	17,388	100.00

- **hb002 : Lived in this House Before Purchased it from Your Work Unit**

	No	%
1 Yes	468	59.69
2 No	316	40.31
Total	784	100.00

- **hb003 : Did You Obtain the Deed of Housing**

	No	%
1 Yes	702	91.76
2 No	63	8.24
Total	765	100.00

- **hb004 : Is this Your Current Residence**

	No	%
1 Yes	580	76.12
2 No	182	23.88
Total	762	100.00

- **hb005 : Do You Still Own the House**

	No	%
1 Yes	670	87.93
2 No	92	12.07
Total	762	100.00

- **hb006 : Year**

Mean	Min	Max	OBS
1,993.2	1,958.0	2,007.0	81

- **hb007_1 : Total Price**

Mean	Min	Max	OBS
446.4	0.1	35,000.0	79

- **hb007_2 : Unit Price**

Mean	Min	Max	OBS
147.4	0.1	700.0	8

- **hb007_check : Are You Sure**

	No	%
1 Yes	3	75.00
2 No	1	25.00
Total	4	100.00

- **hb008 : Amount Spent on Purchasing the House**

	No	%
4 20,000 to 50,000	1	100.00
Total	1	100.00

- **hb009 : Type of Price**

	No	%
1 Market price	8	8.89
2 Subsidized by working unit	75	83.33
4 other	7	7.78
Total	90	100.00

- **hb010_1 : Total Price**

Mean	Min	Max	OBS
851.1	0.1	48,000.0	57

- **hb010_2 : Unit Price**

Mean	Min	Max	OBS
140.8	0.8	700.0	5

- **hb010_check : Are You Sure**

	No	%
1 Yes	1	100.00
Total	1	100.00

- **hb011 : Construction Area**

Mean	Min	Max	OBS
70.6	20.0	230.0	90

- **hb012 : Year**

Mean	Min	Max	OBS
2,003.4	1,989.0	2,011.0	85

- **hb013_1 : Total Price**

Mean	Min	Max	OBS
2,444.5	0.0	190,000.0	78

- **hb013_2 : Unit Price**

Mean	Min	Max	OBS
126.3	0.0	500.0	4

- **hb013_check : Are You Sure**

	No	%
1 Yes	1	100.00
Total	1	100.00

- **hb014 : Total Price You Sold**

	No	%
1 less than 200,000	1	100.00
Total	1	100.00

- **hb015 : Who did You Sell the Housing Unit to**

	No	%
1 Back to work unit	5	5.68
2 Commercial market	51	57.95
3 Child, which one?	9	10.23
4 Parents, of who respondent or spouse?	1	1.14
5 Other relative ,specify	6	6.82
6 Other	16	18.18
Total	88	100.00

- **hb015_1 : Which Child**

	No	%
1 BC002[1]	2	22.22
2 BC002[2]	6	66.67
3 BC002[3]	1	11.11
Total	9	100.00

- **hb015_2 : R or Spouse**

	No	%
1 Respondent	1	100.00
Total	1	100.00

- **hb016 : Market Price or Below**

	No	%
1 Market price	60	69.77
2 Below market price	26	30.23
Total	86	100.00

- **hb017_1 : Total Price**

Mean	Min	Max	OBS
8.7	0.0	40.0	18

- **hb017_2 : Unit Price**

Mean	Min	Max	OBS
138.8	1.0	550.0	4

- **hb017_check : Are You Sure**

No Observations

- **hb018 : Reason Not Purchase House from Work Unit**

	No	%
1 I have no work unit	12,691	76.27
2 Not eligible to purchase	944	5.67
3 Eligible but no housing unit was made available	726	4.36
4 Eligible but could not afford	738	4.44
5 Eligible but decided not to purchase	307	1.84
6 Other	1,234	7.42
Total	16,640	100.00

- **hc001 : Cash Held by You and Your Spouse**

Mean	Min	Max	OBS
1,506.3	0.0	350,000.0	16,632

- **hc002_a : Min of Bracket**

Mean	Min	Max	OBS

862.0	0.0	10,001.0	771

- **hc002_b : Max of Bracket**

Mean	Min	Max	OBS
42,283,730.2	499.0	99,999,996.0	771

- **hc003_1 : Amount**

Mean	Min	Max	OBS
996.3	0.0	250,000.0	1,696

- **hc003_2 : Percentage**

Mean	Min	Max	OBS
53.9	0.0	5,000.0	10,211

- **hc004 : Holding Any Deposits**

	No	%
1 Yes	2,777	16.12
2 No	14,451	83.88
Total	17,228	100.00

- **hc005 : Amount of Deposits**

Mean	Min	Max	OBS
31,552.0	0.0	3,000,000.0	2,562

- **hc006_1 : 2,000 yuan**

	No	%
1 Less than 2,000 yuan	4	36.36
2 About 2,000 yuan	3	27.27
3 More than 2,000 yuan	4	36.36
Total	11	100.00

- **hc006_2 : 5,000 yuan**

	No	%
1 Less than 5,000 yuan	11	44.00
2 About 5,000 yuan	7	28.00
3 More than 5,000 yuan	7	28.00
Total	25	100.00

- **hc006_3 : 10,000 yuan**

	No	%
1 Less than 10,000 yuan	27	36.49
2 About 10,000 yuan	13	17.57
3 More than 10,000 yuan	34	45.95
Total	74	100.00

- **hc006_4 : 50,000 yuan**

	No	%
1 Less than 50,000 yuan	79	51.97
2 About 50,000 yuan	34	22.37
3 More than 50,000 yuan	39	25.66
Total	152	100.00

- **hc006_5 : 100,000 yuan**

	No	%
1 Less than 100,000 yuan	11	32.35
2 About 100,000 yuan	8	23.53
3 More than 100,000 yuan	15	44.12
Total	34	100.00

- **hc006_6 : 200,000 yuan**

	No	%
1 Less than 200,000 yuan	7	50.00
2 About 200,000 yuan	2	14.29
3 More than 200,000 yuan	5	35.71
Total	14	100.00

- **hc006_7 : 500,000 yuan**

	No	%

2 About 500,000 yuan	1	25.00
3 More than 500,000 yuan	3	75.00
Total	4	100.00

- **hc007 : Have Any Government Bonds**

	No	%
1 Yes	66	0.38
2 No	17,281	99.62
Total	17,347	100.00

- **hc008 : Total Face Value of Government Bonds**

Mean	Min	Max	OBS
19,783.8	0.0	150,000.0	61

- **hc009_a : Min of Bracket**

Mean	Min	Max	OBS
133,333.4	0.0	500,000.0	9

- **hc009_b : Max of Bracket**

Mean	Min	Max	OBS
22,363,332.1	9,999.0	99,999,996.0	9

- **hc010 : Held Any Stocks**

	No	%
1 Yes	136	0.78
2 No	17,232	99.22
Total	17,368	100.00

- **hc011 : Earned Money from Stock or Losed Money**

	No	%
1 Earned	9	6.38
2 Lost	94	66.67
3 Unchanged	38	26.95
Total	141	100.00

- **hc012 : How Much did You Earn**

Mean	Min	Max	OBS
10,687.5	200.0	50,000.0	8

- **hc012_2 : How Much did You Loss**

Mean	Min	Max	OBS
66,653.8	500.0	2,000,000.0	80

- **hc013 : Present Value of all the Stocks Currently Holding**

Mean	Min	Max	OBS
84,475.2	0.0	4,000,000.0	117

- **hc014_a : Min of Bracket**

Mean	Min	Max	OBS
54,210.7	0.0	500,000.0	19

- **hc014_b : Max of Bracket**

Mean	Min	Max	OBS
36,913,156.0	9,999.0	99,999,996.0	19

- **hc015 : Held Any Funds**

	No	%
1 Yes	106	0.61
2 No	17,264	99.39
Total	17,370	100.00

- **hc016 : Earned Money from Funds or Losed Money**

	No	%
1 Earned	6	5.77
2 Lost	84	80.77
3 Unchanged	14	13.46
Total	104	100.00

- **hc017 : How Much did You Earn or Loss**

Mean	Min	Max	OBS
19,409.8	1.0	200,000.0	78

- **hc018 : Present Value of all the Mutual Funds Currently Holding**

Mean	Min	Max	OBS
23,050.0	0.0	150,000.0	92

- **hc019_a : Min of Bracket**

Mean	Min	Max	OBS
16,428.9	0.0	100,001.0	14

- **hc019_b : Max of Bracket**

Mean	Min	Max	OBS
42,891,426.4	9,999.0	99,999,996.0	14

- **hc020 : Percentage that is Fully Controlled by You and Not Your Spouse**

	No	%
0	323	11.18
5	1	0.03
10	1	0.03
15	1	0.03
20	4	0.14
25	2	0.07
30	5	0.17
33	3	0.10
50	1,163	40.24
60	7	0.24
70	1	0.03
80	4	0.14
100	1,375	47.58
Total	2,890	100.00

- **hc021 : Any other Assets Belong to You But Held in a Person's Name Other Than You or You**

	No	%

1 Yes	148	1.03
2 No	14,177	98.97
Total	14,325	100.00

- **hc022 : Value of Such Assets**

Mean	Min	Max	OBS
24,489.7	0.0	200,000.0	135

- **hc023 : Receive any Other Income from Other Investment**

	No	%
1 Yes	155	0.89
2 No	17,230	99.11
Total	17,385	100.00

- **hc024 : Amount Received**

Mean	Min	Max	OBS
15,448.1	-100,000.0	500,000.0	154

- **hc025_a : Min of Bracket**

Mean	Min	Max	OBS
1,500.3	0.0	5,000.0	4

- **hc025_b : Max of Bracket**

Mean	Min	Max	OBS
50,002,497.8	4,999.0	99,999,996.0	4

- **hc026 : Percentage that is Owned Jointly with Your Spouse**

	No	%
0	20	12.90
50	78	50.32
100	57	36.77
Total	155	100.00

- **hc027 : Do You Have Public Housing Funding**

	No	%
1 Yes	450	2.59
2 No	16,927	97.41
Total	17,377	100.00

- **hc028 : Amount of Money in Your Public Housing Fund**

Mean	Min	Max	OBS
18,043.1	0.0	210,000.0	302

- **hc029_1 : 5,000 yuan**

	No	%
1 Less than 5,000 yuan	18	51.43
2 About 5,000 yuan	13	37.14
3 More than5,000 yuan	4	11.43
Total	35	100.00

- **hc029_2 : 10,000 yuan**

	No	%
1 Less than 10,000 yuan	34	40.00
2 About 10,000 yuan	10	11.76
3 More than 10,000 yuan	41	48.24
Total	85	100.00

- **hc029_3 : 50,000 yuan**

	No	%
1 Less than 50,000 yuan	89	77.39
2 About 50,000 yuan	17	14.78
3 More than 50,000 yuan	9	7.83
Total	115	100.00

- **hc029_4 : 100,000 yuan**

	No	%
1 Less than 100,000 yuan	4	57.14
2 About 100,000 yuan	2	28.57
3 More than 100,000 yuan	1	14.29

Total	7	100.00

- **hc029_5 : 200,000 yuan**

	No	%
3 More than 200,000 yuan	1	100.00
Total	1	100.00

- **hc029_6 : 500,000 yuan**

	No	%
2 About 500,000 yuan	1	100.00
Total	1	100.00

- **hc030 : Any Jizikuan**

	No	%
1 Yes	132	0.76
2 No	17,249	99.24
Total	17,381	100.00

- **hc031 : Amount of Your Jizikuan**

Mean	Min	Max	OBS
17,853.8	0.0	180,000.0	128

- **hc032_1 : 5,000 yuan**

	No	%
1 Less than 5,000 yuan	2	100.00
Total	2	100.00

- **hc032_2 : 10,000 yuan**

	No	%
1 Less than 10,000 yuan	2	100.00
Total	2	100.00

- **hc032_3 : 50,000 yuan**

	No	%
1 Less than 50,000 yuan	2	100.00
Total	2	100.00

- **hc032_4 : 100,000 yuan**

No Observations

- **hc032_5 : 200,000 yuan**

No Observations

- **hc032_6 : 500,000 yuan**

No Observations

- **hc033 : Any Unpaid Salary**

	No	%
1 Yes	412	2.37
2 No	16,969	97.63
Total	17,381	100.00

- **hc034 : Amount of Unpaid Salary**

Mean	Min	Max	OBS
11,036.9	0.0	700,000.0	397

- **hc035_a : Min of Bracket**

Mean	Min	Max	OBS
16,667.0	0.0	200,001.0	15

- **hc035_b : Max of Bracket**

Mean	Min	Max	OBS
26,674,998.4	4,999.0	99,999,996.0	15

- **hc036 : Participated in Any Rotating Savings and Credit Association**

	No	%
1 Yes	15	0.09
2 No	17,363	99.91
Total	17,378	100.00

- **hc037 : Total Amount Funds You Are Still Obligated to Pay**

Mean	Min	Max	OBS
2,278.6	0.0	10,000.0	14

- **hd001 : Total Amount of Loan not Repaid Yet**

Mean	Min	Max	OBS
3,338.0	0.0	1,000,000.0	17,341

- **hd002_1 : 5,000**

	No	%
1 Less than 5,000 yuan	4	66.67
3 More than 5,000 yuan	2	33.33
Total	6	100.00

- **hd002_2 : 10,000**

	No	%
1 Less than 10,000 yuan	5	45.45
2 About 10,000 yuan	1	9.09
3 More than 10,000 yuan	5	45.45
Total	11	100.00

- **hd002_3 : 50,000**

	No	%
1 Less than 50,000 yuan	14	58.33
2 About 50,000 yuan	4	16.67

3 More than 50,000 yuan	6	25.00
Total	24	100.00

- **hd002_4 : 100,000**

	No	%
1 Less than 100,000 yuan	2	40.00
2 About 100,000 yuan	2	40.00
3 More than 100,000 yuan	1	20.00
Total	5	100.00

- **hd002_5 : 200,000**

	No	%
3 More than 200,000 yuan	1	100.00
Total	1	100.00

- **hd002_6 : 500,000**

No Observations

- **hd003 : Amount of Your Credit Card Balance**

Mean	Min	Max	OBS
92.4	0.0	150,000.0	17,374

- **hd004_1 : 5,000**

	No	%
1 Less than 5,000 yuan	2	100.00
Total	2	100.00

- **hd004_2 : 10,000**

	No	%
1 Less than 10,000 yuan	2	100.00
Total	2	100.00

- **hd004_3 : 50,000**

	No	%
1 Less than 50,000 yuan	10	90.91
3 More than 50,000 yuan	1	9.09
Total	11	100.00

- **hd004_4 : 100,000**

No Observations

- **hd004_5 : 200,000**

No Observations

- **hd004_6 : 500,000**

No Observations

- **hd005 : Ever Inherited Anything**

	No	%
1 Yes	680	3.91
2 No	16,696	96.09
Total	17,376	100.00

- **hd006 : Amount Your Have Inherited**

Mean	Min	Max	OBS
17,570.0	0.0	1,000,000.0	648

- **hd007_1 : 5,000**

	No	%
1 Less than 5,000 yuan	15	55.56
2 About 5,000 yuan	9	33.33
3 More than 5,000 yuan	3	11.11
Total	27	100.00

- **hd007_2 : 10,000**

	No	%
1 Less than 10,000 yuan	28	71.79
2 About 10,000 yuan	6	15.38
3 More than 10,000 yuan	5	12.82
Total	39	100.00

- **hd007_3 : 50,000**

	No	%
1 Less than 50,000 yuan	40	88.89
2 About 50,000 yuan	1	2.22
3 More than 50,000 yuan	4	8.89
Total	45	100.00

- **hd007_4 : 100,000**

	No	%
1 Less than 100,000 yuan	1	50.00
2 About 100,000 yuan	1	50.00
Total	2	100.00

- **hd007_5 : 200,000**

No Observations

- **hd007_6 : 500,000**

No Observations

- **hd008s1 : From Whom You Inherited**

	No	%
1 Parents	622	100.00
Total	622	100.00

- **hd008s2 : From Whom You Inherited**

	No	%
2 Parents-in-law	34	100.00
Total	34	100.00

- **hd008s3 : From Whom You Inherited**

	No	%
3 Children	1	100.00
Total	1	100.00

- **hd008s4 : From Whom You Inherited**

	No	%
4 Relatives	13	100.00
Total	13	100.00

- **hd008s5 : From Whom You Inherited**

	No	%
5 Others	31	100.00
Total	31	100.00

- **hd009 : Year**

Mean	Min	Max	OBS
1,984.4	1,922.0	2,011.0	609

- **hd010 : Value of That Inheritance**

Mean	Min	Max	OBS
15,701.0	0.0	1,000,000.0	632

- **hd011_1 : 5,000**

	No	%
1 Less than 5,000 yuan	19	73.08
2 About 5,000 yuan	5	19.23
3 More than 5,000 yuan	2	7.69
Total	26	100.00

- **hd011_2 : 10,000**

	No	%
1 Less than 10,000 yuan	26	86.67
2 About 10,000 yuan	2	6.67
3 More than 10,000 yuan	2	6.67
Total	30	100.00

- **hd011_3 : 50,000**

	No	%
1 Less than 50,000 yuan	30	88.24
2 About 50,000 yuan	1	2.94
3 More than 50,000 yuan	3	8.82
Total	34	100.00

- **hd011_4 : 100,000**

	No	%
1 Less than 100,000 yuan	1	33.33
3 More than 100,000 yuan	2	66.67
Total	3	100.00

- **hd011_5 : 200,000**

	No	%
2 About 200,000 yuan	1	100.00
Total	1	100.00

- **hd011_6 : 500,000**

No Observations

- **hd012 : Assistance in Section Individual Income**

	No	%
1 Never	14,158	81.41
2 A few times	2,619	15.06
3 Most or all of the times	614	3.53
Total	17,391	100.00

10 Housing Characteristics

- **householdID : Household ID**

A String Variable	
OBS:	10,081

- **communityID : Community ID**

A String Variable	
OBS:	10,081

- **i001 : Construction Area of Your Residence**

Mean	Min	Max	OBS
112.3	0.0	1,000.0	9,794

- **i002 : Total Housing Land Area**

Mean	Min	Max	OBS
187.5	0.0	10,000.0	9,693

- **i003 : Residence is Used for Business As Well**

	No	%
1 Yes	626	6.22
2 No	9,443	93.78
Total	10,069	100.00

- **i004 : Structure Type of This Building**

	No	%
1 Concrete and steel	3,507	34.86
2 Bricks and wood	4,079	40.55
3 Mixed structure	981	9.75
4 Wood, bamboo, grass	283	2.81
5 Woolen felt	3	0.03
6 Sheet iron	1	0.01
7 Cave dwelling	114	1.13
8 Tent	1	0.01
9 adobe	954	9.48
10 Other	137	1.36

Total	10,060	100.00

- **i005 : Year**

Mean	Min	Max	OBS
1,992.1	1,900.0	2,011.0	9,286

- **i005_1 : Category**

	No	%
1 0-5years	23	3.10
2 5-10years	61	8.23
3 10-20years	154	20.78
4 20-30years	147	19.84
5 30-40years	92	12.42
6 above 40 years	264	35.63
Total	741	100.00

- **i006 : One Story or Multi-Level**

	No	%
1 One-story building	6,109	60.70
2 Multi-story building	3,955	39.30
Total	10,064	100.00

- **i007 : Is the Story Independent or Compound**

	No	%
1 Independent story	5,590	91.09
2 Compound	547	8.91
Total	6,137	100.00

- **i008 : Which Story is this Building on**

Mean	Min	Max	OBS
2.5	-1.0	501.0	3,950

- **i009 : Does It Has Elevator**

	No	%
1 Yes	90	4.24

2 No	2,032	95.76
Total	2,122	100.00

- **i010 : Are There Any Handicapped Facilities**

	No	%
1 Yes	2,591	25.74
2 No	7,474	74.26
Total	10,065	100.00

- **i011 : How Many Steps**

	No	%
1 1- 5	5,582	74.68
2 6 to 15	494	6.61
3 16 to 25	374	5.00
4 More than 25	1,025	13.71
Total	7,475	100.00

- **i012_1 : Num. of Bedrooms**

	No	%
0	20	0.20
1	1,467	14.62
2	3,423	34.11
3	2,579	25.70
4	1,303	12.98
5	456	4.54
6	366	3.65
7	110	1.10
8	131	1.31
9	51	0.51
10	61	0.61
11	12	0.12
12	27	0.27
13	2	0.02
14	5	0.05
15	2	0.02
16	4	0.04
17	1	0.01
18	4	0.04
20	8	0.08
24	1	0.01
30	1	0.01
36	1	0.01
Total	10,035	100.00

- **i012_2 : Num. of Living Rooms**

	No	%
0	1,587	15.81
1	6,619	65.96
2	1,559	15.54
3	162	1.61
4	62	0.62
5	13	0.13
6	9	0.09
7	2	0.02
8	5	0.05
9	5	0.05
10	3	0.03
11	9	0.09
Total	10,035	100.00

- **i012_3 : Num. of Toilets**

	No	%
0	2,728	27.18
1	6,352	63.30
2	696	6.94
3	152	1.51
4	46	0.46
5	15	0.15
6	10	0.10
7	3	0.03
8	4	0.04
9	2	0.02
10	2	0.02
11	25	0.25
Total	10,035	100.00

- **i012_4 : Num. of Kitchens**

	No	%
0	967	9.64
1	8,666	86.36
2	329	3.28
3	42	0.42
4	9	0.09
5	4	0.04
6	1	0.01
8	1	0.01
9	3	0.03
10	3	0.03
11	9	0.09

12	1	0.01
Total	10,035	100.00

- **i012_5 : Num. of Balcony**

	No	%
0	6,613	65.90
1	3,022	30.11
2	338	3.37
3	33	0.33
4	19	0.19
6	4	0.04
9	4	0.04
10	1	0.01
12	1	0.01
Total	10,035	100.00

- **i013 : How Far is the Nearest Toilet to Your House**

Mean	Min	Max	OBS
29.6	0.0	1,000.0	2,729

- **i014s1 : Type of Toilet**

	No	%
1 Toilet without a seat	8,324	100.00
Total	8,324	100.00

- **i014s2 : Type of Toilet**

	No	%
2 Toilet with a seat	1,858	100.00
Total	1,858	100.00

- **i015 : Is the Toilet Flushable**

	No	%
1 Yes	4,033	40.14
2 No	6,015	59.86
Total	10,048	100.00

- **i016 : Residence Has Electricity**

	No	%
1 Yes	8,273	82.22
2 No	1,789	17.78
Total	10,062	100.00

- **i017 : Residence Has Running Water**

	No	%
1 Yes	6,262	62.22
2 No	3,803	37.78
Total	10,065	100.00

- **i018 : In-House Shower or Bath Facility**

	No	%
1 Hot water provided	87	0.86
2 Water heater installed by the household	3,704	36.80
3 No	6,273	62.33
Total	10,064	100.00

- **i019 : Residence Has Coal Gas or Natural Gas Supply**

	No	%
1 Yes	1,285	12.77
2 No	8,780	87.23
Total	10,065	100.00

- **i020 : Residence Has Heating**

	No	%
1 Yes	991	9.85
2 No	9,075	90.15
Total	10,066	100.00

- **i021 : Heating Energy Source**

	No	%
1 Solar	204	2.25
2 Coal	2,642	29.17
3 Natural gas	173	1.91

4 Liquefied Petroleum Gas	179	1.98
5 Electric	1,377	15.20
6 Crop residue/Wood buring	2,778	30.67
7 Other	1,704	18.81
Total	9,057	100.00

- **i022 : Main Source of Cooking Fuel**

	No	%
1 Coal	1,052	10.45
2 Natural gas	1,304	12.96
3 Marsh gas	133	1.32
4 Liquefied Petroleum Gas	1,408	13.99
5 Electric	1,778	17.67
6 crop residue/Wood burning	4,310	42.83
7 other	79	0.78
Total	10,064	100.00

- **i023 : Residence Has a Telephone Connection**

	No	%
1 Yes	4,775	47.44
2 No	5,291	52.56
Total	10,066	100.00

- **i024 : Residence Has Broad-Band Internet Connection**

	No	%
1 Yes	1,645	16.36
2 No	8,411	83.64
Total	10,056	100.00

- **i025 : How Clear and Tity is in this Household**

	No	%
1 Excellent	870	8.66
2 Very clear	1,977	19.67
3 Clear	3,855	38.36
4 Fair	2,642	26.29
5 Poor	706	7.02
Total	10,050	100.00

- **i026 : How is the Temperature in this Household**

	No	%
1 Very hot	204	2.03
2 Hot	1,047	10.42
3 Bearable	8,351	83.12
4 Cold	377	3.75
5 Very cold	68	0.68
Total	10,047	100.00

11 Interviewer Observation

- **ID : Individual ID**

A String Variable	
OBS:	17,326

- **householdID : Household ID**

A String Variable	
OBS:	17,326

- **communityID : Community ID**

A String Variable	
OBS:	17,326

- **j001s1 : Any Third Persons Present During The Inerview**

	No	%
1 Nobody	5,432	100.00
Total	5,432	100.00

- **j001s2 : Any Third Persons Present During The Inerview**

	No	%
2 Spouse or partner	8,163	100.00
Total	8,163	100.00

- **j001s3 : Any Third Persons Present During The Inerview**

	No	%
3 Parent or parents	265	100.00
Total	265	100.00

- **j001s4 : Any Third Persons Present During The Inerview**

	No	%
4 Child or children	3,019	100.00
Total	3,019	100.00

- **j001s5 : Any Third Persons Present During The Inerview**

	No	%
5 Other relatives	1,338	100.00
Total	1,338	100.00

- **j001s6 : Any Third Persons Present During The Inerview**

	No	%
6 Other persons present	2,514	100.00
Total	2,514	100.00

- **j002 : Intervene During The Interview**

	No	%
1 Yes, often	538	5.97
2 Yes, occasionally	3,611	40.10
3 No	4,856	53.93
Total	9,005	100.00

- **j003 : Describe the Willingness of Respondent**

	No	%
1 Very good	9,763	56.39
2 Good	5,816	33.59
3 Fair	1,135	6.56
4 Bad	296	1.71
5 Good in the beginning, got worse during the interview	200	1.16
6 Bad in the beginning, got better during the interview	104	0.60
Total	17,314	100.00

- **j004s1 : Reason that Respondents' Willingness to Answer Get Worse During the Interview**

	No	%
1 The respondent was losing interest	59	100.00
Total	59	100.00

- **j004s2 : Reason that Respondents' Willingness to Answer Get Worse During the Interview**

	No	%
2 The respondent was losing concentration or was getting tired	115	100.00
Total	115	100.00

- **j004s3 : Reason that Respondents' Willingness to Answer Get Worse During the Interview**

	No	%
3 Other, please specify	62	100.00
Total	62	100.00

- **j005 : Respondents Ask for Clarification on Questions**

	No	%
1 Never	3,715	21.46
2 Almost never	6,695	38.67
3 Now and then	4,273	24.68
4 Often	1,808	10.44
5 Very often	549	3.17
6 Always	275	1.59
Total	17,315	100.00

- **j006 : Understood or Not**

	No	%
1 Never	186	1.07
2 Almost never	317	1.83
3 Now and then	873	5.04
4 Often	5,176	29.89
5 Very often	7,336	42.37
6 Always	3,427	19.79
Total	17,315	100.00

- **j007 : Respondents Need Help Reading the Showcards during the Interview**

	No	%
1 Yes, due to sight problems	2,920	16.87
2 Yes, due to literacy problems	5,451	31.49
3 No	8,942	51.65
Total	17,313	100.00

12 Biomarkers

- **ID : Individual ID**

A String Variable	
OBS:	13,974

- **householdID : Household ID**

A String Variable	
OBS:	13,974

- **communityID : Community ID**

A String Variable	
OBS:	13,974

- **pa001 : Measure or Not**

	No	%
1 Yes	14	0.10
5 No	13,833	99.90
Total	13,847	100.00

- **pa002 : Understand Directions or Not**

	No	%
1 YES	13,853	99.79
5 NO	29	0.21
Total	13,882	100.00

- **qa001_1 : Other (Specify)**

Chinese Character String

- **qa001s1 : Why didn't Complete**

	No	%
1 R felt it would not be safe	10	100.00

Total	10	100.00

- **qa001s2 : Why didn't Complete**

	No	%
2 IWER felt it would not be safe	1	100.00
Total	1	100.00

- **qa001s3 : Why didn't Complete**

	No	%
3 R refused or was not willing to complete the test	9	100.00
Total	9	100.00

- **qa001s4 : Why didn't Complete**

	No	%
4 R tried but was unable to complete test	40	100.00
Total	40	100.00

- **qa001s5 : Why didn't Complete**

	No	%
5 R did not understand the instructions	1	100.00
Total	1	100.00

- **qa001s6 : Why didn't Complete**

	No	%
6 R had a rash, a cast, edema, etc. on arm; other health reason	4	100.00
Total	4	100.00

- **qa001s7 : Why didn't Complete**

	No	%
7 No suitable space	1	100.00
Total	1	100.00

- **qa001s8 : Why didn't Complete**

	No	%
8 Problem with equipment or supplies	35	100.00
Total	35	100.00

- **qa001s97 : Why didn't Complete**

	No	%
97 Other Specify	22	100.00
Total	22	100.00

- **qa002 : Minute**

	No	%
.D	23	0.17
.E	25	0.18
.R	2	0.01
00	257	1.85
01	220	1.59
02	225	1.62
03	206	1.49
04	230	1.66
05	217	1.56
06	227	1.64
07	217	1.56
08	235	1.69
09	242	1.74
1	1	0.01
10	246	1.77
11	235	1.69
12	242	1.74
13	201	1.45
14	237	1.71
15	243	1.75
16	248	1.79
17	223	1.61
18	235	1.69
19	232	1.67
20	241	1.74
21	224	1.61
22	216	1.56
23	236	1.70
24	217	1.56
25	240	1.73
26	215	1.55
27	224	1.61
28	225	1.62
29	204	1.47

30	289	2.08
31	230	1.66
32	223	1.61
33	232	1.67
34	203	1.46
35	232	1.67
36	207	1.49
37	221	1.59
38	230	1.66
39	225	1.62
40	245	1.77
41	229	1.65
42	222	1.60
43	250	1.80
44	223	1.61
45	213	1.54
46	228	1.64
47	263	1.90
48	217	1.56
49	221	1.59
50	250	1.80
51	262	1.89
52	202	1.46
53	258	1.86
54	213	1.54
55	246	1.77
56	219	1.58
57	245	1.77
58	225	1.62
59	230	1.66
60	1	0.01
7	1	0.01
82	1	0.01
9	3	0.02
Total	13,870	100.00

- **qa002_1 : Hour**

	No	%
.D	23	0.17
.E	24	0.17
.R	2	0.01
0	10	0.07
00	34	0.25
1	8	0.06
10	1,186	8.55
101	2	0.01
11	1,248	9.00
111	1	0.01
1118	1	0.01
11:24	1	0.01

12	840	6.06
13	843	6.08
131	1	0.01
1312	1	0.01
14	962	6.94
141	1	0.01
15	1,185	8.54
150	1	0.01
151	2	0.01
1511	1	0.01
1512	1	0.01
15e	1	0.01
16	1,294	9.33
161	1	0.01
17	1,246	8.98
170	1	0.01
171	2	0.01
1716	1	0.01
17165	1	0.01
18	1,080	7.79
19	792	5.71
1945	1	0.01
2	7	0.05
20	651	4.69
2013	1	0.01
21	406	2.93
22	155	1.12
23	31	0.22
24	1	0.01
3	2	0.01
4	3	0.02
5	22	0.16
6	92	0.66
7	257	1.85
8	602	4.34
81	1	0.01
9	839	6.05
91	1	0.01
9112	1	0.01
Total	13,870	100.00

- **qa003 : Systolic Reading**

Mean	Min	Max	OBS
136.4	61.0	993.0	13,829

- **qa004 : Diastolic Reading**

Mean	Min	Max	OBS

76.9	30.0	145.0	13,772

- **qa005 : Pulse**

Mean	Min	Max	OBS
72.8	30.0	138.0	13,768

- **qa006 : Hour**

	No	%
.D	31	0.22
.E	1	0.01
.R	5	0.04
0	8	0.06
00	3	0.02
01	31	0.22
02	1	0.01
1	9	0.07
10	1,167	8.46
101	1	0.01
109	1	0.01
10e	1	0.01
11	1,239	8.98
11104	1	0.01
1111	1	0.01
11158	1	0.01
11‘	2	0.01
12	854	6.19
12152	1	0.01
13	842	6.10
13136	1	0.01
1397	1	0.01
14	946	6.86
15	1,177	8.53
151	1	0.01
15164	1	0.01
16	1,281	9.28
16115	1	0.01
16167	1	0.01
16178	1	0.01
162	1	0.01
1695	1	0.01
17	1,249	9.05
171	2	0.01
17199	1	0.01
18	1,083	7.85
181	1	0.01
18111	1	0.01
18&1	0.01	

19	797	5.78
191	1	0.01
19178	1	0.01
19&1	0.01	
1&1	0.01	
1	1	0.01
2	7	0.05
20	640	4.64
201	1	0.01
20116	1	0.01
21	417	3.02
22	158	1.15
23	33	0.24
25	1	0.01
3	2	0.01
4	2	0.01
5	23	0.17
6	88	0.64
7	249	1.80
71	2	0.01
8	587	4.25
8103	1	0.01
8104	1	0.01
9	828	6.00
91	1	0.01
9129	1	0.01
9150	1	0.01
9171	1	0.01
Total	13,798	100.00

- **qa006_1 : Minute**

	No	%
.D	31	0.22
.E	9	0.07
.R	5	0.04
00	243	1.76
01	225	1.63
02	224	1.62
03	195	1.41
04	257	1.86
05	200	1.45
06	218	1.58
07	251	1.82
08	195	1.41
09	245	1.78
10	232	1.68
11	236	1.71
12	248	1.80
13	209	1.51
137	1	0.01

14	230	1.67
15	245	1.78
16	237	1.72
17	238	1.72
18	224	1.62
19	233	1.69
20	266	1.93
21	229	1.66
22	218	1.58
23	215	1.56
24	235	1.70
25	203	1.47
26	229	1.66
27	212	1.54
28	247	1.79
29	215	1.56
30	238	1.72
31	242	1.75
32	213	1.54
33	225	1.63
34	232	1.68
35	221	1.60
36	225	1.63
37	210	1.52
38	204	1.48
39	243	1.76
40	228	1.65
41	239	1.73
42	215	1.56
43	243	1.76
44	210	1.52
45	246	1.78
46	220	1.59
47	243	1.76
48	231	1.67
49	227	1.65
50	235	1.70
51	259	1.88
52	227	1.65
53	238	1.72
54	243	1.76
55	237	1.72
56	212	1.54
57	233	1.69
58	237	1.72
59	210	1.52
60	2	0.01
64	1	0.01
7	1	0.01
74	1	0.01
87	1	0.01
9	1	0.01

91	1	0.01
93	1	0.01
95	1	0.01
97	1	0.01
Z	1	0.01
Total	13,798	100.00

- **qa007 : Systolic Reading**

Mean	Min	Max	OBS
131.5	60.0	993.0	13,761

- **qa008 : Diastolic Reading**

Mean	Min	Max	OBS
75.8	35.0	143.0	13,739

- **qa009 : Pulse**

Mean	Min	Max	OBS
72.2	33.0	153.0	13,734

- **qa010 : Hour**

	No	%
.D	29	0.21
.E	6	0.04
.R	8	0.06
0	8	0.06
01	2	0.01
02	21	0.15
03	10	0.07
04	1	0.01
1	3	0.02
10	1,162	8.43
10101	1	0.01
10‘	1	0.01
11	1,241	9.01
11155	1	0.01
12	858	6.23
121	1	0.01
12103	1	0.01
12149	1	0.01
13	827	6.00
131	2	0.01

135	1	0.01
14	949	6.89
15	1,160	8.42
151	1	0.01
16	1,284	9.32
161	1	0.01
17	1,261	9.15
171	1	0.01
17112	1	0.01
17127	1	0.01
17132	1	0.01
18	1,079	7.83
181	1	0.01
18128	1	0.01
18160	1	0.01
19	805	5.84
19130	1	0.01
19137	1	0.01
19148	1	0.01
2	6	0.04
20	648	4.70
21	416	3.02
21104	2	0.01
22	161	1.17
23	34	0.25
26	1	0.01
3	5	0.04
4	2	0.01
5	23	0.17
6	80	0.58
7	255	1.85
8	585	4.25
81	1	0.01
9	818	5.94
90	1	0.01
91	2	0.01
9132	1	0.01
9‘	1	0.01
Total	13,777	100.00

- **qa010_1 : Minute**

	No	%
.D	29	0.21
.E	12	0.09
.R	8	0.06
00	197	1.43
01	224	1.63
02	239	1.73
03	226	1.64
04	207	1.50

05	216	1.57
06	235	1.71
07	233	1.69
08	225	1.63
09	224	1.63
10	224	1.63
11	239	1.73
117	1	0.01
12	228	1.65
13	231	1.68
132	1	0.01
14	223	1.62
15	235	1.71
156	1	0.01
16	229	1.66
17	238	1.73
18	232	1.68
19	219	1.59
20	241	1.75
21	249	1.81
22	242	1.76
23	220	1.60
24	230	1.67
25	219	1.59
26	215	1.56
27	223	1.62
28	242	1.76
29	215	1.56
30	218	1.58
31	217	1.58
32	270	1.96
33	204	1.48
34	219	1.59
35	220	1.60
36	242	1.76
37	215	1.56
38	206	1.50
39	205	1.49
40	225	1.63
41	246	1.79
42	218	1.58
43	240	1.74
44	240	1.74
45	219	1.59
46	219	1.59
47	234	1.70
48	237	1.72
49	238	1.73
50	221	1.60
51	230	1.67
52	256	1.86
53	232	1.68

54	232	1.68
55	240	1.74
56	256	1.86
57	229	1.66
58	212	1.54
59	251	1.82
60	3	0.02
61	1	0.01
64	1	0.01
67	2	0.01
69	2	0.01
71	1	0.01
75	1	0.01
9	3	0.02
Total	13,777	100.00

- **qa011 : Systolic Reading**

Mean	Min	Max	OBS
130.1	61.0	993.0	13,729

- **qa012 : Diastolic Reading**

Mean	Min	Max	OBS
75.2	32.0	141.0	13,712

- **qa013 : Pulse**

Mean	Min	Max	OBS
72.5	33.0	166.0	13,704

- **qa014 : Which Arm**

	No	%
1 Left arm	11,812	85.72
2 Right arm	1,967	14.28
Total	13,779	100.00

- **qa015 : How Compliant**

	No	%
1 R was fully compliant	13,760	99.76
2 R was prevented from fully complying due to illness, pain, or other symptoms or discomforts	22	0.16

	No	%
3 R was not fully compliant, but no obvious reason for this	11	0.08
Total	13,793	100.00

- **qa016 : R's Position**

	No	%
1 Standing	305	2.21
2 Sitting	13,449	97.53
3 Lying down	35	0.25
Total	13,789	100.00

- **qa017 : Smoke, Exercise or Consume Alcohol or Food**

	No	%
1 Yes	2,554	18.52
5 No	11,186	81.10
8 Dont know	53	0.38
Total	13,793	100.00

- **pb001 : Understand Directions or Not**

	No	%
1 Yes	13,453	97.30
5 No	373	2.70
Total	13,826	100.00

- **qb001_1 : Other (Specify)**

Chinese Character String

- **qb001s1 : Why didn't Complete**

	No	%
1 R felt it would not be safe	21	100.00
Total	21	100.00

- **qb001s2 : Why didn't Complete**

	No	%
2 IWER felt it would not be safe	23	100.00

Total	23	100.00

- **qb001s3 : Why didn't Complete**

	No	%
3 R refused or was not willing to complete the test	55	100.00
Total	55	100.00

- **qb001s4 : Why didn't Complete**

	No	%
4 R tried but was unable to complete test	121	100.00
Total	121	100.00

- **qb001s5 : Why didn't Complete**

	No	%
5 R did not understand the instructions	13	100.00
Total	13	100.00

- **qb001s6 : Why didn't Complete**

	No	%
6 R could not participate due to health reasons	104	100.00
Total	104	100.00

- **qb001s7 : Why didn't Complete**

	No	%
7 No suitable space	1	100.00
Total	1	100.00

- **qb001s8 : Why didn't Complete**

	No	%
8 Problem with equipment or supplies	253	100.00
Total	253	100.00

- **qb001s97 : Why didn't Complete**

	No	%
97 Other Specify	7	100.00
Total	7	100.00

- **qb002 : Measurement Reading-1**

Mean	Min	Max	OBS
247.1	30.0	999.0	13,466

- **qb003 : Measurement Reading-2**

Mean	Min	Max	OBS
268.2	30.0	993.0	13,259

- **qb004 : Meaurement Reading-3**

Mean	Min	Max	OBS
279.9	30.0	999.0	13,256

- **qb005 : How Effort**

	No	%
1 R gave full effort	12,636	95.14
2 R was prevented from giving full effort by illness, pain, or other symptoms or discomforts	164	1.23
3 R did not appear to give full effort, but no obvious reason for this	482	3.63
Total	13,282	100.00

- **qb006 : R's Position**

	No	%
1 Standing	10,746	80.96
2 Sitting	2,517	18.96
3 Lying down	10	0.08
Total	13,273	100.00

- **pc001 : Any Problem**

	No	%
1 Yes	444	3.20

5 No	13,443	96.80
Total	13,887	100.00

- **pc002 : Which Hand**

	No	%
1 BOTH HANDS	203	26.99
2 LEFT HAND ONLY	269	35.77
3 RIGHT HAND ONLY	280	37.23
Total	752	100.00

- **pc003 : Understand Directions or Not**

	No	%
1 YES	13,613	98.59
5 NO	194	1.41
Total	13,807	100.00

- **qc001_1 : Other (Specify)**

Chinese Character String

- **qc001s1 : Why didn't Complete**

	No	%
1 R felt it would not be safe	31	100.00
Total	31	100.00

- **qc001s2 : Why didn't Complete**

	No	%
2 IWER felt it would not be safe	33	100.00
Total	33	100.00

- **qc001s3 : Why didn't Complete**

	No	%
3 R refused or was not willing to complete the test	24	100.00
Total	24	100.00

- **qc001s4 : Why didn't Complete**

	No	%
4 R tried but was unable to complete test	15	100.00
Total	15	100.00

- **qc001s5 : Why didn't Complete**

No Observations

- **qc001s6 : Why didn't Complete**

	No	%
6 R could not participate due to surgery, swelling, etc.; other health reason	130	100.00
Total	130	100.00

- **qc001s7 : Why didn't Complete**

No Observations

- **qc001s8 : Why didn't Complete**

	No	%
8 Problem with equipment or supplies	100	100.00
Total	100	100.00

- **qc001s97 : Why didn't Complete**

	No	%
97 Other Specify:	20	100.00
Total	20	100.00

- **qc002 : Dominant Hand**

	No	%
1 Right hand	12,025	88.28
2 Left hand	1,006	7.39
3 Both hands equally dominant	591	4.34

Total	13,622	100.00

- **qc003 : Left Hand-1kg**

Mean	Min	Max	OBS
29.6	0.0	999.0	13,550

- **qc004 : Right Hand-1kg**

Mean	Min	Max	OBS
31.0	0.0	999.0	13,520

- **qc005 : Left Hand-2kg**

Mean	Min	Max	OBS
29.6	0.0	999.0	13,535

- **qc006 : Right Hand-2kg**

Mean	Min	Max	OBS
31.5	0.0	999.0	13,509

- **qc007 : How Much Effort**

	No	%
1 R gave full effort	13,328	98.17
2 R was prevented from giving full effort by illness, pain, or other symptoms or discomforts	113	0.83
3 R did not appear to give full effort, but no obvious reason for this	136	1.00
Total	13,577	100.00

- **qc008 : R's Position**

	No	%
1 Standing	11,102	81.81
2 Sitting	2,459	18.12
3 Lying down	10	0.07
Total	13,571	100.00

- **qc009 : R Rest Their Arm or Not**

	No	%
1 YES	11,648	85.84
5 NO	1,922	14.16
Total	13,570	100.00

- **pd001 : Any Problem**

	No	%
1 YES	1,877	13.52
5 NO	12,004	86.48
Total	13,881	100.00

- **pd002 : Understand Directions or Not**

	No	%
1 YES	13,516	97.38
5 NO	364	2.62
Total	13,880	100.00

- **qd001_1 : Other (Specify)**

Chinese Character String

- **qd001s1 : Why didn't Complete**

	No	%
1 R felt it would not be safe	95	100.00
Total	95	100.00

- **qd001s2 : Why didn't Complete**

	No	%
2 IWER felt it would not be safe	150	100.00
Total	150	100.00

- **qd001s3 : Why didn't Complete**

	No	%

3 R refused or was not willing to complete the test	29	100.00
Total	29	100.00

- **qd001s4 : Why didn't Complete**

	No	%
4 R tried but was unable to complete test	95	100.00
Total	95	100.00

- **qd001s5 : Why didn't Complete**

	No	%
5 R did not understand the instructions	8	100.00
Total	8	100.00

- **qd001s6 : Why didn't Complete**

	No	%
6 R had surgery, injury or other health condition that prevented R from standing	274	100.00
Total	274	100.00

- **qd001s7 : Why didn't Complete**

	No	%
7 No suitable space	8	100.00
Total	8	100.00

- **qd001s8 : Why didn't Complete**

No Observations

- **qd001s97 : Why didn't Complete**

	No	%
97 Other Specify	42	100.00
Total	42	100.00

- **qd002 : How Complete**

	No	%
1 YES	13,159	97.32
5 NO Enter amount of time R held stand in seconds to two decimal places: Sec	182	1.35
993 R tried but was unable	126	0.93
999 R chose not to do it	54	0.40
Total	13,521	100.00

- **qd003 : Seconds**

Mean	Min	Max	OBS
5.5	0.0	10.0	179

- **qd004 : Use Any Compensatory Movements**

	No	%
1 YES	1,391	10.43
5 NO	11,932	89.43
8 Dont Know	19	0.14
Total	13,342	100.00

- **pe001 : Which Test Item**

	No	%
1 30 seconds full-tandem balance measurement	1,985	15.09
5 60seconds full-tandem balance measurement	11,171	84.91
Total	13,156	100.00

- **pe002 : Understand Directions or Not**

	No	%
1 YES	13,033	99.05
5 NO	125	0.95
Total	13,158	100.00

- **qe001_1 : Other (Specify)**

Chinese Character String

- **qe001s1 : Why didn't Complete**

	No	%
1 R felt it would not be safe	72	100.00
Total	72	100.00

- **qe001s2 : Why didn't Complete**

	No	%
2 IWER felt it would not be safe	71	100.00
Total	71	100.00

- **qe001s3 : Why didn't Complete**

	No	%
3 R refused or was not willing to complete the test	19	100.00
Total	19	100.00

- **qe001s4 : Why didn't Complete**

	No	%
4 R tried but was unable to complete test	155	100.00
Total	155	100.00

- **qe001s5 : Why didn't Complete**

	No	%
5 R did not understand the instructions	3	100.00
Total	3	100.00

- **qe001s6 : Why didn't Complete**

	No	%
6 R had surgery, injury or other health condition that prevented R from standing	110	100.00
Total	110	100.00

- **qe001s7 : Why didn't Complete**

	No	%
7 No suitable space	1	100.00
Total	1	100.00

- **qe001s8 : Why didn't Complete**

No Observations

- **qe001s97 : Why didn't Complete**

	No	%
97 Other Specify	27	100.00
Total	27	100.00

- **qe002 : How Complete**

	No	%
1 YES	10,023	76.90
5 NO Enter amount of time R held stand in seconds to two decimal places: Sec	2,761	21.18
993 R tried but was unable	212	1.63
999 R chose not to do it	38	0.29
Total	13,034	100.00

- **qe003 : Seconds**

Mean	Min	Max	OBS
22.4	0.0	60.0	2,753

- **qe004 : Use Any Compensatory Movements**

	No	%
1 YES	3,410	26.67
5 NO	9,359	73.21
8 Dont Know	15	0.12
Total	12,784	100.00

- **qe005 : Type of Floor Surface**

	No	%
1 Linoleum/tile/wood	5,454	42.65
2 carpet	29	0.23
3 Clay	585	4.57
4 Concrete	6,404	50.08
5 Not sure	38	0.30
97 Other Specify	277	2.17

Total	12,787	100.00

- **qe005_1 : Other (Specify)**

Chinese Character String

- **pf001 : Understand Directions or Not**

	No	%
1 YES	341	93.68
5 NO	23	6.32
Total	364	100.00

- **qf001_1 : Others**

Chinese Character String

- **qf001s1 : Why didn't Complete**

	No	%
1 R felt it would not be safe	12	100.00
Total	12	100.00

- **qf001s2 : Why didn't Complete**

	No	%
2 IWER felt it would not be safe	16	100.00
Total	16	100.00

- **qf001s3 : Why didn't Complete**

	No	%
3 R refused or was not willing to complete the test	4	100.00
Total	4	100.00

- **qf001s4 : Why didn't Complete**

	No	%
4 R tried but was unable to complete test	16	100.00
Total	16	100.00

- **qf001s5 : Why didn't Complete**

	No	%
5 R did not understand the instructions	2	100.00
Total	2	100.00

- **qf001s6 : Why didn't Complete**

	No	%
6 R had surgery, injury or other health condition that prevented R from standing	46	100.00
Total	46	100.00

- **qf001s7 : Why didn't Complete**

No Observations

- **qf001s8 : Why didn't Complete**

No Observations

- **qf001s97 : Why didn't Complete**

	No	%
97 Other Specify	6	100.00
Total	6	100.00

- **qf002 : How Complete**

	No	%
1 YES	263	77.13
5 NO Enter amount of time R held stand in seconds to two decimal places 993 R tried but was unable	15	4.40
993 R tried but was unable	33	9.68
999 R chose not to do it	30	8.80
Total	341	100.0

- **qf003 : Seconds**

Mean	Min	Max	OBS
3.9	0.0	9.0	15

- **qf004 : Use Any Compensatory Movements**

	No	%
1 YES	45	16.19
5 NO	233	83.81
Total	278	100.00

- **qf005 : Type of Floor Surface**

	No	%
1 Linoleum/tile/wood	97	34.89
2 carpet	1	0.36
3 Clay	19	6.83
4 Concrete	148	53.24
5 Not sure	1	0.36
97 Other Specify	12	4.32
Total	278	100.00

- **qf005_1 : Other (Specify)**

Chinese Character String

- **qf006 : How Compliant**

	No	%
1 R was fully compliant	12,990	99.46
2 R was prevented from fully complying due to illness, pain, or other symptoms or discomforts	53	0.41
3 R did not appear to be fully compliant, but no obvious reason for this	18	0.14
Total	13,061	100.00

- **pg001 : R is Eligible or Not**

	No	%
1 Yes, R Older Than 60	6,281	100.00
Total	6,281	100.00

- **pg002 : Safe or Not**

	No	%
1 No apparent restriction	5,917	94.35
2 Yes, recent surgery	27	0.43
3 Yes, injury	36	0.57
4 Yes, other health condition	291	4.64
Total	6,271	100.00

- **pg003 : Understand Directions or Not**

	No	%
1 YES	5,831	98.38
5 NO	96	1.62
Total	5,927	100.00

- **qg001_1 : Other (Specify)**

Chinese Character String

- **qg001s1 : Why didn't Complete**

	No	%
1 R felt it would not be safe	65	100.00
Total	65	100.00

- **qg001s2 : Why didn't Complete**

	No	%
2 IWER felt it would not be safe	110	100.00
Total	110	100.00

- **qg001s3 : Why didn't Complete**

	No	%
3 R refused or was not willing to complete the test	35	100.00
Total	35	100.00

- **qg001s4 : Why didn't Complete**

	No	%
4 R tried but was unable to complete test	25	100.00
Total	25	100.00

- **qg001s5 : Why didn't Complete**

	No	%
5 R did not understand the instructions	5	100.00
Total	5	100.00

- **qg001s6 : Why didn't Complete**

	No	%
6 R had surgery, injury, or other health condition that prevented R from walking	196	100.00
Total	196	100.00

- **qg001s7 : Why didn't Complete**

	No	%
7 No suitable space	95	100.00
Total	95	100.00

- **qg001s8 : Why didn't Complete**

	No	%
8 Problem with equipment or supplies	1	100.00
Total	1	100.00

- **qg001s97 : Why didn't Complete**

	No	%
97 Other Specify	32	100.00
Total	32	100.00

- **qg002 : Walking Speed Time-1**

Mean	Min	Max	OBS
6.1	0.0	999.0	5,779

- **qg002_1 : Repeat the Measurement**

	No	%
1	5,828	100.00
Total	5,828	100.00

- **qg003 : Walking Speed Time-2**

Mean	Min	Max	OBS
5.3	0.0	993.0	5,774

- **qg004 : Type of the Floor Surface**

	No	%
1 Linoleum/tile/wood	2,028	34.82
2 carpet	12	0.21
3 Clay	377	6.47
4 Concrete	3,220	55.29
5 Not sure	24	0.41
97 Other Specify	163	2.80
Total	5,824	100.00

- **qg004_1 : Other (Specify)**

Chinese Character String

- **qg005 : Aid Used**

	No	%
1 None	5,745	98.66
2 Walking stick or cane	64	1.10
3 Elbow crutches	1	0.02
4 Walking frame	1	0.02
97 Other Specify:	12	0.21
Total	5,823	100.00

- **qg006 : How Compliant**

	No	%
1 R was fully compliant	5,801	99.62
2 R was prevented from fully complying due to illness, pain, or other symptoms or discomforts	16	0.27

3 R was not fully compliant, but no obvious reason for this	6	0.10
Total	5,823	100.00

- **ph001 : Understand Or Not**

	No	%
1 Yes	13,228	95.26
5 No	658	4.74
Total	13,886	100.00

- **qh001_1 : Other (Specify)**

Chinese Character String

- **qh001s1 : Why didn't Complete**

	No	%
1 R felt it would not be safe	196	100.00
Total	196	100.00

- **qh001s2 : Why didn't Complete**

	No	%
2 IWER felt it would not be safe	262	100.00
Total	262	100.00

- **qh001s3 : Why didn't Complete**

	No	%
3 R refused or was not willing to complete the test	97	100.00
Total	97	100.00

- **qh001s4 : Why didn't Complete**

	No	%
4 R tried but was unable to complete test	99	100.00
Total	99	100.00

- **qh001s5 : Why didn't Complete**

	No	%
5 R did not understand the instructions	8	100.00
Total	8	100.00

- **qh001s6 : Why didn't Complete**

	No	%
6 R had surgery, injury or other health condition that prevented R from standing	439	100.00
Total	439	100.00

- **qh001s7 : Why didn't Complete**

	No	%
7 No suitable space	11	100.00
Total	11	100.00

- **qh001s8 : Why didn't Complete**

	No	%
8 Problem with equipment or supplies	3	100.00
Total	3	100.00

- **qh001s97 : Why didn't Complete**

	No	%
97 Other Specify	66	100.00
Total	66	100.00

- **qh002 : Did R Can Stand Up Straight**

	No	%
1 Yes Enter amount of time R held stand in seconds to two decimal places: Sec	12,937	97.71
5 No Enter amount of time R held stand in seconds to two decimal places: Sec	48	0.36
993 R tried but was unable	135	1.02
999 R chose not to do it	120	0.91
Total	13,240	100.00

- **qh003 : Seconds**

Mean	Min	Max	OBS

10.8	0.1	99.8	12,908

- **qh004 : Seconds**

Mean	Min	Max	OBS
12.9	2.5	45.2	47

- **qh005 : Times**

	No	%
0	1	2.04
1	8	16.33
2	9	18.37
3	18	36.73
4	13	26.53
Total	49	100.00

- **qh006 : Height**

Mean	Min	Max	OBS
43.7	0.4	452.0	12,960

- **qh007 : Use His/Her Trunk Arms**

	No	%
1 YES	1,525	11.75
5 NO	11,442	88.16
8 Dont Know	12	0.09
Total	12,979	100.00

- **pi001 : Understand Or Not**

	No	%
1 YES	13,762	99.08
5 NO	128	0.92
Total	13,890	100.00

- **qi001_1 : Other (Specify)**

Chinese Character String

- **qi001s1 : Why didn't Complete**

	No	%
1 R felt it would not be safe	16	100.00
Total	16	100.00

- **qi001s2 : Why didn't Complete**

	No	%
2 IWER felt it would not be safe	30	100.00
Total	30	100.00

- **qi001s3 : Why didn't Complete**

	No	%
3 R refused or was not willing to complete the test	22	100.00
Total	22	100.00

- **qi001s4 : Why didn't Complete**

	No	%
4 R tried but was unable to complete test	6	100.00
Total	6	100.00

- **qi001s5 : Why didn't Complete**

No Observations

- **qi001s6 : Why didn't Complete**

	No	%
6 R had humpback, cannot stand, etc. other health reasons	100	100.00
Total	100	100.00

- **qi001s7 : Why didn't Complete**

	No	%

7 No suitable space	4	100.00
Total	4	100.00

- **qi001s8 : Why didn't Complete**

	No	%
8 Problem with equipment or supplies	1	100.00
Total	1	100.00

- **qi001s97 : Why didn't Complete**

	No	%
97 Other Specify	10	100.00
Total	10	100.00

- **qi002 : Height**

Mean	Min	Max	OBS
158.5	1.4	993.0	13,709

- **qi003 : How Compliant**

	No	%
1 R was fully compliant	13,684	99.51
2 R was prevented from fully complying due to illness, pain, or other symptoms or discomforts	49	0.36
3 R was not fully compliant, but no obvious reason for this	18	0.13
Total	13,751	100.00

- **pj001 : Understand Or Not**

	No	%
1 YES	13,838	99.63
5 NO	51	0.37
Total	13,889	100.00

- **qj001_1 : Other (Specify)**

Chinese Character String

- **qj001s1 : Why didn't Complete**

	No	%
1 R felt it would not be safe	3	100.00
Total	3	100.00

- **qj001s2 : Why didn't Complete**

	No	%
2 IWER felt it would not be safe	6	100.00
Total	6	100.00

- **qj001s3 : Why didn't Complete**

	No	%
3 R refused or was not willing to complete the test	31	100.00
Total	31	100.00

- **qj001s4 : Why didn't Complete**

	No	%
4 R tried but was unable to complete test	2	100.00
Total	2	100.00

- **qj001s5 : Why didn't Complete**

No Observations

- **qj001s6 : Why didn't Complete**

	No	%
6 R could not participated due to health reasons	16	100.00
Total	16	100.00

- **qj001s7 : Why didn't Complete**

No Observations

- **qj001s8 : Why didn't Complete**

	No	%
8 Problem with equipment or supplies	2	100.00
Total	2	100.00

- **qj001s97 : Why didn't Complete**

	No	%
97 Other Specify	1	100.00
Total	1	100.00

- **qj002 : Arm Length**

Mean	Min	Max	OBS
34.0	0.0	999.0	13,780

- **qj003 : Which Arm**

	No	%
1 Left	4,627	33.47
2 Right	9,199	66.53
Total	13,826	100.00

- **qj004 : How Compliant**

	No	%
1 R was fully compliant	13,806	99.80
2 R was prevented from fully complying due to illness, pain, or other symptoms or discomforts	22	0.16
3 R was not fully compliant, but no obvious reas	6	0.04
Total	13,834	100.00

- **pk001 : Understand Directions or Not**

	No	%
1 YES	13,819	99.50
5 NO	69	0.50
Total	13,888	100.00

- **qk001_1 : Other (Specify)**

Chinese Character String

- **qk001s1 : Why didn't Complete**

	No	%
1 R felt it would not be safe	6	100.00
Total	6	100.00

- **qk001s2 : Why didn't Complete**

	No	%
2 IWER felt it would not be safe	11	100.00
Total	11	100.00

- **qk001s3 : Why didn't Complete**

	No	%
3 R refused or was not willing to complete the test	33	100.00
Total	33	100.00

- **qk001s4 : Why didn't Complete**

	No	%
4 R tried but was unable to complete test	3	100.00
Total	3	100.00

- **qk001s5 : Why didn't Complete**

No Observations

- **qk001s6 : Why didn't Complete**

	No	%
6 R could not participated due to health reasons	27	100.00
Total	27	100.00

- **qk001s7 : Why didn't Complete**

No Observations

- **qk001s8 : Why didn't Complete**

	No	%
8 Problem with equipment or supplies	1	100.00
Total	1	100.00

- **qk001s97 : Why didn't Complete**

	No	%
97 Other Specify	3	100.00
Total	3	100.00

- **qk002 : Knee Height**

Mean	Min	Max	OBS
48.0	0.0	993.0	13,743

- **qk003 : Which Knee**

	No	%
1 Left	13,808	100.00
Total	13,808	100.00

- **qk004 : How Compliant**

	No	%
1 R was fully compliant	13,770	99.65
2 R was prevented from fully complying due to illness, pain, or other symptoms or discomforts	41	0.30
3 R was not fully compliant, but no obvious reason for this	7	0.05
Total	13,818	100.00

- **pl001 : Eligible for Weight Measurement**

	No	%
1 Yes	13,876	99.95

	7	0.05
Total	13,883	100.00

- **pl002 : Understand Directions or Not**

	No	%
1 YES	13,737	98.94
5 NO	147	1.06
Total	13,884	100.00

- **ql001_1 : Other (Specify)**

Chinese Character String

- **ql001s1 : Why didn't Complete**

	No	%
1 R felt it would not be safe	12	100.00
Total	12	100.00

- **ql001s2 : Why didn't Complete**

	No	%
2 IWER felt it would not be safe	26	100.00
Total	26	100.00

- **ql001s3 : Why didn't Complete**

	No	%
3 R refused or was not willing to complete the test	24	100.00
Total	24	100.00

- **ql001s4 : Why didn't Complete**

	No	%
4 R tried but was unable to complete test	9	100.00
Total	9	100.00

- **ql001s5 : Why didn't Complete**

No Observations

- **ql001s6 : Why didn't Complete**

	No	%
6 R is not able to stand or balance on scale; other health reason	76	100.00
Total	76	100.00

- **ql001s7 : Why didn't Complete**

	No	%
7 No suitable space	13	100.00
Total	13	100.00

- **ql001s8 : Why didn't Complete**

	No	%
8 Problem with equipment or supplies	23	100.00
Total	23	100.00

- **ql001s97 : Why didn't Complete**

	No	%
97 Other Specify	12	100.00
Total	12	100.00

- **ql002 : Weight Measurement**

Mean	Min	Max	OBS
58.8	0.0	162.0	13,728

- **ql003 : Type of Floor Surface**

	No	%
1 Linoleum/tile/wood	5,865	42.69
2 carpet	8	0.06
3 Clay	484	3.52
4 Concrete	7,066	51.44

	No	%
5 Not sure	42	0.31
97 Other Specify:	272	1.98
Total	13,737	100.00

- **ql003_1 : Other (Specify)**

Chinese Character String

- **ql004 : Wearing Shoes or Not**

	No	%
1 YES	1,376	10.02
5 NO	12,352	89.98
Total	13,728	100.00

- **ql005 : How Compliant**

	No	%
1 R was fully compliant	13,675	99.56
2 R was prevented from fully complying due to illness, pain, or other symptoms or discomforts	38	0.28
3 R was not fully compliant, but no obvious reason for this	22	0.16
Total	13,735	100.00

- **pm001 : Stand or Not**

	No	%
1 YES	13,798	99.36
5 NO	89	0.64
Total	13,887	100.00

- **pm002 : Understand Or Not**

	No	%
1 YES	13,798	99.99
5 NO	2	0.01
Total	13,800	100.00

- **qm001_1 : Other (Specify)**

Chinese Character String

- **qm001s1 : Why didn't Complete**

	No	%
1 R felt it would not be safe	8	100.00
Total	8	100.00

- **qm001s2 : Why didn't Complete**

	No	%
2 IWER felt it would not be safe	16	100.00
Total	16	100.00

- **qm001s3 : Why didn't Complete**

	No	%
3 R refused or was not willing to complete the test	34	100.00
Total	34	100.00

- **qm001s4 : Why didn't Complete**

	No	%
4 R tried but was unable to complete test	4	100.00
Total	4	100.00

- **qm001s5 : Why didn't Complete**

	No	%
5 R did not understand the instructions	3	100.00
Total	3	100.00

- **qm001s6 : Why didn't Complete**

	No	%
6 R is not able to stnd; other health reason	46	100.00
Total	46	100.00

- **qm001s7 : Why didn't Complete**

No Observations

- **qm001s8 : Why didn't Complete**

No Observations

- **qm001s97 : Why didn't Complete**

	No	%
97 Other Specify	11	100.00
Total	11	100.00

- **qm002 : Waist Measurement**

Mean	Min	Max	OBS
84.4	8.4	999.0	13,771

- **qm003_1 : Other (Specify)**

Chinese Character String

- **qm003s1 : Difficulties Occurred**

	No	%
1 None	13,534	100.00
Total	13,534	100.00

- **qm003s2 : Difficulties Occurred**

	No	%
2 R had breathing difficulties	58	100.00
Total	58	100.00

- **qm003s3 : Difficulties Occurred**

	No	%
3 R was unable to hold breath at the end of the exhale	48	100.00
Total	48	100.00

- **qm003s4 : Difficulties Occurred**

	No	%
4 R was prevented from giving full effort by illness, pain, or other symptoms or discomforts	151	100.00
Total	151	100.00

- **qm003s5 : Difficulties Occurred**

	No	%
5 R did not appear to give full effort, but no obvious reason for this	18	100.00
Total	18	100.00

- **qm003s6 : Difficulties Occurred**

	No	%
6 Had difficulty or unable to locate navel	10	100.00
Total	10	100.00

- **qm003s97 : Difficulties Occurred**

	No	%
97 Other Specify:	17	100.00
Total	17	100.00

- **qm004 : How Compliant**

	No	%
1 R was fully compliant	13,702	99.33
2 R was prevented from fully complying due to illness, pain, or other symptoms or discomforts	70	0.51
3 R was not fully compliant, but no obvious reason for this	22	0.16
Total	13,794	100.00

- **qm005 : Wearing Bulky Clothing During Measurement**

	No	%
1 YES	513	3.72

5 NO	13,273	96.28
Total	13,786	100.00

13 Community

- **communityID : Community ID**

A String Variable	
OBS:	454

- **sub_commuID : Sub Community ID**

A String Variable	
OBS:	6

- **ja001 : Community or Village**

	No	%
1 Village	301	66.45
2 Community	146	32.23
3 Both village and community	6	1.32
Total	453	100.00

- **ja002 : Year**

Mean	Min	Max	OBS
1,987.1	1,930.0	2,011.0	440

- **ja003 : Square of Kilometers or Mu**

	No	%
1 Square of Kilometers	235	54.52
2 Mu	196	45.48
Total	431	100.00

- **ja003_1 : Square of Kilometers**

Mean	Min	Max	OBS
2,320.8	0.0	540,000.0	238

- **ja003_2 : Mu**

Mean	Min	Max	OBS

7,989.3	1.0	137,000.0	198

- **ja004 : Has the Boundary of this V/C Even Changed**

	No	%
1 Yes	81	17.92
2 No	371	82.08
Total	452	100.00

- **ja005 : Year**

Mean	Min	Max	OBS
2,000.3	1,960.0	2,011.0	88

- **ja006 : Square of Kilometers or Mu**

	No	%
1 Square of Kilometers	41	69.49
2 Mu	18	30.51
Total	59	100.00

- **ja006_1 : Square of Kilometers**

Mean	Min	Max	OBS
496.6	0.0	20,000.0	45

- **ja006_2 : Mu**

Mean	Min	Max	OBS
4,555.7	800.0	16,400.0	19

- **ja007 : Was Changed to Community from Village**

	No	%
1 Yes	32	20.92
2 No	121	79.08
Total	153	100.00

- **ja008 : Year**

Mean	Min	Max	OBS
2,001.2	1,980.0	2,011.0	33

- **ja009 : Has Your Village Been Designated into the City Planning Area**

	No	%
1 Yes	66	21.64
2 No	239	78.36
Total	305	100.00

- **ja010 : Year**

Mean	Min	Max	OBS
2,004.5	1,985.0	2,011.0	66

- **ja011 : Mu of Arable Land**

Mean	Min	Max	OBS
3,708.1	0.0	96,000.0	306

- **ja012 : Among Arable Land, How Many is Irrigatable**

Mean	Min	Max	OBS
1,207.6	0.0	19,000.0	306

- **ja013 : How Many Arable Land at the End of 2007**

Mean	Min	Max	OBS
3,447.7	0.0	96,000.0	306

- **ja014 : How Many Construction Land at the End of 2010**

Mean	Min	Max	OBS
774.0	1.0	37,500.0	307

- **ja015 : Carried Out Property Rights Delineation and Land Titling in the Recent 5 Years**

	No	%
1 Yes	94	30.82
2 No	211	69.18
Total	305	100.00

- **ja016 : Year**

Mean	Min	Max	OBS
2,007.9	2,006.0	2,011.0	100

- **ja017 : Receiving Contract**

	No	%
1 Yes	233	76.39
2 No	72	23.61
Total	305	100.00

- **ja018 : Year**

Mean	Min	Max	OBS
1,999.6	1,978.0	2,011.0	237

- **ja019 : Receiving Contract**

	No	%
1 Yes	197	64.59
2 No	108	35.41
Total	305	100.00

- **ja020 : Year**

Mean	Min	Max	OBS
1,997.3	1,978.0	2,011.0	195

- **ja021 : Certificate for Housing Ownership**

	No	%
1 Yes	148	48.52
2 No	157	51.48
Total	305	100.00

- **ja022 : Year**

Mean	Min	Max	OBS
1,997.3	1,978.0	2,011.0	152

- **ja023 : Forest Rights Certificate**

	No	%
1 Yes	164	53.42
2 No	66	21.50
3 Not applicable	77	25.08
Total	307	100.00

- **ja024 : Year**

Mean	Min	Max	OBS
2,004.5	1,980.0	2,011.0	168

- **ja025 : Certificate for Collective Assets**

	No	%
1 Yes	18	5.90
2 No	287	94.10
Total	305	100.00

- **ja026 : Year**

Mean	Min	Max	OBS
2,000.4	1,980.0	2,011.0	18

- **ja027 : Land Reallocation Since 2005**

	No	%
1 Yes	57	18.63
2 No	249	81.37
Total	306	100.00

- **ja028 : Year**

Mean	Min	Max	OBS

2,008.0	2,005.0	2,011.0	57

- **ja029 : Num. of New Arable Land**

Mean	Min	Max	OBS
140.5	-17.0	1,000.0	56

- **ja030 : Construction Land Reallocation since 2005**

	No	%
1 Yes	36	11.76
2 No	270	88.24
Total	306	100.00

- **ja031 : Year**

Mean	Min	Max	OBS
2,008.3	2,005.0	2,011.0	39

- **ja032 : Decreased Construction Land**

Mean	Min	Max	OBS
79.3	0.0	800.0	36

- **ja033 : Percentage of Rental Land**

Mean	Min	Max	OBS
13.7	0.0	100.0	302

- **ja034 : Rental Price/Mu**

Mean	Min	Max	OBS
526.4	-999.0	32,000.0	294

- **ja035 : Land Procurement**

	No	%
1 Yes	144	31.79
2 No	309	68.21

Total	453	100.00

- **ja036 : Year**

Mean	Min	Max	OBS
2,007.5	2,000.0	2,011.0	145

- **ja037 : Compensation Amount/Mu**

Mean	Min	Max	OBS
9,545.5	0.0	85,000.0	136

- **ja038 : Type of Main Landscape of Your V/C**

	No	%
1 Plain	207	45.70
2 Hill	122	26.93
3 Mountainous Region	93	20.53
4 Plateau	20	4.42
5 Basin	11	2.43
Total	453	100.00

- **ja039_1 : Minimum Temperature**

Mean	Min	Max	OBS
-8.3	-60.0	35.0	449

- **ja039_2 : Maximum Temperature**

Mean	Min	Max	OBS
37.1	22.0	43.0	452

- **ja040 : How Many Rainy Days in the Past Year**

Mean	Min	Max	OBS
52.4	0.0	298.0	440

- **ja041 : How Many Snowy Days**

Mean	Min	Max	OBS
8.1	0.0	150.0	445

- **ja042_1_1_ : Education Level**

	No	%
1 Primary school	2	1.32
2 Middle school	7	4.61
3 High school	40	26.32
4 Middle professional school	12	7.89
5 Advanced professional school	65	42.76
6 University and above	26	17.11
Total	152	100.00

- **ja042_1_2_ : Education Level**

	No	%
1 Primary school	12	3.99
2 Middle school	121	40.20
3 High school	96	31.89
4 Middle professional school	28	9.30
5 Advanced professional school	41	13.62
6 University and above	2	0.66
7 Illiterate	1	0.33
Total	301	100.00

- **ja042_1_3_ : Education Level**

	No	%
1 Primary school	15	4.97
2 Middle school	92	30.46
3 High school	110	36.42
4 Middle professional school	23	7.62
5 Advanced professional school	54	17.88
6 University and above	8	2.65
Total	302	100.00

- **ja042_1_4_ : Education Level**

	No	%
1 Primary school	12	4.05
2 Middle school	94	31.76
3 High school	123	41.55
4 Middle professional school	38	12.84

5 Advanced professional school	23	7.77
6 University and above	6	2.03
Total	296	100.00

- **ja042_2_1_ : Age**

	No	%
24	1	0.66
26	1	0.66
28	1	0.66
30	3	1.97
32	4	2.63
33	3	1.97
34	2	1.32
35	4	2.63
36	7	4.61
37	10	6.58
38	4	2.63
39	3	1.97
40	3	1.97
41	8	5.26
42	3	1.97
43	4	2.63
44	4	2.63
45	9	5.92
46	4	2.63
47	9	5.92
48	8	5.26
49	9	5.92
50	5	3.29
51	2	1.32
52	8	5.26
53	7	4.61
54	3	1.97
55	5	3.29
56	5	3.29
57	2	1.32
58	3	1.97
59	3	1.97
60	2	1.32
62	1	0.66
66	1	0.66
72	1	0.66
Total	152	100.00

- **ja042_2_2_ : Age**

	No	%
26	1	0.33

29	1	0.33
30	2	0.67
32	4	1.33
34	5	1.67
35	7	2.33
36	6	2.00
37	3	1.00
38	9	3.00
39	5	1.67
40	12	4.00
41	8	2.67
42	11	3.67
43	14	4.67
44	5	1.67
45	14	4.67
46	22	7.33
47	18	6.00
48	24	8.00
49	19	6.33
50	18	6.00
51	14	4.67
52	10	3.33
53	11	3.67
54	9	3.00
55	11	3.67
56	7	2.33
57	10	3.33
58	2	0.67
59	5	1.67
60	5	1.67
61	2	0.67
62	2	0.67
65	3	1.00
70	1	0.33
Total	300	100.00

- **ja042_2_3_ : Age**

	No	%
26	2	0.66
29	1	0.33
30	1	0.33
32	2	0.66
33	2	0.66
34	1	0.33
35	5	1.66
36	9	2.98
37	3	0.99
38	5	1.66
39	8	2.65
40	8	2.65

41	8	2.65
42	8	2.65
43	16	5.30
44	5	1.66
45	18	5.96
46	18	5.96
47	15	4.97
48	21	6.95
49	20	6.62
50	13	4.30
51	13	4.30
52	13	4.30
53	11	3.64
54	14	4.64
55	11	3.64
56	8	2.65
57	9	2.98
58	8	2.65
59	5	1.66
60	5	1.66
61	3	0.99
62	3	0.99
63	2	0.66
65	3	0.99
66	1	0.33
67	2	0.66
68	1	0.33
75	1	0.33
Total	302	100.00

- **ja042_2_4_ : Age**

	No	%
23	1	0.34
24	1	0.34
25	2	0.68
26	2	0.68
27	3	1.02
29	1	0.34
30	1	0.34
31	4	1.36
32	5	1.70
33	1	0.34
34	1	0.34
35	4	1.36
36	4	1.36
37	2	0.68
38	5	1.70
39	10	3.40
40	13	4.42
41	8	2.72

42	10	3.40
43	10	3.40
44	5	1.70
45	6	2.04
46	8	2.72
47	17	5.78
48	16	5.44
49	16	5.44
50	10	3.40
51	10	3.40
52	10	3.40
53	11	3.74
54	7	2.38
55	20	6.80
56	11	3.74
57	7	2.38
58	13	4.42
59	5	1.70
60	11	3.74
61	4	1.36
62	4	1.36
63	5	1.70
65	2	0.68
66	2	0.68
67	3	1.02
70	2	0.68
100	1	0.34
Total	294	100.00

- **ja042_2_5_ : Age**

	No	%
47	1	100.00
Total	1	100.00

- **ja042_3_1_ : How Long did He/She Hold this Position**

	No	%
0	1	0.66
1	15	9.93
2	9	5.96
3	21	13.91
4	7	4.64
5	13	8.61
6	12	7.95
7	3	1.99
8	9	5.96
9	11	7.28
10	17	11.26

11	4	2.65
12	6	3.97
13	5	3.31
14	5	3.31
15	3	1.99
16	1	0.66
17	1	0.66
18	2	1.32
20	1	0.66
22	1	0.66
24	1	0.66
27	2	1.32
45	1	0.66
Total	151	100.00

- **ja042_3_2_ : How Long did He/She Hold this Position**

	No	%
0	2	0.67
1	39	13.00
2	20	6.67
3	35	11.67
4	25	8.33
5	16	5.33
6	31	10.33
7	13	4.33
8	15	5.00
9	20	6.67
10	23	7.67
11	3	1.00
12	13	4.33
13	8	2.67
14	6	2.00
15	5	1.67
16	4	1.33
17	2	0.67
18	3	1.00
19	2	0.67
20	6	2.00
21	1	0.33
22	2	0.67
24	1	0.33
25	1	0.33
26	1	0.33
27	1	0.33
30	1	0.33
35	1	0.33
Total	300	100.00

- **ja042_3_3_ : How Long did He/She Hold this Position**

	No	%
0	1	0.33
1	20	6.64
2	24	7.97
3	28	9.30
4	20	6.64
5	11	3.65
6	27	8.97
7	17	5.65
8	17	5.65
9	16	5.32
10	25	8.31
11	9	2.99
12	10	3.32
13	11	3.65
14	11	3.65
15	15	4.98
16	5	1.66
17	3	1.00
18	4	1.33
19	2	0.66
20	4	1.33
21	3	1.00
22	3	1.00
23	1	0.33
24	1	0.33
25	3	1.00
26	2	0.66
27	2	0.66
29	1	0.33
30	2	0.66
36	1	0.33
37	1	0.33
40	1	0.33
Total	301	100.00

- **ja042_3_4_ : How Long did He/She Hold this Position**

	No	%
1	28	9.49
2	21	7.12
3	20	6.78
4	15	5.08
5	13	4.41
6	23	7.80
7	13	4.41
8	12	4.07
9	12	4.07
10	21	7.12
11	4	1.36

12	13	4.41
13	9	3.05
14	6	2.03
15	13	4.41
16	7	2.37
17	3	1.02
18	7	2.37
19	2	0.68
20	8	2.71
21	4	1.36
22	2	0.68
23	4	1.36
24	4	1.36
25	5	1.69
26	5	1.69
27	5	1.69
30	5	1.69
31	1	0.34
34	2	0.68
35	1	0.34
37	2	0.68
38	1	0.34
40	1	0.34
42	2	0.68
45	1	0.34
Total	295	100.00

- **ja042_3_5_ : How Long did He/She Hold this Position**

	No	%
9	1	100.00
Total	1	100.00

- **ja043 : Does Your Village has Large Surnames**

	No	%
1 Yes	244	80.00
2 No	61	20.00
Total	305	100.00

- **ja044 : How Many Large Surnames**

	No	%
1 one	88	35.92
2 two	90	36.73
3 three	55	22.45
4 more than three	12	4.90

Total	245	100.00

- **ja045_1 : The 1st Largest Surname**

Chinese Character String

- **ja045_2 : The 2nd Largest Surname**

Chinese Character String

- **ja045_3 : The 3rd Largest Surname**

Chinese Character String

- **ja046_1 : Num. of Households with 1st Largest Surname**

Mean	Min	Max	OBS
247.0	16.0	1,800.0	238

- **ja046_2 : Num. of Households with 2nd Largest Surname**

Mean	Min	Max	OBS
136.4	15.0	800.0	153

- **ja046_3 : Num. of Households with 3rd Largest Surname**

Mean	Min	Max	OBS
117.8	13.0	475.0	65

- **ja048 : Respondent Identity for Part A**

	No	%
1 Village head	76	16.78
2 Village secretary	67	14.79
3 Village accountant	93	20.53
4 Director of the street committee	61	13.47

5 Others	156	34.44
Total	453	100.00

- **ja050 : Average Area of House Site Per Person**

Mean	Min	Max	OBS
17.2	0.0	2,310.0	289

- **ja051s1 : The Condition that the Household May Get New House Site**

	No	%
1 Single adult son	141	100.00
Total	141	100.00

- **ja051s2 : The Condition that the Household May Get New House Site**

	No	%
2 Married son with a wife coming to his family	205	100.00
Total	205	100.00

- **ja051s3 : The Condition that the Household May Get New House Site**

	No	%
3 Married son going to the wifes family	45	100.00
Total	45	100.00

- **ja051s4 : The Condition that the Household May Get New House Site**

	No	%
4 Single adult daughter	45	100.00
Total	45	100.00

- **ja051s5 : The Condition that the Household May Get New House Site**

	No	%
5 Married daughter going to her husbands family	33	100.00
Total	33	100.00

- **ja051s6 : The Condition that the Household May Get New House Site**

	No	%
6 Married daughter with a husband coming to her family	130	100.00
Total	130	100.00

- **ja052 : Source of House Site**

	No	%
1 Private plots of the household	138	46.62
2 Contracted field of the household	46	15.54
3 Road-side public land	28	9.46
4 Others	84	28.38
Total	296	100.00

- **ja052_1 : Other Source**

Chinese Character String

- **ja053 : Have the Rules of House Site Allocation Been Changed Since 2000**

	No	%
1 Yes	17	5.57
2 No	288	94.43
Total	305	100.00

- **ja054 : Year**

Mean	Min	Max	OBS
2,005.0	2,000.0	2,011.0	16

- **jb001 : Main Type of Roads**

	No	%
1 Paved road	324	71.37
2 Pathway/Dirt/unpaved road	88	19.38
3 Sand-stone road	40	8.81
5 Other	2	0.44
Total	454	100.00

- **jb002 : How Many Days Were the Roads Not Passable Last Year**

Mean	Min	Max	OBS
30.7	0.0	366.0	447

- **jb003 : How Many Bus Lines Run through this V/C**

Mean	Min	Max	OBS
-4.1	-999.0	32.0	449

- **jb004 : How Far from the V/C Office to the Most Commonly Used Bus Stop**

Mean	Min	Max	OBS
3.0	0.0	100.0	446

- **jb005 : How Far from the V/C Office to the Most Commonly Used Train Station**

Mean	Min	Max	OBS
50.5	-999.0	999.0	445

- **jb006_1 : Tap Water**

Mean	Min	Max	OBS
1,002.3	0.0	20,000.0	434

- **jb006_2 : Well Water**

Mean	Min	Max	OBS
277.9	0.0	3,000.0	423

- **jb006_3 : Pool Water**

Mean	Min	Max	OBS
32.3	0.0	999.0	412

- **jb006_4 : River, Lakes and Brooks Water**

Mean	Min	Max	OBS
39.7	0.0	1,200.0	410

- **jb006_5 : Rain or Snow Water**

Mean	Min	Max	OBS
37.8	0.0	1,200.0	413

- **jb006_6 : Cellar Water**

Mean	Min	Max	OBS
34.9	0.0	999.0	413

- **jb006_7 : Spring**

Mean	Min	Max	OBS
69.5	0.0	2,500.0	415

- **jb006_8 : Other**

Mean	Min	Max	OBS
41.6	0.0	999.0	402

- **jb007_1 : Hay**

Mean	Min	Max	OBS
236.5	0.0	2,500.0	434

- **jb007_2 : Coal**

Mean	Min	Max	OBS
203.0	0.0	4,859.0	419

- **jb007_3 : Marsh Gas**

Mean	Min	Max	OBS
46.9	0.0	999.0	422

- **jb007_4 : Natural Gas**

Mean	Min	Max	OBS
406.1	0.0	6,494.0	424

- **jb007_5 : Liquefied Petroleum Gas**

Mean	Min	Max	OBS
565.9	0.0	19,000.0	434

- **jb007_6 : Other**

Mean	Min	Max	OBS
91.9	0.0	3,500.0	413

- **jb008 : Does Your V/C Have Heating System for Winter**

	No	%
1 Yes	56	12.33
2 No	398	87.67
Total	454	100.00

- **jb009_1 : other**

Chinese Character String

- **jb009s1 : Which Kind of Heating System**

	No	%
1 Electronic heating	4	100.00
Total	4	100.00

- **jb009s2 : Which Kind of Heating System**

	No	%
2 Coal heating	48	100.00
Total	48	100.00

- **jb009s3 : Which Kind of Heating System**

	No	%
3 Natural gas heating	7	100.00
Total	7	100.00

- **jb009s4 : Which Kind of Heating System**

	No	%
4 Others, specify	3	100.00
Total	3	100.00

- **jb010 : Any Sewer System in this V/C**

	No	%
1 Yes	180	39.65
2 No	274	60.35
Total	454	100.00

- **jb011 : Year**

Mean	Min	Max	OBS
1,994.9	1,949.0	2,008.0	161

- **jb012 : How is the Waste Being Managed**

	No	%
1 Moved away by truck	225	49.56
2 Buried in this village	24	5.29
3 Burn away	12	2.64
4 Put into nearby river	3	0.66
5 Do not manage	190	41.85
Total	454	100.00

- **jb013 : Main Type of Toilet**

	No	%
1 Inside toilet with water	196	43.17
2 Inside toilet without water	15	3.30
3 Outside toilet with water	18	3.96
4 Outside public toilet without water	41	9.03
5 Open-air	170	37.44

6 Others, please specify:	14	3.08
Total	454	100.00

- **jb013_1 : Others**

	No	%
.E	438	96.48
	1	0.22
	1	0.22
	1	0.22
	1	0.22
	1	0.22
	2	0.44
	1	0.22
	1	0.22
	1	0.22
	1	0.22
	1	0.22
/	1	0.22
	1	0.22
	1	0.22
	1	0.22
Total	454	100.00

- **jb014 : Have Public Restroom**

	No	%
1 Yes	191	42.07
2 No	263	57.93
Total	454	100.00

- **jb015 : Reconstructed the Toilet and Sewer System**

	No	%
1 Yes	139	44.84
2 No	171	55.16
Total	310	100.00

- **jb016 : Year**

Mean	Min	Max	OBS
2,005.0	1,970.0	2,011.0	139

- **jb017 : Percentage of Households Use Electricity**

Mean	Min	Max	OBS
99.7	50.0	100.0	452

- **jb018 : Degree of Industrial Pollution**

	No	%
1 Poor	12	2.64
2 Fair	61	13.44
3 Good	98	21.59
4 Very good	283	62.33
Total	454	100.00

- **jb019 : Degree of Nearby Industrial Pollution**

	No	%
1 Poor	18	3.97
2 Fair	107	23.62
3 Good	121	26.71
4 Very good	207	45.70
Total	453	100.00

- **jb020 : Num. of Days with Electricity Supply One Year**

Mean	Min	Max	OBS
356.9	0.0	366.0	453

- **jb021 : Num. of Hours with Electricity Supply Per Day**

Mean	Min	Max	OBS
23.6	8.0	24.0	453

- **jb022 : Year**

Mean	Min	Max	OBS
1,978.5	1,929.0	2,009.0	303

- **jb023 : Percentage of Households with Telephone**

Mean	Min	Max	OBS

42.1	0.0	100.0	309

- **jb024 : Percentage of Households with Cell Phone**

Mean	Min	Max	OBS
86.8	10.0	100.0	309

- **jb025 : Percentage of Households with TV**

	No	%
0	1	0.32
30	1	0.32
33	1	0.32
40	1	0.32
50	1	0.32
60	2	0.65
70	4	1.29
80	18	5.81
85	7	2.26
86	1	0.32
87	1	0.32
90	32	10.32
93	2	0.65
95	26	8.39
96	5	1.61
97	6	1.94
98	21	6.77
99	16	5.16
100	164	52.90
Total	310	100.00

- **jb026 : Percentage of Households with Refrigerator**

Mean	Min	Max	OBS
44.4	0.0	100.0	310

- **jb027 : How Far is the Nearest Industrial Pollution Site**

Mean	Min	Max	OBS
25.6	0.0	1,000.0	412

- **jb028_1_1_ : Num. of this Facility**

	No	%
0	203	45.01
1	156	34.59
2	44	9.76
3	19	4.21
4	10	2.22
5	8	1.77
6	2	0.44
7	2	0.44
8	2	0.44
10	5	1.11
Total	451	100.00

- **jb028_1_2_ : Num. of this Facility**

	No	%
0	186	41.33
1	228	50.67
2	21	4.67
3	10	2.22
4	4	0.89
6	1	0.22
Total	450	100.00

- **jb028_1_3_ : Num. of this Facility**

	No	%
0	369	82.18
1	70	15.59
2	9	2.00
4	1	0.22
Total	449	100.00

- **jb028_1_4_ : Num. of this Facility**

	No	%
0	416	92.65
1	30	6.68
2	3	0.67
Total	449	100.00

- **jb028_1_5_ : Num. of this Facility**

	No	%

0	363	80.67
1	79	17.56
2	7	1.56
3	1	0.22
Total	450	100.00

- **jb028_1_6_ : Num. of this Facility**

	No	%
0	207	45.90
1	223	49.45
2	15	3.33
3	3	0.67
4	1	0.22
5	2	0.44
Total	451	100.00

- **jb028_1_7_ : Num. of this Facility**

	No	%
0	232	51.33
1	203	44.91
2	9	1.99
3	2	0.44
4	2	0.44
5	2	0.44
6	1	0.22
15	1	0.22
Total	452	100.00

- **jb028_1_8_ : Num. of this Facility**

	No	%
0	301	66.89
1	62	13.78
2	40	8.89
3	24	5.33
4	14	3.11
5	2	0.44
6	3	0.67
7	2	0.44
8	1	0.22
9	1	0.22
Total	450	100.00

- **jb028_1_9_ : Num. of this Facility**

	No	%
0	422	93.99
1	25	5.57
2	1	0.22
3	1	0.22
Total	449	100.00

- **jb028_1_10_ : Num. of this Facility**

	No	%
0	388	86.80
1	51	11.41
2	6	1.34
5	1	0.22
11	1	0.22
Total	447	100.00

- **jb028_1_11_ : Num. of this Facility**

	No	%
0	35	7.92
1	34	7.69
2	43	9.73
3	35	7.92
4	38	8.60
5	46	10.41
6	23	5.20
7	14	3.17
8	17	3.85
9	5	1.13
10	29	6.56
11	3	0.68
12	6	1.36
13	1	0.23
14	1	0.23
15	15	3.39
17	2	0.45
18	5	1.13
20	19	4.30
21	2	0.45
25	3	0.68
26	2	0.45
27	2	0.45
28	2	0.45
30	13	2.94
32	3	0.68
35	3	0.68

40	6	1.36
41	1	0.23
45	3	0.68
50	10	2.26
57	1	0.23
60	2	0.45
61	1	0.23
70	1	0.23
75	1	0.23
80	2	0.45
89	1	0.23
98	1	0.23
99	1	0.23
100	10	2.26
Total	442	100.00

- **jb028_1_12_ : Num. of this Facility**

	No	%
0	300	66.82
1	129	28.73
2	13	2.90
3	5	1.11
5	1	0.22
6	1	0.22
Total	449	100.00

- **jb028_1_13_ : Num. of this Facility**

	No	%
0	274	61.02
1	66	14.70
2	40	8.91
3	24	5.35
4	9	2.00
5	14	3.12
6	5	1.11
7	1	0.22
8	6	1.34
10	4	0.89
11	1	0.22
13	1	0.22
18	1	0.22
28	1	0.22
30	1	0.22
87	1	0.22
Total	449	100.00

- **jb028_2_1_ : Distance**

Mean	Min	Max	OBS
2.6	0.0	140.0	445

- **jb028_2_2_ : Distance**

Mean	Min	Max	OBS
1.4	0.0	140.0	447

- **jb028_2_3_ : Distance**

Mean	Min	Max	OBS
4.2	0.0	140.0	442

- **jb028_2_4_ : Distance**

Mean	Min	Max	OBS
18.2	0.0	600.0	441

- **jb028_2_5_ : Distance**

Mean	Min	Max	OBS
5.8	0.0	500.0	443

- **jb028_2_6_ : Distance**

Mean	Min	Max	OBS
28.0	0.0	9,999.0	438

- **jb028_2_7_ : Distance**

Mean	Min	Max	OBS
2.9	0.0	35.0	449

- **jb028_2_8_ : Distance**

Mean	Min	Max	OBS

3.6	0.0	45.0	447

- **jb028_2_9_ : Distance**

Mean	Min	Max	OBS
21.4	0.0	999.0	435

- **jb028_2_10_ : Distance**

Mean	Min	Max	OBS
12.3	0.0	999.0	432

- **jb028_2_11_ : Distance**

Mean	Min	Max	OBS
0.2	0.0	11.0	445

- **jb028_2_12_ : Distance**

Mean	Min	Max	OBS
4.6	0.0	200.0	445

- **jb028_2_13_ : Distance**

Mean	Min	Max	OBS
5.5	0.0	193.0	445

- **jb029_1_1_ : Does this V/C Have this Type of Activity**

	No	%
1 Yes	195	43.05
2 No	258	56.95
Total	453	100.00

- **jb029_1_2_ : Does this V/C Have this Type of Activity**

	No	%
1 Yes	21	4.65
2 No	431	95.35

Total	452	100.00

- **jb029_1_3_ : Does this V/C Have this Type of Activity**

	No	%
1 Yes	165	36.50
2 No	287	63.50
Total	452	100.00

- **jb029_1_4_ : Does this V/C Have this Type of Activity**

	No	%
1 Yes	185	40.84
2 No	268	59.16
Total	453	100.00

- **jb029_1_5_ : Does this V/C Have this Type of Activity**

	No	%
1 Yes	189	41.91
2 No	262	58.09
Total	451	100.00

- **jb029_1_6_ : Does this V/C Have this Type of Activity**

	No	%
1 Yes	119	26.27
2 No	334	73.73
Total	453	100.00

- **jb029_1_7_ : Does this V/C Have this Type of Activity**

	No	%
1 Yes	49	10.86
2 No	402	89.14
Total	451	100.00

- **jb029_1_8_ : Does this V/C Have this Type of Activity**

	No	%
1 Yes	175	38.80

2 No	276	61.20
Total	451	100.00

- **jb029_1_9_ : Does this V/C Have this Type of Activity**

	No	%
1 Yes	170	37.69
2 No	281	62.31
Total	451	100.00

- **jb029_1_10_ : Does this V/C Have this Type of Activity**

	No	%
1 Yes	124	27.43
2 No	328	72.57
Total	452	100.00

- **jb029_1_11_ : Does this V/C Have this Type of Activity**

	No	%
1 Yes	183	40.40
2 No	270	59.60
Total	453	100.00

- **jb029_1_12_ : Does this V/C Have this Type of Activity**

	No	%
1 Yes	150	33.26
2 No	301	66.74
Total	451	100.00

- **jb029_1_13_ : Does this V/C Have this Type of Activity**

	No	%
1 Yes	51	11.28
2 No	401	88.72
Total	452	100.00

- **jb029_1_14_ : Does this V/C Have this Type of Activity**

	No	%

1 Yes	76	16.81
2 No	376	83.19
Total	452	100.00

- **jb029_2_1_ : Who Initiated This**

	No	%
1 Upper-level government	80	40.40
2 U/C government	78	39.39
3 U/C residents	9	4.55
4 Others	31	15.66
Total	198	100.00

- **jb029_2_2_ : Who Initiated This**

	No	%
1 Upper-level government	5	23.81
2 U/C government	4	19.05
3 U/C residents	2	9.52
4 Others	10	47.62
Total	21	100.00

- **jb029_2_3_ : Who Initiated This**

	No	%
1 Upper-level government	78	47.27
2 U/C government	61	36.97
3 U/C residents	8	4.85
4 Others	18	10.91
Total	165	100.00

- **jb029_2_4_ : Who Initiated This**

	No	%
1 Upper-level government	72	39.56
2 U/C government	75	41.21
3 U/C residents	12	6.59
4 Others	23	12.64
Total	182	100.00

- **jb029_2_5_ : Who Initiated This**

	No	%

1 Upper-level government	27	14.44
2 U/C government	85	45.45
3 U/C residents	64	34.22
4 Others	11	5.88
Total	187	100.00

- **jb029_2_6_ : Who Initiated This**

	No	%
1 Upper-level government	42	35.29
2 U/C government	63	52.94
3 U/C residents	5	4.20
4 Others	9	7.56
Total	119	100.00

- **jb029_2_7_ : Who Initiated This**

	No	%
1 Upper-level government	9	18.37
2 U/C government	21	42.86
3 U/C residents	17	34.69
4 Others	2	4.08
Total	49	100.00

- **jb029_2_8_ : Who Initiated This**

	No	%
1 Upper-level government	10	5.65
2 U/C government	70	39.55
3 U/C residents	89	50.28
4 Others	8	4.52
Total	177	100.00

- **jb029_2_9_ : Who Initiated This**

	No	%
1 Upper-level government	72	42.11
2 U/C government	87	50.88
3 U/C residents	6	3.51
4 Others	6	3.51
Total	171	100.00

- **jb029_2_10_ : Who Initiated This**

	No	%
1 Upper-level government	78	62.40
2 U/C government	41	32.80
3 U/C residents	3	2.40
4 Others	3	2.40
Total	125	100.00

- **jb029_2_11_ : Who Initiated This**

	No	%
1 Upper-level government	49	26.78
2 U/C government	114	62.30
3 U/C residents	13	7.10
4 Others	7	3.83
Total	183	100.00

- **jb029_2_12_ : Who Initiated This**

	No	%
1 Upper-level government	47	31.33
2 U/C government	81	54.00
3 U/C residents	20	13.33
4 Others	2	1.33
Total	150	100.00

- **jb029_2_13_ : Who Initiated This**

	No	%
1 Upper-level government	32	62.75
2 U/C government	6	11.76
3 U/C residents	9	17.65
4 Others	4	7.84
Total	51	100.00

- **jb029_2_14_ : Who Initiated This**

	No	%
1 Upper-level government	20	25.97
2 U/C government	29	37.66
3 U/C residents	18	23.38
4 Others	10	12.99
Total	77	100.00

- **jb029_3_1_ : Main Investor**

	No	%
1 Upper-level government	98	50.00
2 U/C government	59	30.10
3 U/C residents	10	5.10
4 Others	29	14.80
Total	196	100.00

- **jb029_3_2_ : Main Investor**

	No	%
1 Upper-level government	5	23.81
2 U/C government	4	19.05
3 U/C residents	2	9.52
4 Others	10	47.62
Total	21	100.00

- **jb029_3_3_ : Main Investor**

	No	%
1 Upper-level government	88	53.33
2 U/C government	54	32.73
3 U/C residents	4	2.42
4 Others	19	11.52
Total	165	100.00

- **jb029_3_4_ : Main Investor**

	No	%
1 Upper-level government	81	44.26
2 U/C government	69	37.70
3 U/C residents	10	5.46
4 Others	23	12.57
Total	183	100.00

- **jb029_3_5_ : Main Investor**

	No	%
1 Upper-level government	32	17.11
2 U/C government	77	41.18
3 U/C residents	66	35.29
4 Others	12	6.42
Total	187	100.00

- **jb029_3_6_ : Main Investor**

	No	%
1 Upper-level government	48	40.68
2 U/C government	57	48.31
3 U/C residents	4	3.39
4 Others	9	7.63
Total	118	100.00

- **jb029_3_7_ : Main Investor**

	No	%
1 Upper-level government	11	22.92
2 U/C government	16	33.33
3 U/C residents	19	39.58
4 Others	2	4.17
Total	48	100.00

- **jb029_3_8_ : Main Investor**

	No	%
1 Upper-level government	11	6.25
2 U/C government	76	43.18
3 U/C residents	80	45.45
4 Others	9	5.11
Total	176	100.00

- **jb029_3_9_ : Main Investor**

	No	%
1 Upper-level government	80	46.78
2 U/C government	77	45.03
3 U/C residents	5	2.92
4 Others	9	5.26
Total	171	100.00

- **jb029_3_10_ : Main Investor**

	No	%
1 Upper-level government	81	65.32
2 U/C government	38	30.65
3 U/C residents	2	1.61
4 Others	3	2.42
Total	124	100.00

- **jb029_3_11_ : Main Investor**

	No	%
1 Upper-level government	50	27.47
2 U/C government	109	59.89
3 U/C residents	15	8.24
4 Others	8	4.40
Total	182	100.00

- **jb029_3_12_ : Main Investor**

	No	%
1 Upper-level government	44	29.33
2 U/C government	82	54.67
3 U/C residents	20	13.33
4 Others	4	2.67
Total	150	100.00

- **jb029_3_13_ : Main Investor**

	No	%
1 Upper-level government	34	68.00
2 U/C government	3	6.00
3 U/C residents	9	18.00
4 Others	4	8.00
Total	50	100.00

- **jb029_3_14_ : Main Investor**

	No	%
1 Upper-level government	21	27.27
2 U/C government	28	36.36
3 U/C residents	18	23.38
4 Others	10	12.99
Total	77	100.00

- **jb030 : Have Community-Based Elderly Care Center**

	No	%
1 Yes	38	25.00
2 No	114	75.00
Total	152	100.00

- **jb031 : How Large is the Elderly Care Center**

Mean	Min	Max	OBS
174.1	10.0	1,000.0	40

- **jb032 : Num. of Elderly Using the Elderly Care Center**

Mean	Min	Max	OBS
532.0	8.0	3,000.0	32

- **jb033_1 : Others**

	No	%
.E	34	89.47
	1	2.63
	1	2.63
	1	2.63
	1	2.63
Total	38	100.00

- **jb033s1 : Which Type of Service**

	No	%
1 Health care	25	100.00
Total	25	100.00

- **jb033s2 : Which Type of Service**

	No	%
2 Entertain activities	23	100.00
Total	23	100.00

- **jb033s3 : Which Type of Service**

	No	%
3 Exercise equipments	15	100.00
Total	15	100.00

- **jb033s4 : Which Type of Service**

	No	%

4 Other, please specify:	3	100.00
Total	3	100.00

- **jb034 : How Much Each Elderly Need to Pay**

Mean	Min	Max	OBS
44.4	0.0	1,500.0	36

- **jb035 : Who Provides the Funds**

	No	%
1 Community	5	13.51
2 Country finance	15	40.54
3 Both	14	37.84
4 Others	3	8.11
Total	37	100.00

- **jb035_1 : Others**

Chinese Character String

- **jb036 : Percentage of Households with Nanny**

Mean	Min	Max	OBS
1.3	0.0	60.0	444

- **jb037 : Family-Based Elder-Care Center**

	No	%
1 Yes	8	2.60
2 No	300	97.40
Total	308	100.00

- **jb038 : Num. of People Served**

Mean	Min	Max	OBS
120.9	0.0	600.0	8

- **jb040 : Respondent Identity for Part B**

	No	%
1 Village head	43	9.47
2 Village secretary	76	16.74
3 Village accountant	92	20.26
4 Director of the street committee	59	13.00
5 Others	184	40.53
Total	454	100.00

- **jc001 : Population**

Mean	Min	Max	OBS
4,029.8	40.0	44,000.0	453

- **jc002 : How Many of Them Have Non-Agriculture Hukou**

Mean	Min	Max	OBS
1,797.8	0.0	18,046.0	447

- **jc003 : How Many of Them Have Hukou in Your V/C**

Mean	Min	Max	OBS
2,999.9	0.0	18,046.0	438

- **jc003_1 : Num. of Households**

Mean	Min	Max	OBS
924.9	-999.0	6,000.0	438

- **jc004 : How Many of Them Don't Have Hukou in Your V/C**

Mean	Min	Max	OBS
954.8	0.0	45,000.0	440

- **jc004_1 : Num. of Households**

Mean	Min	Max	OBS
404.0	-999.0	40,000.0	440

- **jc005 : Num. of Peoples with Hukou in Your V/C but Not Living Here**

Mean	Min	Max	OBS
578.2	0.0	9,050.0	429

- **jc005_1 : Num. of Households**

Mean	Min	Max	OBS
179.7	-999.0	2,662.0	429

- **jc006 : Migrant**

Mean	Min	Max	OBS
379.3	0.0	35,000.0	427

- **jc007 : Population in the Year before Last**

Mean	Min	Max	OBS
3,813.8	4.0	41,000.0	447

- **jc008 : How Many of Them Have Hukou in Your V/C**

Mean	Min	Max	OBS
2,975.8	10.0	18,046.0	444

- **jc009 : Total Number of Adult Population**

Mean	Min	Max	OBS
2,615.4	100.0	37,200.0	429

- **jc009_1 : How Many Males**

Mean	Min	Max	OBS
1,367.4	-999.0	18,600.0	430

- **jc010 : Total Number of Population Aged 65 and Over**

Mean	Min	Max	OBS

480.9	17.0	5,600.0	425

- **jc010_1 : How Many Males**

Mean	Min	Max	OBS
242.1	8.0	2,801.0	421

- **jc011 : Total Number of Population Aged 80 and Over**

Mean	Min	Max	OBS
79.1	0.0	2,800.0	424

- **jc011_1 : How Many Males**

Mean	Min	Max	OBS
35.1	0.0	1,176.0	420

- **jc012 : Num. of Bachelors Aged Above 40 Who Have Never Been Married**

Mean	Min	Max	OBS
19.8	0.0	356.0	430

- **jc012_1 : How Many Males**

Mean	Min	Max	OBS
7.2	-999.0	250.0	431

- **jc013 : Does Your V/C Have Minorities**

	No	%
1 Yes	233	52.13
2 No	214	47.87
Total	447	100.00

- **jc013_1 : How Many**

	No	%
1	96	40.85

2	40	17.02
3	37	15.74
4	28	11.91
5	17	7.23
6	5	2.13
7	5	2.13
8	5	2.13
16	2	0.85
Total	235	100.00

- **jc014 : Which Minority Has the Largest Population**

Chinese Character String

- **jc015 : How Many Households Belong to this Minority**

Mean	Min	Max	OBS
154.1	0.0	4,800.0	228

- **jc016 : Which Minority Has the Second Largest Population**

Chinese Character String

- **jc017 : How Many Households Belong to this Minority**

Mean	Min	Max	OBS
37.6	0.0	870.0	131

- **jc018 : How Many Households Belong to other Minorities**

Mean	Min	Max	OBS
45.4	0.0	1,309.0	92

- **jc019 : Percentage of the Adult Population That are Illiterate/Semi-Illiterate**

Mean	Min	Max	OBS
11.5	0.0	90.0	411

- **jc019_1 : Percentage Category**

	No	%
1 0-20	27	93.10
2 20-40	2	6.90
Total	29	100.00

- **jc020 : Percentage of the Adult Population Have Completed Only Up to Primary Schools**

Mean	Min	Max	OBS
25.9	0.0	100.0	401

- **jc020_1 : Percentage Category**

	No	%
1 0-10	16	41.03
2 10-20	11	28.21
3 20-30	6	15.38
4 30-40	1	2.56
5 Large than 40	5	12.82
Total	39	100.00

- **jc021 : Percentage of the Adult Population Have Completed Only Up to Junior High Schools**

Mean	Min	Max	OBS
35.5	0.1	85.0	409

- **jc021_1 : Percentage Category**

	No	%
2 10-20	8	22.86
3 20-30	8	22.86
4 30-40	10	28.57
5 Large than 40	9	25.71
Total	35	100.00

- **jc022 : Percentage of the Adult Population Have Completed Only Up to Senior High School**

Mean	Min	Max	OBS

18.8	0.0	73.0	410

- **jc022_1 : Percentage Category**

	No	%
1 0-10	4	11.76
2 10-20	10	29.41
3 20-30	12	35.29
4 30-40	4	11.76
5 Large than 40	4	11.76
Total	34	100.00

- **jc023 : Percentage of the Adult Population Have Completed Only Up to College**

Mean	Min	Max	OBS
5.4	0.0	70.0	406

- **jc023_1 : Percentage Category**

	No	%
1 None	1	2.78
2 0-1	5	13.89
3 1-5	20	55.56
4 5-10	5	13.89
5 10-15	3	8.33
6 Large Than 15	2	5.56
Total	36	100.00

- **jc024 : Percentage of the Adult Population Have Completed Up to Graduate Schools and Abo**

Mean	Min	Max	OBS
0.8	0.0	20.0	410

- **jc024_1 : Percentage Category**

	No	%
1 None	8	23.53
2 0-1	21	61.76
3 1-5	2	5.88
4 5-10	3	8.82

Total	34	100.00

- **jc026 : Respondent Identity for Part C**

	No	%
1 Village head	59	13.05
2 Village secretary	67	14.82
3 Village accountant	104	23.01
4 Director of the street committee	54	11.95
5 Others	168	37.17
Total	452	100.00

- **jd001 : How Many Households Were Engaged in Non-Agricultural Work**

Mean	Min	Max	OBS
161.1	0.0	2,800.0	303

- **jd002 : How Many GETIHU**

Mean	Min	Max	OBS
119.6	0.0	3,800.0	446

- **jd003 : How Many Enterprises**

Mean	Min	Max	OBS
13.0	0.0	850.0	446

- **jd004s1 : Major Products**

	No	%
1 Handicraft products	180	100.00
Total	180	100.00

- **jd004s2 : Major Products**

	No	%
2 Minerals	57	100.00
Total	57	100.00

- **jd004s3 : Major Products**

	No	%
3 Light industry products	107	100.00
Total	107	100.00

- **jd004s4 : Major Products**

	No	%
4 Part manufacturing	56	100.00
Total	56	100.00

- **jd005 : How Many Employees Worked for the Local Firms**

Mean	Min	Max	OBS
416.0	0.0	24,000.0	411

- **jd006 : How Many Males**

Mean	Min	Max	OBS
88.7	0.0	2,500.0	282

- **jd007_1 : Daily Salary of Males**

Mean	Min	Max	OBS
60.2	0.0	1,800.0	114

- **jd007_2 : Monthly Salary of Males**

Mean	Min	Max	OBS
1,592.8	0.0	4,000.0	168

- **jd008_1 : Daily Salary of Females**

Mean	Min	Max	OBS
34.9	0.0	120.0	114

- **jd008_2 : Monthly Salary of Females**

Mean	Min	Max	OBS

1,253.8	0.0	3,600.0	160

- **jd009 : Average Monthly Salary**

	No	%
0	18	10.47
15	1	0.58
18	1	0.58
20	13	7.56
22	11	6.40
23	2	1.16
24	1	0.58
25	27	15.70
26	26	15.12
27	5	2.91
28	40	23.26
29	1	0.58
30	26	15.12
Total	172	100.00

- **jd010 : Average Daily Wage for Manual Male Labor**

Mean	Min	Max	OBS
73.8	0.0	150.0	301

- **jd011 : Average Daily Wage for Manual Female Labor**

Mean	Min	Max	OBS
56.3	0.0	150.0	301

- **jd012 : Monthly Salary for Nannies in Addition to Living and Meal**

Mean	Min	Max	OBS
718.9	0.0	3,000.0	331

- **jd014 : Respondent Identity for Part D**

	No	%
1 Village head	57	12.61
2 Village secretary	71	15.71
3 Village accountant	100	22.12
4 Director of the street committee	53	11.73

5 Others	171	37.83
Total	452	100.00

- **je001 : Total Labor Working Outside More Than 3 Months**

Mean	Min	Max	OBS
499.2	0.0	4,000.0	304

- **je002 : Percentage of Female**

Mean	Min	Max	OBS
36.3	0.0	100.0	305

- **je003 : Percentage of Them Works at Other V/C in this County**

	No	%
1 0-20	223	72.40
2 20-40	38	12.34
3 40-60	24	7.79
4 60-80	13	4.22
5 80-100	10	3.25
Total	308	100.00

- **je004 : Percentage of Them Works at Other Counties in this City**

	No	%
1 0-20	209	67.86
2 20-40	62	20.13
3 40-60	22	7.14
4 60-80	5	1.62
5 80-100	10	3.25
Total	308	100.00

- **je005 : Percentage of Them Works at Other Cities in this Province**

	No	%
1 0-20	205	66.56
2 20-40	59	19.16
3 40-60	22	7.14
4 60-80	14	4.55
5 80-100	8	2.60
Total	308	100.00

- **je006 : Percentage of Them Works at Other Provinces**

	No	%
1 0-20	148	48.05
2 20-40	36	11.69
3 40-60	54	17.53
4 60-80	38	12.34
5 80-100	32	10.39
Total	308	100.00

- **je007s1 : Which Province**

	No	%
1 Anhui	11	100.00
Total	11	100.00

- **je007s2 : Which Province**

	No	%
2 Beijing	85	100.00
Total	85	100.00

- **je007s3 : Which Province**

	No	%
3 Chongqing	7	100.00
Total	7	100.00

- **je007s4 : Which Province**

	No	%
4 Fujian	36	100.00
Total	36	100.00

- **je007s5 : Which Province**

	No	%
5 Gansu	7	100.00
Total	7	100.00

- **je007s6 : Which Province**

	No	%
6 Guangdong	162	100.00
Total	162	100.00

- **je007s7 : Which Province**

	No	%
7 Guangxi	17	100.00
Total	17	100.00

- **je007s8 : Which Province**

	No	%
8 Guizhou	10	100.00
Total	10	100.00

- **je007s9 : Which Province**

	No	%
9 Hainan	11	100.00
Total	11	100.00

- **je007s10 : Which Province**

	No	%
10 Henan	9	100.00
Total	9	100.00

- **je007s11 : Which Province**

	No	%
11 Hebei	22	100.00
Total	22	100.00

- **je007s12 : Which Province**

	No	%
12 Heilongjiang	12	100.00
Total	12	100.00

- **je007s13 : Which Province**

	No	%
13 Hunan	12	100.00
Total	12	100.00

- **je007s14 : Which Province**

	No	%
14 Hubei	9	100.00
Total	9	100.00

- **je007s15 : Which Province**

	No	%
15 Inner mongolia	15	100.00
Total	15	100.00

- **je007s16 : Which Province**

	No	%
16 Jiangsu	68	100.00
Total	68	100.00

- **je007s17 : Which Province**

	No	%
17 Jiangxi	6	100.00
Total	6	100.00

- **je007s18 : Which Province**

	No	%
18 Jilin	9	100.00
Total	9	100.00

- **je007s19 : Which Province**

	No	%
19 Liaoning	12	100.00

Total	12	100.00

- **je007s20 : Which Province**

	No	%
20 Ningxia	6	100.00
Total	6	100.00

- **je007s21 : Which Province**

	No	%
21 Qinghai	4	100.00
Total	4	100.00

- **je007s22 : Which Province**

	No	%
22 Shandong	25	100.00
Total	25	100.00

- **je007s23 : Which Province**

	No	%
23 Shanghai	77	100.00
Total	77	100.00

- **je007s24 : Which Province**

	No	%
24 Shannxi	12	100.00
Total	12	100.00

- **je007s25 : Which Province**

	No	%
25 Shanxi	14	100.00
Total	14	100.00

- **je007s26 : Which Province**

	No	%
26 Sichuan	23	100.00
Total	23	100.00

- **je007s27 : Which Province**

	No	%
27 Tianjin	28	100.00
Total	28	100.00

- **je007s28 : Which Province**

	No	%
28 Tibet	7	100.00
Total	7	100.00

- **je007s29 : Which Province**

	No	%
29 Xinjiang	26	100.00
Total	26	100.00

- **je007s30 : Which Province**

	No	%
30 Yunnan	11	100.00
Total	11	100.00

- **je007s31 : Which Province**

	No	%
31 Zhejiang	113	100.00
Total	113	100.00

- **je007s32 : Which Province**

	No	%
32 Hongkong	1	100.00
Total	1	100.00

- **je007s33 : Which Province**

No Observations

- **je008 : Main Jobs That Male Seek**

	No	%
1 Agriculture, forestry, herd, fishing	11	3.70
2 Mining	9	3.03
3 Manufacture	80	26.94
4 Energy sectorincluding electricity, gas and water	3	1.01
5 Construction	145	48.82
6 Transportation, warehousing industry and postal industry	10	3.37
7 Information transmission, computer service and software industry	2	0.67
8 Wholesale and Retail	17	5.72
9 Lodging and catering sector	7	2.36
11 Real estate business	4	1.35
12 Leasing industry and Business Service Industry	2	0.67
13 Science research, technical service and geology prospecting industry	1	0.34
14 Water conservancy, environments and public facilities management	1	0.34
15 Personal Services and other service industry	4	1.35
19 Common administration and society organize	1	0.34
Total	297	100.00

- **je009 : Average Monthly Wage for These Male Migrants**

Mean	Min	Max	OBS
2,554.8	0.0	15,000.0	299

- **je010 : Main Jobs That Female Seek**

	No	%
1 Agriculture, forestry, herd, fishing	11	3.68
2 Mining	1	0.33
3 Manufacture	143	47.83
4 Energy sectorincluding electricity, gas and water	2	0.67
5 Construction	11	3.68
7 Information transmission, computer service and software industry	6	2.01
8 Wholesale and Retail	18	6.02
9 Lodging and catering sector	53	17.73
10 Finance	3	1.00
12 Leasing industry and Business Service Industry	7	2.34
13 Science research, technical service and geology prospecting industry	1	0.33
15 Personal Services and other service industry	41	13.71
18 Culture, sports and entertainment	1	0.33

19 Common administration and society organize	1	0.33
Total	299	100.00

- **je011 : Average Monthly Wage for These Female Migrants**

Mean	Min	Max	OBS
1,882.7	0.0	15,000.0	299

- **je012 : Num. of Households Has Permanently Moved Outside**

Mean	Min	Max	OBS
56.6	0.0	6,000.0	423

- **je014 : Respondent Identity for Part D**

	No	%
1 Village head	55	12.17
2 Village secretary	71	15.71
3 Village accountant	103	22.79
4 Director of the street committee	56	12.39
5 Others	167	36.95
Total	452	100.00

- **je016 : Num. of Households Moved in Since 2000**

Mean	Min	Max	OBS
83.7	0.0	5,000.0	326

- **jf001s1 : Choose Which Types of Medical Facilities**

	No	%
1 General hospitals	268	100.00
Total	268	100.00

- **jf001s2 : Choose Which Types of Medical Facilities**

	No	%
2 Specialized hospitals	80	100.00
Total	80	100.00

- **jf001s3 : Choose Which Types of Medical Facilities**

	No	%
3 Chinese medicine hospitals	111	100.00
Total	111	100.00

- **jf001s4 : Choose Which Types of Medical Facilities**

	No	%
4 Nearby pharmacy store	104	100.00
Total	104	100.00

- **jf001s5 : Choose Which Types of Medical Facilities**

	No	%
5 Community health care center	65	100.00
Total	65	100.00

- **jf001s6 : Choose Which Types of Medical Facilities**

	No	%
6 Community health care medical post	69	100.00
Total	69	100.00

- **jf001s7 : Choose Which Types of Medical Facilities**

	No	%
7 Township health clinic hospital	255	100.00
Total	255	100.00

- **jf001s8 : Choose Which Types of Medical Facilities**

	No	%
8 Village medical post	246	100.00
Total	246	100.00

- **jf002_1_ : Does Your V/C Have**

	No	%
1 Yes	42	15.56

2 No	228	84.44
Total	270	100.00

- **jf002_1_1_ : How Many**

	No	%
1	33	76.74
2	5	11.63
3	2	4.65
4	1	2.33
8	1	2.33
10	1	2.33
Total	43	100.00

- **jf002_1_2_ : How Many**

	No	%
1	9	75.00
2	3	25.00
Total	12	100.00

- **jf002_1_3_ : How Many**

	No	%
1	18	94.74
2	1	5.26
Total	19	100.00

- **jf002_1_4_ : How Many**

	No	%
1	21	23.33
2	20	22.22
3	21	23.33
4	7	7.78
5	9	10.00
6	1	1.11
8	3	3.33
10	4	4.44
12	1	1.11
16	2	2.22
39	1	1.11
Total	90	100.00

- **jf002_1_5_ : How Many**

	No	%
1	41	89.13
2	5	10.87
Total	46	100.00

- **jf002_1_6_ : How Many**

	No	%
1	45	84.91
2	3	5.66
3	1	1.89
4	3	5.66
6	1	1.89
Total	53	100.00

- **jf002_1_7_ : How Many**

	No	%
0	1	1.05
1	83	87.37
2	9	9.47
4	1	1.05
5	1	1.05
Total	95	100.00

- **jf002_1_8_ : How Many**

	No	%
1	145	64.44
2	40	17.78
3	19	8.44
4	12	5.33
5	1	0.44
6	3	1.33
7	2	0.89
8	2	0.89
12	1	0.44
Total	225	100.00

- **jf002_2_ : Does Your V/C Have**

	No	%

1 Yes	11	13.25
2 No	72	86.75
Total	83	100.00

- **jf002_3_ : Does Your V/C Have**

	No	%
1 Yes	18	15.79
2 No	96	84.21
Total	114	100.00

- **jf002_4_ : Does Your V/C Have**

	No	%
1 Yes	91	89.22
2 No	11	10.78
Total	102	100.00

- **jf002_5_ : Does Your V/C Have**

	No	%
1 Yes	45	69.23
2 No	20	30.77
Total	65	100.00

- **jf002_6_ : Does Your V/C Have**

	No	%
1 Yes	56	83.58
2 No	11	16.42
Total	67	100.00

- **jf002_7_ : Does Your V/C Have**

	No	%
1 Yes	93	36.61
2 No	161	63.39
Total	254	100.00

- **jf002_8_ : Does Your V/C Have**

	No	%
1 Yes	228	93.06
2 No	17	6.94
Total	245	100.00

- **jf003_1_1_ : Province**

	No	%
1 This province	225	98.68
2 Other province	3	1.32
Total	228	100.00

- **jf003_1_2_ : Province**

	No	%
1 This province	72	100.00
Total	72	100.00

- **jf003_1_3_ : Province**

	No	%
1 This province	95	100.00
Total	95	100.00

- **jf003_1_4_ : Province**

	No	%
1 This province	11	100.00
Total	11	100.00

- **jf003_1_5_ : Province**

	No	%
1 This province	20	100.00
Total	20	100.00

- **jf003_1_6_ : Province**

	No	%
1 This province	9	81.82
2 Other province	2	18.18

Total	11	100.00

- **jf003_1_7_ : Province**

	No	%
1 This province	162	100.00
Total	162	100.00

- **jf003_1_8_ : Province**

	No	%
1 This province	16	100.00
Total	16	100.00

- **jf003_2_1_ : City/County**

	No	%
1 This county / city	220	97.78
2 Other county / city	5	2.22
Total	225	100.00

- **jf003_2_2_ : City/County**

	No	%
1 This county / city	63	88.73
2 Other county / city	8	11.27
Total	71	100.00

- **jf003_2_3_ : City/County**

	No	%
1 This county / city	91	95.79
2 Other county / city	4	4.21
Total	95	100.00

- **jf003_2_4_ : City/County**

	No	%
1 This county / city	11	100.00
Total	11	100.00

- **jf003_2_5_ : City/County**

	No	%
1 This county / city	20	100.00
Total	20	100.00

- **jf003_2_6_ : City/County**

	No	%
1 This county / city	9	100.00
Total	9	100.00

- **jf003_2_7_ : City/County**

	No	%
1 This county / city	160	98.77
2 Other county / city	2	1.23
Total	162	100.00

- **jf003_2_8_ : City/County**

	No	%
1 This county / city	16	100.00
Total	16	100.00

- **jf003_3_1_ : Township/District**

	No	%
1 This township/district	95	43.38
2 Other township/district	124	56.62
Total	219	100.00

- **jf003_3_2_ : Township/District**

	No	%
1 This township/district	28	44.44
2 Other township/district	35	55.56
Total	63	100.00

- **jf003_3_3_ : Township/District**

	No	%
1 This township/district	41	45.56
2 Other township/district	49	54.44
Total	90	100.00

- **jf003_3_4_ : Township/District**

	No	%
1 This township/district	10	90.91
2 Other township/district	1	9.09
Total	11	100.00

- **jf003_3_5_ : Township/District**

	No	%
1 This township/district	17	85.00
2 Other township/district	3	15.00
Total	20	100.00

- **jf003_3_6_ : Township/District**

	No	%
1 This township/district	8	88.89
2 Other township/district	1	11.11
Total	9	100.00

- **jf003_3_7_ : Township/District**

	No	%
1 This township/district	155	96.88
2 Other township/district	5	3.13
Total	160	100.00

- **jf003_3_8_ : Township/District**

	No	%
1 This township/district	13	81.25
2 Other township/district	3	18.75
Total	16	100.00

- **jf003_4_1_ : Village/Street**

	No	%
1 This village/street	41	40.59
2 Other village/street	60	59.41
Total	101	100.00

- **jf003_4_2_ : Village/Street**

	No	%
1 This village/street	5	15.63
2 Other village/street	27	84.38
Total	32	100.00

- **jf003_4_3_ : Village/Street**

	No	%
1 This village/street	9	21.43
2 Other village/street	33	78.57
Total	42	100.00

- **jf003_4_4_ : Village/Street**

	No	%
1 This village/street	2	20.00
2 Other village/street	8	80.00
Total	10	100.00

- **jf003_4_5_ : Village/Street**

	No	%
1 This village/street	8	47.06
2 Other village/street	9	52.94
Total	17	100.00

- **jf003_4_6_ : Village/Street**

	No	%
1 This village/street	4	50.00
2 Other village/street	4	50.00
Total	8	100.00

- **jf003_4_7_ : Village/Street**

	No	%
1 This village/street	16	10.46
2 Other village/street	137	89.54
Total	153	100.00

- **jf003_4_8_ : Village/Street**

	No	%
1 This village/street	5	38.46
2 Other village/street	8	61.54
Total	13	100.00

- **jf004_1_ : Is This Facility Public or Private**

	No	%
1 Public	258	95.91
2 Private	11	4.09
Total	269	100.00

- **jf004_2_ : Is This Facility Public or Private**

	No	%
1 Public	60	74.07
2 Private	21	25.93
Total	81	100.00

- **jf004_3_ : Is This Facility Public or Private**

	No	%
1 Public	107	95.54
2 Private	5	4.46
Total	112	100.00

- **jf004_4_ : Is This Facility Public or Private**

	No	%
1 Public	12	11.76
2 Private	90	88.24
Total	102	100.00

- **jf004_5_ : Is This Facility Public or Private**

	No	%
1 Public	50	78.13
2 Private	14	21.88
Total	64	100.00

- **jf004_6_ : Is This Facility Public or Private**

	No	%
1 Public	48	73.85
2 Private	17	26.15
Total	65	100.00

- **jf004_7_ : Is This Facility Public or Private**

	No	%
1 Public	235	92.52
2 Private	19	7.48
Total	254	100.00

- **jf004_8_ : Is This Facility Public or Private**

	No	%
1 Public	89	36.93
2 Private	152	63.07
Total	241	100.00

- **jf005_1_ : The Level of This Facility**

	No	%
1 county/district	217	80.97
2 Regional /city	29	10.82
3 Provincial/ affiliated to a ministry	11	4.10
4 Military	2	0.75
5 Others	6	2.24
6 Not applicable	3	1.12
Total	268	100.00

- **jf005_2_ : The Level of This Facility**

	No	%

1 county/district	52	65.00
2 Regional /city	12	15.00
3 Provincial/ affiliated to a ministry	8	10.00
5 Others	6	7.50
6 Not applicable	2	2.50
Total	80	100.00

- **jf005_3_ : The Level of This Facility**

	No	%
1 county/district	85	77.98
2 Regional /city	16	14.68
3 Provincial/ affiliated to a ministry	5	4.59
6 Not applicable	3	2.75
Total	109	100.00

- **jf006_1_ : Distance from The Facility to The V/C Office**

Mean	Min	Max	OBS
14.1	0.0	130.0	262

- **jf006_2_ : Distance from The Facility to The V/C Office**

Mean	Min	Max	OBS
50.0	0.0	900.0	80

- **jf006_3_ : Distance from The Facility to The V/C Office**

Mean	Min	Max	OBS
31.9	0.0	2,000.0	109

- **jf006_4_ : Distance from The Facility to The V/C Office**

Mean	Min	Max	OBS
0.6	0.0	10.0	102

- **jf006_5_ : Distance from The Facility to The V/C Office**

Mean	Min	Max	OBS
2.5	0.0	100.0	64

- **jf006_6_ : Distance from The Facility to The V/C Office**

Mean	Min	Max	OBS
0.4	0.0	15.0	64

- **jf006_7_ : Distance from The Facility to The V/C Office**

Mean	Min	Max	OBS
7.7	0.0	999.0	250

- **jf006_8_ : Distance from The Facility to The V/C Office**

Mean	Min	Max	OBS
3.6	0.0	800.0	242

- **jf007_1_ : Main Means of Transportation**

	No	%
1 Walk	40	15.04
2 Bus	149	56.02
3 Car	29	10.90
4 Bicycle or other manual vehicles	11	4.14
5 Electric bicycle/electric tricycle	12	4.51
6 Motorcycle	25	9.40
Total	266	100.00

- **jf007_2_ : Main Means of Transportation**

	No	%
1 Walk	12	14.81
2 Bus	52	64.20
3 Car	8	9.88
4 Bicycle or other manual vehicles	1	1.23
5 Electric bicycle/electric tricycle	4	4.94
6 Motorcycle	3	3.70
8 Train	1	1.23
Total	81	100.00

- **jf007_3_ : Main Means of Transportation**

	No	%
1 Walk	13	11.71

2 Bus	72	64.86
3 Car	8	7.21
4 Bicycle or other manual vehicles	2	1.80
5 Electric bicycle/electric tricycle	10	9.01
6 Motorcycle	6	5.41
Total	111	100.00

- **jf007_4_ : Main Means of Transportation**

	No	%
1 Walk	82	80.39
2 Bus	5	4.90
3 Car	1	0.98
4 Bicycle or other manual vehicles	2	1.96
5 Electric bicycle/electric tricycle	3	2.94
6 Motorcycle	9	8.82
Total	102	100.00

- **jf007_5_ : Main Means of Transportation**

	No	%
1 Walk	45	69.23
2 Bus	7	10.77
4 Bicycle or other manual vehicles	1	1.54
5 Electric bicycle/electric tricycle	9	13.85
6 Motorcycle	3	4.62
Total	65	100.00

- **jf007_6_ : Main Means of Transportation**

	No	%
1 Walk	50	75.76
2 Bus	3	4.55
4 Bicycle or other manual vehicles	2	3.03
5 Electric bicycle/electric tricycle	5	7.58
6 Motorcycle	6	9.09
Total	66	100.00

- **jf007_7_ : Main Means of Transportation**

	No	%
1 Walk	67	26.59
2 Bus	41	16.27
3 Car	16	6.35
4 Bicycle or other manual vehicles	13	5.16

5 Electric bicycle/electric tricycle	43	17.06
6 Motorcycle	71	28.17
9 Animal or animal pulled cart	1	0.40
Total	252	100.00

- **jf007_8_ : Main Means of Transportation**

	No	%
1 Walk	171	70.95
2 Bus	1	0.41
4 Bicycle or other manual vehicles	16	6.64
5 Electric bicycle/electric tricycle	22	9.13
6 Motorcycle	31	12.86
Total	241	100.00

- **jf008_1_1_ : Hours**

	No	%
0	203	76.89
1	43	16.29
2	12	4.55
3	2	0.76
4	2	0.76
6	1	0.38
20	1	0.38
Total	264	100.00

- **jf008_1_2_ : Hours**

	No	%
0	57	71.25
1	16	20.00
2	3	3.75
4	2	2.50
10	1	1.25
20	1	1.25
Total	80	100.00

- **jf008_1_3_ : Hours**

	No	%
0	87	79.09
1	17	15.45
2	2	1.82
4	2	1.82

5	1	0.91
10	1	0.91
Total	110	100.00

- **jf008_1_4_ : Hours**

	No	%
0	97	95.10
1	4	3.92
2	1	0.98
Total	102	100.00

- **jf008_1_5_ : Hours**

	No	%
0	64	98.46
1	1	1.54
Total	65	100.00

- **jf008_1_6_ : Hours**

	No	%
0	65	100.00
Total	65	100.00

- **jf008_1_7_ : Hours**

	No	%
0	232	93.55
1	13	5.24
2	2	0.81
3	1	0.40
Total	248	100.00

- **jf008_1_8_ : Hours**

	No	%
0	232	96.67
1	7	2.92
2	1	0.42
Total	240	100.00

- **jf008_2_1_ : Minutes**

	No	%
0	36	13.64
3	3	1.14
4	1	0.38
5	24	9.09
6	2	0.76
7	2	0.76
8	2	0.76
9	1	0.38
10	57	21.59
12	1	0.38
13	1	0.38
15	22	8.33
20	32	12.12
25	2	0.76
30	55	20.83
35	1	0.38
40	11	4.17
45	4	1.52
50	6	2.27
55	1	0.38
Total	264	100.00

- **jf008_2_2_ : Minutes**

	No	%
0	14	17.50
1	1	1.25
5	8	10.00
10	14	17.50
15	3	3.75
20	13	16.25
30	14	17.50
40	6	7.50
45	2	2.50
50	5	6.25
Total	80	100.00

- **jf008_2_3_ : Minutes**

	No	%
0	18	16.36
2	1	0.91
3	1	0.91
4	1	0.91
5	7	6.36

7	1	0.91
10	17	15.45
12	1	0.91
13	1	0.91
15	11	10.00
17	1	0.91
20	16	14.55
25	1	0.91
30	20	18.18
40	9	8.18
45	1	0.91
50	3	2.73
Total	110	100.00

- **jf008_2_4_ : Minutes**

	No	%
0	17	16.67
1	3	2.94
2	2	1.96
3	4	3.92
4	1	0.98
5	26	25.49
6	2	1.96
7	2	1.96
10	26	25.49
15	7	6.86
20	4	3.92
30	7	6.86
40	1	0.98
Total	102	100.00

- **jf008_2_5_ : Minutes**

	No	%
0	5	7.69
1	2	3.08
2	2	3.08
3	4	6.15
5	15	23.08
6	1	1.54
9	1	1.54
10	18	27.69
12	1	1.54
15	8	12.31
20	4	6.15
30	3	4.62
50	1	1.54
Total	65	100.00

- **jf008_2_6_ : Minutes**

	No	%
0	5	7.69
1	1	1.54
2	1	1.54
3	3	4.62
4	2	3.08
5	23	35.38
6	1	1.54
8	1	1.54
9	1	1.54
10	19	29.23
15	5	7.69
30	3	4.62
Total	65	100.00

- **jf008_2_7_ : Minutes**

	No	%
0	15	6.05
1	3	1.21
2	1	0.40
3	2	0.81
5	29	11.69
6	3	1.21
7	1	0.40
8	5	2.02
9	1	0.40
10	66	26.61
13	1	0.40
15	29	11.69
16	1	0.40
20	35	14.11
25	4	1.61
30	40	16.13
40	5	2.02
45	2	0.81
50	5	2.02
Total	248	100.00

- **jf008_2_8_ : Minutes**

	No	%
0	22	9.17
1	2	0.83

2	4	1.67
3	6	2.50
4	2	0.83
5	83	34.58
6	2	0.83
7	1	0.42
8	7	2.92
10	68	28.33
12	2	0.83
15	18	7.50
20	10	4.17
25	2	0.83
30	10	4.17
40	1	0.42
Total	240	100.00

- **jf009_1_ : Total Transportation Cost**

Mean	Min	Max	OBS
6.7	0.0	150.0	260

- **jf009_2_ : Total Transportation Cost**

Mean	Min	Max	OBS
26.6	0.0	500.0	80

- **jf009_3_ : Total Transportation Cost**

Mean	Min	Max	OBS
7.0	0.0	80.0	110

- **jf009_4_ : Total Transportation Cost**

Mean	Min	Max	OBS
0.5	0.0	10.0	102

- **jf009_5_ : Total Transportation Cost**

Mean	Min	Max	OBS
0.8	0.0	10.0	65

- **jf009_6_ : Total Transportation Cost**

Mean	Min	Max	OBS
0.3	0.0	4.0	65

- **jf009_7_ : Total Transportation Cost**

Mean	Min	Max	OBS
-1.0	-999.0	30.0	246

- **jf009_8_ : Total Transportation Cost**

Mean	Min	Max	OBS
-3.8	-999.0	10.0	238

- **jf010s1 : Insurances are Implemented through V/C Committee**

	No	%
1 Urban resident medical insurance	140	100.00
Total	140	100.00

- **jf010s2 : Insurances are Implemented through V/C Committee**

	No	%
2 Rural cooperative medical insurance	338	100.00
Total	338	100.00

- **jf011 : When Did Your V/C Start The Urban Resident Medical Insurance**

Mean	Min	Max	OBS
2,007.6	2,005.0	2,011.0	132

- **jf012_1 : Num. of Households Participated Urban Resident Medical Insurance Up to 2010**

Mean	Min	Max	OBS
1,150.3	-999.0	37,000.0	131

- **jf012_2 : Num. of Households Participated Urban Resident Medical Insurance Up to 2009**

Mean	Min	Max	OBS
741.6	-999.0	12,500.0	127

- **jf012_3 : Num. of Households Participated Urban Resident Medical Insurance Up to 2008**

Mean	Min	Max	OBS
540.7	-999.0	12,090.0	107

- **jf012_4 : Num. of Households Participated Urban Resident Medical Insurance Up to 2007**

Mean	Min	Max	OBS
462.7	-999.0	11,692.0	61

- **jf012_5 : Num. of Households Participated Urban Resident Medical Insurance Up to 2006**

Mean	Min	Max	OBS
-7.0	-999.0	5,560.0	23

- **jf012_6 : Num. of Households Participated Urban Resident Medical Insurance Up to 2005**

Mean	Min	Max	OBS
-366.8	-999.0	770.0	12

- **jf013 : Premium for Participants in Urban Resident Medical Insurance**

Mean	Min	Max	OBS
311.3	0.0	3,672.0	139

- **jf014 : When Did Your V/C Start The RCMI**

Mean	Min	Max	OBS
2,006.2	2,003.0	2,011.0	332

- **jf015_1 : Num. of Households Participated The RCMI Up to 2010**

Mean	Min	Max	OBS
636.1	0.0	5,660.0	319

- **jf015_2 : Num. of Households Participated The RCMI Up to 2009**

Mean	Min	Max	OBS
610.7	0.0	5,300.0	306

- **jf015_3 : Num. of Households Participated The RCMI Up to 2008**

Mean	Min	Max	OBS
591.0	0.0	4,800.0	292

- **jf015_4 : Num. of Households Participated The RCMI Up to 2007**

Mean	Min	Max	OBS
545.1	0.0	3,910.0	250

- **jf015_5 : Num. of Households Participated The RCMI Up to 2006**

Mean	Min	Max	OBS
517.2	0.0	2,870.0	172

- **jf015_6 : Num. of Households Participated The RCMI Up to 2005**

Mean	Min	Max	OBS
504.1	0.0	2,840.0	98

- **jf015_7 : Num. of Households Participated The RCMI Up to 2004**

Mean	Min	Max	OBS
582.9	0.0	2,820.0	49

- **jf015_8 : Num. of Households Participated The RCMI Up to 2003**

Mean	Min	Max	OBS

622.7	0.0	2,800.0	27

- **jf016 : How Much do Villagers Pay for the RCMI Each Year**

Mean	Min	Max	OBS
56.8	-999.0	6,800.0	334

- **jf017_1 : Num. of Deaths In the Past 3 Years**

Mean	Min	Max	OBS
48.2	0.0	458.0	429

- **jf017_2 : Num. of People Who are Died of Suicide in the Past 3 Years**

	No	%
0	335	78.27
1	41	9.58
2	22	5.14
3	10	2.34
4	5	1.17
5	9	2.10
7	1	0.23
8	1	0.23
10	1	0.23
16	1	0.23
18	1	0.23
36	1	0.23
Total	428	100.00

- **jf018_1_1_ : Disease**

Chinese Character String

- **jf018_1_2_ : Disease**

Chinese Character String

- **jf018_1_3_ : Disease**

Chinese Character String

- **jf018_2_1_ : Num. of Deaths**

Mean	Min	Max	OBS
-34.6	-999.0	999.0	411

- **jf018_2_2_ : Num. of Deaths**

Mean	Min	Max	OBS
-58.2	-999.0	999.0	411

- **jf018_2_3_ : Num. of Deaths**

Mean	Min	Max	OBS
-112.3	-999.0	999.0	411

- **jf020 : Respondent Identity for Part D**

	No	%
1 Village head	49	10.84
2 Village secretary	61	13.50
3 Village accountant	84	18.58
4 Director of the street committee	49	10.84
5 Others	209	46.24
Total	452	100.00

- **jg001 : Does Your V/C Have Unemployement Subsidies**

	No	%
1 Yes	68	15.04
2 No	384	84.96
Total	452	100.00

- **jg002 : Year**

Mean	Min	Max	OBS
2,005.3	1,993.0	2,011.0	69

- **jg003 : Num. of People Received Unemployment Subsidies in 2010**

Mean	Min	Max	OBS
168.2	0.0	2,000.0	66

- **jg004 : Average Subsidies Per Month**

Mean	Min	Max	OBS
360.8	0.0	1,000.0	66

- **jg005 : Does Your V/C Have Minimum Living Allowance**

	No	%
1 Yes	373	82.34
2 No	80	17.66
Total	453	100.00

- **jg006 : Year**

Mean	Min	Max	OBS
2,003.9	1,988.0	2,010.0	367

- **jg007 : Num. of People Received Minimum Living Allowance in 2010**

Mean	Min	Max	OBS
150.8	0.0	1,421.0	370

- **jg008 : Average Subsidies Per Month**

Mean	Min	Max	OBS
134.8	0.0	1,100.0	342

- **jg009_1_ : Does Your V/C Have Other Subsidies**

	No	%
1 Yes. Please specify	177	39.25
2 No	274	60.75
Total	451	100.00

- **jg009_1_1_ : Name of Subsidy Program**

Chinese Character String

- **jg009_1_2_ : Name of Subsidy Program**

Chinese Character String

- **jg009_1_3_ : Name of Subsidy Program**

Chinese Character String

- **jg009_1_4_ : Name of Subsidy Program**

	No	%
.D	4	80.00
.E	1	20.00
Total	5	100.00

- **jg009_1_5_ : Name of Subsidy Program**

	No	%
.D	2	50.00
.E	2	50.00
Total	4	100.00

- **jg009_2_ : Does Your V/C Have Other Subsidies**

	No	%
1 Yes. Please specify	17	9.50
2 No	162	90.50
Total	179	100.00

- **jg009_3_ : Does Your V/C Have Other Subsidies**

	No	%
1 Yes. Please specify	1	5.88

2 No	16	94.12
Total	17	100.00

- **jg009_4_ : Does Your V/C Have Other Subsidies**

No Observations

- **jg009_5_ : Does Your V/C Have Other Subsidies**

	No	%
2 No	2	100.00
Total	2	100.00

- **jg010_1_ : When Did this Subsidy Start**

Mean	Min	Max	OBS
2,000.0	1,949.0	2,010.0	177

- **jg010_2_ : When Did this Subsidy Start**

Mean	Min	Max	OBS
2,002.8	1,958.0	2,011.0	16

- **jg010_3_ : When Did this Subsidy Start**

Mean	Min	Max	OBS
2,003.0	2,003.0	2,003.0	1

- **jg010_4_ : When Did this Subsidy Start**

Mean	Min	Max	OBS
2,010.0	2,010.0	2,010.0	1

- **jg010_5_ : When Did this Subsidy Start**

No Observations

- **jg011_1_ : How Many Households Received This Subsidy**

Mean	Min	Max	OBS
33.1	0.0	600.0	175

- **jg011_2_ : How Many Households Received This Subsidy**

Mean	Min	Max	OBS
41.8	1.0	263.0	17

- **jg011_3_ : How Many Households Received This Subsidy**

Mean	Min	Max	OBS
189.0	189.0	189.0	1

- **jg011_4_ : How Many Households Received This Subsidy**

Mean	Min	Max	OBS
1,198.0	1,198.0	1,198.0	1

- **jg011_5_ : How Many Households Received This Subsidy**

No Observations

- **jg012_1_ : Average Subsidy Standard of this Subsidy Program**

Mean	Min	Max	OBS
468.7	0.0	50,000.0	173

- **jg012_2_ : Average Subsidy Standard of this Subsidy Program**

Mean	Min	Max	OBS
984.3	8.0	13,000.0	16

- **jg012_3_ : Average Subsidy Standard of this Subsidy Program**

	No	%

10	1	100.00
Total	1	100.00

- **jg012_4_ : Average Subsidy Standard of this Subsidy Program**

	No	%
10	1	100.00
Total	1	100.00

- **jg012_5_ : Average Subsidy Standard of this Subsidy Program**

No Observations

- **jg013 : Does Your V/C Have Subsidies for Being Parents of Single Child**

	No	%
1 Yes	366	80.97
2 No	86	19.03
Total	452	100.00

- **jg014 : Year**

Mean	Min	Max	OBS
2,002.2	1,976.0	2,011.0	350

- **jg015 : Num. of Households Received Subsidies for Being Parents of Single Child**

Mean	Min	Max	OBS
92.6	0.0	4,900.0	348

- **jg016 : Average Subsidy Standard**

Mean	Min	Max	OBS
200.9	0.0	50,000.0	355

- **jg017 : Does Your V/C Have Farm Subsidies**

	No	%
1 Yes	291	94.48
2 No	17	5.52
Total	308	100.00

- **jg018 : Year**

Mean	Min	Max	OBS
2,005.4	1,998.0	2,011.0	288

- **jg019 : Num. of Households Received Farm Subsidies**

Mean	Min	Max	OBS
591.7	0.0	7,000.0	288

- **jg020 : Average Subsidy Standard**

Mean	Min	Max	OBS
74.6	0.0	848.0	285

- **jg021 : Does Your V/C Have Subsidies for Reforestation**

	No	%
1 Yes	114	37.38
2 No	191	62.62
Total	305	100.00

- **jg022 : Year**

Mean	Min	Max	OBS
2,003.5	1,988.0	2,011.0	115

- **jg023 : Num. of Households Received Subsidies for Reforestation**

Mean	Min	Max	OBS
169.2	0.0	1,000.0	114

- **jg024 : Average Subsidy Standard**

Mean	Min	Max	OBS
201.7	0.0	3,000.0	109

- **jg025 : Does Your V/C Issue Pension to Persons Older than 65**

	No	%
1 Yes	103	22.94
2 No	346	77.06
Total	449	100.00

- **jg026 : Year**

Mean	Min	Max	OBS
2,007.5	1,985.0	2,011.0	111

- **jg027 : Num. of Persons Received**

Mean	Min	Max	OBS
246.1	0.0	3,000.0	106

- **jg028 : Average Subsidy standard**

Mean	Min	Max	OBS
84.7	0.0	800.0	108

- **jg029 : Does Your V/C Have Subsidy for Persons Older than 80**

	No	%
1 Yes	140	31.11
2 No	310	68.89
Total	450	100.00

- **jg030 : Year**

Mean	Min	Max	OBS
2,008.0	1,975.0	2,011.0	143

- **jg031 : Num. of Persons Received**

Mean	Min	Max	OBS
50.7	0.0	700.0	137

- **jg032 : Average Subsidy Standard**

Mean	Min	Max	OBS
79.8	4.0	900.0	139

- **jg033 : Does Your V/C Have NRPS**

	No	%
1 Yes	141	45.93
2 No	166	54.07
Total	307	100.00

- **jg034 : Year**

Mean	Min	Max	OBS
2,009.0	1,988.0	2,011.0	144

- **jg035 : Num. of Persons Received NRPS**

Mean	Min	Max	OBS
332.2	0.0	3,000.0	134

- **jg036 : Average Subsidy Standard**

Mean	Min	Max	OBS
90.3	0.0	1,000.0	132

- **jg037 : Does Your V/C Have the ORPS**

	No	%
1 Yes	51	16.83
2 No	252	83.17
Total	303	100.00

- **jg038 : Year**

Mean	Min	Max	OBS
2,002.8	1,986.0	2,011.0	49

- **jg039 : Num. of Persons Received ORPS**

Mean	Min	Max	OBS
182.9	0.0	1,504.0	46

- **jg040 : Average Subsidy Standard**

Mean	Min	Max	OBS
115.2	0.0	750.0	40

- **jg042 : Respondent Identity for Part D**

	No	%
1 Village head	55	12.14
2 Village secretary	64	14.13
3 Village accountant	109	24.06
4 Director of the street committee	53	11.70
5 Others	172	37.97
Total	453	100.00

- **jh001_1 : Year**

Mean	Min	Max	OBS
2,011.0	2,011.0	2,012.0	436

- **jh001_2 : Month**

	No	%
1	2	0.46
5	115	26.38
6	223	51.15
7	44	10.09
8	24	5.50
9	2	0.46
10	5	1.15
11	8	1.83
12	13	2.98
Total	436	100.00

- **jh001_3 : Day**

	No	%
1	11	2.52
2	10	2.29
3	18	4.13
4	13	2.98
5	20	4.59
6	12	2.75
7	11	2.52
8	10	2.29
9	10	2.29
10	19	4.36
11	18	4.13
12	4	0.92
13	11	2.52
14	13	2.98
15	12	2.75
16	9	2.06
17	11	2.52
18	16	3.67
19	11	2.52
20	29	6.65
21	19	4.36
22	14	3.21
23	22	5.05
24	15	3.44
25	17	3.90
26	9	2.06
27	20	4.59
28	15	3.44
29	17	3.90
30	15	3.44
31	5	1.15
Total	436	100.00

- **jh001_4 : Time**

	No	%
1	2	0.46
2	6	1.38
3	2	0.46
4	3	0.69
6	2	0.46
7	4	0.92
8	15	3.44
9	39	8.94
10	61	13.99
11	35	8.03
12	16	3.67

13	19	4.36
14	41	9.40
15	60	13.76
16	43	9.86
17	36	8.26
18	12	2.75
19	14	3.21
20	17	3.90
21	8	1.83
22	1	0.23
Total	436	100.00

- **jh001_5 : Number**

	No	%
2	254	58.26
3	182	41.74
Total	436	100.00

- **jh002_1_1_ : Education**

	No	%
1 Primary school	108	24.60
2 Junior high school	160	36.45
3 High school	92	20.96
4 Middle professional school	33	7.52
5 Advanced professional school	19	4.33
6 University and above	12	2.73
7 illiterate	15	3.42
Total	439	100.00

- **jh002_1_2_ : Education**

	No	%
1 Primary school	122	27.73
2 Junior high school	150	34.09
3 High school	76	17.27
4 Middle professional school	39	8.86
5 Advanced professional school	18	4.09
6 University and above	19	4.32
7 illiterate	16	3.64
Total	440	100.00

- **jh002_1_3_ : Education**

	No	%
1 Primary school	68	31.48
2 Junior high school	74	34.26
3 High school	42	19.44
4 Middle professional school	11	5.09
5 Advanced professional school	7	3.24
6 University and above	3	1.39
7 illiterate	11	5.09
Total	216	100.00

- **jh002_2_1_ : Gender**

	No	%
1 Male	370	84.09
2 Female	70	15.91
Total	440	100.00

- **jh002_2_2_ : Gender**

	No	%
1 Male	350	79.55
2 Female	90	20.45
Total	440	100.00

- **jh002_2_3_ : Gender**

	No	%
1 Male	168	78.14
2 Female	47	21.86
Total	215	100.00

- **jh002_3_1_ : Age**

	No	%
23	1	0.23
24	1	0.23
25	1	0.23
31	1	0.23
32	1	0.23
36	1	0.23
37	1	0.23
38	1	0.23
39	3	0.68
40	1	0.23

41	5	1.14
42	3	0.68
43	2	0.45
44	3	0.68
45	4	0.91
46	9	2.05
47	9	2.05
48	6	1.36
49	4	0.91
50	15	3.41
51	7	1.59
52	6	1.36
53	5	1.14
54	5	1.14
55	9	2.05
56	15	3.41
57	20	4.55
58	12	2.73
59	12	2.73
60	20	4.55
61	9	2.05
62	20	4.55
63	13	2.95
64	10	2.27
65	21	4.77
66	14	3.18
67	13	2.95
68	12	2.73
69	7	1.59
70	28	6.36
71	5	1.14
72	8	1.82
73	10	2.27
74	9	2.05
75	9	2.05
76	12	2.73
77	4	0.91
78	9	2.05
79	1	0.23
80	11	2.50
81	8	1.82
82	2	0.45
83	6	1.36
84	5	1.14
85	1	0.23
87	2	0.45
88	3	0.68
89	1	0.23
90	1	0.23
91	1	0.23
100	2	0.45
Total	440	100.00

- **jh002_3_2_ : Age**

	No	%
30	1	0.23
32	1	0.23
33	1	0.23
34	1	0.23
35	2	0.46
36	2	0.46
37	1	0.23
38	4	0.91
39	3	0.68
40	2	0.46
41	3	0.68
42	6	1.37
43	4	0.91
44	3	0.68
45	7	1.59
46	2	0.46
47	3	0.68
48	13	2.96
49	5	1.14
50	13	2.96
51	8	1.82
52	7	1.59
53	11	2.51
54	14	3.19
55	10	2.28
56	16	3.64
57	12	2.73
58	12	2.73
59	11	2.51
60	22	5.01
61	17	3.87
62	13	2.96
63	12	2.73
64	8	1.82
65	21	4.78
66	11	2.51
67	16	3.64
68	13	2.96
69	4	0.91
70	24	5.47
71	12	2.73
72	10	2.28
73	10	2.28
74	6	1.37
75	7	1.59
76	8	1.82
77	2	0.46
78	9	2.05
79	2	0.46

80	8	1.82
81	1	0.23
82	2	0.46
83	2	0.46
84	3	0.68
85	4	0.91
86	6	1.37
87	2	0.46
88	2	0.46
90	2	0.46
100	2	0.46
Total	439	100.00

- **jh002_3_3_ : Age**

	No	%
29	1	0.47
35	1	0.47
36	3	1.40
37	1	0.47
39	2	0.93
40	1	0.47
41	1	0.47
43	2	0.93
45	3	1.40
46	1	0.47
47	2	0.93
48	4	1.86
49	4	1.86
50	9	4.19
51	1	0.47
52	5	2.33
53	3	1.40
54	6	2.79
55	7	3.26
56	6	2.79
57	8	3.72
58	7	3.26
59	7	3.26
60	13	6.05
61	3	1.40
62	8	3.72
63	5	2.33
64	3	1.40
65	13	6.05
66	6	2.79
67	12	5.58
68	6	2.79
69	1	0.47
70	14	6.51
71	1	0.47

72	5	2.33
73	5	2.33
74	7	3.26
75	5	2.33
76	5	2.33
78	3	1.40
79	2	0.93
82	2	0.93
83	2	0.93
85	1	0.47
87	3	1.40
90	1	0.47
92	1	0.47
99	2	0.93
100	1	0.47
Total	215	100.00

- **jh003 : When did Your Village Found the People's Commune**

Mean	Min	Max	OBS
1,959.7	1,949.0	1,998.0	289

- **jh004 : When did the People's Commune Disintegrate**

Mean	Min	Max	OBS
1,980.2	1,952.0	2,006.0	294

- **jh005 : When did the Household Contract Responsibility System Operate**

Mean	Min	Max	OBS
1,981.6	1,959.0	1,996.0	303

- **jh006 : Has Your village Reallocated Land Since the HCRS Operated**

	No	%
1 Yes	216	70.59
2 No	90	29.41
Total	306	100.00

- **jh007 : Year**

Mean	Min	Max	OBS
1,996.9	1,960.0	2,010.0	215

- **jh008 : Year that Residents First Allowed to Subcontract, Rend Land to Family Member**

Mean	Min	Max	OBS
1,994.4	1,956.0	2,008.0	274

- **jh008_1 : Year that Residents First Allowed to Subcontract, Rend Land to Non Family Member**

Mean	Min	Max	OBS
1,995.3	1,956.0	2,011.0	269

- **jh009 : How Many Rusticated Youth did Your Village Accept During 1960s and 1970s**

Mean	Min	Max	OBS
38.8	-999.0	2,012.0	297

- **jh010 : Year**

Mean	Min	Max	OBS
1,971.5	1,900.0	2,011.0	251

- **jh011 : How Many Rusticated Youth Settled Down instead of Going Back to Town**

	No	%
0	229	83.88
1	14	5.13
2	8	2.93
3	6	2.20
4	3	1.10
5	5	1.83
6	1	0.37
8	1	0.37
10	2	0.73
14	1	0.37
15	1	0.37
20	1	0.37
32	1	0.37
Total	273	100.00

- **jh012 : Year**

Mean	Min	Max	OBS
1,977.0	1,900.0	2,011.0	255

- **jh013 : Where Are the Youth Rusticated to Your Village Mostly from**

	No	%
1 This province	226	90.04
2 Other province,	25	9.96
Total	251	100.00

- **jh013_1 : Province**

	No	%
1 Anhui	1	3.85
2 Beijing	2	7.69
3 Chongqing	4	15.38
8 Guizhou	1	3.85
12 Heilongjiang	1	3.85
13 Hunan	1	3.85
23 Shanghai	13	50.00
27 Tianjin	2	7.69
30 Yunnan	1	3.85
Total	26	100.00

- **jh013_2 : Where Are the Youth Rusticated to Your Village Mostly from**

	No	%
1 This county/city	156	62.15
2 Other county/city, specify	95	37.85
Total	251	100.00

- **jh014 : Num. of Barefoot Doctors During 1970s**

	No	%
0	4	1.32
1	64	21.05
2	90	29.61
3	72	23.68
4	30	9.87

5	22	7.24
6	8	2.63
7	5	1.64
8	2	0.66
10	4	1.32
12	1	0.33
20	1	0.33
45	1	0.33
Total	304	100.00

- **jh015 : Was Any of Primary Schools Merged or Revocated**

	No	%
1 Yes	165	53.57
2 No	143	46.43
Total	308	100.00

- **jh016 : Year**

Mean	Min	Max	OBS
1,998.9	1,969.0	2,010.0	163

- **jh017 : How Many Primary Schools Before that**

	No	%
0	1	0.61
1	100	60.61
2	25	15.15
3	22	13.33
4	8	4.85
5	5	3.03
7	3	1.82
10	1	0.61
Total	165	100.00

- **jh018_1_1_ : Num. of People Join the Army**

	No	%
0	106	37.32
1	90	31.69
2	51	17.96
3	20	7.04
4	9	3.17
5	4	1.41
8	1	0.35

9	1	0.35
10	1	0.35
11	1	0.35
Total	284	100.00

- **jh018_1_2_ : Num. of People Join the Army**

	No	%
0	95	33.93
1	91	32.50
2	50	17.86
3	29	10.36
4	9	3.21
5	3	1.07
6	1	0.36
7	1	0.36
8	1	0.36
Total	280	100.00

- **jh018_1_3_ : Num. of People Join the Army**

	No	%
0	79	29.48
1	84	31.34
2	58	21.64
3	27	10.07
4	11	4.10
5	5	1.87
6	3	1.12
7	1	0.37
Total	268	100.00

- **jh018_1_4_ : Num. of People Join the Army**

	No	%
0	78	30.71
1	84	33.07
2	59	23.23
3	22	8.66
4	10	3.94
7	1	0.39
Total	254	100.00

- **jh018_1_5_ : Num. of People Join the Army**

	No	%
0	81	33.61
1	72	29.88
2	56	23.24
3	17	7.05
4	12	4.98
5	1	0.41
7	1	0.41
8	1	0.41
Total	241	100.00

- **jh018_1_6_ : Num. of People Join the Army**

	No	%
0	31	13.54
1	22	9.61
2	28	12.23
3	32	13.97
4	26	11.35
5	11	4.80
6	18	7.86
7	8	3.49
8	10	4.37
9	5	2.18
10	17	7.42
11	2	0.87
12	4	1.75
13	1	0.44
14	3	1.31
15	4	1.75
16	2	0.87
17	1	0.44
18	1	0.44
20	3	1.31
Total	229	100.00

- **jh018_1_7_ : Num. of People Join the Army**

	No	%
0	34	15.96
1	22	10.33
2	27	12.68
3	16	7.51
4	32	15.02
5	24	11.27
6	11	5.16
7	5	2.35
8	7	3.29

9	2	0.94
10	9	4.23
11	5	2.35
12	2	0.94
13	4	1.88
14	3	1.41
15	5	2.35
16	1	0.47
20	3	1.41
22	1	0.47
Total	213	100.00

- **jh018_1_8_ : Num. of People Join the Army**

	No	%
0	30	15.00
1	29	14.50
2	28	14.00
3	16	8.00
4	18	9.00
5	22	11.00
6	12	6.00
7	7	3.50
8	8	4.00
9	5	2.50
10	6	3.00
11	2	1.00
12	2	1.00
13	1	0.50
15	4	2.00
16	2	1.00
17	2	1.00
18	2	1.00
20	1	0.50
21	1	0.50
22	1	0.50
30	1	0.50
Total	200	100.00

- **jh018_1_9_ : Num. of People Join the Army**

	No	%
0	35	17.59
1	22	11.06
2	22	11.06
3	20	10.05
4	9	4.52
5	26	13.07
6	10	5.03

7	4	2.01
8	10	5.03
9	2	1.01
10	15	7.54
11	1	0.50
12	5	2.51
13	3	1.51
14	1	0.50
15	2	1.01
16	2	1.01
18	1	0.50
20	4	2.01
23	1	0.50
25	2	1.01
30	1	0.50
75	1	0.50
Total	199	100.00

- **jh018_1_10_ : Num. of People Join the Army**

	No	%
0	33	16.58
1	26	13.07
2	17	8.54
3	17	8.54
4	17	8.54
5	17	8.54
6	9	4.52
7	6	3.02
8	7	3.52
9	5	2.51
10	14	7.04
11	3	1.51
12	9	4.52
13	2	1.01
15	7	3.52
16	1	0.50
18	1	0.50
20	2	1.01
24	2	1.01
25	1	0.50
30	1	0.50
46	1	0.50
99	1	0.50
Total	199	100.00

- **jh018_1_11_ : Num. of People Join the Army**

	No	%

0	34	17.17
1	17	8.59
2	16	8.08
3	21	10.61
4	17	8.59
5	18	9.09
6	17	8.59
7	5	2.53
8	5	2.53
9	5	2.53
10	11	5.56
11	2	1.01
12	9	4.55
13	4	2.02
14	2	1.01
15	3	1.52
16	2	1.01
17	1	0.51
18	1	0.51
20	2	1.01
21	1	0.51
28	2	1.01
30	1	0.51
35	2	1.01
Total	198	100.00

- **jh018_2_1_ : Num. of People Admitted to the University**

	No	%
0	51	18.55
1	32	11.64
2	47	17.09
3	27	9.82
4	25	9.09
5	24	8.73
6	16	5.82
7	9	3.27
8	11	4.00
9	4	1.45
10	12	4.36
11	1	0.36
12	1	0.36
13	1	0.36
14	2	0.73
15	2	0.73
16	2	0.73
18	1	0.36
20	4	1.45
23	1	0.36
30	1	0.36
50	1	0.36

Total	275	100.00

- **jh018_2_2_ : Num. of People Admitted to the University**

	No	%
0	53	19.78
1	36	13.43
2	41	15.30
3	32	11.94
4	25	9.33
5	20	7.46
6	22	8.21
7	6	2.24
8	7	2.61
9	3	1.12
10	7	2.61
11	2	0.75
12	4	1.49
15	4	1.49
19	1	0.37
20	3	1.12
21	1	0.37
40	1	0.37
Total	268	100.00

- **jh018_2_3_ : Num. of People Admitted to the University**

	No	%
0	50	19.92
1	38	15.14
2	47	18.73
3	31	12.35
4	27	10.76
5	14	5.58
6	12	4.78
7	4	1.59
8	4	1.59
9	4	1.59
10	5	1.99
11	1	0.40
12	2	0.80
13	2	0.80
14	2	0.80
15	2	0.80
16	2	0.80
17	1	0.40
18	1	0.40
20	1	0.40
40	1	0.40

Total	251	100.00

- **jh018_2_4_ : Num. of People Admitted to the University**

	No	%
0	52	21.58
1	56	23.24
2	35	14.52
3	31	12.86
4	17	7.05
5	16	6.64
6	10	4.15
7	2	0.83
8	4	1.66
9	2	0.83
10	5	2.07
11	2	0.83
12	1	0.41
14	1	0.41
15	2	0.83
16	1	0.41
18	1	0.41
20	2	0.83
23	1	0.41
Total	241	100.00

- **jh018_2_5_ : Num. of People Admitted to the University**

	No	%
0	61	26.64
1	47	20.52
2	37	16.16
3	25	10.92
4	18	7.86
5	11	4.80
6	9	3.93
7	2	0.87
8	3	1.31
10	6	2.62
12	4	1.75
13	2	0.87
15	1	0.44
16	1	0.44
20	1	0.44
21	1	0.44
Total	229	100.00

- **jh018_2_6_ : Num. of People Admitted to the University**

Mean	Min	Max	OBS
6.7	0.0	108.0	218

- **jh018_2_7_ : Num. of People Admitted to the University**

	No	%
0	73	37.06
1	24	12.18
2	24	12.18
3	15	7.61
4	12	6.09
5	11	5.58
6	10	5.08
7	3	1.52
8	7	3.55
9	2	1.02
10	2	1.02
11	1	0.51
12	4	2.03
15	2	1.02
16	1	0.51
20	1	0.51
23	1	0.51
45	1	0.51
47	1	0.51
50	2	1.02
Total	197	100.00

- **jh018_2_8_ : Num. of People Admitted to the University**

	No	%
0	80	42.33
1	20	10.58
2	32	16.93
3	20	10.58
4	7	3.70
5	10	5.29
6	2	1.06
7	2	1.06
8	1	0.53
9	3	1.59
10	3	1.59
11	2	1.06
12	2	1.06
20	2	1.06
29	1	0.53
30	1	0.53

46	1	0.53
Total	189	100.00

- **jh018_2_9_ : Num. of People Admitted to the University**

	No	%
0	86	44.79
1	33	17.19
2	28	14.58
3	19	9.90
4	5	2.60
5	6	3.13
6	3	1.56
7	1	0.52
9	1	0.52
10	2	1.04
13	2	1.04
14	1	0.52
16	1	0.52
17	1	0.52
30	1	0.52
38	1	0.52
40	1	0.52
Total	192	100.00

- **jh018_2_10_ : Num. of People Admitted to the University**

	No	%
0	103	54.50
1	31	16.40
2	26	13.76
3	6	3.17
4	6	3.17
5	6	3.17
6	2	1.06
7	1	0.53
8	1	0.53
10	2	1.06
11	1	0.53
13	1	0.53
15	1	0.53
30	1	0.53
35	1	0.53
Total	189	100.00

- **jh018_2_11_ : Num. of People Admitted to the University**

	No	%
0	119	63.98
1	36	19.35
2	7	3.76
3	12	6.45
4	3	1.61
5	3	1.61
8	1	0.54
9	1	0.54
10	1	0.54
14	1	0.54
20	1	0.54
21	1	0.54
Total	186	100.00

- **jh019 : When Did Your V/C Start to Execute the Family Planning Policy**

Mean	Min	Max	OBS
1,979.7	1,970.0	2,011.0	436

- **jh020 : The Specific Policy for Ethnic Han**

	No	%
1 A couple can only birth of the one-child	171	38.08
2 Can have a second child if the first child is a girl	135	30.07
3 A couple can birth of two-children	125	27.84
4 A couple can birth of more than two children	18	4.01
Total	449	100.00

- **jh021 : Was There Any Change on the Fmaily Planning Policy**

	No	%
1 Yes	179	39.96
2 No	269	60.04
Total	448	100.00

- **jh022 : Changed to What**

	No	%
1 A couple can only birth of the one-child	54	29.67
2 Can have a second child if the first child is a girl	101	55.49
3 A couple can birth of two-children	21	11.54
4 A couple can birth of more than two children	6	3.30
Total	182	100.00

- **jh023 : Year**

Mean	Min	Max	OBS
1,993.3	1,973.0	2,011.0	179

- **jh024 : The Current Family Planning Policy for Ethnic Han**

	No	%
1 A couple can only birth of the one-child	180	40.27
2 Can have a second child if the first child is a girl	218	48.77
3 A couple can birth of two-children	45	10.07
4 A couple can birth of more than two children	4	0.89
Total	447	100.00

- **jh025 : Family Planning Policy for Ethnic Minorities**

	No	%
1 A couple can only birth of the one-child	95	25.82
2 Can have a second child if the first child is a girl	105	28.53
3 A couple can birth of two-children	137	37.23
4 A couple can birth of more than two children	31	8.42
Total	368	100.00

- **jh026 : Is There Penalty for Unplanned Birth**

	No	%
1 Yes,when year	377	87.47
2 No	54	12.53
Total	431	100.00

- **jh026_1 :**

Mean	Min	Max	OBS
1,983.8	1,968.0	2,011.0	377

- **jh027 : What Was the Initial Penalty Amount**

Mean	Min	Max	OBS
3,036.9	0.0	100,000.0	354

- **jh028 : Any Change on the Penalty Policy**

	No	%
1 Yes	288	77.01
2 No	86	22.99
Total	374	100.00

- **jh029a_1_ : When**

Mean	Min	Max	OBS
1,989.4	1,952.0	2,010.0	249

- **jh029a_2_ : When**

Mean	Min	Max	OBS
1,996.8	1,980.0	2,009.0	226

- **jh029a_3_ : When**

Mean	Min	Max	OBS
2,004.0	1,980.0	2,010.0	204

- **jh029b_1_ : Amount**

Mean	Min	Max	OBS
5,962.4	0.0	260,000.0	245

- **jh029b_2_ : Amount**

Mean	Min	Max	OBS
33,487.7	100.0	5,001,500.0	222

- **jh029b_3_ : Amount**

Mean	Min	Max	OBS
23,957.0	600.0	420,000.0	201

- **jh030 : In the Early 1970s, Any 'Late, Scarcy, Few' Policy**

	No	%

1 Yes	211	48.96
2 No	220	51.04
Total	431	100.00

- **jh031 : Describe the 'Late, Scarcy, Few' Policy**

Chinese Character String

- **jh032 : When did the Residents Start to Get National Identiciation Cards**

Mean	Min	Max	OBS
1,987.0	1,950.0	2,010.0	431

- **jh033 : When did the Residents Start to Get the Second-Generation National Identificatio**

Mean	Min	Max	OBS
2,005.9	1,992.0	2,010.0	448

- **ji001 : Any Serious Natural Disasters in the Past 5 Years**

	No	%
1 Yes	160	35.40
2 No	292	64.60
Total	452	100.00

- **ji002_a_1_ : Were This Natural Disaster in Your V/C**

	No	%
1 Yes	73	43.98
2 No	93	56.02
Total	166	100.00

- **ji002_a_2_ : Were This Natural Disaster in Your V/C**

	No	%
1 Yes	79	47.88
2 No	86	52.12
Total	165	100.00

- **ji002_a_3_ : Were This Natural Disaster in Your V/C**

	No	%
1 Yes	19	11.52
2 No	146	88.48
Total	165	100.00

- **ji002_a_4_ : Were This Natural Disaster in Your V/C**

	No	%
1 Yes	34	20.61
2 No	131	79.39
Total	165	100.00

- **ji002_a_5_ : Were This Natural Disaster in Your V/C**

	No	%
1 Yes	13	7.88
2 No	152	92.12
Total	165	100.00

- **ji002_a_6_ : Were This Natural Disaster in Your V/C**

	No	%
1 Yes	70	42.42
2 No	95	57.58
Total	165	100.00

- **ji002_a_7_ : Were This Natural Disaster in Your V/C**

	No	%
1 Yes	13	7.88
2 No	152	92.12
Total	165	100.00

- **ji002_b_1_ : Times Occurred**

	No	%
1	36	45.00
2	20	25.00

3	9	11.25
5	6	7.50
6	1	1.25
8	2	2.50
10	2	2.50
15	4	5.00
Total	80	100.00

- **ji002_b_2_ : Times Occurred**

	No	%
1	33	41.25
2	21	26.25
3	8	10.00
4	6	7.50
5	11	13.75
6	1	1.25
Total	80	100.00

- **ji002_b_3_ : Times Occurred**

	No	%
1	10	52.63
2	6	31.58
3	2	10.53
10	1	5.26
Total	19	100.00

- **ji002_b_4_ : Times Occurred**

	No	%
1	32	91.43
2	3	8.57
Total	35	100.00

- **ji002_b_5_ : Times Occurred**

	No	%
1	5	29.41
2	4	23.53
3	3	17.65
4	1	5.88
11	1	5.88
15	2	11.76
20	1	5.88

Total	17	100.00

- **ji002_b_6_ : Times Occurred**

	No	%
1	59	83.10
2	8	11.27
3	1	1.41
4	1	1.41
5	2	2.82
Total	71	100.00

- **ji002_b_7_ : Times Occurred**

	No	%
1	11	78.57
3	1	7.14
5	1	7.14
15	1	7.14
Total	14	100.00

- **ji002_c_1_ : Year**

Mean	Min	Max	OBS
2,008.5	2,005.0	2,011.0	77

- **ji002_c_2_ : Year**

Mean	Min	Max	OBS
2,009.0	2,006.0	2,011.0	81

- **ji002_c_3_ : Year**

Mean	Min	Max	OBS
2,008.6	2,006.0	2,011.0	19

- **ji002_c_4_ : Year**

Mean	Min	Max	OBS
2,008.3	2,006.0	2,011.0	34

- **ji002_c_5_ : Year**

Mean	Min	Max	OBS
2,008.4	2,006.0	2,010.0	13

- **ji002_c_6_ : Year**

Mean	Min	Max	OBS
2,008.3	2,006.0	2,011.0	71

- **ji002_c_7_ : Year**

Mean	Min	Max	OBS
2,008.4	2,006.0	2,011.0	13

- **ji002_d_1_ : Month**

	No	%
1	1	1.27
2	1	1.27
4	1	1.27
5	7	8.86
6	16	20.25
7	38	48.10
8	11	13.92
9	1	1.27
10	1	1.27
11	2	2.53
Total	79	100.00

- **ji002_d_2_ : Month**

	No	%
1	1	1.23
2	2	2.47
3	7	8.64
4	8	9.88
5	12	14.81
6	11	13.58
7	16	19.75
8	10	12.35
9	4	4.94
10	7	8.64
11	2	2.47

12	1	1.23
Total	81	100.00

- **ji002_d_3_ : Month**

	No	%
1	1	5.26
2	1	5.26
3	1	5.26
4	7	36.84
5	3	15.79
8	1	5.26
9	1	5.26
10	2	10.53
12	2	10.53
Total	19	100.00

- **ji002_d_4_ : Month**

	No	%
2	1	2.86
4	1	2.86
5	29	82.86
9	1	2.86
10	2	5.71
11	1	2.86
Total	35	100.00

- **ji002_d_5_ : Month**

	No	%
3	1	7.14
4	2	14.29
5	3	21.43
6	1	7.14
7	1	7.14
8	5	35.71
9	1	7.14
Total	14	100.00

- **ji002_d_6_ : Month**

	No	%
1	30	42.25
2	8	11.27

3	1	1.41
4	1	1.41
6	5	7.04
8	1	1.41
11	6	8.45
12	19	26.76
Total	71	100.00

- **ji002_d_7_ : Month**

	No	%
4	1	7.69
5	2	15.38
6	1	7.69
7	2	15.38
8	4	30.77
9	3	23.08
Total	13	100.00

- **ji002_e_1_ : Num. of People Injured or Dead**

	No	%
0	67	91.78
1	2	2.74
2	2	2.74
6	1	1.37
15	1	1.37
Total	73	100.00

- **ji002_e_2_ : Num. of People Injured or Dead**

	No	%
0	77	97.47
1	1	1.27
8	1	1.27
Total	79	100.00

- **ji002_e_3_ : Num. of People Injured or Dead**

	No	%
0	16	84.21
1	1	5.26
2	1	5.26
3	1	5.26
Total	19	100.00

- **ji002_e_4_ : Num. of People Injured or Dead**

	No	%
0	28	82.35
1	3	8.82
5	1	2.94
20	1	2.94
36	1	2.94
Total	34	100.00

- **ji002_e_5_ : Num. of People Injured or Dead**

	No	%
0	11	84.62
3	2	15.38
Total	13	100.00

- **ji002_e_6_ : Num. of People Injured or Dead**

	No	%
0	64	91.43
1	2	2.86
2	2	2.86
4	1	1.43
10	1	1.43
Total	70	100.00

- **ji002_e_7_ : Num. of People Injured or Dead**

	No	%
0	12	92.31
2	1	7.69
Total	13	100.00

- **ji002_other : Other Disaster**

Chinese Character String

- **ji003_1a : The Most Serve One**

Chinese Character String

- **ji003_1b : Year**

Mean	Min	Max	OBS
1,982.9	1,945.0	2,011.0	262

- **ji003_1c : Month**

	No	%
1	21	8.02
2	4	1.53
3	19	7.25
4	20	7.63
5	35	13.36
6	49	18.70
7	63	24.05
8	25	9.54
9	9	3.44
10	7	2.67
11	1	0.38
12	9	3.44
Total	262	100.00

- **ji003_1d : Year**

Mean	Min	Max	OBS
1,983.4	1,946.0	2,011.0	262

- **ji003_1e : Month**

	No	%
1	16	6.11
2	10	3.82
3	2	0.76
4	9	3.44
5	26	9.92
6	32	12.21
7	40	15.27
8	45	17.18
9	30	11.45
10	33	12.60

11	3	1.15
12	16	6.11
Total	262	100.00

- **ji003_1f : Num. of People Injured or Dead**

Mean	Min	Max	OBS
9.4	0.0	600.0	262

- **ji003_1g : Percentage of People Injured or Dead**

Mean	Min	Max	OBS
1.5	0.0	99.0	261

- **ji003_1h : Percentage of Drop in Grain Production**

	No	%
0	94	36.15
2	2	0.77
5	2	0.77
7	1	0.38
10	6	2.31
11	1	0.38
15	1	0.38
20	9	3.46
30	18	6.92
40	13	5.00
45	2	0.77
50	19	7.31
60	12	4.62
66	2	0.77
70	13	5.00
72	1	0.38
75	1	0.38
80	22	8.46
87	1	0.38
90	12	4.62
98	2	0.77
99	2	0.77
100	24	9.23
Total	260	100.00

- **ji003_2a : The Second Serve One**

Chinese Character String

- **ji003_2b : Year**

Mean	Min	Max	OBS
1,992.2	1,945.0	2,011.0	261

- **ji003_2c : Month**

	No	%
1	32	12.26
2	11	4.21
3	19	7.28
4	13	4.98
5	34	13.03
6	52	19.92
7	51	19.54
8	23	8.81
9	10	3.83
10	5	1.92
11	3	1.15
12	8	3.07
Total	261	100.00

- **ji003_2d : Year**

Mean	Min	Max	OBS
1,992.6	1,945.0	2,011.0	261

- **ji003_2e : Month**

	No	%
1	23	8.81
2	17	6.51
3	6	2.30
4	9	3.45
5	25	9.58
6	24	9.20
7	42	16.09
8	47	18.01
9	28	10.73
10	24	9.20
11	2	0.77
12	14	5.36
Total	261	100.00

- **ji003_2f : Num. of People Injured or Dead**

Mean	Min	Max	OBS
-4.6	-999.0	300.0	261

- **ji003_2g : Percentage of People Injured or Dead**

Mean	Min	Max	OBS
0.6	0.0	99.0	260

- **ji003_2h : Percentage of Drop in Grain Production**

	No	%
0	115	44.40
2	1	0.39
5	4	1.54
6	2	0.77
8	1	0.39
10	15	5.79
15	1	0.39
18	1	0.39
20	14	5.41
25	1	0.39
30	15	5.79
33	1	0.39
35	1	0.39
40	13	5.02
45	1	0.39
48	1	0.39
50	16	6.18
60	8	3.09
65	1	0.39
67	1	0.39
70	8	3.09
72	1	0.39
80	12	4.63
86	1	0.39
90	10	3.86
95	3	1.16
98	1	0.39
100	10	3.86
Total	259	100.00

- **ji003_3a : The Third Serve One**

Chinese Character String

- **ji003_3b : Year**

Mean	Min	Max	OBS
1,999.5	1,945.0	2,011.0	261

- **ji003_3c : Month**

	No	%
1	46	17.62
2	15	5.75
3	21	8.05
4	18	6.90
5	33	12.64
6	37	14.18
7	37	14.18
8	23	8.81
9	7	2.68
10	6	2.30
11	8	3.07
12	10	3.83
Total	261	100.00

- **ji003_3d : Year**

Mean	Min	Max	OBS
1,999.6	1,945.0	2,011.0	261

- **ji003_3e : Month**

	No	%
1	30	11.49
2	28	10.73
3	12	4.60
4	10	3.83
5	24	9.20
6	28	10.73
7	29	11.11
8	42	16.09
9	23	8.81
10	20	7.66

11	4	1.53
12	11	4.21
Total	261	100.00

- **ji003_3f : Num. of People Injured or Dead**

Mean	Min	Max	OBS
-6.7	-999.0	99.0	261

- **ji003_3g : Percentage of People Injured or Dead**

Mean	Min	Max	OBS
0.4	0.0	99.0	260

- **ji003_3h : Percentage of Drop in Grain Production**

	No	%
0	144	55.60
1	4	1.54
2	1	0.39
3	1	0.39
5	2	0.77
6	1	0.39
8	2	0.77
10	13	5.02
15	3	1.16
20	11	4.25
24	1	0.39
25	2	0.77
30	17	6.56
35	1	0.39
36	1	0.39
40	10	3.86
50	13	5.02
59	1	0.39
60	8	3.09
65	1	0.39
70	5	1.93
80	4	1.54
85	1	0.39
88	1	0.39
90	4	1.54
91	1	0.39
100	6	2.32
Total	259	100.00

- **jj001_1a : First Crop**

Chinese Character String

- **jj001_1b : Mu**

Mean	Min	Max	OBS
1,867.4	0.0	19,100.0	283

- **jj001_1c : Jin**

Mean	Min	Max	OBS
87,817.0	0.0	14,400,000.0	281

- **jj001_1d : Mechanical Harvesting**

	No	%
1 Yes	137	48.07
2 No	148	51.93
Total	285	100.00

- **jj001_1e : Current Number of Crop Maturity**

	No	%
0	1	0.35
1	226	79.58
2	53	18.66
4	1	0.35
6	2	0.70
12	1	0.35
Total	284	100.00

- **jj001_1f : Highest Number of crop maturity**

	No	%
0	1	0.35
1	197	69.37
2	76	26.76
3	5	1.76
4	1	0.35
7	2	0.70

11	1	0.35
12	1	0.35
Total	284	100.00

- **jj001_2a : Second Crop**

Chinese Character String

- **jj001_2b : Mu**

Mean	Min	Max	OBS
1,126.2	0.0	12,000.0	279

- **jj001_2c : Jin**

Mean	Min	Max	OBS
24,375.7	0.0	2,400,000.0	277

- **jj001_2d : Mechanical Harvesting**

	No	%
1 Yes	69	24.21
2 No	216	75.79
Total	285	100.00

- **jj001_2e : Current Number of Crop Maturity**

	No	%
0	8	2.88
1	239	85.97
2	27	9.71
6	3	1.08
7	1	0.36
Total	278	100.00

- **jj001_2f : Highest Number of crop maturity**

	No	%
0	8	2.88
1	230	82.73

2	30	10.79
3	3	1.08
5	1	0.36
6	1	0.36
7	3	1.08
11	2	0.72
Total	278	100.00

- **jj001_3a : Second Crop**

Chinese Character String

- **jj001_3b : Mu**

Mean	Min	Max	OBS
551.1	0.0	9,999.0	272

- **jj001_3c : Jin**

Mean	Min	Max	OBS
15,628.2	0.0	2,000,000.0	272

- **jj001_3d : Mechanical Harvesting**

	No	%
1 Yes	43	15.09
2 No	242	84.91
Total	285	100.00

- **jj001_3e : Current Number of Crop Maturity**

	No	%
0	15	5.45
1	226	82.18
2	23	8.36
3	6	2.18
4	2	0.73
6	2	0.73
11	1	0.36
Total	275	100.00

- **jj001_3f : Highest Number of crop maturity**

	No	%
0	15	5.45
1	219	79.64
2	27	9.82
3	8	2.91
4	3	1.09
5	1	0.36
7	2	0.73
Total	275	100.00

- **jj002 : Percentage of Mechanical Harvesting**

Mean	Min	Max	OBS
37.4	0.0	100.0	302

- **jj003 : Rent Big Machines**

	No	%
1 Yes	99	32.57
2 No	205	67.43
Total	304	100.00

- **jj004 : Rental Price**

Mean	Min	Max	OBS
2,690.3	0.0	50,000.0	88

- **jj005 : Where are These Big Machines Coming from**

	No	%
1 This County/City	67	67.68
2 Other City/County in this Province	11	11.11
3 Other Province	21	21.21
Total	99	100.00

- **jj006 : Name of Most Use Fertilizer**

Chinese Character String

- **jj007 : Price of That Fertilizer**

Mean	Min	Max	OBS
5.9	0.0	260.0	295

- **jj008 : Price of Rice Seed**

Mean	Min	Max	OBS
13.4	0.0	140.0	237

- **jj009 : Price of Rice Seedling**

Mean	Min	Max	OBS
10.1	0.0	999.0	158

- **jj010 : Price of Wheat Seed**

Mean	Min	Max	OBS
8.0	0.0	999.0	216

- **jj011 : total Agricultural Revenue in 2010**

Mean	Min	Max	OBS
75,476.5	0.0	5,370,000.0	277

- **jj012 : Total Industrial Revenue in 2010**

Mean	Min	Max	OBS
11,211.5	0.0	1,200,000.0	262

- **jj013 : Total V/C Public Expenditure in 2010**

Mean	Min	Max	OBS
727,368,936.0	0.0	200000000000.0	277

- **jj014 : Total V/C Income in 2010**

Mean	Min	Max	OBS

9,120,079.3	0.0	1000000000.0	281

- **jj015 : Total V/C Net Investment in 2010**

Mean	Min	Max	OBS
8,198,591.0	0.0	2000000000.0	263

- **jj016 : Per-Capita Net Income of Your V/C**

Mean	Min	Max	OBS
5,569.6	0.0	50,415.0	423

- **jj017 : Average Price of the LIJI Part of Pork**

Mean	Min	Max	OBS
14.4	0.0	25.0	445

- **jj018 : Average Price of Chicken Eggs**

Mean	Min	Max	OBS
9.9	0.0	1,700.0	450

- **jj019 : Average Price of Rice**

Mean	Min	Max	OBS
2.5	1.0	25.0	451

- **jj020 : Average Price of Wheat**

Mean	Min	Max	OBS
2.0	0.0	12.0	424

- **jj021 : Average Price of Natural Gas**

Mean	Min	Max	OBS
17.0	0.0	999.0	236

- **jj022 : Average Price of Liquefied Petroleum Gas**

Mean	Min	Max	OBS
101.4	0.0	180.0	415

- **jj023 : Average Price of Water**

Mean	Min	Max	OBS
3.1	0.0	458.0	403

- **jj024 : Average Price of Electricity**

Mean	Min	Max	OBS
0.9	0.0	8.0	451

- **jj025 : Average Price of Coal**

Mean	Min	Max	OBS
14.6	0.0	1,800.0	330

- **jj026 : Average Price of New Apartment**

Mean	Min	Max	OBS
3,144.8	0.0	150,000.0	379

- **jj027 : Average Price of Used Apartment**

Mean	Min	Max	OBS
2,863.4	0.0	100,000.0	316

- **jj028 : Average Rental Price of 2-Bedroom Apartment**

Mean	Min	Max	OBS
411.6	0.0	4,500.0	346

- **jj029 : Rental Percentage**

Mean	Min	Max	OBS
7.5	0.0	100.0	432

- **jj030 : Percentage of Households Living in Commercial Housing**

Mean	Min	Max	OBS
12.8	0.0	100.0	432

- **jj031 : Percentage of Households that Loan from Financial Institutions**

Mean	Min	Max	OBS
13.6	0.0	90.0	283

- **jj032 : Degree of Difficulty in Loaning**

	No	%
1 Very difficult	72	23.53
2 Somewhat difficult	68	22.22
3 The same	55	17.97
4 Somewhat easy	100	32.68
5 Easily	11	3.59
Total	306	100.00

- **jj034 : Respondent Identity for Part J**

	No	%
1 Village head	52	11.50
2 Village secretary	65	14.38
3 Village accountant	109	24.12
4 Director of the street committee	52	11.50
5 Others	174	38.50
Total	452	100.00

- **jj036 : Maximum Loan**

Mean	Min	Max	OBS
21,015.6	0.0	300,000.0	220

- **jj037s1 : Pawn**

	No	%
1 Rural Housing	99	100.00
Total	99	100.00

- **jj037s2 : Pawn**

	No	%
2 Zhaijidi	49	100.00
Total	49	100.00

- **jj037s3 : Pawn**

	No	%
3 Contracted land	21	100.00
Total	21	100.00

- **jj037s4 : Pawn**

	No	%
4 Option of Collective Assets	9	100.00
Total	9	100.00

- **jj037s5 : Pawn**

	No	%
5 None of the above	107	100.00
Total	107	100.00

- **jj038_1 : Percentage**

Mean	Min	Max	OBS
83.2	0.0	100.0	195

- **jj038_2 : Percentage**

	No	%
0	20	41.67
1	2	4.17
2	1	2.08
10	4	8.33
20	4	8.33
25	1	2.08
30	1	2.08
40	2	4.17
50	5	10.42
70	1	2.08

80	2	4.17
90	1	2.08
100	4	8.33
Total	48	100.00

- **jj038_3 : Percentage**

	No	%
0	23	60.53
1	2	5.26
5	1	2.63
8	1	2.63
10	2	5.26
15	2	5.26
20	4	10.53
40	1	2.63
50	2	5.26
Total	38	100.00

- **jj038_4 : Percentage**

	No	%
0	25	58.14
2	3	6.98
5	1	2.33
10	1	2.33
15	1	2.33
20	3	6.98
30	1	2.33
35	1	2.33
50	1	2.33
60	1	2.33
80	1	2.33
98	1	2.33
99	1	2.33
100	2	4.65
Total	43	100.00

- **jj038_5 : Percentage**

	No	%
0	25	43.86
2	1	1.75
5	4	7.02
8	2	3.51
10	5	8.77
18	1	1.75

20	6	10.53
40	1	1.75
50	1	1.75
80	1	1.75
98	1	1.75
99	2	3.51
100	7	12.28
Total	57	100.00

- **jj038s1 : Lending Channel**

	No	%
1 Formal Commercial Bank	194	100.00
Total	194	100.00

- **jj038s2 : Lending Channel**

	No	%
2 Town/Village Bank	38	100.00
Total	38	100.00

- **jj038s3 : Lending Channel**

	No	%
3 Rural Credit Union Funds	26	100.00
Total	26	100.00

- **jj038s4 : Lending Channel**

	No	%
4 Underground Bank	30	100.00
Total	30	100.00

- **jj038s5 : Lending Channel**

	No	%
5 Other	45	100.00
Total	45	100.00

- **jk001 : This V/C Has Roads Passing through**

	No	%
1 Yes	427	94.05
2 No	27	5.95
Total	454	100.00

- **jk002 : Social Economic Status of This V/C**

	No	%
1 1	21	4.66
2 2	56	12.42
3 3	89	19.73
4 4	149	33.04
5 5	84	18.63
6 6	46	10.20
7 7	6	1.33
Total	451	100.00

- **jk003 : The Degree of Tidiness of the Road in this V/C**

	No	%
1 1	23	5.09
2 2	57	12.61
3 3	93	20.58
4 4	104	23.01
5 5	102	22.57
6 6	55	12.17
7 7	18	3.98
Total	452	100.00

- **jk004 : Construction Structure of this V/C**

	No	%
1 1	60	13.30
2 2	70	15.52
3 3	92	20.40
4 4	102	22.62
5 5	62	13.75
6 6	46	10.20
7 7	19	4.21
Total	451	100.00

- **jk005 : The Degree of Crowdness of this V/C**

	No	%

1 1	12	2.65
2 2	42	9.29
3 3	53	11.73
4 4	95	21.02
5 5	96	21.24
6 6	94	20.80
7 7	60	13.27
Total	452	100.00

- **jk006 : The Degree of Handicapped Access**

	No	%
1 1	240	53.22
2 2	64	14.19
3 3	46	10.20
4 4	55	12.20
5 5	34	7.54
6 6	6	1.33
7 7	6	1.33
Total	451	100.00

- **jk007 : The Degree of Mandarin Fluency**

	No	%
1 1	41	9.07
2 2	68	15.04
3 3	92	20.35
4 4	75	16.59
5 5	60	13.27
6 6	64	14.16
7 7	52	11.50
Total	452	100.00

14 Weight

- **communityID : Community ID**

A String Variable	
OBS:	18,245

- **householdID : Household ID**

A String Variable	
OBS:	18,245

- **ID : Individual ID**

A String Variable	
OBS:	18,245

- **HH_weight : Household Weight without Non-response Adjustment**

Mean	Min	Max	OBS
22,902.2	7,772.4	500,653.2	18,245

- **HH_weight_ad1 : Household Weight with Non-response Adjustment**

Mean	Min	Max	OBS
29,574.9	9,374.2	1,001,306.4	18,245

- **ind_weight : Individual Weight without Non-response Adjustment**

Mean	Min	Max	OBS
24,679.6	7,772.4	500,653.2	18,244

- **ind_weight_ad1 : Individual Weight with Household Non-response Adjustment**

Mean	Min	Max	OBS
31,790.6	9,374.2	1,001,306.4	18,244

- **ind_weight_ad2 : Individual Weight with Household and Individual Non-response Adjustment**

Mean	Min	Max	OBS
31,753.6	9,378.4	1,032,705.3	17,708

- **bio_weight1 : Biomarker Weight with Household Non-response Adjustment**

Mean	Min	Max	OBS
38,484.6	10,358.3	3,332,315.5	13,974

- **bio_weight2 : Biomarker Weight with Household and Individual Non-response Adjustment**

Mean	Min	Max	OBS
38,944.8	10,408.8	3,378,585.8	13,974

- **iyear : Interview Year**

	No	%
2011	17,898	98.10
2012	347	1.90
Total	18,245	100.00

- **imonth : Interview Month**

	No	%
01	150	0.82
02	110	0.60
03	87	0.48
06	458	2.51
07	8,811	48.29
08	6,941	38.04
09	513	2.81
10	287	1.57
11	426	2.33
12	462	2.53
Total	18,245	100.00

- **urID : Interviewer ID**

	No	%
043	117	0.64
060	78	0.43
064	99	0.54
066	4	0.02
083	17	0.09
093	39	0.21
094	87	0.48
164	2	0.01
194	51	0.28
195	43	0.24
196	38	0.21
197	70	0.38
198	87	0.48
199	99	0.54
200	75	0.41
201	81	0.44
202	39	0.21
203	31	0.17
204	84	0.46
205	112	0.61
206	52	0.29
207	108	0.59
208	26	0.14
209	52	0.29
210	66	0.36
211	81	0.44
212	69	0.38
213	67	0.37
214	78	0.43
215	90	0.49
216	79	0.43
217	71	0.39
218	48	0.26
219	66	0.36
220	78	0.43
221	56	0.31
222	61	0.33
223	52	0.29
224	109	0.60
225	93	0.51
227	84	0.46
228	58	0.32
229	51	0.28
230	78	0.43
231	66	0.36
232	92	0.50
234	17	0.09
235	12	0.07
236	128	0.70
237	154	0.84
238	65	0.36

239	73	0.40
240	80	0.44
241	54	0.30
242	56	0.31
243	56	0.31
244	56	0.31
245	64	0.35
247	48	0.26
248	103	0.56
249	54	0.30
250	65	0.36
251	7	0.04
252	3	0.02
253	68	0.37
254	63	0.35
255	49	0.27
257	78	0.43
258	90	0.49
259	78	0.43
260	23	0.13
261	35	0.19
262	70	0.38
263	167	0.92
264	38	0.21
265	46	0.25
266	66	0.36
267	68	0.37
268	71	0.39
269	79	0.43
270	101	0.55
271	75	0.41
272	130	0.71
273	76	0.42
274	86	0.47
276	60	0.33
277	59	0.32
278	89	0.49
279	64	0.35
280	54	0.30
281	57	0.31
282	71	0.39
283	56	0.31
284	73	0.40
285	153	0.84
286	44	0.24
287	42	0.23
288	74	0.41
289	66	0.36
290	57	0.31
291	72	0.39
292	80	0.44
293	79	0.43

294	72	0.39
295	73	0.40
296	98	0.54
297	68	0.37
298	81	0.44
299	78	0.43
300	64	0.35
301	49	0.27
302	57	0.31
303	54	0.30
304	33	0.18
305	35	0.19
306	65	0.36
307	89	0.49
308	68	0.37
309	39	0.21
310	57	0.31
311	55	0.30
312	67	0.37
313	57	0.31
314	49	0.27
315	31	0.17
316	43	0.24
317	69	0.38
318	74	0.41
319	58	0.32
320	59	0.32
321	66	0.36
322	63	0.35
323	9	0.05
324	17	0.09
325	33	0.18
326	34	0.19
327	70	0.38
328	69	0.38
329	61	0.33
330	73	0.40
331	88	0.48
332	79	0.43
333	58	0.32
334	41	0.22
336	97	0.53
337	53	0.29
338	60	0.33
339	37	0.20
340	72	0.39
341	71	0.39
342	56	0.31
343	62	0.34
344	30	0.16
345	57	0.31
346	68	0.37

347	58	0.32
348	56	0.31
349	51	0.28
350	64	0.35
351	66	0.36
352	61	0.33
353	25	0.14
354	24	0.13
357	43	0.24
358	58	0.32
359	100	0.55
360	108	0.59
361	98	0.54
362	90	0.49
363	22	0.12
364	9	0.05
365	17	0.09
366	17	0.09
367	78	0.43
368	75	0.41
369	57	0.31
370	54	0.30
371	56	0.31
372	43	0.24
373	58	0.32
374	41	0.22
376	55	0.30
377	34	0.19
378	14	0.08
379	61	0.33
380	71	0.39
381	46	0.25
382	43	0.24
383	99	0.54
384	49	0.27
385	61	0.33
386	58	0.32
387	37	0.20
388	59	0.32
389	57	0.31
390	70	0.38
391	53	0.29
392	10	0.05
393	15	0.08
394	53	0.29
395	71	0.39
396	63	0.35
397	80	0.44
400	68	0.37
401	54	0.30
402	12	0.07
403	35	0.19

405	50	0.27
406	57	0.31
407	88	0.48
408	87	0.48
410	51	0.28
411	79	0.43
412	77	0.42
413	54	0.30
416	59	0.32
417	63	0.35
418	75	0.41
421	109	0.60
422	111	0.61
423	49	0.27
424	53	0.29
425	83	0.45
426	70	0.38
427	59	0.32
428	43	0.24
429	50	0.27
430	47	0.26
431	58	0.32
432	61	0.33
433	53	0.29
434	59	0.32
435	67	0.37
436	61	0.33
437	67	0.37
438	58	0.32
439	66	0.36
440	69	0.38
442	51	0.28
443	21	0.12
444	20	0.11
445	43	0.24
446	41	0.22
447	7	0.04
449	78	0.43
450	80	0.44
451	52	0.29
452	52	0.29
453	47	0.26
454	38	0.21
458	27	0.15
459	70	0.38
460	70	0.38
461	51	0.28
462	52	0.29
463	35	0.19
464	38	0.21
465	46	0.25
466	27	0.15

467	64	0.35
468	50	0.27
469	87	0.48
470	66	0.36
471	51	0.28
472	65	0.36
473	71	0.39
474	67	0.37
475	73	0.40
476	69	0.38
477	116	0.64
478	62	0.34
479	22	0.12
480	19	0.10
481	46	0.25
482	50	0.27
483	47	0.26
484	67	0.37
485	61	0.33
486	65	0.36
487	37	0.20
488	209	1.15
489	71	0.39
490	46	0.25
493	58	0.32
494	67	0.37
495	64	0.35
496	27	0.15
497	63	0.35
498	42	0.23
510	42	0.23
511	32	0.18
601	72	0.39
602	30	0.16
603	23	0.13
604	53	0.29
616	4	0.02
617	17	0.09
618	4	0.02
619	5	0.03
620	1	0.01
621	15	0.08
622	13	0.07
624	6	0.03
625	21	0.12
626	15	0.08
627	8	0.04
628	48	0.26
629	9	0.05
630	36	0.20
631	14	0.08
632	55	0.30

633	2	0.01
634	29	0.16
637	1	0.01
Total	18,245	100.00

- **mainr : Main R or Spouse**

	No	%
Spouse	7,988	43.78
MainR	10,257	56.22
Total	18,245	100.00

15 PSU

- **communityID : Community ID**

A String Variable	
OBS:	450

- **province : Province Name**

Chinese Character String

- **city : City Name**

Chinese Character String

- **urban_nbs : Urban or Rural according to NBS**

	No	%
Rural	237	52.67
Urban	213	47.33
Total	450	100.00

- **areatype : Area Type according to NBS**

	No	%
111	96	21.33
112	18	4.00
121	62	13.78
122	34	7.56
123	3	0.67
210	14	3.11
220	223	49.56
Total	450	100.00
